# An Integrative Developmental Genomics and Systems Biology Approach to Identify an In Vivo Sox Trio-Mediated Gene Regulatory Network in Murine Embryos

**DOI:** 10.1155/2017/8932583

**Published:** 2017-05-28

**Authors:** Wenqing Jean Lee, Sumantra Chatterjee, Sook Peng Yap, Siew Lan Lim, Xing Xing, Petra Kraus, Wenjie Sun, Xiaoming Hu, V. Sivakamasundari, Hsiao Yun Chan, Prasanna R. Kolatkar, Shyam Prabhakar, Thomas Lufkin

**Affiliations:** ^1^Genome Institute of Singapore, 60 Biopolis Street, Singapore 138672; ^2^McKusick-Nathans Institute of Genetic Medicine, Johns Hopkins University School of Medicine, 733 N. Broadway, Baltimore, MD 21205, USA; ^3^Apta Biosciences Pte Ltd, 31 Biopolis Way, Singapore 138669; ^4^Department of Biology, Clarkson University, 8 Clarkson Avenue, Potsdam, NY 13699, USA; ^5^The Jackson Laboratory for Genomic Medicine, 10 Discovery Drive, Farmington, CT 06030, USA; ^6^Qatar Biomedical Research Institute, Hamad Bin Khalifa University, Qatar Foundation, P.O. Box 5825, Doha, Qatar

## Abstract

Embryogenesis is an intricate process involving multiple genes and pathways. Some of the key transcription factors controlling specific cell types are the* Sox* trio, namely,* Sox5*,* Sox6*, and* Sox9*, which play crucial roles in organogenesis working in a concerted manner. Much however still needs to be learned about their combinatorial roles during this process. A developmental genomics and systems biology approach offers to complement the reductionist methodology of current developmental biology and provide a more comprehensive and integrated view of the interrelationships of complex regulatory networks that occur during organogenesis. By combining cell type-specific transcriptome analysis and in vivo ChIP-Seq of the Sox trio using mouse embryos, we provide evidence for the direct control of* Sox5* and* Sox6* by the transcriptional trio in the murine model and by Morpholino knockdown in zebrafish and demonstrate the novel role of* Tgfb2*,* Fbxl18*, and* Tle3* in formation of* Sox5*,* Sox6*, and* Sox9* dependent tissues. Concurrently, a complete embryonic gene regulatory network has been generated, identifying a wide repertoire of genes involved and controlled by the Sox trio in the intricate process of normal embryogenesis.

## 1. Introduction

The Sox family of proteins that are encoded by at least 20 genes in mammals plays a myriad of roles during embryonic cell type specification and organogenesis [1–8]. Three of the* Sox* genes,* Sox5*,* Sox6*, and* Sox9*, while having unique roles in specific cell types, together play a concerted role during embryonic skeletogenesis, being absolutely necessary for proper chondrogenesis [9–17]. The skeleton is a complex system, fulfilling critical functions such as movement, hematopoiesis, and the protection of vital organs. The incidence of skeletal diseases is estimated to be 1 in 4000 births with half of them being early lethal but the real frequency is probably higher as some disorders develop later in life [18]. Genes controlling skeletogenesis are often involved in other developmental processes, resulting in complex syndromes with skeletal disorders being one of the outcomes. To develop greatly needed therapies for human diseases, it is necessary to understand the molecular basis behind them. Deciphering the fundamentals of skeletogenesis will aid in the identification of the molecular mechanisms behind the prevalent skeletal diseases.

Chondrocytes are the first skeleton-specific cell type to appear during development and defects in chondrogenesis; an essential event during endochondral ossification (responsible for the formation of most of the skeleton) and postnatal cartilage maintenance lead to chondrodysplasias and osteoarthritis [19, 20]. The commitment of progenitor cells to the chondrogenic fate is largely determined by the expression of the transcription factor,* Sox9 *[21]. Heterozygous mutations in and around* Sox9* were shown to cause campomelic dysplasia, a severe form of human chondrodysplasia often accompanied by male sex reversal and defects in other nonskeletal organs, highlighting its critical role in chondrogenesis and other tissues [22, 23]. Early chondrocyte differentiation and subsequent maturation are controlled by* Sox9* and its family members,* Sox5* and* Sox6 *[24, 25]. The expression of the trio in nonchondrogenic cells has been shown to induce cartilage formation [26].* Sox5* and* Sox6* which are highly related to each other are expressed downstream of* Sox9 *[27]. They exhibit functional redundancy for each other during chondrogenesis with the double-gene knockout mice displaying severe chondrodysplasia as compared to the single knockouts, which showed mild skeletal defects [28]. Recent molecular studies done in vitro have shown that the trio synergistically activates the expression of cartilage-specific genes such as* Col2a1*,* Acan*, and* Matn1 *[29, 30].

The genetic and molecular events underlying a biological process such as chondrogenesis are often complex and the complete understanding of the gene regulatory network (GRN) involved requires a comprehensive developmental genomics and systems biology approach [31].* Sox9*,* Sox5*, and* Sox6* were previously found to be the major transcription factors regulating chondrogenesis [27]. The targets of the Sox trio in chondrogenesis uncovered by conventional experimental approaches previously mentioned are probably only a portion of the network involved and only partially recapitulate what is happening in vivo. In order to have a comprehensive view of what is occurring during skeleton development, classical mouse genetics in combination with expression profiling and chromatin immunoprecipitation-sequencing (ChIP-Seq) were carried out for* Sox9*,* Sox5*, and* Sox6 *using E13.5 mouse embryos, a stage representing the second stage of chondrocyte differentiation preceding the transition of chondrocytes into hypertrophic chondrocytes [27, 32]. Though the roles of these three proteins have been studied at E13.5 previously with their individual targets [27, 28, 33, 34], there is presently no comprehensive systems biology in vivo gene list established for the Sox trio at this stage. Here we report the identification of direct and indirect targets of the transcriptional Sox trio in vivo, generating an extensive GRN at E13.5, separating cohorts of genes regulated by Sox9 alone or together with Sox5 and/or Sox6. We have also verified* Tgfb2*,* Tle3*, and* Fbxl18* as novel chondrogenic targets regulated by the Sox trio. The modes of action of the Sox trio and the discovery of their additional target genes in this valuable dataset have increased the understanding of the molecular mechanisms behind postmesenchymal condensation processes in chondrogenesis.

## 2. Materials and Methods

### 2.1. Generation of Transgenic Mice

BAC clones, RP24-248D4, RP23-82L9, and RP23-403L18 containing regions of the genome for* Sox9*,* Sox5*, and* Sox6*, respectively, were ordered from BACPAC, Resources Centre, CHORI, Oakland, USA (http://bacpac.chori.org) [35, 36]. BAC modification to create the transgenes was done using the Quick and Easy BAC Modification Kit (Gene Bridges) according to the manufacturer's protocol. The modified BACS were then subcloned to the pBSSK+ (Stratagene) or minimal vector (Gene Bridges) as targeting vectors for homologous recombination in ESC. All vectors used were verified by restriction enzyme mapping and sequencing [37, 38]. Correctly modified ESC lines were microinjected into 2- to 8-cell stage mouse embryos to generate chimeric embryos and chimeric mice that were subsequently used to generate mouse colonies [39]. The* Neo *cassettes in the transgene were removed by crossing with mice expressing Flpe recombinase driven by the* ROSA26* promoter (Stock number 3946, The Jackson Lab) or Cre recombinase driving by the* Zp3* promoter as previously described [40, 41]. All animal procedures were conducted according to IACUC guidelines. Further details can be found in Supplementary Material available online at https://doi.org/10.1155/2017/8932583.

### 2.2. Ethical Statement

All animal procedures were performed according to the Singapore A^*∗*^STAR Biopolis Biological Resource Center (BRC) Institutional Animal Care and Use Committee (IACUC) guidelines which are set by the National Advisory Committee for Laboratory Animal Research (NACLAR) for the ethical treatment of animals. The IACUC protocols employed were reviewed and approved by the aforementioned committee before any animal procedures were undertaken for this study described here (IACUC Protocol numbers 110689 and 110648). The mouse strains used in this study were provided, housed, and maintained in IVC cages by the A^*∗*^STAR Biopolis Biological Resource Center following the aforementioned guidelines for the ethical treatment of animals.

### 2.3. Gene Targeting in ESC

V6.4 and R1 mouse ESC were used for gene targeting. Briefly, they were cultured at 37°C with 5% CO2 on gelatin-coated feeder plates with culture medium made from DMEM (Gibco) supplemented with 15% heat-inactivated ES-grade fetal bovine serum (FBS) (Gibco), 500 U/ml LIF (Chemicon), 0.1 mM *β*-mercaptoethanol (Invitrogen), 4 mM L-glutamine (Invitrogen), 1 mM sodium pyruvate (Invitrogen), and 40 *µ*g/ml gentamicin (Sigma) [42, 43]. 10–15 *µ*g of the linearized vector was used for each electroporation at 125 *µ*F, 0.4 kV into approximately 10 million ESC. Individual G418-resistant ESC colonies were picked after 10 days of selection with 200–400 *µ*g/ml G418 and screened by Southern blot analysis.

### 2.4. Genotyping

Mouse ESC, tail biopsies, and embryonic tissues were digested with Proteinase K (Sigma). Genomic DNA was extracted using phenol-chloroform followed by ethanol precipitation and used for genotyping [44]. All modified ESC and founder mice from the transgenic lines were confirmed with Southern blot analysis. Southern blot analysis was carried out as previously described [40] using 10–20 *μ*g of genomic DNA digested with the indicated restriction enzyme. Routine genotyping was done by PCR [45].

### 2.5. FACS

E13.5 mouse embryos were harvested in ice-cold Leibovitz's L-15 (Gibco). EGFP-expressing embryos were identified using a fluorescent dissection microscope (Leica) and dissected such that all internal organs were removed, leaving the axial and appendicular skeleton intact. Tissue dissociation into single cells was carried out by mechanical pipetting using an enzymatic solution comprising 100 U/ml Collagenase I and Collagenase II (Invitrogen), 50 U/ml DNase I (Invitrogen), and 0.05% Trypsin (Invitrogen) in Leibovitz's L15. The enzymes were stopped by the addition of 20% FBS (Gibco) in Leibovitz's L15. The dissociated cells were filtered through a 100 *μ*M followed by a 40 *μ*M cell strainer. Cells were pelleted down and resuspended in 5% FBS, 4 mM EDTA in Leibovitz's L15 for sorting using a 4-laser FACSAria Cell Sorter (BD Biosciences).

### 2.6. Microarrays

Total RNA was isolated from the sorted cells using the RNeasy Micro Kit (Qiagen). The RNA extracted from all the samples was quantified using Ribogreen (Invitrogen) and their RNA integrity was assessed to ensure the high quality using the Bioanalyzer (Agilent Technologies). For the* Sox9* microarray, 50 ng of total RNA per sample was labeled using the Illumina TotalPrep RNA Amplification Kit (Ambion) and hybridized on the MouseWG-6 v1.1 Expression BeadChip (Illumina). For the* Sox5/Sox6* microarray, 25 ng of total RNA per sample was labeled using the TargetAmp-Nano Labeling Kit for Illumina Expression BeadChips (Epicentre) and hybridized on the MouseWG-6 v2.0 Expression BeadChip (Illumina). Microarray hybridization was carried out according to Illumina's guidelines. The microarray data can be found in GEO under the accession number GSE33173.

### 2.7. Microarray Data Analysis

Raw data was extracted with background subtraction employed using Genome Studio (Illumina). Further analysis was done with Genespring 11 (Agilent). log2 transformation and percentile shift normalization at 75th percentile were performed. Default detection *p* value range as suggested by the software for present, marginal, and absent flags was used. Entities with present and marginal flags were further filtered by the percentile of their expression using the default settings before statistical analysis. Unpaired *t*-test with the Benjamini-Hochberg multiple test correction was performed for the* Sox9* microarray and the* Sox5/6* microarray, respectively. A corrected *p* value cutoff of 0.05 with a minimum fold change of 1.5 was used to find the differentially expressed probes.

### 2.8. Histology

Mouse embryos were fixed overnight at 4°C in 4% paraformaldehyde and processed as previously described [46, 47]. RNA SISH was carried out with 10 *μ*m paraffin-embedded sections as previously described [40, 48]. The following cDNAs were used as templates for synthesizing the antisense probes: 0.5 kb* Col2a1* [46]; 0.7 kb* Matn4* (IMAGE Clone ID: 1366191); 4.7 kb* Hapln1* (IMAGE Clone ID: 30430221); 0.5 kb* 3110079O15Rik* (IMAGE Clone ID: 40104070); 0.6 kb* Sox5* (IMAGE Clone ID: 40047865); and 0.5 kb* Sox9* (Sox9 exon 1 sequence from BAC clone RP24-248D4). Alcian Blue staining was performed as described [46, 47, 49]. Crossing of the* Col2a1-Cre* transgenic line (Stock number 3554, The Jackson Lab) with the conditional* Sox9* knockout line was performed as described [50]. All sections were photographed using Zeiss Axio Imager Z1.

### 2.9. ChIP-Seq and Peak Calling

Tissue from limbs and tails of E13.5 CD1 wildtype embryos were used for Sox6 and Sox9 ChIP. The same tissues were dissected from E13.5 *Sox*5^*HA*/*HA*^ embryos for the Sox5 ChIP. Crosslinking, chromatin isolation, sonication, and immunoprecipitation were carried out as previously described [51]. Preclearing was done with rabbit or goat IgG (sc-2027 and sc-2028, Santa Cruz). Immunoprecipitation was done using an anti-Sox9 antibody (AF3075, R&D Systems), anti-Sox6 antibody (ab30455, Abcam), and anti-HA antibody (AP09230PU-N, Acris). All antibodies employed have been shown to be specific for their specific target protein and not to cross-react with any other protein (e.g., Figure S1). 10–15 ng of purified ChIP DNA from each sample was used to synthesize the sequencing library as instructed by the ChIP-Seq DNA sample Prep Kit (Illumina). The libraries were then subjected to the Solexa sequencing according to Illumina's instruction. Sequence reads produced by the Illumina Genome Analyzer II/IIx that passed the signal purity filtering were mapped to the mouse genome mm9, using the Illumina Genome Analyzer Pipeline. All uniquely mapped reads that are with two or fewer mismatches were retained. Genomic binding sites in the ChIP-Seq datasets were identified using the peak calling algorithm MACS (version 1.4.0 beta) with default settings (band width = 300, model fold = 10, 30, *p* value cutoff = 1.00*e* − 05, and range for calculating regional lambda = 1000 and 10000 bps) [52]. The corresponding control libraries were used for all the peak callings. The datasets can be found at GEO under the accession number GSE33419.

### 2.10. Motif Analysis

Briefly, binding sites within 1 kb upstream and downstream of the transcription start site (TSS) of the gene were considered in the TSS region. Binding sites 1–5 kb upstream of the TSS were considered to be in the promoter region. Intragenic binding sites were defined as being within the gene but out of the TSS and promoter region. Proximal binding sites were binding sites out of the gene but within 5–10 kb upstream of the TSS and 1–10 kb downstream of the TSS. Distal binding sites were defined as 10–100 kb upstream and downstream of the TSS and out of the gene. Other binding sites found more than 100 kb away from the TSS and out of the gene were classified as others. Peaks called by MACS were ranked according to the total tags count as defined in the MACS output file. The top 200 peaks were used for motif analysis and the repeat masked genome sequences +/−50 bp from the summit of these 200 peaks were downloaded from the UCSC genome browser (http://genome.ucsc.edu/). After masking repeats to *N*, we performed de novo motif finding using MEME ver. 4.3.0 with the sequences. MAST was used to scan for the occurrences of the primary de novo motif obtained using all the sequences +/−50 bp of the ChIP-Seq peak summit. The cutoff for motif match in MAST used was the default *p* = 1.0*e* − 4.

### 2.11. Zebrafish Assays

Fish were maintained in the GIS zebrafish facility according to the standard of IACUC guidelines. The stages of embryos were indicated as hpf (hours after fertilization) or dpf (days after fertilization) [53]. Morpholinos were injected into about 200 1-cell stage embryos at a concentration of 1.2 picomolar. To reduce cell death due to off-target effects 1.8 picomolar of p53 Morpholino was coinjected as previously described [54]. Embryos were maintained in a 28°C incubator overnight and dead embryos were removed the next morning before scoring for morphants. Alcian Blue staining was performed on 24 hpf wildtype and morphant embryos and embryos were sectioned in a transverse orientation as previously described [38, 54]. All sections were imaged with the Zeiss Axio Imager.

### 2.12. Luciferase Assay

Using an oligo cloning strategy, the binding site sequences were cloned between the* BamHI* and* XhoI* sites of the pGL4.23 luciferase vector (Promega Corporation, USA). NIH/3T3 or HEK293 cells (5-6 × 10^4^ cells/well) were cultured in DMEM supplemented with 10% FBS and 40 *µ*g/ml of gentamycin sulphate. For the assays conducted for the Sox9 binding sites alone, cells were transfected with 500 ng of luciferase vector containing the binding site, 300 ng of Sox9 expression vector, and 5 ng of Renilla luciferase vector (transfection control) using 6 *μ*l of FuGENE HD transfection reagent (Roche Diagnostic, USA), in 100 *μ*l of OPTI-MEM I medium (Invitrogen, USA). For the assays validating the Sox trio binding sites, 300 ng of luciferase vector containing the binding sites, 200 ng of each of Sox9, Sox5, and Sox6 expression vectors, and 2 ng of Renilla luciferase vector (transfection control) using 6 *µ*l of FuGENE HD transfection reagent (Roche Diagnostic, USA), in 100 *µ*l of OPTI-MEM I medium (Invitrogen, USA), were transfected into cells. The ratios between the different vectors were the same for all transfections except where indicated. The cells were grown for 48 hours and the luminescence was measured using Dual Luciferase Reporter Assay System on a Glomax Multidetection System Luminometer as per manufacturer's instructions (Promega Corporation).

### 2.13. GO Analysis and GRN Construction

GO analysis was carried out using DAVID [55]. The differential genes were ranked based on expression fold change and if the number of genes analyzed exceeded 3000, the 3000 genes with the highest fold change were used for GO analysis. The GO-terms for biological process were filtered using the corrected *p* value < 0.05 adjusted by the Benjamini-Hochberg multiple test correction and ranked according to fold enrichment as determined by DAVID. The Sox trio-mediated GRN was generated using Cytoscape [56].

## 3. Results

### 3.1. In Vivo Identification of the Genes Involved in the Sox Trio-Associated Functions

**Figure 1:**
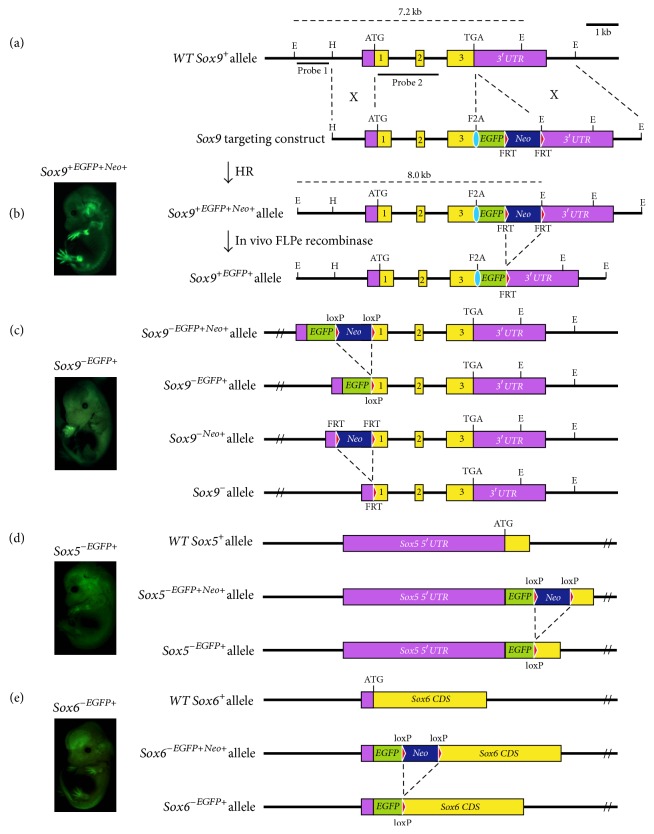
*Sox9*,* Sox5*, and* Sox6* gene targeting and their E13.5 transgenic embryos generated for FACS and expression Profiling. (a) Wildtype* Sox9* allele (*Sox9*^*+*^) is indicated with exons depicted as black boxes. Translation start site (ATG) as indicated. (b)* Sox9* with the* F2A-EGFP-FRTNeo* inserted in exon 3 (*Sox*9^+(EGFPNeo)^) and after FLPe recombination (*Sox*9^+(EGFP)^). *Sox*9^+/+(EGFP)^ E13.5 embryo shown on the right. (c)* Sox9-null *allele with the* EGFP-loxPNeo* inserted after the ATG (*Sox*9^−(EGFPNeo)^) and after CRE recombination (*Sox*9^−(EGFP)^).* Sox9-null *allele with* FRTNeo* inserted in exon 1 (*Sox*9^−(Neo)^) and after FLPe recombination (*Sox*9^−^). *Sox*9^−/−(EGFP)^ E13.5 chimeric embryo shown on the right. (d) Exon containing the ATG of wildtype* Sox5* (*Sox5*^*+*^) shown.* EGFP-loxPNeo* inserted after the ATG (*Sox*5^−(EGFPNeo)^) and after CRE recombination (*Sox*5^−(EGFP)^). *Sox*5^+/−(EGFP)^ E13.5 embryo shown on the right. (e) Exon containing the ATG of wildtype* Sox6* (*Sox6*^*+*^) shown.* EGFP-loxPNeo* inserted after the ATG (*Sox*6^−(EGFPNeo)^) and after CRE recombination (*Sox*6^−(EGFP)^). *Sox*6^+/−(EGFP)^ E13.5 embryo shown with a wildtype embryo (*WT*) under same lighting condition. EGFP, green boxes; F2A, blue ovals; FRT sites, red triangles; loxP sites, yellow triangles (see also Figure S1 and Table S1).

To isolate the* Sox9-*expressing cells directly from developing mouse embryos for in vivo comparative expression profiling, the endogenous* Sox9* locus was targeted with the* enhanced green fluorescent protein (EGFP)* reporter gene via homologous recombination, generating* Sox9-wildtype (Sox*9^+/+(EGFP)^) and* Sox9-null (Sox*9^−/−(EGFP)^) gene targeted lines (Figures 1(a)–1(c)) for fluorescence-activated cell sorting (FACS). *Sox*9^+/+(EGFP)^ was made with the reporter linked via the foot-and-mouth disease virus 2A peptide (F2A) at the 3′ end of the* Sox9* locus (Figure 1(b)), forming a bicistronic system with a single open reading frame [45, 57]. The *Sox*9^+/+(EGFP)^ mice generated were viable and fertile, indicating that the gene targeted mice are normal given the lethality of the* Sox9* heterozygous mutation. Western blot analysis using E13.5 *Sox*9^+/+(EGFP)^ and E13.5 wildtype embryos verified that the Sox9 and EGFP proteins were expressed individually and Sox9 protein levels in our *Sox*9^+/+(EGFP)^ were similar to that of the wildtype (Figures S1).* Sox9* heterozygotes mice die at birth and thus cannot be intermated to generate* Sox9 *loss-of-function animals. To overcome this problem, *Sox*9^−/−(EGFP)^ embryonic stem cells (ESC) were generated by sequential inactivation of each* Sox9* allele in ESC via gene targeting and used to generate high-percentage chimeras for FACS (Figure 1(c)). Both *Sox*9^+/+(EGFP)^ and *Sox*9^−/−(EGFP)^ embryos showed EGFP expression in the* Sox9*-expressing domains, indicating that the reporter is replicating the* Sox9* expression endogenously.

Likewise, the* Sox5* and* Sox6* loci were tagged with the* EGFP* reporter in mice for FACS. The* EGFP* reporter gene was inserted into the endogenous* Sox5 *loci after the translation start site (Figure 1(d)), disrupting the longer protein isoform of* Sox5* which is the predominant form in chondrocytes [58]. Similarly, the* EGFP* reporter gene was used to inactivate the* Sox6* allele (Figure 1(e)). Viable and fertile lines of heterozygous mice were generated from the targeted *Sox*5^+/−^ and *Sox*6^+/−^ ESC, respectively, and their phenotypes were similar to what was previously described [28]. As* Sox5* and* Sox6* are considered functionally redundant during chondrogenesis [33], the two mouse lines were crossed to generate double homozygous embryos with both* Sox5* and* Sox6 *inactivated (*Sox*5^−/−^*Sox*6^−/−^) for the transcriptome analysis.

*Sox9 *is expressed specifically during embryogenesis in a variety of organs such as the male gonad, heart, nervous system, and kidney [9].* Sox5* and* Sox6 *are expressed in the neurons, oligodendrocytes, and further cell types other than chondrocytes [59, 60]. For enriching skeletal cells expressing the* Sox *trio, the E13.5 embryos had their internal organs removed, leaving the axial and appendicular skeleton intact before dissociation into single cells for FACS. The remaining tissues present expressing the Sox trio would be from the skeletal and central nervous system. It was not possible to dissect out the central nervous system without compromising cell viability; hence the results here represent both chondrogenic and neurogenic cells types. Cells from a wildtype E13.5 embryo were used to set the gating for FACS (Figure S1). Postsort analysis of the collected EGFP-positive fraction from all genotypes routinely showed that the EGFP-expressing cells in the sorted population make up more than 95% as compared to the presorted population with 0.5–3% EGFP-expressing cells, indicating that the* Sox*-expressing cells have been successfully isolated from the nonexpressing cells (Figure S1). Total RNA from the sorted cells was then used for microarray analysis.

5742 genes were found to be differentially expressed between *Sox*9^+/+(EGFP)^ and *Sox*9^−/−(EGFP)^ (Table S1). The loss of Sox9 resulted in the upregulation of 3105 genes and downregulation of 2637 genes. Genes that were upregulated represented the putative genes that are normally repressed by Sox9 and those that are downregulated represented the putative targets activated by Sox9. RNA section in situ hybridization (SISH) was carried out on 25 differentially expressed genes identified, including that of a novel gene,* 3110079O15Rik*, and it was ascertained that their expression domains overlapped with that of* Sox9 *(Table S2). To further validate our* Sox9* microarray results, RNA SISH on some of the targets was performed using *Sox*9^+/+(EGFP)^ embryos and conditional* Sox9* knockout embryos previously described [50]. A reduction in the expression of* Col2a1*, a known Sox9 target, was observed as expected when* Sox9 *was inactivated (Figure S2). Downregulated genes such as* Matn4*,* Hapln1*, and the novel gene,* 3110079O15Rik*, were observed to have reduced expression whereas upregulated genes like* Pax1* showed an increase in expression, confirming the expression profiling results, giving rise to a list of genes associated with the function of* Sox9* at the postmesenchymal condensation stage (Figure S2) and concurrently, indicating that the experimental approach was robust.

Comparative profiling at E13.5 was carried out for the sorted cells from *Sox*5^−/−^*Sox*6^−/−^ embryos, against sorted cells from *Sox*6^+/−^ embryos, which have previously been reported to be of a wildtype phenotype [28]. In the absence of Sox5 and Sox6, a total of 4780 genes were found to be differentially expressed with 1446 genes downregulated and 3334 genes upregulated (Table S1). The fact that* Sox9* was not found to be differentially expressed validates previous findings describing* Sox9* as an upstream regulator of* Sox5* and* Sox6*, unlikely to be regulated by Sox5 and Sox6 [27].

**Figure 2:**
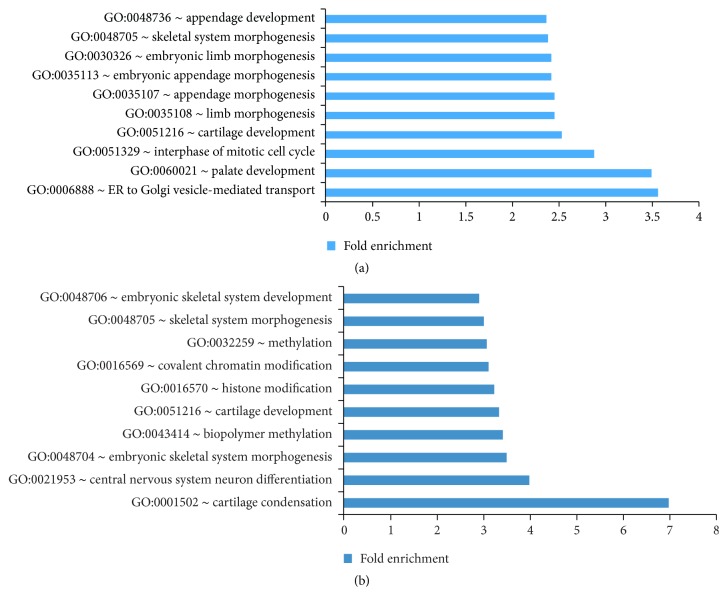
Differentially expressed genes from the* Sox9* microarray and* Sox5/Sox6* double-null microarray. (a) Top 10 GO-terms for genes that are downregulated when* Sox9* is inactivated. (b) Top 10 GO-terms for genes that are downregulated when* Sox5* and* Sox6* are inactivated (see also Figure S2 and Table S2).

To assess the major biological functions associated with the differentially expressed genes from the microarray analysis, gene ontology (GO) analysis was carried out for the upregulated and downregulated genes using the Database for Annotation, Visualization and Integrated Discovery (DAVID) [61]. The top GO-terms for biological processes that were overrepresented in the downregulated gene sets for both the* Sox9* and the* Sox5/6* microarray were enriched for terms associated with skeletogenesis such as skeleton system morphogenesis and cartilage development (Figures 2(a) and 2(b)). The top GO-terms for the upregulated genes did not show skeletal-related terms (Figure S2), supporting previous observations that the Sox trio acts as chondrogenic activators [1].

### 3.2. Tissue ChIP-Seq Shows Sox9 Acting as a Homodimer and Chondrogenic Activator

**Figure 3:**
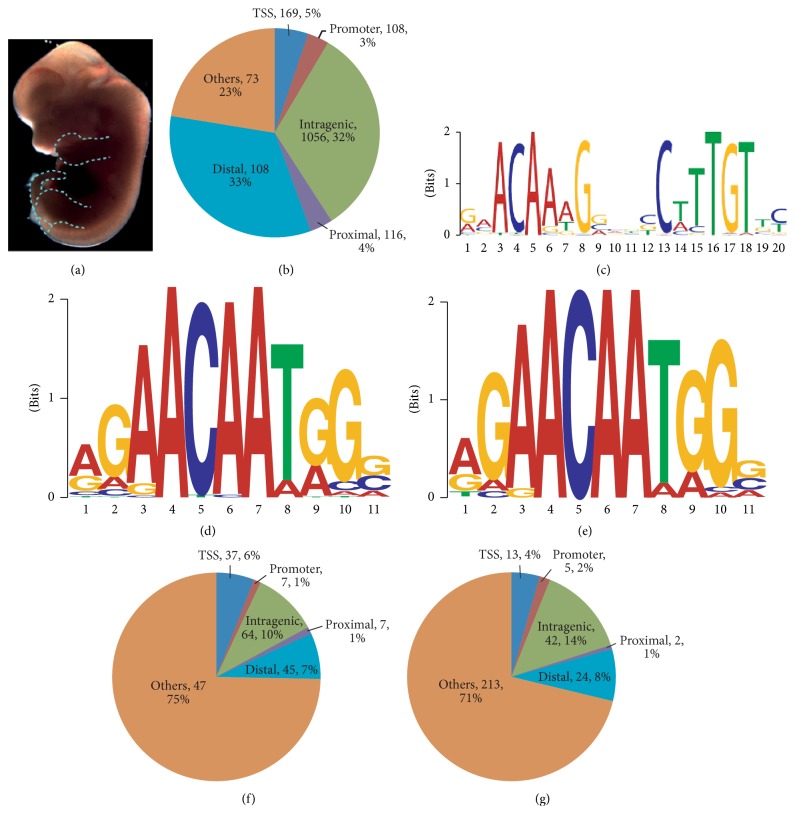
ChIP-seq results for Sox9, Sox5, and Sox6. (a) Dissected tissues of the E13.5 embryo used for ChIP are indicated by the dotted blue lines. (b) Distribution of the Sox9 binding sites with respect to their nearest genes. (c) Primary Sox9 motif found shows Sox9 acts as a homodimer with the binding sites in opposite orientation. (d) Primary motif found from the Sox5 binding profile. (e) Primary motif found from the Sox6 binding profile. (f) Distribution of the Sox5 binding sites with respect to their nearest genes. (g) Distribution of the Sox6 binding sites with respect to their nearest genes (see also Figure S3 and Table S3).

The pool of differentially expressed genes from the microarray results consisted of target genes that were regulated directly by Sox9 and other indirect target genes whose expression was altered due to the loss of Sox9 affecting their upstream regulators. To find genes involved in chondrogenesis directly controlled by Sox9, ChIP-Seq with an anti-Sox9 antibody using dissected tissues from limbs and tails of wildtype E13.5 embryos, naturally enriched for the chondrogenic lineage, was performed (Figure 3(a)), with IgG ChIP as background control. Using the model-based analysis for ChIP-Seq (MACS) [52] algorithm, 3260 peaks were identified. ChIP-quantitative PCR validation on randomly selected peaks identified from the analyzed ChIP-Seq results showed good correlation between the two datasets, indicating that the peaks called were specific (Figure S3; Table S3). To investigate the function of the binding sites, the peaks were annotated based on their positional information relative to the nearest gene (Figure 3(b)). The breakdown of the distribution of binding sites showed 77% within 100 kb of the transcription start site of a gene or within the gene itself with the majority present in distal and intragenic regions.

The Sox9 tissue ChIP-Seq identified peaks at previously characterized chondrocyte-specific enhancers in vitro such as* Col2a1* and* Col9a1 *[62, 63]. Luciferase assays were carried out on some of the identified Sox9 binding sites in or nearest to the putative target genes like* Matn4*,* Mia1*,* Hapln1*,* Acan*,* Papss2*,* Sox5*, and* Sox6*, validating the transactivation function of the binding sites (Figure S4; Table S3).

To study the in vivo Sox9 binding sequence, de novo motif finding was performed using Multiple EM for Motif Elicitation (MEME) [64] with the top 200 Sox9 peaks with the highest tags count as defined by MACS. The primary motif identified confirmed in vivo that Sox9 functions as a homodimer with the consensus Sox motifs in opposing orientation (Figure 3(c)) as reported in in vitro studies previously [65]. Motif Alignment and Search Tool (MAST), part of the MEME suite, was then used to scan for the primary motif identified in all the Sox9 binding sites. About 37% (1197 out of 3260) of the Sox9 binding sites were found to have the Sox9 primary homodimer motif with the most common spacer number of four base pairs found between them (Figure S3). The spacing requirement for Sox9 binding has been reported in in vitro experiments previously [65] and it has also been shown that certain patients with campomelic dysplasia possess a mutant Sox9 incapable of homodimerization [66]. Our study supports recent findings on genomewide surveys of the homodimer motifs and spacing in vivo [7, 8, 67, 68] reviewed in [69, 70]. Interestingly, in the study by Ohba et al. [7] they suggest that the Sox9 protein can have two different mechanisms of action, one where it interacts directly with the TSS and a second one where it binds as a homodimer to its DNA recognition motifs [7] reviewed in [69, 70].

### 3.3. Regulation of* Sox5* and* Sox6* Expression by the Sox Trio

*Sox5* and* Sox6 *are known to act downstream of* Sox9 *during chondrogenesis. Together, these three transcription factors are thought of as the chondrogenic master regulators [24, 33, 71] and Sox9 binding sites were previously found at the* Sox5 *locus in a ChIP-on-chip experiment using rat chondrosarcoma cells [72]. None have been reported for* Sox6* so far. In this study, multiple Sox9 binding sites in vivo were found in both* Sox5* and* Sox6 *loci (Figure S3).* Sox5* and* Sox6 *were both downregulated in the microarray by 5.6-fold and 2.5-fold average, respectively, when* Sox9* was inactivated. Taken together, this confirms that Sox9 activates* Sox5* and* Sox6 *expression, confirming them as direct Sox9 targets at E13.5.

A previous in vitro study showed that the DNA binding of Sox5 and Sox6 was not restricted to their consensus Sox motifs but instead they can bind to variable motifs on cartilage-specific enhancers on genes such as* Acan *[29], suggesting that in silico approaches are not the ideal way to find the binding sites. Thus, to determine the in vivo binding profile, tissue ChIP-Seq was done for Sox5 and Sox6 using E13.5 mouse embryos. Sox5 ChIP-Seq was carried out using limbs and tails from E13.5 embryos of *Sox*5^*HA*/*HA*^ mice previously described [40]. In these *Sox*5^*HA*/*HA*^ mice, only the longer isoform of Sox5, relevant in chondrogenesis, was tagged with* haemagglutinin* (HA). To demonstrate that the HA-tag was not interfering with the Sox5 protein function, the *Sox*5^*HA*/*HA*^ mice were crossed to the *Sox*5^+/−^ gene targeted line to generate *Sox*5^*HA*/−^ mice, which were indistinguishable from wildtype mice. E13.5 wildtype limbs and tails immunoprecipitated with HA antibody were used as a negative control. To determine the Sox6 binding sites, anti-Sox6 antibody was used to immunoprecipitate limbs and tails of E13.5 wildtype embryos with the IgG ChIP serving as a negative control.

A total of 632 and 299 peaks were identified for Sox5 and Sox6, respectively, by the MACS analysis. Motif analysis for the Sox5 and Sox6 ChIP-Seq dataset showed that the consensus Sox motif 5′-(A/T)(A/T)CAA(A/T)G-3′ [73] was enriched (Figures 3(d) and 3(e)), highlighting the robustness of the in vivo ChIP-Seq. The peaks were then annotated with respect to the nearest genes and classified as described for the Sox9 ChIP library (Figures 3(f) and 3(g)). The distribution of the binding sites found in the TSS and the promoter region of the gene for the Sox5 and Sox6 ChIP library was similar to those of the Sox9 ChIP library. It was observed that most of the Sox5 and Sox6 binding sites were not found in the proximity of the genes unlike what was seen for the Sox9 binding sites. In particular, multiple Sox5 and Sox6 binding sites were found in their loci (Figure S3), suggesting that there is a self-regulatory feedback mechanism in addition to the Sox9 regulation. Complementary although not identical results to those described here have previously been described [67] and reviewed in [69, 70].

### 3.4. Genes That Are Directly Regulated by the Sox Trio

To find genes that are regulated by Sox9, Sox5, and Sox6, the differentially expressed genes from the* Sox9* microarray were overlapped with the Sox9 ChIP-Seq library and the differentially expressed genes from the* Sox5/6* microarray were overlapped with the Sox5 and Sox6 ChIP-Seq data. 490 genes, 113 genes, and 52 genes were found to be controlled by Sox9, Sox5, and Sox6, respectively (Table S4).

**Figure 4:**
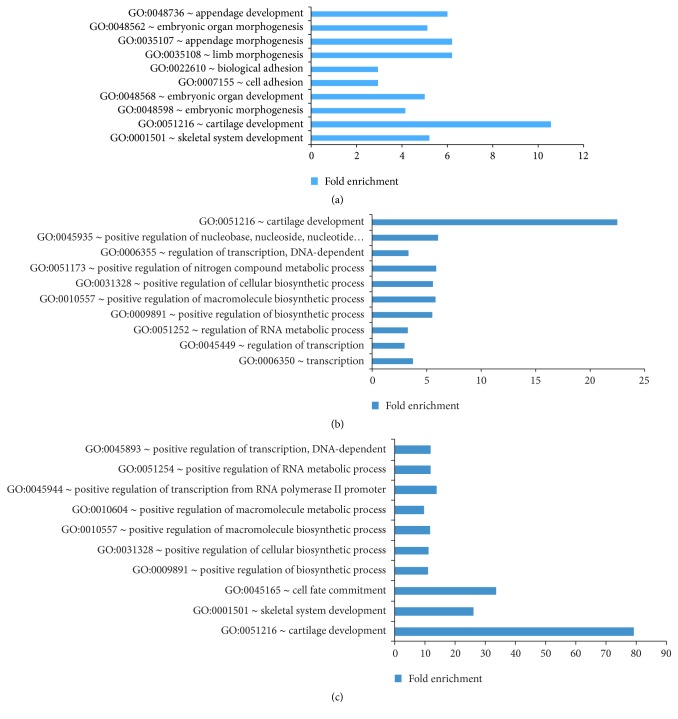
(a) Top 10 GO-terms for genes that are activated by Sox9. (b) Top 10 GO-terms for genes that are activated by Sox5. (c) Top 10 GO-terms for genes that are activated by Sox6 (see also Figure S4 and Table S4).

More than two-thirds of the 490 genes with Sox9 binding sites were downregulated when* Sox9* was inactivated, reiterating that Sox9 is more of an activator for gene expression rather than a repressor during this stage [27, 74]. To assess the direct targets of Sox9 in relation to their biological relevance, GO analysis was used. The upregulated genes when Sox9 was inactivated did not have any GO-terms with a *p* value < 0.05. The downregulated genes, however, were clearly enriched for skeletal-related genes with the GO-term skeletal system development and cartilage development topping the list (Figure 4(a)), indicating that Sox9 activates skeletal genes in vivo and that the dataset generated was robust and specific.

More genes were found to be upregulated rather than downregulated by Sox5 and Sox6 during the overlap, opposing the trend observed for* Sox9*. The downregulated genes during Sox5 inactivation with Sox5 binding sites were overrepresented by GO-terms for transcription and cartilage development (Figure 4(b)), showing that Sox5 activates cartilage genes. Similarly, genes shown to be activated by Sox6 were enriched for cartilage development and skeletal system development (Figure 4(c)). Despite the larger number of upregulated genes for both Sox5 and Sox6, which overlap, none of the GO-terms has a significant adjusted *p* value < 0.05.

### 3.5. *Fbxl18*,* Tgfb2*, and* Tle3* Are Targets of the Sox Trio

**Figure 5:**
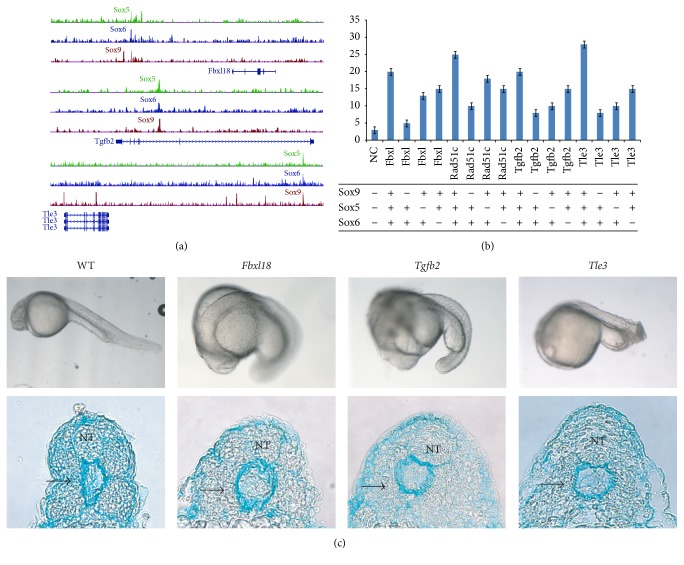
Genes regulated by the Sox trio. (a) Binding profile of Sox9, Sox5, and Sox6 for* Fbxl18*,* Tgfb2*, and* Tle3*. (b) Transactivation assay for the Sox binding sites found for* Fbxl18*,* Tgfb2*, and* Tle3*. Different Sox proteins were transfected as indicated with the enhancers containing the Sox trio binding sites and the luciferase activity was then measured. (c) Top panel shows the whole mount picture of the 24 hpf zebrafish embryos injected with the Morpholinos for* Fbxl18*,* Tgfb2*, and* Tle3* along with the wildtype (WT) injected with the scramble Morpholino as a negative control. Bottom panel shows the respective transverse sections of the Morpholino-injected embryos stained with Alcian Blue. The WT embryo shows normal neural tube (NT) and a well-defined emerging sclerotome (black arrow). The morphants at the same stage show extensive midline phenotype with defects in both neural tube and the sclerotome as seen by reduced Alcian Blue staining and loss of structural integrity (see also Figure S5 and Table S6).

As observed earlier, a large portion of the Sox5 and Sox6 binding sites was present more than 100 kb away from the TSS or outside the gene. To understand the biological relevance of these binding sites, another approach was taken in which differentially expressed genes on both sides of the Sox5, Sox6, and Sox9 binding sites were considered (Table S5). Previously, only the nearest gene next to the binding site was used for analysis. 21 genes were found to be differentially expressed in both sets of microarray with binding sites for all three of the Sox proteins. These included targets like* Sox5* and* Sox6* themselves,* Tgfb2*,* Tle3*, and* Fbxl18* (Figure S3, Figure 5). To confirm the transcriptional activity of the binding sites found for the Sox trio near some of these targets, luciferase assay was carried out for the Sox trio-bound enhancers of* Fbxl18*,* Rad51c*,* Sox11*,* Sox5*,* Sox6*,* Tgfb2*, and* Tle3*. Luciferase reporters were constructed with the element bearing the binding sites for the three Sox proteins of each of the target genes upstream of a minimal promoter (Table S3). The results showed that the DNA elements bound by all three Sox proteins were activated in the presence of the Sox trio, validating the functionality of the identified binding sites (Figure S5).

The different Sox proteins were then further tested for their individual contribution in the transcriptional activation of the Sox trio-bound regulatory regions found near the genes* Fbxl18*,* Rad51c*,* Tgfb2*, and* Tle3 *(Figures 5(a) and 5(b)). These four genes were shortlisted as little has been reported about their role in chondrogenesis. It was observed from the luciferase assay that the absence of Sox9 alone caused the biggest drop in the reporter activity of all the Sox-bound enhancers whereas the loss of Sox5 and/or Sox6 resulted in a smaller loss of activity. The results indicated and supported the hypothesis that Sox9 is the main activator for the regulatory regions of chondrogenic genes with Sox5 and Sox6 supporting it.

**Figure 6:**
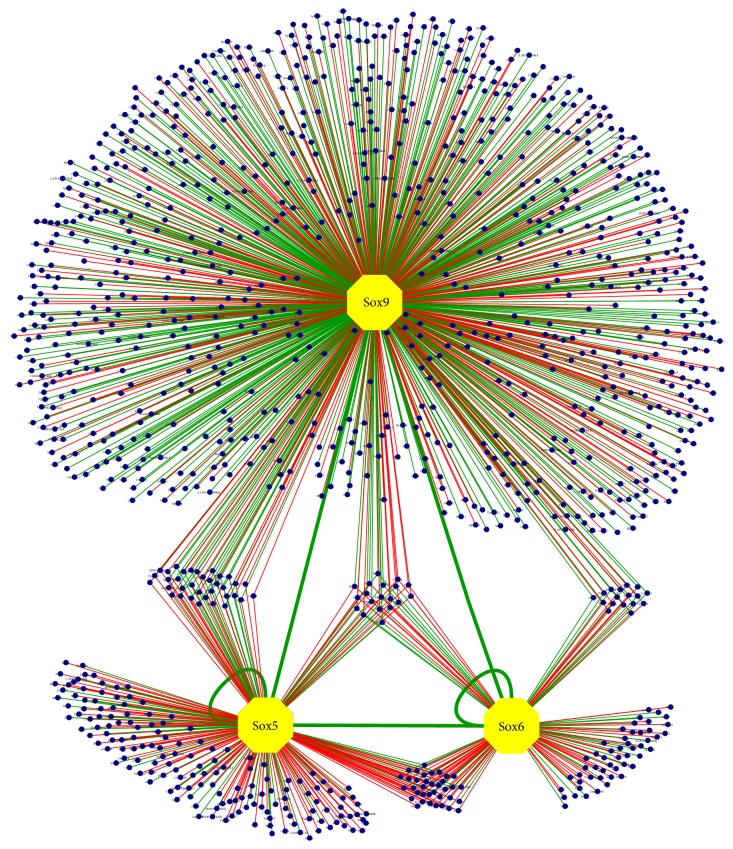
Sox trio-regulated network of genes. Blue dots represent genes activated (green lines) or repressed (red lines) by the transcription factors Sox9, Sox5, and Sox6 (see also Table S5).

To investigate the biological function of these Sox trio-regulated genes in particular with respect to skeletal formation, Morpholino-mediated knockdowns of* Fbxl18*,* Rad51c*,* Tgfb2*, and* Tle3* were performed in zebrafish by injecting the translation blocking Morpholino into the 1-cell stage of zebrafish embryos.* Tle3 *has two homologues in zebrafish,* groucho1 (gro1)* and* groucho2 (gro2)*. Since* gro1* is expressed primarily in the central nervous and hematopoietic system [75, 76], we chose to focus on* gro2.* Reduced expression of* Fbxl18*,* Tgfb2*, and* Tle3 (gro2)* resulted in a marked midline phenotype compared to the control that was injected with the scramble Morpholino at 24 hpf (Figure 5(c); Table S6). Transverse sections of the Morpholino-injected embryos showed extensive defects in the emerging sclerotome as demonstrated by the reduced Alcian Blue staining and loss of structural integrity, indicating that the three genes played a key role in developing chondrocytes (Figure 5(c)). Knockdown of* Rad51c* in the zebrafish was inconclusive, as the mutants do not survive after Morpholino injection. In summary, the in vivo results from the microarray and ChIP-Seq done in mice, together with the zebrafish knockdowns and in vitro luciferase assays performed, indicate that* Fbxl18*,* Tgfb2*, and* Tle3* are involved in sclerotome formation and are transcriptionally regulated by the Sox trio. This also provides validation to the in vivo datasets generated in this study, allowing the construction of the most comprehensive Sox trio GRN done to date (Figure 6).

## 4. Discussion

It is becoming increasingly clear that the complete understanding of any biological process in vertebrates will require a comprehensive approach looking at multiple transcription factors simultaneously and incorporating systems biology approaches [77–79]. Coupling of genomewide binding site data with expression profiling will give a detailed snapshot of the network of genes involved in a particular process [80, 81]. Previously only* Sox2* was subjected to a comprehensive genomewide systems approach [82] but this was limited to in vitro cell culture lines which often fail to recapitulate the actual in vivo function. Our study looked at three major transcription factors involved in organogenesis in the developing embryo to understand the molecular and genetic events underlying the process of chondrogenesis. Previously there had been numerous attempts to look at the role of the Sox trio in gene regulation. These studies often involved analyzing single gene loci in in vitro systems [29, 30, 83]. Our three-factor ChIP-Seq coupled with extensive expression profiling performed using developing mouse chondrocytes in an in vivo context has made it possible to perform a global analysis, deciphering the genomewide interactive roles of Sox9, Sox5, and Sox6 in regulating the genes involved in chondrogenesis thus providing an extensive Sox trio-driven gene regulatory network in vivo for the first time.

The approach of enriching for specific chondrogenic tissues in both ChIP-Seq and transcriptome profiling experiments has allowed us to specifically look at genes and pathways involved primarily in chondrogenesis. By analyzing the gene expression profiles together with the identified binding sites for the Sox trio, we identified a wide repertoire of genes that are regulated directly by Sox9 alone such as* Prelp*,* Hapln1*, and the novel target* 3110079O15Rik* as well as those that are regulated by Sox5 and/or Sox6 alone or together with Sox9 during embryogenesis (Figure 6).

To provide biological insight, hypothesis generating screens such as ChIP-Seq experiments need to be combined with meaningful assays for downstream functional validation. For this study, we took advantage of the zebrafish model as it allows high-throughput knockdown analysis in vivo in the context of the entire developing organism. By employing the multifactor ChIP-Seq and microarray, together with the zebrafish validation, three new genes* Fbxl18*,* Tgfb2*, and* Tle3* were discovered to be coregulated by the Sox trio, highlighting the biological relevance of the network generated. Other genes previously identified to be part of skeletogenesis but not known to be controlled by the Sox trio were also uncovered in our network. These include* Notch4*, part of the Notch-Delta signaling pathway, and* Sox11* whose homozygous mutants display craniofacial and skeletal abnormalities [84]. In addition, multiple Sox binding sites in and around* Sox5* and* Sox6* were found and validated, providing evidence of the autoregulatory role of the Sox trio in vivo. The network also includes genes that were previously not thought to play any role in chondrogenesis like* Fbxl18*,* Tle3*,* Rad51c*, and* Bach2 *and we have validated two of these novel targets in this study.

The knockdowns of* Fbxl18*,* Tgfb2*, and* Tle3* showed clear sclerotome defects thus providing evidence for their role in skeletal under the control of the Sox trio enhancers. A recent report linked the receptor of* Tgfb2 (Tgfbr2)* to intervertebral disk formation [85]. Our data clearly shows that the ligand* Tgfb2* is directly controlled by the Sox trio. The transcriptional activation assay with Sox trio-bound regulatory regions proves and supports the hypothesis that in the process of chondrogenesis Sox9 plays a primary and compensatory role with Sox5 and Sox6 complimenting it. This may explain why Sox5 and Sox6 single null mutants have a mild phenotype up to a very late stage in development as Sox9 can compensate to a large extent for their loss.

Sox9 was previously shown to bind to pairs of inverted* Sox* motifs from studies done on campomelic dysplasia patients and in cell culture [29, 66]. Our Sox9 ChIP-Seq data on mouse embryonic tissue confirm this observation in vivo and further detects the presence of Sox5 and Sox6 binding sites along with Sox9 near many genes, helping to uncover new genes controlled by the Sox trio with a potential role in chondrogenesis.

One of the defining features of these Sox-bound enhancers that we found is that they are at a fairly large distance from the TSS. There have been increasing numbers of publications from genomewide studies indicating that most of the transcription factor binding sites identified are found in the intragenic region rather than at the TSS [79, 86]. Similar binding profiles are observed for other transcription factors in mid gestation mouse embryos (unpublished data) leading us to believe that the primary role for these transcription factors at these stages is not just to initiate transcription of downstream genes but mainly to direct the tissue specificity by controlling a subset of their regulatory elements simultaneously in a fine-tuned manner in a way we are beginning to understand.

This study has provided an extensive network of genes that are directly and indirectly controlled by* Sox9*,* Sox5*, and* Sox6* in the context of cell type specification in mouse embryos. Evidence supporting the role that the Sox trio plays in this biological process has been previously reported. This however is the first systems biology genomewide in vivo analysis done and reported, validating some of the known players such as* Sox5* and* Sox6* while at the same time discovering and verifying novel ones like* Tle3*. The information generated from this extensive study will allow for future work to be conducted on some of the downstream genes identified, paving the way for a comprehensive understanding of the vertebrate chondrogenic gene regulatory network that will be insightful in the treatment of human skeletal diseases.

## Supplementary Material

Supplementary Figure 1: Sox9+/+ (EGFP) was made with the EGFP reporter linked via F2A, giving rise to a bicistronic system with a single open reading frame shown in Figure 1B. Western blot in upper panel of Figure S1 shows that the Sox9 protein and the F2A protein have been efficiently cleaved from each other, giving rise to a functional Sox9 protein. Transgenic embryos expressing EGFP were used for FACS and lower panel of Figure S1 shows how the gating was set using wildtype mice and the efficiency of the sort.
Supplementary Figure 2: Differentially expressed genes from the Sox9 and Sox5/6 microarray were verified in Figure S2 before using them for GO analysis. The GO-terms for the up-regulated genes when the Sox trio is inactive are also displayed relative to the down-regulated genes in Figure 2.
Supplementary Figure 3: Figure 3 focuses on the Sox9, Sox5, and Sox6 ChIP-seq performed. Top panel of Figure S3 shows the ChIP-qPCR validation of the ChIP-seq. Centre panel of Figure S3 shows the distribution of the number of bp between the dimerization binding sites of Sox9 as reflected in Figure 3C. Bottom panel of Figure S3 shows the binding site profile for Sox5 and Sox6 by the Sox trio themselves.
Supplementary Figure 4: Figure S4 shows the validation of the Sox9 binding sites using the luciferase assay before the GO-analysis was applied as reflected in Figure 4.
Supplementary Figure 5: Figure S5 shows the luciferase assay of the transcriptional activity of the Sox9, Sox5 and Sox6 binding sites in genes, Fbxl18, Rad51c, Sox11, Sox5, Sox6, Tgfb2, and Tle3. The sites for Fbxl18, Rad51c, Tgfb2, and Tle3 were further tested for the individual Sox protein contributions in Figure 5c.
Supplementary Table 1: Differentially expressed genes in the Sox9 and Sox5/6 microarray generated from the transgenic mice made shown in Figure 1 and used for further analysis in Figure 2 and to overlap with the ChIP-seq data.
Supplementary Table 2: List of differentially expressed genes analyzed by RNA SISH from the Sox9 microarray on E13.5 mouse embryos.
Supplementary Table 3: Primers used for qPCR for Figure S3 and primers used for the amplifying the luciferase sequences of all the luciferase assays done in Figure S4 and Figure S5.
Supplementary Table 4: List of genes that are differentially expressed from the microarray were overlapped with the Sox trio binding sites from the ChIP-seq analysis. These were put through the GO analysis that is shown in Figure 4.
Supplementary Table 5: Differentially expressed genes on both sides of the Sox9, Sox5, and Sox6 binding sits used to construct the GRN on Figure 6.
Supplementary Table 6: Statistics for zebrafish morphants and morpholino sequences that were used in Figure 5.

## References

[1] Lefebvre V. (2010). The SoxD transcription factors—Sox5, Sox6, and Sox13—are key cell fate modulators. *International Journal of Biochemistry and Cell Biology*.

[2] Sarkar A., Hochedlinger K. (2013). The SOX family of transcription factors: versatile regulators of stem and progenitor cell fate. *Cell Stem Cell*.

[3] Kamachi Y., Kondoh H. (2013). Sox proteins: regulators of cell fate specification and differentiation. *Development*.

[4] Castillo S. D., Sanchez-Cespedes M. (2012). The SOX family of genes in cancer development: Biological relevance and opportunities for therapy. *Expert Opinion on Therapeutic Targets*.

[5] Chew L.-J., Gallo V. (2009). The Yin and Yang of Sox proteins: activation and repression in development and disease. *Journal of Neuroscience Research*.

[6] Harley V., Lefebvre V. (2010). Twenty Sox, twenty years. *International Journal of Biochemistry and Cell Biology*.

[7] Ohba S., He X., Hojo H., McMahon A. P. (2015). Distinct transcriptional programs underlie sox9 regulation of the mammalian chondrocyte. *Cell Reports*.

[8] Kadaja M., Keyes B. E., Lin M. (2014). SOX9: a stem cell transcriptional regulator of secreted niche signaling factors. *Genes and Development*.

[9] Akiyama H. (2008). Control of chondrogenesis by the transcription factor Sox9. *Modern Rheumatology*.

[10] Kiefer J. C. (2007). Back to basics: Sox genes. *Developmental Dynamics*.

[11] Henry S. P., Liang S., Akdemir K. C., De Crombrugghe B. (2012). The postnatal role of Sox9 in cartilage. *Journal of Bone and Mineral Research*.

[12] Dy P., Wang W., Bhattaram P. (2012). Sox9 directs hypertrophic maturation and blocks osteoblast differentiation of growth plate chondrocytes. *Developmental Cell*.

[13] Leung V. Y. L., Gao B., Leung K. K. H. (2011). SOX9 governs differentiation stage-specific gene expression in growth plate chondrocytes via direct concomitant transactivation and repression. *PLoS Genetics*.

[14] Wang Z. H., Li X. L., He X. J. (2014). Delivery of the Sox9 gene promotes chondrogenic differentiation of human umbilical cord blood-derived mesenchymal stem cells in an in vitro model. *Brazilian Journal of Medical and Biological Research*.

[15] Sun W., Zhang K., Liu G. (2014). Sox9 gene transfer enhanced regenerative effect of bone marrow mesenchymal stem cells on the degenerated intervertebral disc in a rabbit model. *PLoS ONE*.

[16] Oh C.-D., Lu Y., Liang S. (2014). SOX9 regulates multiple genes in chondrocytes, including genes encoding ECM proteins, ECM modification enzymes, receptors, and transporters. *PLoS ONE*.

[17] Ahmed N., Iu J., Brown C. E., Taylor D. W., Kandel R. A. (2014). Serum- and growth-factor-free three-dimensional culture system supports cartilage tissue formation by promoting collagen synthesis via Sox9-Col2a1 interaction. *Tissue Engineering - Part A*.

[18] Lefebvre V., Bhattaram P. (2010). Vertebrate skeletogenesis. *Current Topics in Developmental Biology*.

[19] Superti-Furga A., Unger S., Beighton P. (2007). Nosology and classification of genetic skeletal disorders: 2006 Revision. *American Journal of Medical Genetics, Part A*.

[20] Zelzer E., Olsen B. R. (2003). The genetic basis for skeletal diseases. *Nature*.

[21] Bi W., Deng J. M., Zhang Z., Behringer R. R., de Crombrugghe B. (1999). Sox9 is required for cartilage formation. *Nature Genetics*.

[22] Foster J. W., Dominguez-Steglich M. A., Guioli S. (1994). Campomelic dysplasia and autosomal sex reversal caused by mutations in an SRY-related gene. *Nature*.

[23] Wagner T., Wirth J., Meyer J. (1994). Autosomal sex reversal and campomelic dysplasia are caused by mutations in and around the *SRY*-related gene *SOX9*. *Cell*.

[24] Akiyama H., Lefebvre V. (2011). Unraveling the transcriptional regulatory machinery in chondrogenesis. *Journal of Bone and Mineral Metabolism*.

[25] Ikeda T., Kawaguchi H., Kamekura S. (2005). Distinct roles of Sox5, Sox6, and Sox9 in different stages of chondrogenic differentiation. *Journal of Bone and Mineral Metabolism*.

[26] Ikeda T., Kamekura S., Mabuchi A. (2004). The combination of SOX5, SOX6, and SOX9 (the SOX trio) provides signals sufficient for induction of permanent cartilage. *Arthritis and Rheumatism*.

[27] Akiyama H., Chaboissier M.-C., Martin J. F., Schedl A., de Crombrugghe B. (2002). The transcription factor *Sox9* has essential roles in successive steps of the chondrocyte differentiation pathway and is required for expression of *Sox5* and *Sox6*. *Genes and Development*.

[28] Smits P., Li P., Mandel J. (2001). The transcription factors L-Sox5 and Sox6 are essential for cartilage formation. *Developmental Cell*.

[29] Han Y., Lefebvre V. (2008). L-Sox5 and Sox6 drive expression of the aggrecan gene in cartilage by securing binding of Sox9 to a far-upstream enhancer. *Molecular and Cellular Biology*.

[30] Nagy A., Kénesi E., Rentsendorj O. (2011). Evolutionarily conserved, growth plate zone-specific regulation of the matrilin-1 promoter: L-Sox5/Sox6 and Nfi factors bound near TATA finely tune activation by Sox9. *Molecular and Cellular Biology*.

[31] Lufkin T. (1996). Transcriptional control of Hox genes in the vertebrate nervous system. *Current Opinion in Genetics and Development*.

[32] Bi W., Huang W., Whitworth D. J. (2001). Haploinsufficiency of Sox9 results in defective cartilage primordia and premature skeletal mineralization. *Proceedings of the National Academy of Sciences of the United States of America*.

[33] Smits P., Dy P., Mitra S., Lefebvre V. (2004). Sox5 and Sox6 are needed to develop and maintain source, columnar, and hypertrophic chondrocytes in the cartilage growth plate. *Journal of Cell Biology*.

[34] Nakamura Y., Yamamoto K., He X. (2011). Wwp2 is essential for palatogenesis mediated by the interaction between Sox9 and mediator subunit 25. *Nature Communications*.

[35] Chen X., Li X., Wang W., Lufkin T. (1996). Dlx5 and Dlx6: An evolutionary conserved pair of murine homeobox genes expressed in the embryonic skeleton. *Annals of the New York Academy of Sciences*.

[36] Kraus P., Lufkin T. (1999). Mammalian Dlx homeobox gene control of craniofacial and inner ear morphogenesis. *Journal of Cellular Biochemistry*.

[37] Chatterjee S., Bourque G., Lufkin T. (2011). Conserved and non-conserved enhancers direct tissue specific transcription in ancient germ layer specific developmental control genes. *BMC Developmental Biology*.

[38] Chatterjee S., Min L., Karuturi R. K. M., Lufkin T. (2010). The role of post-transcriptional RNA processing and plasmid vector sequences on transient transgene expression in zebrafish. *Transgenic Research*.

[39] Kraus P., Leong G., Tan V. (2010). A more cost effective and rapid high percentage germ-line transmitting chimeric mouse generation procedure via microinjection of 2-cell, 4-cell, and 8-cell embryos with ES and iPS cells. *Genesis*.

[40] Lee W. J., Kraus P., Lufkin T. (2012). Endogenous tagging of the murine transcription factor Sox5 with hemaglutinin for functional studies. *Transgenic Research*.

[41] Lewandoski M., Wassarman K. M., Martin G. R. (1997). Zp3-cre, a transgenic mouse line for the activation or inactivation of IoxP-flanked target genes specifically in the female germ line. *Current Biology*.

[42] Wang Z.-X., Kueh J. L. L., Teh C. H.-L. (2007). Zfp206 is a transcription factor that controls pluripotency of embryonic stem cells. *Stem Cells*.

[43] Kraus P., Lufkin T. (2006). Dlx homeobox gene control of mammalian limb and craniofacial development. *American Journal of Medical Genetics, Part A*.

[44] Wang W., Lo P., Frasch M., Lufkin T. (2000). Hmx: an evolutionary conserved homeobox gene family expressed in the developing nervous system in mice and Drosophila. *Mechanisms of Development*.

[45] Chan H. Y., Sivakamasundari V., Xing X. (2011). Comparison of IRES and F2A-based locus-specific multicistronic expression in stable mouse lines. *PLoS ONE*.

[46] Tribioli C., Lufkin T. (1999). The murine Bapx1 homeobox gene plays a critical role in embryonic development of the axial skeleton and spleen. *Development*.

[47] Zhao F., Lufkin T., Gelb B. D. (2003). Expression of Tfap2d, the gene encoding the transcription factor Ap-2*δ* during mouse embryogenesis. *Gene Expression Patterns*.

[48] Tribioli C., Robledo R. F., Lufkin T. (2002). The murine fork head gene Foxn2 is expressed in craniofacial, limb, CNS and somitic tissues during embryogenesis. *Mechanisms of Development*.

[49] Sheng D., Qu D., Kwok K. H. H. (2010). Deletion of the WD40 domain of LRRK2 in zebrafish causes parkinsonism-like loss of neurons and locomotive defect. *PLoS Genetics*.

[50] Yap S. P., Xing X., Kraus P., Sivakamasundari V., Chan H. Y., Lufkin T. (2011). Generation of mice with a novel conditional null allele of the Sox9 gene. *Biotechnology Letters*.

[51] Schmidt D., Wilson M. D., Spyrou C., Brown G. D., Hadfield J., Odom D. T. (2009). ChIP-seq: using high-throughput sequencing to discover protein-DNA interactions. *Methods*.

[52] Zhang Y., Liu T., Meyer C. A. (2008). Model-based analysis of ChIP-Seq (MACS). *Genome Biology*.

[53] Kimmel C. B., Ballard W. W., Kimmel S. R., Ullmann B., Schilling T. F. (1995). Stages of embryonic development of the zebrafish. *Developmental Dynamics*.

[54] Robu M. E., Larson J. D., Nasevicius A. (2007). p53 activation by knockdown technologies. *PLoS Genetics*.

[55] da Huang W., Sherman B. T., Lempicki R. A. (2009). Systematic and integrative analysis of large gene lists using DAVID bioinformatics resources. *Nature Protocols*.

[56] Smoot M. E., Ono K., Ruscheinski J., Wang P. L., Ideker T. (2011). Cytoscape 2.8: new features for data integration and network visualization. *Bioinformatics*.

[57] Trichas G., Begbie J., Srinivas S. (2008). Use of the viral 2A peptide for bicistronic expression in transgenic mice. *BMC Biology*.

[58] Lefebvre V., Li P., De Crombrugghe B. (1998). A new long form of Sox5 (L-Sox5), Sox6 and Sox9 are coexpressed in chondrogenesis and cooperatively activate the type II collagen gene. *The EMBO Journal*.

[59] Stolt C. C., Schlierf A., Lommes P. (2006). SoxD proteins influence multiple stages of oligodendrocyte development and modulate SoxE protein function. *Developmental Cell*.

[60] Connor F., Wright E., Denny P., Koopman P., Ashworth A. (1995). The Sry-related HMG box-containing gene Sox6 is expressed in the adult testis and developing nervous system of the mouse. *Nucleic Acids Research*.

[61] Dennis G., Sherman B. T., Hosack D. A. (2003). DAVID: database for annotation, visualization, and integrated discovery. *Genome Biol*.

[62] Zhang P., Jimenez S. A., Stokes D. G. (2003). Regulation of human COL9A1 gene expression: activation of the proximal promoter region by SOX9. *Journal of Biological Chemistry*.

[63] Genzer M. A., Bridgewater L. C. (2007). A Col9a1 enhancer element activated by two interdependent SOX9 dimers. *Nucleic Acids Research*.

[64] Bailey T. L., Boden M., Buske F. A. (2009). MEME SUITE: tools for motif discovery and searching. *Nucleic Acids Research*.

[65] Bridgewater L. C., Walker M. D., Miller G. C. (2003). Adjacent DNA sequences modulate Sox9 transcriptional activation at paired Sox sites in three chondrocyte-specific enhancer elements. *Nucleic Acids Research*.

[66] Bernard P., Tang P., Liu S., Dewing P., Harley V. R., Vilain E. (2003). Dimerization of SOX9 is required for chondrogenesis, but not for sex determination. *Human Molecular Genetics*.

[67] Liu C.-F., Lefebvre V. (2015). The transcription factors SOX9 and SOX5/SOX6 cooperate genome-wide through super-enhancers to drive chondrogenesis. *Nucleic Acids Research*.

[68] Garside V. C., Cullum R., Alder O. (2015). SOX9 modulates the expression of key transcription factors required for heart valve development. *Development (Cambridge)*.

[69] Liu C., Samsa W. E., Zhou G., Lefebvre V. (2017). Transcriptional control of chondrocyte specification and differentiation. *Seminars in Cell & Developmental Biology*.

[70] Hojo H., McMahon A. P., Ohba S. (2016). An emerging regulatory landscape for skeletal development. *Trends in Genetics*.

[71] Akiyama H., Stadler H. S., Martin J. F. (2007). Misexpression of Sox9 in mouse limb bud mesenchyme induces polydactyly and rescues hypodactyly mice. *Matrix Biology*.

[72] Oh C.-D., Maity S. N., Lu J.-F. (2010). Identification of SOX9 interaction sites in the genome of chondrocytes. *PLoS ONE*.

[73] Harley V. R., Lovell-badge R., Goodfellow P. N. (1994). Definition of a consensus DNA binding site for SRY. *Nucleic Acids Research*.

[74] Lefebvre V., Huang W., Harley V. R., Goodfellow P. N., De Crombrugghe B. (1997). SOX9 is a potent activator of the chondrocyte-specific enhancer of the pro*α*1(II) collagen gene. *Molecular and Cellular Biology*.

[75] Scholpp S., Delogu A., Gilthorpe J., Peukert D., Schindler S., Lumsden A. (2009). Her6 regulates the neurogenetic gradient and neuronal identity in the thalamus. *Proceedings of the National Academy of Sciences of the United States of America*.

[76] Dayyani F., Wang J., Yeh J.-R. J. (2008). Loss of TLE1 and TLE4 from the del(9q) commonly deleted region in AML cooperates with AML1-ETO to affect myeloid cell proliferation and survival. *Blood*.

[77] He A., Kong S. W., Ma Q., Pu W. T. (2011). Co-occupancy by multiple cardiac transcription factors identifies transcriptional enhancers active in heart. *Proceedings of the National Academy of Sciences of the United States of America*.

[78] Soler E., Andrieu-Soler C., De Boer E. (2010). The genome-wide dynamics of the binding of Ldb1 complexes during erythroid differentiation. *Genes and Development*.

[79] Tijssen M. R., Cvejic A., Joshi A. (2011). Genome-wide analysis of simultaneous GATA1/2, RUNX1, FLI1, and SCL binding in megakaryocytes identifies hematopoietic regulators. *Developmental Cell*.

[80] Fang X., Yoon J.-G., Li L. (2011). The SOX2 response program in glioblastoma multiforme: an integrated ChIP-seq, expression microarray, and microRNA analysis. *BMC Genomics*.

[81] Tamplin O. J., Cox B. J., Rossant J. (2011). Integrated microarray and ChIP analysis identifies multiple Foxa2 dependent target genes in the notochord. *Developmental Biology*.

[82] Engelen E., Akinci U., Bryne J. C. (2011). Sox2 cooperates with Chd7 to regulate genes that are mutated in human syndromes. *Nature Genetics*.

[83] Liu C.-J., Zhang Y., Xu K., Parsons D., Alfonso D., Di Cesare P. E. (2007). Transcriptional activation of cartilage oligomeric matrix protein by Sox9, Sox5, and Sox6 transcription factors and CBP/p300 coactivators. *Frontiers in Bioscience*.

[84] Sock E., Rettig S. D., Enderich J., Bösl M. R., Tamm E. R., Wegner M. (2004). Gene targeting reveals a widespread role for the high-mobility-group transcription factor Sox11 in tissue remodeling. *Molecular and Cellular Biology*.

[85] Sohn P., Cox M., Chen D., Serra R. (2010). Molecular profiling of the developing mouse axial skeleton: a role for Tgfbr2 in the development of the intervertebral disc. *BMC Developmental Biology*.

[86] Holmstrom S. R., Deering T., Swift G. H. (2011). LRH-1 and PTF1-L coregulate an exocrine pancreas-specific transcriptional network for digestive function. *Genes and Development*.

