# Supplementary material for: An Integrative Developmental Genomics and Systems Biology Approach to Identify an In Vivo Sox Trio-Mediated Gene Regulatory Network in Murine Embryos
Source: Biomed Res Int. 2017 May 28;2017:8932583. doi: 10.1155/2017/8932583 (PMC5467288; doi:10.1155/2017/8932583)
Supplement: Supplementary file 1 — Supplementary Figure 1: Sox9+/+ (EGFP) was made with the EGFP reporter linked via F2A, giving rise to a bicistronic system with a single open reading frame shown in Figure 1B. Western blot in upper panel of Figure S1 shows that the Sox9 protein and the F2A protein have been efficiently cleaved from each other, giving rise to a functional Sox9 protein. Transgenic embryos expressing EGFP were used for FACS and lower panel of Figure S1 shows how the gating was set using wildtype mice and the efficiency of the sort. Supplementary Figure 2: Differentially expressed genes from the Sox9 and Sox5/6 microarray were verified in Figure S2 before using them for GO analysis. The GO-terms for the up-regulated genes when the Sox trio is inactive are also displayed relative to the down-regulated genes in Figure 2. Supplementary Figure 3: Figure 3 focuses on the Sox9, Sox5, and Sox6 ChIP-seq performed. Top panel of Figure S3 shows the ChIP-qPCR validation of the ChIP-seq. Centre panel of Figure S3 shows the distribution of the number of bp between the dimerization binding sites of Sox9 as reflected in Figure 3C. Bottom panel of Figure S3 shows the binding site profile for Sox5 and Sox6 by the Sox trio themselves. Supplementary Figure 4: Figure S4 shows the validation of the Sox9 binding sites using the luciferase assay before the GO-analysis was applied as reflected in Figure 4. Supplementary Figure 5: Figure S5 shows the luciferase assay of the transcriptional activity of the Sox9, Sox5 and Sox6 binding sites in genes, Fbxl18, Rad51c, Sox11, Sox5, Sox6, Tgfb2, and Tle3. The sites for Fbxl18, Rad51c, Tgfb2, and Tle3 were further tested for the individual Sox protein contributions in Figure 5c. Supplementary Table 1: Differentially expressed genes in the Sox9 and Sox5/6 microarray generated from the transgenic mice made shown in Figure 1 and used for further analysis in Figure 2 and to overlap with the ChIP-seq data. Supplementary Table 2: List of differentially expressed genes analy [file 8932583.f1.pdf]

Figure S1

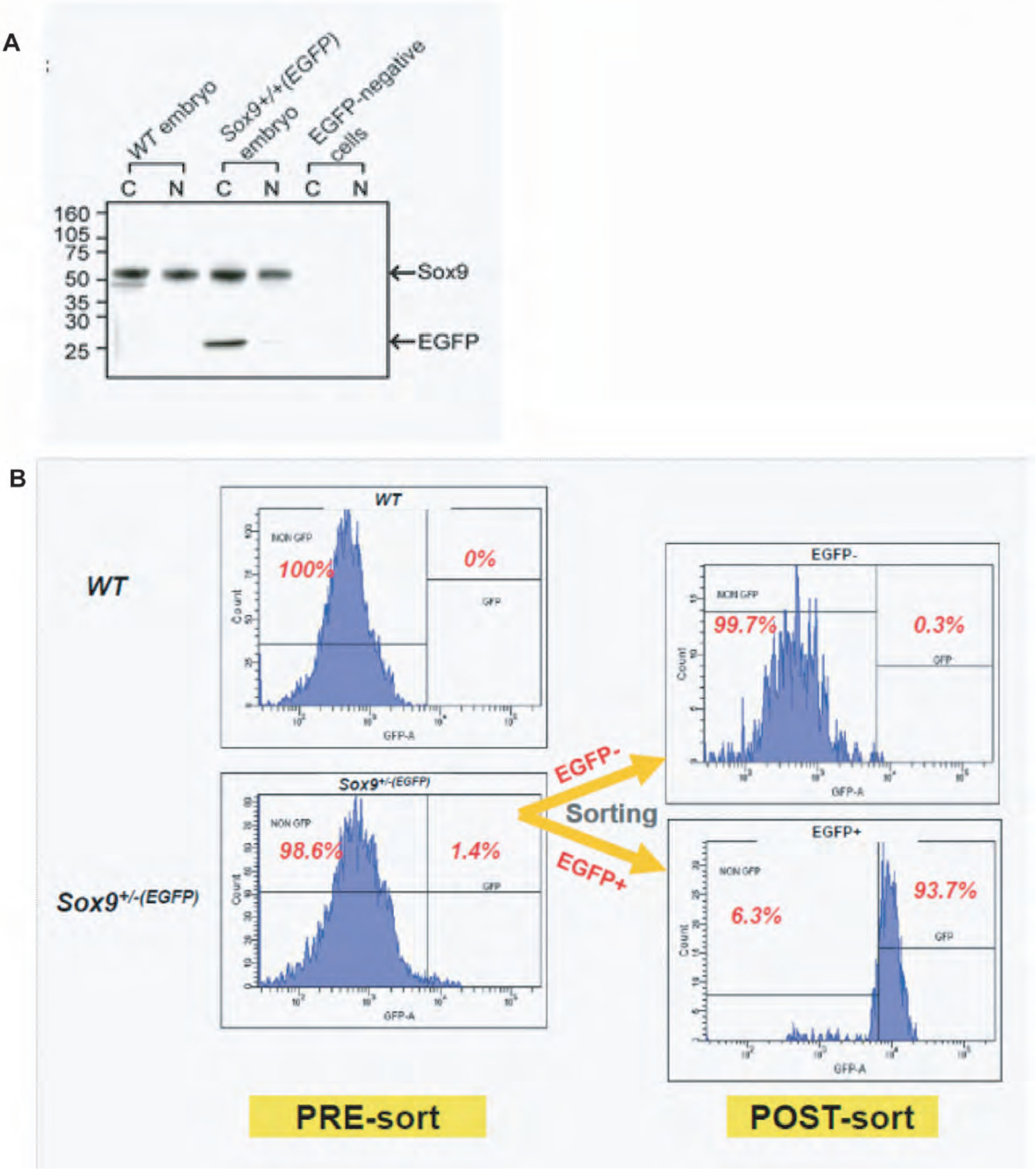
A
WT embryo
Sox9+/+(EGFP) embryo
EGFP-negative cells
C
N
C
N
C
N
160
105
75
50
35
30
25
Sox9
EGFP
B
WT
Sox9+/-(EGFP)
NON GFP
GFP
100%
0%
98.6%
1.4%
Count
GFP-A
EGFP-
EGFP+
Sorting
99.7%
0.3%
6.3%
93.7%
PRE-sort
POST-sort

Figure S2

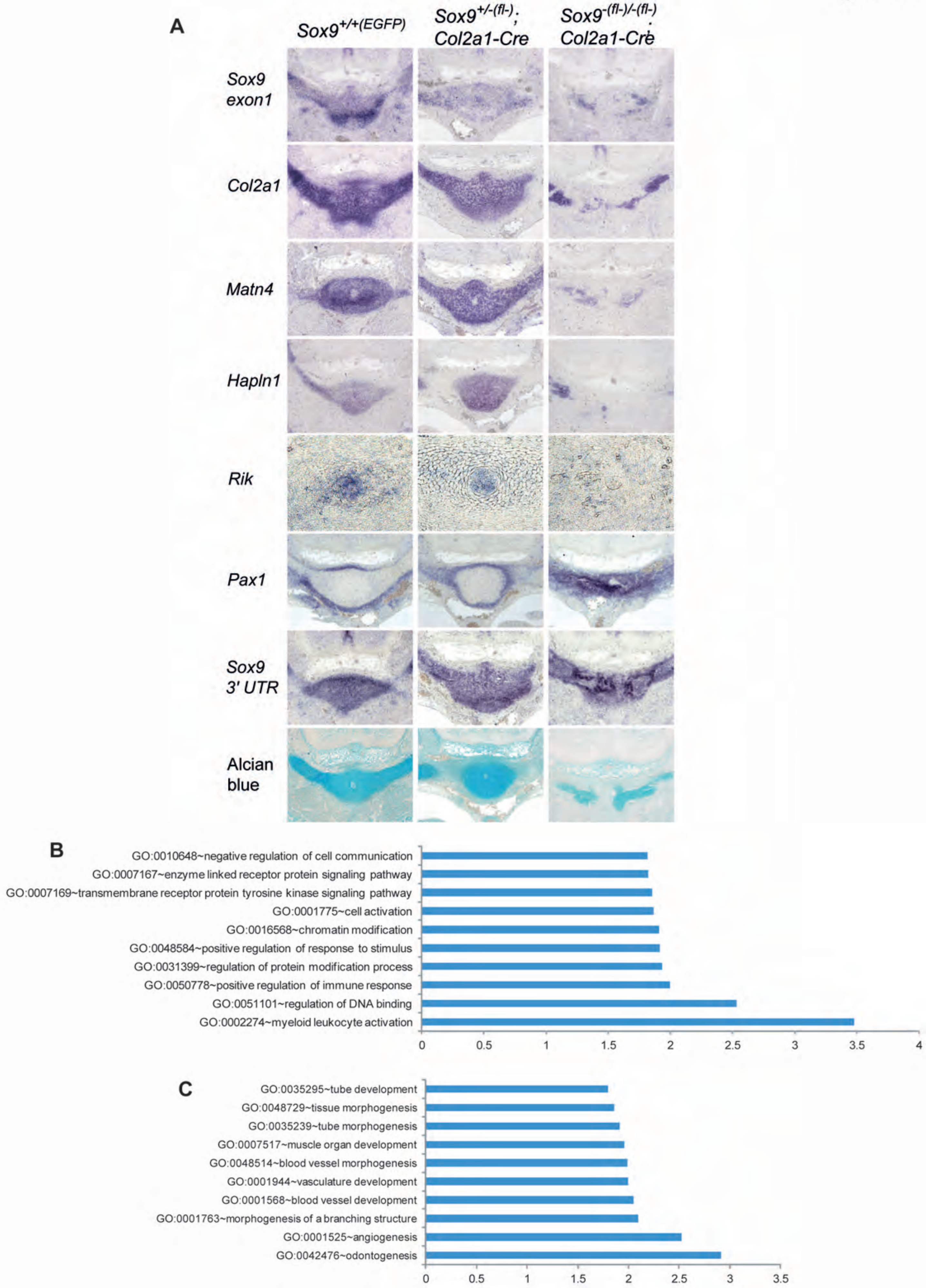

A
Sox9+/+(EGFP)
Sox9+/-(fl-); Col2a1-Cre
Sox9-(fl-)/-(fl-); Col2a1-Cre
Sox9 exon1
Col2a1
Matn4
Hapln1
Rik
Pax1
Sox9 3' UTR
Alcian blue
B
GO:0010648~negative regulation of cell communication
GO:0007167~enzyme linked receptor protein signaling pathway
GO:0007169~transmembrane receptor protein tyrosine kinase signaling pathway
GO:0001775~cell activation
GO:0016568~chromatin modification
GO:0048584~positive regulation of response to stimulus
GO:0031399~regulation of protein modification process
GO:0050778~positive regulation of immune response
GO:0051101~regulation of DNA binding
GO:0002274~myeloid leukocyte activation
0 0.5 1 1.5 2 2.5 3 3.5 4
C
GO:0035295~tube development
GO:0048729~tissue morphogenesis
GO:0035239~tube morphogenesis
GO:0007517~muscle organ development
GO:0048514~blood vessel morphogenesis
GO:0001944~vasculature development
GO:0001568~blood vessel development
GO:0001763~morphogenesis of a branching structure
GO:0001525~angiogenesis
GO:0042476~odontogenesis
0 0.5 1 1.5 2 2.5 3 3.5

Figure S3

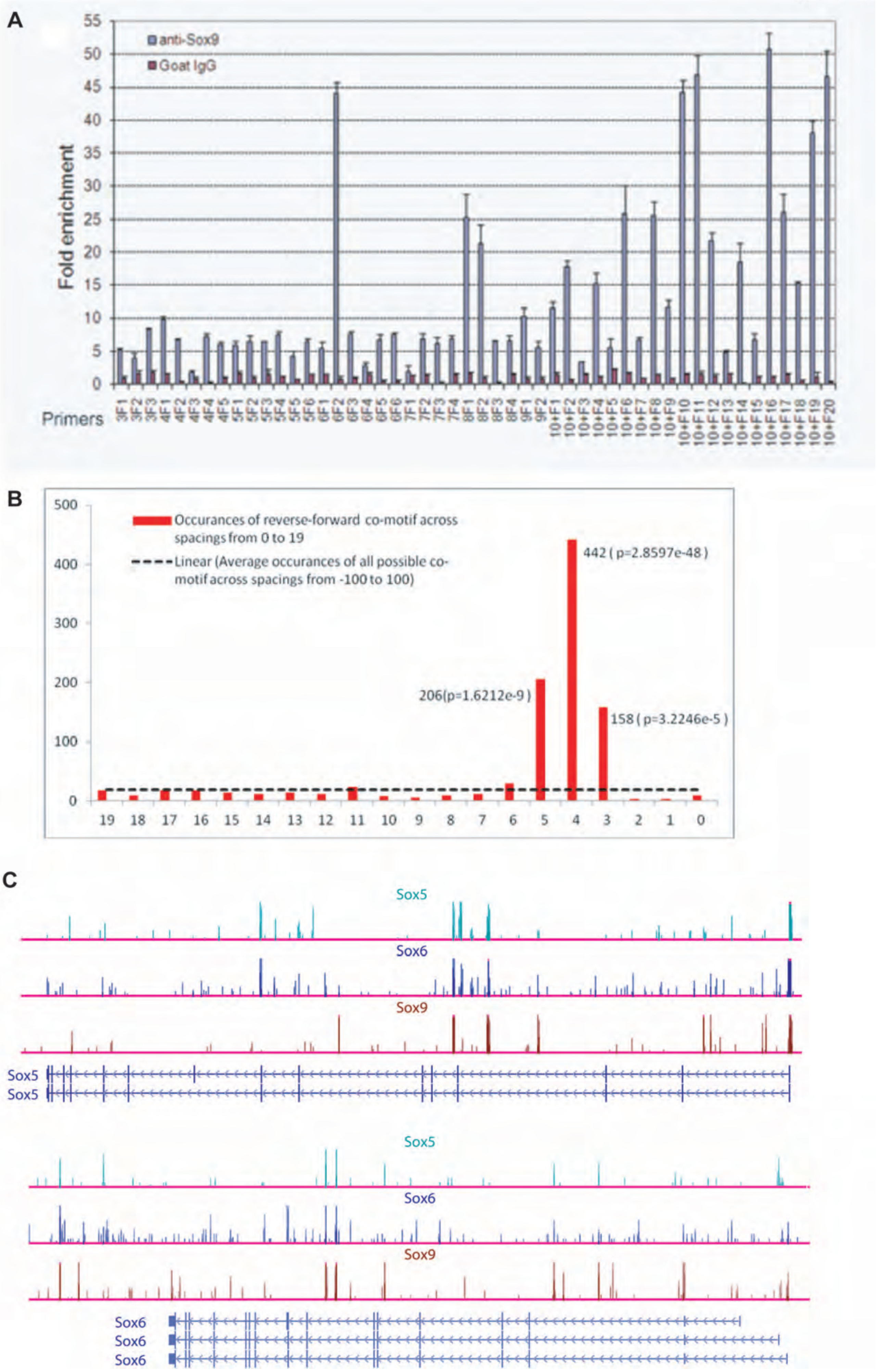
A
anti-Sox9
Goat IgG
Fold enrichment
Primers
3F1 3F2 3F3 4F1 4F2 4F3 4F4 4F5 5F1 5F2 5F3 5F4 5F5 5F6 6F1 6F2 6F3 6F4 6F5 6F6 7F1 7F2 7F3 7F4 8F1 8F2 8F3 8F4 9F1 9F2 10+F1 10+F2 10+F3 10+F4 10+F5 10+F6 10+F7 10+F8 10+F9 10+F10 10+F11 10+F12 10+F13 10+F14 10+F15 10+F16 10+F17 10+F18 10+F19 10+F20
B
Occurances of reverse-forward co-motif across spacings from 0 to 19
Linear (Average occurances of all possible co-motif across spacings from -100 to 100)
442 (p=2.8597e-48)
206(p=1.6212e-9)
158 (p=3.2246e-5)
C
Sox5
Sox6
Sox9
Sox5
Sox5
Sox5
Sox6
Sox9
Sox6
Sox6
Sox6

Figure S4

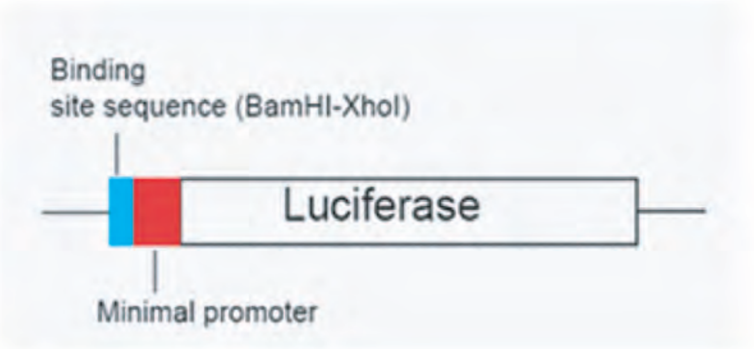
Binding
site sequence (BamHI-XhoI)
Luciferase
Minimal promoter

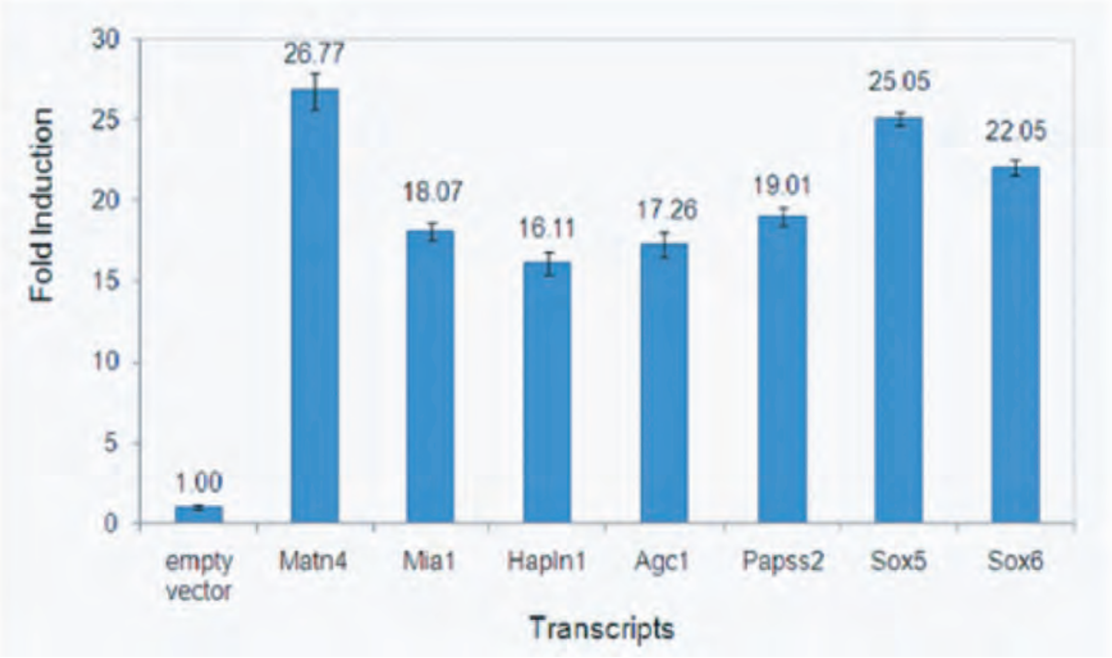
Fold Induction
Transcripts
30
25
20
15
10
5
0
1.00
26.77
18.07
16.11
17.26
19.01
25.05
22.05
empty vector
Matn4
Mia1
Hapln1
Agc1
Papss2
Sox5
Sox6

Figure S5

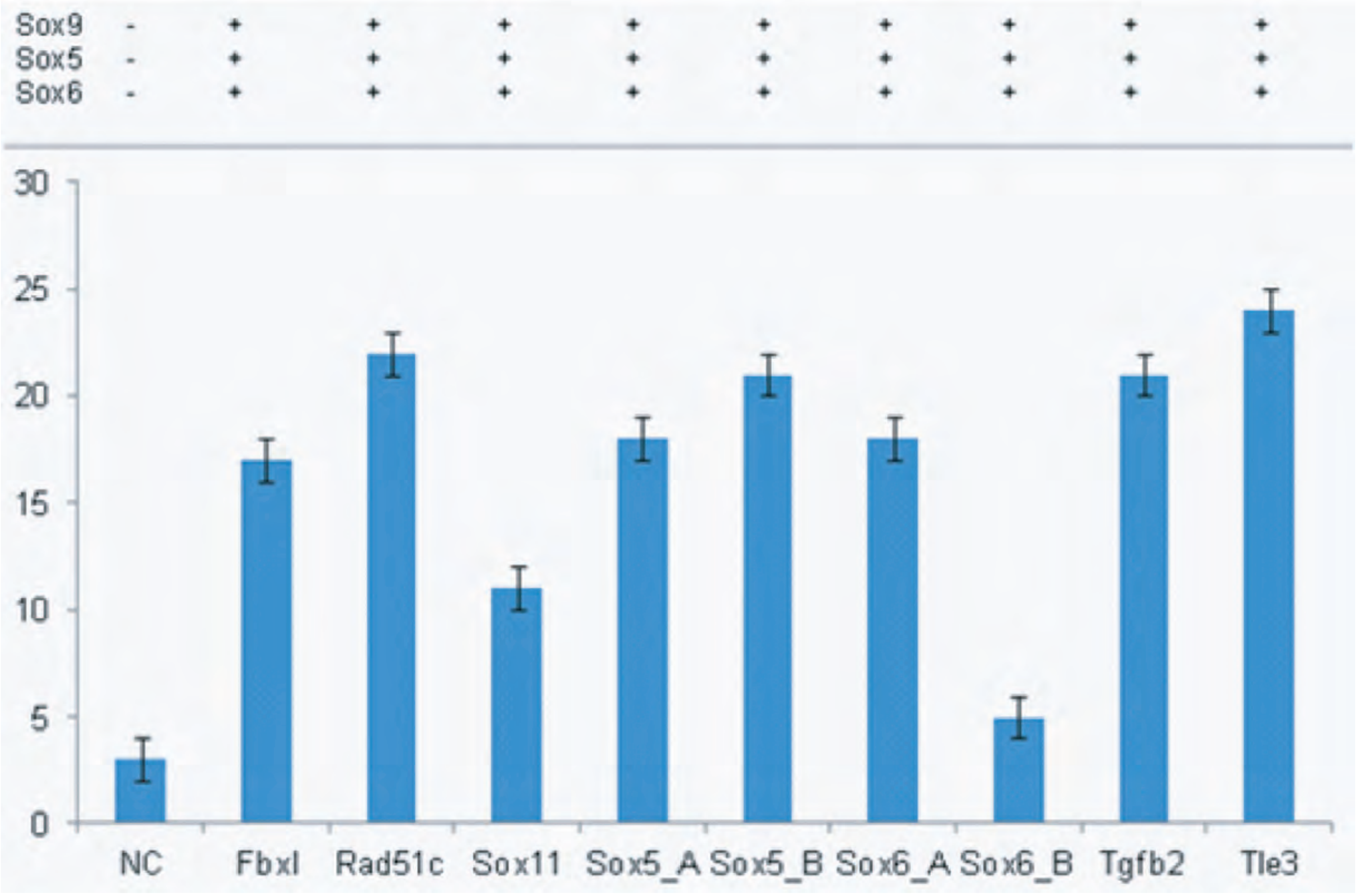

Sox9 - + + + + + + + + +
Sox5 - + + + + + + + + +
Sox6 - + + + + + + + + +
30
25
20
15
10
5
0
NC
Fbxl
Rad51c
Sox11
Sox5_A
Sox5_B
Sox6_A
Sox6_B
Tgfb2
Tle3

Table S1 Sox5 Sox6 (113 Pages)

Symbol	Corrected p-value	FCAbsolute	Regulation
LOC100045241	0.000126	1.979569435	down
Whsc1	0.000126	3.392271519	down
Tssc8	0.00014	5.709008217	down
Hist1h2ah	0.000268	4.491474628	down
Masp2	0.000437	3.014621258	up
N4bp2	0.000437	2.461571693	down
Clcn3	0.000471	2.399149418	down
Csnk2a1-rs3	0.000471	5.857130528	down
Cort	0.000535	2.484235287	up
Rcor1	0.000677	3.296764374	down
Smchd1	0.000743	3.363729715	down
D030068E18Rik	0.000835	4.346210957	down
Sema5a	0.000835	2.67321682	up
A130096D14Rik	0.000835	2.049284935	down
Chic1	0.000835	4.537966251	down
Sfrs11	0.000835	3.639235735	down
Sec22c	0.000835	1.736928225	down
Tm7sf3	0.000835	10.5032444	down
Cenpf	0.000835	6.796654701	down
Srrm1	0.000856	1.515657187	down
Sox5	0.000856	6.362275124	down
Tmf1	0.000905	1.663706541	down
2610305D13Rik	0.000905	2.057766914	down
Tcfap2b	0.000905	5.2552104	down
2810408P10Rik	0.000905	3.362867355	down
Bclaf1	0.000905	1.719573617	down
Gpm6b	0.000905	2.81131506	down
Adam12	0.000905	2.027531862	up
2700063P19Rik	0.000905	4.18237114	down
4933432B13Rik	0.000905	2.330044508	down
B930006L02Rik	0.000905	2.314308405	down
Ddx6	0.000905	3.059930563	down
1110067B18Rik	0.000905	1.937761426	down
Egr3	0.000905	2.336658955	up
C530036F05Rik	0.000905	2.751230955	down
Lyrm2	0.000905	2.123450041	down
Pccb	0.000905	1.506727815	up
Anxa4	0.000905	1.867211223	up
1110059G02Rik	0.000905	3.253538132	up
Cdh10	0.000905	1.741377115	up
A730094H17Rik	0.000905	1.949303627	down
Npal2	0.000905	1.52602005	up
Il16	0.000905	1.760058761	down
Sema5a	0.000905	2.359884501	up
Nrip1	0.000905	2.405695438	down
Cc1	0.000905	5.117405415	down
Hist1h2ad	0.000905	4.205818176	down

EG383436	0.000905	2.115112543	up
A730021C13Rik	0.000957	2.032049656	down
Pcdh17	0.000957	1.946148992	down
LOC100047419	0.000984	3.524956942	down
Aoc3	0.000999	2.760412693	up
Cxxc6	0.000999	3.383396864	down
Akap9	0.000999	3.478304148	down
Tbl1x	0.000999	4.250277042	down
Cntn2	0.001019442	7.897854805	down
5830407P18Rik	0.001019442	4.236233234	down
LOC100045266	0.001019442	2.012479544	down
OTTMUSG00000010673	0.001084496	3.460067511	up
Nhlrc2	0.001133001	2.536384344	down
Fam107a	0.001133001	2.099262953	up
Atp7a	0.001188304	1.979646802	down
C330023M02Rik	0.001207266	2.53552413	down
A730036E13Rik	0.001207266	3.14649272	down
B130047N10Rik	0.001236391	1.65657866	down
Nrxn3	0.001236391	2.162994862	down
Dct	0.001252045	7.746574402	up
Mgea5	0.001252045	1.750078559	down
Abca8b	0.001252045	1.598145366	up
Ddx6	0.001252045	5.072891235	down
Mlp	0.001252045	2.12168932	down
Tcf4	0.001252045	1.630245209	down
Mllt4	0.001252045	3.550232172	down
D030017L14Rik	0.001252045	2.083675861	down
Cryz	0.001252045	1.538257957	up
Pofut1	0.001252045	1.603772879	up
Pdcd4	0.001321057	8.171095848	down
Twist1	0.001321057	1.775350571	up
Zdhhc21	0.001321057	2.122733355	down
Cnot4	0.001346767	3.769899845	down
Hnrnpk	0.001346767	1.691924214	up
Exosc10	0.001346767	3.312430859	down
Casc5	0.001369625	2.576614142	down
Zzz3	0.001384872	4.973208904	down
Eif3a	0.001384872	2.08261323	down
LOC386164	0.001387765	4.183255673	down
Mfhas1	0.001387765	1.77922082	down
Expi	0.001431931	2.290077686	up
Atp7a	0.001447044	3.527199984	down
Gm1943	0.00146609	1.800988078	down
Gpnmb	0.00146609	3.003995895	up
Masp2	0.00146609	4.408613682	up
scl0001118.1_0	0.001481147	4.556073189	down
LOC381445	0.001481147	2.828824043	down
B130065G19Rik	0.001481147	1.761445165	down

B230339C08Rik	0.001481147	2.219897509	down
2410089E03Rik	0.001493363	2.022233725	down
4922502B01Rik	0.001505361	2.130442858	down
LOC229810	0.001505361	3.544888496	down
Slitrk5	0.00151972	1.690063119	down
Catnb	0.00151972	4.65704155	down
Slc4a7	0.001544275	3.262524128	down
Casp8	0.001544275	1.512689591	up
C87860	0.001548931	2.352594614	down
Tmod2	0.001548931	1.810086608	up
Sfrs11	0.001548931	4.205660343	down
A230077I10Rik	0.001548931	2.612783909	down
Rn18s	0.001548931	28.15727615	down
Cited2	0.001548931	1.61139214	down
Adssl1	0.001548931	2.195277691	up
LOC636687	0.001548931	2.30185461	down
2600011E07Rik	0.001548931	4.089639187	down
Nrxn2	0.001548931	3.475767374	down
Hmox1	0.001548931	1.975618482	up
A530089A20Rik	0.001566832	3.25717783	down
scl0001284.1_18	0.001566832	4.112894058	down
Phf20	0.001566832	2.636844873	down
D030064C08Rik	0.00158621	2.240285873	down
Rai1	0.001596302	2.476820707	down
2210008N01Rik	0.001599585	1.590283632	up
Prpf40a	0.001622462	6.006502628	down
4833426J09Rik	0.001622462	3.022704125	down
4832404P21Rik	0.001622462	1.580677509	down
Mll3	0.001622462	2.042486906	down
Fgf10	0.001622462	2.72319746	up
Satb1	0.001622462	2.155455589	down
Asxl3	0.001622462	3.112105608	down
Thsd4	0.001622462	3.020121813	down
C730009D12	0.001622462	2.332720518	down
Mfap3	0.001622462	2.062714338	down
9530082I15Rik	0.001622462	2.436363936	down
Cyfip1	0.001622462	1.673766494	up
2310043D08Rik	0.001639069	2.310451031	down
Nfic	0.001639069	2.677012205	down
Pdpk1	0.001639069	2.793810844	down
EG667728	0.001639069	4.284719467	down
Tgs1	0.001639069	2.577982903	down
Gcc2	0.001639069	2.556368351	down
Uimc1	0.001649925	1.521978378	down
Chd7	0.001654211	3.721209765	down
Tcfap2b	0.001671571	5.70921278	down
Gramd3	0.001699621	1.683848739	up
Hist1h2ai	0.001699621	3.275393248	down

Grhl2	0.001699621	1.951004982	up
0610007P08Rik	0.001699621	1.85313654	down
D130078K04Rik	0.001699621	2.518232822	down
Cttnbp2	0.001699621	2.236723423	down
Ctdsp2	0.001699621	1.619929195	up
4930527B16Rik	0.001699621	2.372690678	down
Pqlc3	0.001699621	1.66099906	up
B230386D16Rik	0.001699621	3.909979105	down
Zswim6	0.001699621	3.327031136	down
Bat2d	0.001699621	11.11145401	down
Spag9	0.001699621	2.312184811	down
Arl5b	0.001699621	1.976490736	down
D230029K13Rik	0.001699621	1.632989049	up
C130064E22Rik	0.001699621	2.518179178	down
Vmn2r-ps14	0.001699621	3.321276426	down
Actb	0.001699621	2.419744492	down
Hist1h2ah	0.001699621	3.9784832	down
Gas7	0.001699621	2.229600668	up
Ntng1	0.001699621	2.279101133	up
Bach1	0.001699621	3.790873051	down
Zfp553	0.001699621	1.862444997	down
LOC386144	0.001699621	3.415578842	down
Rif1	0.001699621	2.582983732	down
2310032M22Rik	0.001699621	2.79638052	down
Tshz1	0.001699621	2.919794083	down
LOC668492	0.001699621	1.59711802	down
A330049H05Rik	0.001700977	6.968859673	down
St8sia5	0.001700977	2.520494223	up
Trim33	0.001714952	2.696403503	down
Col5a2	0.001714952	1.793847203	up
Rlf	0.001714952	2.288247347	down
Gpr106	0.001714952	2.19508791	up
Rapgef5	0.001714952	4.095921993	down
Zfp553	0.001714952	2.516846895	down
D430007A19Rik	0.001714952	3.118346214	down
Hoxa7	0.001722037	2.206652403	down
Ebf1	0.00172265	3.610209703	down
3110007F17Rik	0.001776419	3.020109892	down
6430590A07Rik	0.001800553	1.660326004	down
A030010B05Rik	0.001817547	1.777314305	down
Pcyt1a	0.001817547	3.771603584	down
Tns1	0.001817547	1.844639897	down
B230323D24Rik	0.001817547	1.872745156	up
Ubn1	0.001817547	1.646087647	down
A430090G16Rik	0.001817547	2.383386374	down
Nkd2	0.001873706	2.789045095	up
C330006A16Rik	0.001873706	1.781713247	up
Braf	0.001876352	1.763750553	down

D230020C06Rik	0.001876352	2.677211046	down
Reep3	0.001902016	4.164883614	down
Peg3	0.001906506	3.412580729	down
D330022O09Rik	0.001923039	2.995478153	down
Cd55	0.00193676	1.56550014	up
Col20a1	0.001973813	4.662111282	up
Mki67	0.001973813	1.912924767	down
C130022E19Rik	0.001983357	2.944473267	down
Rere	0.001993988	2.487741947	down
D9Ertd402e	0.001993988	2.257170439	up
LOC381891	0.001993988	1.665171385	down
Tpm1	0.001993988	2.47113204	up
Atp10b	0.001993988	4.808668137	up
1300010F03Rik	0.001993988	1.556489348	up
Mfap2	0.001993988	1.812550783	up
Dcc	0.001993988	2.583023071	down
Ube2b	0.001993988	1.501930594	up
Nvl	0.001993988	2.314384937	down
Slco5a1	0.001993988	1.964363217	down
LOC671878	0.001995887	6.171885014	down
2610319H10Rik	0.002000658	2.309181452	down
Srrm1	0.002000658	1.568955302	down
B230208H17Rik	0.002000658	3.07472229	down
2810408P10Rik	0.002000658	2.048011303	down
9845300_5100	0.002000658	1.742226481	up
Hist1h2ak	0.002000658	3.852180481	down
Bat2d	0.002000658	11.87066841	down
Ppp1r14c	0.002000658	1.737563252	down
B430201A12Rik	0.002000658	3.403898001	down
Rnf122	0.002000658	2.272558212	down
B230213E18Rik	0.002000658	2.527683497	down
Rufy2	0.002000658	2.299056053	down
2010007K12Rik	0.002000658	3.044680834	down
Zfp148	0.002000658	2.152908802	down
2600005C20Rik	0.002024822	2.9771595	down
Rnf135	0.002067311	1.67632401	up
Rsrc2	0.0021475	2.5628438	down
Rasip1	0.002152312	1.670943975	up
A930019L04Rik	0.002152312	2.277096272	down
LOC226017	0.002152312	1.719550371	down
LOC100041569	0.002152312	2.351627588	down
4933417E08Rik	0.002152312	2.430617333	down
LOC100046781	0.002152312	2.830364466	down
A930004J17Rik	0.002152312	2.8519454	down
Thsd1	0.002152312	1.963858008	up
Col28a1	0.002152312	2.050136805	up
Egr2	0.002152312	1.968085527	up
6530443I23Rik	0.002152312	2.353471518	down

Msi2h	0.002155256	2.21756196	down
Psmd11	0.002236816	2.703939676	down
Bcor	0.00229542	1.938883066	up
scl0002975.1_346	0.002297732	6.940559864	down
1200016B10Rik	0.002344545	1.812214136	down
Ddx26	0.002366696	2.24638629	down
Peg3	0.002366696	3.523910761	down
Csrp3	0.002366696	3.043993473	up
Klhl5	0.002408065	1.673710585	down
Stat3	0.002408065	4.546752453	down
Mark3	0.002408065	2.442866087	down
Znrf1	0.002408065	2.388609886	down
Sfrs5	0.002424738	2.535691977	up
Them4	0.002424738	1.536995053	up
LOC676704	0.002424738	2.788088083	up
Msx1	0.002424738	2.461048126	up
Lgals4	0.002424738	1.728363872	up
Ift80	0.002424738	1.537944078	up
Grik1	0.002436229	1.907122612	up
Lhfpl2	0.002439372	3.675370693	up
2310007F21Rik	0.002439372	1.938352227	down
Tecta	0.002439372	2.851201773	up
EG627022	0.002449249	1.757732272	up
Trim2	0.002516103	2.939060688	down
LOC100043821	0.002520246	4.291506767	down
Ezh2	0.002520495	3.44827652	down
BC028528	0.002520495	1.583291173	up
Ddx21	0.00253616	2.607344866	down
LOC100046483	0.002539726	2.432278872	down
Gal3st2	0.002564475	1.659373522	up
LOC333917	0.002637185	1.822413206	up
Ap1g1	0.002644724	1.829310775	down
1810013B01Rik	0.002644724	1.71312809	up
Stard4	0.002678604	2.152586222	down
Ppm1l	0.002678604	1.917000532	down
LOC100048816	0.00269998	1.705911994	up
Nup188	0.00269998	1.950923562	up
Hoxb4	0.002712341	9.586476326	down
Gm484	0.002712341	2.060320854	up
2700055A20Rik	0.002747602	1.654114246	down
LOC386298	0.002775091	8.970158577	down
Stx4a	0.002784912	1.667314172	up
Zcchc17	0.002784912	2.049758911	down
Syne2	0.002797721	3.046834946	down
LOC100045679	0.002813986	2.550547361	down
Alox12b	0.002833908	1.721888423	up
Appbp2	0.002839923	2.40660739	down
Ncapg	0.002844835	1.8440516	down

Iqgap1	0.002852635	2.730445385	down
Scrt2	0.002852635	3.080980301	down
Efhd1	0.002852635	2.056644678	up
Trf	0.002852635	2.420217037	up
Xrn2	0.002852635	1.805908084	down
Eif4ebp2	0.002852635	3.025116205	down
Cbx3	0.002865495	3.409720898	down
Pros1	0.002878058	2.084843397	up
LOC100046781	0.002878058	3.634115934	down
1700049G17Rik	0.002886125	2.146261931	down
A930007B11Rik	0.0029021	2.288319111	down
Camk2b	0.002968505	3.404276133	up
Sox5	0.002973807	4.62940979	down
C8b	0.002977562	2.156763315	up
Blmh	0.003005815	1.692749858	up
Zfp668	0.003005815	1.656523585	down
2600009P04Rik	0.003036049	2.49938345	down
2900083I11Rik	0.003036049	2.954364061	down
Gdnf	0.003044692	2.697883368	up
Igfbp4	0.003059143	2.211507082	up
2010321I05Rik	0.003059143	2.100106001	down
LOC385653	0.003059143	1.824099422	down
Rasal2	0.003085814	2.635222197	down
Trappc3	0.003092976	2.015714407	up
LOC385063	0.003092976	1.538074255	up
Depdc6	0.003097231	2.535854816	up
Zbtb8os	0.003097231	2.524297953	down
Clic4	0.003098282	1.885798574	down
Fgfr1op	0.003100789	2.712619543	down
Odz2	0.003101471	2.188945532	down
Gad1	0.003101471	3.517460346	down
Sox10	0.003101471	4.689880848	up
Cort	0.003101471	2.570096016	up
Fgf10	0.003101471	2.86425519	up
IGFBPL	0.003101471	2.645238161	down
Cdc42	0.003105573	1.699200034	up
Grip1	0.003105573	1.532074928	down
Golga5	0.003105573	1.562871337	up
Elovl6	0.003105573	3.462438345	down
Aatk	0.003105573	2.335288048	up
9030016H15Rik	0.003105573	4.149542332	down
Pdap1	0.003151634	5.496873379	down
Nsd1	0.003151634	2.560655594	down
Ctgf	0.003151634	5.581324577	down
LOC234486	0.003196267	1.624858975	up
Cnot6l	0.003196267	1.637559295	down
Adamts16	0.003196267	1.956285119	up
Mafg	0.003206996	2.137374401	down

C730026O12Rik	0.003226428	2.757518053	down
Tnrc6b	0.003232072	1.850290775	down
Krt23	0.003236658	2.035083771	up
St8sia1	0.003291511	1.859755993	down
Ngfr	0.00330888	3.533227444	up
A230057M07Rik	0.003358118	2.462555409	down
Gbf1	0.003362038	2.705598354	down
LOC386268	0.003362038	1.891545653	down
LOC385985	0.003362038	1.871003985	down
Syt9	0.003362038	1.731947303	up
Mx2	0.003362038	1.552976728	up
Socs2	0.003362038	2.229184389	down
Slc30a3	0.003392438	3.175337076	down
Sfxn5	0.003392438	1.69588387	up
C730037N04Rik	0.003392438	1.883291602	up
LOC382128	0.003392438	2.909168959	down
Dem1	0.003392438	1.932738781	down
Nssr	0.003392438	3.501837015	down
Rplp2	0.003392438	1.905174494	down
LOC384806	0.003400741	1.686281681	up
9430091F09Rik	0.003424362	2.187004089	down
Si	0.003476268	6.297748566	up
3110045I18Rik	0.003482583	1.833001494	down
Thsd1	0.003494327	1.874257326	up
Pdgfrl	0.003520094	1.941183329	up
D330046C14Rik	0.003520094	1.876979828	down
Pigk	0.003565899	2.236676931	up
Sox5	0.003586253	1.902192593	down
Zfp106	0.003587402	2.108716726	down
Ccnd2	0.003587402	4.043251991	down
Ophn1	0.003587402	1.766860843	down
LOC100039590	0.003587402	1.735718012	up
LOC277385	0.003587402	1.559720755	down
Ankhd1	0.003587402	4.114300728	down
Emilin2	0.003587402	1.918661356	up
LOC332100	0.003593922	2.07954073	up
Cpne4	0.003619366	1.857071757	up
6330408A02Rik	0.003619366	1.517202377	up
LOC665047	0.003623013	1.717778564	up
Dynlt1	0.00364717	1.645379782	up
Tmem159	0.003718815	1.886527419	up
Mcoln3	0.003725343	2.695793152	up
Trfr2	0.003746883	1.591099858	up
C330013J21Rik	0.003755712	1.60490334	up
5730507C05Rik	0.003784398	1.865052462	up
Hist1h2ag	0.003803332	4.092349052	down
Sema3e	0.003851038	1.625473976	up
A430103B12Rik	0.003851038	2.11023283	down

Tdrd6	0.003851038	1.922942162	up
Phf20	0.003851038	2.595990419	down
E330034G19Rik	0.003851038	2.091925383	up
Wisp1	0.003851038	1.826170802	up
Mtmr11	0.003852484	1.810917616	up
Gprc5b	0.003852484	1.710953713	up
Slc45a2	0.003852484	1.913041115	up
Dio3	0.003852484	2.048040152	up
Olfml1	0.003852484	2.11557436	up
4833426J09Rik	0.003854741	3.128195763	down
Usp1	0.003873624	2.097961426	down
4930565D16Rik	0.00387948	1.980524778	up
Lysmd1	0.00387948	1.503440857	down
Odc1	0.00387948	1.588709831	down
Depdc6	0.00387948	1.628386498	up
Ddx31	0.00387948	1.615046024	up
LOC386218	0.00387948	2.067317009	down
Slc26a4	0.00387948	1.762005448	up
D230019K20Rik	0.00387948	2.04913044	down
Golga2	0.00387948	3.592981577	down
Prss12	0.003890534	3.801356077	up
Xkr6	0.003910657	1.549505115	down
Hamp2	0.003910657	1.9332546	up
Gabarap	0.003955028	1.600666881	down
Tkt	0.003988665	1.707558632	up
2700069A02Rik	0.003988665	1.632886529	up
Postn	0.003988665	2.306335211	up
Axl	0.003988665	1.744459152	up
2310047A01Rik	0.003988665	2.222008228	up
C330008K14Rik	0.003988665	2.10133791	up
Ptbp2	0.003988665	2.669814825	down
Mdk	0.003988665	1.70645988	up
Fmn2	0.003988665	1.803737402	down
LOC386002	0.003989679	1.763634682	down
Ctdsp2	0.003989679	1.791823387	up
Gs2na-pending	0.003989679	1.732313633	down
LOC100040573	0.003989679	1.949356914	down
LOC628101	0.003989679	2.467933178	down
Fmn2	0.003989679	1.898942232	down
Fgfbp3	0.003996336	3.325478792	down
Elmod2	0.00400336	1.708793163	up
Ccnd2	0.004006404	3.149492264	down
Top2a	0.004030433	2.437822819	down
Pkp2	0.00403371	1.564621568	up
Prss12	0.00403371	3.068991184	up
4921506J03Rik	0.004036784	2.013284206	down
Eif5	0.004040268	2.383437872	down
A130004B21Rik	0.004042468	1.808148742	down

Rhbdl3	0.004042468	2.234582424	down
Dlx1	0.004042468	4.123897076	up
Rab3c	0.004064715	3.192802429	down
Ets1	0.004064715	3.387423277	up
Hist1h2ad	0.004064715	3.079777718	down
Afap1l1	0.004069591	1.846763253	up
B230345P09Rik	0.004103392	2.239587784	down
A930010C08Rik	0.004103392	1.50810039	up
Pitpnm3	0.004104899	1.629771113	up
9630055A16Rik	0.004105434	1.84425199	down
Stk40	0.004106835	1.524536729	down
OTTMUSG00000004551	0.004134541	2.317496061	up
Zfp445	0.004135276	6.528080463	down
Palmd	0.004150326	2.123199225	up
LOC386179	0.004173989	3.148241043	down
Plxnb3	0.004173989	2.010650873	up
D230034L24Rik	0.004173989	2.333503723	down
Nasp	0.004173989	3.257022858	down
Il28ra	0.004173989	2.330181122	up
Masp1	0.004173989	3.221175671	down
4930400K19Rik	0.004173989	1.715313435	up
Stag3	0.004197383	1.781137586	up
Raf1	0.00422702	2.488537073	down
Adamts18	0.00424145	2.398599625	up
D930024N12Rik	0.00424468	2.204247713	up
Pdzd2	0.00424468	1.697885037	up
Hist1h2af	0.00424468	3.017111778	down
Ndst1	0.00424468	1.687913656	down
6030408C04Rik	0.00424468	1.727607727	down
B830007D08Rik	0.00424468	1.861119866	down
Sipa1	0.00424468	1.815942884	up
Il15	0.00424468	1.798205018	up
C130040D06Rik	0.00424468	1.62017858	down
4931403I22Rik	0.00424468	1.816438913	down
3110007F17Rik	0.004260927	2.347302675	down
Celsr2	0.004260927	3.531636953	down
2210018M11Rik	0.004279594	1.521722674	up
C030014C12Rik	0.00428007	2.566443443	down
Lcn8	0.00428007	1.591693759	up
Hnrnpa2b1	0.00428007	1.727271676	up
Adssl1	0.00428007	2.078445435	up
scl0070954.1_214	0.00428007	1.957226753	down
Rsrc1	0.00428007	4.078332424	down
Slc45a2	0.00430863	1.967946529	up
Orc2l	0.00430863	1.668040276	up
Ypel5	0.004327614	1.505259752	down
1200008A14Rik	0.004333043	2.136365891	down
Nnmt	0.004333043	2.223613977	up

HPBRII4	0.004333043	2.380132437	down
D930014N22Rik	0.004333043	1.923139691	down
Sep-03	0.004333043	3.362284899	down
A230057G18Rik	0.004342289	2.102051497	down
Nrxn3	0.004357657	2.003535509	down
Psmb2	0.004357657	1.822611094	up
Mex3a	0.004363656	3.356859446	down
LOC100043402	0.004363656	7.32914114	down
Rufy3	0.004363656	1.619865298	down
Ffar3	0.004363656	1.757079363	up
3300001P08Rik	0.004363656	2.557412148	down
Fnip1	0.004363656	1.972114444	down
Nrxn3	0.004363656	3.034490824	down
2310057M21Rik	0.004363656	1.765078068	up
Casp2	0.004368847	1.834133387	down
Nfe2l3	0.004368847	1.547719479	up
Klf12	0.004368847	1.812991619	down
Dnajc7	0.004368847	1.837562919	up
Wdr6	0.004368847	1.67057097	down
9030612M13Rik	0.004381892	1.901643038	down
Mrgprf	0.004392166	1.802424312	up
Ralb	0.004395306	1.624117613	up
Arhgap24	0.004425271	1.760208368	up
scl0002690.1_1	0.004434391	1.926185727	up
Nalcn	0.004438524	1.715703368	up
4921518A06Rik	0.004442342	1.753007293	down
LOC386360	0.004457909	2.948600054	down
LOC386199	0.004457909	7.661334992	down
Tnfrsf19	0.004457909	1.982052088	up
Lipf	0.004457909	3.143088102	up
Nlrp14	0.004460373	1.65374589	up
Hectd1	0.004460373	6.389221191	down
Mll3	0.004460512	2.595492125	down
Pten	0.00447098	2.432378292	down
Ep300	0.004473693	2.288798809	down
Chd4	0.00449493	2.537227631	down
C030009J22Rik	0.00449493	1.791762471	up
scl0001259.1_60	0.004500159	1.783114553	up
H2afy2	0.00450229	1.929085732	up
9030227G01Rik	0.00450229	1.633226037	down
6330404F12Rik	0.00450229	1.706807613	down
Alx3	0.00450229	1.506923556	up
Rab7l1	0.00450229	1.946507812	up
Kif15	0.004538247	1.9450984	down
Pdlim1	0.004565872	3.782613516	up
Eif4a2	0.004565872	1.96769321	up
Btn1a1	0.004571454	1.923624635	up
Rrp1b	0.0045816	2.953103781	down

Meis1	0.0045816	2.1638093	down
A930002H24Rik	0.004586838	1.719869375	up
LOC100048413	0.004586838	1.599472284	down
Usp33	0.004586838	3.03154397	down
Stat6	0.004586838	1.68626821	up
Slco3a1	0.004586838	1.509677291	up
Fv1	0.004586838	1.869678617	up
LOC381151	0.004597899	1.573311806	down
Fzd5	0.004597899	3.699457407	up
Angptl4	0.004597899	2.096686363	up
Fgfbp3	0.004597899	4.066598415	down
Bach2	0.004597899	3.964662314	down
Gpr17	0.004597899	3.242673874	up
Gfra3	0.004598572	3.552607775	up
Rbbp2	0.004598572	1.731868744	down
Cd200	0.004601521	2.365979672	up
LOC381739	0.004603106	1.69522655	down
Aldh1a3	0.004627538	2.671280623	up
Bcam	0.004627538	1.691012144	up
Mtap6	0.004627538	2.106295824	down
Cyp39a1	0.004627538	1.605235815	up
Wdr5	0.004627538	1.588445306	down
LOC100046883	0.004655968	2.968738556	up
4933439C20Rik	0.004655968	3.286760092	down
Hist1h2af	0.004655968	4.633497238	down
Snx27	0.004655968	2.50328517	down
Gna13	0.004667085	2.552011967	down
Ppp3ca	0.004671362	1.813813686	down
A830092P18Rik	0.004671362	1.51265657	down
Smpx	0.004689072	1.57802403	up
Zbtb43	0.004705825	1.869483829	down
Gab3	0.004711352	1.770696521	up
Stard5	0.004716454	1.91793406	up
2310043I08Rik	0.004716454	1.756198764	up
1810043M20Rik	0.004734241	2.402331591	down
C730004I03Rik	0.004734241	2.080106497	down
Dhh	0.004734241	3.853497744	up
Usp37	0.004734241	2.824853897	down
Tapbp	0.004734241	1.739006877	up
Auts2	0.004743735	3.004128218	down
Ddx58	0.004751281	1.568745971	up
Adrb2	0.004757195	1.594719172	up
Meg3	0.004757195	2.333277702	down
LOC236060	0.004776551	2.06487155	up
Adamts18	0.004776551	2.357272148	up
EG232599	0.004785434	1.530244112	up
Skap2	0.004795559	2.074286223	down
A430037M23Rik	0.004814238	1.548640847	down

Efcab5	0.004824178	1.894885778	up
Srrp	0.004828567	1.715488672	up
Htra1	0.004829389	2.11162734	up
LOC100047261	0.004832887	2.417116404	up
Cthrc1	0.004832887	1.708385348	up
Hkdc1	0.004842994	1.629693627	up
Rttn	0.004842994	2.071027756	down
Usp1	0.004850945	2.008660793	down
Sox11	0.004870411	2.488309383	down
A930006A19Rik	0.004893755	1.86356926	down
Pdcd5	0.004893755	1.530370951	up
C230075M21Rik	0.004893755	1.976967335	down
Phf21b	0.004893755	1.757664919	down
Tmc6	0.004893755	2.202062845	up
Pscd3	0.004893755	1.602603078	up
Eif4e	0.004893755	2.347019672	down
Pkig	0.004893755	2.022256851	up
Fmo1	0.004893956	3.091035128	up
Olig1	0.004897203	5.111002445	down
Gatad2b	0.004900901	2.021556139	down
4632427E13Rik	0.004912466	2.964967728	down
Insc	0.004926327	6.45871973	up
6030446B09Rik	0.00494496	1.571084023	up
Robo1	0.004948861	1.873947024	down
Sostdc1	0.004948861	2.06722784	up
Tcerg1	0.004948861	1.740078449	down
Vsnl1	0.004948861	2.174836397	up
Cilp2	0.004948861	1.771610737	up
Gas5	0.004948861	1.75621593	up
2810474O19Rik	0.004948861	5.535286427	down
Aldh3a2	0.004948861	1.929371238	up
Zfp326	0.004948861	2.000721216	down
BC030863	0.004948861	1.570042849	up
Chpf	0.004948861	1.853567362	down
4833414E09Rik	0.004948861	2.671931267	down
Nipbl	0.004953511	2.361480951	down
Rbmx	0.004961778	1.734710574	down
1520401A03Rik	0.004973099	1.746812582	up
Actl6a	0.004973751	2.658664465	down
LOC384362	0.004981522	1.816571236	up
Nol3	0.004981522	1.722426415	up
Col6a1	0.004981522	2.340614796	up
Vwa2	0.004981522	1.675891042	up
LOC245668	0.004991073	1.703509927	down
Gss	0.004998614	1.652871847	up
Pcdh19	0.005000604	1.512954831	up
Pole2	0.005042526	1.681133509	down
Cldn2	0.005042526	1.802921772	up

LOC382362	0.005050725	1.586464882	up
Dscr3	0.005050725	1.569319129	up
Slc7a11	0.005050725	1.724648714	up
Adamts9	0.005050725	2.108854771	up
LOC383078	0.005050725	1.950703144	up
C130071C03Rik	0.005050725	1.865056992	down
B130018P07Rik	0.005050725	1.690133095	down
Ahnak	0.005093016	1.763386846	up
Klc1	0.005121896	2.019265175	down
Ctf1	0.005124364	1.802195549	up
LOC100047583	0.005138603	2.430857182	up
D230021F18Rik	0.005140017	1.867432475	up
Nfic	0.005145176	1.655638099	down
Agtrap	0.005146599	1.963665724	down
Mllt10	0.005146599	2.038018942	down
BC031575	0.005168764	2.099843264	down
Metapl1	0.005185254	1.774073601	down
Hoxb3	0.005185254	2.653430224	down
A330078B09Rik	0.005185254	1.544308305	up
4930429B21Rik	0.005185254	1.868144989	up
Lmo4	0.005185254	2.143743992	down
Cgrrf1	0.005185254	1.553932667	up
Pdik1l	0.005185254	1.953572273	down
Tcf12	0.005185254	2.977221966	down
1700094D03Rik	0.005198562	1.54252398	down
Zfp106	0.005198562	1.973884344	down
Trhde	0.005198562	2.109780312	up
Mup2	0.005198562	1.523922443	up
Lrig1	0.005203011	1.621649146	up
LOC331507	0.005203011	2.20334053	down
Rapgef5	0.00520531	1.954174519	down
A030011F13Rik	0.00520828	1.653781176	down
Slc35f4	0.005210541	1.797384501	up
Chl1	0.005226567	1.921963215	down
Slc4a8	0.005226567	1.742372155	up
6330505N24Rik	0.005229767	2.759076119	up
1200002N14Rik	0.005237064	1.83902657	up
Sec22c	0.005247693	1.569401503	down
Best1	0.005259762	1.740502596	up
Wbscr14	0.005262208	1.878883004	up
Parp14	0.005262208	3.012121201	up
Rtn4	0.005262208	1.835431218	up
Vamp8	0.005262208	1.797014237	up
Cdh19	0.005262208	2.516232729	up
A730098D12Rik	0.005262208	1.594929934	down
Uchl3	0.005262208	1.79935813	down
LOC386117	0.005262208	2.390728474	down
Neto1	0.005262208	1.735059023	up

Edg5	0.005262208	1.766367674	up
Afap1l2	0.005285901	1.825451851	up
Meis3	0.005285901	1.73090148	down
Rem2	0.005285901	1.555891156	down
D630043K02Rik	0.005285901	1.862826586	up
Zfp131	0.005293127	1.94246161	down
Brd2	0.005293809	3.862645149	down
Klf7	0.005293809	2.45822978	down
Cck	0.005326394	1.699229956	up
Lmbr1	0.005326394	2.06909132	down
Mtap2	0.005326394	2.897240162	down
Csnk1g3	0.005331163	1.628932357	down
9430064K01Rik	0.005331163	2.687591076	down
B230340J04Rik	0.005338991	1.56095326	down
Kpna1	0.005357154	1.551834703	down
Trp53i11	0.005358178	2.236171961	down
2610030H06Rik	0.005358178	2.189422607	down
Plekha5	0.005381373	1.954667211	down
Cnih4	0.005397065	2.013272047	down
LOC383101	0.005399176	1.850438118	up
Snap23	0.005403684	1.558082461	up
A830009P14Rik	0.005412018	1.590291023	up
Zmiz1	0.005412018	2.562799931	down
C430002P19Rik	0.00541515	1.639683843	up
scl0004190.1_3	0.005415535	5.637984276	down
2610034M16Rik	0.005427	2.208107948	up
Meis1	0.005427	1.565403462	down
Mfap5	0.005427	2.247203112	up
Ryr3	0.005427	1.800640822	up
Ppfibp2	0.005427	1.914390922	up
Lin7a	0.005427	1.879538298	down
Rnf135	0.005427	1.93558228	up
B230343A10Rik	0.005427	3.068618298	up
Tmc6	0.005427	2.330271721	up
Heph	0.005427	2.259020329	up
Rbm8a	0.005427	1.543013811	down
Nktr	0.005431587	2.454688311	down
C130023A14Rik	0.005506493	2.062721014	down
4921505C17Rik	0.005506493	1.628192067	down
Zfp1	0.005506493	1.69206357	down
Palmd	0.005506493	2.522102594	up
Ier3	0.005507129	1.763753891	up
Oxr1	0.005516001	1.74464941	up
Epb4.1l2	0.005516572	1.960246444	down
Crybb3	0.005529006	1.63102138	up
A630064D23Rik	0.005544541	1.728334665	up
Trappc3	0.005557003	2.023467064	up
Srpk3	0.005565666	1.944763541	up

Rcn3	0.005565666	1.58309114	up
Gadd45g	0.005565666	2.253056765	down
Eng	0.005565666	1.68507874	up
2600011C06Rik	0.005594803	2.700891972	down
Pou4f1	0.005600505	3.066137791	down
Metrnl	0.005602825	1.525995374	up
Edg7	0.005602825	1.699133992	up
Mbp	0.005602825	2.937065363	up
Gcs1	0.005602825	1.972009659	down
Zfp820	0.005602825	1.56748879	up
Mapkapk3	0.005602825	1.707570195	up
Ndor1	0.005602825	1.804365635	up
Klhl30	0.005602825	2.35766077	up
Dnajc5b	0.005602825	1.910481453	up
9630028G16Rik	0.005605367	1.684952855	up
EG245651	0.005605367	1.52816689	up
Kif13a	0.005605367	1.524483919	up
Camk2d	0.005605367	1.736002922	up
Pgk1	0.005610062	1.874397755	up
Rbpms	0.005624093	1.854561448	up
LOC100045484	0.005636469	4.588802338	up
Phldb2	0.005636469	1.582591534	up
Enah	0.005636469	2.390565872	down
Pou2f1	0.005640426	1.96668911	down
LOC100047888	0.005640426	2.109181404	down
LOC270589	0.005640426	12.62540245	down
A230057E24Rik	0.005640426	1.830949187	down
Elavl3	0.005640426	1.840135336	down
Fam20a	0.005640426	2.370486736	up
Vezt	0.005640426	1.987881064	down
Aldh3b1	0.005640426	1.684391022	up
Lef1	0.005640426	1.844891191	up
Nme2	0.005657101	1.696917296	up
Sox6	0.005714556	2.446631193	down
Leprel1	0.005714556	1.809220314	up
Tbc1d2b	0.005730511	1.600965858	up
Scg2	0.005730511	2.221801043	up
LOC381524	0.005730511	1.606394172	up
Bcl6b	0.005730511	1.802271843	up
4932408C11Rik	0.005730511	1.609389067	up
Tmprss2	0.005730511	2.118969917	up
Blvrb	0.005730511	1.588738442	up
Heph	0.005730511	3.076563358	up
Robo3	0.005730511	2.499913693	down
Mtrr	0.005742189	1.570049286	up
Mtf2	0.005742189	2.279771328	down
Sft2d2	0.005742346	1.930163026	up
Thsd7b	0.005742346	2.112111569	up

Lgr6	0.005742346	1.99344039	up
Lgi4	0.005742346	3.417974472	up
Abi3bp	0.005742346	2.414456606	up
Gmfb	0.005766725	1.870676756	down
F8	0.005766725	1.691141486	up
Anxa4	0.005766725	1.544162273	up
Copg1	0.005766725	1.927163839	up
D6Wsu116e	0.005769094	1.81710422	up
Egfl8	0.005774987	1.952938795	up
Trim21	0.005780984	1.836067081	up
Zfp804a	0.005794081	1.961005211	down
Gls2	0.005794081	1.619387507	up
Slc22a18	0.005794081	1.672028542	up
5930437C20Rik	0.005794081	1.914514542	down
Setd4	0.005794081	1.749804497	up
C230055K21Rik	0.005794081	2.029025555	down
Acbd4	0.005794081	1.550271988	up
Megf10	0.005794081	3.052629471	up
2210418G03Rik	0.005794081	1.695583224	up
Zfp185	0.005830702	2.167747736	up
Cenpf	0.005904581	2.440191984	down
A330057G13Rik	0.00596557	2.619669676	down
1500034J01Rik	0.006068948	1.630295873	down
C330006D17Rik	0.006077457	2.173792124	down
Tnfsf12-tnfsf13	0.006077457	1.538737059	up
LOC243368	0.006077457	1.941149592	up
Cdh6	0.006077457	4.927514076	up
Col10a1	0.006091377	2.159101963	up
Lrrc15	0.006091377	1.567529798	up
Nudt7	0.006091377	1.800465822	up
Cdkn2b	0.006091377	1.740302086	up
Centd3	0.006091377	2.447141409	up
Lamc1	0.006091377	1.921959519	up
Rnase4	0.006091377	2.284752607	up
Aclp7	0.006120202	1.690021277	up
Olfr66	0.006123345	1.706213474	up
P2rx4	0.006133485	1.720799327	up
AV249152	0.006145213	1.651141524	up
C330018M05Rik	0.006152857	1.703523755	up
Sox5	0.006152857	2.341292381	down
Snx27	0.006152857	2.400335074	down
B130052F17Rik	0.006152857	1.904535532	down
H2afy	0.006152857	1.844942689	down
2310043N10Rik	0.006152857	1.825740695	down
Gzmd	0.006164147	1.56538713	up
6430527G18Rik	0.006164147	2.4160676	down
9530095P18Rik	0.006164147	1.648895979	up
Mboat2	0.006164147	2.754043102	down

Chmp1b	0.006170394	1.647567391	down
Grm1	0.006170394	1.608059526	up
2010001J22Rik	0.006170394	1.755378485	up
Itgb6	0.006170394	1.661608934	up
Adam15	0.006170394	1.86102283	up
Abcc9	0.006183874	1.529268026	up
Sp6	0.006183874	1.741514564	up
Prss18	0.006187691	1.875510454	up
Twistnb	0.006196451	3.508275986	down
Sfrs15	0.006210886	2.035793304	down
Tapbpl	0.006210886	1.579133391	up
Tmsb10	0.006210886	1.593579888	down
Unc5c	0.006210886	1.731092215	down
Itih3	0.006210886	1.998142004	up
BC005624	0.006210886	1.732021689	up
S1pr3	0.006210886	1.749848127	up
Adamtsl3	0.006210886	2.042346239	up
LOC231620	0.006210886	1.805250645	up
9630061B06Rik	0.006220574	1.717678309	up
Acly	0.006220574	1.688016772	down
1110003F05Rik	0.006271072	1.819800019	up
LOC245545	0.006271105	1.70277071	up
Arrdc1	0.006272468	1.702329874	up
9030607L17Rik	0.006280317	1.715411186	up
LOC674195	0.006280317	1.68887043	up
Txndc1	0.006280317	1.639881969	up
D030029G14Rik	0.006280317	1.692007661	up
Atf3	0.006280317	1.596185684	up
4930529M08Rik	0.006280317	1.572241664	up
B130040C13Rik	0.006280317	1.857302547	up
BC005624	0.006280317	1.620916247	up
1300003B13Rik	0.006285865	1.730767608	up
LOC380844	0.006314359	1.502296686	up
Hapln1	0.006314359	8.148877144	down
Rpe65	0.006323306	1.505985856	up
LOC386288	0.00635571	8.322160721	down
3110050L10Rik	0.006373179	2.385466099	down
Rif1	0.006375426	1.705016971	down
C430014K22Rik	0.006376337	1.992624044	down
Arpc5	0.006381696	2.671667814	up
LOC100044708	0.006383176	1.756627321	up
2700046A07Rik	0.006395905	1.586668968	up
Irf6	0.006396025	4.210696697	up
LOC333751	0.006401515	1.915848732	down
LOC100048331	0.006401515	1.725289702	up
Nmu	0.006405504	1.700039864	up
B130018F13Rik	0.006434305	2.144836903	down
Abhd5	0.006434305	1.856976986	up

Hoxa13	0.006434305	1.560345769	up
Akap12	0.006434305	1.908554912	up
Olfr419	0.006434305	1.833456516	up
Lphn1	0.006434305	2.254355192	down
Nfib	0.006436858	2.964277744	down
E330013P04Rik	0.006436858	1.579671145	up
1700029G01Rik	0.006436858	2.582047463	up
Prom2	0.006442905	1.61573267	up
Scara5	0.006442905	2.956916332	up
Hist1h2ai	0.006458707	2.9195292	down
Dpf1	0.006460445	1.532499075	up
Snx30	0.006492721	1.611051083	down
Tpm1	0.006495196	1.721640348	up
Rasd2	0.006505511	1.682884097	up
Sh3kbp1	0.006505511	1.640888691	up
Ndr3	0.006505511	1.535937429	up
Nktr	0.006518775	3.614581347	down
Mcm7	0.006534044	3.058141708	down
Krt18	0.006534044	1.778751135	up
Plcb3	0.006534044	1.722563744	up
4930579E17Rik	0.006534044	1.539593458	up
Crym	0.006534044	2.929093599	up
Sep-11	0.006534044	1.5313164	down
Ccdc47	0.006534044	1.832548142	up
LOC674706	0.006534044	1.549422741	down
Atp6ap1	0.006534044	1.529991746	up
Cnp	0.006534044	3.567034721	up
2610036L11Rik	0.006559932	1.65525651	down
1700019D03Rik	0.006559932	1.755156875	up
Hsd17b12	0.006583273	1.627995014	down
Alox15	0.006584859	1.722136021	up
B230219J02Rik	0.006584859	1.635278344	up
Mll5	0.006584859	2.700719357	down
Ppp2ca	0.006584859	1.536321878	up
Nell2	0.006600254	2.951565743	down
Adamts1	0.006614545	3.106287241	up
2410008K03Rik	0.006629752	1.837318778	up
D11Lgp2e	0.006629752	1.724197388	up
2310014F06Rik	0.006629752	2.257092238	up
LOC666038	0.006629752	1.760875225	up
scl0003901.1_33	0.006629752	1.526523948	up
E2f2	0.006629752	1.762496948	down
Atf7ip	0.006629752	3.180575371	down
4921513O20Rik	0.006629752	1.663370132	up
Sertad2	0.006629752	1.950149417	down
Kcna6	0.006629752	1.818800926	up
Tnfaip2	0.006629752	2.049736977	up
Lpin2	0.006647016	1.651542187	up

Calr3	0.006692842	1.62598145	up
Hist2h2ab	0.006701651	6.196415901	down
Cacna2d1	0.006717011	2.062205315	down
Ctnnb1	0.006725502	1.81031704	up
Zfhx3	0.006725502	2.569363833	down
Zcchc8	0.006725502	1.553623915	down
Ssh3	0.006725502	1.629922748	up
LOC383301	0.006725502	1.956738472	up
Hebp2	0.006725502	1.914132834	up
Zfp326	0.006725502	2.138542891	down
Phyhipl	0.006725502	3.186303616	down
Helz	0.00675097	2.73856616	down
EG433144	0.006774929	1.598647475	down
Tcfcp2l1	0.006782831	1.967756152	up
5730455P16Rik	0.006791696	1.612658024	down
Capns2	0.006791696	1.841194511	up
4932441P04Rik	0.006791696	1.615100503	down
Tub	0.006791696	1.646720886	up
Wnk4	0.006791696	2.388968945	down
5930429A15Rik	0.00680544	1.631694913	up
Cd74	0.00680544	1.822950959	up
Fam19a4	0.00680544	1.991144896	up
Araf	0.00680544	1.668467164	down
Papln	0.00680544	1.908900261	up
LOC278188	0.00680544	1.561708689	up
Klhl21	0.00680544	1.577566743	down
C130002K18Rik	0.00680544	1.701230407	down
Sep-03	0.006808474	1.992466807	down
Neurod2	0.006810492	3.529103756	down
Cyfip2	0.006810492	2.251601219	down
Ccdc116	0.006810492	1.669013143	up
Slc5a7	0.006810492	1.691467285	up
Moxd1	0.006810492	2.806173563	up
Rn18s	0.006810492	5.893964768	down
Rad51c	0.006810492	1.950005174	down
Abcc9	0.006818637	1.902769804	up
Xrn2	0.006818637	1.667892456	down
Ssbp1	0.006818637	1.507613778	up
Phactr1	0.006820121	1.801604271	up
CKLF5	0.00682967	1.83629787	up
Hoxd4	0.006832071	4.644217968	down
Fbxo30	0.006833334	1.701165438	down
Nol3	0.00683459	1.77370882	up
Angpt2	0.006839463	1.679457068	up
Lmbr1l	0.006839463	2.864704847	up
Unc84a	0.006839463	1.602512956	up
Cds1	0.006839463	2.031857491	up
Maob	0.006839463	2.398277998	up

Rilpl1	0.006844088	1.89732337	up
C430017H16Rik	0.006844088	1.687273145	up
Thsd4	0.006844088	2.118721247	down
Theg	0.006844088	1.89477241	up
Ttbk1	0.006854275	1.811812997	down
Gab3	0.006854275	2.066807032	up
Epha1	0.006854275	2.248092413	up
4833424P18Rik	0.006854275	1.800855041	up
BC040758	0.006854275	1.881813645	up
Camk2a	0.006857767	1.672849536	up
Zfp316	0.006887345	2.404848576	up
Cdh16	0.006887345	1.757674575	up
C730031B13Rik	0.006887345	1.59008038	up
Arsj	0.006887345	2.018283606	down
Trim34	0.006891481	1.660337687	up
Bcl2l11	0.006891481	1.717076063	up
B130005I07Rik	0.006894814	1.776559472	up
LOC218476	0.006894814	1.954159975	up
LOC242235	0.006894814	1.599460244	up
Axud1	0.006900339	1.728597283	up
Fst	0.006900339	1.821462512	up
Abca8	0.006900339	1.695486903	up
Rffl	0.006902471	1.813576341	up
Sall3	0.006902471	2.016688347	down
Pcolce2	0.006902471	4.951421261	down
Casp4	0.006902471	1.708208203	up
Renbp	0.006902471	1.954621315	up
Gm428	0.006905012	1.682952523	up
Mapk13	0.006923934	1.829774261	up
Cdh3	0.006925982	1.991959691	up
4932443E23Rik	0.006941421	1.856648922	up
Bok	0.006944013	1.725970626	up
Hyal3	0.006974815	1.691222668	up
LOC331336	0.006974815	1.923565149	up
Svil	0.006974815	1.883203507	up
EG665806	0.006974815	1.538271427	up
Plaa	0.006974815	1.906274915	down
Olfr437	0.006974815	1.75923121	up
Elf2	0.006974815	1.971977711	down
Fmo5	0.006979238	1.681067348	up
Chrnb1	0.006979779	1.756003737	up
Atp6v1c2	0.006979779	1.659458637	up
D15Bwg0759e	0.006980874	1.640074372	up
Fem1c	0.006980874	2.018807888	down
Styxl1	0.006983957	1.769035101	up
Cygb	0.006983957	2.201939583	down
Ftsj1	0.007030493	1.663455963	up
Hes5	0.007030493	1.800835371	down

S100a16	0.007030493	1.734155178	up
AA536749	0.007030493	1.6500386	up
Stat3	0.007030493	2.110694408	down
Nhlrc2	0.007061729	1.710560799	down
Prox1	0.007061729	1.736420631	down
2810468N07Rik	0.007061729	1.82218039	up
Fhl2	0.007062017	1.584973693	up
A230060F14Rik	0.007062017	1.656142712	up
Msrb2	0.007066617	1.665276408	up
Shisa3	0.00707235	2.274885893	up
5830430H09Rik	0.007092048	1.623436332	up
LOC100046895	0.007110078	1.836684942	down
Zfp667	0.007124276	1.671773434	down
9330159N05Rik	0.007150918	1.969331265	up
Pnpla1	0.007153324	1.512577653	up
Spcs3	0.007154265	1.514374852	up
Crhbp	0.007159021	1.597204566	up
Cxcl4	0.007159021	13.25275803	down
4931403M11Rik	0.007188464	1.84330225	up
Nasp	0.007192605	2.297074795	down
A430023D23Rik	0.00719588	1.716188669	down
Gdap1	0.00719588	1.61664021	up
Cyp4v3	0.00719588	1.596699596	up
Actr3	0.007201419	1.721425533	up
Dpysl3	0.007201419	3.517597914	down
Olfr1012	0.007201419	1.837745905	up
Yod1	0.007206895	2.08818841	down
Itch	0.00721038	1.991962671	down
Rgs9	0.007252101	1.727088451	up
Hdac2	0.007252101	1.909250617	down
Ddx24	0.007255989	2.493500233	down
Shhrs	0.007255989	1.819928527	up
LOC384590	0.007255989	1.794127703	up
Cgnl1	0.007255989	1.691678286	up
Ctcfl	0.007255989	1.633858681	up
Mfap2	0.007255989	1.751133323	up
Vav2	0.007267179	1.586883783	down
Mamdc2	0.007276483	1.944421411	up
St6galnac4	0.007279167	1.586206198	up
Abca8a	0.007279167	2.4294765	up
Nid2	0.007279167	2.354711771	up
Arap1	0.007279167	1.756273747	up
D330038K10Rik	0.007279167	1.515064955	up
Sprn	0.007279167	1.790618658	up
9830169C18Rik	0.007279167	2.056295633	down
Cyp2d22	0.007279167	1.703147292	up
Cast	0.007279167	1.640567184	up
Lpcat2	0.007279167	1.584900975	up

Sox12	0.007292023	1.672815085	up
Bdnf	0.007310998	1.615655899	up
Wnt6	0.007311164	2.307042122	up
LOC100044177	0.00732174	1.856105566	down
2410022M11Rik	0.00732174	1.69636941	up
Bag3	0.00732174	1.983270645	up
4930473A06Rik	0.00732174	1.749178767	up
Slc1a2	0.00732174	1.705502272	up
Cntn6	0.00732174	2.016876459	up
Ccl11	0.00732174	1.968761802	up
Fa2h	0.00732174	1.655112386	up
Mecr	0.00732174	1.605198264	up
Wdfy3	0.00732174	1.845359206	down
Mvk	0.00732174	1.720483065	up
D930016B10Rik	0.007333499	1.795434833	up
4930415O10Rik	0.007334078	1.793777227	up
Serpinf1	0.007334078	2.480190516	up
1500010G04Rik	0.007348674	6.718616486	down
Clstn1	0.007359137	3.314177036	down
Fkbpl	0.007389454	1.572482348	up
Usp53	0.007401914	1.667365432	up
Cd40	0.007411639	1.74896884	up
Arf2	0.007411639	1.555084348	down
LOC100046844	0.007411639	1.75037837	up
Gca	0.007411639	1.53303206	up
Tnrc4	0.007411639	1.768378377	up
Vax1	0.00741622	1.763728619	up
Rassf10	0.007422095	1.730568051	up
Eif4e3	0.007440148	1.622558117	up
C230040D17Rik	0.007440148	1.909767866	down
Zfp462	0.007440148	2.887309313	down
Fam168a	0.007443503	2.017491341	down
Dlx2	0.007443503	2.451479197	up
Sult1a1	0.007447255	1.99036324	up
Galr1	0.007447255	1.568322897	up
Tgfb1i1	0.007453127	1.581996679	up
A130081B01	0.00745955	1.580934048	up
Gtf2ird1	0.00745955	1.828932643	up
Pld2	0.00745955	1.680220246	up
Dncic2	0.00745955	1.769789934	down
Vgll2	0.00745955	1.534909844	up
Cbr3	0.007459671	2.138172865	up
Col18a1	0.007466195	2.501227617	up
Rhbdf1	0.007466195	2.64759326	up
Fbxw4	0.007466195	1.767644286	up
Tptf-pending	0.007466195	1.530443311	up
Ctsr	0.007474311	1.632862806	up
Abhd14b	0.007474311	2.05855608	up

Fmo2	0.007484068	1.892902613	up
Car5b	0.007484068	1.63581562	up
Cd34	0.007484068	1.670154095	up
Asf1a	0.007484068	1.856505036	down
6820406G21Rik	0.007484068	1.692243815	up
Nat10	0.007509483	1.53066957	up
Pdcl3	0.007509483	1.602994084	up
Ky	0.007509483	1.5535568	up
LOC381727	0.007509483	1.716349363	up
Gkap1	0.0075322	3.127353668	down
2900024C23Rik	0.0075322	1.965977311	up
Ntsr1	0.0075322	1.598834753	up
D930030O05Rik	0.0075322	1.947984695	down
Idi2	0.0075322	1.644993901	up
Snx22	0.007533087	1.846445441	up
Mgst1	0.007533087	1.981680512	up
2010300C02Rik	0.007533087	1.583681583	up
Pdzrn4	0.007533087	1.714657188	up
LOC100047712	0.007549857	1.938086033	down
9030607L17Rik	0.007550064	1.810695887	up
LOC238726	0.007560236	1.6189574	up
9626100_224_rc	0.00757332	1.62882328	up
Fignl1	0.007580963	1.58681941	down
Cd44	0.007580963	1.650398016	up
Rasgef1c	0.007580963	1.714436173	up
Clspn	0.007580963	2.254021168	down
LOC382157	0.007580963	3.274966478	down
Nhlh1	0.007580963	1.535525203	up
Hist2h2ac	0.007580963	2.687710762	down
Ddb2	0.007580963	1.699692726	up
Tdp1	0.007584044	1.513340592	down
Irak4	0.007590463	1.620100856	up
Slc25a4	0.007590463	2.276545525	down
Zfp429	0.007592386	1.922096849	up
Cpa6	0.007592386	1.919758916	up
D430020J02Rik	0.007592386	1.765411258	up
Rbm47	0.007598253	1.687064171	up
Slc35f4	0.007609872	2.122945547	up
Dgka	0.007609872	2.415873766	up
Kcna10	0.007609872	1.668903232	up
Npr3	0.007609872	1.855009079	up
Rab14	0.007610234	2.481125593	down
Il13ra1	0.007610234	1.975075603	up
Cdkl2	0.007610234	1.730247855	up
LOC383293	0.007610234	2.054222584	up
Cdh3	0.007612692	1.725340486	up
Gpc5	0.007612692	1.679262877	up
Ugcgl2	0.007612692	1.9065516	up

Unc5h3	0.007612692	1.822271228	down
Prkch	0.007612692	1.788521528	up
3110006E14Rik	0.007629851	2.25151825	down
2410002O22Rik	0.007629851	1.671185613	down
Arhgap21	0.007629851	2.339950562	down
Anxa9	0.007653587	1.68926847	up
Fbn2	0.007673904	1.576816559	down
Psen2	0.007673904	1.833816052	up
Traf1	0.007686668	1.651134849	up
9430073L23Rik	0.007689212	1.595974684	up
Ddx6	0.007691832	1.673915267	down
Sh3tc2	0.007707933	2.7875247	up
7330438C15Rik	0.007717744	1.57926023	up
Jarid1a	0.007717744	1.509106159	down
LOC100046343	0.007721309	5.954205513	down
Ctgf	0.007721309	3.30226922	down
Nnat	0.007727933	1.97736907	down
Meox1	0.007727933	2.52753973	up
Ahnak	0.007727933	2.406894445	up
Cdgap	0.007727933	1.524498344	up
LOC328031	0.007727933	1.745523691	up
Ube2e3	0.007743188	1.698887825	down
LOC382372	0.007752416	1.577971458	up
Tmc6	0.007752416	2.286953688	up
Cttnbp2	0.007752416	1.67754519	down
Sord	0.007752955	1.748370409	up
Fas	0.007752955	1.665715337	up
LOC385923	0.007752955	12.18055344	down
G3bp2	0.007752955	2.240312576	down
Trim40	0.007752955	1.882770658	up
AI480653	0.007752955	1.83018291	up
BC057079	0.007752955	1.598837972	up
LOC665506	0.007752955	1.612027764	up
3110001A05Rik	0.007767999	2.145489216	up
9030003C19Rik	0.007807687	1.511081219	up
Hoxb2	0.007816674	1.67150557	down
Alk	0.007825272	1.786246419	up
2310015A05Rik	0.007825272	1.740077615	up
Dlgap1	0.007825272	1.693743587	up
Glb1l2	0.007825272	1.642053604	up
Nthl1	0.007825272	1.699536085	up
C1qtnf1	0.007825272	1.724541187	up
Dnase1l2	0.007831065	1.528948069	up
AB182283	0.007854151	1.70322156	down
Llph	0.007864648	1.773000598	down
Tnfrsf12a	0.007868233	1.775237918	up
Tsc22d2	0.007868233	1.512153626	down
2010203O07Rik	0.007868233	1.745176435	down

Jarid2	0.007895046	2.334523439	down
B2m	0.007902038	1.536436677	up
Tnk1	0.007904307	1.766756654	up
4831403C07Rik	0.007904307	1.660034657	up
Bbc3	0.007904307	1.583478689	up
Ttyh2	0.007904307	1.741921544	up
Arpc3	0.007929187	1.610699415	up
D230050J18Rik	0.007929187	1.686151862	down
Ptpre	0.007929187	1.711907625	up
Ugt8a	0.007929187	2.305081129	up
Sh3tc1	0.007929187	1.720714808	up
Bcl2l12	0.007929187	2.196415424	up
C630013B14Rik	0.007929187	1.732495785	up
C130069I09Rik	0.007929187	1.549565673	up
Epb4.1	0.007929187	2.568125486	down
Mdga2	0.007929187	1.944506049	down
Emid2	0.007929187	1.697889805	up
4930523M17Rik	0.007929187	1.730317593	up
3321401G04Rik	0.007929187	1.671618223	up
Gabrb3	0.007929187	2.091973782	up
2610302F08Rik	0.007929187	1.775606036	up
Relb	0.007958808	1.69336462	up
Cog4	0.007973351	1.84077239	up
LOC211591	0.007982783	1.802177072	up
H2-T23	0.007982783	1.835627437	up
4933407C03Rik	0.008013042	1.636318803	down
Jam2	0.008013042	1.81919992	down
BC030046	0.008013042	1.688476563	up
Slc17a7	0.008013042	1.624390841	up
Elavl4	0.008016469	5.826591492	down
E130318A13Rik	0.008045985	1.812476873	down
LOC100042427	0.008045985	1.882529974	down
Mst1r	0.008053708	1.614369988	up
Ktn1	0.008053708	1.746398807	up
D530005L17Rik	0.008056264	1.705158114	up
Matn1	0.008056264	17.49271202	down
Pdk4	0.008056264	1.632621765	up
2010001M09Rik	0.008066949	1.624148011	up
Bace2	0.008066949	1.819162846	up
Spon2	0.008066949	2.846888065	up
Nudt7	0.008073842	1.697523594	up
Nit2	0.008073842	2.124674082	up
C2	0.00808227	2.060898304	up
Camk2d	0.00808227	1.501883268	up
LOC100044736	0.008108694	2.259430885	up
Mag	0.008116387	1.843911767	up
1700009P03Rik	0.008131432	2.747610807	down
Pramel3	0.008131432	1.899806261	up

Ash1l	0.008131432	1.631873131	down
D230046H12Rik	0.008131432	2.742769957	up
Myog	0.008146079	1.942202568	up
Dscr6	0.008158859	1.506543756	up
Il13	0.008158859	1.610215068	up
1200008N06Rik	0.008158859	1.67321372	up
Eef2k	0.008158859	1.51352334	up
A830033B12Rik	0.008172254	1.663051128	up
Gucy1a3	0.008180053	1.522813916	up
Map3k5	0.008184159	1.780693173	up
Pear1	0.008188862	1.705453515	up
Itgae	0.008193313	1.626609445	up
Tnfsf13b	0.008194066	1.609321356	up
Ildr1	0.008194066	1.788333178	up
Kcnc2	0.008194066	1.71026814	up
1810031K17Rik	0.008194066	1.666445136	up
0610007P22Rik	0.008194066	1.894989491	up
Zap70	0.008194066	1.654173493	up
Gm1661	0.008194066	1.723530293	up
Il15	0.008194066	1.629272938	up
Slc44a1	0.008194066	1.802622199	up
AI427809	0.008194066	1.684773922	up
Crx	0.00820628	1.709802032	up
Laptm4b	0.008207075	1.735547662	up
Rbmy1a1	0.008207075	1.672153235	up
A130001G05Rik	0.008207075	2.089229584	down
A930033C23Rik	0.008207075	1.590865731	up
Tgfbr3	0.008219182	1.704247832	up
Gfap	0.008232384	1.728727818	up
Ephx1	0.008232384	1.981066227	up
Pou3f4	0.008255162	2.166515112	down
1810062O18Rik	0.008255162	1.619299412	up
D330004O07Rik	0.008266584	2.180675268	up
1700007P14Rik	0.008266584	1.613148332	up
Klk6	0.008266584	1.93628788	up
Scn1a	0.008266584	1.547603011	down
Decr1	0.008266584	2.292868376	up
Cxcl12	0.008266584	2.093463182	up
E130113E03Rik	0.008266584	1.747346163	up
Nid1	0.008266584	2.851094723	up
As3mt	0.008266584	1.656816363	up
Lgals1	0.008266584	1.780216098	up
LOC100046207	0.008266584	1.537519813	down
AI317223	0.008266584	2.557655573	up
Ldb2	0.008266584	2.027406693	up
3732409C05Rik	0.008266584	1.727020264	down
Acyp2	0.008266584	1.970337987	up
Tmtc2	0.008266584	1.857040048	up

LOC100046035	0.008266584	1.927466393	up
Rnu6	0.008266584	2.246017695	down
Dgat2l4	0.008266584	1.606304765	up
8430408G22Rik	0.008266584	1.553248644	up
Mrpl45	0.00826992	1.62528801	up
A130086G11Rik	0.008276437	1.595142365	up
Ubl4	0.008276437	1.597399592	up
scl0002066.1_0	0.00828695	1.799826026	up
Gclc	0.008306993	1.722256184	down
Cnp1	0.008306993	1.889697194	up
Fer1l3	0.008322276	1.75781095	up
Tbc1d2	0.008324308	1.615794063	up
LOC100041722	0.008368284	1.764884234	up
Igf2bp1	0.008368284	3.780094385	down
Nfib	0.008386457	3.720114231	down
Mgmt	0.008426491	1.876641154	up
Hist1h2an	0.008426491	6.114239693	down
Entpd2	0.008429697	2.361177445	up
Krt32	0.008429697	1.717882037	up
Acot3	0.008430935	1.656251311	up
D630026G14Rik	0.008430935	1.820043206	up
9530026P05Rik	0.008430935	1.871360064	up
Fxyd5	0.008430935	1.842514753	up
Ube2v1	0.008430935	1.691733241	down
LOC100041516	0.00843975	3.315711498	down
Tspan7	0.00843975	2.516344547	up
Vmo1	0.008441206	1.792012215	up
Htra1	0.008443809	2.110677004	up
Arvcf	0.008453381	1.665472508	up
Plod2	0.008453381	1.915130138	down
Sds	0.008464897	1.653308392	up
Dusp22	0.008476226	1.864755988	up
Gm239	0.008494746	2.181171656	up
Fancc	0.008497479	1.768537283	up
4833412L08Rik	0.008497479	1.737890244	up
Gm603	0.00850749	1.703496695	up
C130067A03Rik	0.008513128	2.097831726	down
Man2a1	0.008513128	1.847724199	up
Dnajc1	0.008513128	1.504798055	down
D230017C05Rik	0.008513128	1.978462935	down
Stard5	0.008545738	1.781101823	up
Unc13c	0.008549956	1.767547011	up
Stxbp5l	0.00855079	2.166321278	up
Trim66	0.00855079	1.791775823	up
Zfp759	0.008573219	1.723873377	up
Cbx6	0.008578933	1.60664022	up
ENSMUSG00000043795	0.008585238	2.958674192	up
Aard	0.008600202	1.796426177	up

Arhgef10l	0.008600202	1.61591661	up
Gyg	0.008610106	1.733781338	up
Ano4	0.008614907	1.943389058	up
Twistnb	0.008614907	3.384165525	down
Sdk1	0.008632889	2.000249386	up
Nudt7	0.008632889	1.527867675	up
Abca9	0.008632889	1.87240243	up
Nudt21	0.008632889	1.905255437	down
Klk8	0.008632889	1.878411293	up
Mapbpip-pending	0.008632889	2.114540339	up
Tcfap2c	0.008632889	2.427585125	up
Twist2	0.008634337	1.793907762	up
Scg5	0.008642821	1.857169628	down
4930482J15Rik	0.008642821	1.642644048	up
F2rl1	0.008643521	1.919010401	up
Prkar2a	0.008643521	2.038744688	down
C030032C09Rik	0.008644829	1.583710194	up
Opn1mw	0.008644829	1.705514789	up
D930049D19Rik	0.008662812	1.834710956	up
Timp3	0.00867389	1.833983421	up
Nit1	0.008690017	1.75846231	down
3632431M01Rik	0.008690017	2.012543917	down
Dmrt2	0.008690017	1.751652598	up
A530017D24Rik	0.008690017	1.706673503	up
Fam115c	0.008690017	1.934285164	up
Cbfb	0.008690017	1.530669212	up
Rasgrf1	0.008690017	1.944167733	up
Anxa6	0.008690017	1.643191695	up
Cma1	0.008690017	1.626428604	up
Ppcs	0.008690017	1.688676357	up
Vcam1	0.008691782	1.600209951	up
Wisp1	0.008691782	1.771409392	up
Pja2	0.008692887	1.638751626	down
6430524C05Rik	0.008696577	1.834477663	up
Prpf40b	0.008696577	1.96666193	down
Rsad1	0.008702136	1.844706655	up
4921537F17Rik	0.008705314	1.577659965	up
Trpm3	0.008705314	1.723128915	up
LOC280097	0.008705691	5.131315708	down
Vstm2a	0.008705691	1.9843961	up
F430201B04Rik	0.008705691	1.592969537	up
EG434168	0.008705691	2.421325207	down
Cxcl16	0.008706657	1.801973343	up
Sobp	0.008708064	2.54166007	down
Bnipl	0.008752538	1.981240034	up
Wnt5b	0.008765893	2.029637575	up
Ddx56	0.008765893	1.738734722	up
LOC100047645	0.008765893	1.882153153	down

Parp4	0.008766856	1.571952105	up
Chrna1	0.008777285	1.515834332	up
Cbr1	0.008797905	1.87748456	down
F8	0.008797905	1.519818425	up
Lgals8	0.008797905	1.729083657	up
Olfr1436	0.008816095	1.747491241	up
C330034C07Rik	0.008816095	2.522972822	down
A830021G05Rik	0.008816416	1.785650253	down
Nck1	0.008816416	1.675292373	up
Aplnr	0.008816416	1.82122314	up
Nrg1	0.008816416	1.679394722	up
Edil3	0.008819844	1.579653502	up
Spsb2	0.008819844	1.781366587	up
Alx4	0.008819974	1.694449902	up
Arl9	0.008819974	1.554883003	up
Ahnak	0.008828232	2.140681744	up
Megf6	0.008828232	1.756297708	up
Chmp2b	0.008828232	1.695015669	up
Tpcn2	0.008828232	2.939031839	up
2700089E24Rik	0.008830302	2.184946537	down
Pcdh10	0.008830302	1.861829281	up
Cyp11b2	0.008853156	1.628949761	up
Gpbp1l1	0.008853156	1.726452112	up
Garnl3	0.008853156	1.759131193	up
LOC383085	0.008853156	1.618908882	up
AI593442	0.008853156	1.956992865	up
Adamts16	0.008856634	1.713822365	up
Lig1	0.008868489	1.777177453	down
Tfpt	0.008868489	1.611630082	down
Hic1	0.008868489	2.263477325	up
Zfp316	0.008897967	2.60804224	up
Lama3	0.008897967	1.753758192	up
Tnfrsf25	0.008897967	1.869071364	up
Cacna2d1	0.008897967	2.822113752	down
Ube2g2	0.008907036	1.697912097	up
LOC234187	0.008931518	1.862615347	up
Doc2b	0.008934243	1.792932868	up
Htatip2	0.008934243	1.737832785	up
Usp42	0.008934243	1.578997731	down
Epb4.1l4a	0.008934243	2.136504173	up
Cd82	0.008934243	1.639499545	up
Rgp1	0.008972336	1.638210297	up
Col7a1	0.008972336	1.732517362	up
Tesc	0.008972336	1.570125699	up
LOC666979	0.008983539	1.747996569	up
BC037594	0.008983539	1.669944406	up
Neurod2	0.008990856	2.692448854	down
Npr3	0.008990856	1.937196493	up

Chrd	0.008990856	1.864286184	up
Hars	0.008999921	1.892925143	up
LOC241293	0.008999921	1.739557147	up
Trim29	0.009006418	2.351221323	up
Lxn	0.009009441	1.571093559	down
Brsk2	0.009010558	1.788912416	down
Acaa2	0.009010558	2.446219921	up
D630016P04Rik	0.009023962	2.351085186	down
Csgalnact2	0.009024526	1.872832775	up
Cobl	0.009025703	1.632187724	up
Cd44	0.009026258	2.136491537	up
2610008E11Rik	0.009029027	1.920101523	down
Sepp1	0.009041655	2.628118753	up
Sf3b5	0.009043137	1.583710909	down
Lox	0.009045014	2.739596367	up
B430320O11Rik	0.009049997	1.826068878	up
Rgnef	0.009049997	1.53875792	up
Pwwp2b	0.00905255	1.522789717	up
A930011E24Rik	0.009054946	1.627388954	up
Stk3	0.009054946	2.009322882	up
5830434K11Rik	0.009054946	1.941124439	up
Prph	0.009054946	1.726447105	up
Rps6ka3	0.009057363	1.576730967	up
Gpnmb	0.009057363	1.590310454	up
Pax1	0.009057363	1.705499768	up
A230065C20Rik	0.009059799	1.517260313	up
Btla	0.009061727	1.748383045	up
Pja2	0.009061727	1.559509635	down
Trappc3	0.009061727	2.250225306	up
6530404N21Rik	0.009061727	2.422698259	down
Dennd5b	0.009076538	1.552022696	up
Cbx5	0.009076851	2.214555979	down
LOC386146	0.009076851	1.50862205	down
LOC234281	0.009125171	1.605422258	up
Col5a1	0.009160657	1.594780684	up
6720453O12Rik	0.009167681	2.122038841	down
Vmn2r42	0.009167681	1.694909096	up
Slc22a7	0.009179139	1.850149989	up
AI317223	0.009181267	2.754652977	up
3930401K13Rik	0.009211071	1.629343033	up
Ap1m2	0.009213029	1.756723166	up
LOC382853	0.009222842	1.703528643	up
4632427C23Rik	0.009232591	1.84664011	up
A730086L23Rik	0.009252652	1.637778044	down
scl0003749.1_1	0.009258654	1.771020532	up
Iqsec2	0.009260654	1.749108076	up
Pla2g4e	0.009274702	1.731672645	up
Dbc1	0.009296128	1.927104592	up

Mesdc2	0.009309726	1.638982177	up
Kcnh1	0.009317075	1.685253739	up
Tectb	0.009317075	1.826779246	up
2900056M20Rik	0.009323944	1.768056512	down
Marveld1	0.009323944	1.905035377	down
Rpap2	0.009350875	1.824938774	up
Krt82	0.009350875	1.685524821	up
Ky	0.009350875	1.556844831	up
Plac1	0.009350875	2.048282147	up
Efcab1	0.009350875	1.954356194	down
Glce	0.00935186	1.682875991	up
Ankrd11	0.00935186	1.77718401	down
L3mbtl3	0.009355946	1.790064216	up
Ccdc46	0.009356299	1.526793838	up
Asap3	0.009356299	1.729180336	up
Fas	0.009356302	1.652994633	up
Fmn2	0.009378376	1.58002758	down
5730521P14Rik	0.009378376	1.823807716	down
Prpf4b	0.009378376	1.873102784	up
A930010I20Rik	0.009378376	1.576213956	up
1700021K14Rik	0.009378376	1.734856129	up
Lgi1	0.009378376	1.720410585	up
Egfl7	0.009378376	2.257963657	up
Dct	0.009381647	5.408703327	up
Aim1	0.009382416	2.137357235	up
AW551984	0.009382416	2.160153389	up
9430047F21Rik	0.009399107	2.200658083	down
Tek	0.009403303	2.240086794	up
Rftn1	0.009403303	1.694047093	up
2310014B11Rik	0.009403303	1.707872987	up
Ssbp3	0.009403303	1.716467619	down
A230069A22Rik	0.009403303	2.012366772	up
Dcamkl1	0.009403303	2.415606022	down
2410187C16Rik	0.009403303	1.793356061	up
Thoc3	0.009423662	1.74524796	up
Csnk1e	0.009423662	1.974310756	down
Stambpl1	0.009439745	1.552924633	up
2610017I09Rik	0.009442161	2.153576136	down
Mthfsd	0.009465211	1.579182625	up
Igf2bp1	0.009466274	4.299771309	down
Car13	0.0094798	1.923994303	up
Polm	0.0094798	1.749252677	up
Ednrb	0.009485351	2.049070835	up
Gstt1	0.009485351	1.692538023	up
MJ-7000-176_2342	0.009506363	1.790103555	up
Lamc1	0.009507799	1.813621044	up
Cks2	0.009509658	1.66986084	up
Bclaf1	0.009511272	1.715369105	down

M6a-pending	0.009511272	1.719253659	up
Chd2	0.009511272	1.681903601	down
Spna1	0.009511272	1.511319161	up
Sgtb	0.009511272	2.044890881	down
Olfr51	0.009511272	1.790260196	up
Polr2h	0.009511272	1.651274562	down
Traf4	0.009511272	1.509787679	down
Zcchc16	0.009511272	1.827062964	up
Slc32a1	0.009511272	1.809121847	down
Tas2r143	0.009511272	1.756335616	up
H2-T18	0.009511272	1.576084495	up
LOC639396	0.009511272	2.553806782	down
Rnf113a1	0.009511272	1.73279798	down
A630040K04Rik	0.009511272	1.715204954	up
A230021I18Rik	0.009533898	1.677199125	up
Wnt3a	0.009533898	1.846368909	up
Rnf168	0.009533898	1.717544675	down
Hrasls	0.009533898	1.888400435	up
Rnf32	0.009533898	1.609242559	up
2310047K21Rik	0.009543013	1.793278933	up
Med18	0.009543013	1.547109008	down
Tmco5	0.009543013	1.612348199	up
Grik1	0.00955644	1.662040234	up
Nfib	0.009564897	2.907538176	down
Grasp	0.009569681	1.795322418	up
6430511F03	0.009575986	1.850068808	down
1500041B16Rik	0.009575986	1.641217709	up
D530030K12Rik	0.009575986	1.790443778	up
LOC329305	0.009575986	1.742141366	up
Smtnl1	0.009575986	1.797136784	up
Ang1	0.009575986	1.589321375	up
9630031F12Rik	0.009575986	1.63048625	up
Sh3pxd2b	0.009575986	1.673878074	up
4930451E10Rik	0.009575986	1.890608788	up
Arhgef15	0.009575986	1.632623673	up
Mtap7d2	0.009584787	1.686928392	up
Stag1	0.009584787	1.589204788	down
Olfr1158	0.009585924	1.75974834	up
Plekha4	0.009585924	2.825376749	up
Osbpl3	0.009585924	1.779070735	up
Ap3b2	0.009585924	1.839215517	down
Itsn2	0.009589402	1.550005555	up
Amdhd1	0.009592187	1.691668153	up
Bean	0.009618859	1.650404096	up
Ptch2	0.009618859	1.617135406	up
2610042F04Rik	0.009618859	1.627019048	up
4933439F18Rik	0.009618859	1.707402825	up
4632428D17Rik	0.009618859	1.684673429	up

Clasp1	0.009618859	1.849764943	down
Rac1	0.009618859	2.171116591	up
Kank4	0.009618859	1.810952306	up
C030014I23Rik	0.0096207	1.833017111	up
Dnahc2	0.00965141	1.718784094	up
Ibtk	0.009675338	1.689213276	down
Zeb2	0.009684069	1.910193563	down
Gls	0.009684069	1.872848392	up
Adamts2	0.00969049	2.20483923	up
Bcl2l1	0.00969049	1.635137916	up
LOC386486	0.00969049	2.146090508	up
Setd1b	0.00969049	1.837556601	down
Snx22	0.009693194	1.624597073	up
C1galt1	0.009693194	1.780945063	up
Stat5a	0.009693194	1.863171816	up
Mfrp	0.009693194	1.697880507	up
Arid4a	0.009699852	3.754585743	down
4933402K11Rik	0.009699852	1.772885919	up
Syn1	0.009699852	1.60662806	up
9530081N05Rik	0.009705481	2.117772341	up
Chrnb2	0.009718569	1.82761991	down
Ncor1	0.009718569	1.59107554	up
Zfp709	0.009718569	1.565183044	up
Ppap2a	0.009724128	1.914561749	up
2610312E17Rik	0.009726942	1.835261941	up
Syngr2	0.009726942	1.786611199	up
Tnxb	0.009726942	1.727104425	up
Col2a1	0.009729158	2.52992034	down
Gm1965	0.009748752	1.606631279	up
A330042I05Rik	0.009748752	1.825091958	up
Tcf12	0.009768348	4.621706009	down
F730047E07Rik	0.009768348	1.615518808	up
Stra13	0.009768348	1.548165321	up
2810409C01Rik	0.009768348	1.822673321	up
Prodh	0.009776405	1.683742285	up
Hip1	0.009776405	1.915647626	down
B430109P06Rik	0.009791596	1.735945463	down
Efcab4a	0.009791596	1.717207909	up
A730048K03Rik	0.009791596	1.536135435	up
Tmem74	0.009791596	1.620414615	up
Actl6b	0.009791596	1.56866312	up
E2f2	0.009791596	1.546877265	down
Ptprm	0.009791596	1.611815929	up
Trak1	0.009791596	1.625074744	down
C1qtnf2	0.009791596	1.971712709	up
Abca9	0.009791596	1.729775071	up
0710001D07Rik	0.009804831	1.632119894	down
5430405G24Rik	0.009808603	1.777567267	down

Il17ra	0.009829191	1.719638467	up
Gamt	0.009842878	1.931936264	up
LOC245350	0.009845682	1.576711178	up
9630028B13Rik	0.009845682	1.894447446	up
4930532M18Rik	0.009847682	2.035574436	up
D830027H13Rik	0.009847682	3.069585562	down
H2-T23	0.00989536	2.005981445	up
0610007P22Rik	0.009906671	1.580251098	up
9130014G24Rik	0.009911442	1.707457423	up
Acad8	0.009915068	1.820392132	up
E330013M12Rik	0.009915068	1.608363867	down
Pbrm1	0.009915068	10.04542637	down
Dhx58	0.009915068	1.88868928	up
4930507C10Rik	0.009941685	1.529292464	up
4931408A02Rik	0.009955286	1.687048078	up
Phf14	0.00996006	7.448523998	down
6030458C11Rik	0.009961322	2.476020098	down
Ssfa2	0.009961322	2.143637896	up
Plscr4	0.009961322	1.84641397	up
Rassf1	0.009961322	1.630946398	up
Map3k11	0.009961322	1.851284146	up
Elf3	0.009961322	1.655043244	up
Abcg5	0.009961322	1.60523212	up
Cacnb1	0.009961322	1.712791205	up
Dusp16	0.009961322	1.527858377	up
Bicc1	0.009961322	1.860623479	up
Rab13	0.009961322	1.957671285	up
Vit	0.009961322	2.304959536	down
Sema3c	0.009967953	2.030423641	down
Ntrk3	0.009967953	2.776690722	down
Alg8	0.009967953	1.792995334	up
Snx21	0.009967953	1.751144052	up
4921507O14Rik	0.009967953	1.731690049	up
Col17a1	0.009967953	1.843631864	up
Hoxb9	0.009967953	6.184360504	down
A730008L03Rik	0.009967953	2.121744871	up
9930033H14Rik	0.009967953	1.52192843	down
LOC638024	0.009967953	1.598380804	up
Qpct	0.009967953	2.19092536	up
Htr2a	0.009967953	1.957232952	up
Ttc5	0.009967953	1.716967344	up
Oxa1l	0.009967953	2.237709045	up
LOC385644	0.009967953	1.925369024	up
Tssc1	0.009968081	1.781689525	up
Egfl8	0.009977075	2.60512495	up
Mcph1	0.009977075	1.624047279	up
Peg10	0.009983914	1.66422832	down
Trps1	0.009986705	3.692302942	down

Tnfrsf10b	0.010004637	1.596958637	up
Grn	0.010004637	1.63796401	up
Man1a2	0.010019645	1.686466217	up
Plcxd3	0.010021418	1.775030851	down
Oplah	0.010022948	1.667140842	up
Psmc5	0.010022948	1.638295174	down
AW551984	0.010022948	1.863375545	up
Irf3	0.010022948	1.528971195	up
Eef1a1	0.010022948	2.054626703	up
Kcna6	0.010035668	2.52366209	up
D9Ertd280e	0.010044813	1.673281074	up
Rc3h2	0.010047354	2.368113995	down
9930031P18Rik	0.010047354	2.466335297	down
D330037H01Rik	0.010047354	1.654175878	up
0610008C08Rik	0.010047354	1.601561904	up
Slc44a1	0.010047354	2.06903863	up
Fv1	0.010047354	1.683701873	up
A230054D04Rik	0.010047354	1.551310659	down
Usp47	0.010059968	1.620498776	down
Syngr1	0.010059968	1.831097484	up
Rpp40	0.010059968	1.588795781	up
4933404K08Rik	0.010059968	1.799144387	up
Gtf3c5	0.010059968	1.67328465	up
Cldn1	0.010059968	2.176027298	up
1810033B17Rik	0.010059968	1.673642397	up
Ctps2	0.010065473	1.531679392	up
Cybrd1	0.010065473	1.770406127	up
B230353J12Rik	0.010065473	1.740118265	up
Ssb	0.010065473	1.570199251	down
Arhgap20	0.010065473	1.94337976	up
Il7r	0.010065473	1.738043547	up
Adprt2	0.010065473	1.704805136	up
Cntrob	0.010065519	1.559865594	up
LOC100045688	0.010065519	1.638296366	up
Suclg2	0.01006832	1.683843017	up
0610040J01Rik	0.010098255	1.941571832	up
Camk2a	0.010098255	1.671045065	up
Bax	0.010098255	2.59913969	up
Dcc	0.010098255	1.855513096	down
Doc2b	0.010100557	1.560938716	up
4930438B07Rik	0.010100557	1.82436502	up
Sfrp5	0.010100557	1.507822037	up
Gstp1	0.010101304	1.720479012	up
LOC240921	0.010104514	1.79729414	up
Olfr1331	0.010104514	1.694071412	up
LOC384313	0.010108046	1.735565305	up
A930014I12Rik	0.010111998	1.630590797	up
Stat4	0.010111998	2.192642212	up

Bdnf	0.010111998	1.702487707	up
Lip1	0.010111998	1.640202284	up
9530064J02	0.010111998	1.9157722	up
Pld5	0.010111998	1.620617271	up
A630012P03Rik	0.010111998	3.847237349	down
Ccdc58	0.010111998	1.656779289	up
scl000981.1_40	0.010112815	2.174271584	up
Mab21l1	0.010112866	1.900460005	down
2310033K02Rik	0.010113706	1.728074551	up
5430435G22Rik	0.010113706	1.874828219	up
LOC383456	0.010124582	1.912583947	up
Btbd11	0.010124582	2.153679848	up
Tacr3	0.010124582	1.636482	up
Fbxl18	0.010124582	1.584769487	down
AK157302	0.010124582	1.736867666	up
2610040L17Rik	0.010124582	1.710104346	down
Vwa2	0.010124582	1.701166034	up
A530051J20Rik	0.010124582	1.544611812	up
Cd200	0.01013366	2.449304104	up
Wipi1	0.01013366	2.193827868	up
Rbpms	0.01013366	2.178504229	up
5830417I10Rik	0.010154501	1.682996512	down
Inhba	0.010167067	2.203859806	up
D630002J18Rik	0.010169013	1.919835687	up
Nkx2-2	0.010178126	2.26326251	down
Nos3	0.01018383	2.211401463	up
Adamts7	0.01018383	1.656754255	up
Upp1	0.01018383	1.545446515	up
Mknk1	0.01018383	1.735313416	up
Padi1	0.01018383	1.597110868	up
Npc1	0.01018383	2.358113527	up
Cebpb	0.01018383	1.678616881	up
MJ-6000-172_3824	0.01018383	1.656733036	up
Txnrd3	0.01018383	1.578746557	up
6330500D04Rik	0.01018383	1.766164661	up
Antxr2	0.01018383	1.697791338	up
Osbpl6	0.01018383	1.717961907	down
Adam4	0.01018383	1.572851539	up
Mink1	0.010184122	1.607851982	up
Tmem79	0.010187298	1.647562146	up
Coq5	0.010196088	1.914298534	up
Baz2a	0.010199621	1.527067423	down
LOC633360	0.010199621	1.744291663	up
4932441K18Rik	0.010199802	1.581308961	up
Plekhg3	0.010199802	1.722677112	up
Cd44	0.010213082	1.831806302	up
Arhgap8	0.010213082	1.601900935	up
A730037C10Rik	0.010213082	1.595314503	up

Arhgef4	0.010213082	1.551455975	down
LOC329240	0.010213082	1.819801927	up
2410016F01Rik	0.010213082	2.466628552	down
Gss	0.010217038	1.690559864	up
Jun	0.010224382	2.801584482	up
Sorcs2	0.010224382	1.928766966	up
scl0002993.1_1671	0.010224382	1.907090306	up
Abat	0.010224382	2.595728159	down
Afg3l1	0.010243389	1.557559371	up
scl0003176.1_43	0.010243389	1.646537304	up
Cntd1	0.010259579	1.743093133	up
Bambi-ps1	0.010266212	1.516471863	up
LOC382153	0.010278828	1.966159701	up
Egfl7	0.010288999	1.650214195	up
B230107K20Rik	0.010289455	1.545329928	up
Ablim2	0.010302333	1.742497087	up
Foxk1	0.010323258	1.6241889	up
LOC229875	0.010323742	1.66113472	up
Ggn	0.01032668	1.521133542	up
A130093I21Rik	0.010338224	1.566944838	up
Eral1	0.010343298	1.503149867	up
0610038D11Rik	0.010343298	1.62931037	up
Fancm	0.010348055	1.65803957	down
Foxq1	0.010362607	1.986995697	up
Gstp1	0.010411408	2.557179689	up
Frmd8	0.010411408	1.641643882	up
Edaradd	0.010411408	1.752348661	up
Rchy1	0.010423307	1.690673351	up
LOC381359	0.010425204	1.837481856	up
Pcdhb6	0.010444157	1.606399894	up
Hif3a	0.010457199	1.68010354	up
Zfp187	0.010457199	1.760539413	up
Ebf2	0.010457199	1.842913866	down
Pparg	0.010457199	1.60936296	up
Espn	0.010457199	1.580123186	up
Camk1g	0.010457199	1.87213552	up
Cnih3	0.010457199	1.655857921	up
Slc30a9	0.010457199	1.819581509	up
LOC100045491	0.010457199	1.745486736	up
LOC381362	0.010457199	1.538586736	up
LOC100041585	0.010457199	2.415282726	down
Clmn	0.010457199	1.59999311	up
Shroom2	0.010457199	1.989614606	up
Nlrp6	0.010457199	1.617817998	up
BC004004	0.010463444	1.615346551	up
Fosl2	0.010464838	2.030889034	up
Slc7a11	0.010464838	1.922748923	up
1700009P03Rik	0.010471516	1.873085976	down

Fam118a	0.010503588	1.677536368	up
Gstm6	0.010515872	1.643243671	up
Whsc1	0.010515872	2.009817362	down
Ednra	0.010515872	2.376912355	up
2610042L04Rik	0.010521354	1.583521843	up
Thnsl2	0.010523095	1.63825047	up
LOC382009	0.010527131	1.940844417	up
9330179O15Rik	0.01054594	1.590577841	up
Dpp4	0.010547425	2.14354825	up
LOC100047090	0.010547425	1.77200067	up
4932442L11Rik	0.01054912	2.323912859	up
Actb	0.010575866	2.425012589	down
Hvcn1	0.010583269	2.098302364	up
Klf10	0.010596232	1.968455076	up
Hbb-b1	0.010596232	2.382575512	down
Tspan8	0.010596232	1.7260077	down
Prss29	0.010598878	1.732155323	up
Ube2t	0.010629043	2.584891558	down
LOC280487	0.010656527	3.227423191	down
Olfr503	0.010656527	1.650408387	up
Cdh19	0.010658056	1.980118632	up
B3galtl	0.010663029	1.861565471	up
Olfr1161	0.010663029	1.579416752	up
Prkar2b	0.010663029	1.91885066	down
Shoc2	0.010665107	1.506416559	up
Tlr5	0.010668595	1.629420519	up
Rab9	0.010671971	1.627312064	up
Kif3c	0.010695607	2.409147978	down
Shcbp1	0.010695607	1.849149823	down
D16Ertd472e	0.010695607	1.583073974	down
C030013C21Rik	0.010700243	1.637208819	up
Gpr27	0.010707911	1.97866106	up
Trak2	0.010715419	1.622221828	up
Hdac4	0.010715419	1.53506124	up
Tnfaip2	0.010715419	1.799211144	up
Elf2	0.010715419	1.779895663	down
Nek3	0.010722333	1.684841752	up
D330001F19Rik	0.010725058	1.605007768	down
Rwdd2	0.010725058	1.694927335	up
Lgals3	0.010732843	2.737843514	down
Neurod6	0.010732843	1.713907242	down
Eya2	0.010755229	1.58567214	up
Hlcs	0.010756576	1.835754395	up
Eif4e1b	0.010760221	1.676146388	up
4631416M11Rik	0.010760221	1.605516553	up
Wipi1	0.010760221	1.80566597	up
Defb36	0.010760221	1.562290192	up
4631424J17Rik	0.010761268	1.834288001	down

Hdc	0.01076162	1.878775954	up
Sgcb	0.010763219	1.523356319	up
Arf4	0.010767346	1.944905996	down
Hmcn1	0.010767346	2.41880703	up
BC049806	0.010775796	1.508741498	down
Nagk	0.010775796	1.683791757	up
Plxnd1	0.01077733	1.647153854	up
Lpgat1	0.01077733	3.97980094	down
Ifit2	0.01077733	1.608265281	up
2310045L10Rik	0.01077733	2.148965597	up
Mef2b	0.01078061	1.606473804	up
Tenc1	0.010784611	1.59923029	down
2610208M17Rik	0.010784611	1.901391029	up
E330031M19Rik	0.010821248	1.600414753	down
A230050P20Rik	0.010821248	1.616741896	up
Sfxn4	0.010821248	1.505086184	down
4732489I21Rik	0.010821248	1.650205255	up
Rabggtb	0.010821248	1.624390602	up
A630029M15Rik	0.010821248	1.617559314	up
Xkrx	0.010821248	1.51066339	up
Si	0.010821248	2.492833853	up
Rps13	0.010821248	1.508597493	down
4022450E19Rik	0.010821248	1.548230887	up
D16Bwg1494e	0.010821248	1.797379851	up
4732458O05Rik	0.010847346	1.851853132	up
Nudt12	0.010860155	1.879082084	up
Ddx26	0.010860155	1.972530484	down
Nsmaf	0.010861693	1.773298979	up
Tmem201	0.010866455	1.515426159	up
Tjp3	0.010879863	1.695411682	up
Kctd12	0.010882232	2.749942064	up
LOC100043609	0.010886484	1.598501802	up
D130092D14Rik	0.010903551	1.723791242	up
Flrt3	0.010903551	1.648629069	down
Pdlim3	0.010939837	2.682033539	up
Rhpn1	0.010960905	1.836043477	up
1700072E05Rik	0.010960905	1.87967062	up
Cp	0.010960905	1.718404055	up
Hoxb5	0.010960905	2.151054144	down
Ube3a	0.010983762	5.17346096	down
Slc7a8	0.010984358	2.080375433	up
6530406M24Rik	0.010993189	1.637717843	up
Mink1	0.010993189	1.634619474	up
Yod1	0.010993709	2.121422529	down
F730003H07Rik	0.011007063	1.864122152	down
Meis1	0.011007063	1.549613714	down
Msc	0.011011572	1.783364654	up
Dph2	0.011011572	1.525639892	down

Kank3	0.011011572	1.58940959	up
Aqp11	0.011011572	1.727133036	up
4930426D05Rik	0.01103376	1.927877069	up
Ptpru	0.01103376	2.411968231	up
5530401J07Rik	0.01103376	1.547336459	up
Mthfs	0.011034815	1.594273329	up
Epb4.1l3	0.011035223	1.673310518	up
Eps8l1	0.011038736	1.747138858	up
Ush1c	0.011042186	1.809464455	up
Frk	0.011056833	1.541083217	up
Bmp1	0.011056833	1.860135555	up
scl0001416.1_21	0.011056833	1.683419824	up
Stoml1	0.011056833	1.722013474	up
Lynx1	0.011056833	1.686983228	up
A630072J24Rik	0.011056833	1.832256794	down
1700003B17Rik	0.011056833	1.528068423	up
Nek6	0.011056833	1.50329876	up
LOC100041388	0.011056833	2.745271683	down
AV249152	0.011056833	1.850089669	up
Sc4mol	0.011056833	1.743438602	down
Slc25a35	0.011056833	1.623518825	up
LOC331752	0.011056833	1.690129757	up
Gm1141	0.011056833	1.73587954	up
Col14a1	0.011059135	1.706919313	up
Dclk3	0.011059135	1.66853416	up
LOC100041504	0.011099011	1.78561306	up
4932416G22Rik	0.011099011	1.541840434	up
A430083B19Rik	0.011099011	1.779715538	up
Tmem63b	0.011099011	2.38227582	down
Rom1	0.011099011	1.533647656	up
Sult5a1	0.011101321	1.642968297	up
Stmn4	0.011101321	1.679844499	up
Dcn	0.011101321	1.504612088	up
Tsg101	0.011101321	2.23314023	down
Mb	0.011101321	1.653795242	up
Ccdc62	0.011151237	1.583951712	up
Arpc1b	0.011152206	3.204498053	up
Dll3	0.011164614	3.92890954	down
Klhdc4	0.01116605	1.662284851	up
Pex13	0.011176942	1.754096985	up
Arhgap27	0.011206553	1.80379498	up
Cmtm7	0.011206553	1.633191705	up
9830143E02Rik	0.011206553	1.969175935	down
Rbbp7	0.01121337	1.541571498	up
Cabin1	0.01121337	1.713338733	up
Adamts2	0.011218472	2.847491264	up
Csrp3	0.011233195	1.636179805	up
4833424O15Rik	0.011265426	1.593027949	up

9430047L24Rik	0.011267471	2.012345552	down
5430426F23Rik	0.011267471	1.530526161	up
4930422I07Rik	0.011268648	1.743861914	up
Ptcd2	0.011283846	1.757236362	up
Asb7	0.011283846	1.58927846	up
9030416H16Rik	0.011288506	1.598853231	down
Tom1l1	0.011288506	1.984600902	up
Tnfaip2	0.011288506	1.765173793	up
Adamtsl2	0.011290228	1.870079875	up
Grm8	0.011290228	1.525563598	up
Lcat	0.011290228	1.563041687	up
Stxbp1	0.011290228	1.580361486	down
Efemp2	0.011290228	1.500731707	up
Slc41a3	0.011290228	1.578166723	up
Olfr310	0.011292169	1.769384861	up
Atxn3	0.011297166	2.043799639	up
2310045K21Rik	0.011297987	2.006486654	down
Rgl3	0.011307555	1.608974099	up
Apoc1	0.011327446	1.570577979	up
Supt16h	0.011337802	1.534345388	up
Ifngr1	0.011343291	1.574216843	up
Nsg2	0.01134892	1.534624696	up
2700008B19Rik	0.01134892	1.766015649	up
Pldn	0.011349508	1.753665328	down
LOC668978	0.011353021	1.736880064	up
A730002K21Rik	0.011353517	1.66293931	up
Dapk2	0.011353517	1.687832594	up
Slc45a2	0.011353517	2.087293148	up
Brd8	0.011353517	1.553807855	up
Ctbp1	0.011372117	1.546590805	up
Tgm1	0.011372117	1.680336237	up
Scrn1	0.011403981	1.710395575	up
Mmp11	0.011403981	2.377553701	up
Tspo	0.011403981	1.640835285	up
Acaa2	0.01141217	2.12840867	up
Lama5	0.011413309	1.59391427	up
Zfp366	0.011413309	1.672422528	up
Smtnl2	0.011423902	3.16843009	up
Alkbh8	0.011423902	3.524726868	down
Asb4	0.011423902	2.067730427	down
BC004728	0.011435959	1.598439455	up
Hist1h2ao	0.011440333	2.653503656	down
LOC100047052	0.01145961	2.033623934	down
Galc	0.011466484	1.791874766	up
Rdh5	0.011479498	1.650363207	up
2610034E13Rik	0.011479498	1.544367313	up
Calr3	0.011492014	1.521449924	up
A930024E05Rik	0.011505215	1.513987064	up

Igf1	0.011505215	2.030432224	up
Tmem119	0.011510411	2.143751383	up
Dusp16	0.011510411	2.082497358	up
LOC385532	0.011510411	1.794663072	up
6430598A04Rik	0.011510411	2.148559332	up
Ptprz1	0.011510411	1.955455542	down
Pa2g4	0.011510411	7.276388645	down
2610507B11Rik	0.011510411	1.598342657	up
BC020535	0.011510411	1.756281138	up
Wdr31	0.011512122	1.515628338	up
Cd300a	0.011542867	1.574785948	up
4930488F09Rik	0.011542867	1.620055795	up
9330120H11Rik	0.011542867	1.512983203	up
Cyp2f2	0.011547794	2.035455942	up
Gcap14	0.011547794	1.821886778	down
Mall	0.011547794	1.700117588	up
Il17rc	0.011550861	1.590351939	up
Cd274	0.011560827	1.644887447	up
Stx3	0.011567494	1.806425929	up
Smox	0.011577917	1.976448059	up
Spata19	0.011583062	1.591976285	up
Cldn15	0.01158365	1.727388144	up
Mcc	0.0115845	1.996290565	up
Bcl2l13	0.011597067	1.887636542	down
Ccdc127	0.011597067	1.529310584	up
2310046K01Rik	0.011597067	1.599696279	up
LOC384384	0.011597067	1.945411801	up
Arhgdig	0.011605384	1.661142945	up
Samm50	0.011617559	1.691869259	up
Hspg2	0.011617559	1.609863281	down
scl0002377.1_1	0.011632521	1.640759945	up
Iqca	0.011632879	1.954819679	up
Cryz	0.011632879	1.898544669	up
Miox	0.011632879	1.738213658	up
Col14a1	0.011637186	2.206558228	up
LOC332788	0.011637186	1.530412436	up
Gatad1	0.011637186	1.754793286	down
Scmh1	0.011637186	1.642729163	down
A330009N23Rik	0.011637186	1.646012902	up
2610020C11Rik	0.011640362	1.654411078	down
Ppfibp2	0.011640362	1.640716553	up
Khsrp	0.01164239	2.094214678	down
1700028D13Rik	0.01164239	1.905315876	up
Itgae	0.011650023	1.50032568	up
Camsap1	0.011650193	6.284976959	down
E430033B07Rik	0.011695062	7.471291542	down
Adh1	0.011696849	2.213549376	up
Mospd1	0.01170554	1.53570044	down

Zcchc11	0.01170554	1.990891933	down
Adam32	0.01170554	1.6147753	up
5031425D22Rik	0.01170554	1.616536617	up
C330050A14Rik	0.01170554	2.041301251	up
A830050C03Rik	0.011714445	1.653364301	up
9330134C04Rik	0.011723714	1.896582842	up
Mtrr	0.01173574	1.65552938	up
Fbxo34	0.011739329	1.988857865	up
Zfp566	0.011740608	1.568594933	down
Abhd14a	0.011749625	1.92773521	up
Herc1	0.011749625	1.824690461	down
4933427G17Rik	0.011761366	1.761082768	up
4932411N02Rik	0.011763196	1.621507645	up
LOC384490	0.011770179	1.563772559	up
Acvr1b	0.011770179	1.574692726	up
Pold4	0.011771417	1.705862045	up
Lyrm1	0.011771417	1.564330816	up
Gprc5b	0.011771417	2.001590252	up
Rfesd	0.01180018	1.502003551	up
Bxdc5	0.011805233	2.002387524	up
9530058B02Rik	0.011808085	1.680402875	up
Tacr3	0.011808085	1.649571657	up
Hebp1	0.011810059	2.555747032	up
Timp2	0.011821125	1.572137356	up
Dusp6	0.011821125	1.823010564	up
Clcn3	0.011823925	5.63412714	down
Vmn2r-ps14	0.011823925	1.542326212	down
4631433D01Rik	0.011823925	1.662487745	up
LOC381211	0.011824336	1.778090715	up
Acan	0.011828069	4.83136034	down
Col25a1	0.011830881	1.686721325	up
1700020N15Rik	0.011830881	1.690655351	up
Atrnl1	0.011830881	3.97765851	down
Cd80	0.011830881	1.749535322	up
1810019D21Rik	0.011839077	1.565328002	up
Csrp1	0.011839273	1.954150319	up
Ttc28	0.011840531	3.188261271	down
Lrrc50	0.011840531	1.704210043	up
Cyb5d2	0.011855832	1.692643166	up
9030611K07Rik	0.011855832	1.762512803	up
Snx2	0.011855832	2.040891409	up
Lrch1	0.011855832	1.595708489	up
Irak4	0.011855832	1.55948031	up
Dhrs4	0.011855832	2.075644732	up
E030010A14Rik	0.011855832	1.676272988	up
Rap2b	0.011856477	1.678804159	up
Vamp4	0.011856477	1.706032634	up
Ch25h	0.011901325	1.531963229	up

2810030E01Rik	0.011919791	1.580499411	down
Fchsd1	0.011919791	1.565979362	up
Sirpa	0.011922749	1.617762208	up
A230057G18Rik	0.011937295	1.646129251	down
D130012P04Rik	0.011946568	1.660106897	up
Tmem204	0.011946568	1.852750659	up
Nol5	0.011947446	1.735115767	down
Osbpl1a	0.011951336	1.746912599	up
Gnai1	0.011951336	1.896371126	down
Acot2	0.011951336	1.769756436	up
Lphn3	0.011957283	1.501995325	up
Lhfp	0.011957283	2.397145271	up
Fryl	0.011957283	1.656945348	down
Cxcl12	0.011968267	2.090909004	up
B2m	0.011969728	1.702445984	up
Igf2bp1	0.011969728	5.167349815	down
9630015D15Rik	0.011970542	1.786219358	up
Trp63	0.011980224	3.432834864	up
Tspan13	0.012004233	2.623078585	down
3830406C13Rik	0.012004233	1.763822675	up
Cpz	0.012004233	1.823085785	up
Gpm6a	0.012004233	1.517501831	up
Mcm3ap	0.012004233	1.575897813	up
1500035H01Rik	0.012020534	1.806617737	up
Scin	0.012042096	3.04027009	down
Rfx4	0.012042096	1.924811006	down
Hbegf	0.012042096	2.184522867	up
9530048O09Rik	0.012042096	1.754866004	up
Flot2	0.012042096	1.732309699	up
Ahdc1	0.012042096	1.549701452	down
Cldn9	0.012042096	1.798755169	up
3110047P20Rik	0.012042096	1.61097467	up
Hdac8	0.012048183	2.034495354	up
Bace2	0.012048183	1.835004568	up
D030069G17Rik	0.012052483	1.678161025	down
Ubxn8	0.012054345	1.541211724	up
Slco1a5	0.012054752	1.770416856	up
Pigo	0.012054772	1.686749935	up
Phox2b	0.012066298	1.636369348	up
scl0003723.1_3	0.012080154	1.731036305	up
Srgap3	0.012080154	1.577518106	up
Col8a2	0.012080154	1.783504725	up
Svep1	0.012080154	1.949231148	up
AW146020	0.012080154	1.638059497	down
Zmym1	0.012080154	1.515415788	up
1810043G02Rik	0.012080154	1.733139515	up
Glt8d2	0.012092791	1.522481918	up
Hspb3	0.012104414	1.599420905	up

LOC386285	0.012106214	1.731864572	up
Gab1	0.012111018	1.867735624	up
Ela2	0.012111018	1.618599415	up
Lmna	0.012111018	1.75941813	up
Vgll2	0.012111018	1.748288512	up
Casp8	0.012111018	1.708166122	up
Tlcd2	0.012112362	2.037941217	up
Zfp50	0.012118196	1.973639727	up
Rasa1	0.012118196	1.679017305	down
Igsf3	0.012118196	2.070895195	down
Ncapg	0.012136094	2.25593853	down
Chst11	0.012142859	1.832973957	down
H2afj	0.012154991	1.823526502	down
Suz12	0.012173476	1.758094192	up
Thsd7a	0.012173476	1.778657198	down
Tdpoz2	0.012173476	1.694103122	up
H2-M3	0.012173476	1.616079927	up
Tspan17	0.012175052	1.734998941	up
Card10	0.012175052	1.886260033	up
Snx19	0.012175052	1.599865317	up
Col2a1	0.012175052	2.746902704	down
Rreb1	0.012176498	1.613272786	up
Rasgrf1	0.012176498	1.806313515	up
Egfr	0.012176498	1.74305439	up
Ascl2	0.012195625	1.572358847	up
B430005K18Rik	0.012217346	1.512902379	up
1110031I02Rik	0.012217346	1.559653163	up
Rdx	0.012217346	2.672815085	down
Fusip1	0.012217346	1.528244495	up
scl0001849.1_2273	0.012217346	1.745334864	down
9430095K15Rik	0.01222843	1.762827516	down
Pxt1	0.012228584	1.694844842	up
Gmcl1	0.012232078	1.608548164	down
Gstm6	0.01223862	1.689009309	up
Acss2	0.01223862	1.701149583	up
Tacr1	0.012245439	1.642027021	up
Trim34	0.012245439	1.819759131	up
Lypd6	0.012245439	1.614569068	up
A830007N09Rik	0.012245439	1.547014117	up
H2afj	0.012245439	1.516836047	down
Msi2h	0.012245439	1.569906712	down
BC016495	0.012245439	1.574391484	up
2810409K11Rik	0.012245439	1.585009575	up
LOC100048616	0.012257522	2.466482639	down
E430021P16Rik	0.012264363	1.58927834	up
Stk3	0.012277322	2.437416554	up
Ctxn1	0.012277322	1.515300751	down
2010007L08Rik	0.012282359	1.555556417	up

Moxd1	0.012315157	2.676060677	up
B930059J09Rik	0.012326201	1.590711951	up
D030016E14Rik	0.012326201	1.598960757	up
A230107C01Rik	0.012326201	1.639726877	up
Metrn	0.012326201	2.639432669	up
Tcof1	0.012326201	5.537293911	down
HPBRII-4	0.012326201	1.589234114	up
Adat1	0.012333787	1.708295822	up
LOC234582	0.012370657	1.859015703	up
Faah	0.012381743	1.539129734	up
Zcchc12	0.012381743	1.586298585	up
A730054J21Rik	0.01239005	1.878707051	down
Ube2o	0.01239005	1.56218338	down
Ric3	0.01239005	1.631143808	up
Uso1	0.01239005	1.944169879	up
Nab1	0.012399819	1.925327063	up
Sox7	0.012399819	1.825410008	up
Tgfbr2	0.012404503	1.528139591	up
Nos3	0.012404503	1.930089355	up
Kars	0.012404503	1.650103092	up
9626965_214_rc	0.012404503	1.670157194	up
Sema3b	0.012404503	1.55468154	up
5930403N24Rik	0.012404503	1.611163139	up
Vstm2b	0.012404503	1.874649525	up
LOC100047856	0.012406936	2.004663229	down
A130094L10Rik	0.012411513	1.726833344	up
Efcab2	0.01241444	1.510913014	up
Ift140	0.012429806	1.821974278	up
Akap8	0.012429806	2.049084425	down
Sesn1	0.012429806	1.625318408	up
Olfr1198	0.012429806	1.854960322	up
6720469N11Rik	0.012429806	1.77593267	up
Col23a1	0.012429806	2.248467445	up
Ddc	0.012429806	1.98083055	up
Dgkb	0.012429806	1.706154347	up
A230067E15Rik	0.012429806	1.908667803	up
Tgif1	0.012434861	2.285291433	up
Gstp2	0.012456207	1.599606276	up
Gpr27	0.012456207	1.926563382	up
5330431K02Rik	0.012456207	1.608683229	down
5330431N19Rik	0.012456207	1.510100365	up
Nos3	0.012456207	2.049824476	up
B130024G19Rik	0.012456207	1.804042935	up
Cmya1	0.012456207	1.798689008	up
Lifr	0.012458962	1.600601077	up
Slmo1	0.012469377	1.660951376	up
Itgb6	0.012495746	1.526900768	up
Tbc1d10a	0.012500916	1.637268424	up

2410012H22Rik	0.012500916	1.506266356	up
Zfp26	0.012500916	1.586893439	up
A530064L23Rik	0.012505763	1.781459928	down
Capg	0.012505763	1.580980301	up
D3Ertd300e	0.012516508	1.958466172	up
5730409N16Rik	0.012526029	1.678104401	up
Tle3	0.012539485	1.613103151	down
Clock	0.012541889	1.684468985	up
Ovol2	0.0125477	1.971618772	up
Hmgcs2	0.012549467	1.83754611	up
Bik	0.012559793	1.895194888	up
Stx3	0.012564512	1.710233808	up
E430002G05Rik	0.012564512	1.923956871	up
Arrdc4	0.012564512	1.847822547	up
Spag4l	0.012567148	1.828890324	up
Ryr3	0.012567453	1.513333321	up
Mapk13	0.012567453	2.042782068	up
Col4a1	0.012567453	2.682374954	up
Mogat2	0.012570755	1.589121342	up
Dnajc10	0.012570755	1.686121702	up
B130064M22Rik	0.012570755	1.721751452	up
Lrrk2	0.012586059	1.682609081	up
Tes	0.012587901	1.577889919	up
Cd247	0.012605749	1.833793163	up
Rpl4	0.012608816	1.899412274	down
Rnf25	0.012617152	1.89558363	up
Gabra3	0.012658064	1.783182979	up
LOC383077	0.01266209	1.519384742	down
Aptx	0.01266209	1.746626854	up
Rbm18	0.01266209	2.32854414	up
Tmprss5	0.01266209	1.838545561	up
Rsf1	0.012672777	2.011774778	down
Zfp791	0.012673094	1.601089835	up
1700037C18Rik	0.012673094	1.587375403	up
Wdr62	0.012673094	1.502382755	up
9830134K01Rik	0.012673094	1.531498432	down
Manba	0.012673094	1.667890787	up
Atp5s	0.012679826	1.554974794	up
Ankrd22	0.012679826	1.674831152	up
Gzmn	0.012695862	1.816921473	up
Btaf1	0.012695862	2.073349714	down
Etl4	0.012695862	1.922305465	up
Tmem183a	0.012695862	1.510905027	down
Trcg1	0.012695862	1.997215509	up
scl0002657.1_5	0.012695862	1.909353852	up
Gstcd	0.012695862	1.739566803	up
Zcchc18	0.012695862	1.741515279	up
Matk	0.012695862	1.540915251	up

LOC100047659	0.012708938	1.703507543	up
5830415F09Rik	0.012708938	1.622027755	up
2810406K13Rik	0.012709986	2.14847827	down
1300018J18Rik	0.012722744	1.787652731	up
Optn	0.012722744	1.730549336	up
Gas7	0.012722744	1.828306913	up
1700113I22Rik	0.012722744	1.693020344	up
Gpd2	0.012722744	1.948267102	down
LOC385681	0.012722744	1.618152261	up
Rffl	0.01272374	1.875655532	up
Rprm	0.012748334	1.770799041	up
Cdc2l6	0.012748334	1.851906538	up
Tpm2	0.012748334	1.922654033	up
scl0002275.1_1	0.012777639	1.519159555	up
Tmem201	0.012789974	1.509128809	up
9530096D07Rik	0.012789974	1.645857692	up
D330038K10Rik	0.012792939	1.648094177	up
Arhgap12	0.012792939	1.598216772	up
Slc9a2	0.012792939	1.539561272	up
Col4a2	0.012792939	1.961358666	up
D330037A04Rik	0.012810879	1.506274223	up
Tle2	0.012810879	2.246054649	up
Chd9	0.012811218	1.541359901	down
Zbp1	0.012811218	1.652341247	up
Olfr638	0.01282654	1.841046929	up
2010205O06Rik	0.01282654	1.603883028	down
1700023B23Rik	0.01282654	1.738013268	up
Rtkn2	0.01282654	1.669357896	up
Myo1c	0.01282654	1.879260302	up
E430021A19Rik	0.01282654	1.5871737	up
2700090O03Rik	0.01282654	4.646639347	down
C130033H03Rik	0.012831846	3.533616543	down
C1qtnf6	0.012831846	1.556827784	up
LOC240312	0.012831846	1.966213822	up
BC024659	0.012832207	1.630675435	up
Slc12a1	0.012832207	1.738138199	up
scl0001379.1_70	0.012832207	1.690096617	up
Rab43	0.012832207	1.514781714	up
Klhl31	0.012832207	1.611522913	up
Palld	0.012840713	1.622717738	up
2610524G07Rik	0.012840713	1.849295616	up
Armcx6	0.012842964	1.744770885	up
Serpine2	0.012848096	1.963433266	up
Dtl	0.012848096	1.59987545	up
6430526J12Rik	0.012849328	1.554057241	up
Osbpl10	0.012850302	1.558575511	up
Snhg11	0.01285346	1.722093344	up
4933412E12Rik	0.012857186	1.580498338	up

Adam24	0.012857186	1.821695924	up
Purb	0.012857186	1.840871215	down
Fxyd5	0.012857186	2.069130659	up
Trappc3	0.012857186	2.624509573	up
Zfx	0.012857186	1.662116885	up
Cycs	0.012857186	1.542874456	down
Olfml3	0.012857186	2.854255676	up
5730593H20Rik	0.012868495	1.561411738	up
Pipox	0.012868495	1.723615646	up
Lrrc3	0.012883328	1.509327769	up
Asl	0.01288882	1.837278009	up
Traf1	0.01288882	1.561558366	up
Col17a1	0.012892193	2.151578903	up
Sos1	0.012893601	1.515380025	up
Tbx22	0.012895339	1.950731993	up
Gng2	0.012908777	1.860420585	up
mKIAA0515	0.012908777	1.528864622	up
Wdr13	0.012908777	2.114797115	up
Tmem49	0.012908777	1.716255188	up
Tnnc2	0.012908777	1.70280242	up
Nrbf2	0.012908777	1.756625056	up
Lix1	0.012908777	1.604478955	up
E330020K23Rik	0.012908777	1.519379139	down
Klhl5	0.01292166	1.647059679	down
Senp7	0.01292166	1.749804497	down
Gria2	0.012924749	3.888768911	down
Gpx8	0.012939198	1.822176814	up
Prei4	0.012959784	1.919138789	up
Ddhd2	0.012959784	1.815763116	up
Cog7	0.012980813	1.605686307	up
Megf11	0.012981579	1.626389742	up
Capg	0.012982037	2.307087421	up
Cxcl13	0.012996545	1.561014772	up
Senp5	0.013002073	1.610194445	up
2610001E17Rik	0.013002073	1.531848192	up
Ppp1r13l	0.013004345	1.629480481	up
Smyd2	0.013011406	2.191441298	up
H2-Q5	0.013017764	1.760607481	up
Erbb2	0.013017764	2.000452042	up
Ddc8	0.013030097	1.551798344	up
Homer1	0.013043122	1.878014684	up
Sort1	0.013048375	1.571092129	up
Omp	0.013052785	1.676357746	up
Usp12	0.013065291	1.800607562	up
Myh8	0.013082539	1.666018248	up
Mapre2	0.013082539	1.696675301	down
Mobkl2c	0.013082539	1.58933425	up
1700019N12Rik	0.013083887	2.632347584	up

Stx1a	0.013083887	1.617613077	up
Mef2c	0.013083887	1.849823237	down
Txnip	0.013083887	2.389566183	up
Gpr155	0.013084268	1.540424824	up
Slco3a1	0.013098188	1.675041556	up
Ret	0.013098188	1.752171278	up
Gm2a	0.013099621	2.049192429	up
Piwil1	0.013105671	1.564337134	up
E330027P06Rik	0.013114644	1.581228972	down
EG545253	0.013133048	1.927534342	up
4930579E17Rik	0.013141112	1.569415569	up
1810049N02Rik	0.01314389	1.941341043	up
Nudt10	0.013150268	1.65554738	down
Dbndd2	0.013150268	1.703242064	up
Wnt6	0.013176431	2.33391428	up
Tmem17	0.01317995	1.55810988	up
C130047K18Rik	0.01319913	1.60621047	up
Crat	0.01319913	1.761101842	up
LOC100043798	0.01319913	1.667848706	up
C330013E15Rik	0.01319913	1.556067944	up
Ccbp2	0.01319913	1.657898426	up
3632413B07Rik	0.01321679	1.513736486	down
Accn2	0.013218284	1.881206751	down
D130007C19Rik	0.013218468	1.986842871	down
Rassf1	0.013218468	2.214915991	up
LOC380741	0.013218468	1.574386716	up
Il15	0.013218468	1.812394142	up
4930429A08Rik	0.013234877	1.798853636	up
Ttk	0.013237063	2.500431538	down
Arhgef16	0.013237063	1.68380928	up
Trim16	0.013246129	1.743749976	up
Zmat4	0.013246129	1.88993454	up
Itm2b	0.013246129	1.63623178	up
Neurog1	0.013246129	1.684467912	up
scl0003949.1_31	0.013246129	1.526632071	up
Ly6g6d	0.013246129	1.887906909	up
Erbb2ip	0.013264354	2.249682903	up
Pfkp	0.013264354	2.039280653	up
Pnck	0.013266735	1.708408237	up
Rad23b	0.013269431	1.56151259	down
4732488M06Rik	0.013269613	1.664440393	up
Rapgef6	0.013269905	1.677681565	up
Olfr665	0.013269905	1.753969193	up
Mbc2	0.013269905	1.871707082	up
LOC381140	0.013269905	1.831867099	up
Gabarapl1	0.013269905	2.037134409	up
E130112L23Rik	0.013280982	1.592687845	up
Otog	0.01329397	2.109349489	up

2610204M08Rik	0.013299095	2.319484711	up
Arc	0.013309997	1.530025601	up
Ina	0.013311707	2.131155491	down
Ncam1	0.013311707	3.885772705	down
Col6a2	0.013322825	1.604469419	up
Bcr	0.013346478	2.248598814	down
Rspo3	0.013369494	1.756639481	up
Lypd6	0.013379875	1.807082057	up
Dusp6	0.013397329	1.53362	up
Slc13a3	0.013397329	1.656684756	up
4921513J16Rik	0.013401387	1.581577659	up
Lrrc7	0.013402397	1.692220926	up
BC031781	0.01341891	1.589268327	down
Nedd1	0.013449693	1.841316819	down
LOC382237	0.013483062	1.889789343	down
LOC100044862	0.013483062	2.049538374	down
Hvcn1	0.013483062	1.953550935	up
Fgf7	0.013487279	1.610567689	up
LOC674960	0.013489854	2.695344448	down
4833413E03Rik	0.013491326	1.625584841	up
Svil	0.013491326	1.920827508	up
2410091C18Rik	0.013491326	1.747476816	up
1810035L17Rik	0.01350009	1.741219163	down
Usp33	0.013511907	2.246424437	down
scl0002368.1_75	0.013513994	2.23694396	up
Ski	0.013518501	1.679907203	down
Tmem16j	0.013532957	1.684244871	up
Magee2	0.013533409	1.83665514	up
Ercc2	0.013533409	1.784334898	up
Kng1	0.013533409	1.873396277	up
Col27a1	0.013539515	1.62655139	down
Klhdc5	0.013544205	1.913423777	up
Cpt1c	0.013572304	1.640739441	up
Dctn1	0.013575318	1.785241962	down
G430055L02Rik	0.013578988	1.522780657	up
Gipc1	0.013585857	1.569516063	up
Gad1	0.013609231	3.400148869	down
Igfbp4	0.013611255	1.788637281	up
E230013M07Rik	0.013611255	1.680230022	up
Pacsin1	0.013611255	1.596661806	up
Gm382	0.013611255	1.656086922	up
Clns1a	0.013611255	1.644989371	up
Pdgfra	0.013611255	1.561200857	up
Dom3z	0.013616579	1.575715065	up
Npat	0.013622694	2.451817513	down
Hey2	0.013622694	1.967078209	up
Slc6a15	0.013626277	2.112127066	up
Sntb1	0.013626277	1.548276424	up

Plcb2	0.013626277	1.627833486	up
C130073O12Rik	0.013626277	2.107435465	down
Mbd6	0.013634127	1.618043661	up
LOC381019	0.013634127	1.809211135	up
Asah2	0.013634127	1.868495584	up
Ift57	0.013634127	2.54534483	down
Tsen34	0.013634127	1.633738399	up
2810047L02Rik	0.013634127	1.50482142	down
1810011O10Rik	0.013634127	1.522880793	up
Sult5a1	0.013634127	1.795954108	up
Tead4	0.013634127	1.55796504	up
Fcgrt	0.013634127	1.703067899	up
Loxl1	0.013634127	1.638782024	up
Rnpepl1	0.013634127	1.562986255	up
Ahnak2	0.013634127	1.829839945	up
1700001L05Rik	0.013651146	1.602116227	up
Txndc9	0.013651146	1.561188817	up
Glrx5	0.013654993	1.620049	down
1110032E23Rik	0.013654993	1.980955362	up
Hs3st3b1	0.013654993	1.7552495	down
Col25a1	0.013654993	1.512342572	up
Frmd5	0.01365511	1.656143069	up
Tcea2	0.013658457	1.66322422	up
Rnf19a	0.013658782	1.513768196	up
E330031I04Rik	0.013658782	1.585134029	up
Msn	0.013665783	1.552477598	up
Rab11fip5	0.013665783	1.571208239	up
Arl4c	0.013665783	1.891957521	down
1600021P15Rik	0.013667509	1.7874192	up
OTTMUSG00000020946	0.013667509	1.590955973	up
6030446N20Rik	0.01366932	1.664617658	up
2810436B12Rik	0.013671587	1.894216299	down
Macrod1	0.013682196	1.719940782	up
Nap1l1	0.013687552	1.707957149	up
Birc2	0.013707012	1.747507811	up
Foxo1	0.013707012	2.203903914	up
9830144J08Rik	0.013707012	1.803768039	up
Syp	0.013726926	1.501088858	down
2700024D06Rik	0.013726926	1.62184	up
Acsl5	0.013729753	1.600696802	up
Sstr2	0.013730879	1.86632812	down
LOC100048616	0.013791034	1.855272889	down
Mad2l1bp	0.013791034	1.966350675	up
Zfp36	0.013797794	1.763923407	up
Repin1	0.013801619	1.642057657	up
B230387C07Rik	0.013803557	1.979465127	down
B4galt6	0.01380568	1.585220337	up
Pdlim2	0.01380568	1.722751617	up

Dusp28	0.013809233	1.530064225	up
4930544G21Rik	0.013809233	1.634804606	up
Upf1	0.013820853	1.605972171	down
Lcat	0.013834346	1.50973928	up
Pafah1b1	0.013835111	1.863887906	down
Mcm7	0.013835393	2.914779425	down
Abp1	0.013835393	1.619427919	up
2410170E21Rik	0.013835393	1.547989368	up
Eftud1	0.013850171	1.511290073	up
Rsrc2	0.013868733	1.75848186	down
Heyl	0.013874807	2.174468756	up
Agpat2	0.013884665	1.629495025	up
Adh5	0.013902674	1.949717641	down
D230007K08Rik	0.013913746	1.551835418	up
Anxa6	0.013920775	1.625330448	up
1110003E08Rik	0.013923276	1.524046063	up
Pcdhb8	0.013923276	1.683185697	up
Tspo	0.013923276	1.532778502	up
Syngr2	0.013923276	1.573250413	up
Usp10	0.013925021	2.52157855	down
Fbxl21	0.013928648	1.700338483	up
Ercc5	0.013952961	1.576063037	down
Hnrpa1	0.013956532	1.902873397	up
P2rx4	0.013969489	2.36952281	up
Angpt1	0.013969489	2.204510689	up
Grhl1	0.013969522	1.689110994	up
Pno1	0.013969522	1.926160812	down
Pum2	0.013969522	1.527429819	down
Ybx2	0.013969522	1.764156342	up
Det1	0.013983971	1.675942421	up
Anapc11	0.013997621	1.650258422	up
Gimap6	0.014000086	1.667111635	up
D930020N02Rik	0.014000086	1.678035498	up
Tbxa2r	0.014000086	1.663612247	up
Ctsw	0.014000086	1.725126505	up
Setd6	0.014000086	1.6902951	up
Phka2	0.014000086	1.793971777	up
1810062G17Rik	0.01400717	1.560486317	up
Tmtc2	0.014017923	1.687301159	up
6330416G13Rik	0.014028094	1.870222926	up
Nisch	0.014028094	2.552846432	down
Adam23	0.014048461	2.003586531	up
Dpysl4	0.014058662	1.761554241	down
4930584N22Rik	0.014058662	1.702664852	up
Eya4	0.014064595	1.789567709	up
Lce3c	0.014064595	1.551779747	up
Mfap4	0.014064733	2.381771803	up
D030020M24Rik	0.014064733	1.570013762	up

LOC383099	0.014077373	1.788389921	down
LOC380923	0.014086179	1.583407164	up
AI875142	0.014086179	1.770733833	up
Paox	0.014097165	1.672313929	up
Add3	0.014109118	2.550353766	up
Madd	0.014110732	1.632431388	up
LOC382421	0.014124274	1.661821961	up
LOC382102	0.01412527	1.555842519	up
2810474O19Rik	0.01412527	4.609839916	down
LOC383092	0.01412527	1.572983861	up
LOC277837	0.01412527	1.813723922	down
C730029A08Rik	0.01412527	1.71424365	up
Mapk10	0.01412527	2.018272877	down
Eif2s2	0.01412527	1.508484244	up
Zfp703	0.01415264	1.685684443	up
Wdr31	0.01415264	1.564507008	up
Scml4	0.01415264	1.782431245	up
2900011L18Rik	0.01415264	1.886803865	down
1810064L21Rik	0.014164309	2.083154202	up
Plekha4	0.014164309	1.899433494	up
Pcsk9	0.014164309	1.775391221	up
Myrip	0.014177469	1.641140223	up
Olfr166	0.0141776	1.747463226	up
B3galt1	0.014201336	2.86161685	down
Prpf31	0.014210763	1.795861125	up
Dgka	0.014212452	2.297182322	up
Cyhr1	0.014218831	1.716747403	up
Nradd	0.014218831	1.98875165	up
Auh	0.014218831	1.553457499	up
Plxnd1	0.014218872	2.074588776	up
Tsc22d3	0.014218872	1.615373969	up
Htr3a	0.014220302	2.059222221	up
Alcam	0.014223813	3.924258232	down
Casp7	0.014224262	2.180449009	up
Synj2	0.014240557	1.641053677	up
Sergef	0.014243893	1.677181482	up
Tmem62	0.014243893	1.811025262	up
C330006H24Rik	0.014245119	1.736635566	up
Tmed4	0.014245119	1.775812268	up
Ica1	0.014245119	1.667012453	up
Egln1	0.014245119	1.546107292	up
Crls1	0.014245119	1.510665774	up
2810403A07Rik	0.014245119	1.660168648	down
1700041B20Rik	0.014253892	1.544244885	up
Tnmd	0.014255907	1.557863236	up
Crry	0.014257797	1.596698523	up
Igfbp3	0.01428549	2.336178064	up
Pfdn2	0.014290274	1.936927915	up

Chrna4	0.014300482	1.716660023	down
LOC433886	0.014306307	1.533434153	up
Itch	0.014318481	1.681077004	down
2310007H09Rik	0.014330474	1.752916455	up
Atg4c	0.014330474	1.544730544	up
Vrk2	0.014330474	1.834310412	up
Elmo1	0.014367481	1.722578287	up
Mtcp1	0.014367481	1.784079671	up
4832408C21Rik	0.014367481	1.642045617	down
Saal1	0.014367481	1.638142943	up
Olfr267	0.014370917	1.562190294	up
Snx19	0.014370917	1.590021849	up
Gga2	0.014374672	2.126532793	up
Rnf19a	0.014401651	1.563728333	up
Mbtps2	0.014413244	1.532248855	up
Ap4m1	0.014413244	1.759465694	up
Mapkapk3	0.014413244	1.81407094	up
Stx8	0.014414357	1.503189087	up
scl000528.1_16	0.014420311	1.554077268	up
Dcbld2	0.014420311	1.717579365	up
LOC241262	0.014420311	1.630625606	up
Gm960	0.014428946	1.647107601	up
2900052M09Rik	0.014433549	1.657208085	up
4833436C18Rik	0.014448089	1.631286502	up
2310058D17Rik	0.014455225	1.558657765	up
Mllt3	0.014481396	1.572801113	down
Il23a	0.014484429	1.766286612	up
2010204N08Rik	0.014493306	1.709066033	up
4931408A02Rik	0.014494159	1.554969788	up
Pkp3	0.014494159	1.582759023	up
Ptar1	0.014494159	1.687353253	up
Antxr2	0.014494159	1.591258168	up
A830007F21Rik	0.014494159	1.584760547	up
LOC623466	0.014512978	1.620241165	up
Lingo2	0.014517918	1.716675162	up
5430405C01Rik	0.014530717	1.673426867	up
Slc38a2	0.014549396	1.7501899	up
1700025G04Rik	0.014550493	2.360963821	down
2210419D22Rik	0.014564308	1.819339275	up
Pttg1ip	0.014580193	2.390427828	up
Mtap1b	0.014585038	2.097805023	down
Gjb5	0.014594515	1.674165487	up
Ccdc114	0.014621565	1.693051457	up
Atp6v1c2	0.014628421	1.887662649	up
LOC100046746	0.014628812	3.623121738	down
Sphk1	0.014646932	2.023423195	up
Clk4	0.014646932	1.539265275	down
Pip5k1c	0.014647911	1.713070989	up

Pias2	0.014650574	2.012918711	up
1700027A23Rik	0.014650574	1.573133707	up
Fgd5	0.014650574	1.583142996	up
Col2a1	0.014667247	2.265813828	down
scl0003020.1_1	0.014667247	1.68411541	up
Megf9	0.014682405	1.756153822	down
scl0001544.1_68	0.014688253	1.619564295	up
Olfr352	0.014724299	1.65255475	up
2010311D03Rik	0.014730224	1.713189364	up
Tgfb2	0.014732455	4.134972572	down
4432404P07Rik	0.014732455	2.896472216	down
Rasl11b	0.014732455	2.123251438	up
Igf1	0.014738426	2.360509396	up
AI507611	0.014739122	1.624577761	up
Arl2bp	0.014741469	1.97984755	up
Peli3	0.014745706	1.644215226	up
Olfr270	0.014745706	1.669532299	up
Prrg1	0.014750493	1.857349992	up
Rffl	0.014764269	1.621606946	up
mKIAA1686	0.014772357	1.645732403	up
4632427K15Rik	0.014772357	1.523310423	down
Gtf2h1	0.014772357	1.76902163	up
Sec22l3	0.014772357	2.232342005	down
Sumo3	0.014779127	2.12762022	up
B130017M24Rik	0.014801525	1.729433656	up
Vim	0.014811567	1.553122878	up
Prrx2	0.014824606	1.711831927	up
1110008J03Rik	0.014824606	1.766311765	up
Zfp111	0.014831599	1.600939274	up
4631416L12Rik	0.014831599	2.401184797	up
B430110C06Rik	0.014833784	1.655187964	up
Nsmaf	0.014833784	2.10326004	up
4833421E05Rik	0.014834432	1.561596274	up
LOC224532	0.014842451	2.562713623	up
Larp-pending	0.014843697	1.591766357	up
Bbs12	0.014863434	1.653618217	up
Rad51l3	0.014863434	1.548033953	up
Bbx	0.014863434	3.876441717	down
LOC386112	0.014863434	4.158618927	down
LOC627626	0.014863434	1.516692996	up
Foxred1	0.014863434	1.602311254	up
Zfp622	0.014875645	1.717239976	up
Lyzs	0.014875645	2.604290247	down
Ptprv	0.014875645	1.889452219	up
Fnbp1	0.014880369	1.514274001	up
1110034A24Rik	0.014903376	2.949354172	down
Gls	0.01490956	1.942672491	up
H47	0.014909916	1.649338722	down

Daglb	0.014912994	1.75685072	up
Rnf213	0.014913261	1.866276145	up
2010015J01Rik	0.014913261	1.633465767	up
Ptgis	0.014913261	1.936197639	up
2610524H06Rik	0.014915748	1.691795707	up
BC014795	0.014926729	1.511735797	up
Olfr1437	0.014926729	1.829902172	up
As3mt	0.014926729	1.978042603	up
Arhgap20	0.014926729	1.717409968	up
2700060E02Rik	0.014926729	1.762716532	up
Fn3k	0.014939282	1.660053372	up
Gabrp	0.014939282	1.616749406	up
D230040E23	0.014942837	1.51877892	up
Inadl	0.014942837	1.922322393	up
Thnsl2	0.014948658	1.639492273	up
2210404O07Rik	0.014964229	1.598262668	up
Fgf1	0.014990621	1.616851687	up
Tapbp	0.014990621	1.529372096	up
Gm784	0.014990621	1.597453356	up
Slc35a3	0.014990621	1.524765372	up
A730089E01Rik	0.014990621	1.806908608	down
Nif3l1	0.014990621	1.509633184	up
Prr12	0.014990621	1.5633111	up
Tle2	0.015003369	2.098104954	up
Sdf2	0.01504455	1.863868475	up
Stap2	0.015047802	1.619958282	up
1190002H23Rik	0.015052724	1.538797021	up
Sorl1	0.015059365	1.710558295	down
LOC386186	0.015072259	1.60037303	up
Pax1	0.015082571	1.610585928	up
Rpl21	0.015082571	1.556022644	up
Tgfb3	0.015102916	2.167383194	up
Ccdc39	0.015135118	1.613043547	up
Rnf130	0.015140302	1.514383078	up
Myl6	0.015140302	1.684257507	up
Fermt1	0.015154142	2.125511169	up
Morn3	0.01515819	1.78691411	up
Ckmt1	0.015176937	1.796265841	up
Slc34a1	0.015176937	1.597052932	up
Pclo	0.015176937	1.637142658	up
Gsdmdc1	0.015176937	1.948074579	up
2610001J05Rik	0.015176937	1.686969757	up
BC024139	0.015182142	2.274022341	down
Cdh15	0.015182142	1.886306167	up
Tcfap2c	0.015184633	2.369262219	up
LOC100047837	0.015190722	1.855508804	down
Cdh3	0.015194433	1.588147759	up
Iah1	0.015194433	1.873577833	up

EG545216	0.015194433	6.050276279	down
LOC232974	0.01522027	1.582114935	up
Ddit3	0.01522027	2.218580246	up
Dhx58	0.01522027	1.726668596	up
Taf7	0.015235992	1.597987771	up
Dnahc9	0.015256922	1.635884285	up
Faim	0.015256922	1.587582946	down
Edg3	0.015256922	1.722615957	up
LOC668047	0.015256922	2.542768002	down
Hoxd13	0.015256922	2.149003744	up
Dagla	0.015261078	1.714764834	up
Pld1	0.015264832	1.599910617	up
LOC381375	0.015265875	1.604890108	up
Trrp1	0.015265875	1.674335241	up
Cpa3	0.01527503	1.761353493	up
Wdsof1	0.01527503	2.105128288	down
2410008A19Rik	0.015280085	1.567484379	up
Eps8l2	0.015300641	1.649840236	up
C330036H15Rik	0.015301053	1.631038308	up
Serpinb6a	0.015301053	2.102301598	up
Gja5	0.015301053	2.181316137	up
Hspb8	0.01530246	2.084914208	up
Dysf	0.015305009	1.529547811	up
Irf1	0.015305009	1.827239752	up
Rfx1	0.015324852	1.734866142	up
Cux1	0.015335555	1.760463476	down
Aqp1	0.015336008	2.098461151	up
1110035H17Rik	0.015359527	1.737440944	up
Rfc1	0.015359527	1.612091661	up
Upk2	0.015359527	1.896573186	up
Nrbp2	0.015359527	1.794901729	up
Tspan15	0.015377536	1.526413918	up
Ptpn11	0.015380763	1.648685455	up
Dgkb	0.015382416	1.540017366	up
Nqo2	0.01538295	1.760188937	up
AW124722	0.015386781	1.768404603	up
Ncstn	0.015386781	1.897603989	down
Decr2	0.015399882	1.796769381	up
Ing4	0.015399882	1.761608839	up
Mst1	0.0154318	1.615269184	up
Slc25a11	0.015435619	2.4178195	up
6230412O07Rik	0.015445104	1.684808135	up
Pabpc1	0.015449311	1.883055449	up
scl0002223.1_98	0.015460342	1.510006428	down
Crb1	0.015477028	1.519437194	up
Gas2l1	0.01549784	1.680579901	up
Prmt6	0.01549784	1.552326798	up
Mxra8	0.015513633	1.585093021	up

A930011O12Rik	0.015522184	2.90604043	down
LOC100044468	0.015523958	1.84954071	down
Junb	0.01555189	1.767952919	up
Klhl13	0.01555189	4.878860951	down
9629514_325	0.015555255	1.536189318	up
Ggcx	0.015566977	1.810103059	up
Kif16b	0.015566977	1.816019297	up
Poli	0.015570754	1.531311512	up
AI987944	0.015570754	1.702910543	up
Smek2	0.015570754	3.407826662	down
Kcnj10	0.015570754	1.558370829	up
Abcc6	0.015570754	1.665177465	up
A830019P03Rik	0.015584946	1.749596238	up
Fdxr	0.015611158	1.543902755	up
Sdccag33l	0.015611158	1.75575757	down
Tuft1	0.015611158	1.694327235	up
Arhgap28	0.015611158	1.562302589	up
Kcnmb1	0.015623871	1.640515566	up
D14Ertd668e	0.015634487	1.620386243	up
Stac3	0.015634487	1.654299259	up
2210010B09Rik	0.015634487	1.71366787	up
1300011L04Rik	0.015672548	1.864464402	up
Llgl2	0.015672548	1.54646492	up
G3bp1	0.015702609	1.583943844	up
Ttn	0.015709428	1.53362596	up
Slc14a2	0.015712598	1.54462719	up
Rpgr	0.015712598	1.589429617	up
Col17a1	0.015712598	1.66506803	up
0610010E21Rik	0.015712598	1.702003598	down
Hopx	0.015712598	2.21042347	down
Pwwp2b	0.015712598	1.738709688	up
LOC100039175	0.015712598	1.738140464	up
Il18bp	0.015718319	1.725533962	up
Dnajc19	0.0157797	1.548030853	up
Dek	0.0157797	6.360548019	down
Spin2	0.015788345	1.793452263	up
Ifitm1	0.01579231	1.962481976	up
LOC670044	0.015807653	1.5104146	up
6030408C04Rik	0.015809959	1.69873476	down
Vegfc	0.015812602	1.64735055	up
Pdgfb	0.015815824	1.928518772	up
Pi16	0.015815824	1.572577477	up
Lims2	0.01582339	1.615815043	up
1190005I06Rik	0.01582339	1.753389835	up
Ube2e1	0.015824881	1.688503385	up
Gpr1	0.015824881	1.725373387	up
Zfp277	0.015824881	1.588775158	up
Sfrs5	0.015855072	2.319268465	up

Slc24a3	0.0158576	1.602561235	up
Krt14	0.015859652	5.577585697	up
Cyp2j9	0.015859652	1.525645852	up
C330006P03Rik	0.015868926	1.683944106	down
Kat5	0.015886607	1.606329083	up
scl0002337.1_39	0.015895378	1.860794783	up
E130001F20Rik	0.015895378	1.699567795	up
Ezh1	0.015895378	1.571545243	up
Ppap2a	0.015900277	1.625725627	up
Clec11a	0.015901094	1.841278553	up
Colec12	0.01591206	1.744508863	up
Olfr748	0.015924279	1.878022671	up
Pkn2	0.015924279	1.831421852	down
Ndrg2	0.015924279	2.341609716	down
Tmem176a	0.015924279	1.56439805	up
1700030J22Rik	0.015924279	1.529548526	up
Rhov	0.015924359	1.705793262	up
Bag4	0.015926981	1.54095614	up
Dnajc28	0.015952224	1.623480201	up
Syap1	0.015976781	1.50850904	up
1810046K07Rik	0.015976781	1.597674608	up
Abca2	0.015976781	1.623272419	up
Ndst1	0.015976781	1.568942547	down
Ankrd11	0.015976781	1.871367455	down
Sorcs1	0.015976781	1.559650183	up
Fam102a	0.015976781	1.700731516	up
Rarres2	0.015976781	2.030186653	up
Ptpru	0.015983593	2.041645289	up
Steap2	0.015986154	1.779416561	up
Dll4	0.01599353	1.669487715	up
Atp8b1	0.016004279	1.733767867	up
Cnot4	0.016018091	2.203994751	down
Klf4	0.016032334	1.602704167	up
LOC330134	0.016032873	1.597900987	up
3110035E14Rik	0.016032873	1.559252024	down
4732462B05Rik	0.016043887	1.77134347	down
Wdr59	0.016043887	1.597825289	up
D430020J02Rik	0.016043887	1.59990859	up
Ccdc82	0.016051238	1.595047355	up
Ino80	0.016054813	1.616994739	up
Mks1	0.016075429	1.507858634	up
Psmd4	0.016090656	1.606945515	up
Sbsn	0.016090656	1.549102068	up
Copb2	0.016118703	1.599265814	down
Setd6	0.016118703	1.633074284	up
Svop	0.016118703	1.900954962	down
Epha7	0.016131142	2.36863327	up
Pex26	0.016149512	1.788718343	up

Cited1	0.016149512	1.599921107	up
Magmas	0.016153956	1.532277346	down
Fbxw17	0.016172359	2.030739546	up
Emilin3	0.016210258	1.661230207	up
Mtap2	0.016215332	2.891719818	down
Col6a3	0.016216913	1.879945278	up
LOC333459	0.016224878	1.674965859	up
Zc3h18	0.016224898	2.65169549	down
Pnldc1	0.016224898	1.581427336	up
Heca	0.016224898	1.738848686	up
Sgip1	0.016224898	1.610694766	up
Gins1	0.016224898	2.134769678	up
Lsr	0.016254855	1.807268739	up
Chrm3	0.016258748	1.576372027	up
Terf2	0.016258748	1.684105873	down
Mar-10	0.016261362	1.589142799	up
LOC100045522	0.016272966	1.563743115	up
Cbfa2t3h	0.016272966	1.518231273	down
Nnmt	0.016272966	1.736355305	up
Cops5	0.016274514	1.505086184	down
4930402I24Rik	0.016274979	2.281369448	down
Gldn	0.016274979	1.729434252	up
Parva	0.016275929	2.095227003	up
LOC677144	0.016275929	1.549839497	up
Dysf	0.016276673	1.574743271	up
B130008O17Rik	0.016278623	1.681232572	down
Klhdc8b	0.016278623	1.614795566	up
Kcnj5	0.016278623	1.514858723	up
0610010E21Rik	0.016278623	1.796568394	down
Rassf2	0.016278623	1.706613541	up
Slc24a2	0.016309332	1.517876148	up
3000003G13Rik	0.016329374	1.69746232	down
Prss8	0.016329374	1.644970894	up
LOC100040505	0.016350823	1.567203522	up
C530044C16Rik	0.016366422	1.522976637	up
Ptrh1	0.016374717	1.626769424	up
Usp46	0.016374717	1.752451301	up
Shisa2	0.016377427	1.638281584	up
Phlpp	0.016379349	1.530176282	down
Acbd4	0.016387666	1.589290977	up
9130213B05Rik	0.016400479	1.882205248	up
C030026E19Rik	0.016400479	1.640537381	up
Fv1	0.016405623	1.695414066	up
1110049N09Rik	0.016421571	1.575163722	up
D11Bwg0280e	0.016426234	1.613652825	up
Eya1	0.016426234	2.043495178	up
Pla2g10	0.016432229	1.551245809	up
Dynll2	0.016462905	2.353850842	up

Asph	0.016462905	1.914113045	up
1810022O10Rik	0.016462905	1.738990545	down
Myeov2	0.016462905	1.703793526	down
Serpina1a	0.01646312	1.673175216	up
Ppap2c	0.016507382	1.577884793	up
E130309F12Rik	0.016513169	1.818326473	up
LOC670356	0.016520541	2.187096119	down
Hey2	0.016543159	1.881920815	up
Aebp2	0.016550323	1.62104094	up
Mrg1	0.016588008	2.256049633	down
Muc2	0.016588008	1.506180763	up
9630025H16Rik	0.016588008	1.662733913	down
1300007L22Rik	0.016588008	1.778340697	up
Ogn	0.016588008	2.169056892	up
Il28ra	0.016599085	1.824501157	up
Hel308-pending	0.016599085	1.662665963	up
Hoxd11	0.016620832	1.634416461	up
6430514E13Rik	0.016634196	1.502450347	up
Ppapdc1b	0.016652731	1.798686862	up
Clcn3	0.016652731	1.885497212	down
Gucy1a3	0.016652731	1.521927595	up
Prkcbp1	0.016670641	1.705720425	down
Doc2g	0.016683295	1.547374487	up
Glipr2	0.016683295	1.686236024	up
Scnm1	0.016684104	1.854778171	up
Srd5a3	0.01673205	2.019578457	up
Relt	0.01673205	1.751316428	up
AA407270	0.01673205	1.708680868	up
Ssh3	0.016733609	1.650605798	up
Cpne8	0.016733609	1.792655706	up
Mylpf	0.016768323	1.605014205	up
Rnf144a	0.016773403	1.957864404	down
LOC382002	0.016773403	1.553564549	up
Atp9a	0.016777998	1.946009755	up
E130112N23Rik	0.016797269	1.626826763	up
Terf2	0.016807623	4.621348858	down
D130027H15Rik	0.016833093	1.618040681	up
9430088F20Rik	0.016833093	1.616253495	up
Tarsl2	0.01683775	1.814433694	up
Elac1	0.01683775	1.687741518	up
Fat1	0.01683775	1.527489185	up
Tilz3c	0.01683775	1.779049635	up
Odf4	0.016844142	1.561619043	up
9330177P20Rik	0.016844656	1.803807378	up
Il17rc	0.016849209	1.577741861	up
AI593442	0.016849209	1.768951058	up
Egfr	0.016849209	1.669088602	up
Map3k5	0.016851207	1.551097274	up

B230312L03Rik	0.016861562	1.542481303	down
Nsun5	0.016861562	1.694179654	up
Cpsf4l	0.016861562	1.634483099	up
Arl4a	0.016861562	1.618903995	up
Rgs6	0.016863013	1.59399128	up
Cd151	0.016887652	2.299678087	up
4930544G11Rik	0.016887652	1.847041011	up
Bmp4	0.016909976	2.180193901	up
1190003J15Rik	0.016909976	1.527339935	up
6330562C20Rik	0.016909976	1.713461995	up
4930403O06Rik	0.016909976	2.34967041	down
Acta2	0.016941633	2.341049433	up
0610031J06Rik	0.016946133	1.553770065	up
5430434G16Rik	0.016946133	1.592814326	down
Asah3	0.016946133	1.565306902	up
Man1a	0.016946133	1.514261127	up
8430419L09Rik	0.016946133	2.374649286	up
Tbc1d2b	0.016946133	2.312612295	up
Mpdz	0.016946133	1.739132762	up
Chrng	0.016946133	1.60495317	up
Fhod1	0.016946133	1.772767901	up
Hist1h3f	0.016946133	1.787804365	down
Rrm1	0.016946133	2.613793373	down
Usp48	0.016946133	1.625525951	up
Olfr725	0.016946133	1.622147203	up
1600021P15Rik	0.016988386	1.906464338	up
2010100O12Rik	0.016992521	1.561709404	up
Pfas	0.017002273	1.503924131	up
2310036D04Rik	0.017002273	1.642930627	up
Fibp	0.017004967	1.897043705	up
LOC232606	0.017030537	2.191022635	down
Ermp1	0.017030537	1.627520919	up
Nol3	0.01704221	1.528957605	up
Foxj3	0.01704221	1.540936589	up
Hoxa4	0.017079974	1.69625628	down
BC002216	0.017079974	3.583628893	up
Zdhhc14	0.017080702	1.819124103	up
Mylk	0.017088102	2.00488019	up
Rxrg	0.017100141	1.762061358	up
Gls2	0.017103985	1.783101201	up
Wdr13	0.017110249	1.66203475	up
Coro2a	0.017116696	1.79226923	up
Siat7b	0.017116696	2.866936445	down
LOC385952	0.017116696	1.502984166	up
Pbrm1	0.017116696	1.555051923	down
Chd5	0.017124495	1.799323916	up
Slmap	0.017130984	1.559376121	up
Calr4	0.017130984	1.616881728	up

Tmem54	0.017147914	1.836074352	up
Ilvbl	0.017151028	1.759037256	up
A430106G13Rik	0.017166771	1.965897083	down
Acaa2	0.017168785	2.013747454	up
Etv1	0.017182538	1.515308499	up
Ghr	0.017182538	2.060193062	up
Sema6d	0.017207995	1.892050147	up
Crmp1	0.017221388	1.67746222	down
D10Ertd610e	0.017231699	1.93592155	up
Obrgrp	0.017236836	1.924490452	up
P140	0.017236836	1.722195268	up
Mst1r	0.017239882	1.682134748	up
scl0001487.1_50	0.017248849	1.911684394	up
Nrsn2	0.017251032	1.601861954	up
Nufip2	0.017251032	1.586651802	down
Olfr982	0.01725944	1.690323234	up
A330021D07Rik	0.017264539	1.56759584	down
5430432N15Rik	0.017272055	1.839270115	up
Pkp4	0.017276937	1.990332007	up
Zfp281	0.017276937	2.413378	down
Pcdhga4	0.017283516	1.61386168	up
ORF34	0.017299729	1.591590524	up
Slc7a3	0.017302141	1.533806324	up
1700065O13Rik	0.017307157	1.884607196	up
Slc9a6	0.017308209	1.627861142	up
Plekha7	0.017308209	1.545806408	up
Hbp1	0.017312452	1.762895346	up
Tyk2	0.017318981	1.578006864	up
D130017N08Rik	0.017318981	1.503252387	down
Tgif1	0.017371154	1.705920577	up
Plek	0.017371154	3.714321613	down
5830415F09Rik	0.017371154	1.678330898	up
Tle6	0.017371154	1.832705855	up
LOC381134	0.017374128	1.74949038	up
Pcdh10	0.017374128	2.201182365	up
Sort1	0.017386068	1.556075573	up
Tbxa2r	0.017386563	1.70928371	up
Nxph4	0.017419066	2.202250958	down
Thbs3	0.017426914	1.538815856	up
Avil	0.017441306	1.523539305	up
Slc39a7	0.017477715	1.568816781	up
Lifr	0.017486943	1.614066362	up
Nup37	0.017507335	1.745683432	up
Neu2	0.017507335	1.667151451	up
Thy1	0.017507335	1.66460228	up
Oprs1	0.017507335	1.626602054	up
Nfrkb	0.017507335	1.554434538	up
Rffl	0.017507335	1.590759277	up

Hpcal1	0.017516965	1.623110533	up
Col4a5	0.017525455	1.772486687	up
C2cd2l	0.017536944	1.941580057	down
Rad23b	0.01754104	1.756997824	down
Mtfmt	0.017565804	1.533189416	up
Slc14a1	0.017571062	1.913003087	up
LOC383873	0.017584661	1.695057988	up
G630007B09Rik	0.017584661	1.543118596	up
LOC386094	0.017588949	2.268827438	down
Pnmal1	0.017588949	1.550006747	up
Mcf2l	0.017598696	1.55270195	up
1700087I21Rik	0.017602705	1.600924373	up
A930002I21Rik	0.017610809	1.626118541	up
Marcksl1	0.017610809	1.63034296	down
2010111I01Rik	0.017610809	1.647946477	up
Cpxm1	0.017610809	2.043609619	up
Inha	0.017610809	1.801936984	up
Clca3	0.017617293	1.826119542	up
Mina	0.017636214	1.515146375	up
LOC385583	0.017644007	1.588102102	up
Icam2	0.017661203	2.001645088	up
Zc3h14	0.017703872	1.662815332	up
Sh2d3c	0.017708598	1.79164958	down
6030470M02Rik	0.017719222	3.118816376	down
Nedd4	0.017721515	1.531288147	up
Pacsin3	0.017725209	1.818226814	up
Kcnab1	0.017725209	1.682526231	up
Mbnl	0.017725209	1.739828706	up
Ephb6	0.017725209	1.591968179	up
Mppe1	0.017725209	1.542050719	up
Anks6	0.017736709	1.587164044	up
Stfa1	0.01774041	1.737041831	up
2610507B11Rik	0.01774041	1.554041505	up
1200009I06Rik	0.01774041	1.580189824	up
Vgll3	0.017746802	1.692026377	up
Slc20a2	0.017746802	1.564995647	up
Mrgpra2	0.017746802	1.640662432	up
A830054H12Rik	0.017746802	1.759100437	up
Cmya5	0.017746802	1.676169157	up
Ppt1	0.017748324	1.9413445	up
Plxna4	0.017756783	6.065916538	down
Seh1l	0.017774912	1.68353653	up
Slc35b1	0.017793	2.897785187	down
Col6a1	0.017793	1.927818418	up
Fgfr1op2	0.017793	6.043513298	down
Mtmr11	0.017807081	1.623983622	up
Nrn1l	0.017817088	1.598055244	up
Kif26a	0.01782793	1.76064384	up

LOC100045542	0.01783796	2.050422192	up
A130090K04Rik	0.017841084	1.515704513	up
Klk7	0.017859813	1.512227535	up
Csnk1g1	0.017885832	2.190920591	down
4930432K21Rik	0.017887132	1.884579778	up
Epb4.1l4b	0.017916821	1.772772312	up
6430573H23Rik	0.017917058	1.621554613	down
Sh3md4	0.017917069	1.545723915	up
Lonrf2	0.017931627	1.935533762	up
Dlg2	0.017931627	1.799797654	up
AV273951	0.017931627	1.500017166	up
Slc16a6	0.017931627	1.603480697	down
Styx	0.017931627	1.584901691	down
Epb4.1l3	0.017931627	1.582636118	up
Snx2	0.017940605	2.141568184	up
1700111D19Rik	0.017940605	1.520380139	up
Rfc2	0.017940605	1.726641178	down
Cntnap4	0.017940605	1.501362324	up
Spata2L	0.017940605	1.837315202	up
Qars	0.017944405	1.589814186	up
B230209C24Rik	0.017953733	1.805113912	up
LOC100038993	0.017954553	1.915179372	up
Sema4f	0.017957036	1.874284148	up
Galm	0.017977804	1.981630206	up
LOC385877	0.017987138	2.17973733	down
LOC100044411	0.017987138	1.711489081	up
8030404L10Rik	0.018000597	1.597732067	down
1700080G18Rik	0.018000597	1.53419447	up
Timeless	0.018019052	1.771934152	down
Cova1	0.018028304	1.854526758	down
Larp-pending	0.018046448	1.56851089	up
Galnt10	0.018046448	1.869497895	up
Ripk4	0.018046448	2.464329481	up
Pcp4l1	0.018059416	2.00957036	up
1600017E01Rik	0.018059416	1.624910235	up
Magi1	0.018068826	1.938788414	up
Srpx2	0.018068826	1.873832703	up
E030038G15Rik	0.018068964	1.732204914	down
Il17d	0.018070715	1.535343528	up
Ube2q1	0.018070728	1.695650458	down
Mpst	0.018101076	2.012371302	up
Mre11a	0.018132227	1.593019128	up
Psmb5	0.018176934	1.981876135	down
Rabif	0.018176934	2.049121618	up
Ltb4r1	0.018199939	1.560956717	up
Tmem111	0.018227374	1.872704864	up
Bcl11b	0.018227447	1.740461111	down
Arl2bp	0.018232346	1.997327089	up

Foxp2	0.018240303	1.948508143	up
Gad1	0.018246351	4.026839256	down
Prss36	0.018246351	1.539316893	up
Cd97	0.018246351	1.944955826	up
LOC666676	0.018255321	1.783014298	up
Mrc2	0.018262032	1.662971735	up
Tnrc6c	0.018262032	1.673379421	down
4930515G13Rik	0.018262032	1.834805727	up
D11Wsu99e	0.018272938	1.643987894	up
Slmap	0.018280786	2.184310198	up
Cnksr3	0.018285697	1.523344159	up
Dncl2a	0.018285697	1.727690816	down
Luc7l	0.018285697	1.792991281	up
LOC674997	0.018285697	1.743226409	up
1700106J16Rik	0.018299839	1.589351893	up
Pkp1	0.018305644	2.543884277	up
Stx18	0.018307459	1.50829339	up
Scml2	0.018307459	1.662477732	up
Nfatc1	0.01830828	2.385699749	up
Ripk4	0.018316345	1.717285276	up
Apobec3	0.018316345	1.546275139	up
EG626367	0.018316345	1.793059588	down
Akr1b8	0.018321302	1.755342364	up
Angptl2	0.018321302	1.627439618	up
Kcnk2	0.018326594	1.952589035	down
Pds5a	0.018326594	1.643751979	up
Laptm4a	0.018326594	2.131327391	up
Morn2	0.018355195	1.85149312	down
Uaca	0.018355195	1.759548187	up
Actn2	0.018408369	1.730874062	up
Cpeb4	0.018409768	2.556651115	down
Pak1ip1	0.018409768	1.760069728	down
Lasp1	0.018409768	1.632334471	up
4930565B19Rik	0.018412946	1.743886113	up
Hspa4l	0.018440392	1.973282814	down
Ctsc	0.018440392	1.798335791	up
Foxred1	0.018454129	1.520336628	up
Kif20b	0.018462252	3.039420605	down
LOC100043257	0.018473985	2.056152582	up
Slc7a7	0.018473985	1.505616546	up
9430077D24Rik	0.01848428	1.6399194	up
Eln	0.018489299	2.773694277	up
382044	0.018501489	1.546400785	up
LOC333405	0.018515199	1.749730349	up
Epyc	0.018515514	2.602643728	down
Col22a1	0.01851595	1.713129163	up
Stx17	0.01851595	1.600913525	up
Cartpt	0.018528407	1.640872955	up

Asph	0.018531429	1.620009184	up
LOC386246	0.018531429	2.770797968	down
Ube2j2	0.018544886	1.550761223	up
Ftsj	0.018544886	1.537119865	up
Ift81	0.018546728	1.550028563	up
LOC269919	0.01855175	1.501199484	up
Cntnap4	0.018580405	1.730726719	up
Prmt2	0.018592948	1.630178094	up
8030453O22Rik	0.018596754	1.599879384	up
Irak4	0.018605145	1.560069084	up
Pxn	0.018605145	1.657881379	up
1200014J11Rik	0.018605145	1.666870475	up
Spag5	0.018605145	1.768015385	down
Prkcdbp	0.018605145	1.842539907	up
Slc30a7	0.018605145	1.856644154	up
Rab25	0.018605145	2.317437649	up
Rxrb	0.018605145	1.658181667	up
Col11a1	0.018626818	2.961684942	down
Bard1	0.018627707	1.500862002	up
Farsb	0.018642658	1.514271975	up
Tnip1	0.01866921	1.568222761	up
Comtd1	0.018673882	1.623176098	up
Wdr4	0.018686699	1.544607997	up
Nfkb1	0.018686757	1.526234984	up
Pip5k1a	0.018687887	1.764115572	down
LOC381431	0.018710595	1.536129952	up
Trrp2	0.018710595	1.75210321	up
Galt	0.018763412	2.111257792	up
Csnk2a1	0.018772544	1.672163248	up
Bcl11b	0.018772544	4.278352261	down
Il1rap	0.018772544	1.638616681	up
Nsmaf	0.018782882	1.606626153	up
1700065A05Rik	0.018785477	1.742350578	up
Agtr1a	0.018788563	1.549103022	up
Yipf5	0.018788563	2.609052897	down
Sox2	0.018788563	4.030270577	down
Ret	0.018788563	1.627711773	up
Zc3h14	0.018801935	2.140615463	up
Jazf1	0.01887276	1.949540854	up
Fes	0.018873638	1.656446934	up
Mpzl1	0.018873638	1.752617836	up
Znrf2	0.018876877	1.567787528	down
LOC245305	0.018892689	1.56282413	up
9430015L11Rik	0.018904449	1.698910713	up
LOC100047675	0.018904449	1.861332536	up
Vps39	0.018904449	1.600494981	up
Slc25a45	0.01890545	1.86148417	up
Gstcd	0.018916216	2.100327969	up

Smox	0.018921141	1.809186101	up
Invs	0.018946121	1.556851029	up
2610035D17Rik	0.018946121	1.550805688	down
9530068E07Rik	0.018946121	1.569489718	up
Ptcd2	0.018947847	1.771337509	up
Hbp1	0.018949969	1.848447442	up
Ptgfrn	0.018949969	1.705863476	up
Kctd5	0.018949969	1.717784882	up
Ctdspl2	0.018949969	1.746198177	up
Olfr100	0.01895364	1.598871589	up
Acan	0.018957976	2.910867214	down
Thbs2	0.018977318	2.531825304	up
Bahcc1	0.019014407	1.869986296	up
2310047M10Rik	0.019018065	1.690625787	up
Ccdc93	0.019018065	1.533792257	up
Taok1	0.019022422	1.614958406	down
5830405N20Rik	0.019022422	1.6229105	up
Fus	0.019024855	1.859306812	up
Zfp526	0.019028571	1.63176477	up
Nfkbia	0.019037595	1.601578713	up
Slc4a8	0.019045835	1.590860844	up
1700016J18Rik	0.019046893	1.521749139	up
Golga7	0.019047411	1.911085486	up
Apobec1	0.019047411	1.563542962	up
4833408A19Rik	0.019057207	1.689747334	up
LOC545208	0.019057207	1.587709785	up
Tyms	0.01906701	1.540188313	up
Hs3st1	0.01909117	1.711952686	up
Prkcbp1	0.01909117	1.601651907	down
A430073A17Rik	0.01909117	1.539066434	up
Ankmy2	0.01909117	1.569953919	down
Ece1	0.019136557	2.07595396	up
LOC100044699	0.019136557	1.665133715	up
LOC100039728	0.019150907	1.566990852	up
Gigyf1	0.019152992	1.500017285	down
1110012D08Rik	0.019152992	1.915297866	up
6130401L20Rik	0.019152992	1.591998339	up
1700027N10Rik	0.019152992	1.521033526	up
Cdk2	0.019152992	1.665256023	up
Nr1h3	0.019153344	1.587687135	up
Tnfsf13b	0.019168587	1.764905214	up
Notch4	0.019172888	2.045795679	up
Zmpste24	0.019172888	1.803760052	up
4930546H06Rik	0.019172888	1.604742765	up
Arsg	0.019187808	1.923086882	up
6430548M08Rik	0.019187808	1.660259128	down
Acad10	0.019215489	1.611324072	up
Tlcd2	0.019223901	1.53439188	up

Kifc2	0.019241247	1.704722166	up
Tpd52	0.019248843	1.818713784	up
C8g	0.019248843	1.574211359	up
6330416L11Rik	0.019265076	2.140543938	up
Gnpat	0.019265076	1.632168293	up
Zfp750	0.019272536	1.831043005	up
0610007P14Rik	0.019272536	1.584949851	up
A830081I21Rik	0.01931783	1.559014678	up
Macrod2	0.019318473	1.506709218	up
Phtf1	0.019337624	1.58409822	up
LOC386330	0.019364636	5.670508385	down
Cln3	0.019365735	1.785495997	up
Asb4	0.019371817	5.664105892	down
Slc27a4	0.019380923	1.590130091	up
Tmco4	0.019380923	1.670428515	up
Fbxl12	0.019390566	1.704402566	up
C230091E20Rik	0.01941031	1.540088534	down
Manba	0.019445384	1.523487091	up
Lpcat2	0.019452056	1.648693323	up
Pard3	0.019468626	1.587090254	up
6430578G21Rik	0.019495532	1.536979079	down
Tekt1	0.019516265	1.640228748	up
6430537F04	0.019517733	1.56843245	up
D530033A12Rik	0.019517733	1.686513901	up
1700025H01Rik	0.019517733	1.664431453	up
D330027H18Rik	0.019530708	1.723230958	down
Pter	0.019530708	1.612497807	up
Zfp641	0.019539241	1.799044251	up
LOC329506	0.019539241	2.016060829	up
Cuta	0.019545576	1.827519774	up
Galntl2	0.019545576	1.778004289	up
4930402H24Rik	0.019545576	1.629089117	down
Gtf3c5	0.019545576	1.655191898	up
Rnf8	0.019545576	1.738879561	up
Mbd3l2	0.019545576	1.880525589	up
Eef1b2	0.019545576	1.817223549	up
D030070I18Rik	0.019566519	1.534617543	up
Myo9b	0.019566519	2.033993959	up
Olfml2a	0.019579794	1.589794517	up
Hsd17b11	0.019579794	1.58490479	up
Masp2	0.019601461	1.59989953	up
Dst	0.019620908	1.986138701	up
C920027I18Rik	0.019621775	1.936953425	down
LOC100045678	0.019641917	1.620488524	down
Egfr	0.019685267	1.636463761	up
2810405K07Rik	0.019685267	2.502218962	down
C630016N16Rik	0.019705122	1.573440433	up
0610012D14Rik	0.019705122	1.600484848	up

1700047I17Rik1	0.019709148	1.913920283	up
Ppbp	0.019769434	3.933816433	down
LOC624662	0.019769434	1.875378609	up
Csf2ra	0.019769434	1.566088319	up
Gsta4	0.019791061	2.21587038	up
Neu3	0.019808099	1.607979894	up
Lpcat4	0.019814968	1.509519458	up
4632411B12Rik	0.01981649	1.805941463	up
Srpk2	0.01981649	1.759709477	up
R74862	0.01985397	1.972116828	up
6330406I15Rik	0.019856818	1.697691321	up
D030067L12Rik	0.019880369	1.68990016	down
Mobkl2c	0.019887889	1.5072577	up
Bcl2l11	0.019895401	2.099246025	up
6030408B16Rik	0.019902388	1.747760296	up
Smek2	0.019910645	3.201736927	down
Depdc5	0.019922899	1.676931262	up
Adcy3	0.019945916	1.940603852	up
Rab38	0.019945916	1.532924414	up
Otud7b	0.019945916	1.671985984	up
Cxcl16	0.019946849	1.745026112	up
Ppm1f	0.019952619	1.617442846	up
Msn	0.019952619	1.935514569	up
Ythdc2	0.019952619	1.631167531	up
Ttll4	0.019952619	1.584427357	up
Mef2c	0.019952619	1.64749217	down
Polr3d	0.019965924	1.57905829	up
Rhob	0.019981235	2.576362133	down
Gulp1	0.019981235	1.82683444	up
Mvp	0.019993104	1.699517965	up
Zc3h12a	0.019996004	1.607051253	up
Hexim2	0.019996004	1.728829384	up
Car15	0.019996004	1.514352918	up
Ube2i	0.019996004	1.527671099	up
LOC380854	0.019996004	1.524170518	down
Nadsyn1	0.019996004	1.636990905	up
Ptprm	0.020007389	1.672431231	up
Sertad3	0.02002774	2.51334405	up
Crk	0.02002774	1.567750692	up
Plekhg2	0.02002774	1.619399309	up
Pknox2	0.02002774	2.066895723	down
Txnip	0.020034289	2.000159264	up
Rapgef5	0.020037541	1.586317897	down
Prdx2	0.020037541	2.441828966	down
Ifnar2	0.020052493	1.557979584	up
2900056M07Rik	0.020052493	1.669989467	down
Rgs11	0.020052493	1.60239768	up
Cabyr	0.020067066	1.641574979	up

Fbxl11	0.020067066	1.554006815	down
LOC385822	0.020067066	1.643773794	up
Mdga2	0.020067066	1.596553206	down
Slc46a3	0.020067066	1.502277136	up
Rad51l3	0.020102821	1.593988299	up
Col18a1	0.020129181	2.608283281	up
2700062C07Rik	0.020129181	1.990626335	up
Dock6	0.020129181	1.790713787	up
1700082G03Rik	0.020169903	1.814059138	up
Pcmt1	0.020174058	2.676912308	down
Trpm6	0.020175999	1.524550438	up
Plcz1	0.020176793	1.522794366	up
St6galnac6	0.020198923	1.534910202	up
Nup88	0.020206871	1.874432564	up
Zfp212	0.020231422	1.533407688	up
Dnahc11	0.020231422	1.538279057	up
Ildr1	0.020249326	1.650166154	up
Nr5a2	0.020249326	1.584414005	up
Eefsec	0.020251149	1.600026965	up
Phka2	0.020254023	1.675261259	up
Cpsf3	0.020283788	2.64977026	down
Khdrbs3	0.020312212	1.813898087	up
Mis12	0.02032567	2.050075293	down
Aof1	0.020327551	1.623465657	up
5730409G15Rik	0.020331061	1.540131569	up
Stard13	0.020331061	1.853927612	up
Cdk2	0.020349132	1.997962952	up
A530079D03Rik	0.020370152	1.503989816	up
Ankrd27	0.020374037	1.578647733	up
H2-Ab1	0.020376839	1.633212209	up
Xrcc6	0.020376839	1.554399371	up
Dusp11	0.020384604	1.866850615	up
Slc11a2	0.020393895	1.672279	up
2210010N04Rik	0.020393895	1.621187687	up
Kbtbd7	0.020399446	1.873059869	up
Aipl1	0.020400038	1.658350945	up
Rab11fip3	0.020411616	1.526675224	up
Cyp4f13	0.020411616	1.668002963	up
BC006965	0.020411616	1.501077652	up
Cobll1	0.020426372	1.781957388	up
Ppl	0.020426372	2.220977068	up
Arhgdig	0.020427963	1.735333562	up
3230401L03Rik	0.020427963	1.623861074	down
Scn8a	0.020427963	1.608902454	up
3110079O15Rik	0.020427963	5.158185005	down
Atp2c1	0.020427963	1.673639298	up
Micall2	0.020436537	1.729387164	up
Chchd3	0.02044363	1.594190598	up

Dnaja2	0.020455549	1.651825547	up
Pou4f2	0.020455549	1.656441689	up
Il3ra	0.020464836	1.611567378	up
9330104G04Rik	0.020467071	1.573187232	up
Vegfb	0.020493534	1.550295949	up
LOC386205	0.020509731	1.727622628	up
Rad51	0.020515097	1.847840071	up
Galntl4	0.020522121	1.817670822	up
Ndfip1	0.020522121	1.962070584	up
Tbx2	0.020522421	1.751884937	up
Ggt7	0.020527292	1.516577125	up
2810453I06Rik	0.020531317	1.689994812	up
Amhr2	0.020533517	1.585989475	up
Slc11a1	0.0205436	2.14029789	down
Ei24	0.0205436	1.6140939	up
Cdh3	0.0205436	1.704200745	up
Rnf8	0.020556936	1.532548547	up
B830045N13Rik	0.020556936	1.912617445	up
Dok5	0.020584293	1.709348679	up
2300006M17Rik	0.020584293	1.811195493	down
Krt5	0.020586362	2.158652544	up
Gm609	0.020616418	1.836810947	up
Edem1	0.020622162	1.60655725	up
Lep	0.020626465	1.846356869	up
1520402A20Rik	0.020641664	1.55072844	up
Il6ra	0.020655325	1.864237309	up
Ric8b	0.020671457	2.12016654	down
LOC380986	0.020687413	1.566044927	up
Chic2	0.020693155	1.564494252	up
scl0002050.1_27	0.020716788	1.552209616	up
Wdr90	0.02071951	1.675659537	down
Gnpda1	0.020736147	1.529057264	up
LOC382555	0.020763814	6.83583355	down
Actr1a	0.020770241	1.69549489	up
Mkrn1	0.020776117	1.732840061	down
Tm2d3	0.020779649	1.517449737	up
BC006779	0.020783709	1.524500847	up
Plch2	0.020783709	1.546811104	up
Klf6	0.020783709	1.616447687	down
Pdpk1	0.020805543	1.717574596	down
Pik3ca	0.020807648	2.848953486	down
C85492	0.020814469	1.771116018	up
Pigs	0.020817675	1.539017439	up
Tmem132e	0.020818476	2.019565582	up
Stmn4	0.020818476	2.048155069	up
Zfp288	0.020850908	1.895069122	down
Il4i1	0.020853834	1.516913533	up
Ramp1	0.020853834	1.611636877	up

B3galt4	0.020861516	1.649116874	up
EG432681	0.020861516	1.558766246	down
6720480F11Rik	0.020887401	1.501701117	down
3110001P07Rik	0.020889109	1.538215756	down
Ggta1	0.020943923	1.651818872	up
Dazap1	0.020950966	1.567214131	up
Aldh1a2	0.020951601	1.605857968	up
Fabp7	0.020987548	5.064826012	down
Lbxcor1	0.020987548	1.517209172	down
Rbms2	0.020987548	1.594272733	up
Ttc19	0.02099626	1.550361991	up
St5	0.02099688	1.537122846	up
Slc37a4	0.021032162	1.787099719	down
Syngr1	0.021064593	1.836171627	up
Hs2st1	0.021065243	1.738355517	up
Ctnnd2	0.021099117	1.884401202	up
Mylk	0.021102142	1.819881797	up
Nab1	0.021140594	1.537516475	up
Epb4.1	0.021140594	1.884973764	down
mKIAA1090	0.021140594	1.57806778	up
St6galnac3	0.021146337	1.525290251	up
D830030K20Rik	0.021148397	1.529114246	up
Gstk1	0.021153895	1.935761571	up
C430049K18Rik	0.021179311	1.532276034	up
Rxfp3	0.021179311	1.524842739	up
Smad3	0.021179311	1.546805143	up
Stoml2	0.021179311	1.661239624	up
Lima1	0.021179311	1.978877544	down
Greb1	0.021179311	1.583178043	up
E330034L11Rik	0.021179311	1.558530211	up
Flot1	0.021179311	1.589307427	up
Sirt4	0.021179311	2.099212885	down
Mylk	0.021179311	1.5818367	up
Ccl25	0.021179311	1.951863527	down
Sil1	0.021211697	1.600662828	up
Nme7	0.021215862	1.547311306	down
Car2	0.021216676	1.550095081	up
D4Ertd681e	0.021239903	2.287807465	down
Cdkn1b	0.021243591	1.570105076	down
Gdf1	0.021243591	1.551178575	up
Thsd2	0.021243591	1.529016018	up
Abca15	0.021246577	1.542894125	up
Slc26a6	0.021246577	1.767056584	up
Ank3	0.021250289	2.969679833	down
Gsc	0.021276303	2.027689219	up
Gpsm3	0.021276303	1.547520876	up
BC068157	0.021279801	3.287061453	down
C78339	0.021303391	1.605430007	up

Wrn	0.021303391	1.769755602	up
Fstl5	0.021303391	4.335448742	down
Tmprss5	0.021310216	1.589292526	up
Idb2	0.021319006	1.550766587	down
Prkcdbp	0.02133303	1.972087622	up
Lefty1	0.021346349	1.564576626	up
Cobll1	0.021372603	1.749427795	up
Tlk2	0.021372603	1.80414927	down
Ndel1	0.021419523	1.547183991	up
Plcg2	0.021480626	1.661407828	up
Brd3	0.021528855	2.717964172	down
D130046C19Rik	0.021528855	2.021287203	up
Tmem132c	0.021528855	1.72615552	up
Hsf2	0.021535965	1.519537687	down
Rnaset2b	0.021549933	1.563409925	up
Cryab	0.021555008	1.600483537	up
Rbms2	0.02155583	1.65386641	up
LOC384887	0.02155583	1.537421346	up
Echdc2	0.021560969	1.587525249	up
Srd5a3	0.021561157	1.662363052	up
Pi4k2b	0.021573726	1.681227088	up
Gm1587	0.021574479	1.520992518	up
Mnat1	0.021574479	1.602127791	up
Ccdc3	0.021598799	2.054102659	up
C530008M17Rik	0.021598799	3.264620781	down
Efr3a	0.021598799	2.16063714	up
Prim2	0.021627683	1.696676493	up
Ntrk3	0.021638358	1.626795888	down
Auh	0.021639856	1.594566107	up
D830014E11Rik	0.021639856	1.508249283	up
Prkd2	0.021656668	1.667649865	up
Trip6	0.02166483	1.731377482	up
Mfsd3	0.02166483	1.551036835	up
Ptrh1	0.021680325	1.55980444	up
D330050I23Rik	0.021680325	1.676500797	down
Mtss1	0.021691643	1.642255783	up
Lrmp	0.021697888	1.515024781	up
Rab11fip2	0.021702031	1.938655257	up
D030005B14Rik	0.021740791	1.505499363	up
Nek1	0.021740791	1.817376852	up
Inadl	0.021740791	1.53197968	up
AI428936	0.021740791	1.596413255	up
Sos2	0.021740791	2.000430107	down
Col24a1	0.021790117	1.610278606	up
BC021919	0.021808773	1.54728508	up
A230050P20Rik	0.021813124	1.5283072	up
Tes	0.021813562	1.631334186	up
Pogz	0.021814093	2.138189554	up

Jam2	0.021819953	2.400185823	down
Bcl11a	0.021835379	2.041830063	down
Myo5b	0.021845812	1.77299726	up
Rimbp2	0.021845812	1.59570837	up
Trip10	0.021872154	1.618603349	up
Vps35	0.02188685	1.514089108	up
Egln1	0.021903884	1.659017444	up
Gja5	0.021903884	2.117199421	up
Cacna2d3	0.021903884	1.594289899	up
Mical1	0.021946779	1.595190167	up
Nfatc1	0.021946779	2.159632921	up
Dhx9	0.021946779	1.600957274	up
Pex10	0.021946779	1.834176183	up
Susd2	0.021958865	1.653440595	up
Eef1e1	0.021958865	1.677147627	down
Rab26	0.02196043	1.932594061	up
D230005E09Rik	0.021976218	1.941574216	down
scl0004010.1_24	0.021976218	1.581531048	up
LOC100048332	0.021976218	1.680161595	up
2310036I02Rik	0.021983109	1.648163676	up
Mpv17	0.021984389	1.551789999	up
Narg1	0.021984389	1.971160889	down
Actg2	0.021996483	2.055608511	up
Mscp	0.021996483	1.649073839	up
Cdh8	0.02201118	1.718152165	up
E130114P18Rik	0.022015598	3.115866661	down
Wfdc10	0.022038236	1.570064902	up
Ccdc71	0.022038912	1.612516642	up
Mllt4	0.022042671	1.815965772	down
2310046M24Rik	0.022045812	1.55982244	up
Prmt5	0.022112366	1.651705265	up
Pip4k2b	0.02212848	1.613613844	up
LOC626309	0.022130856	2.09135747	up
Asb2	0.022133796	1.587048531	up
LOC675567	0.022161042	1.589429736	up
3110004L20Rik	0.022191562	1.882207155	up
Lonrf3	0.022203172	1.767793417	up
Rasl11b	0.022211712	1.523000121	up
Btrc	0.022216052	1.599065304	down
Nrn1	0.022216052	1.510068417	down
Zbtb40	0.022224484	1.660732865	up
1810015C11Rik	0.022224484	1.775437951	up
Klc1	0.022231374	1.524459362	down
5730593F17Rik	0.022231374	1.600599647	up
Sec63	0.022246111	1.722755909	down
Zfp62	0.022246111	1.545906782	down
LOC384842	0.022253875	1.75547874	up
Npy	0.022259386	1.670900345	up

Spast	0.022271477	2.048665524	down
Gusb	0.022280375	1.816432238	up
Tirap	0.022286011	1.842573404	up
P2ry13	0.022287119	1.505988598	up
Dnajb11	0.02229788	1.507345796	down
Ajap1	0.022307318	1.863787413	up
LOC100047670	0.022312582	1.604246497	up
Lias	0.022312582	3.216734886	down
Limk2	0.022323865	1.603653193	up
Fblim1	0.022333605	2.173412561	up
Accn3	0.022333907	1.655033469	up
2400003C14Rik	0.022334963	2.138006926	up
Zfp365	0.022380251	1.525925994	up
Aifm3	0.022380251	1.57817471	up
Tnrc15	0.022380251	1.602196813	up
LOC100044376	0.022388142	1.593915939	down
Lrrk1	0.022388142	1.701816559	up
Becn1	0.022399722	1.993611932	up
Dnmbp	0.022410052	1.64814353	up
Arl3	0.022429289	2.178170204	down
Crip3	0.022429289	1.524590492	up
Zbtb9	0.022432169	1.556682229	up
Ramp3	0.022432752	1.684131265	up
D6Wsu176e	0.022432752	1.580286741	up
Dek	0.022432752	6.341698647	down
Scrn1	0.022459136	1.55444026	up
1810022C23Rik	0.02247211	1.533457995	up
D4Wsu114e	0.022479946	1.681880117	down
LOC244495	0.022480419	1.55705142	up
Ugt1a10	0.022528546	1.567747712	up
Mzf1	0.022528546	1.608070016	up
Hcfc2	0.022528546	1.668494701	down
Ttl	0.022541963	1.659464002	up
Pik3c3	0.022541963	1.652829647	up
Paox	0.022582641	1.856645346	up
Tmub1	0.022582641	1.527398825	up
Nxph2	0.022582641	1.577316999	up
Rnf186	0.022582641	1.598333716	up
Ctsh	0.022582641	1.71374166	up
Reep4	0.022588424	1.658675551	up
Niban	0.022604272	1.511819124	up
Tssc1	0.022620894	1.842508674	up
Sox4	0.022620894	1.783293724	down
Fastkd2	0.022622502	1.568434835	up
Glrb	0.022630373	1.672539115	up
Foxp3	0.02263473	1.628240347	up
Ttn	0.022642314	1.648602247	up
C030003D03Rik	0.022662357	1.633996368	up

Atp12a	0.022680193	1.53197968	up
Tor1b	0.022684358	1.570459366	down
LOC669168	0.022684358	2.294208288	down
Coq10a	0.022684358	1.695808411	up
Wtip	0.022695728	1.727808833	up
1700037C18Rik	0.022697877	1.607562184	up
Cxcl12	0.022697877	1.714631081	up
Scly	0.022734879	1.608123064	up
LOC100045877	0.022741614	1.578719258	up
4931403I22Rik	0.022741614	1.79199779	down
Itgb4	0.022741614	2.070733547	up
Mrpl10	0.022741614	1.524195671	up
6330442E10Rik	0.022755973	1.594863772	up
B930097H17Rik	0.022762269	1.542278647	up
Vps8	0.022766646	1.594685435	up
Gas6	0.02277375	1.773910284	up
9030409G11Rik	0.022777239	1.598408818	up
Dhrs1	0.022828376	1.789581418	up
EG434907	0.022830237	1.586091518	up
Nde1	0.022832707	2.13058877	up
Rrp7a	0.022833409	1.528066397	up
Pla2g4b	0.022873538	1.782537818	up
A230020C19Rik	0.022873538	1.814780951	down
Plekhg5	0.022873538	1.780961394	up
Ptprk	0.022873538	1.602407932	up
Cd248	0.022873538	2.008024216	up
Snora65	0.022887066	1.664454937	up
Glt8d2	0.022894763	1.711622596	up
Rab34	0.022928283	1.829192758	up
LOC641366	0.022943756	2.390524387	down
9530027K23Rik	0.022949552	1.856426597	up
Cacna1h	0.022983547	1.644453049	up
Hhat	0.022988441	1.552055717	up
LOC100047009	0.022996048	1.652647853	down
Dgat1	0.022996048	1.595440269	up
Prpf31	0.022996113	1.722579956	up
LOC100047579	0.022996113	1.598755717	up
LOC545487	0.023002705	3.931880474	down
Frrs1	0.023008572	1.740227461	up
Sulf2	0.023008572	2.155371189	down
Coq6	0.02307003	1.615132213	up
Tnks	0.023080926	1.661463022	down
Slc43a1	0.023088943	1.548285723	up
LOC100047264	0.023117075	1.900161505	up
Rps19	0.023119031	1.515654564	up
D430023I21Rik	0.023119031	1.620405436	up
Adam15	0.023120472	1.826420546	up
Clta	0.023125744	1.79290092	up

Papss2	0.023125744	2.034782171	down
Plscr3	0.023125744	1.514851689	up
Slc7a2	0.023133865	1.593890786	up
Fvt1	0.023141012	1.590964317	up
D230048N11Rik	0.02314801	1.796143413	up
Snn	0.023168709	1.511974692	up
Dgke	0.023168709	1.510702014	up
Ephb2	0.023169875	1.642067432	down
Oxt	0.023182906	1.528424501	up
Zscan20	0.023207942	1.565991044	up
Efna5	0.023245871	1.599610806	up
Snhg11	0.023266332	1.806068659	up
LOC381981	0.023266332	1.520785093	up
Rhoc	0.023277732	1.640039682	up
2610016A17Rik	0.023277732	1.520070195	up
Dhcr24	0.023277732	1.623161435	down
Sh3md4	0.023277732	1.657510042	up
Rnf121	0.023277732	1.608963132	down
Klhl26	0.023277732	1.548696876	up
Prdx5	0.023282845	2.447168112	down
Ppa2	0.023296118	1.507208467	down
Khk	0.023296118	1.691097617	up
Tia1	0.023301015	1.673606753	up
Pcdh21	0.023301015	2.120504856	up
Dync1i1	0.023307001	1.557070732	up
H2-Q7	0.023314515	1.657483101	up
Slc29a3	0.023327082	1.84835577	up
Tsc22d1	0.023327265	2.271643877	up
Fbxo3	0.023327265	2.7089746	down
5330439J01Rik	0.023348827	2.089283228	down
Ccdc65	0.023368228	1.611338258	up
Kcnq5	0.02338283	2.125079393	down
Eif4enif1	0.02338283	1.791041136	up
5430406J06Rik	0.02339516	1.775479794	down
Tmprss4	0.023409253	1.544905782	up
Col11a1	0.023426889	2.004609585	down
1110018J18Rik	0.023446994	1.536654949	up
1110018J23Rik	0.02344949	1.574401498	up
LOC384104	0.023465941	2.113748312	down
MGC41689	0.023536885	1.823236585	up
1700019G17Rik	0.023537116	1.714512825	up
Dnajc13	0.023544507	2.327007532	up
C80913	0.023546703	1.702159762	down
Akap2	0.023546703	1.625469327	up
6230425C21Rik	0.023557546	1.824457288	up
Ppargc1b	0.023582641	1.516608834	up
Mapre3	0.023589345	1.673989296	down
2810021B07Rik	0.023624774	1.736855626	down

LOC271505	0.023631707	3.971617699	down
Fahd2a	0.023648931	1.603322625	up
Gemin7	0.023668733	1.771687985	up
Sel1l	0.023694467	3.993265629	down
C130057N11Rik	0.023694467	1.556792498	up
1810058I24Rik	0.023733042	3.318252325	down
BC008163	0.023749603	2.872507334	down
4933401P20Rik	0.023750432	1.675714493	up
Trpm5	0.023750432	1.525869846	up
LOC386270	0.023750432	1.92858398	down
Tmem9b	0.023750432	2.020874262	up
Ankrd6	0.023788381	1.752595902	up
Zfp212	0.023788381	1.551650167	up
2810021O14Rik	0.02379065	1.523215175	up
Zpld1	0.023792507	1.533877492	up
LOC223653	0.023792507	1.754093289	up
Mdm4	0.023792507	1.684967756	up
Acy3	0.023806425	1.578506351	up
Zfp275	0.023806425	1.649734855	up
A130047F11Rik	0.023806425	1.588010311	down
Pde3a	0.023811156	1.607645631	up
Golgb1	0.023812143	1.563291907	up
9130213B05Rik	0.023820758	1.559818626	up
Myl9	0.023820916	2.611768007	up
Ate1	0.023823107	1.598100185	up
6330415B21Rik	0.023832373	1.670081615	down
Mizf	0.02383608	1.876211047	up
Atp6ap2	0.023844006	1.978302479	up
Ubox5	0.023857005	1.654751301	up
Pcdhga10	0.023868516	1.606812477	up
Ptbp1	0.023880256	1.554322958	up
Rnf135	0.023880256	1.578371644	up
Epb4.9	0.023900524	1.571344495	up
Ehd1	0.023900524	1.636761189	up
Sumo3	0.023900524	2.002801895	up
OTTMUSG00000000421	0.023903871	1.591713429	up
Lamp1	0.023907052	1.815142393	up
Npr3	0.023911178	1.580506921	up
LOC381525	0.023911178	1.576633692	up
Mrpl15	0.023911178	1.657515287	up
LOC100046608	0.023925681	1.590238571	up
Snx14	0.023932144	1.564789772	up
Odf3l1	0.023945196	1.682940722	up
Agfg1	0.023953376	1.663761973	up
Igfbpl1	0.023953376	1.91601491	down
Abcf1	0.023955768	1.522230983	up
Ltbp2	0.024025012	1.659017682	up
Trdmt1	0.024051283	1.582988262	up

Popdc3	0.024061745	1.537994146	up
Slc25a2	0.024095271	1.589147568	up
Plec1	0.024100356	1.604737997	up
Nol4	0.0241067	3.287555695	down
1700008H02Rik	0.024122192	2.490914345	down
1810063B05Rik	0.024122192	1.856878996	down
2900024O10Rik	0.024148388	1.703561902	up
Nagpa	0.024164081	1.571860433	up
Mbtps2	0.024165578	1.51530087	up
2310016C08Rik	0.024176354	1.670640349	up
Hrmt1l2	0.024176354	2.043538094	up
Apoc2	0.024176354	1.662402511	up
2810405K02Rik	0.024176354	1.770153046	up
Aamp	0.024186686	1.546469212	down
Adsl	0.024186686	1.793107987	up
Eral1	0.024186686	1.823162675	up
C77080	0.024186686	1.512732506	up
Abhd10	0.024186686	2.023592711	up
Fam120b	0.024208803	1.600319147	down
Actn3	0.024220793	1.540004253	up
Ipmk	0.024220895	1.672956586	up
Hspa9	0.024249226	1.525951982	up
LOC676974	0.024260305	1.531931639	up
Amotl1	0.024260338	1.862997174	down
Tmem161b	0.024260338	2.033568144	down
Tsn	0.024260338	4.40406847	down
Brd8	0.024288654	1.827092886	up
Cnp	0.024289805	1.800862432	up
Slc25a15	0.024290664	1.734380007	up
Tgs1	0.024290664	2.016076088	down
Copg2as2	0.024302702	1.893936753	down
Dusp22	0.02432132	1.761821628	up
Pbx2	0.024321504	1.587426662	up
Clint1	0.024321504	2.272943258	down
Tm9sf2	0.024328524	2.36627388	down
Pde6d	0.024338225	1.723430872	up
Ttpal	0.024338225	1.538209915	up
Trim2	0.024366964	3.839619875	down
Clic6	0.024366964	1.562488794	up
Myst3	0.024371266	1.679836869	down
Qars	0.024401613	1.783143997	up
Dcun1d4	0.024401613	1.65478456	up
Nup54	0.024405032	1.577212215	up
LOC385256	0.024421034	1.634436607	up
Ppid	0.024446977	1.511601687	down
Tipin	0.024446977	2.504835129	down
Pik3ip1	0.024446977	1.815788627	up
Art4	0.024448287	1.582185507	up

Sar1a	0.024453528	1.637433529	up
BC027344	0.024459502	1.884385228	up
Mtap	0.024463318	2.11685133	up
LOC381255	0.024497351	1.722620368	up
Pde2a	0.024540091	1.623135567	up
Tnip2	0.024565756	1.520159125	up
5430440L12Rik	0.024565756	2.565839052	down
Rnaset2	0.024571283	1.512865543	up
Sh3gl2	0.024594786	1.622609735	up
BC021438	0.024594786	1.686490417	up
Smad6	0.024594786	1.611704707	up
Dock1	0.024606509	1.668456078	up
Rnmtl1	0.024610108	1.867081404	up
Coq6	0.024613494	1.565078378	up
4930528A17Rik	0.024613494	1.534745932	up
Acadl	0.024641054	2.07760644	up
2600001B17Rik	0.024641054	2.794253111	up
Gpr85	0.024641054	2.229593277	down
BC004728	0.024675852	1.708168387	up
scl0002720.1_68	0.024678361	1.53012836	up
Bex4	0.024678361	1.611734152	up
Atp9a	0.024678361	1.845288873	up
Zdhhc13	0.024678361	1.633460999	up
Dlk2	0.02472619	1.664902329	up
Rbbp7	0.024752462	2.38526535	up
Tarsl2	0.024752462	1.836077333	up
Robo4	0.024752462	1.980917573	up
Cenpk	0.024752462	2.191295385	down
St5	0.024752462	1.883800745	up
Pin4	0.02477007	3.59882474	down
Exosc2	0.02477007	1.750701308	up
Ndrl	0.024785008	1.729287267	up
EG667410	0.024785008	1.553994179	up
Scp2	0.024789129	1.795739889	up
Dsp	0.024789129	3.034396887	up
A730058G16Rik	0.024789129	1.501344919	down
Phf12	0.024821799	1.738739848	up
Csnk2a1-rs4	0.024823898	1.59814918	down
Lgals3bp	0.024831537	1.609948516	up
Ddx19b	0.024833541	1.514054775	up
Barx1	0.024833541	1.983679891	up
Mamdc4	0.024835633	1.786732793	up
Lrrn1	0.024838448	1.575416923	down
Zfp239	0.024850052	1.93827045	up
Kist	0.024850916	1.575038075	up
Mat2b	0.024903977	1.663796783	up
Rara	0.024949867	1.639087081	up
LOC100044190	0.024953106	1.638198495	up

Rhoj	0.02495648	1.530031443	up
Pet112l	0.024982216	1.854303718	up
scl0004175.1_57	0.024997147	1.761688471	up
Crk	0.025018156	1.630154014	up
Slc35e3	0.025018156	2.007858038	up
Rnaseh2c	0.025030997	1.73447597	up
Myl9	0.025097314	1.679783702	up
Mcph1	0.025101082	1.53249979	up
2610528B01Rik	0.025101082	1.738859057	down
Def6	0.025114	1.623725414	up
Ascc3	0.025126007	1.722253919	up
Pcdhb17	0.025128463	1.805248141	down
Wnt3	0.025136031	1.793203712	up
Lpar3	0.025136031	1.631058812	up
Cxxc4	0.025136087	1.547854066	down
Hsf2	0.025145717	2.529181719	down
Etv4	0.025146311	1.609756589	up
Arpc1a	0.025154928	1.645087481	up
Tmem164	0.025163205	1.568869829	up
Nedd4b	0.025164926	1.538226128	down
Kctd10	0.025174495	1.677629113	up
9130020K20Rik	0.025177149	2.746968508	down
Fbxo27	0.025178876	1.653633595	up
Slc10a7	0.025231214	1.626215696	up
Chi3l1	0.025231214	1.596438646	down
Freq	0.025231214	1.559261322	up
Mfsd10	0.025231883	1.553831816	up
Ankrd50	0.025239116	1.767619491	up
Nt5dc3	0.025239116	1.59585011	up
Thnsl1	0.025239116	1.586515665	up
Ddx10	0.025239116	1.509826541	up
A630007B06Rik	0.025241843	1.511982322	up
LOC100043906	0.025313072	4.887526989	down
Sra1	0.025313072	1.999520302	up
6030446N20Rik	0.025313072	1.531085014	up
Akap2	0.025333304	1.990472913	up
Dnajc19	0.025349712	1.51751399	up
Rapgef4	0.025403667	1.735148907	up
Plekhg2	0.025416916	1.719652534	up
Sema4f	0.025416916	1.563287735	up
LOC381105	0.025416916	2.001013279	down
Hecw1	0.025416916	1.857788563	down
Sgk3	0.025429606	1.639897585	up
Col6a2	0.025434684	1.888553977	up
Cart	0.02544588	1.606099963	up
Cdh5	0.025475081	2.108512402	up
4732415M23Rik	0.025485471	1.595445871	up
Rabggtb	0.025492046	1.670616269	up

LOC385959	0.025497114	2.51709652	down
Dok5	0.025520928	1.511301041	up
Tmed4	0.025520928	1.571489215	up
LOC329575	0.025520928	2.877915621	down
Rbmxrt	0.025520928	1.749711752	down
Ints9	0.025538733	1.551462174	up
Pigb	0.025543677	1.531557202	up
Macrod2	0.025543677	1.518496275	up
Chmp6	0.025597274	1.510669947	up
D430004H20Rik	0.025597274	1.594581246	up
Zfp322a	0.025605898	3.740749121	down
Mtbp	0.025626093	1.509504795	up
Tyw1	0.025656398	1.565536022	up
D630011N09Rik	0.025687009	1.539307118	up
2310047C04Rik	0.025691306	2.365804195	up
A930024F17Rik	0.025711713	1.55544889	up
4631422C13Rik	0.025711713	1.520938516	up
Rhbdl7	0.025711713	1.599536896	up
Tmem134	0.025711713	1.792229056	up
Accn3	0.025711713	1.692849755	up
Spg20	0.025711713	1.628908157	up
Lias	0.025718255	1.530318379	down
Pi4k2b	0.025720315	1.549804926	up
9430001M03Rik	0.025742406	1.604554176	up
Rbm12	0.025754886	1.520947218	down
3830408P04Rik	0.025754886	1.718554735	up
Itprip	0.025754886	1.958350062	up
Wbscr27	0.025754886	1.643675447	up
Foxn4	0.025754886	1.533078671	up
Cldn6	0.025774211	2.557539463	up
Ywhaz	0.025782675	2.540822029	down
Mtx2	0.025789164	1.664726734	up
Atpaf2	0.025823472	1.623954177	up
Mpdu1	0.025823472	1.503123879	up
Ltbr	0.025823472	1.537944913	up
Lyst	0.025823472	1.550898194	up
Abcg4	0.025823472	1.742854118	up
Bbs7	0.025831513	1.578111529	up
Lingo2	0.025840325	1.55466485	up
Calu	0.025844796	1.555542827	up
Krt7	0.025844796	1.59222734	up
Gpbp1	0.025847625	1.555667758	down
Dleu2	0.02586331	1.781032801	up
Prrt1	0.02586857	1.604215026	up
C030033M19Rik	0.025881652	1.563244462	down
Il20rb	0.025886897	1.710139871	up
Armc10	0.025886897	1.664206386	down
St18	0.025907263	7.775743008	down

Echdc2	0.025907263	1.528123021	up
Ubp1	0.025907263	1.568852067	up
Ptpn1	0.025908377	4.380230427	down
Gnb4	0.025913963	1.696318626	up
C330018D20Rik	0.025941892	1.516558051	up
Tnnc1	0.025961159	1.618737221	up
C030033M12Rik	0.025970075	1.879036427	down
Ifitm2	0.025970075	1.698155522	up
Pou5f1	0.026018338	1.68230176	up
Slit3	0.026018338	1.676242232	up
Mbtps1	0.026021894	2.323622465	up
Scarf1	0.026026081	1.534888983	up
Npepl1	0.026026081	1.636174917	up
Adar	0.026026081	2.49283123	down
Cdc26	0.026026081	2.101592779	down
Mylc2b	0.026026081	1.584136009	down
Snx33	0.02603413	1.812818289	up
Pet112l	0.026034968	1.548941016	up
A230106M20Rik	0.026044812	2.00133419	up
Ap4m1	0.026062368	1.707808256	up
Spin2	0.026075995	1.658396363	up
Ctps2	0.026082985	1.601388574	up
Ndufa11	0.026089588	1.790784597	up
Dach1	0.026124049	1.558477759	up
Elavl4	0.026133543	3.793795586	down
Hoxb3	0.026133543	5.049030304	down
Tbc1d10a	0.026133543	1.881717444	up
D10Ertd322e	0.026133543	1.562223434	up
Rspry1	0.026133543	3.558865786	down
Cdc25b	0.026133543	1.504012466	up
LOC383897	0.026133543	1.587224364	up
Mreg	0.026141685	1.93051517	up
Scnm1	0.026170461	1.553744078	up
B3gnt8	0.026170461	1.587229967	up
B3gat2	0.026185624	1.510452867	up
Cc2d1a	0.026200419	1.768219352	up
Faim	0.026202936	2.411091566	down
Map4k5	0.026226556	1.645509005	up
5930434B04Rik	0.026226556	1.681369305	up
Abhd4	0.026226556	1.838956833	up
Thnsl1	0.026226556	1.570795536	up
Pparbp	0.026226556	1.654484034	up
Ifna1	0.026226556	1.745050073	up
Mcam	0.02622674	2.467596769	up
Zfc3h1	0.026250646	1.54704237	up
Als2cr13	0.026262827	3.567935944	down
Pmp22	0.026262827	1.897400498	up
Nphp1	0.026262827	2.021710157	up

Haghl	0.026270641	1.563119054	up
Txndc11	0.026275797	1.518350482	up
Zdhhc24	0.026277332	1.620695353	up
Itfg3	0.026287865	1.783935905	up
Gdf5	0.026287865	1.501856208	up
Emcn	0.026325403	1.786376	up
Ift52	0.026327802	1.582767367	up
4632434I11Rik	0.026351381	1.592755079	up
Nrd1	0.026353965	1.675454736	up
Lass5	0.026353965	1.966161013	up
LOC100047915	0.026358072	1.863041878	down
Lonrf1	0.026361981	1.627084255	up
Gdap10	0.026367342	1.916637421	down
Tcf3	0.026367342	1.614472389	up
Nme2	0.026367342	1.52468586	up
Acss1	0.026367342	1.537099481	down
Ppbp	0.026367342	3.029294968	down
Mbnl1	0.026367342	1.676026583	down
Lypd1	0.026370244	2.41830349	up
Polr1d	0.026381962	1.628500342	up
LOC383836	0.026393026	1.60314846	up
LOC100047427	0.026393026	1.639779925	up
Emilin2	0.026393026	1.836941123	up
D11Bwg0414e	0.02640174	1.660576701	up
Prmt8	0.026403844	5.58437109	down
Tgm2	0.026413081	1.707795024	down
2700055A20Rik	0.026415721	4.811782837	down
Sec31l1	0.026417017	1.637033701	up
100041294	0.026417017	1.742833376	up
Ralyl	0.026419994	1.511092424	up
2810039B14Rik	0.026430406	1.559926748	up
Ptprd	0.026440425	1.909022689	down
2410004N11Rik	0.026462166	1.872613311	up
Arl8b	0.026463216	1.552140713	up
LOC545472	0.026497282	2.392857552	down
Cyp2s1	0.026497282	1.530257344	up
Itga11	0.026497282	1.655565023	up
a2ld1	0.026497282	1.657340646	up
Eif2ak2	0.026497282	2.079792976	up
Wdr51b	0.026510926	1.612763643	up
Cd59a	0.026510926	1.830264568	up
Chrna1	0.026525114	1.568944335	up
6330406I15Rik	0.026531929	1.596174359	up
Bcl9l	0.026561642	1.735089421	down
Mknk1	0.026579022	1.606302977	up
Rps9	0.026588827	1.560705304	up
Txndc11	0.026603427	1.503304601	up
D2Bwg1423e	0.026627649	1.615328789	up

Mfng	0.026661895	4.273496151	down
Maf	0.026681554	1.702198863	up
Nufip2	0.02671833	1.509658575	down
Zfp212	0.02671833	1.538157225	up
Ctnna2	0.02671833	3.798143148	down
Adamts3	0.02671833	1.551538229	up
D830014B20Rik	0.026737785	1.852300167	up
Mertk	0.026746687	2.09731698	down
LOC382465	0.026746687	1.600940466	up
Wars2	0.026746687	1.775277972	down
LOC674004	0.02676554	4.925053597	down
Ptbp1	0.026770931	1.555795074	up
Pdia5	0.026770931	1.963066936	up
Apg5l	0.026784021	1.504658341	up
Tug1	0.026786363	2.389960527	up
Rab8a	0.026787756	1.678788185	up
P4hb	0.026812084	1.65703547	up
Rab11fip5	0.02682505	1.798027277	up
Ercc8	0.026826236	1.857405901	up
Rgs7	0.026829358	1.654751658	up
Gstm2	0.026842535	1.79594624	up
Slc30a10	0.026847303	1.596513391	up
Glrx2	0.026854638	3.083699942	down
4933433P14Rik	0.026855594	1.562795878	up
Sfxn3	0.026855594	1.560568929	up
Clspn	0.026869539	1.504998803	down
S100pbp	0.026869539	1.500028968	up
Asph	0.026869539	1.519088864	up
Stx8	0.026869539	1.527396083	up
2900073G15Rik	0.026869539	1.808086157	up
Timm44	0.026869539	1.574966073	up
Fam122b	0.026879098	1.590674639	up
Prkg2	0.026879098	2.101173639	down
Ogfod1	0.026921377	1.606532216	up
Abhd11	0.026935844	1.530041218	up
Afap1	0.026935866	1.59734416	up
Meis2	0.026946327	1.503676415	down
Tmem38b	0.026946327	1.543681145	up
Sln	0.026946327	1.605552316	up
Ube2d1	0.026946327	1.53482914	up
Zfp521	0.026946327	1.588275909	up
Polm	0.026946327	1.638597131	up
Snx6	0.026946327	1.84070313	up
2610024G14Rik	0.026946327	1.672836423	up
Rps6kl1	0.026946327	1.659649253	up
3110035E14Rik	0.026950484	1.558245421	down
Krt17	0.026951646	2.959543943	up
Pcm1	0.026964886	3.300732374	down

Zfp113	0.02700972	2.708885908	down
Glt8d1	0.027012231	1.665601492	up
Chrna4	0.027013289	4.589321137	down
Dtnb	0.027023206	1.528187871	up
LOC100045644	0.027036538	1.736202836	up
4833420G11Rik	0.027056256	1.55401969	up
Ppnr	0.02705715	1.9111408	down
Map3k7	0.027112283	1.872915387	down
Fbxw7	0.027112544	1.574082971	down
A2bp1	0.027112544	1.828560233	down
Faah	0.02712548	1.591732025	up
Htra3	0.027149113	1.771133304	up
Rilpl1	0.027229013	1.512639761	up
Spin1	0.027229013	2.070182562	up
Ptcd3	0.02725338	1.683154821	down
F11r	0.027272388	1.647493362	up
2010001H16Rik	0.027284723	1.550769568	up
Slc9a6	0.027284723	1.606795669	up
LOC674427	0.027284723	1.690324664	up
Zadh2	0.027298875	1.724560499	up
Acta2	0.027349493	2.663325548	up
Mtfmt	0.027360603	1.568965554	up
Tax1bp1	0.027360603	1.91344583	down
Capzb	0.027360603	1.712669969	up
Pdlim3	0.027360603	1.562441349	up
BC026590	0.027360603	1.891112447	up
Aebp2	0.027429499	1.723865151	up
Zdhhc1	0.027447149	1.519811034	up
scl0002547.1_9	0.02746857	1.590014458	down
Fam131a	0.027507545	2.021424055	down
Usp52	0.027507644	1.517469764	up
Dscr1	0.027546672	1.523755431	up
Afg3l1	0.027546672	1.888124943	up
Jagn1	0.027591392	1.611032486	up
Dcx	0.027594112	3.346492767	down
Uchl5	0.027613277	1.796848655	down
Cyp27a1	0.027642926	1.542849898	up
Aebp2	0.027657917	1.849905491	up
Ganc	0.027672447	1.544990182	up
1200016D23Rik	0.027687056	1.628642678	up
Fam13c	0.027693685	1.620222092	up
Ncoa4	0.027700804	1.569239855	up
Sin3b	0.027702345	1.504166246	up
Mcm10	0.027702345	1.603678584	up
Dorz1	0.027708128	2.552302599	up
Sim2	0.027708622	1.803269506	down
Ccbl2	0.027722266	1.580010414	up
E230020D15Rik	0.027722266	1.615412712	down

Fbxw4	0.027734825	1.662856817	up
Ppp1r14b	0.027737975	1.524020553	down
5730409K12Rik	0.027737975	1.809856296	up
4632404H12Rik	0.027787799	1.588802099	up
Mpg	0.027799141	1.551888585	up
Mrpl18	0.02780522	2.807289124	down
Lamp2	0.02783915	1.607890725	up
Trim69	0.027860686	1.613491774	up
Senp5	0.027873479	1.628500462	up
Timm8a1	0.027875032	1.638612986	down
Osbp	0.027882962	2.718029976	down
Mxd4	0.027882962	1.636723161	up
Myo1b	0.027882962	2.869684696	down
Arl10c	0.027882962	1.541435838	up
Ric8b	0.027885396	2.201886177	down
4931406P16Rik	0.027891882	1.822182775	up
Rpap3	0.027906688	1.705803156	up
2610024H22Rik	0.027939865	2.055477858	down
Camk2d	0.027955076	1.716328144	up
Gm1008	0.027967297	1.513813376	up
Seh1l	0.027967297	1.811846376	up
Tmem101	0.028001213	1.706711412	up
Igk-V1	0.028001213	1.65182507	up
Akr1c19	0.028001213	1.610110879	up
Clptm1l	0.028016031	1.930399776	up
Zfp312	0.028045975	1.586424828	up
0610031J06Rik	0.028061399	1.720502973	up
2310040B03Rik	0.028064515	1.511780143	up
LOC100046841	0.028082036	4.019485951	down
Foxp2	0.028164564	1.624937177	up
Wdr40c	0.028182847	1.794133663	down
Orai3	0.028195217	1.530039907	up
Trim37	0.028239388	2.207437515	down
Uncx	0.028250525	1.661578298	down
E430002G05Rik	0.028250525	1.525925756	up
Mxd4	0.02827296	1.524269223	up
Mett11d1	0.02827296	2.105715752	up
Arhgap22	0.028290734	1.752790451	up
A730008L03Rik	0.028290734	1.877373695	up
1700020O03Rik	0.028293982	2.101079941	down
Aim1l	0.028293982	1.70334363	up
B230365C01Rik	0.028298996	2.474878073	down
2810484G07Rik	0.028372543	2.946617842	down
Olfr538	0.028377615	1.737084508	up
Lrrc8	0.028377615	1.525708199	up
Adamts12	0.028402144	1.665921211	up
B930095G15Rik	0.028402144	1.635040402	down
scl0001534.1_16	0.028427808	1.860778689	up

Ing4	0.028427808	1.596772075	up
Sidt2	0.02843078	1.842948318	up
AI464131	0.028441729	1.597222328	up
Fmnl3	0.028441729	1.78928411	up
Gjb6	0.028444422	2.173983812	up
Rgag4	0.028461123	1.550311446	up
6330439K17Rik	0.028471073	1.916797161	down
Mtrf1l	0.028473882	3.169497013	down
Pgp	0.028492704	1.510418057	down
Grb7	0.028551078	1.983660221	up
Sfrs16	0.028590983	1.658891201	down
9330134C04Rik	0.028590983	1.581057668	up
BC023151	0.028594459	1.51757896	up
Mff	0.028621918	1.713010311	up
Serpini1	0.028621918	1.629582048	down
Slc38a3	0.028625395	1.742062211	up
Pkp4	0.028657824	2.417450666	up
Gm114	0.028665807	1.667310357	up
Tshz3	0.028670009	1.775392175	up
Mcm6	0.028670009	11.24821663	down
Pdha1	0.028678687	1.565930843	up
Rap2c	0.028678687	1.886301637	down
LOC676640	0.028678687	1.958680272	down
Srr	0.028696263	1.622927189	down
Cacnb3	0.028703176	1.682098389	up
2700094K13Rik	0.028703176	1.525568724	down
2810417K24Rik	0.028703176	1.687769175	down
Pum1	0.0287535	1.548317313	down
Refbp2	0.0287535	2.306913853	down
LOC100047184	0.028754348	1.614117384	down
LOC100047173	0.028808892	1.571497202	up
Oc90	0.028808892	4.068250179	up
Taf12	0.028810173	1.681245565	up
Mcart6	0.028845165	1.543673396	up
Pcnx	0.028860036	1.622799873	up
0610030E20Rik	0.028867669	1.571699858	down
Gstm2	0.028901044	1.609073877	up
Plekhf1	0.028949276	1.631626129	up
Mpdz	0.028957715	1.740692735	up
Smg6	0.028957715	1.917463899	up
Cd248	0.028963585	1.562383294	up
Mtap7	0.028974792	1.66708076	up
Cib2	0.028988212	1.696259022	up
Txndc4	0.02900986	2.04467535	down
0610031J06Rik	0.029018952	1.570447445	up
Akp2	0.029024579	1.531524062	up
Mtmr14	0.029024579	1.545539022	up
Mrg1	0.029024579	1.733362079	down

Prmt2	0.029036792	1.519814611	up
Notch3	0.029068582	1.56441009	up
Ubqln1	0.029087836	1.717883945	up
Insl6	0.029089032	1.516557336	up
Lonrf2	0.029110663	1.598227024	up
Wdr43	0.02913104	1.884290338	up
A430024H01Rik	0.02913104	1.626386285	up
1700085B03Rik	0.029166685	1.592478037	up
Zdhhc16	0.029187497	1.628306866	up
Anks3	0.029209739	1.694904804	up
Mfsd10	0.029255815	1.567563772	up
9230112G11Rik	0.029256797	1.514317751	down
Med9	0.029275071	1.729164958	up
D11Wsu99e	0.029311631	1.70733428	up
Scoc	0.029328078	3.418934822	down
Zfp364	0.029344112	1.855145931	down
Thbs3	0.029344924	1.543448329	up
Hspb1	0.029363191	1.815460086	up
Acta2	0.029363191	1.711580515	up
Ethe1	0.029363191	1.768212557	up
Aig1	0.029369617	1.540420175	up
LOC381200	0.029395932	1.582753539	up
9330152L17	0.029408539	1.907881737	down
Prim1	0.02944392	2.214730978	down
Bcas1	0.029475207	1.657806397	down
2310016C16Rik	0.029531336	1.718999267	up
Atp13a3	0.029543078	1.529669285	up
Papss2	0.029543078	1.707736969	down
Mcam	0.029544398	1.807081103	up
Ensa	0.029572913	1.713445067	up
Lmna	0.029575888	1.600558281	up
A930029B02Rik	0.029606462	1.538309097	up
Pnpla2	0.029620072	1.741575718	up
2700033B16Rik	0.029639551	1.681651235	up
Senp6	0.029646043	1.570310593	up
Fzd8	0.029646043	1.668245792	up
Zfp664	0.029646043	1.841240764	up
4732442E24Rik	0.029647617	1.538217902	up
Heatr5a	0.029654384	1.947371602	down
LOC208166	0.029660752	1.696879864	up
Mkrn1	0.029687297	1.699000955	down
Mapk9	0.029738369	1.527712703	up
2310007H09Rik	0.029745437	1.540451527	up
Acacb	0.029752836	1.592097521	up
Myo9b	0.029752836	1.646127701	up
Foxa3	0.029752836	1.894910812	down
Ifi30	0.029768894	1.513687849	up
Ltbp4	0.02977672	2.141326904	up

Ilvbl	0.029818041	2.052839518	up
Cdh5	0.029818041	1.843039036	up
Cd97	0.0298404	1.604313612	up
Mtbp	0.029844366	1.640318275	up
Gabarapl2	0.029846137	1.961745739	down
Afmid	0.029884238	1.736949205	up
Dbx1	0.029888662	1.643950105	down
Mid2	0.029888662	1.558360934	up
Frzb	0.029888662	1.877044559	down
Dicer1	0.029889222	2.214615822	down
Ddx28	0.02989126	1.641943336	up
Skil	0.02994547	1.511991978	down
Mfsd9	0.029947968	1.503466129	up
Anxa3	0.02996574	2.279321671	up
Rpl10a	0.029969674	2.108971834	down
Rabl2a	0.029986566	1.631922603	up
Mbtps1	0.03003272	1.896953344	up
V00785_Ig_heavy_const	0.03003272	1.58356154	up
Aco1	0.030056078	1.642617703	down
Smarca2	0.03007333	1.595980644	up
Trappc1	0.030094326	1.562215686	up
Cxcl14	0.030094326	1.5744313	up
Gpx7	0.030105149	1.595425248	up
Hmga2	0.030112024	2.009763241	down
Mtrf1	0.030113114	1.602805853	up
Asb9	0.030119075	1.501103878	up
Fip1l1	0.030146644	3.076507092	down
Opa1	0.030152256	1.507286549	up
Etfb	0.030229777	1.889317751	up
Stub1	0.030252883	1.602163792	up
Ctsa	0.030253775	1.51715827	up
A130038H09Rik	0.030253775	1.535893798	up
scl000292.1_12	0.030303821	1.765047669	up
Map4k2	0.030307576	1.85468936	up
Gmppa	0.030309053	1.682019949	up
Nrxn1	0.030316748	1.507935166	up
Tmem120b	0.030339377	1.507872343	up
Asph	0.030348077	1.633422256	up
Mcm3	0.030363364	1.743076801	up
Ssbp2	0.030363364	1.765969753	up
Nt5c3l	0.03036668	1.889875889	up
Gabbr1	0.030368153	1.589981437	up
Rplp0	0.030368153	2.356648207	down
A630084D02Rik	0.030382408	2.963712692	down
1600002H07Rik	0.03042924	1.976994515	down
AI837181	0.03042924	1.509674668	down
Oaz2	0.030499885	1.513807893	up
Tbx15	0.030504221	1.590451598	up

Abi1	0.030504221	1.873409629	down
Iars2	0.030504221	1.589694977	up
Nrp2	0.030513294	1.503936052	up
Rnf25	0.030513294	1.867015362	up
Rbms3	0.030513294	2.295167208	down
LOC383860	0.030513294	1.764351845	up
LOC380888	0.030551158	1.667047262	up
Ypel4	0.0305607	1.553146243	up
Lgtn	0.0305692	1.84411788	up
Zfp263	0.03057191	3.186406136	down
Eya1	0.030628974	1.579699397	up
1110021L09Rik	0.030671848	2.676906824	down
Decr2	0.030671848	1.764048815	up
Ncoa6	0.030671848	1.7943151	up
Arid1a	0.030740051	1.668958545	down
Acta2	0.030757951	2.90301919	up
Smox	0.03077961	1.851334691	up
Trex2	0.030830206	1.538734675	up
Osbpl8	0.030830698	1.515735984	up
Asah2	0.030835558	1.529835701	up
Usp7	0.030843636	1.533867955	up
Tulp1	0.030843636	1.618751407	up
Nucb1	0.030851699	1.576314688	up
Ccdc98	0.030851699	1.53328681	down
9330151L19Rik	0.030873148	1.807281137	up
Fancc	0.030883692	1.574878931	up
Cldn5	0.030898245	2.271008253	up
Hbp1	0.03089829	1.503505707	up
Glyctk	0.03089829	1.877753735	up
Atp6ap2	0.03089829	1.710296631	up
Hsd17b4	0.030900534	1.559193134	up
Evi1	0.030902976	1.77359283	up
Tpm4	0.030904748	1.678726554	up
C230060D12Rik	0.030922763	1.507926583	up
Slc44a2	0.03095465	1.523117781	up
Dek	0.031024393	2.914137125	down
B230322F03Rik	0.031049334	1.533273697	up
Cnga1	0.031052679	1.521505237	up
Atox1	0.031058217	1.594394684	up
Ddhd1	0.031058217	1.912640452	up
LOC386021	0.031058217	1.901959539	down
Caskin1	0.031066457	1.753399253	up
Clip2	0.031073825	1.578637362	down
Lypd3	0.031084012	1.906809449	up
Pop7	0.031108018	1.62401104	up
Ndufs2	0.031108018	1.610663772	up
AI851790	0.031115254	1.57173574	up
Cd63	0.031120822	1.798962355	up

Vapb	0.031120822	1.67388463	up
Smurf1	0.031182999	1.509688258	up
EG624138	0.031205697	1.560395122	up
St6galnac4	0.031209065	1.592423916	up
Fbxl10	0.031219957	1.52978003	up
LOC270491	0.031219957	1.547472596	up
Acy1	0.031220693	1.551064611	down
Cdc25a	0.031263851	1.586982369	down
Vps41	0.031263851	1.923785567	up
Dgkh	0.031263851	1.518365383	up
Egln1	0.031292979	1.570744515	up
Psap	0.031294793	1.519998908	up
Nudt19	0.031304129	1.604734182	up
2810002D19Rik	0.031329934	1.994616389	down
EG434280	0.03133573	1.868241072	up
Fgfr2	0.031341277	1.534162521	down
3110001A13Rik	0.03136462	1.868430734	up
Nme7	0.031372234	4.946968556	down
Papd4	0.031372234	1.833988071	up
Micall2	0.031372234	1.692086935	up
Prosc	0.031372476	1.551361799	up
8430415N23Rik	0.031402595	1.786611915	down
9530077C05Rik	0.031433292	1.731611013	up
Rnpc3	0.031433292	1.966447353	down
Pdia3	0.031437375	1.771489024	up
Slmo2	0.031446952	1.659236312	down
Tmem199	0.031469733	1.627209306	down
scl00238693.1_37	0.031506244	1.564427137	up
Trpc3	0.031538252	1.71190989	up
Gm962	0.031586733	1.549809814	up
Crim2	0.031595808	1.725671172	up
Pex19	0.031607453	2.831524611	down
Tmem32	0.031651683	2.048749685	down
Golph3l	0.031651683	1.944182396	down
Wwtr1	0.031651683	1.71940124	up
Rusc1	0.031651683	1.652538657	up
Zcchc14	0.031655647	1.690110087	up
4933421E11Rik	0.031690516	1.576277137	up
Mrps22	0.031711131	1.586488128	up
LOC100043822	0.031720564	1.976833701	down
3110056O03Rik	0.031734686	1.537610412	up
Cox6b1	0.031750556	1.503103733	up
Shq1	0.031750556	1.644196034	up
Nap1l5	0.031769957	2.706310034	down
Zic3	0.031785309	2.627598286	down
Gnb1	0.031785309	1.949632049	up
Rps26	0.031785309	1.664353252	up
Abcd4	0.031785309	1.645703673	up

Pfdn4	0.031786639	4.021124363	down
Cnot4	0.031786639	2.910654545	down
5430437P03Rik	0.031786639	1.62628293	up
Id2	0.031794488	4.620919704	down
Trim45	0.03182929	1.639558554	up
Gck	0.031830173	1.524319291	up
Usp29	0.031834714	1.547872663	up
Mtus1	0.031834714	1.759315252	down
Cenpj	0.031853035	2.299944878	down
Lmx1a	0.031853035	1.600459933	up
Srebf2	0.031859011	1.6712358	up
Uchl5	0.031911381	2.00066638	down
Ehbp1l1	0.031951223	1.636701345	up
Rtl1	0.031957787	1.698897958	up
Tiam1	0.031993203	1.507913351	up
1110005A23Rik	0.031995587	5.427640915	down
1700040I03Rik	0.031995587	1.502797604	up
Zbtb33	0.032002442	1.975539208	down
A930038D23Rik	0.032022722	1.605605245	up
Akr1a4	0.032032557	1.564214706	up
Ccndbp1	0.032050192	1.562249661	up
2310043J07Rik	0.032063741	1.500001788	up
Aldh2	0.032073207	1.549734712	up
LOC382096	0.032120287	3.824896336	down
Rhod	0.03213818	1.561836958	up
Mdh2	0.032179292	2.325920582	down
Rab3d	0.032286231	1.617559314	up
Sez6l2	0.032286231	1.523634195	up
Mpeg1	0.032302115	1.955189943	down
LOC100044008	0.03230812	3.193525314	down
Zfp236	0.03230812	1.918269157	down
1500003O22Rik	0.032315016	2.810074091	down
Rabggta	0.032369256	1.836091638	up
Sema3f	0.032382228	1.584920287	up
Ikbkg	0.032408483	1.664376855	up
Bid	0.032438874	1.51270771	up
Slc10a7	0.032438874	1.511489272	up
Tnc	0.032438874	1.676503539	up
Hnrnpf	0.032438874	1.809911728	up
Znf512b	0.03245138	1.577176929	up
Rab31	0.032474648	1.548935175	up
Plp1	0.032486331	2.875418901	up
Snrk	0.032491464	1.784790635	up
Wars	0.032500491	1.604062557	up
Smox	0.032506835	1.748138428	up
Eya1	0.032506835	1.542437673	up
2900002G04Rik	0.032525931	1.506278396	up
Prdx2	0.032525931	1.892920137	down

A930005L06Rik	0.032553282	1.547621489	up
Plekhg3	0.032553282	1.756416917	up
Ccdc111	0.032646313	1.732067227	up
Zbtb8b	0.032715716	1.73449862	up
Fbxo18	0.032719791	2.497284174	down
9430065L19Rik	0.032753333	1.579486847	down
BC002199	0.03277047	1.544176102	up
2610209M04Rik	0.032800529	2.104150534	down
4732418C07Rik	0.032800529	1.741468072	up
Pnpla7	0.032827478	1.582681418	up
1110008L16Rik	0.03290125	1.641346216	up
Pbx1	0.032902844	1.512648821	up
LOC212963	0.032902844	1.824072242	up
Sema3f	0.032906845	1.528925896	up
Gbx2	0.032907192	1.973621488	down
LOC381986	0.032927413	1.534062862	up
Cog1	0.032938287	1.938012719	up
Ccdc6	0.033014499	3.392610073	down
Ppox	0.033032745	1.538250327	up
Tmem168	0.033033256	1.642778516	up
Lman1	0.033042196	2.043184996	down
Tbx1	0.033042196	1.628616214	up
Frs2	0.033042196	1.629254341	up
Sumf2	0.033042196	1.79304421	up
Uba52	0.033042196	1.668511868	down
Pgm5	0.033044465	1.562931538	up
Rabep2	0.033044465	1.599362612	up
Ttc8	0.033058006	1.641539812	up
Ankfy1	0.033060767	1.592227697	up
Sipa1l2	0.033071749	1.600637078	up
9830166G06Rik	0.033083174	1.521059513	up
Gng8	0.033083174	1.809063554	up
Dhrs7b	0.033095315	1.523376942	up
Spag4	0.033095367	1.524130464	up
Amn	0.033102788	1.532149077	up
Zfp607	0.033105552	1.604794741	up
6030405A18Rik	0.033115994	1.609917402	up
2810423A18Rik	0.03314019	1.867673993	down
Rnpep	0.033176668	1.511849284	up
Myo10	0.033186309	1.700320125	up
Eif2s3x	0.033186309	1.568044424	down
Zmpste24	0.033186309	1.504839659	up
Ccdc40	0.033186309	1.533145547	up
Abcf1	0.033228066	1.551396012	up
BC003331	0.033252131	4.39692831	down
Asphd1	0.033330958	1.619994044	up
Tbrg4	0.033331171	1.51945293	up
LOC100040919	0.033331171	1.843165636	up

Trim8	0.033338793	1.562320828	down
Dync1li2	0.033344671	1.710753679	up
Mospd1	0.033344671	2.053426027	down
Cadps	0.033384834	1.605071545	down
LOC214111	0.033391587	1.513002634	up
Gtf3c4	0.033420928	1.56583786	up
Mpv17l	0.033452768	1.500830293	up
4732441L20Rik	0.033453584	1.664955139	up
Btd	0.033466302	1.662049055	up
B930067C07Rik	0.033483211	1.580323219	down
AI593442	0.033489529	1.749751449	up
D230037D09Rik	0.033496514	1.546057224	down
Arf5	0.033505805	1.649889112	down
Fam173a	0.033544868	1.778350115	up
Srbd1	0.033544868	2.141215324	down
Ankrd6	0.033544868	1.827680707	up
Psmg2	0.033544868	1.707842231	up
Rgs10	0.033643276	2.614722729	down
Srpx2	0.033643276	1.712261438	up
Rsph1	0.033681937	1.518684149	up
Tacstd2	0.033731688	2.791885853	up
Lgi3	0.033732034	1.712386608	up
1500010G04Rik	0.033732034	2.609591484	down
Memo1	0.033732034	2.160106421	down
Cpt2	0.033790402	2.466161728	down
Atp11c	0.033835169	1.644250631	up
Bccip	0.033847004	2.243093491	down
3110003A17Rik	0.033847138	1.683123589	down
2900060N12Rik	0.033925876	1.689997673	up
Kpna6	0.03395528	1.569957137	up
Pecam1	0.03396596	1.752328038	up
Rbm41	0.034003049	1.561410546	up
Aldh1a2	0.034003049	2.144871235	up
Aqp1	0.034003049	1.992206931	up
Ubr1	0.034012083	1.774479032	up
Lmcd1	0.034028444	1.525070906	down
Rbm41	0.03406236	1.504709601	up
Sfi1	0.034139879	1.638821125	up
Fads2	0.034148116	1.6228019	up
Itga10	0.034175087	1.845992923	down
Nudcd2	0.034187533	4.132079125	down
Fam149b	0.034199666	1.645675421	up
5330408N05Rik	0.034201585	1.554896951	down
Traf3ip1	0.034206677	1.588404775	up
Tex264	0.034222692	1.683625221	up
Cnr1	0.034222692	1.51412046	up
LOC100046025	0.034238298	1.517375469	up
Snx2	0.034261242	2.244595051	up

LOC100039346	0.034273893	2.467739105	down
Skd3	0.03428087	1.522257328	up
1110008F13Rik	0.034299959	1.902320027	down
LOC100048726	0.03431765	2.183740139	down
Fbxo32	0.034351312	1.528366208	up
1110003P22Rik	0.03435418	2.448050261	down
Apex1	0.034356151	1.767712712	down
2310035C23Rik	0.034406379	2.121509075	up
Map2k6	0.034418076	1.981641054	up
6430411K18Rik	0.034424644	1.839274287	up
Mpz	0.034433715	2.334480763	up
LOC385651	0.034433715	1.64594996	up
9030025P20Rik	0.034433715	1.769819617	up
Ppfia1	0.034433715	1.931825757	up
Tpr	0.034497909	1.73875773	up
2410016O06Rik	0.034497909	1.525724769	up
Mmrn2	0.034514349	1.787147522	up
6030458A17Rik	0.034516972	1.518116236	up
Rasgrp3	0.03452136	1.540870428	up
9030425E11Rik	0.03452136	2.027069807	down
Shroom3	0.03452136	1.598723769	up
Galnt1	0.03452136	1.563987374	up
Creg1	0.03452136	1.725657105	up
Sh3glb1	0.034547325	3.307876825	down
2810017I02Rik	0.034547325	1.576373816	up
Ctdspl2	0.034547325	1.532184362	up
Fahd2a	0.034547325	1.541128039	up
Sidt2	0.034553971	1.82403326	up
AI314180	0.034553971	2.497552633	down
Anp32a	0.03459812	1.64906764	up
Fundc1	0.03459812	1.525100708	up
Asap3	0.03459812	1.577381969	up
Ppp1r12a	0.03459812	2.00460577	down
Lrp12	0.03460487	1.565947413	down
Ddrgk1	0.034610983	1.554594398	up
Mrgpra2	0.034661915	1.728336692	up
D15Mit260	0.0346676	1.707399488	down
Ppm1b	0.0346676	1.824744344	up
2010009J12Rik	0.034670036	1.669093013	down
LOC100047353	0.034672879	1.701561689	up
Pde4d	0.034672879	1.502422571	up
LOC100047260	0.03473866	1.80736053	up
Neto2	0.03473866	2.341834784	down
Ela1	0.03473866	1.588070631	up
Nsun5	0.034756098	1.722378492	up
Fam168a	0.034756098	4.676870346	down
Fnip1	0.034756098	2.116612673	down
Mme	0.03476052	2.044068337	up

Tprkb	0.034775864	1.776126981	up
Mtap2	0.034798268	1.541220665	down
Zmym1	0.0348043	1.587408304	up
Tspan18	0.034861762	1.70498836	up
Ubiad1	0.034890041	1.51559782	up
Apaf1	0.034907445	1.563602924	up
Fibp	0.034907445	1.590910673	up
Cmtm7	0.034934394	2.043777943	up
Ggnbp1	0.0349392	1.717003942	up
Rps6	0.034941975	1.546259165	up
Mterfd3	0.034941975	1.645665646	down
Irx3	0.035015184	1.56071341	up
LOC100044159	0.035020355	1.555500507	up
Tmed4	0.03502826	1.778216362	up
Lrrc14	0.03502826	2.005975008	down
Slc25a37	0.035043716	1.675470829	up
Nme7	0.035147682	3.791926384	down
Slc15a4	0.035147682	1.574252129	up
Mbnl2	0.035147682	2.915253401	down
5730601F06Rik	0.035147682	1.770135999	up
6030429G01Rik	0.035156418	1.525359154	up
Mtif2	0.035210613	1.629735112	down
Rab3a	0.03524873	2.826663971	down
Tdrd3	0.03524873	1.917447448	up
Cdh1	0.0352493	2.49124527	up
9430028L06Rik	0.0352493	1.688407064	down
Krr1	0.035278715	1.633091927	up
Fam129a	0.035286501	1.504360437	up
Tubd1	0.035286501	1.658228636	up
Nfib	0.035310119	2.002110958	down
Flnc	0.035310119	1.799526095	up
Eapp	0.035323944	2.082078219	down
LOC386123	0.035357311	2.196273804	down
2700007P21Rik	0.035377596	1.626297951	up
Prkab1	0.035395283	1.552347422	up
Egln3	0.035409197	1.56449306	down
Pmm1	0.03540922	1.522615314	up
Dact2	0.035442121	1.580868363	up
D1Bwg0212e	0.03546064	1.576556087	up
Emr1	0.035486233	1.973137856	down
Rab5c	0.035495304	3.106201172	down
Bpnt1	0.035508171	1.588183522	up
Cstf2	0.035512004	1.530222535	down
2210021J22Rik	0.03560156	1.667471051	up
Mpped1	0.035615992	1.53749311	up
Epha7	0.035615992	1.909336686	up
Col9a1	0.035713706	1.630497336	down
LOC637353	0.035730612	1.693844676	up

Dtx2	0.035730612	1.573862076	up
Terf2ip	0.035730612	1.532993317	down
Lims1	0.035730612	2.461640596	down
Ppa2	0.035785299	2.438605547	down
Rorb	0.035785299	1.580135465	up
Nme4	0.035823058	1.649847627	up
E2f6	0.035823058	1.755440831	up
Adck5	0.035836484	1.563684106	up
Ercc4	0.035836484	1.837912321	down
Cox11	0.03591612	2.099522114	down
Slc45a3	0.035939794	1.782197952	up
Fah	0.035939794	1.577612996	up
EG630499	0.036019906	1.809317827	up
Zfhx3	0.036063239	1.584697604	down
Pitx2	0.036096945	1.852009296	up
Slc35f5	0.036118332	1.898015976	down
4930579E17Rik	0.0361466	1.65117991	up
Rasl12	0.036150459	1.698372841	up
Mmp2	0.03616247	1.528495312	up
Rabgap1	0.036163107	1.551641464	up
Sf3b2	0.036163107	1.502104759	up
Tsc1	0.036181167	3.493497133	down
4933407C03Rik	0.036242291	5.307877064	down
LOC331139	0.036270168	1.737972379	up
Bag5	0.036311816	1.797182441	down
Prkcq	0.036317211	1.553976417	up
Top3b	0.036322031	1.837668777	up
Dpp6	0.036336653	1.501231313	up
Mbtps1	0.036336653	1.617158175	up
LOC330267	0.036338519	1.662921548	down
BC011248	0.036338519	1.580245972	up
Smc4	0.036338519	2.181024551	down
Cdc42ep4	0.036338519	1.637778044	up
Ptn	0.036350459	1.604098082	up
Ift88	0.0363761	1.605382919	up
LOC381947	0.0363761	1.625520349	up
1700013A01Rik	0.036377128	1.637412667	down
Zfp28	0.036377408	1.759431243	up
2310040A07Rik	0.036377408	1.95963347	down
Eif3eip	0.036377408	1.6255548	up
Crabp2	0.036407188	1.784733057	up
Atf2	0.036436088	1.594232082	up
5330401F18Rik	0.036477301	2.578150988	down
Iqsec3	0.036477856	1.64064467	up
Hip1	0.036479339	2.011731386	down
Skz1-pending	0.036479339	1.556569815	down
Spc25	0.036532957	2.154967547	down
Pdcd2l	0.036534417	1.523199439	down

LOC381801	0.036620941	1.560330629	up
Afg3l1	0.036662836	1.98920095	up
Shcbp1	0.036662836	1.658955574	down
Myl1	0.036662836	1.598588586	up
Plaa	0.036677048	3.3327384	down
Atxn10	0.036677446	1.682330012	down
Glipr2	0.03668325	1.803641558	up
Fbxo36	0.036834471	1.634528279	up
Rbm4b	0.036967732	1.659240723	down
Ppp2r3a	0.036990497	1.553857327	up
Rnf219	0.037019253	2.326157332	down
Hsd3b7	0.037019253	1.61200738	up
Nfib	0.037044019	1.540476203	down
Mrps31	0.037054565	1.585889101	up
Cxcr4	0.037061397	1.727725983	down
Mfge8	0.037061397	1.573057294	up
4930570C03Rik	0.03706396	1.562776804	up
EG633640	0.037071191	1.595697165	up
Col12a1	0.037111823	1.905227065	up
Elp2	0.037161671	1.50028801	down
Fbxo32	0.037161671	1.561836124	up
Atg4d	0.037161671	1.72950089	up
2010007L18Rik	0.037261438	4.025471687	down
Rad51ap1	0.037276078	1.509172916	up
Tusc2	0.037310872	1.56485951	up
Cd97	0.037332725	1.552051663	up
Fbln1	0.037370987	1.885430932	up
Dvl2	0.037400603	1.563253045	up
Clip3	0.037414525	1.519509435	down
Idh3g	0.037431967	1.502750397	up
Mbtd1	0.03743697	2.740798235	down
Jmy	0.03743697	1.639730692	up
Trim41	0.03743697	1.625254154	up
Slc17a5	0.03743697	1.812611342	up
St8sia3	0.03743697	1.785185814	down
Med8	0.03743697	1.757070065	up
Krba1	0.037448753	1.796358585	down
Mll1	0.037448753	2.11644125	down
9330159F19Rik	0.037448753	2.938209534	down
Kcnmb4	0.03748446	1.544195771	up
Lrp11	0.037486024	1.557338238	down
Tspan17	0.037535992	1.854019165	up
Rbbp7	0.037587646	2.172591448	up
4930438D12Rik	0.037587646	1.546450853	up
2310004I03Rik	0.037596233	2.176558971	down
Hbs1l	0.037627328	1.552370071	up
Fbxo9	0.037686452	1.588924289	up
Rgs16	0.037719477	1.761289358	up

Zfp386	0.037725352	1.507188082	up
Hsp105	0.037770353	2.052255392	down
LOC100044576	0.03777628	1.621023655	up
AI553587	0.03777628	1.595452786	up
Wdr33	0.03778382	1.52545476	up
Als2	0.03778382	1.610647678	up
Xpc	0.03781268	1.749504209	down
Smpd4	0.037819598	1.635833502	down
Tssk4	0.037824281	1.535773397	up
Ankra2	0.037847899	1.563921094	up
D930038J03Rik	0.037890673	1.716406703	up
BC024868	0.037898604	1.517678022	up
Cdc123	0.037898604	3.020993233	down
Noxo1	0.03790085	1.587154269	up
Abce1	0.037915066	4.658296108	down
Lrrc1	0.037953708	1.831821084	up
1810055E12Rik	0.037968125	1.563848972	down
Tomm34	0.038007841	1.550408244	up
LOC674707	0.038014811	3.214371204	down
Phf20	0.038014811	4.161310196	down
Atl2	0.038026899	1.616796136	up
Calm2	0.038047757	1.928426981	down
Tmem147	0.038047757	1.614418268	up
Nefm	0.038047757	1.673852325	down
5031436O03Rik	0.038052075	1.541813135	up
Glul	0.038052075	2.877321482	down
Rnf10	0.038052075	1.905380726	up
AU014645	0.038075812	1.80367589	up
Dync1h1	0.038076181	2.124534369	up
Rexo4	0.038116224	1.988161326	up
Pdia6	0.038116224	2.126909971	down
Col9a1	0.038123295	11.82479382	down
9330171B01Rik	0.038143937	1.620916128	down
Elovl1	0.03818078	1.882636309	up
Zfp39	0.038184948	1.590619206	up
C330002I19Rik	0.038184948	1.841724038	down
9430077D24Rik	0.038249642	1.890314817	up
Maea	0.038249873	1.905144692	up
Hagh	0.038268242	1.548351884	up
Siglech	0.038315766	1.943838	down
Slc38a4	0.03836365	1.964632154	down
Rnf10	0.038364749	1.524564982	up
Myl4	0.038376294	1.67110002	up
Akr7a5	0.038408671	1.610565782	up
Ube2j1	0.038464468	2.660007	down
LOC665237	0.038464468	2.139510393	up
Mknk1	0.038464468	1.59507072	up
E030038D23Rik	0.038480278	1.609140039	down

Acpl2	0.03851977	1.507196546	down
Ppp2r1b	0.038614959	1.505588412	up
Ints3	0.038625814	1.898773432	up
Snhg10	0.038723655	1.571330786	up
A930009E05Rik	0.038744293	1.94148171	down
Lama4	0.038744293	1.517658472	up
Tnnt1	0.038744293	2.21926713	up
5430433G21Rik	0.038755804	1.624912024	down
Psmb2	0.038785659	1.554743052	up
Vgll3	0.038829807	1.569160223	up
Nek1	0.038839541	1.677017689	up
Ttll1	0.038875029	1.539525986	up
St8sia4	0.03890568	3.115969658	down
Tubb2c	0.038912468	1.750983715	down
Mcam	0.038941573	1.855166197	up
Lrrn3	0.039021805	1.840212226	down
Ubfd1	0.03902809	1.921609998	down
Oprl1	0.039039973	1.900141478	up
Adcy4	0.039054859	1.56166184	up
2610016F04Rik	0.039054859	3.460516214	down
Emp3	0.03910007	1.871018648	up
Ppm1e	0.039124407	1.522260547	down
Tbc1d20	0.03917658	1.618924379	down
Sez6	0.039188579	1.700114965	up
Trove2	0.039188579	2.791873217	down
Serpina1b	0.039188579	1.608806372	up
Phox2a	0.039191656	2.039249659	up
Actr10	0.039212421	1.843064904	down
Wdr45l	0.039262526	1.757350922	up
6330407J23Rik	0.039268054	2.838205576	down
Prpf3	0.039293747	1.823522449	up
Wapal	0.039310508	1.597291231	up
Sfrs2	0.039320778	1.666710615	up
Ppp3r1	0.039353088	1.889593482	up
E230027K01Rik	0.039355617	1.62140882	up
Taf11	0.039361734	1.502123833	up
Urm1	0.039384525	1.527867794	up
Ppp1r10	0.039396197	1.944948435	down
Asph	0.039411657	1.554145455	up
D12Wsu95e	0.039444111	2.877092362	down
Necap1	0.039449677	1.63934505	up
Lasp1	0.039449677	1.619863749	up
Utx	0.039491713	1.597622991	down
Taf6l	0.03949922	1.505701065	up
Dusp10	0.039502278	1.548292041	up
Coq5	0.03958289	1.503633499	up
Rreb1	0.039586	1.629484773	up
Taf1	0.039586	2.118201971	down

Frmd4b	0.039591182	2.866351605	down
Kremen	0.039608404	1.516552806	up
Atp6v1a	0.039646652	3.656881571	down
Ydjc	0.039652214	1.979972601	down
LOC665235	0.039686009	5.708820343	down
Yy2	0.039707761	1.666549683	up
Gemin7	0.039718639	1.775089979	up
Reep1	0.039768819	1.611571431	down
B230312I18Rik	0.039775152	1.635258913	up
Gtf2f1	0.039777316	1.786022782	up
Plp2	0.039819136	1.705309033	up
A630036P20Rik	0.039819136	1.795637846	down
5430437P03Rik	0.039854094	1.837265372	up
Sugt1	0.039871227	2.872573614	down
Pla2g4b	0.039884802	1.812785864	up
Gpr23	0.039888471	3.774223805	down
Ctsh	0.039894439	1.76351583	up
Nit1	0.039894439	2.277081251	down
Rnf111	0.039894439	1.66894114	up
Cdk9	0.039894439	1.674052119	up
Mrps15	0.039894439	1.716216207	down
Gabrb3	0.039934386	1.954173803	up
Idi1	0.039934393	3.186533451	down
Elovl1	0.039934393	1.52536428	up
Numa1	0.03994076	1.515494943	up
Tcea3	0.03994076	1.639083862	up
LOC100046143	0.039943885	1.5993191	down
Cnot10	0.039981227	1.846111417	up
A130049L09Rik	0.040069927	1.594388962	up
Pcbp3	0.040072151	1.509883404	up
Josd2	0.040100403	1.928955078	up
Tmem2	0.040107153	3.613222599	down
LOC383872	0.040268526	1.503664017	up
Nelf	0.04027224	1.926457167	up
Ormdl3	0.040316697	1.509365559	up
Gatc	0.04032224	1.657716274	up
3100003M19Rik	0.040328294	1.838279247	up
Cycs	0.0403722	3.634145975	down
Gja4	0.040374443	2.194069386	up
Herpud2	0.040379122	1.715319872	up
Tshz2	0.04039197	1.758626103	down
LOC100044696	0.040404126	3.881947041	down
E2f3	0.040453829	1.648246527	down
Tcf12	0.040509388	1.750166893	down
Dmtf1	0.040535189	3.018440008	down
Cul4a	0.040569056	1.608663082	up
Thoc4	0.040576208	1.734910965	up
Slc25a27	0.040576208	1.652027369	up

Cd93	0.040576208	1.532142758	up
Dnmt1	0.040659122	2.134157419	down
AI314976	0.040690474	1.689166188	down
Ptprs	0.040708423	1.642102122	down
1810022K09Rik	0.040708508	2.546657324	down
2310079P03Rik	0.040718015	2.01312089	down
Klf16	0.040727887	1.552788138	up
732482	0.040737804	1.595599294	up
Apbb1ip	0.040755771	1.507820606	up
Rab8a	0.04075896	1.757183313	up
Bmp1	0.040793464	1.738113046	up
Myh10	0.040793464	1.665982604	up
Nup133	0.040794734	1.659139156	down
Shprh	0.040802702	1.506545067	up
Syt4	0.040802702	1.826842308	down
Elmod3	0.040802702	1.549108028	up
Dcps	0.040821273	1.618342876	up
LOC100046298	0.04085651	3.672389269	down
Plk2	0.040903006	1.823985696	up
Ascc3l1	0.040969335	2.43304348	down
ORF19	0.040979084	2.761018753	down
Mapk1	0.040983804	1.745192647	up
Wdr76	0.041046664	1.913261175	down
2700062C07Rik	0.041053575	1.610464335	up
Sh3kbp1	0.041053575	1.810052514	up
Repin1	0.041053575	1.731721878	up
Psen1	0.041141644	1.591003895	up
Mia1	0.041160375	1.953158498	down
Srfbp1	0.041201122	2.233648777	down
Rbms1	0.04121146	1.739798307	up
Mrps15	0.041259822	2.963686705	down
Nsmce2	0.041276231	1.582140684	down
Stxbp2	0.041276231	1.785886049	up
Sfmbt1	0.041289363	1.642952085	up
Tssc4	0.041307986	1.562817335	up
Dhx29	0.041307986	1.712465286	up
Srd5a3	0.04133039	1.591461539	up
Ccdc59	0.041421294	2.562474012	down
Reep5	0.041425061	1.620755076	up
Wfdc6a	0.041425061	1.577238202	up
Znfx1	0.041454848	1.620736718	up
Snapc3	0.041493386	1.591758251	up
Fance	0.041513957	1.676932693	up
2310022K01Rik	0.041571476	1.518654585	up
Vegfa	0.041598231	1.605612993	up
Cldn12	0.041632406	1.782491326	down
Nudt13	0.041632406	1.582317472	up
Slc9a3r2	0.041632406	1.523306727	up

Tmtc4	0.041632406	1.573067308	up
Pogk	0.041632406	1.70642376	up
Chchd4	0.041632406	1.543181181	up
Kremen2	0.041632406	1.681076527	up
6230415M23Rik	0.041655634	1.573527813	down
Vac14	0.041688055	1.616160274	up
1810030N24Rik	0.041736014	1.533046484	up
Iqwd1	0.041782945	1.672563314	up
Ccdc85b	0.041796345	1.804696798	up
Itgb4	0.041810986	1.925690413	up
C79407	0.041841988	1.791411877	down
Papd4	0.04185677	1.77568388	up
Zfand2b	0.041870873	1.628444076	up
Kcnab2	0.04187768	1.633401513	up
Kctd20	0.041882433	1.508466244	up
Pcdha7	0.041890327	1.561352015	down
D1Ertd471e	0.041890327	1.63830483	down
Bmf	0.041896135	1.800726652	up
Sfrp1	0.041909836	1.593710661	down
Pdcd4	0.041910682	2.215114117	down
Adam22	0.041920077	1.505896688	up
Cxcl14	0.041943714	1.843580723	up
A430041B07Rik	0.041954558	1.52143085	up
Cyb561	0.041954558	1.611446261	up
Epb4.9	0.041964803	1.590167403	up
Pde4dip	0.041964803	1.555087447	up
Map3k7ip1	0.041997369	1.608697891	up
2810452K22Rik	0.042017404	1.589414954	up
Foxred1	0.042032335	1.593425393	up
LOC100048858	0.042040724	2.377880096	down
Cdc42se2	0.042105962	3.283918381	down
LOC677317	0.042178225	2.444622994	down
AW551984	0.042184871	1.561046243	up
Mki67	0.042188063	2.990350723	down
Dnajc27	0.042257574	1.611613393	up
Rps4y2	0.042289194	1.633862019	up
Rras	0.042289998	1.515535235	up
Zfand2b	0.042300306	1.512717962	up
Apc	0.042303242	1.718425632	up
Megf10	0.042333972	2.129940748	up
Prnp	0.042333972	1.532065153	up
Robo2	0.042389616	1.518780828	up
Ftsj3	0.042409144	1.734473825	down
Tmem136	0.042420104	1.62801981	down
Rab7	0.042486277	1.687840939	up
Rps6ka1	0.042517297	1.511804819	up
Tirap	0.042537488	1.606068492	up
Ctsk	0.042537488	1.548027396	up

Zfp329	0.042592812	2.204562426	down
Ccnb1	0.042607497	2.72508049	down
LOC386005	0.042616036	2.025693655	down
Lin7a	0.042655177	1.608685613	down
Cpsf1	0.042684291	1.671303153	up
Psmd11	0.042755745	2.798700094	down
D1Ertd396e	0.042767074	3.044498205	down
Nfia	0.042795286	1.942586541	up
Blcap	0.042850994	1.613950133	up
Tardbp	0.042869546	1.589760065	up
Tgif2	0.042869546	1.563896537	up
LOC100041864	0.042915124	1.896019697	down
Snap29	0.04294651	2.2523489	down
Wdr89	0.043007191	4.202586651	down
2900001A12Rik	0.043012966	1.939251304	down
Dguok	0.043109439	1.570461392	up
Mapk1	0.043142095	1.77041018	up
Rbm3	0.043162394	1.782150388	up
Rnf41	0.043181725	1.573591232	up
0610010F05Rik	0.043221951	2.544930458	down
1110005A23Rik	0.043235108	3.571550608	down
LOC384943	0.043237388	1.580131888	up
Msl2l1	0.043281447	1.533810854	down
Rbm9	0.043282382	1.515487909	up
Vkorc1l1	0.043292563	1.763584137	up
2310047O13Rik	0.043316066	1.775619984	down
Olfr724	0.043347713	1.584443212	up
Ulk2	0.043349467	1.937405109	down
Kctd5	0.043485053	1.724708796	up
Fn1	0.043486372	2.97982192	down
Abhd12	0.043512426	1.517980576	up
Msl2l1	0.043576159	3.342426062	down
Ndfip2	0.043576159	3.606239796	down
Prep	0.043594528	2.69943285	down
BC023744	0.04362144	1.847045302	up
Smo	0.043641176	1.552044272	up
Arfgap2	0.043719001	1.599322915	up
Napg	0.043741513	1.552349567	up
Nfat5	0.043781508	1.719042063	up
Gm129	0.043781508	1.519572616	up
Sqle	0.043781508	1.873963237	up
AA407526	0.043781508	1.723120928	down
Aatf	0.043781508	1.794566512	up
3110001O07Rik	0.043782413	1.654144287	up
Tuba1a	0.043790069	1.930386066	down
2500002L14Rik	0.043803461	1.652361155	up
Tnpo2	0.043815579	1.509243727	up
Rnf26	0.043815579	1.627982259	up

Ppp1ca	0.043867454	1.659392119	up
AA536717	0.043893706	1.560150623	up
A030009H04Rik	0.043917391	2.949975967	down
Syne2	0.043928571	1.561118126	down
Slain2	0.043931734	3.133173466	down
Eml3	0.043931734	1.599015594	up
Chmp2a	0.043932241	1.603190899	up
Zc3hc1	0.043962576	1.598521352	up
Ndufc2	0.043962576	2.711074114	down
Rabl4	0.043970857	1.565464973	up
Atf7ip	0.043970857	1.804197907	down
scl0003067.1_7	0.044005249	1.54697609	up
Rnf138	0.044037737	1.682035804	down
AA408296	0.044098403	1.650300622	up
Krt15	0.044098403	2.829380751	up
Pick1	0.044107813	1.694794178	up
Msx3	0.044146866	1.967513681	down
2700029M09Rik	0.044247638	1.626795292	down
Rrn3	0.044271417	3.851872683	down
1110018J18Rik	0.044272628	1.635349631	up
Acad10	0.044272628	1.52994895	up
Ndufa1	0.04427458	1.566951275	down
Zfp385c	0.044319805	1.506323218	up
Ift172	0.044405732	1.681596756	up
BC027246	0.044415895	1.611726522	up
Dhcr24	0.044462081	2.892962456	down
Pld4	0.044481047	1.522917628	up
LOC100047016	0.044486493	1.649328709	up
Ndn	0.044486646	1.609985113	down
Nol4	0.044508033	1.791897178	down
Cacybp	0.044573195	4.869083405	down
Tmem178	0.044591058	2.772071838	down
Tor2a	0.04463543	1.541945696	down
Prr11	0.04472002	1.541226268	up
Emp1	0.04474083	1.530421853	up
Atp5b	0.044746712	1.504917979	up
LOC666053	0.044756111	1.688090444	down
Ctps	0.044756111	1.560593605	up
D930005D10Rik	0.044756111	1.521612406	up
Schip1	0.044815421	2.091172457	down
Irx2	0.044840399	1.736776829	up
E030040G24Rik	0.044840477	1.937999964	up
2610024G14Rik	0.044884946	1.685945988	up
EG665378	0.044904415	1.609692335	down
Ints3	0.044907905	1.585018158	up
2510002D24Rik	0.044909865	1.503829598	up
Hs1bp3	0.044947673	1.551209569	up
Panx1	0.044952855	1.931941986	down

Polr2a	0.044968978	1.547062993	down
1810022O10Rik	0.045012709	1.613938093	down
Efna4	0.045035712	1.507464409	up
Clk2	0.045119248	1.571323037	up
Zfp191	0.045119248	1.782525182	up
Spc25	0.045134753	2.297004461	down
Tyw1	0.045142017	1.65341568	up
EG624866	0.045186788	1.574350119	up
BC003993	0.04519375	1.595288634	up
D330001F17Rik	0.04519375	1.682675123	up
2610019N13Rik	0.045205791	1.504050016	down
Fdxr	0.045205791	1.51813817	up
6430550H21Rik	0.045233809	2.900570869	down
Bmp1	0.045236804	1.995870352	up
Psmd9	0.045311436	1.72971046	down
Kat5	0.045313828	1.622348547	up
1700011J10Rik	0.04532906	1.705316782	down
Sdf2	0.04532906	1.542481661	up
B930044G13Rik	0.04532906	1.75112772	down
Pde6d	0.04532906	1.674471498	up
Kif21b	0.045335036	4.306611061	down
Tsr1	0.045367807	2.420409441	down
Rbbp7	0.045383625	1.951814175	up
Arhgap8	0.045383625	1.514266491	up
Yap1	0.04554674	1.573176146	up
Pole3	0.045608342	1.514801145	up
Jak1	0.045614041	1.616309404	up
Tfrc	0.045631029	1.614941835	up
Efna4	0.045632884	2.024995565	up
C430048L16Rik	0.045650616	2.14329505	down
Ung	0.045652077	1.714624524	up
H2afy2	0.045685496	1.901653528	up
C230004L04	0.045686554	2.225656748	down
Kctd20	0.045686554	1.829118729	up
Tmed10	0.045686554	1.824732423	up
Mtmr12	0.045686554	2.528917551	down
Lrrc45	0.045701459	1.674571872	up
Eps8	0.045701459	1.565937281	up
Asph	0.045701459	1.518949151	up
Ccna2	0.045707922	1.643437266	down
2010004N17Rik	0.045716822	1.539157748	up
Med11	0.045950092	1.523451805	up
Cyr61	0.045953054	2.024758816	down
Brd4	0.045968633	1.697463751	up
Nbr1	0.04599157	1.665898562	up
Dnd1	0.045991756	1.53112495	up
LOC100043527	0.046030201	3.223002672	down
Camk2n1	0.046030201	1.771095872	down

Prosapip1	0.046052773	1.565313458	up
Cyba	0.046149857	1.730954647	up
Zfp397	0.046176747	1.554591537	up
Nudt2	0.046181265	1.64345181	up
Cited2	0.046197835	1.539243817	down
Bglap2	0.046206947	1.561390162	up
Gtf3a	0.046245862	1.86926043	up
Spg21	0.046265062	1.709075213	up
Cep110	0.04628763	1.570485711	down
Ergic3	0.04628763	1.748751283	up
Trmt1	0.046318419	1.576895714	up
Dhx38	0.046330575	1.535974026	up
Csda	0.046330575	3.899050474	down
Sorbs1	0.046397738	3.83799386	down
Fh1	0.046397738	4.688094139	down
B930095G10Rik	0.046473369	1.629758835	up
D130027K14Rik	0.046473369	1.526666641	down
Prelp	0.046514008	1.568042874	down
Fxr2	0.046559513	2.407452345	down
Dgcr2	0.046614908	1.579515696	up
Abcd3	0.04667072	1.503739834	up
Epha4	0.046758242	1.533346653	up
Cald1	0.046800658	2.376091242	down
Gli3	0.046828698	1.722111464	up
Smarce1	0.046829943	1.618265748	up
Psg23	0.046869643	1.703472614	down
Cyr61	0.046914451	1.985097051	down
Rnf20	0.046938404	2.823944092	down
Coq9	0.04694929	1.583897233	up
Ghitm	0.046957102	2.268934965	down
Zfp639	0.046985906	1.605192184	down
1110033C18Rik	0.046996091	1.509202838	up
Ncapg2	0.046996091	2.070360184	down
6030458C11Rik	0.047014143	2.817769766	down
Mageh1	0.047040835	1.528167605	up
Agap1	0.047097869	1.561980009	down
Asxl1	0.047188308	3.117561579	down
LOC632667	0.047188308	2.608660936	down
1700052N19Rik	0.047188308	1.691929579	up
Gp5	0.047213323	2.465470791	down
Zc3h13	0.047225397	1.66297245	down
Gsk3b	0.047235537	1.857083082	down
1200003C05Rik	0.047253672	1.604827762	down
LOC100039751	0.047264375	5.133409023	down
Uchl5	0.047422584	3.453968525	down
Gnaq	0.047436904	2.459468603	down
Qsox1	0.047464076	1.525249481	up
Ncaph2	0.047464076	1.565834999	up

Cln6	0.047464076	1.633901715	up
5830415L20Rik	0.047464076	3.780449867	down
Aspm	0.047514413	2.961087942	down
Bbs1	0.047522485	1.576906443	up
Mrgprg	0.04758421	1.512146831	up
Garnl1	0.04758421	2.604614258	down
1300006C19Rik	0.047614522	1.657463312	up
Bmyc	0.047614522	2.085510254	down
Bcl11b	0.0476331	1.591125727	down
1110031B06Rik	0.047673501	1.709198117	up
Armcx6	0.047698923	1.572029233	up
Epm2aip1	0.047698923	5.14840889	down
Zdhhc15	0.047760572	1.61785531	up
Ahctf1	0.047765739	1.875687957	down
Yipf6	0.047791574	1.623640895	up
9430052C07Rik	0.047795691	2.694894314	down
Exoc7	0.047805186	1.881109238	down
Treml1	0.047830489	2.249850988	down
Mrpl44	0.04784672	1.56663239	up
Lypla1	0.047851659	1.651063442	up
Psmc6	0.047880087	2.955043793	down
1110007L15Rik	0.04790986	1.5016464	up
Tial1	0.04795358	2.964385033	down
Nfu1	0.047986947	2.798967123	down
8030402P03Rik	0.047986947	1.679128289	down
Dnajb9	0.04810521	4.439086437	down
6720456B07Rik	0.048186306	1.556939125	up
Sep-15	0.048253268	1.571727395	up
Tox3	0.048272651	5.636298657	down
Rfc4	0.04834811	2.769906998	down
Drbp1	0.04842443	2.473812103	down
Smek1	0.048454452	3.159136534	down
Wdr60	0.048558105	1.820384145	down
Capzb	0.048681296	1.548209906	up
Unc45a	0.048742175	1.914470673	up
Inppl1	0.048769519	1.53313446	up
Map1lc3b	0.04877251	5.538018704	down
Pdlim7	0.04877251	1.598556638	up
Tmem25	0.048773546	1.80103612	up
Luc7l2	0.048800197	2.829208851	down
LOC100043209	0.048826642	1.657682061	down
Ube2n	0.048871927	3.194690466	down
LOC381561	0.048917007	2.65517807	down
Gmps	0.048948206	1.572574258	down
Upf3a	0.048987888	1.775831699	up
Sfrs12ip1	0.048987888	1.824899554	down
B3gat3	0.049001496	1.655320168	up
Trim59	0.049037885	3.624661446	down

LOC433955	0.049045999	1.868902803	down
LOC672274	0.049047958	7.474191189	down
Hectd3	0.04905716	2.102703333	down
Rxrb	0.049133807	1.510391116	up
E2f5	0.049141314	1.763564467	down
Mxi1	0.049173378	4.718586445	down
Irgq	0.049189415	1.544440866	down
Akr7a5	0.049189415	1.557494283	up
Pla2g12a	0.049227577	1.516431689	up
Thoc1	0.049227577	1.621263027	up
Srpx	0.049241252	2.213653088	up
Cdyl	0.049255759	1.588329315	up
Efha1	0.049387131	1.696015954	up
Map4k4	0.049401984	1.657858253	down
Ugt1a10	0.049403165	1.688376308	up
1200003I07Rik	0.049482178	1.600456357	down
Spag9	0.049519125	2.197977543	down
Rangrf	0.049601477	1.659832597	up
Kif23	0.049606953	2.474379063	down
Tagln2	0.049606953	1.654120445	up
2310003C23Rik	0.049721658	1.868865371	up
LOC386082	0.049725164	2.323030949	down
Slc25a33	0.049727015	1.513374448	up
Bbs2	0.049759805	1.559252262	down
St3gal5	0.049773734	1.786075234	up
D2Wsu81e	0.049792573	1.653146982	up
Prpsap2	0.049874988	4.381533623	down
Tarbp2	0.049915534	1.526001215	up
Phka2	0.049941957	1.565676451	up
Sas	0.049945593	1.506925583	up
Rab21	0.04996042	1.559420466	up
Prpf38a	0.049970407	2.950079203	down
Mcl1	0.049976468	1.694028497	down
Dhrs7b	0.049980972	1.625535369	up
Elavl2	0.049980972	2.052917719	down

Table S1 Sox9 (123 Pages)

Symbol	Corrected p-value	FCAbsolute	Regulation
Snurf	2.43E-07	16.28224373	down
Snurf	2.57E-07	10.4280653	down
Ahcyl1	2.88E-07	2.944601536	down
G3bp1	5.99E-07	17.03670883	down
Sqle	5.99E-07	11.19085312	down
Cttn	9.85E-07	5.365588188	down
Mela	9.85E-07	193.5155029	up
Atp6v1a	9.94E-07	11.08010292	down
Btg1	9.94E-07	16.80504227	down
Klhl9	9.94E-07	5.711296558	down
Barx1	1.03E-06	5.78196907	down
Clic4	1.03E-06	4.74880743	down
Mia1	1.03E-06	12.40242386	down
Ogn	1.03E-06	8.761684418	down
Pea15a	1.03E-06	7.083482742	down
Fcer1g	0.000001069	6.75583458	up
LOC381215	0.000001069	3.708198786	up
scl000963.1_12	0.000001069	27.45366478	down
Sh3glb1	0.000001069	1.921491623	up
Tpm3	0.000001069	12.23949337	down
Wdr45l	1.08E-06	3.462718725	down
Fgfr3	1.16E-06	5.09095192	down
LOC209281	1.16E-06	7.462760925	down
Rbm6	1.16E-06	3.783868313	down
D10Ertd610e	1.24E-06	4.498394013	down
Mela	1.24E-06	7.264508247	up
Rnf14	1.24E-06	3.462059498	down
9626100_15	1.25E-06	8.96787262	up
Angpt1	1.25E-06	3.246338844	down
LOC224732	1.25E-06	4.148701668	down
Calu	1.48E-06	13.70756626	down
LOC546090	1.55E-06	8.179782867	down
Pmp22	1.55E-06	12.38402176	down
Matn4	1.62E-06	25.97351456	down
Rab9	1.66E-06	3.348532677	down
Ctbp2	1.76E-06	6.877697468	down
Xlr3a	1.96E-06	5.381691933	up
LOC269859	1.97E-06	9.448865891	down
Prkar2b	1.97E-06	2.270573378	down
Recql	1.97E-06	2.369452715	down
Sec23a	1.97E-06	11.70175171	down
2310008I22Rik	2.00E-06	3.89390111	down
2310035C23Rik	2.00E-06	2.845045567	down
2810438F06Rik	2.00E-06	2.042603016	up
Atp6ap2	2.00E-06	6.724124908	down
Clic4	2.00E-06	3.674860239	down
Cugbp1	2.00E-06	4.875402927	down
Cxadr	2.00E-06	2.025967121	down
E130306F01Rik	2.00E-06	5.990415573	down
Ifi27	2.00E-06	4.797798157	up
Kdelr2	2.00E-06	8.655071259	down

LOC333331	2.00E-06	5.009912491	down
Mapk1	2.00E-06	5.47116375	down
Mest	2.00E-06	8.560221672	down
scl0001905.1_3	2.00E-06	10.18041611	down
Sec61a2	2.00E-06	7.059074402	down
Wrnip1	2.00E-06	4.550052166	down
Cthrc1	2.08E-06	7.363954544	down
Elp2	2.08E-06	9.949894905	down
9626953_200	2.12E-06	5.303401947	up
Atp6ap2	2.12E-06	6.21543026	down
Eef2	2.12E-06	11.13695908	down
Scin	2.12E-06	11.84676552	down
Spop	2.15E-06	1.968521237	up
Xist	2.15E-06	4.769120216	up
LOC100047353	2.16E-06	16.09091377	down
Tpm4	2.19E-06	11.63345242	down
Zfp467	2.19E-06	1.528747797	up
Hspd1	2.26E-06	26.63630295	down
LOC623031	2.26E-06	8.619883537	down
Tmed2	2.26E-06	15.25777626	down
Trappc5	2.26E-06	2.098404884	up
2310022M17Rik	2.33E-06	5.095258713	down
Gdi2	2.46E-06	11.7765274	down
B130052P14Rik	2.59E-06	2.191059828	up
2700088M07Rik	2.60E-06	4.098806858	up
1700021K14Rik	2.67E-06	1.773165345	up
Dars	2.71E-06	8.645347595	down
Smc2	2.71E-06	2.254477501	down
Atl2	2.84E-06	3.026691914	down
Bmi1	2.84E-06	7.92621088	down
Ift122	2.84E-06	2.446288109	up
Nrp	2.84E-06	3.627279997	up
Rab12	2.84E-06	3.008924007	down
Sfrs12	2.84E-06	2.5010252	down
Tpm4	2.84E-06	5.901847839	down
Sox10	2.85E-06	6.190069199	down
Tatdn3	2.90E-06	2.010156393	up
Efr3a	2.98E-06	3.849843025	down
Cdc45l	3.09E-06	5.877048492	down
Has2	3.09E-06	2.12053895	down
Pus10	3.09E-06	3.560310125	down
Minpp1	3.12E-06	7.255403996	down
Msh2	3.14E-06	1.977584004	up
LOC100047252	3.15E-06	9.643625259	down
LOC672474	3.15E-06	2.250611305	up
D7Ertd715e	3.18E-06	6.354035378	down
Tcp1	3.27E-06	10.78222847	down
Ube2v1	3.27E-06	3.053926945	down
AW822216	3.40E-06	2.000539064	up
E430024F02Rik	3.42E-06	2.296355009	down
Mboat2	3.42E-06	4.328363895	down
Smox	3.42E-06	4.023831844	down

Rab1b	3.43E-06	4.572256565	down
2310014G06Rik	3.43E-06	3.220025778	down
Calu	3.43E-06	5.714842796	down
EG623867	0.000003518	11.96581936	down
Asb3	3.57E-06	7.99809885	down
Slc4a1	3.63E-06	6.088127136	up
1700029J07Rik	3.67E-06	2.406618357	up
Mtdh	3.67E-06	1.778023362	down
Plxna4	3.68E-06	4.854547024	up
Akap1	3.76E-06	2.008526325	down
Herpud2	3.76E-06	3.54115653	down
Psmd2	3.76E-06	6.272919178	down
Rab6	3.76E-06	13.71080399	down
Ptcd2	3.77E-06	4.998456478	down
Akap2	3.77E-06	5.325752258	down
Btbd2	0.000003773	4.857840061	down
LOC100044314	0.000003773	4.464912415	up
Tmpo	0.000003773	4.254616261	down
Aif1	3.90E-06	4.060036659	up
Cldn5	3.90E-06	4.535059929	up
LOC100048832	3.90E-06	6.488455296	down
9626100_224	3.94E-06	21.01912117	up
Usp8	4.03E-06	3.155760288	down
Trim23	4.08E-06	3.059520483	down
Tram1	4.13E-06	9.069438934	down
H2afy2	4.13E-06	8.006081581	down
9626962_229	4.15E-06	4.103837967	up
Ptpn12	4.16E-06	5.469593525	down
1300006C19Rik	4.30E-06	3.872225285	down
4732415M23Rik	4.30E-06	1.610247612	up
Atad2	4.30E-06	2.804745674	down
LOC100039786	4.30E-06	5.608729363	down
LOC100046775	4.30E-06	5.361951351	down
Nxf1	4.30E-06	3.640837431	down
P4ha3	4.65E-06	2.543177605	down
Rnf167	4.65E-06	2.689976931	down
Tmem49	4.65E-06	3.617798567	down
Rec8	4.67E-06	6.115628243	up
Slc35b2	4.67E-06	5.981288433	down
Cd53	4.76E-06	6.751580238	up
Rnf13	0.0000048	5.99867487	down
Cstf3	4.91E-06	2.267512322	down
LOC333744	0.000004997	7.278337956	down
Ppap2b	0.000004997	4.089593887	down
Armc8	5.04E-06	5.477164745	down
9626958_317	5.13E-06	10.43560219	up
A930019L04Rik	5.13E-06	2.81422925	up
Unc119b	5.13E-06	3.685203075	down
Jarid1d	5.18E-06	4.988981724	up
LOC213411	5.24E-06	18.34956932	down
Gli3	5.60E-06	3.173316717	down
Eef2	5.62E-06	12.79270744	down

Vps16	5.67E-06	3.360454083	down
Gdpd3	5.68E-06	2.35029006	up
Gtf3c6	5.74E-06	1.66050005	up
LOC269365	5.74E-06	2.545342684	up
Srm	5.74E-06	8.38906765	down
Ubqln1	5.74E-06	3.357088804	down
Nup155	5.75E-06	2.335738897	down
Rnf4	6.01E-06	5.546492577	down
Rnf8	6.12E-06	3.67894268	down
Papss2	6.25E-06	6.537036419	down
Tmem214	6.28E-06	2.80851984	down
9626962_211_rc	0.000006281	13.76020813	up
Tmem49	6.42E-06	5.131297112	down
Grid2	0.000006474	2.567212582	up
Col9a1	6.49E-06	19.59921646	down
Hsd11b2	6.49E-06	2.732875347	up
Tank	6.49E-06	1.960616708	down
Pou3f4	6.58E-06	2.28909874	down
LOC380888	6.60E-06	2.474255323	down
Snx2	6.60E-06	5.240269661	down
Prpsap2	6.84E-06	2.040724993	down
LOC667609	6.87E-06	2.895514965	down
Dbc1	6.89E-06	3.628328562	up
Hmgcr	6.89E-06	3.791461468	down
Hsp90ab1	6.92E-06	5.004231453	down
Slc25a36	6.94E-06	5.999230862	down
1110004P21Rik	7.19E-06	3.858745813	down
Ola1	7.21E-06	4.827047825	down
Zfp558	7.21E-06	1.616594911	down
LOC100045971	7.23E-06	2.366033077	down
Csnk1g2	0.000007409	3.149769068	down
Col6a3	7.47E-06	1.872129917	down
Hoxa11s	7.52E-06	3.320790768	up
Tmed4	7.56E-06	5.147822857	down
1110038B12Rik	7.58E-06	2.310216904	down
Gorasp2	7.59E-06	5.674937725	down
Elovl1	0.000007712	3.125793219	down
Maea	0.000007712	4.912212849	down
Xrcc6	0.000007712	5.7110672	down
Prelp	7.95E-06	11.84494877	down
Ubqln1	7.95E-06	2.729489088	down
Cdk2	7.96E-06	5.984936714	down
LOC100044622	8.01E-06	6.554626942	down
Ccnyl1	8.03E-06	3.299476624	down
BC038881	8.07E-06	1.766301632	up
Hdhd2	8.13E-06	4.419561386	down
Oc90	8.13E-06	4.306143761	down
Tmed7	8.13E-06	7.199827671	down
Itm2a	8.44E-06	20.30648041	down
Plxnd1	8.49E-06	2.984797239	up
1700065O13Rik	8.54E-06	2.390968323	down
Aifm1	8.54E-06	3.607730627	down

Got2	8.54E-06	6.931339741	down
Rbbp7	8.54E-06	17.42933273	down
Gmppa	0.000008692	4.846647263	down
1200015F23Rik	8.85E-06	2.735702276	down
C78339	8.85E-06	2.311928272	down
Ncoa4	8.85E-06	3.41334939	down
9230108I15Rik	8.95E-06	2.198386192	up
Slc37a3	9.05E-06	2.924352408	down
Adk	9.09E-06	3.20015502	down
9626096_327	9.15E-06	5.376810551	up
Shisa5	9.15E-06	2.831512213	down
Ap1s1	9.23E-06	3.702727556	down
A830080H07Rik	9.24E-06	1.565250874	up
Vps41	9.24E-06	3.322981596	down
9629514_325	9.47E-06	5.342110634	up
Igfbp3	9.52E-06	5.452507973	down
Mtmr14	9.69E-06	2.83305788	down
Tollip	9.75E-06	3.288922787	down
Kbtbd7	9.88E-06	3.826936007	down
Zdhhc13	9.88E-06	2.961416245	down
Gnpda1	1.00E-05	3.111648083	down
Insig1	1.00E-05	3.88476634	down
Cryz	1.02E-05	4.563667297	down
4921504I02Rik	0.000010238	4.941533566	up
Frmd6	0.000010238	1.692186475	up
Rbpjl	0.000010238	2.951968432	down
1300014I06Rik	0.000010243	2.328416109	down
Sct	0.000010243	2.291469574	up
LOC100047915	1.05E-05	4.770830631	down
Acan	1.08E-05	6.213802338	down
Syradb	1.08E-05	2.403548241	down
LOC100047696	1.11E-05	4.953800678	down
Scp2	1.11E-05	2.886713743	down
Pja1	1.11E-05	2.872586489	down
Slc44a2	1.11E-05	2.969364405	down
Hpcal1	1.12E-05	1.939287782	down
Prei4	1.12E-05	2.491890669	down
St3gal6	1.12E-05	2.495745421	down
Wwp2	1.12E-05	5.120452404	down
Exoc4	1.12E-05	1.859408855	down
Ibtk	1.12E-05	2.420808315	down
Tpm3	1.12E-05	4.65392971	down
Asah1	1.13E-05	6.742412567	down
Cep57	1.13E-05	4.318086147	down
Prosc	1.13E-05	4.061861992	down
EG623133	1.14E-05	3.179880858	down
Mkrn3	0.00001142	6.613668442	down
2700089E24Rik	0.000011457	3.30083251	down
Rbbp7	1.17E-05	15.93036461	down
9630025I21Rik	1.17E-05	1.767906904	up
Msrb3	1.17E-05	2.706700563	down
Atf4	1.18E-05	3.893290997	down

Col11a1	1.18E-05	3.070882559	down
Efha1	1.18E-05	3.982237577	down
Ppp2r2a	1.18E-05	6.375761509	down
2410014A08Rik	1.18E-05	2.083367586	down
Vars	1.19E-05	1.957713485	down
Etnk1	1.23E-05	3.267770767	down
Stk3	0.00001239	4.311697006	down
Mia3	1.25E-05	3.627288818	down
Acan	1.26E-05	3.726875544	down
4933411D12Rik	0.000012829	3.839246273	up
Treml1	0.000012829	3.888500691	up
Umps	0.000012829	7.797474861	down
Cul4a	1.30E-05	3.606418371	down
Prnpip1	1.30E-05	4.535410404	down
4930504E06Rik	1.30E-05	2.830984354	down
Actc1	1.30E-05	2.089511871	up
Alas2	1.30E-05	3.438703537	up
LOC100044411	1.30E-05	6.985604286	down
Ndrg2	1.30E-05	2.594526529	down
Paip2b	1.30E-05	4.85147047	down
Pecam1	1.30E-05	4.577373505	up
LOC100045081	1.30E-05	3.047186613	down
LOC100047759	1.30E-05	1.631498098	down
Klhl9	1.30E-05	5.928988457	down
2010004N17Rik	1.34E-05	2.486142159	down
4933433P14Rik	1.37E-05	2.907008886	down
Cttn	1.37E-05	3.056124687	down
Nt5c2	1.38E-05	4.751771927	down
Mtch1	1.38E-05	7.152193546	down
Mtbp	1.40E-05	3.607527256	down
Acat3	1.40E-05	7.240067959	down
Caprin2	1.40E-05	2.33762145	down
Chmp2a	1.40E-05	2.305363894	down
Cyp51	1.40E-05	3.651641369	down
LOC674427	1.40E-05	2.086430073	down
9930021J03Rik	1.41E-05	1.895258188	up
LOC100048832	1.41E-05	1.708787084	down
Ppm1b	1.41E-05	2.718099833	down
Mpzl1	1.42E-05	2.614463806	down
Zfp449	1.42E-05	2.853039026	down
Apaf1	1.43E-05	4.611181736	down
LOC225468	1.43E-05	2.575030565	up
Rbm6	1.44E-05	2.153254986	down
Xpnpep1	1.44E-05	5.149816513	down
Atg2a	1.45E-05	2.390595436	up
4930511J11Rik	1.45E-05	3.008134604	up
Rgs3	1.45E-05	1.874791026	down
Ptx3	1.45E-05	2.150045157	down
Ccne2	1.48E-05	4.694009304	down
Chd3	1.50E-05	3.354319334	up
Rab7	1.50E-05	4.727404594	down
Skp2	1.55E-05	9.29180336	down

Crtap	1.55E-05	3.247110605	down
D8Ertd82e	1.55E-05	2.100708962	up
Myo9b	1.56E-05	2.652628422	up
Rnf167	1.56E-05	3.185821533	down
Spin	1.58E-05	3.923149109	down
Etv1	1.58E-05	4.297760963	down
Asb4	1.59E-05	3.994550705	down
LOC100046320	1.59E-05	6.290267944	down
Calu	1.60E-05	5.472316742	down
Lin28b	1.60E-05	2.55594039	up
Chmp7	1.60E-05	2.793775082	down
D0Kist3	1.60E-05	2.104086399	up
Etv1	1.60E-05	3.938217402	down
Iah1	1.60E-05	1.688607693	up
Kars	1.61E-05	5.208313942	down
LOC434200	1.61E-05	4.22437048	down
Rabl3	1.61E-05	3.038547039	down
2600002B07Rik	1.63E-05	2.618479013	up
Mat2a	1.63E-05	5.996403217	down
scl0002130.1_20	1.63E-05	3.74867034	down
Ptbp2	1.63E-05	2.011176109	down
Hadh	1.64E-05	5.792607307	down
Gse1	1.65E-05	1.679089785	up
Mospd1	1.65E-05	4.566000938	down
scl0004085.1_27	1.65E-05	4.085368633	down
Prei4	1.65E-05	2.493928909	down
Krit1	1.67E-05	3.371637583	down
Spcs3	1.67E-05	4.002120495	down
Adam11	1.67E-05	1.94789505	up
Fndc3a	1.67E-05	3.297939777	down
Papss2	1.67E-05	3.08749795	down
Tarsl2	1.67E-05	2.547206879	down
Rab23	1.68E-05	4.180167198	down
Mospd1	1.68E-05	3.877674341	down
Vps26a	1.68E-05	2.7923069	down
Napa	1.69E-05	4.319627285	down
Tsc22d1	1.69E-05	2.117260933	down
0610007P14Rik	1.73E-05	8.547762871	down
EG633692	1.73E-05	6.53498745	down
Tyro3	1.73E-05	2.08830595	up
Alg6	1.74E-05	2.485656261	down
Bri3bp	1.74E-05	1.567324281	down
Snai3	1.74E-05	2.162145853	up
Asxl2	1.74E-05	2.471994638	up
Cacna1g	1.74E-05	2.106597185	up
Zfp811	1.74E-05	2.148871183	up
Fkbp1a	1.75E-05	1.817640781	down
LOC100046136	0.000017593	12.50759029	down
Mical1	1.77E-05	2.097376823	up
Cops7a	1.80E-05	2.400217056	down
B3galnt2	1.80E-05	5.006847858	down
Snx14	1.80E-05	3.882187605	down

Extl1	1.81E-05	3.756691933	down
LOC433749	1.81E-05	4.241154671	down
Zfp422-rs1	1.81E-05	6.653063297	down
EG384596	1.82E-05	2.411724091	up
Pcmt1	1.82E-05	4.647210121	down
4931428F04Rik	1.83E-05	1.665858388	up
Bst2	1.83E-05	3.539474964	up
LOC545208	1.83E-05	10.7373724	down
Dock6	1.83E-05	2.06994915	up
1110057K04Rik	0.000018377	3.0881989	down
2610015J01Rik	0.000018377	2.612410307	down
9230111I22Rik	0.000018377	3.087126017	up
Ipmk	0.000018377	1.925785184	down
Car10	1.84E-05	1.700924516	up
Pfn1	1.84E-05	2.536902905	down
Tssc1	1.84E-05	4.981688023	down
Bmpr1a	1.84E-05	4.158643246	down
2410014A08Rik	1.84E-05	2.12929678	down
4930503L19Rik	1.84E-05	4.866831303	down
Gpr125	1.84E-05	2.239007235	down
Fbxl12	1.85E-05	2.775398493	down
4933433P14Rik	1.88E-05	2.382874727	down
C030002B11Rik	1.88E-05	1.840402603	up
Mpeg1	1.88E-05	12.81386948	up
BC002199	1.89E-05	3.518777847	down
Tmem9b	1.89E-05	2.921584129	down
Sep-10	1.89E-05	1.895076871	down
2410003P15Rik	1.89E-05	3.252454758	down
Acvr1b	1.89E-05	2.186805487	down
Ctgf	1.89E-05	4.026652813	down
Fstl1	1.91E-05	30.46147919	down
Myo1b	1.91E-05	2.122879982	down
Mkks	1.91E-05	2.561979294	down
Snx27	1.92E-05	1.875513554	down
4833426H15Rik	1.92E-05	2.979052544	down
Wapal	1.92E-05	2.248007536	down
Crebbp	1.92E-05	2.316209078	up
Necap1	1.92E-05	3.76947999	down
Kat5	1.93E-05	2.21811986	down
Tgoln1	1.94E-05	2.579447985	down
Wnt5a	1.94E-05	3.874965906	down
Islr	1.96E-05	17.02267647	down
Acot7	1.96E-05	4.576246262	down
Clint1	1.97E-05	3.635176659	down
Col27a1	1.99E-05	4.319157124	down
Amotl1	1.99E-05	2.031495333	up
Tmem51	2.01E-05	1.853325009	up
Nupl1	2.01E-05	2.275214911	down
Slc31a1	2.03E-05	2.899451971	down
Bnip2	2.04E-05	2.457861662	down
Chka	2.04E-05	1.859719396	up
Foxred1	2.04E-05	3.15153718	down

Glt8d2	2.04E-05	1.654757619	down
Ube2q2	2.04E-05	3.231334448	down
Snrk	2.04E-05	2.067819834	down
Tmed2	2.05E-05	9.661296845	down
Unc119b	2.05E-05	4.320408344	down
Lrrk2	2.05E-05	1.88201201	up
Kcns1	2.06E-05	2.092708826	down
Tnfrsf11b	2.06E-05	2.316004276	down
A830029A02Rik	2.09E-05	3.166808128	up
Map4k5	2.09E-05	1.932198763	down
Acd	2.13E-05	2.198358774	down
Clec4n	2.13E-05	8.130173683	up
Chpt1	2.14E-05	3.158918142	down
C430010C01	0.000021497	2.339282036	up
Atg9a	2.16E-05	2.526178122	up
Bdh1	2.16E-05	2.179454565	down
LOC380906	2.17E-05	4.781121254	up
Trip6	2.17E-05	1.987746	down
Gnal	2.18E-05	2.386161327	down
Gnpda1	2.18E-05	2.365849018	down
Hyal2	2.18E-05	6.069654942	down
l7Rn6	2.18E-05	2.182899237	down
Mboat2	2.18E-05	4.121282101	down
Slc25a30	2.18E-05	2.348610163	down
Tbc1d14	2.18E-05	3.995583773	down
Uros	2.18E-05	3.532380581	down
Ergic3	2.19E-05	5.783002853	down
EG623818	2.19E-05	1.969997287	down
Gpr108	2.19E-05	2.545031309	down
LOC382050	2.19E-05	1.687505722	up
Mgl1	2.20E-05	5.579555512	up
LOC382237	2.20E-05	1.810676694	up
Polr1c	2.20E-05	1.782086372	up
Tmpo	2.21E-05	5.473426819	down
Arf5	2.23E-05	3.409604311	down
Arfgap1	2.23E-05	4.403191567	down
Heca	2.23E-05	1.8897264	down
Thbs1	2.23E-05	5.464723587	down
Tmtc4	2.23E-05	2.423789501	down
LOC100043982	2.25E-05	3.860621214	up
Mtmr2	2.25E-05	3.86826539	down
E330018D03Rik	2.26E-05	1.895284414	up
LOC633016	2.26E-05	21.21012306	down
Smpdl3b	2.26E-05	1.969936967	down
Ptpn12	2.27E-05	3.96179533	down
Anks3	2.28E-05	3.442865372	down
Csde1	2.28E-05	6.921939373	down
LOC100045963	0.000022956	1.98790288	down
LOC382262	2.31E-05	2.096614361	down
Col14a1	2.32E-05	2.242420435	down
Osbpl1a	2.33E-05	1.919828653	down
Wars	0.00002363	3.663579941	down

Wisp1	2.37E-05	2.730303288	down
1110012F10Rik	2.38E-05	1.65092063	up
Gatad1	2.40E-05	2.695825577	down
Rab7	2.40E-05	3.20967865	down
Sec22a	2.40E-05	5.233076572	down
Slc46a3	2.40E-05	1.976843119	up
Tmed4	2.40E-05	3.844161034	down
Trub1	2.40E-05	2.430801392	down
1500002C15Rik	2.41E-05	1.634621263	down
LOC383775	2.41E-05	3.01973486	up
Ndn	2.42E-05	5.251294136	down
Cd109	2.44E-05	1.82360971	down
Unc119b	2.44E-05	2.544879913	down
Gatc	2.45E-05	3.157745838	down
Mar-07	2.48E-05	4.280188084	down
D030041N04Rik	2.48E-05	2.50186491	up
Tep1	2.48E-05	2.25101471	up
2510002D24Rik	2.49E-05	2.415004015	down
4932441P04Rik	2.49E-05	1.885864019	up
G630097J24Rik	2.49E-05	1.787307024	up
Klhl10	2.49E-05	2.165116549	up
LOC627317	2.49E-05	2.867682695	up
Hnrnpd	2.49E-05	2.492981672	up
Paip1	2.49E-05	5.300552368	down
A730008H23Rik	2.49E-05	2.572934628	down
Gnas	2.49E-05	6.219718933	down
Pdia5	2.49E-05	2.353853941	down
Wnk3	2.52E-05	1.740400434	down
LOC218060	2.53E-05	3.266451836	up
Cep120	2.55E-05	2.166876555	up
Eraf	2.58E-05	3.987465382	up
Nol3	2.58E-05	3.042418957	up
scl00065.1_3086	2.59E-05	2.145645142	up
Man2b1	2.61E-05	3.427057982	up
Metap2	2.61E-05	4.677306652	down
Psmd5	2.61E-05	3.978257418	down
Ranbp3	2.61E-05	2.679462671	down
Lepre1	2.62E-05	3.086449862	down
scl000408.1_6	2.62E-05	4.554535866	down
Tubgcp3	2.63E-05	2.060122728	up
Ggcx	2.64E-05	1.750491977	down
Otud1	2.64E-05	1.665085912	down
Klk8	2.65E-05	3.280446053	up
LOC100047009	2.65E-05	3.624681234	down
EG668300	2.65E-05	3.491343498	up
LOC100045864	2.66E-05	3.138421059	up
Tmtc4	2.66E-05	2.443422556	down
5730446D14Rik	2.69E-05	2.450055122	up
Ruvbl1	2.69E-05	6.580038548	down
scl000215.1_14	2.71E-05	2.171081305	down
LOC381468	0.000027511	2.32368803	down
Zfp326	0.000027511	2.246153832	up

9830166E18Rik	2.75E-05	4.97649765	up
Zfp110	2.75E-05	2.602319241	down
2700060E02Rik	2.77E-05	4.437078953	down
2700086A05Rik	2.78E-05	2.162735224	up
Serpina3n	2.78E-05	1.593630314	down
Capzb	2.79E-05	2.569545031	down
Zfp276	2.79E-05	1.735865235	up
Cyth4	2.80E-05	9.690379143	up
Anapc5	2.80E-05	5.241995812	down
Cnpy3	2.80E-05	2.935933113	down
1700024K14Rik	2.81E-05	1.537546992	up
Rangap1	2.81E-05	3.907580376	down
Myl4	2.81E-05	2.878082514	up
Sdc1	2.81E-05	3.508153439	down
Gpr108	2.81E-05	1.836807251	down
LOC432730	0.000028144	3.928883314	down
E230020D15Rik	2.82E-05	3.314997673	up
Prkar2b	2.83E-05	1.900593281	down
Adk	2.83E-05	6.292619705	down
C80879	2.83E-05	1.891337514	up
Nsdhl	2.83E-05	4.994202614	down
Thbs1	2.84E-05	5.315876007	down
Traf7	2.84E-05	2.057048321	up
Ctss	2.84E-05	7.732981682	up
LOC382091	2.84E-05	2.05813098	down
Lpar2	2.84E-05	2.406290531	up
Psip1	2.84E-05	5.23514986	down
Hp	2.85E-05	1.688666344	up
LOC100038993	2.85E-05	3.241112471	down
Ctsa	2.86E-05	3.247160435	down
Csnk1e	2.86E-05	2.344733954	down
Usp1	2.87E-05	2.713627338	down
Atp5l	2.87E-05	1.816922784	down
Pcnt	2.88E-05	1.886447549	up
scl0002906.1_58	2.88E-05	3.952043057	down
Tmed4	2.88E-05	4.630799294	down
Tpi1	2.88E-05	4.450827122	down
Gtpbp10	2.91E-05	4.138234615	down
Clns1a	2.92E-05	5.645679951	down
9530068E07Rik	2.93E-05	3.322604418	down
Slmap	2.93E-05	3.331158161	down
Slc35b3	2.95E-05	3.153701067	down
Cdc37l1	2.95E-05	2.378768444	down
Nfatc3	2.96E-05	2.062367678	up
Pnpla8	2.97E-05	3.094823122	down
Sec62	2.97E-05	2.038259029	up
B4galt3	2.97E-05	2.832778454	down
LOC545750	2.97E-05	2.744885922	down
Mat2a	2.97E-05	11.0291481	down
Slc38a2	2.97E-05	1.672430515	up
Ptpra	2.99E-05	1.886648774	up
scl0001487.1_50	2.99E-05	5.337049484	down

LOC100046025	3.01E-05	3.741459608	down
Smarcc1	3.01E-05	1.68176949	down
LOC667005	3.01E-05	9.028515816	down
Arl13b	3.03E-05	2.598559856	down
Tyw1	3.05E-05	2.425904274	up
Mlx	3.05E-05	2.359501362	up
Fhl1	3.05E-05	1.621378303	down
Golt1b	3.05E-05	2.930775166	down
Cmtm5	3.09E-05	4.380111694	down
Wipi1	3.09E-05	3.950438023	down
LOC667269	0.000030934	2.614979267	up
Trappc3	3.11E-05	5.257313252	down
LOC226486	3.13E-05	3.338392019	down
Wdr82	3.14E-05	5.608211517	down
Alox5ap	3.16E-05	2.468556643	up
Mtap1b	3.17E-05	1.969672322	down
Hapln1	3.19E-05	6.735909462	down
Mrpl53	3.19E-05	2.2627213	down
Atp6v1c1	0.000032318	1.885682225	down
Dchs1	3.24E-05	3.529207468	up
Nt5dc3	3.25E-05	2.013053179	down
Insig1	3.31E-05	3.23274374	down
Atg2a	3.33E-05	2.355898142	up
Ctdsp2	3.33E-05	2.632240772	up
Drg1	3.33E-05	4.585217953	down
LOC100046087	3.33E-05	5.201648712	up
3110047M12Rik	3.34E-05	3.145421982	down
AI593442	3.35E-05	2.21384573	up
Ccnc	3.35E-05	2.133722782	down
Elovl1	3.35E-05	2.906730413	down
Gabarapl2	3.35E-05	3.902710199	up
Khdrbs1	3.35E-05	3.750445128	down
Ms4a6c	3.35E-05	1.980038524	up
S100a9	3.35E-05	2.608605862	up
Myef2	3.36E-05	4.044755459	down
B3gnt8	3.37E-05	1.630660534	up
EG382843	3.37E-05	1.865279794	up
E2f6	3.38E-05	3.362100363	down
Mbnl1	3.38E-05	1.567408085	down
Exoc1	3.39E-05	2.457518816	down
Sgk3	0.000034189	2.827445507	down
Pex11c	3.42E-05	1.514232874	up
Clk4	3.43E-05	11.9104557	down
1300018I17Rik	3.43E-05	2.145716429	up
Aspscr1	3.47E-05	1.595929027	down
Tnc	3.47E-05	1.869061589	down
Adra2a	3.48E-05	2.713639736	up
D10Ertd610e	3.50E-05	4.690755367	down
LOC674888	3.50E-05	1.599556923	up
Pgam2	3.50E-05	3.395617724	up
Dok1	3.52E-05	2.811432123	down
LOC380637	3.52E-05	2.693228722	up

Ms4a6d	3.53E-05	9.758406639	up
LOC382061	0.000035331	2.40476656	up
Wdr45l	0.000035331	3.310341358	down
Plekhg5	0.000035504	2.338508606	up
A830007P12Rik	3.55E-05	2.419266701	up
Zc3hc1	0.000035933	4.774459839	down
Flot1	3.60E-05	2.07884407	down
Rc3h2	3.60E-05	1.902869225	up
Cd9	3.62E-05	2.94108367	down
LOC100048526	3.62E-05	1.679495454	up
Ranbp3	3.62E-05	3.120012283	down
Parp12	3.67E-05	1.8640939	up
2610101N10Rik	3.68E-05	5.288698196	down
Edg8	3.68E-05	1.852172494	down
Mtmr2	3.68E-05	4.74108696	down
P2ry1	3.68E-05	2.169948816	down
Akap12	3.71E-05	1.613247156	down
EG229879	3.71E-05	1.767364025	up
Sumo3	3.71E-05	7.512158871	down
Tle2	3.71E-05	2.872522831	up
BC030863	3.72E-05	2.173691511	up
Fcgr4	3.73E-05	5.048808575	up
D330001F17Rik	3.73E-05	1.832918167	up
scl0002124.1_39	3.73E-05	1.514492869	down
LOC381996	3.75E-05	2.130673409	down
Trmu	3.75E-05	2.42283392	down
Srd5a3	3.81E-05	2.024572849	down
3930401E15Rik	3.84E-05	3.820165634	down
Prkd2	3.84E-05	2.045428753	up
5730508B09Rik	0.000038552	2.213041544	down
Ptx3	0.00003861	2.430686712	down
Snx24	0.00003861	1.611333132	down
2010205J10Rik	3.86E-05	2.520755291	up
A230106M15Rik	3.88E-05	2.823865414	up
5730453I16Rik	3.88E-05	3.522815466	down
LOC627985	3.89E-05	2.091299772	down
Rab6	3.89E-05	4.517389774	down
Arnt	0.000039093	2.264725447	up
Ppm1a	3.92E-05	4.354636669	down
LOC100043209	3.93E-05	2.027000427	down
Rfc2	3.95E-05	2.749675274	down
Sec24d	3.95E-05	3.02913022	down
Anapc1	3.96E-05	1.925601721	up
Glrx3	3.96E-05	1.531902909	up
Npr3	3.96E-05	2.042585135	down
Rock2	3.96E-05	2.284775257	down
C130066G14Rik	0.000039703	1.663102508	up
Cluap1	3.98E-05	2.32538867	down
9830167H18Rik	3.98E-05	1.837395668	down
Naca	0.000040091	4.634916306	down
AU020206	4.01E-05	2.06699872	up
C1qa	4.01E-05	6.321131229	up

2610202C22Rik	0.000040234	2.063946009	up
Nfkbia	4.04E-05	2.009624243	up
Sgk3	4.05E-05	2.786330223	down
Gnas	4.05E-05	5.683738709	down
C130086J11Rik	4.06E-05	1.635486603	up
Zc3h14	4.06E-05	4.442071915	down
Bfar	4.12E-05	3.680678129	down
Hectd2	4.12E-05	1.818490744	down
Lypla1	4.12E-05	4.616435051	down
Smarca4	4.12E-05	2.639749765	up
Uhrf2	4.12E-05	5.452251911	down
Prg4	4.13E-05	1.597024322	down
Sfrs10	4.14E-05	12.64330864	down
Asph	0.00004143	2.169843674	down
Fn1	0.00004143	3.997449637	down
Nubp2	0.00004143	2.860342741	down
Chd1l	4.15E-05	1.556504011	down
Moxd1	4.15E-05	3.239943504	down
2810408A11Rik	0.000041518	2.954569817	up
Bex1	0.000041518	1.90403223	up
Lrrfip2	0.000041518	2.232184649	down
Zfp592	0.000041518	1.821945787	up
Fyco1	4.15E-05	1.911411285	up
Mfap2	4.15E-05	3.709034443	down
Igf2bp2	4.17E-05	1.925333619	down
2410042D21Rik	4.19E-05	3.766563654	down
AI480653	4.19E-05	1.903862119	down
Csnk2a2	4.19E-05	1.895500898	up
Obsl1	4.19E-05	1.735131741	up
Thyn1	4.19E-05	1.776072979	up
Tox	4.19E-05	4.584356308	down
scl0001419.1_32	0.000042008	2.267399073	down
Matn4	4.25E-05	21.17943192	down
LOC100039227	4.25E-05	1.774461389	down
Tsc22d1	4.27E-05	16.97862816	down
D7Ertd715e	4.28E-05	2.690426111	down
Ppp1cc	4.29E-05	3.024160624	down
1190002N15Rik	4.29E-05	4.486179829	down
Bptf	4.29E-05	3.892836094	up
Alg3	4.32E-05	1.955567122	down
Ddx26b	4.32E-05	2.138787985	down
9030625A04Rik	4.33E-05	2.277412415	down
1700030C10Rik	4.36E-05	2.162940025	up
Gna12	4.37E-05	2.485063076	down
Myo1e	4.37E-05	2.609994412	up
9430088P09Rik	4.38E-05	2.260219574	down
B230339C08Rik	4.38E-05	2.283774853	up
C8b	4.38E-05	3.325871229	down
Dcn	4.38E-05	6.7773633	down
Dedd2	4.38E-05	1.955394745	up
Gtpbp10	4.38E-05	3.97743988	down
Polh	4.38E-05	2.805636644	down

Camk1g	4.42E-05	2.216525078	down
BC005764	4.46E-05	2.138457537	up
Tbxas1	4.47E-05	6.862919331	up
S100a8	4.49E-05	2.954621077	up
AI848100	4.50E-05	3.528762817	down
Cep170	4.50E-05	2.833632231	down
Rps6kb1	4.50E-05	2.721683979	down
Ednra	0.000045378	1.612930536	up
Fem1c	0.000045378	1.696869135	down
LOC269251	0.000045378	2.250858784	up
Tes	0.000045378	5.683325291	down
Ift140	4.58E-05	2.298628807	up
Saps3	4.58E-05	2.561685085	up
2810401C09Rik	4.58E-05	1.798041463	up
Ubr1	4.58E-05	2.174515486	down
Smcx	4.60E-05	4.660027981	up
B3gnt9	4.62E-05	1.84432447	up
Sfxn2	4.62E-05	1.836755395	down
Clasp2	4.63E-05	2.483433247	down
Pex3	4.66E-05	2.427631855	down
Baz2a	0.000046618	1.844004154	up
Nkx6-2	0.000046618	1.737278938	up
Tmem168	0.000046618	2.116677046	down
E130014J05Rik	4.69E-05	2.303111315	up
Sepx1	4.69E-05	2.113198042	up
Ccl3	4.70E-05	2.247771263	up
Cyp20a1	4.70E-05	2.879655123	down
Zmynd11	4.71E-05	3.239306688	down
Sfrs10	4.76E-05	22.50256729	down
Slc12a4	4.80E-05	1.859230638	up
Ube2e1	4.80E-05	3.91310215	down
Atxn7l3	4.81E-05	2.88219285	up
Nudt19	4.81E-05	3.006002665	down
Bat4	4.84E-05	2.089825153	down
Mpv17	0.000048466	2.256426573	down
Mobkl3	4.86E-05	4.701019764	down
Snx2	4.90E-05	6.765829086	down
Fsd1	4.91E-05	1.707734466	up
Ttc9c	4.93E-05	2.534919977	down
AK157302	4.94E-05	1.885556102	down
Ccndbp1	4.94E-05	5.522270203	down
Fgfr1op	4.94E-05	3.044998169	down
LOC100047012	4.94E-05	6.42723465	down
Tnrc6c	4.96E-05	1.789737582	up
B430305P08Rik	4.97E-05	1.688486457	up
Icam2	4.97E-05	2.936601162	up
1110007L15Rik	4.97E-05	1.652600288	down
Actn4	5.05E-05	2.610746384	down
Tgif1	5.07E-05	3.366029501	down
Acad8	0.000050798	3.806711912	down
Naalad2	5.09E-05	2.319501638	up
9430087C24Rik	5.10E-05	2.248124361	up

Peci	5.10E-05	3.512191534	up
Tprgl	5.10E-05	3.196787596	down
Brd2	5.11E-05	1.940654516	down
Cbfb	5.11E-05	3.276631594	down
Tbx1	5.11E-05	2.809188366	up
Ylpm1	5.11E-05	1.884726048	up
Brd8	5.12E-05	1.978004575	down
Cdk8	5.17E-05	1.871616602	down
Adcy7	5.21E-05	1.924390674	up
1700041B20Rik	5.27E-05	1.850744247	down
Mr1	5.27E-05	2.353144884	up
Bsdc1	5.29E-05	2.169983625	up
E330011I20Rik	5.29E-05	3.363369465	down
C330006D17Rik	0.000052935	2.935462952	up
BC021381	5.31E-05	2.085487843	up
Mapk9	5.31E-05	4.267343521	down
Mfsd11	5.31E-05	2.42723465	down
C030040A22Rik	5.32E-05	2.208784342	up
Ctbp1	5.32E-05	1.905156732	up
EG668830	5.32E-05	1.673520804	up
Sbno2	5.36E-05	1.640567541	up
LOC100047223	5.38E-05	2.176020622	down
A330021D07Rik	0.000054138	4.292908669	down
Acot2	0.000054138	2.404225349	down
Cln6	0.000054138	2.33219409	up
Epha3	0.000054138	4.076158524	down
LOC100045882	0.000054138	1.634979725	up
LOC619973	0.000054138	2.362172365	down
Mfsd2	0.000054138	2.035990238	down
Pin1l	0.000054138	2.027514219	up
Rps4y2	0.000054138	2.743528128	down
Sepp1	0.000054138	6.204881191	up
Ascc3l1	0.000054269	2.214868546	up
LOC100048313	0.000054269	1.877420306	up
Xpo4	0.000054269	2.114598036	down
Cdc20	5.45E-05	6.218059063	down
Stard9	5.46E-05	2.167569161	up
Mtap1s	5.47E-05	2.235272646	up
Zcchc3	5.47E-05	3.209930658	down
Zfp1	5.48E-05	2.283190489	up
Eftud2	5.49E-05	1.82590878	up
Gtl3	5.50E-05	2.00747633	up
LOC639554	5.50E-05	2.82462883	down
Melk	5.50E-05	3.728048325	down
Rab18	5.50E-05	3.119390249	down
Ctps2	5.52E-05	7.574419975	down
E2f6	5.52E-05	3.237102509	down
Pdcd5	5.52E-05	2.408194065	down
Ranbp6	5.52E-05	2.431586504	down
Rspry1	5.52E-05	2.347830057	up
A430088C08Rik	5.54E-05	2.430822134	up
Pspc1	5.54E-05	1.834138274	up

Nsun5	5.57E-05	2.757062674	down
Calcoco1	5.60E-05	2.164650917	up
Acad8	5.60E-05	3.208378077	down
8030401N12Rik	5.60E-05	1.867398381	up
Cbll1	5.61E-05	2.064683437	up
Rsrc2	5.61E-05	3.153092146	down
Tnrc6c	5.62E-05	2.169309616	up
Wtap	5.62E-05	2.442561626	up
Skp2	5.62E-05	6.384583473	down
Snap23	5.62E-05	2.974595785	down
Gale	5.63E-05	3.049463987	down
Hsd17b10	5.63E-05	2.501660109	up
Mrpl52	5.63E-05	2.060772419	up
Cdc37l1	5.64E-05	2.50075531	down
Dynll2	5.64E-05	5.332748413	down
Zdhhc1	5.64E-05	2.072454929	up
LOC100047936	0.000056458	1.831988096	up
Fcrls	5.65E-05	17.65731239	up
4933407L23Rik	5.66E-05	2.682318687	up
Blvrb	5.66E-05	2.11733222	up
Ggcx	5.67E-05	2.449159384	down
Nras	5.69E-05	2.501082182	up
Ndufs3	5.74E-05	3.196403027	down
Dmxl1	5.74E-05	2.709820271	down
M6pr	5.77E-05	2.128665686	down
Fndc5	5.82E-05	2.160599947	up
2700033B16Rik	5.84E-05	1.644810796	up
9630032J03Rik	5.84E-05	1.774509311	up
Aytl2	5.86E-05	2.303926706	up
A930007F16Rik	5.86E-05	2.026841164	up
Fgf13	5.86E-05	2.250362873	down
Rfxank	0.000059228	2.077599526	up
scl0002177.1_17	5.95E-05	1.711463332	down
Dcn	5.96E-05	3.202011585	down
Trpc4ap	6.00E-05	3.307366371	down
C730026O12Rik	6.02E-05	1.731827378	up
LOC234360	6.03E-05	4.393483639	down
LOC100047963	6.04E-05	2.117429018	up
Pak2	6.04E-05	2.433626175	down
Srebf2	6.04E-05	2.878751755	up
1810007M14Rik	0.000060634	3.691850185	down
Bai2	0.000060634	2.246994257	up
Pdzd11	0.000060634	3.380865574	down
Rabggtb	0.000060634	2.747635126	down
2610524N02Rik	0.000060671	2.638593197	down
Ube2j2	0.000060671	2.159637451	down
scl000171.1_4	6.09E-05	2.088298798	down
Gorasp2	6.13E-05	3.213990212	down
Ubl5	0.000061343	2.463537931	down
D830012I16Rik	6.19E-05	3.561058521	up
Rab5b	6.20E-05	1.733808637	down
Armc8	6.22E-05	3.523648739	down

Fdx1l	6.23E-05	2.453845024	up
Gas5	6.25E-05	2.00115037	down
3110003A17Rik	6.27E-05	3.366592646	up
LOC666025	6.27E-05	3.047303438	down
Mrpl22	6.27E-05	1.610602021	down
Pfkm	6.27E-05	4.260520458	down
EG240110	6.31E-05	2.760208845	up
Tmem66	6.31E-05	5.062771797	down
Psmd13	0.000063331	7.706672669	down
Tgfbr1	0.000063331	3.13123703	down
D930020N02Rik	6.34E-05	2.03296566	up
9830115L13Rik	6.37E-05	1.775632381	up
Ccdc115	6.37E-05	3.826667309	down
Usp22	6.37E-05	2.396869659	down
Rab24	6.41E-05	2.46876359	down
Capns1	6.42E-05	2.023786545	down
Fech	6.42E-05	2.261281252	down
Gm1818	6.42E-05	2.02170682	down
LOC637353	0.000064277	4.044147015	down
A230057E24Rik	6.46E-05	1.669480562	up
Gch1	6.55E-05	1.952455998	up
LOC100041502	6.55E-05	2.512696981	up
Ndufv2	6.55E-05	3.21805501	up
Zdhhc17	6.57E-05	2.077921391	down
Dok1	6.58E-05	2.928308487	down
2610019A05Rik	6.59E-05	1.767435074	up
LOC100046623	6.59E-05	6.133660793	down
Usf1	6.60E-05	2.526470423	down
Rab11a	6.62E-05	3.261560679	down
Lhfp	0.000066398	3.460046768	down
Rab3il1	6.64E-05	1.988007069	down
1810055E12Rik	6.65E-05	2.839559555	down
Aif1	6.65E-05	5.361550808	up
LOC385699	6.65E-05	2.278853893	up
Nfatc3	6.65E-05	2.160465241	up
Klf15	6.65E-05	1.65427351	down
Lama2	6.65E-05	1.813099623	down
Trim56	6.65E-05	2.105467081	up
EG432466	6.67E-05	1.7499125	down
Cyp4f13	6.67E-05	3.060883999	up
Gm266	6.67E-05	1.854758859	down
2310051N18Rik	6.70E-05	2.077365875	up
Cops4	6.71E-05	4.254882813	down
Pgls	6.71E-05	2.099420548	up
LOC100046322	6.72E-05	2.145213366	down
2610200G18Rik	6.72E-05	2.339172602	down
2900034E22Rik	6.72E-05	2.418410778	up
Ivns1abp	6.72E-05	5.763780594	down
Cnot2	6.75E-05	4.808637142	down
D130060L11Rik	6.75E-05	1.572817087	up
2700029L08Rik	6.78E-05	2.538516045	up
Cyp20a1	6.78E-05	3.088886023	down

H2-T23	6.78E-05	1.710852146	up
Lrmp	6.78E-05	2.638993025	up
Agtr1a	6.80E-05	2.726255655	down
Prkd3	6.80E-05	2.267865658	down
Sqle	6.80E-05	1.627150297	down
Tmpo	0.000068042	4.569718361	down
D430030F08Rik	6.82E-05	2.404030085	up
Eif4ebp3	0.000068255	1.983619571	up
Mtch2	6.83E-05	6.156559944	down
Sulf2	6.83E-05	2.373995304	up
P2ry6	6.85E-05	6.581582546	up
Kat2b	6.86E-05	1.971818686	down
Jrkl	6.86E-05	1.58117497	down
A930015N15Rik	6.88E-05	1.846510887	up
Decr1	6.88E-05	1.583391547	up
Net1	6.88E-05	9.44861126	down
Fnbp4	6.88E-05	1.621601224	down
5033430I15Rik	6.88E-05	1.874239564	down
Cyba	6.88E-05	2.135558605	up
Tspan18	6.88E-05	2.772142649	down
AI597468	6.95E-05	5.438621998	down
Tmem38a	6.95E-05	2.349522114	up
Tspyl2	6.95E-05	1.621193528	up
C030038J10Rik	6.96E-05	2.040082216	down
Gnb1	6.96E-05	3.277168274	down
Wdr32	6.96E-05	2.933605433	down
B3galnt2	7.02E-05	2.113152027	down
Foxred1	7.04E-05	2.010187388	down
Ttc3	7.04E-05	3.968136787	down
Emb	7.07E-05	1.961876035	down
4833425P12Rik	7.10E-05	2.14288187	up
LOC100046898	7.10E-05	2.59269166	down
Col2a1	7.11E-05	8.106253624	down
Oprs1	7.11E-05	2.871074438	down
Cr1l	7.15E-05	2.998526335	down
Ap2b1	7.18E-05	1.683663964	down
Crtc1	7.18E-05	2.469668865	up
1110008J03Rik	7.19E-05	2.30757618	down
Inpp5b	7.20E-05	1.838120103	up
Mageh1	7.20E-05	7.174389839	down
Tspan6	7.20E-05	3.439257383	down
Flnb	7.25E-05	2.532634735	down
Gpsn2	7.25E-05	2.485758781	up
scl000972.1_0	7.25E-05	2.047077894	up
2410091C18Rik	7.26E-05	2.369493246	down
EG433643	7.27E-05	2.151795864	down
Helz	7.27E-05	2.861902237	up
Hps1	7.27E-05	1.93018949	up
Snrpa	7.27E-05	8.081143379	down
Grk5	7.28E-05	1.805803418	down
Bcl2l12	7.29E-05	2.280676603	down
Srgn	7.29E-05	2.236761332	up

Zfp143	7.30E-05	1.934387803	down
LOC435912	7.30E-05	2.963234901	up
Myo9b	7.30E-05	2.22285223	up
scl0002064.1_2	7.30E-05	1.912297845	down
Ankrd27	7.31E-05	2.332244158	down
Snx5	7.31E-05	3.003176451	up
Ptpla	7.32E-05	1.919571996	down
3830432E14Rik	7.34E-05	3.032339573	down
9630027A13Rik	7.34E-05	2.050425291	up
Arfgap2	7.34E-05	2.030695438	down
Dap3	7.34E-05	2.927265406	down
Rars2	7.34E-05	2.62176156	down
C230053E11Rik	7.35E-05	3.408675194	up
2810407C02Rik	7.36E-05	3.824454069	down
Mccc2	7.39E-05	2.450114727	down
Wdr37	7.39E-05	2.707296133	down
LOC100044363	0.000073919	1.934279084	down
EG629383	0.000073934	4.711193562	down
Wipi1	7.45E-05	2.793698072	down
2410005O16Rik	7.46E-05	1.85773778	up
5330431K02Rik	7.46E-05	2.714342833	up
Scp2	7.46E-05	1.973301888	down
Gabt1	7.51E-05	1.565253496	up
4631416L12Rik	7.52E-05	2.325555563	down
Gtf2h4	7.52E-05	1.802882552	up
scl0003749.1_1	7.54E-05	1.678581595	down
2700078E11Rik	7.56E-05	3.013702393	down
Acaa2	7.56E-05	3.110505104	down
Acadl	7.56E-05	3.582536697	down
LOC100046650	7.56E-05	1.857588172	down
Chi3l1	7.56E-05	2.813188076	down
B930066N23Rik	7.58E-05	1.934273124	down
Vasn	7.58E-05	1.751005173	down
0610037L13Rik	7.59E-05	2.36140585	down
Thsd7b	7.60E-05	1.682179809	up
5930404A08Rik	7.61E-05	1.807741284	up
Acsf3	7.61E-05	1.777652144	up
Rbm5	7.62E-05	3.300074101	up
1110038G02Rik	7.63E-05	2.559972048	down
4833418A01Rik	7.65E-05	2.12139225	down
Capn6	7.65E-05	2.12635541	down
Dync1h1	7.65E-05	2.999501467	up
EG235855	7.65E-05	2.109173298	down
Parp1	7.65E-05	3.559139729	down
Psat1	7.65E-05	2.637450457	down
Tcf12	7.65E-05	1.968023896	down
Tjap1	7.65E-05	1.920527577	up
Ncoa4	7.66E-05	2.128906727	down
Fgfr3	7.67E-05	1.774785996	down
Lass2	7.67E-05	6.449664116	down
LOC384836	7.68E-05	2.756736517	up
Foxp2	7.70E-05	2.867068768	down

6330403E01Rik	0.000077601	2.119986773	up
Fut8	7.76E-05	1.797354579	down
Slc30a5	7.76E-05	3.024980068	down
Aldoc	7.79E-05	2.084366083	down
E030024L06Rik	7.79E-05	1.86097908	up
Nola2	7.79E-05	3.155969381	down
Txndc5	7.79E-05	3.610878468	down
Ctdsp1	7.83E-05	2.078402042	up
Ap4m1	7.85E-05	3.282339096	down
Asph	7.85E-05	4.216923714	down
BC030046	7.85E-05	1.69511807	up
LOC386027	7.85E-05	1.682624698	up
Upf1	7.85E-05	2.72619462	up
Sirt6	7.86E-05	2.145502091	down
Laptm4b	7.89E-05	2.49363327	up
Xpot	0.00007928	2.980075121	down
Ubtd2	7.93E-05	2.986867905	down
Srpk1	7.96E-05	1.775823593	up
D430006A07Rik	8.01E-05	3.021985769	up
Notch3	8.02E-05	2.79549861	up
Git1	8.04E-05	1.942364931	up
Rif1	8.04E-05	2.512672663	down
Rnf213	8.04E-05	3.163559914	up
LOC100047393	8.07E-05	3.321138144	down
Fzr1	8.08E-05	1.616291642	down
Bmp5	8.10E-05	1.953491926	down
Tm6sf1	8.10E-05	2.098410606	down
Tmem219	8.10E-05	1.66725719	up
Slco2b1	8.12E-05	8.74333477	up
Mks1	8.17E-05	1.736744881	up
Car3	8.17E-05	2.797692776	down
Cyb5r4	8.17E-05	2.823809862	down
Hsp90ab1	8.19E-05	3.361030817	down
Gtpbp10	8.23E-05	2.405654192	down
4Cytl1	8.26E-05	12.69908905	down
Dkc1	8.26E-05	1.835830331	up
Emr1	8.26E-05	15.34803867	up
Fin15	8.26E-05	3.364711523	down
Narfl	8.26E-05	1.778761506	down
Zfp827	8.26E-05	2.582318783	up
Fam149b	8.26E-05	3.188904762	down
LOC630401	8.30E-05	1.91366148	up
Metapl1	8.30E-05	2.135079384	up
LOC100048622	8.35E-05	2.975434542	down
C1qtnf3	8.35E-05	7.059156895	down
Fzd9	8.35E-05	1.93507576	down
Pmpcb	8.35E-05	1.697584868	up
Wbscr16	8.35E-05	1.689199805	up
Rp23-297j14.5	8.35E-05	2.089379311	up
Gramd3	8.37E-05	3.307068825	down
Cdk2	8.44E-05	4.913955212	down
Ddx39	8.44E-05	3.194382668	up

Eapp	8.44E-05	2.126230717	up
Scp2	8.51E-05	2.563202143	down
Cnih	8.52E-05	5.531797886	down
Pde7a	8.59E-05	4.084401608	down
Eif3s8	8.68E-05	2.311022997	up
Erbb2ip	8.71E-05	2.336574078	down
Adamts7	8.73E-05	2.246266842	up
5730507N06Rik	8.74E-05	1.706574917	up
Acaa2	8.75E-05	3.990022898	down
E230029F23Rik	8.75E-05	11.05878258	up
Kcnk1	8.77E-05	1.755455256	down
Ing4	8.77E-05	1.853547335	up
Hmga1	8.79E-05	2.260566473	up
Snrpa	8.79E-05	8.390077591	down
BC023835	8.81E-05	2.025957108	down
Eya1	8.83E-05	3.289761305	down
Ppig	8.84E-05	2.570088148	down
Rrm2	8.86E-05	2.764849901	down
E030003O11Rik	8.87E-05	1.572151542	up
Mrc1	8.87E-05	9.513801575	up
Tuba3b	8.87E-05	1.886989355	up
D030044M21Rik	0.00008907	1.873750329	up
Rfc5	8.92E-05	2.207399607	down
Papd4	8.92E-05	5.102193356	down
4833442A19Rik	8.99E-05	1.598705053	up
Idh3g	9.03E-05	3.431070328	down
Muc1	9.03E-05	1.912362933	up
Pdzrn4	9.03E-05	1.600646615	down
Zim1	9.03E-05	1.8288486	down
LOC100044363	9.08E-05	3.248797894	down
Rint1	9.13E-05	2.064455509	down
LOC545332	9.15E-05	2.463353634	down
A730050C11Rik	9.16E-05	1.921604276	down
LOC545369	9.16E-05	1.953592658	up
Vkorc1	9.17E-05	2.572053909	down
Plekha1	9.24E-05	1.997643352	down
AI413582	9.25E-05	1.682733178	up
Ppox	9.26E-05	2.119540691	down
1700052O22Rik	9.27E-05	1.758883834	up
Kat2a	9.27E-05	2.078574419	up
Mgea5	9.27E-05	2.379073143	up
Wdr45l	9.27E-05	2.206906557	down
Ccdc80	9.28E-05	5.633236885	down
Stx18	9.28E-05	2.186121464	down
Eif2ak1	9.30E-05	1.69184041	up
Gm166	9.30E-05	2.082437515	up
EG432681	9.32E-05	1.565901279	down
EG628040	9.32E-05	2.528613329	down
Gsk3b	9.32E-05	2.113789797	up
LOC382058	9.32E-05	1.553151608	down
LOC100044190	0.000093329	2.144710779	up
Polr2a	0.000093329	2.61126256	up

Ofd1	9.42E-05	2.403941393	up
Tmed3	9.46E-05	2.500232935	down
Il17d	9.48E-05	1.621251226	down
Zfp248	9.48E-05	1.807385087	down
4933429E06Rik	9.50E-05	1.78855896	down
Hoxd13	9.53E-05	4.240774155	down
Mdm1	9.53E-05	2.247829199	down
Rsrc1	9.53E-05	1.90875876	up
A230106M20Rik	9.54E-05	1.976191759	up
Igsf10	9.54E-05	1.765522003	up
Trspap1	9.54E-05	2.104293346	up
Fbxl18	0.000095595	1.78158164	up
Lbp	9.61E-05	1.747176886	up
Sphk1	9.66E-05	2.671342373	down
6720467C03Rik	9.69E-05	3.01122117	down
Il6st	9.69E-05	2.309660196	up
Map4k4	9.71E-05	1.717309713	up
Nfatc4	0.000097194	1.957414746	up
D730006F06Rik	9.73E-05	1.686440945	up
2310021P13Rik	9.75E-05	5.131253719	up
Gpn1	0.000097552	1.874037266	down
2900073C17Rik	9.76E-05	3.825241804	up
Dpp6	9.77E-05	2.66005373	up
Znhit6	9.77E-05	1.624250531	down
Samd1	9.78E-05	2.003146172	up
D2Ertd173e	9.79E-05	2.240970135	up
9130404D08Rik	0.000098168	1.99350214	up
LOC100046080	0.000098168	2.327822924	down
Vasp	9.84E-05	1.56875813	up
Wdr75	9.85E-05	1.607116222	up
Gstk1	9.85E-05	1.722786427	up
8430426H19Rik	9.88E-05	1.800400019	down
Uncx	0.000099051	1.693639398	up
Zfp180	9.92E-05	1.61986649	down
Rasa3	9.94E-05	1.797145128	up
Utrn	9.95E-05	3.522297144	down
Elk3	9.97E-05	2.433782816	down
Gulp1	9.98E-05	5.826993942	down
LOC100043919	1.00E-04	1.813572764	up
Pvrl1	0.000100283	1.91716814	up
Tceal3	0.000100434	2.379035711	down
5730557L09Rik	0.000100557	1.955601096	up
Nqo1	0.000100883	2.024753332	down
Dyrk1a	0.000101054	1.69041121	up
4833416I09Rik	0.000101672	2.399401903	down
Crebzf	0.000101672	1.833200216	down
Cdc20	0.000101716	7.528128624	down
Igtp	0.000101716	1.856918097	up
Saps3	0.000101716	1.866085053	up
Rcl1	0.000102074	3.034357309	down
Ing4	0.000102202	2.246471405	up
Hspb8	0.000102357	1.629815936	up

Spnb1	0.000102357	2.270959854	up
Plac8	0.000102556	1.642151475	up
scl0002720.1_68	0.000102643	1.88692534	down
Csf1r	0.000102652	10.13853359	up
Gusb	0.000102689	2.571530342	down
Tmem129	0.000102839	2.046130419	down
LOC100047868	0.000103024	1.61555779	up
Pcdhb22	0.000103211	1.942202568	up
Fzd8	0.000103266	1.892960548	down
LOC631204	0.000103266	6.606936932	down
Znrf1	0.000103266	2.471314669	up
4930431J19Rik	0.00010399	2.594918966	down
Mdm1	0.000104214	2.260356188	down
Bach1	0.000104515	2.485815525	up
B930096F20Rik	0.000104582	2.659277678	up
Cd93	0.000104582	4.224112511	up
D130063H01Rik	0.000104582	2.494008064	up
E330024J20Rik	0.000104582	2.55798316	up
Edem2	0.000104582	3.526446343	down
Igf2bp3	0.000104629	1.510372758	up
Idh1	0.000105065	4.234066963	down
Prdx6	0.000105065	1.883167625	up
Isyna1	0.000105451	1.97421813	up
Lig1	0.000105451	2.572464228	down
Nab1	0.00010555	2.304262161	down
LOC100042952	0.000105608	2.865468979	down
Zfp212	0.000105608	1.513079166	down
Tnfrsf21	0.000105662	1.828891873	down
Slc6a15	0.00010601	3.621177435	down
Fut10	0.000106063	1.557229281	up
Rbm4	0.000106063	4.00559473	down
D030041G16Rik	0.000106813	2.153964996	down
Huwe1	0.000106906	2.23468709	up
Baz1a	0.000106921	2.711282492	down
Ndufs2	0.000107273	2.680295229	down
Chka	0.000107911	1.773676992	up
Mthfd1	0.000108067	2.853935003	down
Pycr1	0.000108067	2.10672164	down
Rps10	0.000108067	1.536292553	down
Tubb5	0.000108141	9.476763725	down
Nudt18	0.000108753	1.646762729	up
Mpdz	0.000108887	2.104254484	down
Pcgf6	0.000109145	2.215099096	down
Spc25	0.000109145	2.462505341	down
Ccdc47	0.000109372	1.841088176	down
Gemin4	0.000109372	2.239537478	down
Zfp334	0.000109372	1.533767939	up
Tmem159	0.000110355	2.281646967	down
scl0004175.1_57	0.000110532	3.330337286	down
LOC333765	0.000111028	2.09265995	up
Rhod	0.000111028	2.27553916	down
Srpk2	0.000111028	2.011079073	down

Zmynd11	0.000111028	1.851743341	down
5730437N04Rik	0.000111271	2.95377183	down
Actn4	0.000111584	7.594707966	down
Taf9b	0.000111718	2.151478767	down
9530039I19Rik	0.000111951	1.780998826	up
Atp5e	0.000111951	2.15704608	up
Cacna2d3	0.000111989	2.231243849	up
LOC100039623	0.000112217	1.747301102	up
LOC630936	0.000112217	5.00859642	down
Rnf167	0.000112217	2.502700806	down
Uchl5ip	0.00011241	2.136801243	up
Pxdn	0.000113226	3.044472218	down
Atp2a1	0.000113394	1.950869799	up
Slc29a1	0.000113689	1.97650373	down
Acp2	0.000113727	1.811573982	up
Spnb2	0.000113727	2.274885654	down
Arl6ip5	0.000114638	5.241146088	down
Gpr27	0.000114638	1.85830164	down
Cdca8	0.000114789	3.678204536	down
Asns	0.000114934	3.113725662	down
Ireb2	0.000115267	2.222700357	down
Pwp2	0.000115381	1.874183059	down
Gcnt1	0.000115537	2.185174465	down
Prpf3	0.000115537	2.416350603	down
Scnm1	0.000115652	2.153906584	up
2610319H10Rik	0.00011573	2.622207642	up
Ttyh2	0.000115826	2.502031326	up
Sfrs5	0.000115965	8.224294663	down
Agfg1	0.000116049	4.517463207	down
Chst5	0.000116142	1.624899149	down
LOC194642	0.000116142	1.706556439	down
Tmod3	0.000116142	1.510240793	up
LOC100039001	0.000116467	2.209457159	up
Aof1	0.000116602	1.677131534	down
Stau2	0.000116672	3.744575501	down
Hba-x	0.000116725	12.84805584	up
LOC330553	0.000116815	1.928778887	up
2310003C23Rik	0.000117151	3.682601929	down
Jmjd1b	0.000117151	2.84370327	up
Ppm2c	0.000117151	2.620201826	down
Ubtf	0.000117151	3.054538012	up
Pnkd	0.000117877	1.650520682	down
scl0002624.1_576	0.000117877	1.930971861	down
Top3b	0.000117877	1.889467001	up
D130054E02Rik	0.000117917	2.106685638	up
Pitpnm1	0.000118036	2.619600058	up
Bdh2	0.000118334	1.987216711	up
Dus3l	0.000118334	1.818896174	down
Leprel1	0.000118398	2.813713551	down
Foxj3	0.000118839	1.898161292	down
Sox11	0.000118951	3.148519754	up
Fgfr2	0.000120034	4.836047649	down

Lcp2	0.000120034	3.685230255	up
Tbx15	0.000120034	2.563603878	down
Mrpl3	0.000120142	4.859736443	down
Larp7	0.000120165	1.845251441	up
Tmem158	0.000120514	1.71411705	down
LOC100041504	0.000120518	2.635768414	up
scl000723.1_11	0.000120648	1.805446386	down
C1qc	0.000120748	9.142455101	up
Rgs9	0.00012087	2.232816219	up
Ppp1r3b	0.000121018	2.058467388	down
Serpinb6b	0.000121018	1.588743329	up
Kank1	0.000121201	5.633127213	down
Ube2g1	0.000121229	9.651836395	down
Pard3	0.000121242	1.597406507	up
Cxxc5	0.000121477	2.226545811	down
Mcm4	0.000121712	4.931772709	down
Eid2	0.000122241	3.099127054	down
Ptprk	0.000122264	2.001463413	down
Atp2b1	0.000122544	1.961512804	down
Bcl2a1b	0.000122883	3.496189833	up
Pmaip1	0.000122983	1.64503336	down
Gabbr1	0.00012307	2.014508009	up
Ap2b1	0.00012328	1.770541906	down
B230317C12Rik	0.000125099	1.888229489	down
B130020M22Rik	0.00012518	4.930659771	up
Ash2l	0.000125609	2.318566799	up
Arhgap28	0.000125647	2.059422255	down
4933401B06Rik	0.000125964	1.870440364	down
Rnf122	0.000125964	2.002115727	up
LOC234081	0.000125983	2.682436228	down
Sec14l1	0.000126182	1.908110738	up
Adfp	0.000126225	1.538419843	down
Asph	0.000126393	3.164973259	down
Snhg11	0.000126484	2.531388998	up
Ccbl2	0.00012685	2.05053997	down
Sipa1l2	0.000126959	2.191265822	up
Nsl1	0.000127036	2.034730196	down
Rpain	0.00012767	2.099730015	up
Zfp322a	0.00012767	1.960974693	up
Acad9	0.000127842	2.273583412	down
Zcchc3	0.000127945	3.07166028	down
Dcn	0.000128409	2.875388622	down
Ubxn2b	0.000128829	1.952109337	up
3110018C07Rik	0.00012913	2.153468847	up
Rps20	0.000129135	1.961827636	up
2310007H09Rik	0.000129417	1.623962998	down
Comt	0.000129417	1.879665375	down
Gtf2h1	0.000129417	2.01801014	down
Mrps18b	0.000129778	2.462709427	down
Atp8b2	0.000129782	1.712896466	down
Igfbp2	0.000129808	3.676183224	down
LOC545339	0.000129833	1.719917774	up

MGC60742	0.000129833	3.174955845	down
Sgk	0.000130024	5.802307606	down
Ctdp1	0.000130309	1.903949857	down
Mycbpap	0.000130832	2.147407293	up
LOC100048622	0.000131475	2.674855232	down
Prpf3	0.000131475	2.690169573	down
Fign	0.000131762	2.133958101	down
Xpo5	0.000131762	1.980903268	up
5730601F06Rik	0.000132154	1.877603173	down
Gbl	0.000132154	1.988775134	down
Dnase2a	0.000132285	1.547486782	up
A830080D01Rik	0.000132311	1.836452127	down
Pitx2	0.000132311	2.135507107	down
Tle1	0.000132311	1.573440909	down
Vamp3	0.000132311	2.437590838	down
Zfp462	0.000132311	3.295689821	up
Spsb4	0.000132742	1.836065888	down
Ccdc115	0.000133376	3.600524664	down
Sdccag3	0.000133376	3.617164373	down
LOC215086	0.000133431	2.081698656	up
Ints10	0.000134131	1.54038012	up
Trim25	0.00013425	3.550846815	up
Bcl7c	0.000134265	1.579469085	up
Abhd13	0.000134479	2.573749781	up
Ptpn12	0.000134998	2.747475624	down
2810026P18Rik	0.000135788	3.114598513	down
Tbc1d12	0.000135793	1.953297377	down
C1qb	0.000136514	9.200699806	up
Ethe1	0.000137062	1.57309711	up
Atic	0.000137241	3.777183056	down
Chd4	0.000137241	3.153283358	down
Gyg	0.000137241	2.511395454	down
Atp6v0b	0.000137245	1.995602369	up
LOC100046483	0.000137245	1.714792252	up
scl0002368.1_75	0.000137245	3.614784956	down
6030441I21Rik	0.000137664	2.237386704	up
Gdf10	0.000137664	2.198053122	down
2010209O12Rik	0.000137699	1.510862589	down
Fbxw11	0.0001377	2.155486345	up
Haghl	0.00013786	1.854074359	up
1190002H23Rik	0.000137999	2.61289382	down
Mgp	0.000137999	1.884006143	down
Tubgcp4	0.000137999	1.804013491	down
eGFP	0.000138096	2.251048327	down
LOC100039786	0.000138096	3.559148073	down
Casp1	0.000138109	3.609135389	up
LOC384017	0.000138109	1.737968445	up
Nsun5	0.000138109	2.054802179	down
Rwdd1	0.000138109	2.392724514	down
Ubac1	0.000138109	1.838465571	up
Bmf	0.000138166	2.116255999	up
Ctnnd1	0.000138565	1.712701678	up

Cdc42ep3	0.000139151	2.477018833	down
Gmcl1	0.000139791	2.46929431	down
B230327D02Rik	0.00013985	2.446418285	up
Bcas2	0.000140269	1.929402709	up
LOC100041500	0.0001403	1.77828598	down
5830408B19Rik	0.000140538	2.067686796	up
Mrpl9	0.000140538	2.022320986	down
Ppt1	0.000140538	2.951411963	down
Tsr2	0.000140538	2.192851782	down
Rer1	0.000140932	3.518359423	down
A930001K02Rik	0.00014106	1.60479939	up
Slc11a2	0.00014106	1.905745029	up
Amigo2	0.000141061	1.647422075	up
LOC386534	0.00014129	1.779201269	down
Cacna1g	0.000141463	1.854526758	up
Tssc1	0.000141487	2.46271801	down
LOC675228	0.000141645	1.539102912	up
Rasgef1b	0.000141645	1.734761953	down
Rock1	0.000141645	3.036412716	down
4930579G24Rik	0.000141816	2.567597628	down
LOC224914	0.000142599	3.398641348	down
Mib1	0.000142599	1.643849969	down
Mkl1	0.000142599	3.165697575	up
Dtx3l	0.00014272	1.838681459	up
Rassf1	0.0001428	3.162237644	down
Col2a1	0.00014311	4.782061577	down
Galnt1	0.000143429	2.995471954	down
Grrp1	0.000143429	1.624820709	up
Ssh1	0.000143528	1.837801218	up
Krr1	0.000143639	3.11702013	down
9830001H06Rik	0.000143649	2.590276957	up
Nsccn1	0.000144019	1.940010428	up
Pde1a	0.000144025	1.505442143	up
B230215E14Rik	0.000144219	1.732112527	down
Jarid1b	0.000144414	1.920661807	up
Tyrobp	0.000144967	8.94783783	up
Uros	0.000145046	3.017705441	down
Ibtk	0.000145204	1.854676843	down
Jund1	0.000145623	1.611979365	up
LOC382145	0.000145623	1.808943629	up
1110019O10Rik	0.000145743	2.559967995	up
D12Ertd551e	0.000146949	1.897744536	down
Gnai2	0.000146988	1.68414557	up
LOC100044363	0.000147572	2.188920975	down
Mbtd1	0.000147572	2.307616949	up
Surf2	0.000147572	1.810287476	down
Rbm8a	0.000147651	1.960102916	up
Lrrc59	0.000147744	2.17415309	up
2610036L11Rik	0.000148486	1.846237898	down
Csnk1d	0.000149287	7.794163704	down
Prmt5	0.000149678	4.670490742	down
Clk2	0.000149894	2.464841843	down

Clta	0.000149894	2.785630941	up
Matn1	0.000149894	21.81431389	down
Pik3c2a	0.000149894	2.614270687	down
Rad23a	0.000149894	2.005789518	up
Ddah1	0.000150202	2.672152281	down
Top2b	0.000150433	2.274976969	up
2310061C15Rik	0.000150549	1.859614253	up
6330548G22Rik	0.000150549	2.512672901	down
D930017K21Rik	0.000150549	2.548394442	down
Fam125a	0.000150549	1.557834983	up
Entpd6	0.000150742	1.803019047	down
H2-Ke6	0.000150919	1.691312194	up
Nup54	0.000151071	2.283360004	down
Mrrf	0.000151654	3.472204208	down
LOC386537	0.000152253	2.130187035	down
Tmco3	0.000152503	2.340826511	down
Grin2a	0.000152565	1.686123848	up
Laptm5	0.000152566	14.41507435	up
Fbxo8	0.000153197	2.77720499	down
Adrm1	0.000153259	1.780843496	down
Gpx8	0.000153259	2.936581612	down
Pelp1	0.000153259	1.933809876	up
Slc30a5	0.000153381	2.769304037	down
H2-T23	0.000153863	1.792632103	up
Pwwp2b	0.00015419	1.613749385	up
Rbbp9	0.000154216	2.113550901	down
Snurf	0.000154585	5.384869576	down
C030015D21Rik	0.000154974	2.022259474	up
Gde1	0.00015503	2.987529039	down
Prkg2	0.000155469	2.403168201	down
2810442O16Rik	0.000155825	2.647425175	up
Robo4	0.000155974	3.936722517	up
C530040B18Rik	0.00015612	2.233397722	up
Ddx10	0.000156189	3.112540722	down
Kpna4	0.000156189	1.685567498	up
Ergic2	0.000156554	2.529469729	down
scl0002443.1_418	0.000157052	1.671161413	down
Tubg1	0.000157104	2.520186901	up
A930025D01Rik	0.000157548	1.654757142	up
Nup50	0.000157548	2.112403631	down
AW120700	0.000157574	1.721542716	up
Cdca4	0.000157574	3.118937254	down
Mib2	0.000157574	1.802290916	up
Nckap1l	0.000157574	6.819299221	up
Pcolce2	0.000157574	3.70184803	down
Ptprs	0.000157574	1.818262696	up
Rpain	0.000157574	1.658653379	up
Sash3	0.000157574	5.23313427	up
Strn3	0.000157574	2.576465607	up
Plvap	0.000157636	2.057566881	up
LOC380706	0.000158041	1.573249698	up
2810407C02Rik	0.000159031	2.07134819	down

Srpx	0.000159031	2.234292746	down
Cox4nb	0.000159147	1.87360847	up
Ppp4r4	0.000159147	1.729207873	down
2410015M20Rik	0.000159297	1.890218139	up
Exod1	0.000159297	3.380065441	down
Snhg11	0.000159297	2.141139031	up
Ilf3	0.000159389	1.76270628	down
6330575B07Rik	0.000159506	2.070081949	up
Aqp1	0.000159506	1.709627271	down
scl0002791.1_134	0.000159506	4.224085331	down
Nfya	0.000159534	2.474164963	down
Nte	0.000159715	2.236660004	up
C920016K16Rik	0.000159936	1.780875325	up
LOC213480	0.000159936	6.108706951	up
Ikbkb	0.000160557	2.112020493	up
Hnrpm	0.000160579	3.236380577	down
9930105H17Rik	0.000160743	2.209055662	up
Fpgs	0.000160783	2.574602366	down
Chad	0.000160975	3.34805131	down
C130067A03Rik	0.000161369	1.89114964	up
Pomt1	0.000161649	2.218795538	down
Crls1	0.000161767	1.603158593	down
Alg14	0.000161813	1.762085676	down
Cd320	0.000161848	1.627228022	up
Csda	0.000161948	2.968462229	down
5730526G10Rik	0.000162241	2.624038935	up
Actr10	0.000162241	3.908699751	down
Stxbp2	0.000162241	2.303475142	up
Bcl2a1b	0.000162888	3.849477053	up
Sfxn2	0.000162888	1.936788321	down
Ccl4	0.000163236	5.268384934	up
2310014D11Rik	0.000163965	1.908103347	up
Ift20	0.000163965	2.595682383	down
EG668831	0.000164093	2.309412956	down
Phldb2	0.000164297	2.147843361	up
Smarcd2	0.000164297	2.058829784	up
Mcm6	0.000164502	2.807902575	down
Mll1	0.000166815	2.228741407	up
Slc25a11	0.000166815	1.81892693	down
Prpf4	0.000166852	1.911750555	down
4732418C07Rik	0.000167002	1.560702086	down
Cdk6	0.000167002	1.7740798	down
4930418I18Rik	0.000167869	1.956115007	up
Adora2b	0.000167869	1.651929617	down
Spred1	0.000167979	2.010764599	down
Ak2	0.000168415	2.802975655	down
LOC100040919	0.000168566	1.761731863	down
LOC100044159	0.000168566	1.764511824	up
Osbpl11	0.00016901	1.661468267	up
2610207I05Rik	0.000169109	1.543571949	up
Ect2	0.000169814	2.233487606	down
EG237361	0.000169839	1.583426714	down

Acbd5	0.000169853	1.699282169	down
Zfp687	0.000169853	2.247606039	up
Lamp2	0.000170013	2.00801754	down
Vars2l	0.000170013	1.995289683	up
Csnk2a1-rs4	0.000170201	2.534597397	up
Aktip	0.000170243	1.671546698	up
Nup85	0.000170363	1.640829682	up
Entpd6	0.000170728	1.828014612	down
4833439L19Rik	0.000171146	2.512944937	down
LOC384593	0.000171146	2.057909966	up
5830467E07Rik	0.000171257	1.807436705	up
scl0003237.1_21	0.000171297	1.786426902	down
7420700D11Rik	0.000171324	2.728879929	down
Ccdc97	0.000171358	1.901199222	up
Ube4b	0.000171358	1.58241725	up
Creb3l4	0.000172455	1.835996747	up
Pdzd8	0.000173315	2.089199781	down
mt-Nd6	0.000173548	3.425084829	up
scl000034.1_162_REVCOMP	0.000173548	1.862678766	up
Tubb2b	0.000173548	9.238461494	down
Dph3	0.000173756	1.626571298	down
Nfatc1	0.000173756	3.104269505	down
Slc35e3	0.000173756	2.735560656	down
Cyp4v3	0.00017573	1.880655766	up
Ppil3	0.00017573	3.462114334	down
LOC225924	0.000176484	1.94114542	down
Zhx1	0.000176596	1.641436458	down
C130078D09Rik	0.000176664	1.997104049	up
Plxnb2	0.000176791	2.296969891	up
Nme7	0.000176904	4.145028114	down
Hoxb4	0.000177079	3.129840374	up
1700071K01Rik	0.000177333	1.771579504	up
Cttn	0.000177333	2.442151308	down
Imp4	0.000177333	2.244060278	down
2510042H12Rik	0.000177455	5.464388847	up
Rbms1	0.000177455	1.956834078	down
A430107O13Rik	0.000177985	1.644015312	down
Esam	0.000178354	3.492754221	up
Mak16	0.000178354	2.69032526	down
LOC381132	0.000178773	1.781636596	up
Setd1a	0.000178989	2.38780117	up
Lyplal1	0.000179372	2.084866047	down
Swap70	0.000179372	1.938699961	down
Snx7	0.00018032	2.196594715	down
LOC100046918	0.000180627	6.357720852	down
Minpp1	0.000180671	2.846674681	down
2810449M09Rik	0.000180728	2.424717188	up
Snrpd3	0.000180799	2.356797218	down
Spryd4	0.000180799	2.254445076	down
Hel308	0.00018097	1.657880664	down
LOC669660	0.000181279	2.312144995	down
Gm1673	0.000181618	1.606569052	up

Pcca	0.000181618	2.243657351	down
Lmcd1	0.000181909	2.61991334	down
Txndc11	0.000182272	1.925432921	down
Mrpl18	0.000182329	2.529149294	down
Pmp22	0.000182338	2.244052887	down
4933421O10Rik	0.000182614	1.599602699	down
A630053N20Rik	0.000182614	1.542620421	down
Actn4	0.000182614	1.867175221	down
Ece1	0.000182614	2.585051537	up
Sgsh	0.000182614	2.196357012	up
Tor1aip1	0.000182614	2.358776569	down
Uhrf2	0.000182614	3.120145798	down
Ebag9	0.000182954	1.851425886	down
Pde7a	0.000183294	2.622594118	down
Slc39a1	0.000183908	1.91736424	up
LOC100044324	0.00018419	1.998746514	down
LOC676974	0.000184285	4.909405708	down
2610208M17Rik	0.000184606	1.524551868	down
2610305D13Rik	0.000184869	2.489267111	up
Tulp3	0.000185298	1.678471685	up
B230209J16Rik	0.000185385	2.346597672	down
Bms1	0.000185385	2.213126659	up
D430029G22Rik	0.000185385	1.726414561	up
Lrpprc	0.000185385	2.193031788	down
Pawr	0.000185385	1.703588367	down
Tbc1d25	0.000185385	1.577071071	down
Col9a1	0.000185424	4.804460526	down
Ebf3	0.000185925	2.323424101	down
C730029F17Rik	0.000186285	1.750896812	down
Angptl6	0.000186962	1.557759881	up
Cltc	0.000186962	3.606269836	down
Shc1	0.000186962	2.020826817	down
A630020I15Rik	0.000187293	1.850595832	up
Wdr82	0.000187929	2.318786383	down
Anxa4	0.000188032	2.144967794	up
C630023I10Rik	0.000188347	1.626683354	down
LOC625752	0.000188347	2.01060915	up
A430041B07Rik	0.000188614	1.926435471	down
Kif15	0.000188614	2.141533852	up
LOC100048316	0.000188641	3.269137621	down
2810022L02Rik	0.000188716	1.928429723	down
scl0001764.1_391	0.000189054	1.957418442	up
Creld1	0.000189229	1.6855793	up
Ndufs4	0.000189502	1.725573778	down
Wif1	0.000189969	2.992610455	down
Acsl3	0.000190071	1.660969496	up
LOC100043011	0.000190071	2.338733673	down
Glis2	0.000190262	1.845925927	down
Stra13	0.000190361	1.632276774	down
St5	0.000190435	1.758947134	up
Pmm2	0.000190475	3.062105179	down
LOC674214	0.000190546	1.774911046	up

Snx13	0.000190969	1.833219647	down
A830081L15Rik	0.000191081	3.914179564	up
Rasl11b	0.000191642	4.432396889	down
Bccip	0.000192184	2.060667515	up
2610318N02Rik	0.000192478	2.004095316	down
Coq6	0.000192779	2.683963537	down
Ptgs1	0.000192952	2.401218891	up
Xist	0.000192952	2.453337431	up
2010106G01Rik	0.000192957	2.00118947	down
Txnrd1	0.000193271	2.221156359	up
1700034H14Rik	0.00019368	5.542374134	down
5930404L04Rik	0.00019368	1.618393064	up
Papss1	0.00019368	2.286304712	down
Pdzd11	0.00019368	2.356324673	down
4833426J09Rik	0.000194382	1.871853352	up
Elfn1	0.000194382	1.511797667	up
Fn1	0.000194382	4.103619576	down
LOC100044774	0.000194382	1.791710377	up
LOC219145	0.000194382	5.261116028	down
LOC385644	0.000194382	3.93204689	down
Praf2	0.000194382	2.07302165	down
Tgfbr2	0.000194418	2.211287975	down
Kank4	0.000194497	3.309415579	down
LOC621612	0.000195068	16.27458191	down
D6Wsu163e	0.000195416	1.843824387	down
9030025P20Rik	0.000195492	1.611269712	down
Gsn	0.000195492	3.569870949	down
scl0002500.1_87	0.000195492	1.718197107	down
Vamp8	0.000195685	1.792883992	up
Kifc2	0.000196762	2.367343187	up
E330018D03Rik	0.000197053	2.762667418	up
Fam120b	0.000197096	2.083258629	down
5730409K12Rik	0.000198304	1.716290116	up
Trim68	0.000198596	1.823496938	up
Nsdhl	0.000198772	3.438551664	down
Bxdc1	0.000199054	1.746124148	up
D930028F11Rik	0.000199054	1.836795688	down
LOC100043675	0.000199179	4.093404293	down
Arid4a	0.000199289	2.117062807	down
Twsg1	0.000199575	1.890249968	down
Ttk	0.000200007	1.543180704	down
Pcsk6	0.000200021	2.70586133	down
Man1a	0.000200224	2.250897408	down
Rfc2	0.000200268	3.2469244	down
LOC635297	0.000200399	1.767178178	down
Vkorc1	0.000200648	1.718533993	down
Shox2	0.000200839	2.458697319	up
2310066E14Rik	0.000201798	2.23844552	up
Wbscr27	0.000202149	1.81077075	up
Mccc1	0.000202952	1.944127798	up
zfp507	0.000203364	1.507071495	up
Cks1b	0.000203401	2.060446024	up

LOC385849	0.000203401	1.717190385	up
Rnf34	0.000203401	2.586604357	down
Deb1	0.00020349	1.929145098	down
Camk2a	0.000204044	1.617012858	up
Spg7	0.0002041	1.760671735	up
Ccdc109a	0.000204257	4.056745529	down
Sema3e	0.000204257	1.784626603	down
Insl3	0.000204376	2.253713846	up
Trrp2	0.000204376	1.530748129	up
Afg3l2	0.000204395	1.6511513	down
Dhx16	0.000204395	1.649022222	up
Sav1	0.000204395	1.698193073	down
BC057552	0.0002049	2.077195883	up
Eif2b1	0.0002049	2.284325838	down
Rwdd3	0.000205164	1.748553515	down
Lbr	0.000205577	3.998609781	down
Klhl6	0.000205834	4.3796525	up
LOC100047260	0.000205935	2.021965265	up
LOC383860	0.000205935	2.06079936	down
Stfa1	0.000205935	1.990672827	up
Hs3st6	0.000206971	1.50545454	down
LOC630567	0.00020928	1.660908938	up
scl0002083.1_6	0.000209287	2.339989662	down
BC031853	0.000209613	2.614721775	up
Nat12	0.000209928	2.433582544	down
Tceal1	0.000210063	2.470224142	down
Tmem201	0.000210115	1.929938436	up
LOC380771	0.000210973	1.569788575	up
Nit1	0.00021119	1.603829622	up
Mmrn2	0.000211225	2.364754677	up
Zfp512	0.000211225	1.506615281	down
Vwf	0.000211269	3.012349367	up
Fbn1	0.000211383	1.988004923	up
Cx3cr1	0.000211409	9.247459412	up
BC017612	0.000211431	3.302004099	down
LOC100044736	0.000212035	2.109254122	up
1700123A16Rik	0.000212288	3.86538291	down
Coq10a	0.000212379	1.652246952	up
Lyve1	0.000212481	5.489628315	up
Zc3h13	0.000212622	2.570641756	down
Nt5c	0.000212699	2.678080082	up
Nkiras2	0.000212928	2.277071714	up
Rarb	0.000213139	2.624078751	down
Tbp	0.000213929	2.356360912	down
BC003993	0.000214197	2.68414855	down
BC016423	0.000214197	3.991883993	down
Nus1	0.000214197	3.011378527	down
Il6st	0.000215054	1.585917234	up
Maob	0.000215054	2.211835861	down
Elac2	0.000215058	1.903346419	down
Sdccag8	0.000215058	1.983003497	up
Tbc1d2b	0.000215094	1.949914932	up

5031439A09Rik	0.00021512	1.599751711	down
Fcgr3	0.00021512	9.903422356	up
Lrrc33	0.00021512	3.905851841	up
Pogk	0.00021512	1.748232365	down
Saps1	0.00021512	2.94052577	up
Cxcl4	0.000215307	16.17577362	up
Ccdc111	0.000215316	1.945296645	up
Prdx3	0.000215316	2.87779212	down
Hes6	0.00021544	3.088968277	down
Anxa5	0.000216236	2.012839794	down
Foxa3	0.000216565	2.651058435	down
Plek	0.00021745	2.357760429	up
Fcna	0.000217756	4.94371748	up
LOC100045439	0.000217756	3.446047545	down
Cirh1a	0.00021776	2.133444786	up
LOC626309	0.00021776	6.07749176	down
Dlx6	0.000217916	2.094132662	down
Freq	0.000217916	1.907877088	up
Glcci1	0.000217916	2.098738909	down
Ngfrap1	0.000217916	1.671906591	down
Nol5a	0.000217916	2.633870363	down
Pcgf5	0.000217916	1.571983933	down
1500041N16Rik	0.000218599	1.89926815	up
Ltbp3	0.000218781	2.40032959	down
1300013D05Rik	0.00021886	1.626240134	up
Mthfd2l	0.00021886	1.65741992	down
Endogl1	0.000219798	3.566064835	down
EG268795	0.000219822	2.886470556	down
Mrps26	0.000219822	2.316550255	down
Timm9	0.000219822	1.986933231	down
Akap1	0.000219944	2.098371983	down
Stam	0.000221333	2.468398094	down
Arhgef2	0.000223004	1.610099435	down
Cd84	0.000223829	1.749578714	up
9630013P03Rik	0.000223866	3.472429276	up
Cacna1g	0.000224239	2.350675106	up
St6galnac4	0.000224306	1.634081125	down
Wapal	0.000224306	2.071667671	down
C230091E03Rik	0.000224348	3.405141354	down
Decr2	0.000225822	2.220481634	down
Tk1	0.000225973	2.891421318	down
2810408P10Rik	0.000226344	1.749534845	down
4933424B01Rik	0.000226876	4.310250759	down
Coil	0.000228256	2.063959837	up
Murr2	0.000228654	1.572029591	down
Sfrs2	0.000228654	2.096890688	down
2310005L22Rik	0.000229152	1.930631399	down
Cbfa2t2	0.000229604	1.83970499	up
LOC100040961	0.000230951	2.51737237	down
Nkx6-1	0.000232384	2.532574654	up
LOC638050	0.000232694	2.10479641	up
LOC628836	0.000232986	2.211776257	down

Tle2	0.000232986	2.70662117	up
Sae1	0.000233036	4.142287254	down
Rnaseh2a	0.000234629	2.288468123	up
R3hdm2	0.000235634	2.053689003	up
LOC100043181	0.000235882	1.797769785	up
EG432534	0.000236105	1.695041537	up
4930426D05Rik	0.00023633	2.455380201	up
A430093A21Rik	0.00023633	1.889054894	up
Foxred1	0.00023633	2.407088518	down
Rnf182	0.00023633	1.947802186	down
Siah1b	0.00023633	1.509672284	down
Wnk1	0.00023633	1.862841845	down
Rbm14	0.000237297	2.344337225	up
scl0001757.1_1	0.000237422	3.968798876	down
Zfp317	0.000237422	1.781011343	down
C1qbp	0.000237723	2.832381725	down
Ddx49	0.000237723	2.167202234	up
Kcnn3	0.000238409	26.12154007	down
Ctsh	0.000238578	2.588484526	up
Golga7	0.000238578	2.295824051	up
4921511K06Rik	0.000239266	2.137606382	up
Fam107a	0.000239267	2.543306351	down
mtDNA_ND6	0.000239267	1.663390875	up
Snw1	0.000239267	1.627378225	up
Mcoln3	0.000239617	1.574297309	down
Trmt2a	0.000239924	2.943390369	down
A630097D09Rik	0.000240469	2.245725632	up
5330403J18Rik	0.000240485	1.561487794	up
D930002L09Rik	0.000240485	2.907100201	up
Dnase1l1	0.000240485	1.636565328	up
EG667723	0.000240545	2.104596376	up
Orc4l	0.000240964	1.978468895	up
Arfrp1	0.000241076	1.721571922	down
Fgd4	0.000241076	1.711675644	down
Clspn	0.000241124	1.767380476	down
Iigp2	0.000241124	1.965031624	up
Fam101a	0.000241182	2.988392353	down
LOC100048613	0.000241182	1.636793494	down
Zfp629	0.000241182	1.707373023	up
Ggnbp1	0.000241256	1.772250533	up
C730040L01Rik	0.000241516	2.060833216	up
Kirrel3	0.000241724	1.939929843	up
scl000596.1_38	0.000242036	2.042620659	down
Ddx23	0.000242341	1.651549459	up
LOC100038993	0.000242341	3.092079401	down
Rnmt	0.000242341	2.56573391	down
0910001A06Rik	0.000242445	2.066383362	down
Agpat3	0.000242445	1.554988265	up
Cobll1	0.000242445	1.6672225	down
Iars	0.000242445	2.350120306	down
Kdelr1	0.000243251	2.151457787	up
Caprin1	0.000243279	3.442944765	down

Ikbkap	0.000243279	1.791815281	down
Ddx47	0.000243584	2.545067549	down
D130059P03Rik	0.00024379	2.712814331	up
Gnl3	0.00024379	2.154230833	down
Mrpl48	0.00024379	1.950306773	down
Hpgd	0.000245896	2.150220156	up
Setx	0.000245971	1.906172633	up
Hmgcl	0.000246484	2.804630518	down
Pdlim2	0.000246832	2.173926115	down
A630064D23Rik	0.000247049	1.93936336	down
A230080D12Rik	0.00024725	1.507227182	down
Sh3kbp1	0.000247311	2.331632614	down
Sri	0.000247342	1.731450319	down
LOC384727	0.000247951	1.950770259	up
Ssbp3	0.000247951	2.159426451	up
Sufu	0.000248312	2.23089838	up
Nupl1	0.000249427	1.626747847	down
1500003E24Rik	0.000251124	1.578021526	up
Slc24a2	0.000251124	1.623716593	up
Ankrd6	0.000252291	1.650712609	down
Sec22a	0.0002528	4.064647198	down
Eps8	0.000252871	2.723695993	down
Gm1040	0.000252871	2.341539383	down
Jak2	0.000252871	2.157834053	down
Jazf1	0.000252871	2.499022245	down
LOC100041219	0.000252871	4.021774769	down
Mpv17	0.000252871	2.06839323	down
Hist1h2be	0.000253722	1.672124267	down
Acsl4	0.000254031	1.903488159	up
LOC385985	0.000254031	1.831303954	up
Rbm45	0.000254189	1.55312562	down
D11Wsu47e	0.000254211	1.936860204	up
Ly86	0.000254331	4.478281021	up
Zfp422-rs1	0.000255321	3.085183382	down
Ubxn11	0.000257367	1.5784688	down
Lum	0.000257459	2.972158909	down
Gak	0.000257655	1.94924736	up
BC039210	0.000258203	3.458497286	up
Eml4	0.000258222	1.754005194	up
Fxr2	0.000258222	2.355408669	up
Ube2k	0.000258488	1.606120706	down
St3gal1	0.000259043	1.560395718	down
Hook2	0.000259396	1.91838932	up
Taf5	0.000259582	2.115554094	down
Zfp644	0.000260679	3.020000935	down
Txnl4b	0.000260862	1.848664045	up
Uck2	0.000260862	2.313199997	down
Taf5	0.000260949	2.224843502	down
Bex1	0.000261203	1.94656229	up
Smarca5	0.000261793	3.157713652	up
Sox6	0.000262698	3.285536766	down
Clec4a1	0.000263176	2.431267738	up

C530044N13Rik	0.000263619	1.66377902	up
Pom121	0.000263619	2.041603327	up
Zcchc5	0.000264633	2.593349457	down
5730492I20Rik	0.000265424	1.92709291	up
Fcgrt	0.000265619	3.487201929	up
scl0001093.1_24	0.000266338	2.282047033	up
Crlf3	0.000266856	3.042374849	down
Bcl11a	0.000267144	1.595377803	up
Gpam	0.00026768	1.720186472	down
Col9a2	0.000268026	3.261986256	down
LOC633945	0.000268913	2.131943941	up
5430437P03Rik	0.000269799	2.609329939	up
Fyb	0.000270637	2.301314592	up
Mtfmt	0.000272081	1.839958429	down
Snx9	0.000272313	1.786275148	down
2700038C09Rik	0.000272401	1.782839537	down
Bat3	0.000272788	1.565569878	up
Ppcdc	0.000272913	1.588259459	up
Cat	0.000273567	1.666490912	up
Ddx42	0.000273856	2.100128412	up
Tmem111	0.000273856	2.115414381	down
6720401G13Rik	0.00027524	1.954547167	up
Ifnar2	0.00027524	1.985054493	down
LOC384646	0.000275743	1.69972074	down
Usp9x	0.000275795	1.864694238	down
Cd200	0.000275905	2.098821878	down
D930038M13Rik	0.00027737	2.106680632	up
Ddx49	0.000277921	2.63120532	up
Pctk1	0.000277921	2.52860713	up
LOC626785	0.000278169	3.180973053	down
Ddx52	0.000278189	2.0785079	down
Jph1	0.000278189	1.649755478	down
Sh3pxd2b	0.000278246	1.652979255	up
A430099B18Rik	0.000278365	1.796834588	down
Gnb2l1	0.000278365	1.892762184	up
Islr	0.000278365	2.281063557	down
Slc44a2	0.000278365	4.12567997	down
Asph	0.000279182	2.211745739	down
Mapre3	0.000280988	2.583012819	up
Trim33	0.0002813	3.103994131	down
Irf8	0.000282024	3.621077776	up
LOC100045252	0.000282309	1.658130646	up
4832404P21Rik	0.000282339	2.251608133	up
Zfp825	0.000282339	1.752790809	down
Snord22	0.000282763	1.71735704	up
Khdrbs1	0.000283197	2.394096375	down
Tfpi	0.000283507	2.061903	up
Barx2	0.000284484	2.952434301	down
Zfp821	0.000285377	2.140987158	down
3732409C05Rik	0.000285959	1.59789753	up
Alox12	0.000285959	3.394379854	up
Nup214	0.000285959	1.786797881	up

Runx1	0.000286338	2.467631817	down
Etnk1	0.000286549	1.924120307	down
Gpbp1	0.000286549	1.781497479	down
LOC675709	0.000286549	2.365521193	down
Spast	0.000286549	1.944532991	down
Tgif1	0.000286549	1.82399416	down
Stab1	0.000286615	9.266456604	up
Sfxn4	0.000286682	1.678519487	up
LOC232745	0.000286706	2.384426355	up
Gemin6	0.000287222	3.889459848	down
Pmm1	0.000287222	1.741164446	down
2810484G07Rik	0.000287373	5.494354725	up
Wdr92	0.000287373	1.929363251	up
2610029G23Rik	0.000288029	2.353754759	down
Col5a2	0.000288029	2.087709427	up
Cc2d1b	0.000289273	1.818840265	up
Tpp1	0.000289684	1.962062359	up
B4galt2	0.000289698	1.729569554	up
Myst4	0.000290586	1.949404359	up
Arf3	0.000290894	2.003899574	up
Mrps33	0.000291117	1.514313459	down
LOC100045040	0.000291226	1.772936225	up
LOC675985	0.000291226	2.440829754	up
Slc22a5	0.000291932	2.293816567	down
Actb	0.000292088	6.623762131	up
D430022A14Rik	0.000292088	3.880523205	up
Parp6	0.000292088	2.273993969	down
scl0001060.1_67	0.000292088	3.482131958	down
Wdr70	0.000292803	3.039543867	down
Paqr3	0.000293801	2.051210642	down
Gstt2	0.000293824	1.60252583	up
scl0001550.1_29	0.000294066	2.484349728	down
Pdia3	0.000296423	1.618042231	down
Gcnt1	0.000296742	1.885344744	down
Siat5	0.000296742	2.272691727	up
Cul4a	0.000297025	2.384379625	down
Psmd11	0.000297283	1.839261055	up
Rc3h2	0.000297739	2.95428133	up
2610019N13Rik	0.000297979	4.004569054	up
Aaas	0.000297979	2.296341658	down
LOC629364	0.000298305	2.578338385	down
1110020N13Rik	0.00029939	1.830717802	up
2410166I05Rik	0.000299859	2.43085599	down
5730589K01Rik	0.000299859	1.902543664	down
Dusp28	0.000299859	2.033252239	down
1810027O10Rik	0.000300369	1.753481031	up
Smpd1	0.000301672	2.30628109	down
2410021H03Rik	0.000301779	1.730605006	up
A730063M14Rik	0.000301903	1.602953315	up
6330505N24Rik	0.000302149	1.608774781	down
Setd3	0.000302149	1.939419746	up
Wwc2	0.000302267	1.793375611	up

Nr1h3	0.00030255	2.166537046	up
Cryz	0.000302584	3.602022171	down
Rbm38	0.000303073	2.121245146	up
Eif3e	0.000303773	1.82092607	up
Ccdc88b	0.000304919	3.740888596	up
5033405K12Rik	0.000305644	3.31189394	down
Ccl21a	0.000305644	2.713562489	up
Psmb8	0.000306174	3.476734638	up
LOC545867	0.000307295	1.934822321	up
Kng1	0.000307299	2.542358398	up
Fpgs	0.000307358	1.864728093	down
Mare	0.000307588	2.271430016	down
Ttc8	0.000307588	2.469508648	down
Zfp358	0.000307588	1.875130773	up
D330001D04Rik	0.000308575	1.837897897	up
Aktip	0.000308636	1.555321574	up
LOC384338	0.000308636	1.64816618	up
B830012L14Rik	0.000308922	2.830275536	up
LOC100045019	0.000309022	1.512602568	up
5930412G12Rik	0.000309617	1.563243985	up
Idb4	0.000309617	2.071163416	down
Clec4d	0.000310218	1.903172255	up
A230005G17Rik	0.00031238	1.538556814	up
Psen1	0.00031247	2.346565962	down
Mterfd2	0.000312763	3.043126106	down
Itpkb	0.000312936	1.975757837	up
Ing3	0.000313125	4.327756405	down
Rab1	0.000313244	1.707597256	down
Rbm47	0.000313907	1.734242201	down
LOC668047	0.000314178	1.809623718	up
Agpat5	0.000314188	1.512766242	up
Ankrd46	0.000314889	1.752170563	up
LOC381470	0.000314892	2.462993622	up
Abi3	0.000314902	3.405496597	up
Srprb	0.000314902	1.796862006	up
Erbb2	0.00031517	2.214454889	up
Hs6st2	0.00031517	2.03786087	down
1500041J02Rik	0.000315171	1.572867513	down
Req	0.000315312	1.733262181	up
Zcchc9	0.000315404	2.166278601	down
D930010H05Rik	0.00031679	2.128490925	up
Txndc11	0.000316817	1.765415788	down
Dact1	0.00031699	2.195098639	down
Pitrm1	0.000317562	2.215159655	up
Toe1	0.000317562	1.666069746	down
LOC383770	0.000317617	2.722924709	up
Tnip1	0.00031787	2.316067219	up
2810408A11Rik	0.000318744	1.566996336	up
Arl6	0.00031916	2.691647053	down
Txlna	0.000320709	1.99551332	up
Ms4a7	0.000321043	1.795760512	up
3200002M19Rik	0.000321314	2.12908268	down

D830019K05Rik	0.000321314	4.014717102	up
Fmnl2	0.000321314	5.00082922	down
Rtn4	0.000321314	3.269852638	down
Ard1a	0.00032186	2.350543022	down
Blnk	0.000321874	3.010790348	up
Oip5	0.000321874	3.34657526	down
Psma5	0.000321874	2.644881964	up
Ccna2	0.00032257	2.41788435	up
Gga2	0.00032257	3.426874638	down
Pcdh17	0.000322609	1.968291402	up
Usp33	0.000322921	1.685209632	up
LOC100043040	0.000323462	1.877233267	down
LOC100046895	0.000323913	2.854325295	down
Mrps35	0.000324081	2.100426912	down
Phtf1	0.000324164	1.711342931	up
Mcat	0.000324879	1.739663124	down
Lef1	0.000325198	1.737193108	down
Hbb-b1	0.000325283	6.985387325	up
Rnf11	0.000325283	3.21593833	down
Selm	0.000325283	1.926374793	up
Cdc2a	0.000325948	2.363029003	down
ORF19	0.000326039	1.760895491	up
Papss1	0.000326039	2.200952292	down
Sla	0.000326039	2.832822084	up
Thsd4	0.000326175	2.296028614	up
Glcci1	0.000327833	1.538626552	down
Apoc1	0.000328085	3.77295351	up
Mrps7	0.000328085	2.183539867	up
Tmem47	0.000328393	2.869865656	down
Uba2	0.000328457	2.070644379	down
2010007H12Rik	0.00032894	1.57797122	down
Wee1	0.00032894	1.98002708	up
D030006P03Rik	0.000329658	2.197467804	up
A230019I20Rik	0.000331073	1.978691101	down
Toe1	0.000333144	1.930790901	down
2900097C17Rik	0.00033342	2.592229843	down
Agxt2l2	0.00033342	1.594434738	down
Zfp560	0.000334219	1.89592588	down
Sbno1	0.000334322	2.26995492	down
Ctdsp2	0.000334716	2.14193058	up
Atxn3	0.000335383	1.591643691	down
Purg	0.000335532	2.246056318	down
9830144J08Rik	0.000335736	1.751068831	up
Tnfrsf22	0.000335736	1.571352959	down
Smarca3	0.000336221	1.585238218	down
Znrf1	0.000336738	3.206128597	up
Mocs1	0.000337378	2.173587561	down
Pak2	0.000337378	3.083403587	down
LOC385959	0.00033758	2.97002697	up
Pnpt1	0.00033758	2.704342604	up
Stard9	0.00033758	2.727962017	up
Trmt1	0.000337874	2.112076044	up

4930429O20Rik	0.000337883	1.66469729	up
Map1lc3a	0.000338667	1.730258346	down
Nipsnap3a	0.000340349	3.032202005	down
Zbtb8os	0.000340349	2.307601929	up
2210012G02Rik	0.000340647	2.541077375	down
Samhd1	0.000340859	2.077848673	up
Fndc3b	0.000340907	2.314380884	down
Lman1	0.00034213	1.891891718	down
Cd97	0.00034214	2.5883286	up
2310061F22Rik	0.000342434	2.166169643	up
Fkbp14	0.000342747	2.260330916	down
LOC100046211	0.000342747	2.017640829	up
Mobkl2a	0.000342747	1.881973028	down
Nin	0.000342747	2.095006704	down
Ube2f	0.00034304	2.98213625	down
Nola3	0.000343425	2.109806538	down
Wdr89	0.000343493	1.835692406	up
Irf5	0.000343507	2.473683596	up
LOC100043040	0.000343507	1.890997648	down
LOC100046781	0.000343507	3.458736897	up
Tcfec	0.000344668	1.795204163	up
6330416L07Rik	0.000344828	1.581730008	up
Flrt3	0.000344828	2.207974911	up
6030400A10Rik	0.000344987	1.872022748	up
LOC100040244	0.000345076	2.963864088	down
Srp72	0.000345347	1.933482289	up
Pdgfb	0.000345525	2.834603071	up
Zfp566	0.00034575	1.770658374	down
Nav1	0.00034588	1.721446514	up
Cxxc1	0.00034705	1.529542327	down
Ptges3	0.000347749	2.520469666	up
A730017D01Rik	0.00034777	2.564999819	down
Zc3h8	0.000348173	1.666804075	up
Necap1	0.000348936	1.760411143	down
LOC100048439	0.000349058	2.399495125	up
Kpna3	0.000349606	2.255650282	up
9626984_5_rc	0.000349625	1.714767218	up
Tpd52l1	0.000349686	2.67099905	down
A730063M14Rik	0.000349741	1.726114869	up
Fkbp9	0.000350338	1.577763319	down
1500011H22Rik	0.000350738	2.617086172	down
6130401L20Rik	0.000350738	1.673751831	down
Dncl2a	0.000350738	2.579734087	up
Hist1h2bh	0.000352486	1.928431273	down
Stau1	0.000352537	1.507359505	up
D230048P18Rik	0.000353629	2.699230909	up
9030619K07Rik	0.000353747	2.012353182	up
Prdm15	0.000353747	1.768582821	up
As3mt	0.000354043	2.722685814	down
Cyp20a1	0.000354055	2.824159622	down
Slc25a44	0.000354055	2.207317591	up
Nsun5	0.000354398	2.139414549	down

Arrdc2	0.000355048	1.620995402	up
LOC100048556	0.000355191	2.948627234	up
5031436O03Rik	0.000355273	1.886453629	up
Ednra	0.000355771	2.295722961	up
2310007H09Rik	0.000356269	1.761750221	down
Gmip	0.000356269	2.041738272	up
Gpx8	0.000356269	1.676222563	down
Mybl2	0.000358396	1.677635551	down
Fzd5	0.000360281	1.685383678	down
Uba1	0.000361489	3.490824461	down
LOC381891	0.000362076	3.267707586	down
Pts	0.000362076	2.517258883	down
Sfrs11	0.000362076	2.447832584	down
2810417H13Rik	0.000363187	2.476939917	down
Isoc2b	0.000364313	1.540642738	up
2210013K02Rik	0.000364466	1.75170207	up
A130065C13Rik	0.000364466	2.643453836	up
BC030307	0.000364466	1.571127296	up
Tecta	0.000365161	1.827583432	down
A430070A22Rik	0.000366068	1.879307509	up
scl0001053.1_15	0.000366824	2.442828417	down
Tmem119	0.000366824	1.790971875	up
D130059O18Rik	0.000367135	8.277261734	down
Sf1	0.000367736	2.529525757	up
Smyd2	0.000367736	2.524350405	down
Il1rap	0.0003678	1.749590874	down
Slc29a3	0.000368532	1.676889062	up
F830002E14Rik	0.000369541	3.914186478	down
LOC216036	0.000369541	1.957380533	up
LOC381151	0.000369541	1.694757104	up
Tshz2	0.000369541	3.209869623	up
9130227C08Rik	0.000369815	1.924268603	up
A630001G21Rik	0.000369815	2.302196503	up
Ezh2	0.000369815	3.118177891	down
Mier1	0.000369815	2.383575201	up
Plek	0.000369815	3.731998444	up
Ube2l6	0.000371137	1.839638829	down
Ino80c	0.00037284	1.872328758	down
Tlk2	0.00037284	2.544542074	up
Arpc1a	0.00037351	3.007030964	down
Pan3	0.000374203	2.081888914	up
Birc6	0.000374442	1.661909223	up
Plekhf2	0.000374545	2.222766399	down
Vkorc1l1	0.000374545	1.947618246	down
Aabp3-pending	0.000375498	1.877115607	down
LOC620678	0.000375737	2.31983304	down
LOC668626	0.000375827	2.048596144	up
Slc25a15	0.000375827	1.543455362	down
9530031D18Rik	0.000375947	1.535835624	up
Fam118a	0.000376637	2.430905342	down
Kif13b	0.000377855	1.614841342	up
scl0001069.1_12	0.00037857	1.65809226	down

LOC226538	0.000379141	2.060730696	up
Nde1	0.000379148	3.057065487	down
Trappc3	0.000379148	2.373871088	down
Scn2b	0.000380628	1.706112981	up
LOC100047905	0.000380835	2.097543955	down
Prune	0.000381357	1.892609477	down
E130112L23Rik	0.000381582	1.7558707	up
Tom1l1	0.000383441	1.684084654	down
A630007B06Rik	0.000383453	1.612574816	down
Bmyc	0.00038349	2.091891527	up
Nipsnap3a	0.000383683	4.254371643	down
Ctcf	0.000384234	1.702556372	up
Grasp	0.000384604	1.931490898	down
Pmm2	0.000384758	2.495292187	down
Ccdc56	0.000384759	1.600585938	up
Elmod3	0.000385016	1.647088766	down
6030408C04Rik	0.000385222	3.19098258	down
AI428936	0.000385266	1.637246728	up
Bat3	0.000385266	1.502805352	up
Sec23ip	0.000385266	2.543583155	down
Ung	0.000385266	2.298276901	down
Zmynd11	0.000385266	2.895047188	down
Opa1	0.000385522	1.844746113	down
Shank3	0.000386231	1.616460085	up
EG434907	0.000386541	1.700354934	down
Isca2	0.000386541	2.766831875	up
LOC380910	0.000386541	1.545938134	up
LOC433711	0.000386541	2.451876164	up
Plekhg4	0.000386565	1.922765732	up
Zwilch	0.000387038	2.094277859	down
Sumo1	0.000387053	5.421569824	down
Sumf2	0.000387338	2.313025951	down
Xrcc3	0.000389536	1.766910553	up
LOC100043893	0.000389762	2.395801544	up
Fbxo18	0.000389923	2.492236137	down
Prpf3	0.000389923	1.770958304	down
EG232875	0.000390632	1.967683792	down
Ube2b	0.000390632	1.630861521	up
Dlg2	0.000390814	1.835449576	up
Timd4	0.000392478	2.094157696	up
LOC384382	0.000392698	4.527099133	down
Ndst1	0.000392698	1.803198338	up
Timeless	0.000392698	1.641578794	up
Mrpl4	0.000393372	1.661090493	down
Camta1	0.000395935	2.402442694	down
scl0003500.1_0	0.000395935	1.949169159	down
Myo1b	0.000397132	1.891579628	down
LOC100048863	0.000398189	2.380311966	down
Men1	0.000399069	1.809636116	down
Fech	0.000400261	1.962437391	down
Ptk7	0.000400873	2.03581214	up
Nol3	0.000401117	1.623333931	up

A130090K04Rik	0.000401921	1.913383007	up
4121402D02Rik	0.000403279	1.681304455	up
C330018D20Rik	0.000404255	2.37067771	down
Lamp1	0.000404255	2.232527018	up
Opa1	0.000404255	1.51439774	down
Chd8	0.000404388	1.571517825	up
Cops7a	0.000406286	1.638715267	down
2900011O08Rik	0.000406411	2.581374645	down
Cct3	0.000406454	3.520785809	down
Gtf3c5	0.000406454	1.52745831	down
Ube2c	0.000406622	2.395749807	up
Tbc1d7	0.000407821	1.846446276	down
Fuk	0.000407917	1.65690136	up
Pcdh12	0.000408667	2.061779022	up
Eif3i	0.00040888	2.786333084	down
9230104K21Rik	0.000409006	1.534696221	up
LOC100041932	0.000409006	1.544524193	down
Dnm2	0.000409715	2.15235734	up
Serf1	0.000409729	1.671014071	up
Zfp212	0.000409729	1.58041954	down
Ift74	0.000409837	3.879684925	down
Camkk2	0.000410125	1.752634645	down
Psma1	0.000410134	6.278360844	down
Bdh1	0.000410773	1.856939673	down
6530401P13	0.000411094	1.693964958	up
Slc4a8	0.000411607	1.800826907	up
EG434404	0.000411908	2.221384287	up
Mov10	0.000411908	2.067082405	up
Pofut2	0.000411908	1.859198332	down
Golgb1	0.000412028	2.282741308	up
Chmp6	0.000412202	1.573580861	up
Fzd10	0.000415176	2.343730688	down
Slc4a8	0.000415919	1.677034259	up
A130028M21Rik	0.000416988	1.909258485	up
Zfp262	0.000417003	1.585245609	up
Rabl5	0.00041758	1.91449523	down
LOC100047966	0.000418305	1.591122031	up
Siah1b	0.000418305	2.295234442	down
Myo1f	0.000419546	3.830625773	up
Smoc2	0.000420354	1.738443971	down
1110038H03Rik	0.000420675	1.814168334	up
6330416L11Rik	0.000420675	1.954528451	down
Ccdc45	0.000420675	1.977718353	up
Fam152a	0.000420675	1.883909464	down
Selplg	0.000420675	3.762926102	up
Timm8b	0.000420675	2.266997099	down
Wasf2	0.000420675	2.171854973	up
Ldb2	0.000420989	2.367152691	down
3830408P04Rik	0.000422614	2.091636419	up
Snx25	0.000422689	1.836761713	down
2010003O02Rik	0.000423396	1.794813275	up
2210012G02Rik	0.000423396	1.684122562	down

Ankrd40	0.000423396	2.165974379	up
Hist1h2bn	0.000423396	1.608734846	down
scl0003105.1_6	0.000423396	1.545620799	down
Yeats4	0.000423396	3.189333439	down
Rcn2	0.000424693	1.558866978	down
Xpa	0.000425041	1.615720153	down
D5Ertd579e	0.000425123	1.683857203	up
3010031K01Rik	0.000425963	1.666360259	up
A430106H13Rik	0.000426687	1.609847784	up
Cdr2l	0.000427207	3.26509285	up
Ubxn2a	0.000427329	1.950295568	down
Akap11	0.000428687	2.361427307	down
Csnk1g1	0.000429613	1.744198799	up
Msh6	0.000430945	2.01474762	up
Cd86	0.000431002	4.239417553	up
Commd6	0.000431002	1.578427196	down
Rgs19	0.000432718	1.880624771	up
Nudc	0.000432851	2.195764303	down
Nrsn2	0.000432871	1.524978161	up
Ptpn18	0.000432871	2.851409435	up
Slc38a9	0.00043348	2.155992031	up
LOC384538	0.00043566	2.258776188	up
Slc14a1	0.00043566	2.209163189	down
Whsc1	0.000435845	1.546467185	up
Bbs12	0.000437551	1.699396372	down
Dach1	0.000437551	1.550462127	down
Sbsn	0.000437922	1.949364066	up
2700084L06Rik	0.000438698	1.525933981	up
Hoxa2	0.000438698	1.682744145	down
Tomm34	0.000439583	1.965200901	down
Evi2a	0.000440276	2.567021608	up
Gng10	0.000440944	4.194810391	down
Herpud2	0.000440944	2.4964993	down
Spag4	0.000440944	1.775084257	up
Tcf25	0.000441012	2.250073195	up
Ppp1r9b	0.000441257	2.3627038	up
Zfp191	0.000441257	2.150001049	down
Eif1	0.000441803	1.80425787	up
Cd34	0.000441876	1.728933096	up
Mdh2	0.000441876	2.134009838	up
Acadm	0.000441927	2.481151104	down
BC059177	0.000441927	1.875171661	up
Eya1	0.000443308	4.272898674	down
Npc1	0.000443308	1.648896575	up
Sra1	0.000443511	1.922546625	down
9630050P21Rik	0.000443675	1.524461627	up
Cdc25c	0.000443794	2.524695873	down
Muc1	0.000443794	2.351823568	up
4930452G13Rik	0.000444057	1.554724097	down
Trip12	0.000445935	2.378634691	up
Mint-pending	0.00044594	1.67340982	up
1110018J18Rik	0.000446379	2.577937365	down

Ppm1g	0.000446845	1.966290712	down
Mobkl3	0.000448863	3.029410124	down
Ms4a6d	0.000450948	2.168563128	up
9430090I01Rik	0.000451253	1.704425693	down
Spcs1	0.000451253	2.627771854	down
Ensa	0.000451737	1.533009887	down
Col24a1	0.00045244	1.953402638	down
Exo1	0.000452637	1.9862535	down
Acy1	0.000452779	1.847133756	up
Por	0.000452779	1.717088819	up
Lst1	0.000453598	5.938450337	up
Dhx40	0.00045389	1.63516438	up
Pk3	0.00045389	1.670704246	up
Pml	0.000454123	1.512720943	up
Clcc1	0.000455068	1.603530645	down
LOC622655	0.000455206	2.985795975	down
Kctd12b	0.000455634	2.037118673	up
Gprc5b	0.000456187	1.902659774	down
Azi1	0.000456336	2.047650099	up
Mrpl48	0.000456426	2.831741333	down
BC057627	0.000457896	1.659686327	up
Fam158a	0.000458866	2.143752337	up
LOC100045419	0.000458901	1.782004237	up
Inpp5d	0.000459037	2.931461573	up
8030447N19Rik	0.000459063	1.677915335	up
Hbb-y	0.000461594	16.54873848	up
Plat	0.000461744	1.612457633	down
Slc8a1	0.000462082	1.785566211	up
Wbp11	0.000462082	1.851758361	down
Jmjd2a	0.000462267	2.798635006	down
1200003C05Rik	0.00046232	2.758332968	down
Bcl11a	0.00046232	1.969925761	up
Fut8	0.00046232	2.135364533	down
Gm1614	0.00046232	2.174597263	up
Asph	0.000462575	2.216856718	down
Tns4	0.000464167	1.763277054	up
Bcl2a1a	0.000466046	2.184137106	up
Gabpb1	0.000466105	2.331307411	down
E030022B18Rik	0.000466169	1.974462867	up
2700022B06Rik	0.000466827	2.045573473	up
Plscr1	0.000467354	2.588473558	down
Ccdc43	0.000467961	2.236897945	up
Susd5	0.000467961	1.583449841	down
Zcchc8	0.000468027	1.764305949	up
Cox7b	0.00046855	1.761149645	up
Frmd4b	0.00046855	1.907487989	up
Zmym3	0.00046855	1.802528262	up
Pbx3	0.000468928	2.450549364	down
Ttc3	0.000469252	1.653300285	down
Usp4	0.000469378	1.959793687	up
1110004F10Rik	0.000469478	1.886386275	up
Trmt6	0.000470666	1.822036982	up

5430417J04Rik	0.000471163	3.432449818	down
Hdac11	0.000471379	1.870505929	up
Mageh1	0.000471379	3.569683075	down
Kpnb1	0.000472081	2.2443676	up
Zfp429	0.00047331	1.911138058	down
2900022P04Rik	0.000473574	2.956163406	up
7330409M10Rik	0.000473574	1.581189513	up
Ube1c	0.000475895	1.848529577	down
C130007D14	0.000476283	1.629169345	up
Macf1	0.000477317	1.973552108	up
D11Wsu47e	0.000477433	2.241953373	up
2900010J23Rik	0.000477476	1.735701084	up
Gstm2	0.000477476	2.143873453	down
Stat2	0.000477476	1.589756489	up
LOC278188	0.000477663	1.743216157	up
Ywhaz	0.000477663	2.80567503	up
Cdc123	0.000478586	2.016777039	up
Rfc3	0.000478586	1.708070159	down
Tnni3	0.000479565	1.505256653	up
BC042423	0.000479943	2.574317217	up
Lgals3bp	0.000480158	3.041846037	up
Fbxl8	0.000480856	1.588386655	up
4921537F17Rik	0.000481722	1.505080462	down
Fam178a	0.000481722	1.917191505	up
Mbtd1	0.000481722	1.966357231	up
Sox5	0.00048174	8.529214859	down
EG384770	0.0004823	1.73527801	down
1810009O10Rik	0.000482481	2.56532383	down
Gas2l1	0.000483652	1.992506266	up
Clcn2	0.000484076	2.045736313	up
Zbtb5	0.000484076	1.578484535	up
Aars2	0.000484554	1.886784554	up
AI849286	0.000484554	1.696458578	up
2410015N17Rik	0.000485144	1.686324716	up
Emd	0.000485144	3.765258789	down
Mettl4	0.000485144	1.590278506	down
Tera-pending	0.000485144	3.131609917	down
Uap1	0.000485548	1.807563782	down
D16Bwg1494e	0.000485603	1.577403069	up
Cspg4	0.000486174	1.713827133	down
Nudt9	0.00048646	1.657863975	down
Rab8b	0.00048646	1.745767474	down
2210414L08Rik	0.000488328	1.874861717	up
EG434078	0.000488693	1.517744184	down
B230399E16Rik	0.000489015	1.875124931	up
LOC236294	0.000490485	1.541718364	down
Adam32	0.00049152	1.912282109	up
Bcr	0.000493116	2.038388491	up
Col27a1	0.000493116	2.459280014	down
Pxn	0.000493116	1.854892135	up
Stk25	0.000493116	2.02084899	down
Polr3gl	0.000493643	1.515915394	down

Col4a1	0.000494259	3.014300823	up
Nosip	0.000494259	1.728900671	down
Csmd3	0.000496383	1.743965507	up
Gprc5c	0.00049804	1.778364897	up
E230024I19Rik	0.000498321	1.930791497	up
LOC381873	0.000498321	1.979355335	down
Tsc2	0.000498582	2.261884451	up
Ranbp10	0.000499091	1.830679178	up
LOC386002	0.000499099	1.59231317	up
scl000805.1_121	0.000499099	1.888085246	up
Glt25d2	0.000499923	1.897881746	down
Stk36	0.000499923	1.802004814	up
Vcan	0.000500393	5.157835484	down
Abhd11	0.0005006	2.076901913	up
Gstm4	0.000501075	1.910265923	up
Cttn	0.000501494	3.115800142	down
Tbc1d7	0.000501494	2.657574415	down
Arpc1a	0.000502014	2.072750092	down
2700032M20Rik	0.000502038	1.524716735	down
Nup98	0.000502545	1.658284545	down
D930029E11Rik	0.000502934	2.299012423	up
Cuta	0.00050357	1.595215082	up
Trem2	0.000504071	4.510470867	up
Fbxl6	0.000504182	1.515978575	up
Rps6kb1	0.000504182	2.289428473	down
Glt8d2	0.000504325	1.547719836	down
Tmem33	0.000505915	1.679704547	down
Gpnmb	0.000506459	2.243941784	up
Zmpste24	0.000508061	1.983035088	down
Slc35f5	0.000508911	1.848781109	up
Rpl35	0.000509108	1.512959957	down
Hcfc2	0.000510417	2.133659601	down
Clock	0.000510509	1.502933264	down
Vti1b	0.000510509	1.529601574	down
2410004L22Rik	0.000512562	1.53736639	up
9030420J04Rik	0.000512911	1.744208098	down
Zfp113	0.000512911	2.064691067	up
Dpm1	0.000513191	1.527532697	up
N4wbp5-pending	0.000513191	1.814655066	down
Rab28	0.000513191	2.852120638	down
Alg5	0.00051503	1.831722021	down
Antxr2	0.000516232	1.527885675	up
scl0001371.1_19	0.000516469	1.821559906	down
LOC545396	0.000516702	1.68913734	up
Rpl27a	0.00051714	2.237522602	down
Tpd52	0.00051714	1.524386525	up
A630002D19Rik	0.000517243	1.903132081	up
scl000055.1_1_REVCOMP	0.000517243	1.563292146	up
4632427E13Rik	0.000517256	2.102860928	down
Kat2b	0.000517256	1.707809448	down
Synpo	0.000517528	1.684028506	down
Ing4	0.000517807	2.079653978	up

Impa1	0.000517891	2.390791893	up
9430034F23Rik	0.000518372	1.673377633	up
A930009K04Rik	0.000518372	2.099366427	up
Oas1g	0.000518613	2.206003428	up
Sphk2	0.000518613	2.187416554	up
Agrp	0.000520047	1.536401153	up
Eef1b2	0.000520867	1.821775913	down
4732471D19Rik	0.00052111	1.845188737	down
Cnih4	0.000521441	2.200380802	up
Cct7	0.000523201	1.712223649	up
Pon3	0.000523201	1.707502484	up
B930096H04Rik	0.000523222	1.654890776	down
Zfp286	0.000524841	2.811438799	up
Vill	0.00052516	2.05437851	down
9530055J05Rik	0.000526206	1.589450836	up
1500032P08Rik	0.000526668	2.218639612	up
Clpx	0.000526668	1.604758024	down
Coasy	0.000526668	1.898323655	up
Zfc3h1	0.00052669	1.60190773	down
Dctn3	0.000527164	2.503374577	down
Aprt	0.000527486	1.606891871	up
Zfp711	0.000527675	1.90284586	up
Atp13a3	0.000529676	1.762229323	down
Unc45a	0.000529981	2.227968216	up
BC023882	0.000532165	1.591169238	up
Mtap1b	0.000532165	4.394807816	down
6330503C17Rik	0.000534569	1.68778348	down
Sf3b3	0.000535026	1.603064299	up
Map1lc3b	0.000535731	1.616287947	up
Mar-06	0.000535863	4.818675518	down
A130096D14Rik	0.000535876	2.355529785	up
1500032D16Rik	0.000537693	1.862724304	down
Lect1	0.000537693	1.574525833	down
E230027K01Rik	0.000537848	3.004428387	down
Ncapg	0.000537848	1.624418378	up
Tbc1d8	0.000537848	2.058531761	up
BC023179	0.000538041	1.710401177	down
Pcdhb21	0.000539031	1.847582459	up
Coq5	0.000539278	2.606654406	down
Pik3cg	0.000539292	3.150170565	up
Aff1	0.000539391	2.008762598	up
Atic	0.000539391	1.716966033	down
Dab2ip	0.000539391	1.777291298	down
Gm1815	0.000539391	2.21323967	up
Tor2a	0.000539391	1.609770656	down
1600002O04Rik	0.000539945	1.53888607	up
Renbp	0.000539969	2.295560837	up
Birc5	0.000540201	3.050485134	down
scl0003598.1_52	0.00054063	5.654876232	down
Slc7a3	0.000540954	1.735735536	down
Tbn-pending	0.000541055	1.63851428	up
C920004C08Rik	0.000541243	2.625163794	up

Trrap	0.000541243	2.362481117	up
6430573F11Rik	0.000541806	1.823604465	down
Rnf185	0.000543896	1.994016171	down
LOC210245	0.000545277	1.577197075	down
2410013I23Rik	0.000545615	1.717621088	down
Tbp	0.000545615	1.904569268	down
Pde6d	0.000546345	2.121124744	down
Slc10a7	0.000546345	1.822438002	down
Ankrd54	0.000546354	2.007191658	down
F11r	0.000546382	1.787222028	up
LOC241621	0.000546714	1.893705845	down
2210419D22Rik	0.000547091	1.778120875	up
Cdc14b	0.000547485	1.717513919	down
Fahd2a	0.000547485	1.745841861	down
Trim33	0.000547485	3.768191814	down
1700034H14Rik	0.000548186	2.242739677	down
scl0001650.1_34	0.00054927	1.736213446	up
Ranbp9	0.000550257	3.332662821	down
Fgfr1op2	0.000550402	1.916746616	down
Gnpat	0.000550402	1.954355121	up
Tekt2	0.000550703	2.296439171	up
3010015F07Rik	0.000553122	1.886523485	down
Serp1	0.000553251	1.781548262	up
C230071H17Rik	0.000554144	2.311736822	up
Kif1b	0.000554409	1.772361517	down
Pigc	0.000555064	1.735895395	down
Srpx	0.000555064	2.520544291	down
4930522P08Rik	0.00055707	1.789220572	up
Ipo4	0.000557698	1.585484743	up
EG666668	0.000558225	2.281493187	up
LOC100046670	0.000558225	1.762299061	up
Ndufs2	0.000558225	2.269294024	down
Slc30a4	0.000558225	1.504087567	up
Ror1	0.000558413	1.958731175	down
Stam2	0.000558413	1.636899948	down
Zbtb25	0.000558413	1.616133094	down
Cib2	0.000558458	1.576177001	up
Metapl1	0.000558458	2.237871409	up
BC057893	0.000558675	1.822098255	up
Crls1	0.000558848	2.040552378	down
Smc3	0.000559829	2.958533287	down
LOC669168	0.000560017	1.767401695	up
Peg3	0.000560329	1.969079375	down
scl0002154.1_16	0.000560732	2.049314022	down
Adal	0.000562472	1.82520473	down
Fam108b	0.000563209	2.886203051	down
Sec24c	0.000564105	2.337723732	up
Psmb7	0.000564349	3.241960049	down
Slc14a1	0.000564397	1.935239077	down
Perld1	0.000564809	1.56216228	up
Csda	0.00056525	2.485371828	down
C130015E15Rik	0.000565251	2.043146133	up

LOC100043313	0.000565251	3.332410812	down
LOC384710	0.000565781	2.542629242	up
Tmem98	0.000565831	1.540804029	down
B230339M05Rik	0.000567234	1.886345983	down
Hbb-bh1	0.000567234	8.627151489	up
Actb	0.000567758	3.328948975	up
A830007P12Rik	0.000568515	3.081991673	up
Adamts19	0.000568515	1.678140163	down
Clec2e	0.000568515	1.533934116	up
Rai12	0.000568515	1.985285997	down
Slfn2	0.000568515	2.069435835	up
Tmed9	0.000568515	3.611918688	down
Hdac4	0.000569221	1.829596639	up
Atg10	0.000569692	2.110445023	down
Hoxb3	0.000569692	2.809591055	up
Pex6	0.000569692	1.702731133	down
Lama5	0.000569816	1.521775484	up
LOC383916	0.00057024	1.8674016	down
Asph	0.00057034	2.434479952	down
C630012L17Rik	0.00057034	1.680696011	up
Ccrl1	0.00057034	1.720517874	down
Rpl29	0.00057034	2.101823807	down
Zscan22	0.00057064	1.666167259	down
Ramp3	0.000570835	1.764449358	down
Xpc	0.000570835	2.508820295	up
LOC100045890	0.000571026	2.286283016	down
Best1	0.000572491	1.885774732	up
Gfpt2	0.000573797	2.037197352	down
Ttc8	0.000573839	2.16131115	down
LOC676672	0.000573908	2.361418009	down
Centd3	0.000577352	2.486511469	up
Asph	0.000579218	1.967881799	down
Zfp703	0.000579218	1.959989905	up
Cant1	0.000579598	1.63429606	down
Nthl1	0.000580107	1.620528221	up
Apoe	0.000580302	4.812194824	up
Tcfe3	0.000580302	2.148245335	up
Trappc3	0.000580302	3.298223019	down
Rnf34	0.000580572	1.995469213	down
Lonp2	0.00058146	1.659163117	up
LOC386330	0.000582638	2.845326424	up
Tspan2	0.000582638	2.072137594	down
Rbm16	0.000583236	2.05564642	up
3110079O15Rik	0.000584793	4.763119698	down
Gmfg	0.000584793	3.829207659	up
Sin3a	0.000584793	1.799347878	up
A330075M08Rik	0.000584955	1.512390614	up
LOC218617	0.000585159	3.31820631	up
Dnhd1	0.000585403	1.560232639	up
Rbm18	0.000585403	2.138007641	down
Cdc42ep3	0.00058608	1.739342928	down
Sdccag1	0.000586433	1.543614745	down

Ubxd7	0.000586693	2.770376444	down
Srrd	0.000587074	1.603800893	up
Mex3a	0.000588057	1.618872047	down
LOC385461	0.00058834	1.75891304	down
Mgl2	0.00058916	2.140232325	up
4833420G17Rik	0.000589681	1.845589399	up
Arhgap9	0.000589936	2.086451769	up
Chmp1a	0.000589936	1.654444218	up
Dph2	0.000589936	1.806661606	down
Dnajc12	0.000591924	1.854585886	down
Ankrd13a	0.00059268	2.379570961	up
Brca1	0.000593465	1.582286358	up
D130020G16Rik	0.000593465	2.072193861	up
Gpld1	0.000594402	1.722920895	up
C230021P08Rik	0.000598792	1.664154768	up
5730427M17Rik	0.000598802	2.054805994	up
Swap70	0.000599406	1.588020086	down
Fam114a2	0.000599661	1.651664257	up
Fam152a	0.000599661	1.817336321	down
Rabggtb	0.000599775	1.605100751	down
Atp6v0e	0.000601465	1.697702408	down
Ctsc	0.000601465	1.590392351	up
Lyz2	0.000602121	3.05488348	up
Brd8	0.000602371	1.986169934	down
LOC100043126	0.000603692	1.935382366	down
Lats2	0.000604515	1.704494596	down
EG625917	0.000604595	1.551429033	down
Csnk1a1	0.000605084	1.780482173	up
Txndc10	0.000605753	1.9602952	down
Snf8	0.000606858	2.43382144	up
Rad21	0.000607144	3.052485943	up
Ccdc102a	0.000607423	1.641477823	up
5930405F01Rik	0.000608812	2.653104544	up
6820427D17Rik	0.000608812	2.838063955	up
Tln1	0.000610507	1.925719976	up
Abca1	0.00061226	2.23241663	up
4933425L03Rik	0.000612606	1.563069344	up
Ern1	0.000612606	2.340700626	up
Rad51	0.000614208	2.365881681	down
B230361I03Rik	0.000614938	2.100525856	up
D030035F05Rik	0.000614938	2.997986794	down
Angptl1	0.000615072	2.244338512	down
Nol14	0.000615072	1.579216361	up
Hs3st3a1	0.000615345	1.850928664	down
Scube1	0.000615345	2.461768389	up
Il15	0.000615866	2.160155296	up
0610011F06Rik	0.000616083	1.837564826	down
Dhx15	0.000616083	1.696081638	up
Gca	0.000616083	1.895457029	up
LOC100040573	0.000616235	1.635168791	down
Ap1s2	0.000616439	2.67610836	down
Folr2	0.00061666	3.017388582	up

Lgr5	0.00061666	2.284122944	down
LOC381330	0.00061666	1.626481056	up
Galnt1	0.000617264	2.181607246	down
Kctd3	0.000617264	1.942064524	up
Tspan14	0.0006185	1.533150673	down
Rffl	0.000618773	1.826322436	down
E230017J10Rik	0.000619526	1.541134834	down
scl0004166.1_8	0.000619758	2.012847185	down
LOC100041504	0.000619839	2.407474518	up
LOC100046746	0.000619839	1.517947078	down
Cacna1g	0.000620251	1.548339129	up
Tmem49	0.000621331	1.616242886	down
Accn3	0.000622218	1.816321254	up
Trnt1	0.000622491	2.487767458	down
Cth	0.000623099	1.798004508	down
2210010B09Rik	0.000623415	1.663601279	down
BC019684	0.000623415	1.557382822	up
Sox5	0.000623415	4.999372006	down
Stac2	0.000623545	1.764598489	up
3830430K15Rik	0.000623805	2.569350004	down
Abcg2	0.000623805	1.603134394	up
AI931714	0.000624735	1.926783204	up
Ccdc47	0.000624868	1.879818201	down
Zfp367	0.000624887	3.066204786	down
E430003J01Rik	0.000625443	2.550664425	up
Kctd6	0.000625443	1.924386501	down
Lemd2	0.000625443	1.799606681	up
Hexa	0.000627026	2.404296875	up
Gja4	0.000627179	2.262559414	up
Irf1	0.000627179	1.529480338	up
1700001C14Rik	0.000627567	3.176829815	up
2810408M09Rik	0.000628598	1.580203891	down
Fis1	0.000628598	2.051882505	down
LOC100046500	0.000628598	2.027537346	down
LOC668492	0.000628598	2.055921555	down
Axot	0.000630219	1.821802855	up
Ech1	0.000631515	1.582007647	up
Eef1d	0.000632213	2.400441408	down
LOC100045343	0.000632213	1.699151516	down
Tac1	0.000632213	1.699343801	down
Erlin2	0.000632276	2.231492758	down
Tmem50b	0.000634792	1.701231003	down
Tspan6	0.000635591	1.77331996	down
2410137F16Rik	0.000637119	1.593349218	up
Gng11	0.000638233	1.563430667	up
4833438J18Rik	0.000639143	1.676720262	up
Glt28d1	0.000639308	2.084573746	down
Slmap	0.000639734	2.174184561	down
Uchl5	0.000639734	3.473177195	down
Tmem33	0.000639923	4.580630302	down
Ophn1	0.000640416	2.431102037	up
4733401A01Rik	0.000640479	2.226837874	up

6332415K15Rik	0.000640479	1.630011678	up
E4f1	0.000640479	1.672245145	up
Wins2	0.000640479	1.885013223	down
Zfp521	0.000642601	2.007312298	down
Tmcc2	0.000645878	1.511455655	up
Nudt5	0.000645977	2.207942009	down
Rarb	0.000646429	2.411809683	down
Syne2	0.000646429	1.566098332	up
1110012L19Rik	0.000646612	1.992346644	down
Smc1a	0.000646612	1.786191225	down
Thap3	0.000646876	1.733524919	up
Hspa5	0.000647488	1.969208121	up
B130018F13Rik	0.0006493	2.053495407	up
Ctps	0.0006493	3.44703579	down
Golim4	0.0006493	2.615051508	down
LOC100045617	0.0006493	7.316029072	down
Actc1	0.000650134	2.146131516	up
EG627352	0.000651345	2.949555397	up
Rcan1	0.000651345	3.088190079	down
Dnajc24	0.000651594	2.561006069	down
Rbm10	0.000652691	2.561630964	down
Pigk	0.000653385	1.948712707	down
Wbscr17	0.000653472	2.079356194	up
LOC100047226	0.000653816	2.728532791	down
BC031575	0.00065393	2.428025723	up
Ptpn21	0.000654389	1.709312081	up
G430095P16Rik	0.000656061	1.753244758	up
Prkar2a	0.000656061	1.984657645	up
Rabif	0.000656061	2.122191668	down
Trappc2	0.000658751	3.292268992	down
Zfp78	0.000658751	1.636246562	down
BC038156	0.000659578	1.909920931	down
Lrp1	0.000661608	1.784999251	up
Braf	0.000663285	1.545936108	up
Zfp708	0.000663315	1.72842598	down
Mapkapk2	0.000663693	1.712990046	up
Mrpl12	0.000663693	1.74193573	up
Zfp277	0.000663693	1.587198973	down
Nup54	0.000664038	2.326942205	down
LOC100045679	0.000664809	2.104789257	up
Chek1	0.000664872	1.873754978	down
D17H6S56E-5	0.000664872	1.763670445	down
F630047D10Rik	0.000664872	1.755932212	up
Pcyt2	0.000664872	1.778467178	down
Pecr	0.000664872	1.713209152	up
scl0001055.1_43	0.000664872	1.618322372	down
Ncf2	0.000665109	7.074065685	up
Scrg1	0.00066676	1.8617239	down
Mrg1a	0.000667822	1.653525353	down
BC006779	0.000668177	1.613143325	up
1810011H11Rik	0.000668579	2.079060555	up
Rod1	0.000668589	1.775390625	up

Sec22b	0.00066919	1.934768319	down
9630045H20Rik	0.0006692	1.985624194	up
Sep-06	0.000669405	2.151334286	up
LOC100044636	0.000669405	1.563802123	up
LOC100047674	0.000669405	1.538498282	down
Ntng1	0.000669405	1.566989303	up
Hdgf	0.000669933	1.980574608	up
Jak3	0.000669933	1.966042757	up
Galns	0.000670296	1.895004988	up
Idua	0.000671331	1.696261525	down
A030007L17Rik	0.000671831	2.500057697	down
9630002P13Rik	0.000671835	1.65614593	up
Freq	0.000671835	2.150788784	up
Fahd2a	0.000673221	1.746775508	down
St3gal2	0.000675474	1.535796166	down
Cdh8	0.00067624	1.914093852	up
Tmem50a	0.00067624	2.049880028	down
Fam110a	0.00067722	2.028334618	down
Taf6	0.00067725	1.888170362	up
Ccl7	0.00067729	1.70767343	up
Ckap2	0.00067842	3.240898371	down
LOC100047827	0.000678501	2.475079536	down
Cla3	0.000678992	2.089560032	down
Clcn4-2	0.000679773	1.675450325	down
5830417I10Rik	0.000679903	1.840769291	up
Dlc1	0.000679903	4.186814785	down
Ndst2	0.000679903	1.901230216	up
BC033915	0.000681543	2.05995059	down
Gss	0.000681543	1.652808785	down
Dusp6	0.000682623	1.744878888	down
Slc40a1	0.000682623	4.81821394	up
Rtkn2	0.000683112	1.535490871	down
1500015G18Rik	0.00068348	3.577381373	down
LOC100046843	0.00068519	1.919487357	down
LOC382096	0.000686397	2.223068237	up
Rarres2	0.000687092	2.231111765	up
Trp53	0.000688149	1.753153324	up
Zfp397	0.000688149	1.70320797	down
Hs3st2	0.000688472	2.218337297	up
2610528C06Rik	0.000690042	1.537838817	down
Med24	0.000690969	1.740402103	up
Cherp	0.000691795	1.827370644	up
Ptpn23	0.000694328	2.744419813	up
1110034O24Rik	0.000695082	1.801544547	up
Sharpin	0.000696829	2.31013298	up
Mff	0.000698425	2.018848181	down
Opa1	0.000699747	1.719260216	down
Gabpa	0.000700411	1.962973237	down
Ncaph2	0.000700597	1.600748658	up
9626984_125_rc	0.000701742	2.524171829	up
Adrbk1	0.000702044	2.29949832	up
5830448L21Rik	0.000704075	1.619936824	up

8430434A19Rik	0.000704075	1.912283301	up
LOC242025	0.000705257	2.713181973	up
Lgals3	0.000705312	4.222411156	up
Stom	0.000705312	3.041344881	up
Klhdc10	0.00070567	2.631810665	down
9330133O14Rik	0.000705793	2.004089117	up
Vav1	0.000707719	3.812849522	up
B930085B11Rik	0.00070827	1.577212215	up
Ssr1	0.000708679	1.73304832	up
Tnrc6b	0.000708679	1.602386117	up
Efcab2	0.0007104	1.514025807	down
LOC100048153	0.000710657	1.982411385	down
A930008G19Rik	0.000711104	1.579950929	up
Itgb8	0.000711406	1.710262179	up
Dnajc16	0.000713669	1.691886663	up
AW551984	0.000715419	2.543210268	down
Gfod2	0.000715606	1.715263486	up
Polq	0.00071707	1.578405619	down
2410129H14Rik	0.000718316	6.096076965	down
B3gnt9	0.000718316	1.882631064	up
Gcap3	0.000718316	1.740790725	down
Terf2	0.000718316	1.540364385	up
D930007M16Rik	0.000718318	2.343592644	up
Wipi2	0.000718403	1.976464033	down
2500002A22Rik	0.000718457	2.26513195	down
Camk4	0.0007186	1.705197811	up
Srr	0.0007186	2.932835341	up
9830169E20Rik	0.000719685	1.635277271	up
Srbd1	0.000721022	1.95819664	up
C130045F17Rik	0.000723503	2.233765125	up
Npc2	0.000723503	1.726733923	up
LOC100046844	0.00072377	1.893467903	down
Ints12	0.000725179	2.545965195	down
A230050N15Rik	0.00072519	1.636012793	up
Cpt2	0.00072519	1.654943228	up
Plce1	0.000725831	1.828465343	down
2900036G11Rik	0.00072611	2.836765766	up
9530081N05Rik	0.00072611	2.689374924	down
Hs1bp3	0.000726428	2.154220581	down
Pabpc1	0.000726428	3.044068575	down
LOC100047583	0.000726746	3.196999073	down
Ankle2	0.000728044	2.289969921	down
Tmem104	0.000729463	1.914024949	up
E030028L09Rik	0.000730768	1.967088223	up
Cyhr1	0.00073125	1.674321652	down
2310047O13Rik	0.000731476	1.608363509	down
Cdh10	0.000731476	1.569518328	down
Epdr1	0.000732092	1.641634941	down
Frag1	0.000732222	1.891791701	down
Mutyh	0.000732222	1.77733016	down
Cdh5	0.000732399	2.503920078	up
Cyld	0.000732979	2.369335175	up

1110049F12Rik	0.000733457	1.650130272	down
4833441B18Rik	0.000733457	2.662082672	down
LOC100045044	0.000733457	1.982897759	up
3300001G02Rik	0.000734942	1.731248617	up
Rpap1	0.000734942	1.729886293	up
Stoml1	0.000734942	1.604362011	down
Pomgnt1	0.000735941	1.608802795	down
Nob1	0.000736164	1.675647736	down
Tmem205	0.000737292	1.671764374	up
Rag1ap1	0.000737329	1.651530147	up
Hrsp12	0.000738137	2.312088728	down
Sympk	0.000738137	2.12604928	up
9430013L17Rik	0.000739558	1.783599615	up
Hs1bp3	0.000739638	1.757426143	down
Vps4b	0.00073964	2.351050854	up
Eme2	0.000739762	2.167007446	up
Zfp182	0.000739762	1.811989904	down
E230022H04Rik	0.000740394	2.508119106	up
Msto1	0.00074052	1.781065583	down
Cpe	0.000740596	4.363157749	down
Tmem41a	0.000740657	1.512106419	down
Rhog	0.000741394	1.563458681	up
Kif1c	0.000741698	1.829589963	up
LOC381105	0.000742691	1.750582576	up
Map2k6	0.00074276	5.535485745	down
Icam1	0.000743679	2.282441378	up
Rfwd3	0.000743679	2.023409605	down
Wdr4	0.000743679	2.14149785	down
Tead2	0.000744074	1.736636758	up
D230009O13Rik	0.000745698	1.582300305	up
5730536A07Rik	0.000746194	1.508871079	up
EG384525	0.000746671	1.943549156	up
Slc30a7	0.000746709	1.592523933	down
LOC672215	0.000746715	1.943286061	up
Gtf2i	0.000748198	3.137578249	down
Heatr3	0.000751098	1.859936357	down
Tardbp	0.000751154	2.20495677	down
9030425E11Rik	0.000752192	1.83179903	up
Dok2	0.000752192	2.219880581	up
EG245190	0.000752533	2.060107231	up
Hopx	0.000752976	2.034017324	down
Spg20	0.000752976	1.9073416	down
Camk2g	0.000754634	1.535372615	up
Ccnd2	0.00075501	1.814499736	up
Plxnb1	0.00075501	1.677596808	up
Irf2	0.000755142	1.701186299	up
Srebf2	0.000755142	1.772209525	up
Zeb2	0.000755142	2.707919359	up
Zfyve1	0.000757591	1.554730058	up
Bivm	0.000758249	1.525905371	up
Efnb1	0.000761477	1.559745431	down
LOC215879	0.000761874	1.899343014	up

Ncor1	0.000762308	1.541183472	up
Msi2	0.00076231	2.370386839	down
A730098H14Rik	0.000762381	1.671996236	up
BC002199	0.000762381	1.78702569	down
Recql5	0.000764417	1.615813375	up
EG383436	0.0007649	1.640600205	up
Slc6a9	0.0007649	1.532463551	up
Mdm1	0.00076503	1.770058394	down
Pitx1	0.00076503	2.223091126	up
Stard3	0.000765827	1.97995615	up
Adal	0.000766144	1.734062195	down
Ddost	0.000768321	1.645890832	down
Hmgn3	0.000768321	2.996997595	down
Fh1	0.000768748	1.836696744	up
Taz	0.000769761	1.56415391	down
Ppp2r1a	0.000771649	1.922006249	down
Klhdc1	0.000772018	1.991118908	down
Tyms	0.000773483	2.97887826	down
Inpp1	0.00077514	1.529582381	down
Dncli2	0.000775258	1.980805397	up
S100b	0.000775716	1.687396884	down
D330006C15Rik	0.000776156	3.314432383	down
Cic	0.00077652	1.930728555	up
Glis2	0.00077652	1.50624013	down
Nfe2l1	0.00077652	1.773530483	up
Hif1an	0.000776633	2.473073006	up
Hps5	0.000777358	1.956764102	up
Dhrs4	0.000778238	2.238277435	down
Add1	0.000778502	2.061981916	down
Dhrs7	0.000778877	2.326849461	down
Ankrd40	0.000779186	2.072690725	up
1110034A24Rik	0.000780336	1.730407715	down
Mmp16	0.000780799	2.130325556	up
scl0003857.1_11	0.000780799	1.996368885	down
Ard1a	0.000781101	2.358183146	down
EG627624	0.000782946	2.25526166	down
1700029F09Rik	0.000785744	2.494041443	down
2810426N06Rik	0.000785875	1.513880968	up
Depdc5	0.000786341	1.928196073	up
EG434616	0.000786341	1.567974448	down
Ogfod1	0.000786341	1.723189354	up
Rgs10	0.000787815	2.4998281	up
Minpp1	0.000787903	1.528426886	down
Atrn	0.000789049	1.515519381	down
Rps6ka1	0.000789049	1.815821052	up
Fbxl5	0.000789843	2.615464926	down
1200002N14Rik	0.000790316	1.671218038	up
BC050254	0.000790882	1.68426764	down
Mpdz	0.000790882	1.670507431	down
Zfp364	0.000790882	1.930966616	up
Slc25a37	0.000792096	1.658671856	up
Pabpc1	0.000792471	1.69429946	down

Whsc1l1	0.000792471	2.135353565	up
Cetn3	0.000793806	1.552112699	down
Chchd10	0.000793806	1.82547617	up
Senp6	0.000793806	1.505150318	up
Vezt	0.000793806	1.973753214	up
7530408C15Rik	0.000794091	2.154988527	up
Chic2	0.000794264	2.126761437	down
Fbxo9	0.000794615	1.676539898	down
Tcfap2b	0.000796086	1.707800388	down
Ifitm3	0.000796482	2.425762177	up
Katnb1	0.000798825	2.002580404	up
Slc38a5	0.000799893	2.179512024	up
Psen1	0.000800288	2.081907511	down
Tcp1	0.000800855	1.741256356	down
Muted	0.000801829	1.613294721	down
Rnf40	0.00080243	2.316283464	up
Atl2	0.000805787	2.101182938	down
Tmem126a	0.00080744	1.742002964	down
Usp39	0.000808532	1.534889579	down
H47	0.000810973	1.856332779	down
Nckap1	0.000810973	4.134448052	down
C130039E17Rik	0.000812266	2.067170858	down
Diap2	0.000812738	1.756209016	up
Zfp335	0.000812913	2.123939514	up
Bicd2	0.000814141	1.517958641	up
Aldh1a3	0.000815524	1.922503591	down
Zfp85-rs1	0.000818611	1.522624373	down
Brp16	0.000818751	1.706869483	down
LOC215999	0.000818751	1.535746932	up
Il17rb	0.000820101	1.540724754	down
Papola	0.00082012	2.137450934	down
1110058L19Rik	0.000821078	1.525534511	down
C2	0.000821078	2.389507294	up
Fmr1	0.000822589	1.931776524	up
Ube2g2	0.000822858	1.798889637	down
Pde7b	0.000823892	2.308328867	down
Ak1	0.000824548	1.549378395	up
Rdh14	0.000824548	3.158828259	down
Tsen15	0.000824548	2.167198658	down
Mcm3ap	0.000825224	1.855196714	up
Crabp1	0.000825927	2.721472025	up
Slc12a5	0.000827682	1.775521398	up
Sox6	0.000827682	1.792667627	down
Tmem59	0.000827682	1.704159975	down
Ugt1a10	0.000827996	2.830978394	up
9430032L10Rik	0.000828083	1.667273998	up
Pscd1	0.000831053	1.6057266	down
Tspan17	0.000831053	1.61040771	down
D6Wsu176e	0.000833452	2.482609749	down
9630026C02Rik	0.000834379	1.957079411	up
Gfpt2	0.000835337	1.507639051	down
AI452372	0.000840292	1.674381852	up

LOC100041896	0.000844217	1.653958678	down
Zkscan5	0.000844217	1.659579396	up
Efnb3	0.000844946	1.549831986	up
Epn2	0.000846182	1.793093205	down
Art3	0.000847402	1.571328759	down
Pld4	0.000847402	4.97877264	up
Slc25a3	0.000847699	1.657124519	down
Nmi	0.0008498	2.010234594	up
Pygb	0.0008498	1.859453082	up
Rag1	0.000850037	1.618004084	up
LOC236371	0.000850748	2.055670023	down
Myh10	0.000850748	1.793132901	down
Pik3r3	0.000850748	2.169127703	up
scl0002533.1_1	0.000850748	1.86837697	down
Nog	0.000850948	4.01401329	down
EG243642	0.000852483	1.507992506	down
D030022P06Rik	0.000854854	1.604402542	up
9130011E15Rik	0.000855373	1.630361676	down
Kcnu1	0.000855373	1.704715967	up
Rbm28	0.000856192	1.936598539	up
Snapin	0.000858904	2.041892767	up
Fez2	0.000860843	1.603789568	down
Mbd6	0.000861344	1.573334336	up
LOC100048710	0.000863888	2.228545904	up
Ncrna00117	0.000863888	1.673356652	down
LOC100047199	0.000865372	1.809193492	down
Slc25a45	0.000865653	2.548929215	up
Uhrf2	0.000865653	2.204324007	down
A930006A19Rik	0.000867047	2.320179939	up
Ndufb2	0.000872248	1.524328113	up
Zhx1	0.000873219	1.706879616	down
Six6os1	0.000873456	1.76849699	up
Aplnr	0.000874723	1.825223804	up
Unc13c	0.000877608	1.754839301	up
Phf12	0.000878843	1.59600687	up
8430419L09Rik	0.000879268	1.509615302	down
LOC621824	0.000880059	1.693441272	down
Vcl	0.000880315	1.886447668	up
LOC386298	0.000880532	1.645327568	up
Dctn2	0.00088103	1.706122041	down
AK122525	0.000882149	1.713338494	down
BC048355	0.000884383	2.263658047	down
Cnot3	0.000884675	1.997102022	up
LOC100046781	0.000884787	3.487815142	up
Cdc14a	0.000885033	1.981531024	down
LOC100040671	0.000885856	2.356873512	up
Tyms	0.000885856	2.210950136	down
Crabp2	0.000886363	1.603922963	up
Trim28	0.000886363	1.84439528	up
2610002M06Rik	0.000888345	2.476204872	down
Zfp612	0.000888345	2.382030249	up
0610007P08Rik	0.000892943	1.564627409	down

B3galnt1	0.000893446	2.335953236	down
Ptprz1	0.000894356	1.908211827	down
Ifi30	0.000894615	1.624698877	up
2310036D04Rik	0.000897974	1.517827272	up
Crebbp	0.000897974	1.812695622	up
Grik1	0.00090444	1.551676989	up
LOC100044576	0.00090476	2.048181295	up
LOC386164	0.000907189	2.17485261	up
Dph5	0.000907541	1.571827173	up
scl0002997.1_15	0.00090781	1.546576738	down
Trerf1	0.000909732	1.797348023	up
Rbm15	0.000910276	2.360110521	up
Gripap1	0.000911517	1.921852946	up
Mbtps1	0.000912874	1.951483369	up
Hdac5	0.000916087	2.052684307	up
Ckap2l	0.000916513	2.170403004	down
Crispld2	0.000916513	2.249298334	up
Jmjd2a	0.000916513	2.543379068	down
Ddit4l	0.000917263	1.81448555	up
1810020G14Rik	0.000917364	2.404939652	down
6530401I16Rik	0.00091805	1.822309375	down
Arhgef18	0.000918659	1.762944222	up
Hspa9	0.000918659	1.570945978	down
Kifap3	0.000918659	1.679756284	down
Ets2	0.000920575	1.974570274	down
Palm	0.000921832	2.137369633	up
EG432721	0.000922153	1.830411673	down
Ptpre	0.000924508	1.759332657	up
A830042C15Rik	0.000924703	1.60347414	up
Gbas	0.000924703	1.902266145	down
Hmga2	0.000924703	2.021983624	up
Misc12	0.000924703	2.155719996	up
D130066L18Rik	0.000928041	1.604323268	up
Kdelr3	0.000928076	1.755330801	down
Phlda1	0.000928076	2.164865017	down
LOC386199	0.000928797	2.699589014	up
5730538E15Rik	0.000931173	1.607057929	up
Terf2ip	0.000931173	1.758431196	down
A530092L01Rik	0.000931208	1.771546483	up
Psmf1	0.000933279	1.524743795	up
2010309L07Rik	0.000935799	1.821907044	up
Cog8	0.000935799	1.515040636	up
LOC100046770	0.000935799	2.553110123	down
Nip7	0.000935799	4.187808514	down
Pitpnc1	0.000935799	2.315482378	down
Tnfrsf13b	0.000935799	1.708664775	up
Zscan21	0.000935799	2.050704002	down
Shmt2	0.000935829	3.164767742	down
C030044C12Rik	0.000937239	2.230243206	up
Asph	0.000937887	1.685213924	down
Ncf4	0.000937887	2.664585829	up
Ube2q2	0.000937887	2.607199192	down

4930535I16Rik	0.000938402	1.566395164	down
Msl2l1	0.000940063	1.755577087	down
Dgcr8	0.000940667	2.143640995	up
Xab2	0.000940908	2.177684069	up
Edem1	0.000942904	1.67919004	down
A230075M04Rik	0.00094338	1.660775423	down
Lilrb4	0.00094342	1.830344677	up
Adck5	0.000945435	1.571097732	up
Nek8	0.000945435	1.720896721	up
LOC546133	0.000946278	1.652725697	up
Cmpk	0.000946831	1.773629785	up
Spc24	0.000946831	1.910340905	down
Tmem161a	0.000946831	1.731407762	down
Ddx47	0.000949328	2.307098389	down
Rexo2	0.000949328	2.244694471	down
Cd276	0.000949608	1.84107089	up
Arpc1a	0.000951765	2.271959782	down
Gtdc1	0.000951861	2.286889792	down
Clcn3	0.000953132	1.64994669	up
Mrps33	0.000953132	1.913109779	down
Ccne1	0.000955471	1.816719413	down
Snx1	0.000955471	1.925142765	up
D030028A08Rik	0.000956366	1.591829777	up
Zfp125	0.000956482	1.774166942	up
LOC668183	0.000963151	2.390392065	down
Prdx2	0.000963151	2.261679411	up
Tmem100	0.00096354	1.629628658	down
D630024O03Rik	0.000965172	5.263995171	up
Arl10c	0.000965403	1.75693357	up
Myo1g	0.000965757	2.697003603	up
LOC383850	0.000966677	1.89019084	up
LOC269515	0.000967978	1.556047678	down
Whsc2	0.000968416	1.659742832	up
LOC100046008	0.00096893	1.54093504	up
Pmm2	0.00096893	2.219312668	down
6030438J01	0.00096953	2.103323221	up
Cldn10	0.00096953	1.68889761	down
Ifitm1	0.000969837	2.014776468	up
Zmym6	0.000973584	2.57755208	up
Dok4	0.000974923	2.058350802	up
D2Ertd391e	0.000975583	2.232372046	up
Zfp61	0.000976345	1.989290118	up
Npm3-ps1	0.000976988	2.108114243	down
Acta2	0.000979501	2.506484985	down
Fam181b	0.000979501	1.706959963	down
Gpx7	0.000979501	2.005330801	down
scl0003476.1_2802	0.000982393	2.157479048	up
Slc39a9	0.000982393	1.837125421	up
Oxct1	0.000983893	2.150996447	down
Nsmce2	0.000984079	4.732778072	down
Plekhf1	0.000984974	1.727775216	down
Dbt	0.000987097	1.782038689	down

Rad51c	0.000988342	2.507941961	down
Acad10	0.000988702	1.618595839	up
Rwdd4a	0.00099017	1.693397045	up
Yeats2	0.000994034	1.560504913	up
Lphn1	0.000994251	3.276886225	up
2310022B05Rik	0.000994383	1.517957449	up
W91709	0.000994383	2.12598443	up
Mettl4	0.00099516	1.525905252	down
Hist1h4d	0.000995451	1.672791123	down
Crtc3	0.0009964	2.221070528	up
Otud7b	0.000997051	2.473006249	up
Stx6	0.000998389	1.746162176	down
Rit2	0.000998491	2.271516562	up
2210038L17Rik	0.000999456	1.976977468	up
1600021P15Rik	0.001000329	2.081422806	down
Dctn1	0.001004407	2.952730417	up
Xpo7	0.001004546	1.902608395	down
Bbs9	0.001004716	1.631353974	down
Camk1d	0.001004889	1.549100399	up
Mcart6	0.001007106	1.954837203	down
Arl6ip5	0.001007404	1.767628431	down
LOC382691	0.001007404	2.771187067	down
Tsc22d3	0.001010016	1.754339337	down
Ing4	0.001013749	1.919811487	up
2010311D03Rik	0.001014041	2.806665421	down
Arl11	0.001015426	2.136006832	up
Zfand2b	0.00101671	1.578392267	down
Relb	0.00101733	1.525190473	up
scl0002493.1_0	0.001017962	1.553257108	down
E130301L11Rik	0.00101824	2.675470829	up
scl000707.1_0	0.00101964	2.520102978	up
Gins4	0.001020148	1.642088294	up
Hira	0.00102057	1.93010962	down
Adamts9	0.001022134	1.56271708	up
F3	0.001022134	1.758131027	down
Prkd1	0.001024089	1.653883219	down
Nudt3	0.001024555	2.070083618	up
C430045L21Rik	0.001025426	1.914214134	up
Cks2	0.001026336	1.945866227	up
2700063G02Rik	0.001027303	1.717841029	up
Nfat5	0.001027303	1.673508048	up
Slc3a2	0.001027303	2.600226402	up
Naprt1	0.001027489	1.555171847	down
Parp9	0.001028591	1.737879396	up
Siah2	0.001028778	1.570129395	down
Reck	0.00103131	1.91040349	down
Zfp317	0.001031421	1.669347167	down
Hnrnpul1	0.001031442	1.750171661	down
LOC622901	0.001031671	1.571720839	up
5830427D03Rik	0.001035344	2.215423584	up
CKLF5	0.001035684	1.560355544	up
Trap1	0.001036756	1.591694832	up

Wdr37	0.001037843	1.907283664	down
4930513N10Rik	0.001038607	1.533799529	up
Hmbs	0.001039323	2.112030506	up
Rtn4ip1	0.00104094	1.805151105	up
Flrt1	0.00104106	1.882780433	up
Abcd4	0.001041508	1.616673231	up
Drg1	0.001041508	2.441031933	down
Fam168a	0.001041508	1.668094993	up
Upf1	0.001041508	1.780835986	up
Myo9a	0.001043189	2.053853989	up
Gm106	0.001044875	2.287182808	down
1110018J18Rik	0.001045462	2.008842945	down
Mfsd3	0.001045462	1.808592677	up
EG626841	0.001046873	1.532622218	down
Cnot7	0.001048682	1.924594402	down
Cd68	0.001050191	5.700698376	up
Fut10	0.001051545	1.734608173	up
Rpl21	0.001051545	1.564572334	down
Them2	0.001053505	1.627916813	up
LOC100038887	0.001054136	2.23310113	down
Cotl1	0.001056145	2.605662823	up
Nanos1	0.001056145	1.562861919	down
Wrn	0.001056145	1.957185507	up
Gtl2	0.001056454	1.683142543	up
D4Wsu132e	0.001058019	1.664388657	down
Lcmt1	0.001058587	2.248296022	down
Tnnt2	0.001059862	1.675191402	up
E430003D02Rik	0.001060545	1.925400138	down
Exosc10	0.001062018	1.675004721	up
Mylip	0.00106363	1.537615657	up
LOC100048530	0.001064473	2.146600485	up
BC065120	0.0010652	1.674632192	down
Rab2b	0.001065661	1.590023994	up
Timm44	0.001065946	1.536224008	up
Bahcc1	0.001066029	1.527143121	down
P2ry13	0.001066967	2.462240934	up
Nacc1	0.001067763	1.889197469	up
Hist1h2bf	0.001070402	1.827791691	down
Adck4	0.001073073	2.188557625	up
9626962_1_rc	0.001073401	3.024059534	up
Rpl23	0.001073897	1.558342934	up
Mier3	0.001074366	2.594699621	down
A930019J01Rik	0.001074904	2.279903412	down
Mrpl15	0.001074904	2.821289778	down
Usp5	0.001076855	1.543330193	up
Snx33	0.001080176	1.719529152	up
scl0002275.1_1	0.001081781	2.36147666	down
Tmem101	0.001082365	2.014776707	up
Eif3k	0.001082547	1.668414354	up
Insig2	0.001082784	2.227345228	down
Postn	0.001082874	4.589205265	down
LOC383153	0.001083232	1.526769042	down

Dgcr2	0.001083652	1.656951189	down
D630030B22Rik	0.001084831	1.500329733	up
Tor1b	0.001085869	1.668402076	up
Dedd	0.001087353	1.941467285	down
Etfa	0.001088822	2.289352417	down
Wbp2	0.001088822	1.767673254	down
Cttnbp2nl	0.001089024	1.536500096	down
Aarsd1	0.001089184	1.993360043	up
B930097J01Rik	0.001089184	2.073749781	up
EG546052	0.001089184	1.985993862	down
LOC666053	0.001089184	2.020558834	down
Ttc28	0.001089596	2.133371592	up
Mmp11	0.001090527	2.097203016	up
LOC674195	0.001090818	1.927259088	up
Adamts10	0.001093751	2.813961983	up
Osr2	0.00109451	1.955943227	down
Arpc1b	0.001095492	1.532239556	up
Cep27	0.001096554	1.760466456	down
E330016A19Rik	0.001098942	1.755437613	up
Ppard	0.001098942	1.571586847	down
5830454D03Rik	0.001099095	1.808461785	up
E130118E17Rik	0.001103729	2.198096275	up
Tle3	0.001104039	1.855359912	up
Pacrgl	0.001104756	2.000694037	down
2610039C10Rik	0.001105036	3.718868494	down
Eif4e	0.001106951	1.603933334	up
Rbmxrt	0.001108419	2.461699724	up
Pias3	0.001110806	2.097896099	up
Herc1	0.001112086	1.521829844	up
Tmub2	0.001112086	1.873716712	up
2810410M20Rik	0.001113105	1.58388257	down
Wwp2	0.001114356	1.589534521	down
Grb10	0.001116259	2.013310194	up
Ryr1	0.001117471	1.881572962	up
Rapgef6	0.001119049	1.578709483	down
Sec16a	0.001119572	1.556519032	up
A730094H17Rik	0.001119986	1.957684398	up
C130090G16Rik	0.001119986	1.693909764	up
Zmpste24	0.001119986	2.255541563	down
Igf1	0.001120033	1.768460631	up
Mpnd	0.001120451	1.708215952	up
scl000568.1_1444	0.001121015	1.57271421	up
Supt16h	0.001121015	1.628678083	up
LOC100046298	0.001122942	2.111520052	down
Rab8a	0.001122942	1.560746551	up
LOC100048079	0.001122957	1.628016472	down
LOC100039461	0.001123953	1.772439003	up
D930014E17Rik	0.001125041	1.504359603	up
Actr1b	0.00112538	1.696915984	up
Arfip2	0.001126039	2.38974452	down
Stau2	0.001127039	1.965145826	down
2610507B11Rik	0.001127766	1.654443502	up

Add1	0.001127766	2.574988604	down
Tmepai	0.001127766	1.643532395	down
9530018D06Rik	0.001129034	3.243758917	up
Casp9	0.001129449	1.65629518	up
Cdc6	0.001129449	1.688206434	down
Ctr9	0.001129449	2.080210924	up
Krt18	0.001129449	1.601678252	up
Lpgat1	0.001129449	1.891710401	up
Trmt2b	0.001129449	1.662264347	down
Tubgcp5	0.001129449	1.500069141	up
LOC664892	0.001130711	1.676155567	up
Arl3	0.001134069	2.866230488	down
Cyp39a1	0.001134069	2.22275424	down
Slc35a3	0.001134286	1.659265637	down
Ippk	0.00113465	1.510338426	down
C920011G20Rik	0.001135894	1.925225973	up
Uap1l1	0.001138886	2.030499697	up
Ptpn6	0.001140405	3.077181816	up
6720463M24Rik	0.001142289	1.882989645	down
Ogdh	0.001142302	2.207346439	up
Ehd4	0.001142902	2.178522587	up
Kdelr3	0.001149642	2.137759924	down
Hoxc10	0.001150567	1.764403105	up
Oraov1	0.001150842	2.207996607	up
Ptprm	0.001150842	1.623142839	down
A130010C12Rik	0.001151636	2.763610363	up
1810046K07Rik	0.001151771	2.342598677	down
C1qtnf6	0.001151771	1.661123037	up
Smarcc1	0.001154293	2.587359428	down
9845300_4289	0.001156944	2.811022043	down
Dock5	0.001156944	1.916880131	up
Luzp2	0.001159602	1.675012708	down
Etfdh	0.001160579	1.693230391	down
Gp5	0.00116249	2.846934319	up
A630036P20Rik	0.001163716	2.148339748	up
Zfp101	0.001167731	1.873557925	down
AI851790	0.001168923	2.56488204	down
Rnf20	0.001169237	1.661683679	up
BC059842	0.00117052	1.788316131	up
Ndufa13	0.00117052	1.551068187	up
LOC100044190	0.001170721	1.664128423	up
Itfg3	0.001171949	1.844382405	down
B230104P22Rik	0.001173838	1.806749582	up
Rhou	0.001174149	2.100575924	up
Adnp	0.001178889	5.289130688	down
Ms4a6b	0.001182045	2.225795984	up
Rnf4	0.001184763	2.401705504	down
Stk39	0.001185332	1.816866875	down
Bbs5	0.001185681	1.786554575	down
Bxdc2	0.001186701	2.773817301	down
8030448K23Rik	0.001187263	1.519899726	up
Dhx58	0.001187317	1.745324373	up

EG216185	0.001190561	1.577683449	down
Eno2	0.00119091	2.091520786	up
C330046E03	0.001192985	2.761460543	up
Idi1	0.001192985	1.85785687	up
Mest	0.001193412	1.687818408	down
Ppt2	0.001194181	1.515129924	down
Ccl9	0.001197292	6.151591778	up
scl0003949.1_31	0.001197571	2.054114342	down
Calca	0.001198601	1.879122376	down
Hipk2	0.001198601	2.047196627	up
Hist1h3f	0.001201033	1.840523362	down
Ccdc5	0.001202335	1.62722683	up
Zfp212	0.001205064	1.646409631	down
Trf	0.001205189	2.72731328	up
Acpl2	0.001208577	2.482869387	down
0610010I05Rik	0.001208631	3.44824934	down
1110001C23Rik	0.001208801	1.932467699	up
Hlcs	0.001209013	1.634361148	up
5031439G07Rik	0.001210541	1.632893682	up
9430047G12Rik	0.001210779	3.430176973	up
D230045O07Rik	0.001211126	2.506356001	up
Ddx50	0.001211126	3.383285999	down
LOC100047856	0.001211151	1.56641233	down
D330037A04Rik	0.001214775	1.881937504	up
Mdm1	0.001215128	1.97629261	down
2310003L22Rik	0.00121827	1.681576014	up
Ankrd6	0.001220926	1.580711484	down
2810474O19Rik	0.001222105	2.274237633	up
E430012K20Rik	0.001225638	1.590662479	down
Abca9	0.001226893	1.846090078	up
Cask	0.001228946	1.625333309	up
Smpd4	0.001229867	1.671432376	down
Mtrr	0.001231021	1.812602162	down
Phb2	0.001237359	1.5124048	up
Agfg1	0.00123762	1.679559112	down
2310010J17Rik	0.001238259	1.538585901	up
Fv1	0.001238294	1.607164264	down
Ccl24	0.001239796	3.242741585	up
Arid3a	0.001240479	2.460443974	up
Dst	0.001240479	1.81259644	up
Polr2d	0.001240479	2.717124939	down
AU019823	0.001243073	1.733773112	down
Amy2-2	0.001245599	1.824177146	up
LOC386117	0.001245599	1.71206975	up
Prim2	0.001245599	1.800039053	down
Parp3	0.001249813	1.874984503	up
Dtx2	0.001249833	2.078676939	down
Sap130	0.001253148	1.744393587	up
Tfdp2	0.001253148	1.684639573	down
1600016N20Rik	0.001255958	1.926030755	up
scl0004020.1_31	0.001256218	2.505968809	down
Atp13a1	0.001256327	2.145743609	up

Exoc8	0.001260483	1.648089409	up
scl0003749.1_1	0.001260986	1.539248109	down
Abcd1	0.001261477	1.810402155	up
Hmgb2l1	0.001262462	1.711944699	down
Impact	0.001262462	1.725575924	down
Taf6	0.001262462	1.526983738	up
Hcls1	0.001266285	2.485381603	up
Lpp	0.001267126	2.138572931	up
Hist2h2be	0.001268526	1.951281548	down
D030019N20Rik	0.00127181	2.69084692	up
Zfpm2	0.001274669	1.900486469	down
LOC270589	0.001276972	2.150870085	up
Tlk1	0.001276972	1.675049186	up
2810403A07Rik	0.001279505	1.580392003	up
9630009A08Rik	0.001279794	2.443462372	up
Hras1	0.001280754	1.683243513	up
C130020C07Rik	0.001282111	1.997501731	up
P4ha3	0.001282111	1.80704093	down
LOC631301	0.001284509	2.857666254	down
Tcf20	0.00128461	1.644688368	down
2700078K13Rik	0.001288092	2.503597975	up
Fts	0.001288092	2.210709572	up
Pnrc2	0.001288215	2.147199392	down
B3gntl1	0.001292241	1.502738714	up
Dnmt3a	0.001292241	1.655543208	up
0610007J10Rik	0.001295532	2.341670752	down
A930038D23Rik	0.001295744	1.629433036	up
Micall2	0.001295744	1.728844047	up
Spon2	0.001295895	2.381313324	up
Gstp1	0.001296265	1.726598024	down
Ccdc15	0.00129693	1.529206395	up
Gnai3	0.001297163	1.576985478	up
1810074P20Rik	0.001300037	1.614268303	down
Mbp	0.001300037	1.633033872	down
6720417O19Rik	0.001302543	1.72094667	up
Ggnbp2	0.00130291	1.699626327	up
A230065H16Rik	0.001304143	1.683574081	down
Cd93	0.001304143	2.563290358	up
Stx12	0.001304143	1.661991477	up
Prdm6	0.001309101	2.116293669	down
Trim30	0.001309101	2.020578384	up
scl0001118.1_0	0.001311197	1.874174118	down
Riok3	0.001313401	2.018571138	down
Sfrs11	0.001313607	2.210344076	down
Pik3r1	0.001316492	2.600860834	down
2510002J07Rik	0.001316965	1.505857825	up
LOC270017	0.001317781	3.687342405	up
Gltscr2	0.001317811	1.810175538	up
Psmb8	0.001318577	1.528303981	up
2610202A04Rik	0.001318712	1.565314293	up
Gpc2	0.001318712	2.041071177	up
Plxnd1	0.001319153	2.83500123	up

Abcc10	0.001319443	1.871612191	up
Psmd6	0.001319443	2.030457735	up
BC004728	0.001321819	3.255896807	up
Sgk1	0.001322732	1.599029541	down
4933407C03Rik	0.001322784	2.094254255	up
A630072J24Rik	0.001322986	2.36232543	up
AI894139	0.001325015	1.693979979	up
Rnf181	0.001326282	1.852025271	down
D630035D13Rik	0.001332618	1.799160481	up
LOC100044133	0.001333982	1.77037251	down
6330575P09Rik	0.00133553	1.683894873	up
Alx1	0.00133553	2.564602852	down
Eng	0.00133553	2.155508757	up
Ephb4	0.00133553	1.960762024	up
B230312E02Rik	0.001336744	1.554623842	up
Psme2	0.001338594	2.058163166	down
8430438I05Rik	0.001338704	1.692926049	up
Flii	0.001338732	2.172633886	up
Ift74	0.001339297	1.976718903	down
Thrap3	0.001345004	1.904495597	up
Cdc14b	0.001345496	1.703128696	down
Ehd4	0.001345496	2.332180738	up
Hoxc9	0.001346065	2.073279381	up
Hba-a1	0.001350424	13.63499546	up
Gsto1	0.001353281	1.542165399	down
Ndufb4	0.001353281	2.297720194	up
LOC386288	0.001358223	3.126948357	up
Slc11a1	0.00135934	2.419321537	up
Emg1	0.00136085	1.713222742	down
Rsdr1-pending	0.001363005	1.535302758	down
LOC635253	0.001363536	1.58672297	down
Myc	0.001363536	1.514363527	down
Prkrip1	0.001364302	1.878593087	down
Dgcr14	0.001364609	1.746639967	up
Syncrip	0.001365058	1.773158789	down
LOC100047007	0.001365279	1.627414823	up
Smarcc2	0.001366583	3.076368332	up
Uhrf1	0.001366686	1.690306664	down
Pcdhb16	0.001367903	1.891284585	up
Ptges2	0.001368286	1.518815756	up
Slc12a9	0.001368352	1.710175157	up
Yipf6	0.001372094	1.743624806	down
D930030O05Rik	0.001373269	3.18283844	up
4930455F23Rik	0.001373571	1.867770433	up
Dab1	0.001373571	1.782429457	down
Madh1	0.001373571	1.813425064	up
Map3k3	0.001373571	2.210063934	up
9330171B01Rik	0.001373682	2.062614203	up
2900062L11Rik	0.001376657	1.796715021	down
Cdkn2d	0.001376657	1.792257905	down
Slc7a7	0.001377132	1.755987525	up
Cfb	0.001382249	1.619415164	up

Igf2as	0.001382283	1.710151911	up
Atpbd3	0.001384235	1.517065406	down
Hdhd2	0.001384235	2.030791283	down
Pcbp3	0.001384798	1.630687714	up
Fscn1	0.001384939	2.383372784	up
Cep350	0.001386702	1.842551589	up
A330017A19Rik	0.001390259	1.877308846	up
Wwc2	0.001390259	1.583288193	up
D030016E14Rik	0.001390404	1.644117117	down
Xylb	0.001393348	1.574090004	up
Fig4	0.001394433	2.071825981	up
LOC100047369	0.00139817	1.643435121	up
Mgst1	0.00139817	2.017282248	down
Gria3	0.001398645	1.739444256	up
1810015C11Rik	0.001400115	2.128852367	up
Gucy1a3	0.001400137	2.281820059	up
2010204K13Rik	0.001400432	2.177839518	down
Btk	0.001400432	2.97397089	up
Lsr	0.001400432	1.549177051	down
Pstpip2	0.001400432	1.54054749	down
Rchy1	0.001400432	1.662866712	down
Srp9	0.001400432	2.077630997	down
Spty2d1	0.001401624	1.679500341	down
Nrg1	0.001402922	1.833201647	up
Fus	0.001404416	3.304260731	down
Nat11	0.00140502	1.606286287	down
A930013F10Rik	0.001405513	2.469285727	up
Actb	0.001405513	2.797592163	up
Col4a2	0.001405513	1.671015263	up
Trio	0.001406998	1.626712561	down
Bcl11b	0.001408223	1.845429659	down
2310033P09Rik	0.001409224	1.560455442	up
Cpa3	0.001410989	3.052464247	up
LOC380756	0.001410989	1.993102431	down
Smox	0.001411792	1.739189863	down
Thsd1	0.001411792	1.6396209	up
Hs3st3b1	0.001414025	1.865917325	down
Fip1l1	0.001418474	1.568021417	down
Nme4	0.001419213	1.616770983	down
Med18	0.001424609	1.59346962	up
Nedd4l	0.00142604	1.516248822	down
E430024E16Rik	0.001428528	1.655447125	up
Rhpn1	0.001433004	2.106686354	down
4933400F03Rik	0.001436777	1.712092638	up
Uck2	0.001437029	1.915061951	down
Vdac2	0.001437398	1.665377498	up
Dock2	0.001439338	1.638944745	up
Fundc1	0.0014394	1.708661199	down
Abhd1	0.001441544	1.55307734	up
Ptprb	0.001441753	2.290420294	up
Stard10	0.001442281	1.654127836	down
Stxbp4	0.001442281	1.900921226	down

Ankrd27	0.001443499	1.930335403	down
Vwa2	0.001444164	1.516442657	down
Tln2	0.001444437	1.781723261	up
Polr2d	0.001446054	2.792487383	down
Fbxo25	0.001446564	1.952106476	up
Sncg	0.001448643	1.57569468	down
Adpgk	0.001452042	1.868386626	up
1110029I05Rik	0.001452192	1.935266972	up
LOC385046	0.001453555	1.635743022	down
1200003I07Rik	0.001456614	2.027455807	up
1600010O03Rik	0.001456614	1.922224522	up
Scnm1	0.001456614	1.573735237	up
Col12a1	0.001459592	1.992657542	down
Hoxd9	0.001459709	1.69859314	up
Creld2	0.001460808	2.021269321	down
Ankrd26	0.001461533	1.563565016	down
Mpv17l	0.00146227	1.529810667	up
Rps3	0.001463545	1.836743236	down
Usp12	0.001468573	1.608047366	down
Fbp2	0.001468936	2.343615055	down
Asb2	0.00147255	2.209375143	up
Uqcrh	0.00147255	1.941877365	down
E130301F21Rik	0.00147392	2.725469589	down
Hint3	0.001475331	1.692176342	down
Sash1	0.001479993	2.057686567	up
Dio2	0.00148066	2.13854003	up
Ddt	0.00148402	1.562228441	up
LOC624662	0.001485834	1.810651779	down
A730095J18Rik	0.001487465	1.938135266	up
Tsc1	0.001489082	2.129157066	up
4732431J01Rik	0.001490197	1.749461055	up
Dlgap4	0.001490197	1.550408244	up
LOC671399	0.001490197	1.963004947	down
Ugt1a10	0.001490585	3.018768311	up
2010009J12Rik	0.001491783	1.550962091	up
Epc1	0.001492383	2.106541634	down
3110038B19Rik	0.001495229	1.657213926	down
Ipas	0.001495229	1.907285929	up
2310065K24Rik	0.001496167	1.668307185	up
Tmem189	0.001496167	1.80518496	down
Wbp7	0.001496209	2.01599741	up
Luzp1	0.001497659	2.358450651	up
2810017I02Rik	0.001497711	1.861921787	down
H2afx	0.001497711	1.630644798	up
0610010F05Rik	0.001499229	2.255190849	down
Aph1b	0.001500445	1.798661828	down
Col4a5	0.001502058	2.163853407	down
Ndufa1	0.001502058	1.57775116	down
B230353O14Rik	0.001507869	1.509917855	up
H2-T10	0.001509044	2.040434122	down
Azi2	0.001511271	1.792396307	down
Ahrr	0.001512735	1.562556505	up

Gmds	0.001513188	1.810869336	down
Atp8a1	0.001513474	1.579180002	up
LOC382776	0.001513474	1.595616579	up
LOC638031	0.001514276	1.805624962	up
Slc7a8	0.001515493	1.810862303	up
Tgfbr1	0.001516419	1.533280373	down
Picalm	0.001519016	1.597013712	up
B830008M09Rik	0.001519308	1.869398952	up
LOC237459	0.001519308	2.144380093	down
LOC666979	0.001519308	2.829375744	up
H3f3b	0.00152088	1.547369123	up
Parl	0.001524098	2.023743391	down
Tulip1-pending	0.001525456	1.561953783	up
Dlgap4	0.001525499	1.559213519	up
Tpcn2	0.001526689	1.936539292	up
Cdk5rap3	0.001527603	1.753373385	up
Klhl22	0.001530132	2.581550598	down
5330401P04Rik	0.001532777	1.76407373	down
Atp9b	0.001533461	2.144056559	up
Bsg	0.001536872	2.248334885	down
C330011F01Rik	0.001536872	1.511134982	up
Dgkh	0.001536872	1.762007713	down
Gm70	0.001536872	1.702488422	down
Tarbp2	0.001536872	1.884200335	down
Gps1	0.00153846	1.877121568	down
Doc2a	0.001543539	1.904206038	up
Eif2a	0.00154423	1.829097509	down
Atp6v0e2	0.001550707	3.159582138	down
Fbln2	0.001551942	2.287607193	down
Fbxl7	0.001551942	2.45767808	up
Pax2	0.00155239	2.120502472	down
Sox5	0.00155394	3.311142206	down
2610528E23Rik	0.001555066	1.52293396	up
Srd5a1	0.001557619	1.714048743	down
Sdpr	0.001563412	2.12800312	up
Sdccag33l	0.001564027	1.521818161	up
Mtus1	0.001568072	1.660579801	up
2310022M17Rik	0.001569734	1.829479933	down
AI606181	0.001570328	1.844905257	up
9330134C04Rik	0.001571285	2.420418739	down
Psme4	0.001571285	2.324461222	down
Ddx10	0.001571323	1.621686459	down
LOC386124	0.001571323	3.609391689	up
Brwd3	0.00157639	1.557183862	down
LOC100047369	0.001578806	1.73669529	up
Snx7	0.001579605	1.729214668	down
Bub3	0.001580322	1.503022194	up
Slc25a36	0.001580945	2.071575403	down
Ppcs	0.00158126	1.646772623	down
LOC100042970	0.001585056	2.779963017	down
Zfp251	0.001585056	1.50702095	down
Dmrta1	0.001586785	2.103105068	down

Tada1l	0.001586785	1.913601399	down
Prrc1	0.001589601	1.73284173	down
A130024K02Rik	0.001589847	1.610862851	up
Stub1	0.001589847	1.555882931	up
Rnf41	0.001590438	1.687227964	down
Tmem17	0.00159099	1.602697849	down
A430010E21Rik	0.001591117	1.846939087	down
Arfgap2	0.001593373	1.901801586	down
LOC385923	0.001595054	3.489911318	up
Scrn2	0.001599784	1.905384302	up
Pafah2	0.001600318	1.688534498	down
Eef1b2	0.001600423	2.762474537	down
Atxn2l	0.001602753	1.801717997	up
Sall2	0.001602753	1.735968947	up
2410004N11Rik	0.001604019	2.347200871	down
Zfand1	0.001604603	1.753351569	up
LOC100042405	0.001605056	2.442533732	down
Pdcd10	0.001605056	1.546079278	up
Atp1a2	0.00160584	1.724053264	down
BC018507	0.001606414	2.608114958	down
Gng12	0.001613297	1.896968961	down
Jarid1a	0.001613297	2.408887625	down
Gtf2ird2	0.001614828	1.557289839	down
Taf6	0.001614828	1.530599833	up
Fv1	0.001618028	1.66173625	down
LOC100043821	0.00161852	2.191907167	down
Slit2	0.001618815	2.298761606	up
Zfp710	0.00161959	2.375598192	up
Sugt1	0.0016197	2.026542902	up
Marco	0.001620732	2.083778143	up
1110003F10Rik	0.001620799	1.544133306	up
BC020108	0.001620799	1.996539354	down
2410004P22Rik	0.001621177	1.884745359	up
A130032P05Rik	0.001621177	1.935186505	down
Cytip	0.001621177	1.794008255	up
Dctn4	0.001621177	1.88503623	down
Homer1	0.001621177	2.069010735	down
Osr1	0.001621653	1.59414053	up
Pigq	0.001623747	1.809014201	up
Cav1	0.001625245	2.208571196	up
Pik3r4	0.001629403	1.725981116	up
Grn	0.001631639	1.720488429	up
Mrpl13	0.001635385	1.606062293	up
2010320M18Rik	0.001638621	1.842648506	up
LOC100036521	0.001638621	2.506390095	down
Sema6a	0.001638621	1.69858098	down
Vegfc	0.001640356	1.642066598	up
Pdha1	0.001641727	1.731289983	down
Numa1	0.001642959	1.78566432	up
Tmem121	0.001643751	1.742707968	up
Chd1	0.001644403	2.344444752	down
Mettl9	0.001644471	2.650498867	down

Peg3	0.001648711	1.990690827	down
D130027A21Rik	0.001651892	1.68556881	down
Tcf25	0.001659302	1.569068074	up
Rab10	0.001659577	1.990112066	down
Dolpp1	0.001660539	1.761424422	down
1810063B07Rik	0.001663115	1.734813452	up
Pdk3	0.001665692	1.569211483	down
LOC211870	0.001668349	1.52379477	down
Olfml2a	0.001669301	1.618049026	up
Gsdmdc1	0.001669409	1.638996959	up
Setd8	0.001671291	1.635463357	up
Nfkb2	0.001673096	1.831693173	up
9630055A16Rik	0.001673713	1.656186342	up
C230040D10Rik	0.001677332	2.345288515	up
Cyfip2	0.001679672	2.030926466	up
Ano10	0.00168038	1.689085364	up
Med24	0.001681839	2.157135248	up
Entpd5	0.001684655	1.689548492	up
E030003F13Rik	0.001690843	1.536672115	up
Nit2	0.001696434	1.530493498	down
Bcl7b	0.001696552	1.55273211	up
LOC100045542	0.001697203	1.512580514	down
Ntn2l	0.001697203	1.532775641	up
Dnajb6	0.001700736	2.975131273	down
Tcof1	0.001700736	1.891676545	up
Dleu7	0.00170201	1.752730727	down
Fpgt	0.00170201	2.187808037	down
Hoxa9	0.001705041	1.513581157	down
LOC100047226	0.001705268	6.351510525	down
Hnrpll	0.001706767	1.927691698	down
Tgfbi	0.001709419	2.340261459	down
Nt5c3l	0.001714637	2.24591589	down
Itch	0.001718073	2.403063536	down
Cugbp1	0.001720298	1.709599137	down
EG329521	0.001720472	1.981694102	up
Zfp292	0.001722273	1.720644236	up
Piasg-pending	0.001723063	1.719383478	up
Basp1	0.00172614	1.86092639	down
Gal3st1	0.001727437	2.106734753	down
Trim23	0.001730253	1.913262248	down
Tbkbp1	0.001732204	1.849165797	up
Caskin2	0.001732909	1.535313368	up
EG434402	0.001733374	1.599641442	up
Csgalnact1	0.001735196	1.569012284	up
Aurkb	0.001735869	1.863275528	down
LOC677375	0.001735959	1.691161037	up
Nudt22	0.001735959	1.868268132	up
Mgat1	0.001736927	1.678864241	up
Sgk3	0.001737422	1.572519541	down
Slc25a28	0.001737422	2.085822582	up
Txnl1	0.001738417	1.59683907	up
Ikzf2	0.001738703	1.786896586	up

Mme	0.001738753	1.818969607	down
Mdm1	0.001739094	1.698763251	down
Tug1	0.00174318	1.609519005	down
Hoxa13	0.001744016	1.642590165	down
Znfx1	0.001745367	1.606839299	up
Fam134c	0.001749054	1.92955792	down
A730070K21Rik	0.001749973	1.934476972	up
Pdia4	0.001750231	2.300646305	down
Pex5	0.001750474	1.527123094	down
EG666577	0.001751881	2.477158308	down
Il10rb	0.001754641	1.542899132	up
LOC100047113	0.001754641	2.024441004	up
Zc3h14	0.001757435	2.326521874	down
Spen	0.001758503	2.516223431	up
LOC100047369	0.0017602	1.506572962	up
Gbf1	0.001761413	1.788582444	up
Solh	0.001762503	1.840117693	up
Fndc4	0.001764954	1.521905541	up
Gna14	0.001769265	1.7251333	down
Tipin	0.001770314	2.371179581	down
LOC676912	0.001770897	1.785675168	up
LOC667475	0.001772246	1.634959698	up
0610031J06Rik	0.001773255	1.684084058	up
C030025P15Rik	0.001781111	2.218602419	down
Coro1a	0.001782953	3.400266171	up
B4galt4	0.00178407	1.505052447	up
AI225934	0.001786163	2.097738504	up
Lrfn5	0.00179439	2.241804123	up
LOC671434	0.001795169	1.62929523	up
LOC633238	0.001796207	1.585783958	down
AI427122	0.001796627	1.602066994	down
Cul2	0.001796627	1.710577726	down
Slc36a1	0.001802321	1.776494861	up
Gnb5	0.001805841	2.050322056	down
LOC100039636	0.001810243	1.9667418	up
LOC381684	0.001813858	1.768771052	up
Ccdc28b	0.001816701	1.578166843	up
Baz2a	0.001817902	1.747635245	up
Atox1	0.001820736	1.708862066	up
Plcg2	0.001820884	2.247225523	up
Rpp30	0.00182152	1.884821892	down
Crim1	0.001823318	2.418887138	down
LOC100046406	0.001823318	1.998743773	down
Wdr24	0.001827229	1.558735728	up
3000004C01Rik	0.001827573	1.734568477	up
Gemin5	0.001827573	1.516962171	up
Tmtc2	0.001835532	1.787765741	down
Mapkapk3	0.001835664	1.658428311	up
Chd7	0.001836177	1.861780882	down
Atp5k	0.001837584	1.777037382	up
Tspan13	0.001837802	1.86756146	up
Cyp1b1	0.00183787	2.115233183	down

Mar-07	0.001839083	3.636689901	down
LOC327995	0.001847482	1.552106977	down
Luzp1	0.001848537	1.670756698	up
Herpud1	0.001848892	2.375756979	down
scl0004158.1_6	0.001849017	1.811247468	down
Pfn2	0.001851165	2.781931877	down
B230114H05Rik	0.001854865	2.035285711	down
Rasgrp4	0.001855768	1.526313663	up
LOC100047028	0.001856763	1.797136545	up
Rnu65	0.001856888	1.699950337	down
Pigu	0.001858055	2.101835489	down
BC027231	0.001858352	1.573701859	down
Evi1	0.001858352	2.445608139	down
0610007P22Rik	0.0018607	1.7022053	up
Iars	0.00186703	1.529809594	down
Smc1a	0.001867339	2.085639	down
Sh3tc2	0.001867906	1.793392062	down
LOC100047264	0.001869478	1.626560092	up
Zc3h18	0.001872035	1.691239238	up
Rab32	0.001873077	1.738201737	up
Laptm4a	0.001875477	1.861602426	down
Apobec1	0.001877209	2.472486019	up
C87860	0.001878563	1.643683076	up
Pole3	0.001878827	1.687413931	down
6530415P06Rik	0.001878876	1.596873164	up
LOC383099	0.001880546	1.546484113	up
A830033B12Rik	0.001881454	1.523454428	up
Ap1g1	0.001881454	1.656102896	down
Tex2	0.001882752	1.597836733	up
Slc4a3	0.001883361	1.932770133	up
Alkbh7	0.001885851	1.698295236	up
LOC100044566	0.001885851	1.555592299	up
LOC100043257	0.001886637	6.89162159	down
Rasgrp3	0.001886637	1.777761817	up
Spg7	0.001892713	1.784437299	up
Siat7b	0.0018955	1.969845414	up
A030011F13Rik	0.001895677	2.390878916	up
Col9a3	0.001897846	1.575743914	down
A130052C08Rik	0.001900251	1.804756999	up
Pcm1	0.001900251	2.782887936	up
1300010F03Rik	0.00190052	1.801385999	up
Lyl1	0.001901323	2.016277552	up
Ppbp	0.001901323	1.834048271	up
Rgp1	0.001903786	2.53849411	up
Mtmr7	0.001906075	1.851805091	up
Pdk2	0.001906501	1.658661246	up
Icosl	0.00190684	2.403559446	up
BC030336	0.001908229	1.722659946	down
Mtap7d1	0.001908229	1.89144969	up
Nfkbil2	0.001912505	1.967721105	up
A430104N18Rik	0.001912525	1.602636814	up
2610524H06Rik	0.001912558	2.231719017	down

Vkorc1l1	0.001912565	1.789702892	down
Ulk2	0.001915245	1.989941478	down
Ppic	0.00191558	2.057356834	down
Tm7sf2	0.00191558	1.531668782	up
Rps24	0.001915694	1.870081425	down
Tuba3a	0.001919	2.150995016	up
Trim62	0.001922617	2.022188902	up
Fes	0.001927638	2.007770538	up
Fgd2	0.001927638	2.169153452	up
Smg5	0.001927959	1.648031116	up
Plekhg2	0.001931971	1.521012306	up
LOC100047323	0.001934442	1.564939857	down
LOC636687	0.001935392	2.509327173	up
Cacna1h	0.001938712	1.579277396	up
BC046331	0.001940191	1.535637379	down
Cspp1	0.001940311	1.543517828	up
LOC100047052	0.001940588	1.683980346	down
Scoc	0.001940588	1.64788878	up
Frap1	0.001941006	1.817364335	up
Cpt1c	0.001942442	1.715048313	up
Snw1	0.001942691	1.60593462	up
4930553M18Rik	0.001943397	1.963382483	down
Emd	0.001943397	2.590956211	down
Ing2	0.001943709	1.628379822	up
Ppp1r13b	0.001944209	1.653621316	up
Hel308	0.001945112	1.555132389	down
Pcdh7	0.001945707	1.514340878	up
9330132O05Rik	0.001956617	1.527526259	up
Rad51ap1	0.001956617	1.817406774	down
Bre	0.00195725	1.52516079	up
Itga11	0.00195725	1.52748549	down
Rspo2	0.00196096	1.641341209	up
Usf2	0.00196096	1.963961601	down
Spcs2	0.001962723	1.929940462	up
Eif2s1	0.001963971	1.641513586	down
Dnaja2	0.001964087	2.192937136	up
2310007D09Rik	0.001965489	1.524571419	down
Dock7	0.00196553	1.502476454	down
Tmco1	0.001969233	1.563940764	down
Tnfaip8	0.001973097	1.601357222	down
Nup37	0.001979683	1.984605551	down
2610020H08Rik	0.001981164	1.668486714	up
Pex7	0.00198469	1.907020807	down
Cblb	0.001985817	2.736266136	up
Qtrtd1	0.001986937	2.600658417	down
Gtf2a1	0.001988764	1.520989418	down
Mcm6	0.0019918	1.965644717	down
Fam120a	0.001993029	3.189107657	down
LOC633360	0.001994324	1.542422175	up
Nrf1	0.001994324	1.500810623	up
scl0003155.1_68	0.002003255	1.726139426	up
Eif3s3	0.002010128	3.195585966	down

Foxf2	0.002010128	2.093223333	up
Mtf2	0.002010128	1.923413634	down
4833439L19Rik	0.002012783	2.039529562	down
Znrd1	0.002026417	1.685640454	down
LOC677317	0.002029461	1.87910068	down
5830461H18Rik	0.002029988	1.510795474	up
Cd200	0.002030281	1.843048215	down
4933415A04Rik	0.002032043	1.667817354	up
Usp28	0.002035776	2.089029074	up
2310036O22Rik	0.00203688	1.892043948	up
Sfrp2	0.00203688	2.048348904	down
Thnsl2	0.002038064	1.551214695	up
Agpat6	0.002039883	1.585151076	up
1200003I07Rik	0.002040671	1.990882516	up
Glod4	0.002043503	1.803373098	up
Rab13	0.002050773	1.627658963	down
Cux1	0.002050937	1.542415023	up
D630016P04Rik	0.002050937	1.516773701	up
Prickle3	0.002050967	1.527388811	up
Atg5	0.002055903	1.744779706	down
9330151E04Rik	0.002057609	1.65166688	up
Pabpc1	0.002057609	2.18592906	down
LOC677248	0.002058981	1.753391981	down
Dbc1	0.002059791	1.534218311	up
1190007I07Rik	0.002060189	1.608626485	up
Hhex	0.002062186	1.857045531	up
scl0002194.1_2265	0.002071402	1.694530249	up
Afap1	0.00207586	2.008570194	up
Mapk12	0.002077594	1.643838286	up
Fchsd2	0.002079711	1.642267704	down
Grik5	0.00208099	2.054666758	up
Thra	0.00208475	1.508067846	up
Wdr23	0.002086002	2.06183219	up
Gpr65	0.002094452	2.370083809	up
Lphn1	0.002094672	1.962758541	up
Synj2bp	0.002097165	2.245429277	down
LOC100043703	0.002103693	2.17633009	up
Egfl7	0.002111255	1.795167565	up
Stard7	0.002111255	2.118120194	down
Cd109	0.002116086	1.708335042	down
Nmu	0.002116086	2.020240068	down
LOC100041585	0.002117464	1.855309486	up
Napb	0.002120789	1.901234865	up
Klf8	0.002123433	1.52449286	up
9430098F02Rik	0.002125027	1.514325261	up
Smtn	0.002125167	1.901681423	up
H2-K1	0.002125636	1.562729001	up
Olfr1513	0.002131261	1.558538675	down
1810073N04Rik	0.002131706	1.694420219	up
Clec16a	0.002132251	1.86718154	up
Cdkn2aip	0.002137565	1.746379733	up
Hook1	0.002137565	2.538444281	down

Mrg1	0.002137565	1.852821708	up
4632415L05Rik	0.002139082	1.767496824	up
Itgb5	0.00213987	1.791575909	up
Glul	0.00214331	2.251183271	up
Eya1	0.002144204	1.914695263	down
Tlcd2	0.002146171	1.564034581	down
6330409N04Rik	0.002146994	1.826803684	down
Ddx6	0.00214763	3.527591467	up
2410018M08Rik	0.002147717	1.512026787	up
9030203C11Rik	0.002147717	1.53328979	up
A230106D06Rik	0.002147717	1.654462457	up
Dpysl2	0.002147717	1.98444128	up
Dennd2a	0.002157983	1.606284261	up
Ercc4	0.002158399	1.557668924	up
Cpne3	0.002158849	1.571391106	down
D930015E06Rik	0.00216051	1.57091701	up
Snx4	0.00216051	1.918561816	up
2510049I19Rik	0.002166731	1.586477399	up
9030607L17Rik	0.002166731	1.525445223	down
1700037H04Rik	0.002167488	1.740111709	up
LOC100044008	0.002171397	1.53608489	down
scl0002215.1_2	0.002171397	1.878153324	down
Cul1	0.002172185	1.637100816	up
Ncbp2	0.002172185	2.439580679	down
Slc35f1	0.002172185	2.190327406	down
B930008G09Rik	0.002178293	1.690303683	up
Map2k5	0.00218253	1.849451542	up
Ttc15	0.002184878	1.531815052	up
Taf13	0.002185647	1.507123232	up
Gmfg	0.002188983	2.311543703	up
Ormdl3	0.002189454	1.710891962	up
Synpo	0.002195345	1.866822839	down
Tmem45a	0.002196654	4.09446907	down
LOC244710	0.002196939	1.854791403	up
0610007P08Rik	0.002201536	1.747234583	down
1110012D08Rik	0.002201536	1.561735511	up
Larp4	0.00220346	2.362384558	down
A330049M08Rik	0.002205816	2.046452045	down
Appbp2	0.00220862	1.773905993	down
Galnt14	0.002208981	1.679883838	up
Raet1c	0.002215961	2.150902033	down
Hist1h3h	0.002216293	1.736517668	down
Sep-09	0.002218351	2.099341631	up
LOC100048616	0.00222248	1.53417325	up
Klhl24	0.00222349	1.923350453	down
EG384179	0.002223998	1.954072833	up
Cdc5l	0.002229116	1.562691808	down
LOC100044160	0.002229726	2.071153402	down
Surf1	0.002235074	1.572135568	down
5630401D24Rik	0.002235957	1.859439254	down
Mier2	0.002243793	1.528760314	down
Mpp2	0.002244097	1.561196923	up

Gng4	0.002244691	1.594417691	up
Nbea	0.002247198	1.603463292	down
Gpbp1l1	0.002249725	2.00600338	down
Nfe2l2	0.002250218	2.164215803	down
Ddx58	0.002253005	1.632380843	down
Flt1	0.002254746	2.435525656	up
scl000260.1_64	0.002254746	1.689574122	up
Rnf31	0.002257364	1.935285211	up
Rac2	0.002257895	1.805455566	up
Msx1	0.002262195	1.988319874	up
Psmc6	0.002262195	2.036067009	up
Suv420h1	0.002262195	1.513921738	down
Psmc5	0.002267337	1.573473096	up
1500041N16Rik	0.002276686	1.863206625	up
S100a1	0.002282376	1.660141587	down
1700023F06Rik	0.002282755	1.644182205	up
Ipmk	0.002285496	1.674729943	down
Ebi3	0.002286487	1.833338022	up
Lip1	0.002286487	2.74720335	up
Sin3a	0.002287821	1.715649724	up
LOC100047358	0.002291952	1.578546643	up
Vdac3	0.002292387	2.138645887	up
Dnaja2	0.0022962	1.603211403	up
Slitrk6	0.002297598	1.623122454	down
Apoc1	0.002302005	2.393418551	up
Sorbs2	0.002308401	1.909857392	down
Ube2a	0.002309647	1.672432542	down
D430034A07Rik	0.002310419	3.2743752	up
Ube2q1	0.00231388	1.58532691	down
C530028I08Rik	0.002320393	1.539510965	down
Setd6	0.002320393	1.965571165	up
Palld	0.002321384	2.444493532	up
Ndufa10	0.00232256	1.53215611	up
Foxj3	0.002331779	1.773408651	down
Anp32e	0.002335056	2.025931835	down
Mbnl2	0.002335056	1.756134391	down
2810410M20Rik	0.002335154	1.631465673	down
Asb7	0.002336466	1.655183196	down
Zcchc6	0.002336466	2.124472618	up
Cd68	0.002339354	2.921685934	up
Hsf2	0.002339354	1.642094493	up
BC053749	0.002340072	1.623446345	up
Lsm1	0.002341598	1.744793415	up
LOC100045315	0.00234549	2.115972996	up
Tjp1	0.002347473	1.73206842	up
BC100530	0.002353143	1.717787862	up
Kifc1	0.002353143	1.527059317	down
Oip5	0.002358764	1.800255418	down
LOC215098	0.002365091	1.655896902	up
Iffo1	0.00237136	1.659404159	up
Yipf3	0.002376053	1.588621855	down
Plcb3	0.002383994	1.559627295	up

Gabpb1	0.002384795	2.433291197	down
Ids	0.002390439	1.509627938	down
Prei4	0.002401591	1.993774176	down
Garnl1	0.002402388	2.193291664	up
Lysmd2	0.002402877	1.52040875	down
Fzd4	0.002404447	2.080604315	down
Anxa6	0.002406488	1.643139124	up
A530025E09Rik	0.002407322	2.522298098	up
Fbf1	0.002407322	2.009415865	up
Adat1	0.002407497	1.746153474	up
Ptn	0.002407497	1.975698113	down
Sars	0.00241001	1.554883003	up
Irx3	0.002411333	1.500570893	down
Pnkp	0.002411333	1.556210995	up
Atrn	0.002417642	1.609615684	down
EG434077	0.002417642	1.663578391	up
4932417H02Rik	0.00242601	1.55621779	up
2810474O19Rik	0.002431129	1.64081192	up
Kif21a	0.002432398	2.268258572	down
Cyr61	0.002435529	3.337931395	down
C130020P16Rik	0.002435677	1.939721704	up
Tgoln1	0.002437156	1.883389592	down
Dorz1	0.00243943	1.6090765	up
A930002F06Rik	0.00243982	1.862867117	up
Ptprf	0.00244168	1.939987421	down
E430021A19Rik	0.002447014	1.878671646	down
Mynn	0.002452383	1.664409757	down
Pacs1	0.002460053	1.611176252	up
Lrrc1	0.002462651	1.585395694	down
Scamp3	0.002462962	1.615253449	up
Zfp7	0.002473105	1.537599683	up
LOC218696	0.002476638	4.27033329	down
Cryba4	0.002480959	2.113610029	up
9626984_149_rc	0.002486456	3.164788723	up
Bat2	0.002486974	2.895119667	up
LOC100048413	0.002488584	1.756552696	down
Mr1	0.002488584	1.554100752	up
Birc5	0.002491882	2.299828529	down
D030049N18Rik	0.0024983	1.760187149	up
A830007N09Rik	0.002499007	1.578349233	down
2010106G01Rik	0.002499791	2.39048624	down
scl0001248.1_219	0.00250389	1.583583832	up
Tssc4	0.002510635	1.620042682	down
Top3a	0.002512422	1.544467807	up
Brp44	0.002512526	1.769845605	down
Copz1	0.002512526	1.980276108	up
Pds5a	0.002512719	1.875946164	down
Pcbp1	0.002517761	2.539755344	down
Zubr1	0.002517834	1.615913987	down
Ralgds	0.002520801	1.746110797	down
4921533J23Rik	0.002524715	1.653480768	down
Tomm7	0.002532667	1.536461711	down

Dhx36	0.00253662	1.817358971	down
Zfp90	0.002537745	1.724294186	up
scl0003955.1_3	0.002538818	1.924738407	down
Xylt2	0.002538818	1.902259708	up
1700030K09Rik	0.002541271	1.665150762	up
Mfi2	0.002541271	2.146121979	down
Eif3b	0.002542288	1.67359066	down
Socs4	0.002542288	1.771161676	down
Rere	0.002542503	1.924082041	up
A130028H19Rik	0.002544112	2.485955238	up
LOC100048873	0.002544112	1.638649702	down
2210408E11Rik	0.002546777	2.306398869	up
Tanc1	0.00254755	1.740174651	up
Cep78	0.002549191	1.997898817	down
Rgs12	0.002549191	1.830780387	up
Zfp238	0.002551324	2.892208099	down
E430033D06Rik	0.002558084	1.992217302	down
Il13ra1	0.002558718	1.503205299	down
Dnajb9	0.002566281	2.115036011	up
Rab8a	0.002570099	1.825872421	up
Arhgef19	0.002578699	1.560842991	down
Fgfr2	0.002580701	1.686376214	down
E330023A07Rik	0.002581904	1.624966502	up
2610040L17Rik	0.002586816	1.903311849	up
Alkbh2	0.002589673	1.567934513	up
Chd6	0.002602232	1.612116575	up
Aqr	0.00260475	1.522784352	up
A830081I03Rik	0.002611067	1.919531226	up
Cpeb2	0.00261253	2.725927591	up
B930030B22Rik	0.002617911	1.634834886	up
Cebpa	0.002617911	1.874682903	up
Tacr1	0.002622459	1.609485507	up
Cd24a	0.00262282	1.825586438	up
2510012J08Rik	0.002623608	1.865270853	up
Emp1	0.002623997	1.872230411	down
2010110K16Rik	0.002626244	1.875836492	up
Wnt11	0.002628996	1.79390502	up
Sox4	0.00264353	2.331992149	up
Hmox1	0.002646113	4.230695725	up
Mafb	0.002647133	1.670907617	up
Ncoa6ip	0.002648315	2.249891758	down
Fads2	0.002649836	1.512167692	down
Banf1	0.002650066	1.812149763	down
Pla2g7	0.002650066	2.027091265	up
0610025P10Rik	0.002663985	1.50643301	up
LOC667519	0.002663985	1.549097538	up
2810035J02Rik	0.002668471	1.548156023	down
LOC382461	0.002672095	1.692812443	up
4632404B13Rik	0.002676508	1.557106257	up
Ndufa4	0.002681188	1.763020635	down
Capg	0.002692421	2.358724833	up
Nploc4	0.002695672	1.832088232	up

Dnajc12	0.002699814	1.560367584	down
Ttyh3	0.002708223	1.897138476	up
Arv1	0.002717935	1.637090802	up
Tor1aip2	0.00272261	1.568573952	down
Mark1	0.002725736	2.202377558	down
C130085D15Rik	0.00272787	3.126176596	up
3110005L21Rik	0.002730418	1.762537122	up
Mgea5	0.002737651	2.561894417	up
Polb	0.00274035	2.14051652	up
Sfpi1	0.00274035	1.619255185	up
Ptgfrn	0.00274057	1.626416445	up
Slc44a1	0.002740736	1.844552755	down
Rab43	0.002746571	1.902474165	up
2310047K21Rik	0.002749248	1.717386007	down
Rad51ap1	0.002753431	1.511897922	down
Tirap	0.002754319	1.974227786	up
5430439M09Rik	0.002754585	1.632367969	up
Atp5j	0.002763231	1.566173673	up
LOC100044294	0.002763231	1.602988362	up
Gripap1	0.002763337	1.705757141	up
Foxj3	0.002766586	1.661810517	down
3110001N23Rik	0.002771197	2.28801322	up
C430046P22Rik	0.002774326	1.611743212	up
Rnf11	0.00277471	1.990785837	down
scl0002702.1_3805	0.00277471	2.788537741	up
Serping1	0.00277471	1.609072208	up
Lonrf1	0.00277823	2.00988245	down
2510002P07Rik	0.002780714	1.605831385	up
Ube2h	0.002780714	1.870797277	up
Zc3h13	0.002780714	2.074283838	down
Ctsz	0.002787254	2.486199141	up
Btbd4	0.002790525	1.588028908	up
Ss18	0.002808195	1.854062438	down
D230047F17Rik	0.002809201	1.766026139	up
Swap70	0.0028093	1.667887688	down
Tmem161b	0.002810037	1.719977498	up
Ecm1	0.002811815	1.867858887	up
D430007A19Rik	0.002814274	2.307518244	up
Ywhae	0.002820566	1.634682298	up
Amotl2	0.002824668	1.523490906	down
Pcdh10	0.002824668	1.62713182	up
Mepce	0.002825152	2.317586184	up
LOC100048733	0.002828371	1.577328682	down
Med25	0.002828691	1.673347831	down
1110019N10Rik	0.002829903	1.651130676	down
A430024B14Rik	0.002833771	1.698749542	up
5630401D24Rik	0.002835221	1.60729599	down
2810031B20Rik	0.002837298	1.691209316	down
C130092F19Rik	0.002838582	1.676192164	up
Zfp184	0.002842752	1.631226897	up
Stk32a	0.002842809	1.641725421	down
Fbxo6	0.002843662	1.553890586	up

Mapre2	0.002843662	1.639342785	up
E130007O11Rik	0.00284629	1.509031296	down
6720420G18Rik	0.002850026	1.712918878	up
Eif3b	0.002854191	1.806540251	down
Atpaf2	0.002855776	1.50365603	up
Tmem18	0.00285628	1.606968403	up
2610524F24Rik	0.002856288	1.626529932	down
C230064E07Rik	0.002857576	1.695591331	up
P2rx4	0.002862276	1.588501096	down
Lime1	0.002862613	2.06583643	up
E130010O04Rik	0.002871746	1.539424777	up
Coro1c	0.002875337	1.947839379	up
8430403M15Rik	0.002878809	1.831468344	down
Cd79b	0.002887198	1.883948922	up
Arfip2	0.00290119	1.551674247	down
Atp2c1	0.002903612	1.642340541	down
Fbln1	0.002903612	2.065477371	up
Rnf25	0.00290968	1.781982064	up
Gnb2	0.002914946	1.623925328	up
Jmy	0.002924739	1.592119217	down
Zbtb43	0.002925053	1.658994556	up
Pip4k2b	0.002929294	2.198315144	up
1500010G04Rik	0.002934441	2.523571014	up
Itgb3bp	0.002934441	2.053759575	down
Lpxn	0.002936035	1.77714169	up
4732423C17Rik	0.002945351	1.73161602	down
Rbm6	0.002948351	2.289750576	down
Kcnn1	0.002959336	1.893638134	up
LOC100043402	0.002959336	3.02442193	up
Prpf40b	0.002964168	1.827224493	up
Rab22a	0.002964169	1.512351275	up
Sec61b	0.002964169	1.852417946	down
Rasa1	0.002966602	1.986502409	down
Papd4	0.002972011	1.622642875	down
Slc8a1	0.002972388	1.83786571	up
LOC331511	0.002984675	1.938384652	up
Man2b1	0.002985806	1.862970471	up
Glrb	0.00299762	1.528977275	down
Commd6	0.002998698	1.511464834	down
Nfam1	0.002999326	2.417654753	up
Pou2f1	0.002999326	2.454018354	up
Stk18	0.002999326	1.515773654	up
Hira	0.003000919	1.590050817	down
Map3k4	0.003000919	2.181895018	up
Nup160	0.003000919	2.067004204	down
E130319N12Rik	0.003009267	1.858166337	down
A430110M15Rik	0.003010085	2.050303698	up
Pard6a	0.003011633	1.696772218	down
Psmb3	0.003013288	1.576965928	down
Cetn2	0.003029562	2.201985121	down
Plekhm1	0.003043939	2.258257389	up
Schip1	0.003047182	2.145208836	down

LOC268602	0.003047623	1.63210392	down
Hist1h3d	0.00305236	1.663193345	down
5930422O12Rik	0.003059425	1.745453954	up
Sdhc	0.003059425	1.559278846	up
EG434401	0.003060069	1.665538073	up
2310047M15Rik	0.003065793	1.522659063	up
9130213B05Rik	0.003068462	1.736384034	up
E330018D03Rik	0.003076962	1.631248593	up
Tsc22d4	0.003081192	1.689806819	up
LOC100044170	0.003082999	2.01610589	down
Rbm19	0.003083176	1.841170669	up
Mfsd7a	0.003091416	1.598809719	up
Slc39a8	0.003093493	1.50484395	down
2410001C21Rik	0.003093862	1.814378738	up
Ascc3	0.003098829	1.935636044	down
LOC100046825	0.003102969	1.992267132	up
Serinc4	0.003104531	1.765221	up
Ndufa8	0.003104565	1.663775682	down
2900010M23Rik	0.00310725	1.78678298	down
3202002H23Rik	0.003109176	2.037961483	down
Rab5c	0.003109176	1.578192949	up
Rhoa	0.0031095	1.559029698	up
Tasp1	0.003111075	1.752894998	down
C230094A19Rik	0.003117175	1.979426384	down
Igsf6	0.003120444	1.535048485	up
Agpat2	0.003122466	1.915247798	down
Asf1b	0.003125316	1.900204778	down
Sssca1	0.00312981	1.511388063	up
A530064K17Rik	0.003129953	1.564487696	up
LOC668837	0.003135693	2.497492075	up
LOC674195	0.003135693	1.66988349	up
Lin7a	0.003142472	1.767274857	down
Snrk	0.00314481	1.829528213	down
LOC100038993	0.003145736	2.192788839	down
Ccnd1	0.003149318	1.749664187	down
0610009O20Rik	0.003151571	1.54002142	up
Gadd45gip1	0.003151571	1.674926162	up
B230312A22Rik	0.003156103	1.860005021	up
LOC665386	0.003156103	2.90452981	down
3300002A11Rik	0.003159965	1.582831383	up
Pou3f3	0.003161661	1.736479521	down
Clcn3	0.003162251	1.793439388	up
Tssc1	0.003166017	1.825648665	down
1200009F10Rik	0.003168092	1.541766286	up
Gpr89	0.00317024	1.547614813	up
Th1l	0.003172688	1.537360549	up
LOC100045737	0.003174888	1.721607089	down
Pigp	0.003176411	1.88066709	down
Anapc2	0.00317806	1.506613612	up
Phrf1	0.00317806	1.806850314	up
Tgm2	0.003179314	1.988940001	down
LOC100044059	0.003189047	1.543218732	down

Slc35a3	0.003195917	1.9222188	down
2700069I18Rik	0.003204139	2.096599817	down
Cnot10	0.003204139	1.703911781	up
E130112E08Rik	0.003209221	2.25476861	up
Igfbp4	0.003218223	1.883373022	up
LOC381808	0.003220218	1.581240773	down
Nup62	0.0032256	1.546081662	up
Sec61b	0.003229256	1.893554211	down
Map2k5	0.003229405	1.592213869	up
Inpp5k	0.003233563	1.736221194	up
A630042L21Rik	0.003240851	1.652383089	up
Sqrdl	0.003242426	1.655393481	up
D16Bwg1543e	0.003246188	1.532209992	up
Mobk1b	0.003246188	1.725683808	down
Galnt11	0.003246403	1.576024771	down
Nudcd1	0.003246403	1.789779425	up
Taf7l	0.003246403	1.70212853	up
Galnt1	0.003247299	1.853106022	down
Lphn3	0.003251161	1.831612229	down
Zfp13	0.003255191	1.69451499	up
Tyk2	0.003257957	1.530283332	up
Ccnc	0.003260711	1.893324256	down
Pkn3	0.003262583	1.941288948	up
D130076G13Rik	0.003275933	1.599083543	up
Sdf2l1	0.003283825	1.867284298	down
Nssr	0.003286162	2.351471186	up
scl000032.1_60_REVCOMP	0.003295364	1.78570807	down
Faim	0.003298329	2.021032572	down
Fam171a2	0.003298804	1.624395967	up
Tlr7	0.003299046	2.234384537	up
A230056P14Rik	0.003308681	1.557416081	down
Pramel4	0.00330894	1.828261614	up
Spata5l1	0.003309147	2.046791792	down
Ccnc	0.00331095	2.818184137	down
Zfp692	0.003317443	1.805917025	up
Traf4	0.003317867	1.84571135	down
Atp6v1a	0.003325462	1.781631231	down
Samhd1	0.003332507	1.643926978	up
Fancc	0.00333276	1.544645429	up
Gli2	0.003332995	1.640829682	down
Kras	0.003338463	1.503148794	up
Rabif	0.003357563	1.59619689	down
Tmem85	0.003357563	1.539951801	down
Homer1	0.003358704	1.683333278	down
9430015G10Rik	0.003360189	1.584702492	down
LOC382972	0.003360856	1.65523243	up
6720427H10Rik	0.00336579	2.302752972	up
Atl3	0.003377152	1.884488821	down
Mrps34	0.003378016	1.93731761	up
Hmgcs2	0.003378565	1.805868745	down
Flcn	0.003381534	1.822847724	down
4833408D11Rik	0.003387055	1.761781454	up

Ctage5	0.003387557	2.645906925	down
G6pc3	0.003387557	1.757308245	down
Top2a	0.003388263	1.654109836	up
2310075M15Rik	0.003395082	1.735389829	up
Pcmtd1	0.003398648	1.791262388	down
Mbtps2	0.003399971	1.629961491	down
Sft2d3	0.003402125	1.729820132	up
Als2cr13	0.0034025	2.424989462	down
B230208H17Rik	0.003403729	1.922761679	up
Slc2a8	0.003404644	1.66996932	up
scl000683.1_16	0.003409373	1.674127221	down
Rffl	0.003423323	1.611961484	down
Tuba1a	0.003432405	2.484294176	up
6430706H07Rik	0.003439797	1.631825447	up
Gmfb	0.003448722	1.635286093	up
Gas2	0.003450006	2.430493116	up
Efnb2	0.003451626	2.006764174	up
Mylc2b	0.003451626	2.431245089	down
Cbln1	0.003453856	2.384750605	up
E430025E21Rik	0.003470202	1.801285029	up
Chchd3	0.003471229	1.601028681	up
Fras1	0.003471311	2.030468226	up
Arhgap18	0.003473257	1.810097933	up
EG625514	0.003473298	1.849829316	up
Hmmr	0.003481232	1.599424243	down
Crk	0.003492635	1.565510154	up
Dkk3	0.003493833	1.85215342	down
LOC100047273	0.003507102	1.582581997	up
Chkb	0.003508322	1.602087975	up
Phka1	0.003508322	1.814004064	down
Mrps31	0.003514801	1.877963662	down
Adam33	0.003516111	1.6104846	up
Trappc6b	0.003516111	2.610994101	down
D5Ertd689e	0.003519163	2.334233284	up
B230326O19Rik	0.003522195	1.611258864	up
BC008163	0.003524642	1.663258314	down
Gls	0.003533307	3.115065575	down
LOC100042074	0.003534356	1.642328501	up
Usp46	0.003535178	2.19336009	down
1810009A15Rik	0.003536369	1.642547131	down
C030048B08Rik	0.003544892	1.677407146	down
Matk	0.003546746	1.506923318	up
Syncrip	0.003556234	1.894646764	down
A730069N07Rik	0.003561068	2.787984133	up
Rdm1	0.003565355	1.704985976	up
2600010L24Rik	0.003571567	3.224089861	up
Ehmt1	0.003572499	1.750568152	down
Arhgap20	0.003595054	1.637039781	down
C230058N13Rik	0.00360525	1.530951381	down
Pms1	0.003605627	1.542927265	up
Scap	0.003607507	1.572818518	up
Gadd45a	0.003609364	1.737415195	down

Idb2	0.003614169	2.314078808	down
D230014K01Rik	0.003616988	1.726168275	up
LOC100047419	0.003616988	1.821757913	up
Ahcyl2	0.003619385	1.82755959	down
1810056I18Rik	0.00361984	1.657168865	up
Ppp4r1	0.003621267	2.129499674	down
Pomt2	0.003625459	1.540677428	up
Ube3a	0.003626595	1.832949758	down
0610009J05Rik	0.003626915	2.230975151	up
AF251705	0.003628265	1.610610485	up
Rtkn	0.003628265	1.949686885	down
Rarg	0.003631985	1.786343336	down
Stim1	0.003635292	1.953275561	up
5133401N09Rik	0.003642849	1.737028003	up
B230315N10Rik	0.003642966	1.694989801	up
Polr2d	0.0036439	1.926323533	down
Slu7	0.003655493	1.624868631	down
Spata7	0.003658188	1.78457284	down
Eif2c2	0.003668853	2.184803963	up
Armc7	0.003670684	1.605438232	up
Tbc1d9	0.003670884	1.64367342	down
Arid4b	0.003670922	1.777226567	up
Dock10	0.00367329	1.666438103	down
5730414M22Rik	0.00367604	1.664072633	up
Osr1	0.003682738	2.1889112	up
Cnot4	0.003683566	1.604010463	up
5830472F04Rik	0.003684557	1.51057303	up
Trappc2l	0.003691504	1.767647147	up
Pla2g4b	0.003701275	1.545077562	up
Ssh3	0.003703141	1.593223453	up
Dus2l	0.00371027	1.524039984	up
LOC632454	0.003716338	2.542020559	down
Scrn1	0.003717026	1.52770114	down
Was	0.003717987	3.320369482	up
Lmbr1l	0.003720194	1.618604183	up
Spag5	0.003724968	1.507095933	down
Tlr13	0.00372603	1.988589168	up
Rpa2	0.003730835	2.399958134	down
BC023892	0.003735123	2.652648926	down
Prdx2	0.003738394	1.878647685	up
Scly	0.003738394	1.559446216	up
C77604	0.003739046	1.888645768	up
Col25a1	0.003739326	1.931287408	down
Col8a1	0.00373976	2.177601099	up
LOC100044766	0.003741058	1.541253686	down
Cad	0.003742848	1.964092374	up
ENSMUSG00000043795	0.003742848	2.344728231	up
2610019N19Rik	0.003743986	2.018415451	up
Dync1i2	0.003748593	2.300271273	down
0610025P10Rik	0.003752044	2.05112958	up
Gp49a	0.003752044	1.830824614	up
Prps2	0.003754008	1.518619895	up

Six2	0.003772785	2.700210095	down
Lancl1	0.003775121	1.873844385	up
Camk2d	0.003790436	1.692703247	down
Wdfy2	0.003791419	2.05040741	up
3230401L03Rik	0.003792494	2.112091541	up
Sumf2	0.003805012	1.765727282	down
4931406C07Rik	0.003810988	1.631590962	up
Baz1a	0.003810988	1.765205741	down
Gdap9	0.003817382	1.719667316	up
Slc25a4	0.003818135	2.12780714	up
Mknk1	0.003822201	1.511101246	up
Gin1	0.003823536	1.599875689	down
Gnaq	0.003823536	1.843773246	down
Adam12	0.003837272	1.832150936	down
Sephs2	0.003840852	1.651383042	down
Wbscr22	0.003841751	1.866551638	down
Nde1	0.003845731	1.664103627	down
Mtap7d2	0.003850864	1.639999032	down
G630024G08Rik	0.003857253	1.723655582	up
Scamp1	0.003857253	1.853427053	down
Cdyl	0.003861351	2.133082867	down
Rab38	0.003870166	1.534332275	down
B930053N05Rik	0.003880965	1.890425682	up
Lmtk2	0.003883419	1.658643127	up
Ftsj3	0.003884982	1.698146701	up
Tnrc6a	0.003884982	1.597840071	up
Nedl2	0.003889202	1.502337217	up
scl0002975.1_346	0.003889489	1.894325256	up
Cdadc1	0.003889907	1.581121564	down
Syde2	0.003889907	1.605773211	down
E330033B04Rik	0.003895376	1.597389221	up
A630018P17Rik	0.003897273	1.58425498	down
Top1mt	0.003897288	1.543007493	up
C030044O21Rik	0.003897868	1.944759607	up
Nufip2	0.003900053	1.756588578	up
Fcgr1	0.00391657	1.774687767	up
Cacng7	0.003917256	1.570699692	down
9030417F11Rik	0.003921093	1.957041144	up
Gsk3b	0.003928651	2.003126621	up
Atp2a3	0.003937052	2.465842247	up
Rac3	0.003939823	1.596728802	down
Myh9	0.003945643	1.610285163	up
Zfp709	0.003953994	1.969297528	up
2210010B09Rik	0.003959118	1.505251527	down
4930500L23Rik	0.003966129	1.663146734	up
Tmem14c	0.003966129	2.00219965	down
Mt1	0.003977035	3.477302551	up
Cog2	0.003977235	1.681783676	up
Lims1	0.003984598	2.157703638	up
2700081O15Rik	0.003993027	1.916437268	up
9430085L16Rik	0.003993027	2.265917778	up
LOC385917	0.003993027	1.545158267	up

Phf2	0.003993027	1.750092745	up
Cyb5b	0.003997692	1.975117445	up
Kif26b	0.004011465	1.922086239	down
Ppp3r1	0.004016906	1.757629156	up
LOC245676	0.004021084	1.651540637	up
Nrp2	0.004025776	1.683444738	down
Gimap6	0.004028302	1.717622161	up
2010005J08Rik	0.004041011	1.626974702	up
Tk2	0.004043707	1.934167385	up
Olfml3	0.004054398	1.811375737	up
scl0001912.1_11	0.004054634	2.468482018	down
Hint1	0.004056152	1.908129334	down
Tpd52l1	0.004058182	2.081928015	down
Capn7	0.004077544	2.686601162	down
Cog6	0.004077859	1.77967155	down
8030462N17Rik	0.004082752	1.581686139	down
Sgtb	0.004083979	1.82006669	down
1110025M09Rik	0.004086502	1.628869534	up
Tnmd	0.004092014	2.280372143	down
LOC100047339	0.004092972	1.777156591	up
Pcdha7	0.004092972	1.730584621	up
Car3	0.0040933	1.769499302	down
Akap8	0.00409555	2.021672249	up
Gna15	0.00409555	1.639328718	up
E330027P06Rik	0.004101725	1.536995292	up
6330419J24Rik	0.004101737	1.510502219	down
6530406A20Rik	0.004101737	1.60353291	up
LOC100046608	0.004104266	1.772329927	down
LOC100048436	0.004104764	1.846209168	down
Gata2	0.004106316	2.113579989	down
Etv5	0.004108627	1.707441449	down
1700074L02Rik	0.004112186	1.500760675	up
Cdk2	0.004112186	1.78936398	down
Arih1	0.004125446	2.044676065	up
Trpc2	0.004131077	1.676445723	up
Dnajc15	0.004141653	1.558027268	up
E130303B06Rik	0.004141653	1.669071436	up
Grlf1	0.004148846	1.78775537	up
Prdm4	0.004149471	1.87024951	down
A530020A01Rik	0.004153241	1.565151215	up
Gpiap1	0.004153773	1.73138237	up
LOC329506	0.004161408	1.698061347	down
Dgkz	0.004162489	1.824741364	up
Hook3	0.004164313	2.14341569	up
Ccdc19	0.004167607	1.656460881	up
E030049G20Rik	0.004169922	1.982677817	down
EG633570	0.004177476	1.793519974	up
Rtn3	0.004177476	1.636004448	down
Zxdc	0.004179012	1.786723375	down
Fbxl20	0.004180364	1.65792346	up
Wnt11	0.004180364	1.609444022	up
Eml3	0.004191109	1.538709998	up

Fuz	0.004195858	1.568669081	down
Mkrn1	0.004195963	2.301561832	up
Gstm7	0.004196266	2.020983934	down
Ino80	0.004196266	1.660962105	up
Sema4g	0.004196266	2.031868696	up
Clock	0.004198104	1.949470282	down
Lrch1	0.004199246	1.604456425	down
Med7	0.004224315	1.720327735	down
LOC671523	0.004225403	1.978464365	up
Nono	0.004240745	2.056122541	up
Srrm1	0.004250319	1.615817308	up
Gm2a	0.004263536	1.636600494	up
Glipr1	0.004264311	2.391495943	up
Lrp1b	0.004283449	1.663841486	up
LOC381546	0.00428382	1.672085404	up
2810409M01Rik	0.004286827	1.734881401	up
Isoc1	0.004294823	1.630795598	down
EG245297	0.004297348	2.63060832	down
Kcnab2	0.00429767	1.53936851	up
LOC100039514	0.004303507	1.510446429	up
Fv1	0.004326617	1.651861072	down
Prickle1	0.004326617	3.681575537	down
Supt6h	0.004326617	1.528852344	up
D330008I21Rik	0.004336954	1.646365404	up
Ccdc12	0.004345907	1.749685407	up
Bxdc5	0.004347988	1.857530832	down
Elovl6	0.004349256	1.853826523	up
Slc14a1	0.004349256	1.719521284	down
Tnnt1	0.004349305	1.736913204	up
Ankrd41	0.004356569	1.790842533	up
Brpf1	0.004359727	1.641768813	up
Gng10	0.004382062	1.805803299	down
Lphn3	0.004382062	1.745946884	down
Zc3hc1	0.004388328	1.506136537	down
Stxbp3	0.004388961	1.62592721	up
Alg10b	0.004398928	1.51011014	down
D230019K20Rik	0.004399319	1.542418957	up
Aco2	0.004403157	1.508116126	up
Raly	0.004403343	2.511213303	down
LOC100040897	0.004409718	1.941003323	up
Akap13	0.004415554	1.573993444	up
Osbpl5	0.00442063	1.530594945	up
scl0002547.1_9	0.004421886	1.663756013	down
D330035D07Rik	0.004421991	1.865784168	up
Gpr153	0.004452377	1.725471854	up
Alg8	0.004458599	2.290554285	down
Sar1b	0.004464801	2.015235186	down
Fermt3	0.004465505	2.080588341	up
Snrpd1	0.004472939	2.621015787	down
Gnas	0.00447796	1.52675271	down
Ptprd	0.00447796	1.835583448	up
Tug1	0.00447796	2.31597209	down

Wwtr1	0.004482235	1.507518649	down
Ngfr	0.004483726	2.637902737	up
9530014D17Rik	0.004487468	2.243878365	up
D230032H14Rik	0.004491247	1.522387147	up
Slc26a6	0.004506724	1.759891391	up
Erich1	0.004510757	1.613199472	up
Sumo1	0.004514331	2.395817041	down
Lat	0.004519082	1.69903791	up
Paip2	0.004524263	1.548714042	up
Ugt1a10	0.004525196	1.916568518	up
2610019E17Rik	0.004544528	1.589625716	down
LOC100048721	0.004544528	1.810512781	down
AI316807	0.004551012	1.565317392	up
Hoxb9	0.00455276	2.278700352	up
Bmp1	0.004553463	1.86990571	up
Anxa6	0.004558347	1.601871729	up
mtDNA_ND5	0.004561616	2.46696043	down
Sgsm2	0.004561616	1.837571859	up
Wdr8	0.004562427	1.753470659	down
Asph	0.004563694	1.963395834	down
Neu1	0.004565391	1.840516925	up
2210410D02Rik	0.004565694	1.851871014	down
Ttll12	0.004568313	1.524618149	up
Slc46a1	0.00456836	1.540046215	up
Tmem60	0.004575928	2.360965729	down
Lmnb1	0.004585581	6.803228855	down
Lrfn4	0.004586404	1.824934244	up
C530001D20Rik	0.004591937	1.573381424	down
Ndufb10	0.004591937	1.756159306	up
Dbndd1	0.004604195	1.925771713	up
Hsd17b11	0.004607201	3.419238806	down
EG632248	0.004609945	4.16443634	down
Adamts8	0.004615928	1.504394054	up
Mbtps2	0.004616031	1.571812153	down
Zswim6	0.004623151	2.522022486	down
Ercc5	0.00462606	1.680451274	down
Ctsb	0.00463177	1.987621546	up
B230337C21Rik	0.004631903	2.546240091	up
A730029I18Rik	0.004631994	1.655958653	up
Rhbdd2	0.004631994	1.896586418	up
scl0001602.1_506	0.004631994	2.063677549	down
Lsm14b	0.004633517	3.277315378	down
Cbx6	0.004648326	1.631685615	up
Jup	0.004648326	1.872483373	up
Dlx5	0.004652189	1.559503198	down
Ctsk	0.004652794	1.602247715	down
Rexo1	0.004652794	1.53508544	up
Foxred1	0.004653975	1.834370732	down
D15Ertd528e	0.004654621	1.527486801	up
Ppm1l	0.004674154	1.582718134	up
Rcsd1	0.004674154	1.788772225	up
scl0003996.1_126	0.004691938	1.658458948	down

AI875142	0.004707248	1.615317106	up
LOC545487	0.004707248	1.776695013	up
Bcl2l1	0.004709305	1.546980739	down
Zbtb7a	0.00471114	1.884957552	up
Impdh1	0.004718251	1.510647774	down
Nsbp1	0.004719961	3.795404673	down
Nudt4	0.004723395	2.079841614	down
Rnasen	0.004726149	2.084136725	up
1110046J11Rik	0.004740237	1.934075236	up
Nptn	0.004741892	2.036044359	up
Ggta1	0.004747286	1.871118188	up
C80913	0.004748617	1.696633697	down
Rnf170	0.004752605	1.632795811	down
6030458C11Rik	0.00476515	2.004194737	up
Scyl1bp1	0.004767763	1.622684956	down
2310022K11Rik	0.004777185	1.69041431	down
Ephb2	0.004777502	1.536585331	down
D230023E14Rik	0.004783107	1.550012827	up
Edf1	0.004790453	1.567381144	up
Lrrc14	0.004790453	1.656934977	up
Fmnl2	0.004807578	2.74194026	down
HPBRII4	0.004817992	2.928853989	up
2510022D24Rik	0.004824178	2.407793284	down
6430511F03	0.004827902	1.602097392	up
Polr3f	0.004831156	1.607256055	down
5430433G21Rik	0.004840155	2.294437885	down
5830411J07Rik	0.004840155	1.540522099	up
Ict1	0.004840155	1.980940938	up
Dpp9	0.004868642	1.800584674	up
Naglu	0.004868642	1.592426539	up
Gtf3c4	0.004869718	2.05004859	down
Rnf14	0.004875743	1.554387689	down
5630401D24Rik	0.004882396	1.529340267	down
Car15	0.00488762	1.580145598	up
2810405K02Rik	0.004890215	1.519294739	up
D12Ertd647e	0.004890215	1.83235085	up
Alg9	0.004895086	1.56601882	down
Tmem192	0.004896346	1.616845727	up
Alg8	0.004903709	1.755251885	down
Ankhd1	0.004907392	1.838685513	up
4930402E16Rik	0.004910303	1.959052205	up
Flnc	0.00491357	2.133637667	up
Chst8	0.004914698	1.671821713	up
Asph	0.004919082	1.98672688	down
Frzb	0.004926147	1.579760313	up
LOC100046467	0.004935826	1.92604661	up
4833428M15Rik	0.004943388	1.650403261	up
A930014D08Rik	0.004943388	1.985752583	up
E330024P20Rik	0.004943388	1.625862241	up
Cdh13	0.00495666	1.615247726	up
EG665646	0.004964224	2.124993324	down
D030064C08Rik	0.00496564	1.701019168	up

C030010B13Rik	0.004970646	1.57362318	up
D230035M11Rik	0.004972111	1.536402702	up
Rhobtb3	0.004974162	1.504313469	down
Peo1	0.004979413	1.660571098	down
Cyp27a1	0.004982191	1.676168323	up
Sgol1	0.004995079	1.553628326	up
Mical1	0.005010033	1.592559576	up
2510047L19Rik	0.005012261	1.510557771	down
Axl	0.0050198	1.648225427	up
Slc9a8	0.005031459	1.734564304	up
Ccnt1	0.005037142	1.690817475	up
Cenpt	0.005037142	1.8164258	up
Cars2	0.005040272	1.561414003	up
Lrrc16b	0.005049709	2.217592478	up
Mcts1	0.005049709	1.609264493	down
Grik5	0.005062176	1.657815695	up
2410018L13Rik	0.00506219	2.196941376	down
Hprt1	0.005075932	1.948389411	down
Cse1l	0.005080655	1.519023299	down
LOC331507	0.00509244	2.226765871	down
Npepl1	0.005100557	1.711185455	up
A030007L17Rik	0.005107491	1.544584394	down
Epha4	0.005107491	1.665304303	up
Rab28	0.005108263	1.852225065	down
Yipf5	0.005108263	1.63463676	up
Fam171a1	0.005114235	1.51014781	up
Hdac7	0.005132957	2.353862286	up
LOC100046401	0.005137407	1.946298957	up
Arhgef1	0.005138093	1.593886375	up
LOC268569	0.005141006	2.831551552	down
Nfu1	0.005141006	1.652709723	up
Gphn	0.005141355	1.870468378	up
Eef1e1	0.005146779	2.135507345	down
Asb7	0.005150687	1.532820225	down
1700034P14Rik	0.00516316	1.519303322	down
Bbox1	0.005170662	1.536293984	down
Igsf11	0.005177238	1.629356027	down
2810417K24Rik	0.005183023	2.255563021	down
C130074O09Rik	0.005186057	1.897568703	up
3110082D06Rik	0.005186502	1.806535721	up
Zic4	0.00519353	1.863726735	up
Acpl2	0.005205416	1.52359724	down
Fam122b	0.005219139	1.527989864	down
Adamtsl2	0.005224925	1.906339645	up
Cpne1	0.005230845	2.397340059	up
D530037H12Rik	0.005237694	1.93134892	up
Trappc6b	0.005238731	2.099305868	down
LOC626259	0.005245532	1.688784957	up
Tfip11	0.005251227	1.77641964	up
Blvra	0.005261303	1.72979033	up
A230057M07Rik	0.005264175	1.823416829	up
Bbs10	0.005265005	1.563109994	down

LOC100048372	0.0052724	1.545868874	up
6720422M22Rik	0.005276729	2.376379252	up
Atp5a1	0.005293693	1.523203969	up
D14Ertd436e	0.005303757	1.558619976	up
Rab14	0.005303757	2.007956743	down
Hprt1	0.005306407	1.744814515	down
B930032P17Rik	0.005307295	1.644609213	up
1700020I14Rik	0.005308216	1.623444915	down
Traf2	0.005316884	1.579707861	down
Ssbp1	0.005324197	1.722686291	up
A130021K16Rik	0.005330659	1.829400778	up
Amigo1	0.005336412	1.659139276	up
LOC194905	0.005356513	1.711860538	up
C79267	0.005357334	2.197933912	up
LOC386112	0.005363497	2.618575096	up
LOC100040182	0.005370474	2.000064373	up
Cntfr	0.005375562	1.530892491	down
Nadsyn1	0.00537588	1.519453049	down
9430020M11Rik	0.00538238	1.60305202	up
Mtssk	0.00538238	1.718600035	up
Hsd17b11	0.005383089	2.970896244	down
Zbtb12	0.005383089	1.870143294	down
Rnase4	0.005383271	1.609344482	up
9530048I18Rik	0.005386025	1.995976567	up
Phf12	0.005388652	1.701747537	up
Tank	0.00540736	2.19803071	down
Usp13	0.005408874	1.531322122	up
6030455K13Rik	0.005427571	1.761798143	up
Xkr5	0.005438238	1.539257407	up
Slmap	0.005438376	1.660367608	down
Tmem86a	0.005438531	1.728254199	up
Cd44	0.005446337	2.069503546	up
5330401F18Rik	0.005458402	1.827988505	up
BC003993	0.005466017	1.627212644	down
Ubfd1	0.00547539	1.661820293	up
9530010C24Rik	0.005478845	3.003012419	up
Fndc5	0.00547886	1.599969387	up
LOC547150	0.005492091	1.893796086	up
Senp1	0.005493636	1.832995772	up
Lpl	0.005504496	2.378626585	up
6030422A11Rik	0.005504545	1.707243204	up
Zfp319	0.005504545	1.673882365	up
6820437F20Rik	0.005507312	2.888751984	up
2310047O13Rik	0.005510776	1.553020954	down
Gpr137b-ps	0.005512436	1.671805501	up
2210401K01Rik	0.005512649	2.492856503	up
Spg7	0.005519371	1.590421319	up
Bmp4	0.005522695	1.95533061	down
Casp2	0.005523855	1.78456223	up
C430014K11Rik	0.005543066	1.709342122	up
5530401D11Rik	0.005547452	1.576612711	up
6720482D04	0.005550433	1.572695851	up

Ihpk1	0.005550883	2.123453856	up
3830612M24	0.005551574	1.55578661	down
Nrip1	0.005589256	1.901708245	up
Lats1	0.005634014	1.504541278	down
Efna1	0.005654887	1.963816285	up
5730588I11Rik	0.00565875	2.230804205	down
Meox1	0.005663541	1.516085982	up
Rybp	0.005663541	2.688875437	down
Traf5	0.005663541	1.698015213	up
Coq10a	0.005675729	1.73787272	up
Klf12	0.00568846	1.634319186	up
Nkiras1	0.005691702	1.80529964	up
C130064E22Rik	0.005715569	1.67504251	up
Hbb-b1	0.005717211	11.01275826	up
Ercc8	0.005717423	1.588830948	down
G430046L24Rik	0.005726233	1.94635582	up
LOC270665	0.00572809	2.291697979	down
4930461P20Rik	0.005733859	1.505496025	down
A230054D04Rik	0.005733859	1.724951625	down
Tmem209	0.005734813	1.625501871	up
Nol8	0.005736833	1.943742633	down
A930023F12Rik	0.005738542	2.036361933	up
scl0002917.1_537	0.00574402	2.392357349	up
1700065A05Rik	0.005755828	1.54212153	down
Ptges3	0.005755828	1.512616515	up
A930041H05Rik	0.005756887	2.0625844	up
Slc18a2	0.005756887	1.563637495	down
Tbxa2r	0.005759698	1.522086501	up
Fat3	0.005762611	2.421111107	up
2900086B20Rik	0.005776132	1.876333356	up
Neil3	0.005776764	1.662075877	up
Nrp1	0.005776764	2.144967794	up
Etfb	0.00580356	1.617002487	up
LOC100047199	0.005818188	1.692160964	down
Tmem106a	0.005819449	1.815490961	up
Mis12	0.00582065	1.669887424	up
9430011C21Rik	0.005828396	1.669133425	up
EG639396	0.005833225	2.0125916	up
Pomt1	0.005848301	1.609034896	down
Gpt1	0.005856303	2.055529594	up
LOC632230	0.00585949	1.605922818	up
Polr2k	0.005861284	1.529259324	down
5832402A02Rik	0.005863638	2.08346343	down
D130046C19Rik	0.005868259	1.791828394	up
Tmem138	0.005869458	1.766551733	up
Ltbp4	0.005875729	2.162314415	up
Cd248	0.005890566	1.979718924	up
Mzf1	0.005895618	1.586893082	up
Ift20	0.005898799	1.633449197	down
Cdon	0.005925043	1.829356194	down
Aldh16a1	0.00592869	1.529728532	up
BC024479	0.005941432	1.52166009	up

Msrb3	0.005945401	1.54449749	down
Khk	0.005958943	1.667796016	up
9130415E20Rik	0.005965845	1.927278638	up
Vav2	0.005965845	1.735313058	up
Prodh	0.005970187	1.555651903	up
Rhbdd3	0.005971105	1.788918138	up
Angptl2	0.005972808	1.522541285	down
Samd4b	0.005972808	1.63491559	up
C430002D13Rik	0.00598027	1.831830621	up
Dchs1	0.005982289	1.511523962	up
Tgfb1	0.005983145	2.191161633	up
Adamts18	0.005984629	1.659858108	down
Cd52	0.00600267	1.619703412	up
E130102H24Rik	0.006024243	1.527656674	down
Rrs1	0.006045575	1.514943242	up
Dapk1	0.006055682	1.667805314	up
Gart	0.006068474	2.161578417	up
Tpk1	0.006078617	1.909199596	down
Pdgfrb	0.006105201	1.742510676	up
Sp3	0.006109928	2.323744059	up
Serpinb6a	0.006120017	1.912746906	up
Ssu72	0.006122753	1.519582152	up
Mtap9	0.006129619	1.615566135	up
Nox4	0.006130789	1.560741186	up
scl0001546.1_9	0.006140406	1.5075804	down
5830431I15Rik	0.006146356	1.506254196	up
Sall1	0.006147947	1.54611373	down
Trex1	0.006147947	1.811471939	up
Tro	0.006147947	1.722679257	up
Usp21	0.006163162	1.599565387	up
Olfr297	0.006167786	1.54493022	down
Nit1	0.006177865	1.79923749	up
Zfp346	0.006177906	1.528286099	up
Rapgef2	0.006204609	1.643891573	down
2900076A13Rik	0.006206536	2.147435904	up
A630086P05Rik	0.006206536	1.728210688	up
LOC100040505	0.00620969	1.609685659	down
Eif1ay	0.006212865	2.10759449	down
6030408C04Rik	0.006236507	1.538253665	down
Lrp6	0.006256679	1.753937602	down
Dnahc2	0.006257913	1.502902627	up
Tmpo	0.006257913	1.981946707	down
Slc37a4	0.006259788	1.604335785	up
Ccdc124	0.00627255	1.611684561	up
H1fx	0.0062869	1.55358541	up
Mapk1ip1l	0.006291644	1.577905297	down
Recql4	0.006291644	1.511767268	up
Tmem110	0.006291644	1.717358589	up
Slc6a17	0.006295051	1.5684551	up
Psmd10	0.006296875	1.788839459	down
Llph	0.006307926	1.501343131	down
Acrbp	0.00631118	1.657830715	up

Zdhhc7	0.0063267	1.714392781	up
LOC638301	0.006376049	1.66268611	up
Myd88	0.006377279	1.865250111	up
Pam	0.006381533	1.759858728	up
LOC381739	0.006390806	1.53563726	up
Rgs4	0.006390806	1.52838552	down
Usp40	0.006391777	1.582900286	down
8430438L13Rik	0.006403898	1.507282257	up
Rps6ka5	0.006403898	1.908997893	down
LOC384888	0.006409206	1.544514179	down
Cchcr1	0.006434869	1.753664374	up
2410187C16Rik	0.006446272	1.716914654	down
A430005L14Rik	0.006446718	1.770915151	down
Mrpl20	0.006460141	1.623801112	up
Crybg3	0.006462977	1.740251303	up
Ela1	0.006477522	1.761190891	up
LOC100048196	0.00648168	1.521303892	down
Numbl	0.006511483	1.558754206	up
Gtf3c1	0.006511806	1.64692986	up
H2-Q5	0.006526819	1.563943863	up
LOC280487	0.006532069	4.341693878	up
A430110N23Rik	0.006536723	1.611422777	up
5730412F04Rik	0.006545079	1.517970085	up
6720484G13Rik	0.006558534	2.16824007	up
Rai16	0.006558534	1.506459832	down
Itpr3	0.006563694	1.780572414	up
A630075K04Rik	0.00656485	2.342200518	up
mtDNA_ND3	0.006570143	2.006347656	up
B930095M22Rik	0.00658334	1.835411072	up
Med20	0.006583931	1.579709888	up
Hnrpa0	0.006584297	3.883134604	down
9930016F01Rik	0.006590848	1.582425952	up
Eif5	0.006590848	2.419942617	down
Kat2a	0.006590848	1.551998138	up
LOC677528	0.006590848	2.240868807	down
Orai1	0.006597783	1.714620352	up
Psme3	0.006612435	1.721612215	up
Cops5	0.006613039	1.533055544	down
EG624187	0.006655193	1.574839473	down
D630024O11Rik	0.006657379	1.526559115	down
Eif3m	0.006658135	1.769789219	up
LOC433225	0.006669584	1.571968675	up
Nlrx1	0.006675617	1.595674872	up
Trpm3	0.006679846	1.53155458	down
LOC280097	0.006684156	2.596028566	up
Pqlc2	0.006686962	1.507405043	up
Mapk11	0.006693395	1.957633734	up
Ppp3ca	0.006700583	1.671500087	down
Cacnb1	0.006703834	1.914827824	up
Jdp2	0.006724594	1.564003229	down
D930030O05Rik	0.006726141	2.064258575	up
Ccdc21	0.00675559	1.608210802	up

Plekhb1	0.006772305	1.838961244	down
B930083D07Rik	0.006778633	1.546908021	up
5330435L01Rik	0.00678243	1.730686903	up
Crat	0.006789114	1.672328115	up
Wdhd1	0.006789337	1.890445948	down
Rnf130	0.006796084	1.755230188	down
LOC673251	0.006799945	2.023995638	up
Zbed4	0.006801098	1.814566135	up
6430510B20Rik	0.006827403	2.03238821	up
Plekhh3	0.006844669	1.506163836	up
Ero1l	0.006847903	2.254576683	down
9530082I15Rik	0.006858739	2.117924929	up
Dock7	0.006865747	1.701973796	down
LOC667776	0.006874622	1.606609345	down
Tbrg4	0.006875109	1.539784312	up
Eif3j	0.006879433	1.555539489	up
4933412E12Rik	0.006893668	1.587571621	up
Kif4	0.006901765	1.595390678	down
D130056F09Rik	0.006945476	1.519127488	up
BC028528	0.00694874	1.729522348	up
Yod1	0.006949807	1.717067719	down
Hgf	0.006952351	2.11736989	up
Zfp768	0.006957803	1.780971408	up
Sgpl1	0.006958703	1.68122673	up
Sumf1	0.006966741	1.816872358	up
1110001C20Rik	0.006984963	1.970763207	up
A730059M13Rik	0.006997832	1.756013632	up
Dynlt3	0.007013311	1.600622416	up
Hagh	0.007047785	1.698992133	up
Col23a1	0.007055892	1.654782653	up
2900084I15Rik	0.007061101	2.520194769	up
Topors	0.007063613	1.521262407	down
Sfrs8	0.00706396	1.503165126	down
Whsc1l1	0.007074345	1.747263551	up
LOC433955	0.007074375	1.823660255	down
Rac1	0.007090396	1.570001602	down
Tomm70a	0.007093607	1.830922127	up
Cit	0.007096448	1.884291768	up
Zbed3	0.007097998	1.952820301	up
Crocc	0.007107576	1.50797689	up
Heph	0.007113596	1.744532108	down
Cugbp1	0.00711619	1.674615145	down
2610024G14Rik	0.0071274	1.509647369	down
BC031748	0.007128548	1.636994362	down
Ccdc49	0.007146164	1.593333364	up
9330154F10Rik	0.007173993	1.729670644	up
Gzf1	0.007177319	2.20673728	down
Nfatc1	0.007184153	2.426151514	down
Sidt2	0.007212758	2.055463076	up
LOC100040413	0.007213186	1.538741589	up
Ccnt2	0.007242712	1.706705809	down
Fgl1	0.007250884	1.56188643	up

2610009E16Rik	0.00726379	2.025816679	up
9630027M13Rik	0.007264113	1.78845489	up
Sema3d	0.007264698	2.825622559	down
Bach2	0.007266089	1.610206008	down
D3Ertd254e	0.007266806	1.725127697	up
Yipf1	0.007268749	1.664879918	up
Tsen54	0.00726983	1.732044101	up
Zdhhc20	0.00729516	1.697941423	down
Stat3	0.007295576	1.71934104	up
C820018A03Rik	0.00730188	1.824666023	up
Lbh	0.00730188	1.930043578	down
Megf6	0.007306946	1.525348306	up
LOC385905	0.007320766	1.583931804	down
4432404P07Rik	0.007321294	1.530806541	up
6430540A14Rik	0.007326144	2.133250475	down
C030011O14Rik	0.007326144	2.166157246	down
LOC100045864	0.007334522	2.292016029	up
Sox18	0.007335377	1.547066927	up
LOC100047837	0.00733701	1.754850745	down
Vgll4	0.00733701	1.723965287	down
0610037P05Rik	0.007339487	2.161350489	down
Papd1	0.007360063	1.592164397	up
Gas8	0.007370448	1.571739078	up
Il10ra	0.007370448	1.87403357	up
Tgfb2	0.007371847	2.04001236	down
Pcf11	0.007406446	1.578695774	down
Edil3	0.00742321	1.737264156	down
A830094I09Rik	0.007425921	1.538524389	up
Bcl2l1	0.007446119	1.568951726	down
Ahi1	0.007457739	1.553654432	up
1810006K21Rik	0.007469793	1.502086163	down
3010015K02Rik	0.007486884	1.568228602	down
Csf3r	0.007486884	1.615186572	up
Mrvi1	0.007510294	1.755532622	up
6720411N02Rik	0.007520549	1.959032178	up
P4ha1	0.007525927	3.943559647	down
Cybb	0.007530027	2.103700638	up
Fgf10	0.007545813	1.955385685	up
Arhgap4	0.00755505	2.087489605	up
Slc2a6	0.00755505	1.614160061	up
AI426953	0.007559771	1.505065322	up
6720406L13Rik	0.007574352	1.58174026	up
Cpa2	0.007593787	1.565110564	up
Sertad4	0.007594778	1.795625091	down
B230345P09Rik	0.00760504	1.590972543	up
Cct5	0.007605317	1.723612666	up
Etnk1	0.007617883	1.760130048	down
1810011O10Rik	0.007619218	1.849944472	up
Lemd2	0.007619218	1.64338541	up
Hnrpa3	0.007620641	1.7768327	down
Eral1	0.007623511	1.71159935	down
Mmrn2	0.007642384	1.707075596	up

Hcn3	0.007646611	1.596190691	up
2810008M24Rik	0.007649352	1.763946772	down
9430068D24Rik	0.007649352	1.715135455	up
1600027N09Rik	0.007653305	1.516192198	up
A130064M08Rik	0.007658593	1.746639609	down
2900057K09Rik	0.00765973	1.506816864	up
D430033H22Rik	0.00765973	1.917791724	up
Fkbp2	0.007661977	1.548802495	down
A330081F11Rik	0.007665943	1.566189885	up
Zswim4	0.007668739	1.62197876	up
Snx16	0.007672016	1.689698577	down
Snrpb	0.007672081	2.195340395	down
Arrb2	0.007678956	2.324901342	up
AU022252	0.007708447	1.544862509	down
Kif3a	0.007720362	1.583743095	up
Hddc3	0.007723078	1.602972627	up
LOC625074	0.007729635	2.159573317	down
Timp3	0.007730238	1.953484774	down
Strn4	0.00774313	1.579439163	up
Crlf1	0.007746725	2.248845816	up
4930453J04Rik	0.007749501	1.777502894	up
Sparc	0.007753571	1.542048097	up
Gpt2	0.007764532	1.777121782	up
Setd4	0.007779322	1.624795914	up
Aco2	0.007783343	1.567709565	up
Mapbpip-pending	0.007811056	1.863490582	down
2610204K14Rik	0.007815612	1.655524254	down
Mnat1	0.007825735	1.589990497	down
Spag9	0.007832808	1.92768085	up
Psmb10	0.00783932	1.606684089	up
B930001L07Rik	0.007888787	1.505724788	up
Malt1	0.00789234	1.50060451	up
Wipf1	0.007897073	1.531025767	up
Map3k14	0.007908676	1.595227003	up
Banf1	0.007919827	1.724830985	down
D030067L12Rik	0.007938294	2.271827221	up
Abcd2	0.007941433	1.52710855	down
Cenpa	0.007943437	1.62842083	down
9030420J04Rik	0.007950031	1.654085875	down
Map3k12	0.007965604	1.673395276	up
Fxyd3	0.007980116	1.668602943	down
Hoxa11	0.007983535	2.684180737	up
Igh-6	0.008019278	1.630828857	up
Dpagt1	0.008060529	1.541120768	up
A730085F06Rik	0.008060628	1.764216542	up
D7Ertd791e	0.008063247	1.951877713	up
Fam161b	0.008063247	1.623941183	up
Bbx	0.008063726	1.656403065	up
Trim45	0.008070354	1.506572127	up
A130036M01Rik	0.008071975	1.550291538	up
Lars	0.008085735	1.524615288	down
LOC100043822	0.008118144	1.571355224	up

5930436O19Rik	0.008123036	2.281885386	up
Irf3	0.008123036	1.772134185	up
Myo5a	0.008123604	1.863202572	down
Dnalc1	0.008133831	2.035719633	down
Itgb1	0.008136091	1.658383369	down
Cenpp	0.008140866	1.542114377	down
Dnmbp	0.008140866	1.532333851	down
LOC676779	0.008163164	1.711494803	up
Apip	0.00816882	2.063208818	down
Map2k2	0.008179583	1.528298974	up
4733401H18Rik	0.008183	1.513824701	up
Adra1a, GPCR	0.008184932	1.502889156	up
Chuk	0.008186054	2.294175386	down
6430510B20Rik	0.008188204	1.551620007	up
Lrrc29	0.008195794	1.521802068	down
Mtmr14	0.008195794	1.788615465	down
Phf6	0.008199561	4.024706841	down
H1f0	0.008201217	1.588948965	up
A830085I22Rik	0.008217461	1.662042499	up
LOC231869	0.00822289	1.6033746	down
LOC632664	0.008223435	1.892603517	down
Ltbp2	0.008226108	2.101990461	up
Zfyve16	0.0082346	1.587528944	down
Tbc1d19	0.008238553	1.680420637	up
Cars	0.008244781	1.905957341	up
Rasa4	0.008248474	1.622083306	down
scl0001987.1_16	0.00825443	2.794271469	down
Hbb-b1	0.008257683	10.36927605	up
1110067D22Rik	0.008272398	1.642856002	down
A430005L14Rik	0.0082726	1.847074986	down
Ndor1	0.0082726	2.366375923	up
LOC385653	0.008290377	1.7332021	up
2810428I15Rik	0.008294058	1.82010591	up
5730406M06Rik	0.008306185	2.215594053	down
Heatr1	0.008311891	1.579590082	up
Vat1	0.008313851	2.656697989	up
A130056J21Rik	0.008329433	1.658133864	up
W91709	0.008335569	1.751603484	up
Mvp	0.008339453	2.110854149	up
Asph	0.008341668	1.670410752	down
Gas6	0.008390803	2.446539879	up
A230061N24Rik	0.008395216	1.781887412	up
B9d2	0.008400626	1.663969159	up
Coq10b	0.008403073	2.124149323	down
9930031P18Rik	0.008403851	2.972957611	up
Ogn	0.008420856	2.050099611	down
Ring1	0.008420856	1.561141014	up
Tmem175	0.008420856	1.527140856	up
Gpaa1	0.008434706	1.619491816	up
Cables2	0.008437568	1.5495435	down
Lsm6	0.008448868	1.530593514	up
Odz2	0.008453477	1.597476363	up

Dck	0.008486369	2.230581284	down
Lmf2	0.00848947	1.570897222	up
Samd9l	0.008507475	1.850743532	up
Cdk5rap1	0.008507537	1.920630217	down
Lig3	0.008518401	1.570862413	down
Abcc3	0.008541925	2.24664855	up
Myh8	0.008542935	1.840352058	up
Sobp	0.008555383	1.753223777	down
Cnn2	0.008562737	2.338953495	down
Notch4	0.008597329	1.774282813	up
Chic2	0.008601704	1.743302584	down
D030038A19Rik	0.00860244	1.560141683	down
Ppa2	0.008621655	1.895563006	down
B130047N10Rik	0.008621679	1.734124184	up
1110003P22Rik	0.008646946	1.704634547	up
6030432P03Rik	0.008646946	2.691040039	up
Blcap	0.008646946	1.580827475	up
Pmepa1	0.008646946	1.68865335	up
1810011H11Rik	0.008665488	1.799540281	up
Nol3	0.008674676	1.744079709	up
Sp4	0.008695202	1.592820764	up
Hectd1	0.00869523	1.569911122	up
LOC385157	0.008707533	1.53238976	down
Tspan9	0.008710968	1.64148283	down
C230089D15Rik	0.008739411	1.55996418	up
Shmt1	0.008742416	1.634525418	down
Pcdhb17	0.008748099	2.34493041	up
2900045G02Rik	0.008761293	1.542642236	up
LOC634327	0.008770812	1.843144059	up
2310047C21Rik	0.008778505	1.908691526	up
Pik3cg	0.008778505	2.137208462	up
Elovl4	0.00878603	1.584355354	up
Stat3	0.008799612	1.732340813	up
Hbb-b1	0.008808971	11.68254185	up
Hist1h3c	0.008812988	1.704137564	down
Lmo7	0.008819644	1.513558269	up
Set	0.008820343	1.526901245	up
Rad1	0.008826394	1.649336696	down
Nfe2	0.008831919	1.718353748	up
Vldlr	0.008837847	1.6878438	down
Hnrpa2b1	0.008863835	1.699638963	down
Zfp618	0.008875394	1.567986131	up
Msh2	0.0088838	1.618874908	up
Egflam	0.008915268	1.92677033	down
D630021H01Rik	0.008946964	1.902314067	up
Hmcn1	0.008964982	3.694500208	down
Cenpq	0.00896723	1.5536232	down
Hs3st1	0.008971364	1.553391099	down
Nrf1	0.008974687	1.678323507	up
E130203B14Rik	0.008976108	1.60699892	up
Lrrc59	0.00902264	1.913923025	up
Hs3st3b1	0.00904921	2.216298819	down

Spsb1	0.009068527	2.480366468	down
LOC385019	0.009087567	1.952277184	up
Cd14	0.009090838	1.905990839	up
Fap	0.009111305	1.579413056	up
Chst11	0.00911522	1.705717921	down
Fnbp1	0.009119293	1.915147185	up
LOC100041172	0.009156411	1.973166108	down
LOC622994	0.009161793	1.529569268	down
A630082K20Rik	0.009162616	4.443362713	down
9130024F11Rik	0.009164334	1.864141822	down
Mad	0.009166428	1.920981526	up
Cdkn1b	0.009167351	1.725129128	down
Jag1	0.009167351	2.487917185	up
LOC674960	0.009171879	1.702018976	down
Irak1	0.009172902	1.520924449	up
2810001G20Rik	0.009178686	1.537947655	down
EG633285	0.009201883	1.625074506	down
LOC100042749	0.009208008	1.772324443	down
Myl9	0.009212836	1.785691381	down
4930402H24Rik	0.009220541	1.547660351	up
EG432649	0.009226141	1.652814984	down
B230345P09Rik	0.009262146	1.672502518	up
Psmc4	0.0092637	1.759011865	down
A730008L03Rik	0.00928941	1.506702185	down
Ankrd39	0.009292291	1.502973676	down
D030038I21Rik	0.009300808	1.587367058	down
Pvalb	0.009308955	1.763083696	down
Esm1	0.009309885	1.808077216	up
Osbpl1a	0.009310634	1.561669827	down
Unc5b	0.009335274	1.792660475	down
Zfhx3	0.009338832	1.900578857	up
LOC671641	0.009343702	1.604619265	down
Mon1b	0.009360699	1.941350937	up
Zfp740	0.009386306	1.75446856	up
Sesn3	0.009386758	2.475210667	down
Btf3	0.00941287	1.503463268	down
A630006E02Rik	0.009424369	1.582386255	up
Smchd1	0.009428795	2.070747137	down
Tmem39b	0.009433996	1.683129549	up
Gabarapl1	0.009434327	1.636587262	up
Comp	0.009446345	1.549821734	down
Coro1a	0.009455524	1.964322329	up
4930527B16Rik	0.009458085	2.029313564	up
Fin14	0.009458085	1.638801456	up
Dbnl	0.009463659	1.51051414	up
Evl	0.009470629	1.865595222	up
2900056P18Rik	0.009481836	1.604686618	up
Pcdh7	0.009481836	1.887733936	up
2010013I23Rik	0.009509441	1.552217126	up
Tmem2	0.009520023	1.734024167	down
B330002M01Rik	0.009558775	1.855526209	up
Mgll	0.009578668	1.527316213	down

Chd2	0.00958309	1.902526021	down
Pcdh10	0.009588509	1.756918907	up
BC018101	0.009602986	2.036296606	down
1600002K03Rik	0.009611566	1.805472732	up
Heatr5b	0.009611566	1.657566786	down
Kpnb3	0.009611566	1.710688949	up
Psmb9	0.009641325	1.73197794	up
Lrp12	0.009658743	2.219601631	down
4932415A06Rik	0.009660511	2.045112371	up
5730427N09Rik	0.00966764	1.687896848	down
Pde3a	0.00966764	1.686952591	down
Ncoa5	0.009678399	1.958404422	up
Ddx19b	0.009683654	1.612854123	up
6820401H01Rik	0.009695361	1.560216188	up
Abcg5	0.009718075	1.619966626	down
3110098I04Rik	0.009726824	1.545076847	up
2900064A13Rik	0.009727925	1.531754255	up
Pcdhga2	0.009774162	1.580206633	up
LOC677216	0.00979091	1.531238794	down
Ccdc123	0.009806793	1.604761481	up
Nt5dc1	0.009806793	1.52340281	down
Acta1	0.009818102	2.08486104	up
Armc6	0.009818259	1.69775629	up
Mvd	0.009838119	1.836176395	up
Hbb-b1	0.00984496	10.18876648	up
Asna1	0.009863357	1.699867249	up
Rdx	0.009877494	1.53045404	up
Dact1	0.009898949	2.167698622	down
9230105E05Rik	0.009901953	1.88351047	up
Acox1	0.009907384	1.558616877	up
Chic1	0.009909249	1.547627926	up
Opn3	0.009909249	1.521458864	down
Myg1	0.009915611	1.672569156	down
9130023F12Rik	0.00991959	1.746603489	up
E030022I16Rik	0.009923281	1.870226026	up
Jun	0.009923281	1.758324981	up
Fam13b	0.009942399	1.568321943	down
St6galnac6	0.009942576	1.544875264	down
D630038D15Rik	0.009942678	1.756213546	up
5330423I11Rik	0.009948895	2.764455319	up
Cntnap4	0.009966043	1.799120307	up
3222402P14Rik	0.009970452	1.777629852	down
Zfp704	0.009976918	1.911498547	up
Fkbp10	0.009979163	1.802869082	up
Nov	0.009984454	1.56094873	up
LOC100046056	0.010037093	1.606394649	up
Col4a3bp	0.010038231	1.616967559	down
LOC635455	0.01003891	1.614647269	down
LOC667582	0.010053158	1.81360054	up
Mthfsd	0.010053158	1.535512686	up
Rnd3	0.010053158	2.658306122	down
Zmynd17	0.010063926	1.522825241	up

Abi1	0.010065002	1.689291954	up
Cntn1	0.010065002	1.655365348	down
LOC100044696	0.010082787	1.946960926	down
Msl31	0.010085437	1.614193797	up
Zw10	0.010113312	1.505553246	down
Abcb6	0.010119963	1.611995339	up
2310043N10Rik	0.010135837	2.246186018	up
Foxp2	0.010155139	1.72091639	down
Maml2	0.010211166	1.647469759	down
Cald1	0.010219355	1.581065774	down
Ccdc99	0.010219355	1.664839625	down
Cpm	0.010219355	1.511164546	up
Camkv	0.01023847	1.606053829	up
Skil	0.010240001	1.675926328	up
Itga9	0.010271408	1.867954493	up
scl0001617.1_31	0.010274079	1.660932064	down
Ptprv	0.010318783	2.217592716	up
C030039L03Rik	0.01037596	1.568364263	up
Hoxb7	0.010384735	2.053483725	up
Slc26a7	0.010403797	2.313343048	down
Lrrc8d	0.010409291	1.506517887	down
Phlpp	0.010411132	1.588183165	up
Nbl1	0.010422678	1.588342786	up
Hbb-b1	0.01045256	9.665819168	up
A730036E13Rik	0.010457528	1.947786808	up
Reps1	0.010457947	1.549705744	down
Lca5	0.010472329	1.678013563	down
Gins3	0.010476746	1.531025171	up
Gga3	0.010481935	1.60785532	up
LOC636450	0.010510104	1.676447988	down
9430088B20Rik	0.010548236	1.760051012	up
Eda2r	0.010548236	1.682371259	up
5730406F04Rik	0.010549391	1.636396647	down
S100pbp	0.010558038	1.701884747	down
Soat1	0.010595559	1.511939764	up
Fbn2	0.010622274	1.77337563	up
C230066I17Rik	0.010624968	1.695573926	up
2610207F23Rik	0.010653148	1.546695232	up
Ehd1	0.010679529	2.436515808	up
Cecr5	0.010699264	1.563346624	up
Nrp1	0.010715192	1.895717979	up
Acp1	0.010730736	2.355880022	down
LOC100041388	0.01076128	2.183360577	up
Cfp	0.01076364	1.58499372	up
Artn	0.010781214	1.536741376	up
Zdhhc13	0.010853731	1.505540013	down
Ralb	0.010855238	1.575715542	up
Trpv4	0.010855238	1.773774862	down
Fasn	0.010858424	1.66486764	up
Anln	0.010886479	1.848033309	down
BC064033	0.010899816	1.728478193	down
Kif16b	0.010904597	1.594723463	down

4833423F13Rik	0.010915371	2.081902742	up
1110001M07Rik	0.010917298	2.313227892	up
Mark3	0.010927745	1.69706285	up
9330156P08Rik	0.010928346	1.611035705	up
Alkbh	0.010984945	1.624966383	up
Zfp653	0.010986622	1.773642659	up
1200008A14Rik	0.010992971	1.567101955	up
Wdr4	0.010999728	1.569469333	down
Phka2	0.01100707	1.790020824	up
B330012G18Rik	0.011012007	1.605123282	up
3110045I18Rik	0.011013804	1.723586798	up
Arpc1b	0.011061336	1.580965877	up
Prom	0.011063349	1.662183762	down
Pot1b	0.011066101	1.578420997	down
Cxcl16	0.011086034	1.836426139	up
Gns	0.011086935	1.522681952	up
Lrsam1	0.011139535	1.504536271	up
Csnk2a1-rs3	0.011209672	2.105276346	up
Lamb2	0.011247079	1.996034622	up
Ccdc77	0.011292923	1.5072577	up
B130019G13Rik	0.011344674	1.657706738	up
C2cd3	0.011358844	1.671829581	up
Unc5h3	0.011372172	1.823118329	down
LOC100046255	0.011413404	1.509611607	down
LOC675572	0.011435394	1.612324119	up
9628654_317_rc	0.011446441	1.686406612	up
C79407	0.011491498	1.526719928	down
BC068157	0.0114932	2.052378893	up
Sdccag1	0.011508698	1.696965218	down
2810402E24Rik	0.011517732	1.576729059	up
LOC100046608	0.011533875	1.877640724	down
Nras	0.011534634	1.556427956	up
Heph	0.011559189	1.967778206	down
8430408J07Rik	0.011616032	1.780914068	up
C230052J16Rik	0.011620862	1.656428814	up
Tspan8	0.011627425	1.740787864	down
Msi1h	0.011630057	1.612006068	up
LOC624784	0.011654343	1.581131816	up
6030416D17Rik	0.011659606	1.865470767	up
9030016H15Rik	0.011662223	2.530107737	up
Dock11	0.011679944	2.097579002	down
Ubr7	0.011711195	1.924065709	down
Gria4	0.011712339	1.538630366	up
Ptprc	0.011712614	1.818335176	up
Ubp1	0.011738852	1.889035821	up
Pex16	0.011750234	1.522709847	up
Zcchc12	0.011750234	1.60396421	down
Cpxm1	0.011757615	1.71546936	up
2900041H08Rik	0.011760511	1.583551884	up
Stard7	0.01179724	1.598079443	down
Gyk	0.01180856	1.568989754	down
Rhot2	0.011814421	1.541172743	down

Zfp276	0.011814421	1.911847115	up
B930093H17Rik	0.011881809	2.131170034	up
Cxcl14	0.011881971	1.616492987	up
Rerg	0.011917487	1.511039853	down
Zbtb26	0.011935135	1.621330738	up
Apbb1	0.011989108	1.633399963	up
Fus	0.01200068	6.871912479	down
Med30	0.012007787	1.651039004	down
Atp13a2	0.012010627	2.187206268	up
Myst4	0.012031172	1.982156515	up
Tmc6	0.012031257	1.52470541	up
Ash2l	0.012040528	1.797225118	up
A230058F20Rik	0.012063143	1.628465414	up
Lsm14b	0.01207102	2.001784086	down
Dtx1	0.012080779	1.650102973	up
Tfrc	0.012111018	1.880373597	down
A430102J17Rik	0.012149001	1.819042444	up
Prcc	0.012168446	1.59511435	up
Lmnb1	0.012203296	3.909193039	down
Ndufa5	0.012214908	1.630812168	down
Taldo1	0.012258549	1.577936173	up
Nudt14	0.012267709	1.560656071	up
Irgq	0.012273548	1.692682266	up
Dmd	0.012335468	2.247594595	down
Eml2	0.012349854	1.567892671	down
Arl2bp	0.012392052	1.914765835	down
Faf1	0.012399633	1.815801978	down
9330177L23Rik	0.012409487	1.593108177	up
D5Wsu152e	0.012420347	1.642475367	up
5330439J01Rik	0.01244428	1.93954742	down
Hoxd10	0.012449063	1.923534751	up
Gata3	0.012506727	1.555933714	down
Rtf1	0.012520847	2.416213274	down
A130001G05Rik	0.012542888	1.965363383	up
A630076E03Rik	0.012573157	1.655741572	up
Ltc4s	0.012589843	1.979646564	up
Wdr40a	0.012628798	1.546823978	up
Snapc4	0.012650384	1.57585752	up
1110003O08Rik	0.012693488	1.83167994	up
Col2a1	0.012693488	1.865817666	down
Mfap4	0.012701844	1.59489429	up
Ufd1l	0.012733715	1.587876201	down
Fibp	0.012751528	1.517603874	up
B930036M14Rik	0.012840894	1.731819034	up
Col11a1	0.012857841	2.09778595	down
EG331392	0.012858728	1.573738456	up
Nrd1	0.012873653	1.569431424	up
Rian	0.012912843	2.022321224	up
Fgfr2	0.012926153	1.819450498	down
Mprip	0.012952995	1.705515742	up
6330417A16Rik	0.012978165	2.263194799	up
LOC100044218	0.012995481	1.523691058	down

mtDNA_ND2	0.012995481	3.769546986	down
6430550H21Rik	0.013001067	1.71196413	up
Snx11	0.013001067	2.044929743	up
H2afv	0.013009328	1.559857607	down
Hbb-b1	0.013027807	7.649670601	up
Igh-6	0.013027807	2.170885563	up
Asph	0.013073362	1.784584761	down
Ttc14	0.013116311	1.560758829	down
Eif5	0.013145986	2.975106716	down
Tnfsf13b	0.013145986	1.688338518	up
Mtvr2	0.013153177	1.532585263	up
Traf1	0.01315827	1.850340962	up
2610024E20Rik	0.013176678	1.594546795	up
A530058L02Rik	0.013177666	1.515235663	up
Hmha1	0.013179728	2.101531982	up
Slc9a3r1	0.013180432	1.859540224	up
Cyp26b1	0.013187064	2.649281502	down
Col23a1	0.01324173	1.54298389	up
LOC100044692	0.013299187	1.716753483	up
Nub1	0.013317319	1.562164903	up
E330009F12Rik	0.013334495	1.571594477	up
2310047C04Rik	0.013387998	1.62315023	up
Gja5	0.013416965	1.517576218	up
2810404I24Rik	0.013423672	1.618174672	up
Slamf9	0.013442528	1.585953236	up
Lrrc45	0.013449911	1.662959099	up
LOC100044395	0.013476202	1.634354711	down
D930050H18Rik	0.013528965	1.533668995	up
Prdm2	0.013548303	1.626281858	up
ARP2	0.013554718	1.637546659	up
Eef1a1	0.013558084	1.946922898	up
Msc	0.013558084	1.678006768	down
Map2k6	0.013568964	1.549019337	down
Angptl4	0.013587164	1.558562636	down
Rlf	0.013608064	2.212370157	down
Pthlh	0.013628989	1.747664928	down
9430012M22Rik	0.013665223	2.129438877	up
Stk11	0.013702997	1.590244532	up
Pdgfa	0.01374491	1.625186443	up
Eif1ad	0.013753292	1.647574782	up
Sox21	0.013756869	1.585513711	down
D930014N22Rik	0.013772138	2.07677412	up
9430008C03Rik	0.01382012	1.500455737	down
Cdk3	0.013884417	1.517178655	up
Tor1a	0.013889653	1.500824809	up
Hbb-b1	0.013909161	7.77823925	up
Parg	0.013909161	1.687676549	down
Tle6	0.013939515	1.771229625	up
Dhcr24	0.013956518	1.919084787	up
Hddc2	0.013964236	1.652547717	down
Lcorl	0.013981664	1.54573679	up
Nfkbid	0.014010639	1.532115459	up

Ccdc55	0.014074417	1.528902292	down
Car13	0.014078152	1.591670752	down
A530089A20Rik	0.014092034	2.047591209	down
Unc5c	0.014135574	1.519138575	down
2700022J23Rik	0.01414173	1.541285753	up
Gpr17	0.014154808	1.712632537	down
Sgcb	0.014166679	1.52116406	down
Efemp1	0.014179253	1.692105413	up
Gins2	0.014184191	1.851901412	down
Zwilch	0.014200673	1.548587799	down
Phkb	0.014203393	1.51730001	up
Acbd4	0.014209136	1.532922506	up
Fubp1	0.014217254	2.243709564	down
Dct	0.014221386	1.577306986	down
2810055F11Rik	0.01422507	1.637764573	down
Dcc	0.014239829	1.609910727	up
Wbp1	0.01427663	1.587239981	down
1700029G01Rik	0.014286649	1.573672772	up
2410006H16Rik	0.014286649	1.615646958	down
Hist1h4f	0.014314462	1.525634289	down
4930570C03Rik	0.014324252	1.598093152	up
Hist2h2aa1	0.014331507	1.562488794	up
Akt1s1	0.014368234	1.919832826	up
Mmd2	0.014379609	1.693962097	down
Vps29	0.014383988	1.745148897	down
LOC381230	0.014404518	1.548574686	up
mt-Nd5	0.014421569	3.367965698	up
Polk	0.014422618	1.579787493	down
4930545L23Rik	0.014455425	1.59867847	down
Ubxn8	0.014528827	1.529180646	down
Utp23	0.014547062	1.532169223	down
Crebzf	0.014557002	1.57626915	down
Apobec3	0.014573689	1.500709653	down
2610202E01Rik	0.01457393	1.68234992	up
St7	0.014589873	1.763204098	up
Rars	0.014625328	1.694197536	down
2310067B10Rik	0.014727488	1.686938167	up
D830014B20Rik	0.014750568	2.099839687	down
Adam15	0.014768669	1.615787506	up
Arrdc4	0.014774913	2.774963856	up
D930007M19Rik	0.014786349	1.95210731	down
Zcchc7	0.014799102	1.628791809	up
C430005L07Rik	0.014812215	1.60780263	down
scl0001464.1_61	0.014812215	1.60696435	up
Map3k7ip1	0.014823621	1.727927208	down
4833414E09Rik	0.014855902	3.011304617	up
2610104F20Rik	0.014893767	1.694723845	up
Nup133	0.014899062	1.941984773	up
Lrrc16b	0.014913377	1.91657424	up
LOC100044275	0.014923816	1.643465161	up
Mta2	0.014930181	1.63879776	up
Elf1	0.014981337	1.564953923	down

4930503L19Rik	0.015053246	1.898484469	down
Nagk	0.015056096	1.611242652	up
Mif	0.015058579	1.607923269	down
Arhgap8	0.015098507	1.659457803	up
C130048M12Rik	0.015098507	1.698494554	down
A330089M16Rik	0.015143639	1.646604061	up
Hsd3b7	0.015161737	1.660743237	up
Tmem132a	0.015164442	1.537937641	up
LOC638275	0.015200235	1.821522355	down
Ttc30b	0.015223199	1.529625416	down
Jmjd1a	0.015234897	2.485238075	down
1700021K19Rik	0.015259022	1.645505667	up
D430005F24Rik	0.015279185	1.732679248	up
Plxna3	0.015290167	1.502586842	down
Ttyh1	0.015290167	2.144072533	down
A530020H22Rik	0.015300924	1.763928652	up
Jam2	0.015325007	1.505161285	down
Fn1	0.015341385	1.853502989	down
LOC547380	0.015368923	1.820029497	up
LOC100048105	0.015415511	1.862961054	up
Wnt5a	0.015419887	1.516037941	down
Lrrc50	0.015467605	1.622580171	up
2410012M04Rik	0.015473281	1.502745986	up
Tap2	0.015485013	1.890290976	up
Tbxas1	0.015493888	1.623475432	up
EG620119	0.015512404	2.267612696	down
Eif2s2	0.015512404	2.120897293	down
LOC674004	0.015542697	2.86631608	down
Ccnc	0.015571334	1.713863611	down
Pou6f1	0.015609437	1.708609581	up
2810405F04Rik	0.015615013	2.58091116	down
4932417I16Rik	0.015626036	1.665560842	down
Plekho2	0.015634831	1.590256214	up
Zfp414	0.01563769	1.552692771	up
Klc1	0.015643211	1.50708282	down
Cald1	0.015649566	1.575563431	down
Polr1e	0.015656458	1.607215285	up
Nip7	0.015658187	1.918694973	down
Nrxn2	0.015658187	2.022223234	up
Nhlrc2	0.015691295	2.091707707	down
LOC100046594	0.015709708	2.62715888	down
Ppapdc1b	0.015752522	1.58837533	down
Gp1bb	0.015761692	1.553402424	up
B930083E23Rik	0.015785834	2.655101299	up
Pnpla7	0.015787069	1.557976604	up
C730010J01Rik	0.015796756	1.575244904	up
B930001P03Rik	0.015804652	2.427062988	up
Pax1	0.015903296	2.354618549	up
6430407L02Rik	0.015932487	1.752454042	up
Ppnr	0.015947174	2.886119366	up
2610301N02Rik	0.015954686	1.844610453	down
E030026E10Rik	0.015954686	1.524294138	up

Trib2	0.015954686	1.596457601	up
Abca7	0.015963128	1.73140645	up
Mtmr4	0.015986029	2.094404936	up
Mmp23	0.016031815	1.60755837	up
scl0003040.1_5	0.016046926	1.56754911	down
A130076G11Rik	0.016089123	1.787539244	up
5430404G13Rik	0.016122658	2.256258965	up
Arid1b	0.01616071	1.501429915	up
LOC380925	0.016233882	1.875408649	up
Brap	0.01624731	1.574795961	up
2610044O15Rik	0.016247973	1.609202743	up
Rad51l3	0.016287977	1.614845514	up
A430103D13Rik	0.016318915	1.575238109	up
Rmnd5b	0.016318915	1.510534406	down
Stmn4	0.016323222	1.638828397	down
Slc39a3	0.016325425	1.562371969	up
Slc9a3r2	0.016325425	1.696235776	up
Mcf2l	0.016343722	2.186741591	up
Nfix	0.016355747	1.575346112	down
Odz4	0.016355747	1.744203448	up
Zfp364	0.016362136	1.5711689	up
Hsd3b7	0.016396118	1.597201347	up
Bmp1	0.016406797	1.60881567	up
Il4ra	0.016408974	1.881296396	up
Pi4k2b	0.016434064	1.550752401	down
2900009C24Rik	0.016480325	1.56754005	up
2810474O19Rik	0.016515521	1.764845729	up
Taf2	0.016587799	2.381062269	down
D930050A07Rik	0.016611183	1.571659565	up
Hist3h2ba	0.016619289	1.522813439	up
C87436	0.016638862	1.850239635	down
A630043I21Rik	0.016653638	1.529606819	up
Hprt1	0.016661359	1.646520376	down
5830431N17Rik	0.016698534	1.77746594	down
Chst3	0.016758584	1.71955502	down
BC022687	0.016782846	1.665683985	up
Grk5	0.016837573	1.684292436	down
Lin54	0.016851082	1.594561577	down
1500003O22Rik	0.01685659	1.575522661	up
Nomo1	0.016863186	1.697021246	up
Nfatc1	0.016887963	1.625146389	down
Zfp36l1	0.016965367	2.099901676	down
Snx3	0.016969873	1.603039861	down
Hoxc13	0.017006882	2.708408594	up
Pdzrn3	0.017043423	1.666963577	up
Mamdc4	0.017105119	1.536769629	up
1810046J19Rik	0.017108837	1.519720912	up
Enpp2	0.017148748	1.58511591	down
Evi1	0.017154278	2.067652702	down
3830431G21Rik	0.017192848	1.735956073	up
Hspa4	0.01729572	1.625148892	up
D330040H18Rik	0.017307824	1.860665917	up

Ddx24	0.017328613	1.511216998	up
Erg	0.017328613	1.973852038	down
Pilra	0.017328613	1.545988321	up
Zmiz2	0.017328613	1.840798736	up
Gas7	0.017362045	1.776484132	up
A130020K16Rik	0.017393217	2.07652092	up
Ercc1	0.017423546	1.502855539	up
Hace1	0.017443497	1.551439762	down
2900056M20Rik	0.017461967	1.938312531	up
6530415H11Rik	0.017473679	1.649091125	up
2410022L05Rik	0.017544065	1.528789282	up
Mll3	0.017591413	1.502576351	up
Ptpn1	0.017605349	1.964437604	up
9030223K07Rik	0.01763395	1.717803478	up
Pigb	0.017642314	1.893906355	down
Rps6ka4	0.01769172	1.525422692	up
Smc5l1	0.01769172	1.52446115	up
9430023L20Rik	0.017704897	1.620031238	up
Wdr79	0.017767321	1.513698936	up
4932433N03Rik	0.017805893	1.658770084	up
Wdr86	0.0178135	1.51345706	up
LOC100048812	0.017882092	1.629825354	up
D330050I23Rik	0.017897118	1.730247259	down
Rogdi	0.017897118	1.571779966	up
Fmn2	0.017901592	1.568500757	down
Alg11	0.017913392	1.533117533	down
1500015O10Rik	0.017942723	1.779098392	down
Utx	0.017995914	1.50602603	down
Mar-11	0.018000988	1.643353343	up
Ticam2	0.018007113	1.565997005	up
Cchcr1	0.018030645	1.518537641	up
2310014H01Rik	0.01808024	1.539948702	up
Ccdc66	0.01809505	1.524603486	down
Mt3	0.018117914	1.519596338	down
Ascc2	0.018134113	1.649891019	down
Pfdn5	0.018134113	1.636819124	down
Rhob	0.018134113	1.795479775	up
Nkx2-2	0.018138211	1.520688176	up
Ankrd12	0.018188709	1.690204263	up
Sh3rf1	0.0182072	1.549773216	up
Sgms1	0.01821428	1.556291699	down
Rab33b	0.018244986	1.561764956	up
Zfp160	0.018254852	1.648348689	up
LOC100039888	0.018267162	1.818234801	up
End3	0.018367505	1.620908618	up
9530083O12Rik	0.018480251	1.879650474	up
Ctsb	0.018484259	1.804387212	up
2700069A02Rik	0.018492756	1.649939656	down
D930031I08Rik	0.018492756	1.604698181	up
4930405A21Rik	0.018524321	1.656266213	down
Aplp1	0.018587796	1.950808525	up
Heph	0.018628702	1.680292487	down

Syt5	0.018628702	1.609662056	up
Shox2	0.018677397	2.139605284	up
Casp6	0.018952517	1.554134369	down
St8sia2	0.019010162	1.841214657	up
E130112N23Rik	0.019019207	1.614276886	up
Fanca	0.019019207	1.565289617	up
8430438D04Rik	0.019031048	1.877628923	up
Hbb-b2	0.019067744	7.270130158	up
D130019H05Rik	0.019103719	1.624179483	down
Gtf2f1	0.019148465	1.803111672	up
Pcdhb6	0.019173428	1.576027632	up
Dab2	0.019258801	1.610951066	up
LOC238836	0.019271161	2.153017759	down
EG665685	0.01930489	1.823449254	down
Fusip1	0.01930489	1.946251035	down
Arfgef1	0.019311925	2.161479712	down
Ddx26	0.019317096	1.613991737	up
BC003498	0.0193453	1.520325422	up
Amigo2	0.019347727	1.50480473	up
Foxf1a	0.019437868	1.569296002	down
Adap2	0.019468719	1.609261155	up
8430436C05Rik	0.019525684	1.844633222	up
Pcyt1a	0.019537203	1.650879741	up
Hbb-b1	0.01954609	8.318092346	up
Cfl1	0.019548684	1.53356874	up
Pou3f1	0.019551167	2.063205719	up
Ints1	0.019591827	1.566596627	up
Ddx3y	0.019655107	3.246217966	up
9030607L20Rik	0.019720452	1.899833679	up
Exosc6	0.019747123	2.058234215	up
Pot1b	0.019876078	2.112498522	down
Syce1	0.019920012	1.86384666	up
2610304O13Rik	0.019936338	2.159354925	up
Lmo2	0.019993877	2.961601973	up
Hmgb1	0.02002883	2.085407734	down
Dnm3os	0.020042723	2.063773394	down
Tssc8	0.02005334	3.236780405	up
4833420G11Rik	0.020088904	1.604034543	down
C430004E15Rik	0.020146139	1.56531024	up
Ncl	0.020166822	1.689600945	up
9430022A07Rik	0.020187717	1.68251133	up
C230067O06Rik	0.020236097	1.533300757	down
Col1a1	0.020236097	1.851671577	up
A630076E03Rik	0.020296967	2.192493677	up
Tnni2	0.020329716	1.586446285	up
2410018I08Rik	0.020339169	1.556555748	up
D630047N04Rik	0.020410717	1.625984788	up
Neurl	0.020420333	1.822006226	up
Col25a1	0.020420678	1.570342064	down
2700024H10Rik	0.020474836	1.514556646	up
Plekha2	0.020497326	2.480962277	up
Sox11	0.020497326	1.644564867	up

EG625835	0.020504223	1.709165573	up
Ntrk2	0.020534083	1.564655185	up
Sox8	0.020557594	1.502229095	down
LOC236604	0.020571385	1.52287209	down
4833406M21Rik	0.020616537	1.994479418	up
C920027I18Rik	0.020624859	1.566333056	down
B930069K15Rik	0.020664822	1.807218313	up
Top3b	0.020697853	1.608972907	up
Gprasp1	0.020699658	1.506540656	down
Calm2	0.020710791	1.700144887	up
Copg2	0.020787496	1.639634132	down
1200016E24Rik	0.020792129	1.565907121	up
Ybx3	0.020792922	1.588572502	up
Arid3b	0.020807253	1.576095343	up
9530007E02Rik	0.020834299	1.500366688	up
Nol3	0.020856412	1.673335433	up
Sec22l3	0.020885751	1.526977897	up
B230343A10Rik	0.020981461	1.780857801	up
Pawr	0.021021903	1.504055858	down
0610031J06Rik	0.021027813	1.50747323	up
Gats	0.021061473	1.619213224	up
A530083M17Rik	0.0211579	3.08627677	up
Hk3	0.021170015	1.712053299	up
Hbb-b1	0.021201024	6.973231792	up
A530030B07Rik	0.021343874	1.752098799	up
Cdk5	0.021353282	1.550286531	up
Prpf19	0.021391664	1.56695044	down
Zc3h15	0.02140856	1.564205051	up
Nfatc1	0.02144479	1.971214891	down
Mpp3	0.021554269	1.537477493	up
Rnf3	0.021592744	1.587883592	up
Atg3	0.021593135	1.614169955	down
Ift57	0.021595502	1.671165586	up
Kcnj2	0.021619201	2.289093971	down
4921514E18Rik	0.021643112	1.5726825	down
Lin7c	0.02166128	1.770914078	up
LOC637711	0.021746695	1.544100761	down
Folr1	0.021769602	1.605981231	down
Rala	0.021808939	1.733900785	down
Donson	0.021835454	1.576853991	down
D1Ertd396e	0.021948675	1.584858179	up
Il6ra	0.021968354	1.728919029	up
9930033D15Rik	0.021988338	1.518664122	up
Tnnc1	0.022033962	1.757799506	up
3632413B07Rik	0.022292096	1.672812343	up
2810008D09Rik	0.022395229	1.630763769	up
Tmbim6	0.02252706	1.648805022	up
Anapc7	0.022578396	1.560350776	up
Dusp5	0.022610087	1.514411569	up
4632415K11Rik	0.022809174	1.523635864	up
Tppp3	0.022973267	1.515937686	up
LOC100046608	0.022979464	1.908893704	down

A730058G16Rik	0.022985704	1.822143912	up
2810410A03Rik	0.023044504	1.518968225	up
H3f3b	0.023090782	1.533421636	up
Actn2	0.023095578	1.730810285	up
Arglu1	0.023095578	1.500149727	up
BC026590	0.023128154	1.706980825	up
Ints12	0.023156676	1.654516935	down
Tcf20	0.023161488	1.50092411	down
Cdk7	0.023165254	1.786380172	up
Narg3	0.023167392	1.578389168	up
Trp53inp2	0.023167392	1.553473353	up
Sipa1	0.023201779	1.59102416	up
Meis3	0.023320233	1.669393897	up
Il17rc	0.023322796	1.622000933	up
Serinc5	0.023345973	1.672164798	up
C430048L16Rik	0.023377892	1.643749237	up
Clec7a	0.023394242	1.532199383	up
Nfkb1	0.023435246	1.889028788	up
Ptprz1	0.023490856	2.008227348	down
Rragb	0.023499509	1.565858841	down
3110056O03Rik	0.023512038	1.504541755	up
Gm784	0.023539327	1.975931406	down
Seh1l	0.023612496	1.78618741	down
Fat3	0.023772512	1.660501838	up
A330083M20Rik	0.023837352	1.585550785	up
LOC664956	0.023872476	1.618780375	up
LOC669660	0.023903126	1.801057577	down
9430091N11Rik	0.023923457	3.282517672	up
Neurog2	0.023983184	3.451078653	down
D030073C20Rik	0.024037	1.635189056	up
Ttc13	0.024050135	1.60121882	up
Wnt9a	0.024064122	1.56685245	up
scl0001860.1_2694	0.024070205	1.572082043	up
Nfic	0.024165949	1.547176957	up
C030044B11Rik	0.024186941	1.757001281	down
Myl9	0.024218453	1.544612289	down
Rbmx	0.024218453	1.797032952	down
Tbx18	0.024300017	1.522234917	up
D230017C05Rik	0.024374018	2.547896147	up
Hexim1	0.024374289	1.911100388	up
Limd2	0.024429301	1.720597744	up
Nelf	0.024429301	1.501473069	up
Ncstn	0.024458835	1.723075628	down
Lsamp	0.024467398	1.558461189	down
Ube2e3	0.024551753	2.235800743	up
Gps2	0.024673687	1.501157522	up
C630030A18Rik	0.024702463	1.8217026	up
Myh3	0.024714334	1.999429107	up
Tmem166	0.024791537	1.628471971	down
Pabpc4	0.024886003	1.559286594	down
5430431D22Rik	0.024960838	1.637160063	up
9630005C17Rik	0.024963278	1.675758958	up

Gpr107	0.025039872	1.63171351	up
Ube2g1	0.025054282	1.639479637	down
2410004N09Rik	0.025077798	1.639373064	up
6430403C09Rik	0.025133722	1.556881785	up
Arhgap24	0.025214126	1.706454039	down
Rbm42	0.025283694	1.538809776	up
2610028L16Rik	0.025288999	2.36195302	up
0610006I08Rik	0.025402693	1.700408459	down
A430103B12Rik	0.025592184	2.176637173	up
Ankrd34a	0.025592184	1.72562778	down
Dusp26	0.025719157	2.14577961	up
D830031G23Rik	0.025781283	1.941155791	up
Gpm6a	0.025784677	2.084709644	down
Nfic	0.025964795	1.620805621	up
mtDNA_COXI	0.026019203	2.043129921	down
Ankra2	0.026019333	1.632736206	down
P4ha2	0.026029255	1.98194623	down
4832408C21Rik	0.026202362	3.687877655	up
Trpv2	0.02621866	1.634125352	up
6720470G16Rik	0.026288547	2.175667286	up
EG333830	0.026339775	1.512492538	up
Trio	0.026358793	1.6330235	down
Trps1	0.026364725	2.25335002	down
Arl5b	0.026375158	1.855834484	up
Snip1	0.026404629	1.606954336	down
Frk	0.026437875	1.751980543	up
Tnfrsf12a	0.026474221	1.520322204	down
Bcor	0.026485315	1.556888342	down
Ncam1	0.026524995	1.675677061	up
Tbl3	0.026624341	1.593612194	up
D030074E01Rik	0.026653454	1.585466862	down
LOC433464	0.026796611	1.507876635	down
Fnip1	0.026804889	1.518992543	down
Sh3bgrl	0.026836412	2.048965693	down
Fam20b	0.027013743	1.650509834	down
E130014J05Rik	0.027047353	1.601880193	up
MALT-1	0.027127458	1.655619621	up
EG277333	0.027219711	1.652448535	up
Slit2	0.027277453	2.072291613	up
Cldn4	0.027371962	1.552918911	down
A830006J06Rik	0.02738763	2.073411703	up
Rnd2	0.027398052	2.877049685	down
Kcnma1	0.027493293	2.169963598	up
Cyfip2	0.027529674	1.96624136	up
Cap1	0.027537964	1.58344388	up
6030436C20Rik	0.027550042	1.823342323	up
8030466E21Rik	0.027557021	1.747817874	up
A530076E23Rik	0.027641369	1.635259271	up
Diablo	0.027699878	1.572608233	up
Rev3l	0.027733464	1.678638935	down
LOC100045054	0.027746508	1.642021298	up
Atp6v0c	0.027829561	1.907900691	down

Hspa8	0.027860519	1.590014458	up
Panx3	0.02788059	2.619599581	down
Slit3	0.02788059	2.003383637	up
A430073A17Rik	0.02815743	1.623515129	up
Tmem63b	0.028199595	1.592990995	up
8030479D07Rik	0.028224614	2.156710148	up
1810026B05Rik	0.028257683	2.077821255	up
LOC238943	0.028294431	1.941467166	down
1110033K02Rik	0.028332705	1.563651204	up
Ankrd10	0.028453095	1.543887615	up
Dlst	0.028590931	1.62280333	up
Usp47	0.028646117	1.575356126	down
Ank3	0.028652705	1.608907938	up
Pak3	0.028678091	1.949670911	down
Tbx5	0.028805319	1.863815188	up
Tmco3	0.028866852	1.513917327	down
EG433923	0.028954901	1.783436179	down
Fto	0.028977204	1.872379541	up
Star	0.029008696	1.60982573	up
Mkln1	0.02902166	1.524707913	up
Cndp2	0.029028013	1.714619279	up
Ncdn	0.029063145	1.599232435	up
Aebp2	0.029099127	1.500701666	down
F730003H07Rik	0.029329818	1.620857835	up
2900040J22Rik	0.029390939	1.792720914	up
A630098G22Rik	0.029500375	1.568632245	down
6720464F23Rik	0.029634349	2.279848814	up
Nasp	0.029696375	1.817962408	down
Mll3	0.029732732	1.535362482	up
Mef2c	0.029764548	1.61142087	down
Dct	0.029809175	1.660903931	down
AW228700	0.029932546	1.543149471	up
2410025L10Rik	0.029970994	1.600186348	up
C330006P03Rik	0.030145994	1.623881459	up
Csf1	0.030208422	1.510946751	up
LOC100047888	0.030216232	1.571417809	up
Thbs2	0.030375907	1.530437708	down
Rbm3	0.030448498	1.754942536	down
Atrnl1	0.030655773	1.593916178	up
Ncoa6	0.030689774	1.53946197	down
1110033F14Rik	0.030838693	1.528321624	up
N6amt2	0.030867657	1.640986919	up
Robo2	0.031276278	1.660531163	up
2810427C15Rik	0.031342957	2.818995953	up
2510006D16Rik	0.031394586	1.582388759	up
Cntnap2	0.031414226	2.22788167	down
Gria1	0.031559862	1.693127275	up
C230037E05Rik	0.031613197	2.078089237	up
Birc1cl	0.031911936	2.208615303	up
Setd1b	0.03197594	1.561620235	up
A130022J21Rik	0.032255564	1.541298509	up
1810017N16Rik	0.032534637	1.51070416	up

A630076G18Rik	0.032597665	1.596131206	up
0610030E20Rik	0.032646209	1.60135591	up
Stub1	0.032783683	1.637759924	up
Abca8a	0.032802887	1.678803086	down
3110006E14Rik	0.032907706	2.02323246	up
Tgfbrap1	0.032907706	1.606859326	up
E130202F10Rik	0.032930646	2.224581003	up
E030040P03Rik	0.032961104	2.07277298	up
6720407G21Rik	0.033149082	1.680956841	up
E230008O15Rik	0.033264656	1.666207671	up
Serpini1	0.033286374	1.65614605	down
Sep-07	0.033388555	1.666994572	up
2610005M20Rik	0.033453487	1.878376484	up
3110073H01Rik	0.03346042	1.632067561	up
1810009N02Rik	0.033490706	2.322052956	down
Map4k3	0.033566035	1.625558496	down
Pfdn2	0.033582468	1.515936256	down
B230340J04Rik	0.033624612	1.618609786	up
Cpt1b	0.033880871	1.549491048	up
D130032J17Rik	0.034177993	2.362062454	up
5730437P09Rik	0.034241017	1.573449731	up
Neo1	0.034292344	1.652311444	down
Errfi1	0.034536801	1.543256164	down
Ifrd1	0.034575649	1.517557621	up
Psmc1	0.034588281	3.174249888	down
A930005G04Rik	0.034599535	1.684175611	up
2810405J23Rik	0.034669712	1.725699186	up
Gadd45g	0.034689002	1.811520219	down
Sema6d	0.034857102	1.505709171	down
Egln1	0.034872085	1.570039034	down
Prrx1	0.034901623	1.689611316	up
LOC100047082	0.034981057	1.764771342	up
Ptpn11	0.035001047	1.524289727	up
Tnfrsf25	0.035066426	1.600338817	up
LOC100047619	0.035223246	1.56867981	up
6330439K17Rik	0.035393234	1.524117827	up
Mylk	0.035394479	1.881766915	up
5830474E16Rik	0.035454564	1.558140993	up
Rplp0	0.035475109	1.567043781	up
Slc11a2	0.035735555	1.519895315	up
Ube2g1	0.03584398	1.518849611	down
Barx2	0.035974573	1.63368845	down
Rnf19a	0.036178503	1.57252264	down
A130038M19Rik	0.036188681	1.615169048	up
Nol5	0.036427368	2.087093353	down
Irf3	0.036456235	1.624799252	up
Ccnb1	0.036632683	1.616141439	down
Gs2na-pending	0.036729287	1.814650774	up
Preb	0.036734916	1.568970919	up
9430047F21Rik	0.037352588	2.104152918	up
Metrn	0.037425973	1.586075902	down
Dhx9	0.037614767	1.612222552	down

2900072G11Rik	0.037677776	1.634542227	up
A130082N24Rik	0.037837055	1.627068996	down
LOC100043796	0.038071685	1.638041616	down
A630006M12Rik	0.03809797	1.618706346	up
Isoc2a	0.038104817	3.079586029	down
Scamp5	0.038104817	1.893077731	up
LOC433886	0.038219925	1.539492965	up
Camk2n1	0.038539704	1.784862399	up
4833431D13Rik	0.038543578	1.724560738	up
5430416B10Rik	0.038580053	1.578135252	up
Foxp4	0.038634628	1.508641601	up
4832406H04Rik	0.038729668	1.837097764	up
Gne	0.038805187	1.598900795	up
E130001M03Rik	0.038833767	1.500621676	up
Olig1	0.038884744	2.554029942	down
Cpsf6	0.039143845	1.602885008	up
2700017A04Rik	0.039159138	1.956477404	up
Lman2	0.03927388	1.697033286	up
T	0.039372377	1.527101755	down
Robo2	0.039584518	1.523898602	up
2700023E23Rik	0.039628856	1.67825532	down
E030019B13Rik	0.039729506	2.561062813	up
4930549G23Rik	0.039787475	1.924414992	down
AA410130	0.039826572	2.39052105	up
Bmi1	0.039895792	1.615434647	down
Vmn2r-ps14	0.040216472	1.522837281	up
mtDNA_ND1	0.040286411	2.364932776	down
D930044I17Rik	0.040380888	2.030469656	up
6430519N07Rik	0.040653825	2.722062349	up
Gjb6	0.040720016	1.630724073	down
Cep70	0.040905979	1.532244563	down
LOC674706	0.040905979	1.763733387	down
Kif21a	0.040952981	2.176335573	down
Prss12	0.040958647	1.692567229	up
Fbxo9	0.041259948	1.525456071	down
C130002K18Rik	0.041261639	1.730988503	up
9430010O03Rik	0.041395664	1.658296704	up
9430076C15Rik	0.041396719	1.569241166	up
A130086G11Rik	0.041399073	1.527243018	up
Cyb561d1	0.041399073	1.718616605	up
Robo1	0.041551508	1.563209176	down
3110045A19Rik	0.041559041	1.518151641	up
Copb1	0.041605596	1.720616341	up
Akap9	0.042103283	1.569421291	up
2610008J04Rik	0.042120453	1.675324678	up
2310061A09Rik	0.042130537	1.69962883	up
Kif21b	0.042145066	1.962554336	up
Znhit3	0.042183068	1.626163244	down
C530050I23Rik	0.042409398	1.620764375	up
Pik3r2	0.042504042	1.899691224	up
D230020C06Rik	0.042762451	1.946179628	up
Antxr1	0.042907596	1.62461102	up

6720477C19Rik	0.043008782	2.319058657	up
Polr1a	0.043021828	1.573827148	up
Peg10	0.043026082	1.547018647	down
Zfp330	0.043126062	1.561439037	down
Rgmb	0.043142051	1.638943791	down
Gga1	0.04315066	1.553385496	up
Crkl	0.043219473	1.66804862	down
Hsph1	0.043262195	1.553467989	up
Tmeff1	0.043439072	1.516194105	down
Cox5b	0.043531373	1.542535543	down
Cplx2	0.043664724	1.727807164	down
Atp5c1	0.043669101	1.586648583	down
Klf6	0.04372691	1.667094708	up
C130092E12	0.043761618	2.08492446	down
Npy	0.043897752	1.62522614	up
Lhx9	0.043975215	1.511396647	down
Sfmbt2	0.044018816	1.556919575	up
Ube2j1	0.044270452	1.680078983	up
9530009M10Rik	0.044360209	1.612905264	up
Ust	0.044363979	1.561433435	up
2510009E07Rik	0.044492364	1.914743543	down
Dhx9	0.044631109	1.802828074	down
2210410E06Rik	0.044723138	1.522418141	down
Mitd1	0.044723138	1.593602657	down
E330020G21Rik	0.044845425	2.537300825	up
Ankmy2	0.045014925	1.558947444	up
B130066H02Rik	0.045014925	1.963836074	up
9930013M16Rik	0.045237709	1.518592596	up
D630050H08Rik	0.045465723	1.550317645	up
Ahsa2	0.045551706	1.585852623	up
Hoxb2	0.045596853	1.554855585	up
E230012J19Rik	0.045630492	2.144312143	up
E030017D19Rik	0.045635484	1.569161058	up
Dnajc2	0.045722172	1.603770137	down
Nktr	0.045890831	3.003704071	up
5033413D16Rik	0.045922305	2.17121315	down
Nid1	0.045947753	1.646505833	up
Atad1	0.046240527	1.623005867	down
Purb	0.046308126	1.584069729	up
Ky	0.046456207	1.654770613	up
D130062J21Rik	0.04669074	1.523625374	up
Hoxd12	0.046815649	1.629961252	up
Gria2	0.04682434	1.619322181	down
Atn1	0.046924945	1.50362432	up
4930588G05Rik	0.047056787	1.631296635	up
9530019H20Rik	0.047157411	1.544849038	up
Tbx4	0.047717989	1.557803273	up
Ubg	0.047785584	1.672635078	up
Samd8	0.04783364	1.525051951	up
A430090L17Rik	0.047857635	2.162659884	up
Dyrk3	0.048358429	1.503773332	down
LOC100048105	0.048589252	1.550920487	up

H2-D1	0.048720013	1.83630383	up
Olig2	0.048772559	1.505360365	up
6230415M23Rik	0.048825152	1.565640688	up
Ncdn	0.048962172	1.638199449	up
LOC100048020	0.049430102	1.550496936	up
St8sia4	0.049651053	2.122776032	up
Tns3	0.049662456	1.662731886	down
1110037P13Rik	0.04981003	1.619341135	up
AI504432	0.049942296	1.520092845	down

Table 2 (5 pages)

No.	Probe	Expression Pattern Observed
1	*Cytl1*	1. Fore Limb
		2. Hind Limb
		3. Snout (Follicles of Vibrissae)
		4. Basioccipital Bone
		5. Ganglia
		6. Nasal Cavity
		7. Lungs
		8. Neural Tissue
		9. Brain
		10. Eye
		11. Gut
		12. Liver
2	*Prelp*	1. Tail Cartilage
		2. Mid, Hind Brain
		3. Vertebra Cartilage
		4. Lungs
		5. Fore Limb
		6. Hind Limb
		7. Humerus/Femur Primordia
		8. Rib Cartilage
		9. Neural Tube
		10. Meckel's Cartilage
		11. Vertebral Disc
		12. Surrounding Vertebrae
		13. Notochord
3	*3110079O15Rik*	1. Basioccipital Bone (Clivus) Cartilage
		2. Fore Limb Humerus Cartilage
		3. Vertebra Cartilage
		4. Rib Cartilage
		5. Hind Limb Femur Cartilage
		6. Eye
		7. Notochord
4	*Mia1*	1. Neural Tissue
		2. Ganglia
		3. Mid, Fore, Hind Brain
		4. Lungs
		5. Rib Cartilage
		6. Gut
		7. Follicles of Vibrissae
5	*Hapln1*	1. Basioccipital Bone (Clivus) Cartilage
		2. Nasal Cartilage
		3. Vertebra Cartilage
		4. Humerus
		5. Meckel's Cartilage
		6. Hyoid Bone
		7. Temporal Bone
		8. Ribs
		9. Femur

		10. Fore, Hind Limb
		11. Fore Brain
		12. Neural Tube
		13. Notochord
6	*Hbb-bh1*	1. Liver
		2. Neural Tissue
		3. Genitals
		4. Tongue
		5. Hind Limb
		6. Ganglia
		7. Gut
		8. Brain
		9. Fore Limb
		10. Eye
7	*Cbln1*	1. Neural Tissue
		2. Dorsal Root Ganglia
		3. Nasal Cavity
		4. Tongue
		5. Gonads
		6. Genitals
		7. Lungs
		8. Liver
		9. Gut
8	*Ms4a6d*	1. Non-localized
		2. Clavicle
		3. Brain
		4. Gut
		5. Neural Tissue
		6. Ganglia
		7. Eye
9	*Mpeg1*	1. Interdigital
		2. Non-specific
		3. Liver
10	*Csf1r*	1. Non-localized
		2. Fore Brain
		3. Interdigital
		4. Genitals
		5. Liver
11	*Tyrobp*	1. Non-localized
		2. Clavicle
12	*Col10a1*	1. Eye
		2. Fore, Hind Limb
		3. Liver
		4. Gut
		5. Humerus
		6. Fore, Hind Brain
		7. Ribs
		8. Gonads
		9. Genitals

		10. Lungs
		11. Heart
		12. Ganglia
		13. Fore, Hind Limb Bones
13	*St18*	1. Fore, Mid, Hind Brain
		2. Dorsal Root Ganglia
		3. Upper Palate
		4. Neural Tissue
		5. Trigeminal Ganglion
14	*Shox2*	1. Upper Palate
		2. Mid, Hind Brain
		3. Neural Tissue
		4. Vestibulocochlear Ganglion
		5. Trigeminal Ganglion
		6. Dorsal Root Ganglion
		7. Genitals
		8. Fore, Hind Limb
		9. Inter-digital Region
		10. Eye
		11. Nasal Cavity
15	*Nfib*	1. Fore, Mid, Hind Brain
		2. Genitals
		3. Neural Tissue
		4. Lungs
		5. Upper Palate
16	*Hoxb9*	1. Neural Tissue
		2. Limbs
		3. Widespread
17	*Nfatc1*	1. Fore, Mid, Hind Brain
		2. Neural Tissue
		3. Genitals
18	*E2f6*	1. Brain
		2. Vertebrae
		3. Tongue
		4. Gut
		5. Neural Tissue
		6. Liver
		7. Lungs
		8. Genitals
19	*Pbx3*	1. Neural Tissue
		2. Brain
		3. Ganglia
		4. Gut
		5. Nasal Cavity
		6. Tongue
		7. Limbs
		8. Forelimb Bud
20	*Sox6*	1. Temporal Bone
		2. Brain

		3. Genitals
		4. Lungs
		5. Humerus
		6. Femur
		7. Fore Limb
		8. Nasal Cavity
		9. Vertebrae
		10. Ribs
		11. Dorsal Root Ganglia
21	*Sox5*	1. Nasal Cartilage
		2. Vertebra Cartilage
		3. Rib Cartilage
		4. Tail Cartilage
		5. Meckel's/Tooth Cartilage
		6. Ganglia
		7. Gut
		8. Fore, Mid, Hind Brain
		9. Follicles of Vibrissae
		10. Fore, Hind Limb
		11. Lungs
		12. Neural Tube (Transverse)
22	*Runx1*	1. Fore, Mid, Hind Brain
		2. Lungs
		3. Dorsal Root Ganglia
		4. Clavicle
		5. Neural Tissue
		6. Gut
		7. Tongue
		8. Liver
		9. Nasal Region
		10. Eye
23	*Tle1*	1. Brain
		2. Trigeminal Ganglion
		3. Neural Tissue
		4. Limbs (Surrounding Cartilage)
		5. Eye
		6. Tail (Surrounding Cartilage)
		7. Lungs
		8. Follicles of Vibrissae
		9. Dorsal Root Ganglion
24	Matn4	1. Hind Limb
		2. Meckel's Cartilage
		3. Ribs
		4. Vertebrae
		5. Femur
		6. Temporal Bone
		7. Lungs
		8. Fore Brain
25	Tsc22d1	1. Hind Limb

		2. Fore Limb
		3. Brain
		4. Neural Tissue
		5. Lungs
		6. Cranio-facial Region
		7. Follicles of Vibrissae
		8. Dorsal Root Ganglia
		9. Trigeminal Ganglion

Table S3 (2 pages)

Gene	Chr	Motif Start	Motif End	Luc insert	Insert size (bp)	Primer (Forward)
[illegible]	2	[illegible]<br>[illegible]	[illegible]<br>[illegible]	GTTCTCGGGGCATCCCCTCCCTCCCCACGTCCTACATAACGGGCCATCTGTTCTGGGGCTGAATAAACAAGAGACGGGAAG<br>CAGCGGCCCCCTGCCCCCCAGCCCCAGTACTGGACAATGCGGCTTTCTCCAAGAGGACGCCACCCGGGCGCCTATGAGCAC<br>GCTCGTCTGAACTCCCCTCCCTCTGCCAGCATAGCAATAGCCACAAACCGACCCCGTGAAAACC	226	GTTCTCGGGGCATCC
[illegible]	7	[illegible]	[illegible]	GAGACGCAGATAGGCAGGCTGGACTATCTGATTCCACCTTCAAGTCGTTTTAGGTACCTGGACCCTCCAGAGACCAAGCTAG<br>AGGAGCCAGTATAGAAAACCACCCTGGAATTTCCTGTGCTGAGGTCTGAGAACAAGGCAGTTCTTTGATTAGCAGCCACAG<br>CCAGGGTTCCCAGGTACTGAGCAAACACTCAACGTCTGGCCTCAGGCTAGGCACTGTCCCACCTGGCTCACTGTGCACATT<br>CACAGGCTACTTCCTCACTAGAAGCTAAGCTATTC	279	GAGACGCAGATAGGC
[illegible]	15	[illegible]	[illegible]	TATGTTGTATTTTAACAAATAACATTTTTGTGTGTGTGTGATTCATAGACAGGAAAGTCAGTTACAAAGTTGAGATTTGAGTAT<br>AGGGAGGAGAAACCACAGTGTGATTTCACGGCCAAAGAACAAAGCTCTCTTGTTCTTAGGGCCTTAGACCTGTCTGAGAAC<br>ATGTCCAGCAAATATGTCCAGCAGGTGGCGTTTCACCTCTTAGATTGGTTTTGTAATTACAGAATTCCCATTAATAGGAGACA<br>TTTGGGGAAGTGT	[illegible]	TATGTTGTATTTTAA
[illegible]	7	[illegible]	[illegible]	AAAACAGAGCCAGCCCAAACCCCACAGAGCACCATTGCATCCCAGACAATGTCCAGCTTGTTCAGAGAGCGAGGGAAGAAA<br>CACAACTCCGAAACATACACAAGACTTCCAAGTGAAGGGTGTTCTGAGGGAGGAACAGGCTAGCTTTGTGTCTGGGTCTTG<br>GGTAAAGGTCCCTCTGCAAACACACCTCAGGCTGATCTTCCTTGACCCTCCAGGAAACCATTTCCTAGCCCTGACTGGTTCA<br>CTGATCTTGAACAAGAACCCCTCTCCAACTCTGTGTCTCCT	[illegible]	AAAACAGAGCCAGCC
[illegible]	10	[illegible]	[illegible]	TGCTCTCTAACTCAGTGTCACACTGAACCCCATTTGAGCTGAACACTGAAAGTGGAGGTTCAGCCTTGGGTTGGGCACCTG<br>GCTAACGAAGCCTGTGGCAGAAACAGGCTTTCATTCATCAGTGGAATACAGTGGGTTTCTGTTTCACAGTTTGGGGTGGGC<br>CAATAGTACGGTGGTGGGTCTACTAAAAGATGAATTGGCACCCTTGTTAGGAAGGTGTTAAGTCCCTCCTGGGTCACCTTG<br>AGATGCCCTATTTTTGTAGCAGCAATAACTCATAT	370	TGCTCTCTAACTCAG
[illegible]	[illegible]	[illegible]	[illegible]	TGTTATCAAATCCCAGATAGCCCGACTTTGATAACACGGTACATTTTCATTTCCTCTCATAAAGAAAGTGAAACATCTCCACC<br>ATTGACAGTCACCAACAAAGTCTCAGGGCTTTGAAGAGAACAAAGAGTCCCATTCATAGCCCAGAGCAGGACTGAACTGGA<br>AGGCGGTTCATCTTCCTCAAGTCCCCAGCCACCCGTGTCTCTAACACAATACACCTGGAAACAAAGACGGCTTAGGAAGCTT<br>TCAAACGGATCTCCTAGCTG	[illegible]	TGTTATCAAATCCCA
[illegible]	7	[illegible]	[illegible]	GCCTCCCAGCTGCCGTGACTTATGAAAACAACAGACCCTGCTCTGTTGTCTGGAGAAAGCAGGGTTGTTATGAGTAACTTTA<br>TTGCAGGCACATGAGTGTGTTAAGACTTTAAAACACGCTCAAATGTCATGTGCACACCCAATGACGGGCAAGAATAATGATG<br>AAAAAACAAGAGATCTATGTCTATGAAACCGTGTTGGACAAAATGGGCCATTCATTGTGTTCCCCTGGGAATGCCTGGACTC<br>TGCAGTCAGAAAACAAAAAAGGCACTTTTTTCC	[illegible]	GCCTCCCAGCTGCCG
[illegible]	[illegible]	[illegible]	[illegible]	[illegible]<br>ACCCGCAGCCGCCTCACACTTTTTGCCCGCCTTTCCTTTTTTTGGTAGAAAACCGCTCCCCGGGCCGGGCGCGCGGCGCGC<br>CAACTCCTCCGCCCTCCCGCGGCCCCGCTCCCGCAGCCCCGGAAAAGCCAGCTTTCCTCCCGCCGAGCTCCCCGCTTTTT<br>AATAAAATCCAGGTAGCGCCGGTTTAAAGAATCCCAAATCTCCATATACAGCCCGGGATTGGAAAAGGTACATTACACAACCC<br>CCCTTTCAAGTTCCTCTCGCTGGATCTAAAAAAAAAAAAAAAAAAAAAAAAAGCGAAGGGGGGGCTTAAGGGGGACGGGGG<br>AGGGGAGCCTCGCAGGCGAAGAAACACGCATTGTTCCCGGGTGCCCGCCCCCCCCTTCGGGCCAGCCCCCGGCCGCAGCG<br>CATTGGTCCCGCTCGCCGCCCATCACGCGCATGTAAACACCTTGGCCCTCAGCCATTCCTATAAAACCAATTACGGACCTCG<br>GGGCTCCAGCAGTTTCCAGGCTCCCCACGGCGGGGCTGAATGATCAAAACTGGTGGTGAGAATTTTTCCGAGCCGTTTCTA<br>GGCAGCCCTCCCCCACACCAGCTCCAGCAGACCCCCCCCTTCTTTCTCCTTCTCCCTCCCCCCACATCCCAGCCCCTCCTCC<br>TGCGTCTGGGCTGAGTGATCAAAATAGAGGAAGATGGTTGTCGCCGCAATCCCGGGCATTGCAAGGCCTCCTGTCAGCTTC<br>CTTCTTCGGGAGGAGGTGACAAGCTAGCCGGGAAGAGCCGGGAACCGGCGAGCTGCTCATTGCGCCCGCGCCCGGCGCTC<br>CCTCTCTAGGGTGCCACTCCAAAGAGCGAGGTTCAGACGAAAAATAACAAGGCCTGCTTCTCTCTCGCTTGCTCGCTCACT<br>CTCGCCTTTTAAAATAAAGCAGCGATTCATTCCCTTTCCCCCTAGCAGTCGCGGTCGGGTGGGGTGGGCAGGCAGGCGACA<br>AGGCCTTTTTTTTTTCCTTTGCCCCTGCCTGCGGGTCCCCTCCCTGAAACGCGGTGTCAGAAGGCTTTACTGATTAATATTT<br>TGGACAGGCCGCGGCGGTCGGCAGCGGTGAGTTGCATCTTATAAAAGGTTCCTATAGAAACCCGGGCTGTGGGGCTCTGCG<br>GCCGAGTGCGCACCGCGCGCCCCTCCCCAAAGCCCCTTTTGGCGTGGGCGCCCCTCCTTCCCCCTTTCCCCGCCAAGAAAA<br>ACCAGAGCAGAAAAAAGGATTAGATCGGCGGAACGCGAGCTGAATCCCTCTCGGTTCTGACATTTCAGCCCTATTAGCATTT<br>CTCCACGGGGCCTTCTGAAACCTATTAAGGTGTAATATTAAAAACCTCTTGGCTGGGGGCCTCTCTCGCCTTCCATCCGCCG<br>CGCCCCCTCCAGGGAGGGCGAGACCGGGAAAGGGAAAAAAAAAAATCTGTTGAGCTCAGAGGAAGCAAGCCAAGCCCAAA<br>AGTGCTTGGCTGGCCACGGCGGCCAGAGGGGGAGGGGAGCACTAATCCCCCAGACTTTGTACTTTTATTTTGCTCTTTCAG<br>CAAAAAAAAAAAAAAAAAAAAAAAAAAAAACTTTCGAAGTTAAGTGGCAGGGAGCCGCCGACGGCAGTCTACCTGACTGGG<br>GACCCAGGGAGTGTGATCCTTGCCGAAGGGACCCGTTCTAAAGGGTGGGGAGATCCCCTGCACCCTCTCGGAGCCCGAGC<br>CCAGCTAGGCATTACCTACCCTGGAAAGGAAGCTTGGAAAGACAGAGAGCCTTGCAGATCCTACCTCGAGAGCAACTATAG<br>GGTTTTAATTAAAACAAAAGAAGCCCGCTCTTTCGCCCTGCTCTGCGGACTCAGCGTTTTCCTGCTGCTGCTATTTTTTTTT<br>AAGAAAATCCATTTTTAAGGCAATTTTTGAGAGTTTTTTTTTAATGAGCTAGTTAGTGCTCATCCGTTCTATTTTTCTTTCCTC<br>CACTTTTAGAAGGAAAAAAAAAAGAGGGAAGATTCGAGAGAGAGCTGGGGGTGTCTAATTTAATTCCAGGATAAATTTGAAG<br>GGGGGAAAAAATTCAGCCTCTTGAGAGCAGTTTCAAGTTGCCCTTCCTGGAGCTTTCTGGGCTGGTTTCTAGGCTGCCCTC<br>TTTTAGAAGCTGGAGCTCGGTGGGGGGAGGAGAGAGGAGGGAGAAGAGAGAGGGAGAGGGAGAGAAAAGAACGAAAATA<br>GCCGGAGCTGAGGGGAACTGAGCGGCGGTGTCCGCGCGCTCGGAGCTGGGCGACGGGGTCTCAGTGGCAGCCAGGGGCG<br>ACCGGAGGCGACCGGTGCTGCCCGGGGACCGGCAGGAGCAAAGACGGAGAGAGGGGGGTTAGAGAAAGGAGGTATTGTG<br>TCTGTGTGCCTTAGGGGAGGGGGGGAGTTGATTGCAAGAAATAAAATAAATGCTGTTAAGCTGCGAGGGCTTCTGGAAACA<br>AGAGAGAAACGGGCTGAGGGGGTGGGGGGCGCCTAGTGCCCTTGAGTCTTATCCAGAGGCCTTTGTGGGTTCCTAGTTAAA<br>CCCTCCCACGCCTCAGTCGCTCGCTTTCCCCCACCCCCCAACGCCCTCCAATATTCAGAGCGCAGGCCCTGGTCTCCCCCC<br>AACCCCCTCATCCCTCCCGGGATCCAGAATGTTTTTCTTATTTTAATACAGCTCAAGAAAAAATATGTATCTTGAGAGAGTCG<br>GAGTGGGGTGGGGGGATTGTGCAAAAGGCTTGCGGGGTTGGAAAAAAACAAAGGCCTGTTTTTATAAATGTTTAATGTTTT<br>CACCCCTGGATGCTCCAAGACGCCGTAATTGTGACGGCGGGGTATGTGTGCCATAAATCATTTAGCTGCTAATAAAAAATTAT<br>TCCAATTTGCCCCGGATTTGCCGATGGCGTTTGCATTTTTCAAGTGTGCGCCCCGTCGGCGTGGCATACCGACCGACCCAGA<br>[illegible]	[illegible]	ACTCCGGCAACCGCCGCGGT
[illegible]	11	[illegible]	[illegible]	ATTCCTGTTTGATGTAAAAAACTGGATTTCATGTAGATGTGGCTGGCTTTAAAAATCCTAAAACTGGCTTCAAATTCTTGACC<br>CCTTGCCTTCAGCTACCACGTGCTAGGATTACAGCAGTGAGCCACCATGCCTTTCACCAACAGAATTTACTAAATAGAGAGT<br>GCAGTCATGGAATTGCCATTTTCCCCACGTGGTTTTTCGTTTGTTCCTCACCGGAGGCCTCCGGTTTGAGCCCGAAGTCTAA<br>ACCGGTGCGCGTCTGCGAGCTCGCGTGCGCCTCCGCGGCCGGAGCACAGTCGCGGGCGGGCATCAGGGCGGTGCGTGGC<br>CGTCAGTGACGCATGACCCTTCGCAGGCAGAAGTGGGACGGGTCAAGAGAGGACTGTGATCCGATTGGCTGTTCGGCGAC<br>CGTATGCTAATCGCGGTGACAGTCTGCGTGGTGTAGGGACACGGTAGCGACTCAAAATGCCCCGTGGACGGGGTCTGGAGA<br>GGTGTCGGTCTGTCAGAGAAGGTGTCCCCGCGAGGGCAGACGCGGTGTGAGATAGAGGCGCGGACAGGGATGTCACGGGT<br>CGTCTGTGCGTCAGTCGCCGTGACTAAGATGTGGGGGCGGCCGGTGCAGTGCGGCGCGGTGTGGAGCGTGAGGTTAAGAC<br>TATACTTTCAGGGATCATTTCTATAGTTCGTTACTAGAGAAGTTTCTCTGACTGTGTAGAGCACCCGAAACCACGAGGACGA<br>GACGTAGCGTTCCCTCCTGAGCGTGAAGCCGGCTCTAGGTGCTGCTTGACTGCAGCTGCCTCCTGCCATTGATGATCGTTC<br>TTCCCTCCTTGGGAGGGTCAGAGGGAGGGAACGCAGTCTGAGTGGACTCGGTTTATCTTAAAGGGTTTTAAATATAGAAA<br>GCTTCCTTAGTCTTATGGGTGAGAGTGCTGTTTAAGCCTTACCTAGGTATGTCTTTGAACATTTATTACAGATTGTTATTTGTG<br>ACTATGACCACTCAGGTGTTCTGTTCTTGTGAATTTAGACACACTCAGAAAGGCTACACACTCAAAGACCACACCAAAGTGA<br>AGTACTATCTGGGCCGTGAAACTGGTCACATATCAGTTATTTTGCAGTAAAACAAGGAAGTCAATCATACTGCTTCAGTGGGT<br>CAGGGAGTCAAGAATGCACAGTACAAGAGCTGGTGAGATTCCTCAGCAGGTAAGAGCACTGAATGCTCTTCCAAAGATTCT<br>GAGTTCAAATCCCAGCAACCACGTGGTGGATTTGAACAAAGGATGAAAACAACCATCCATAACCAGATCTGATGCCCTCTTC<br>TGGAGTGTCAAAAGACAGCTATGGTGTACTTACATAAATAAATAAGTAAAAACATTAAATAAGTAATTTAAATAAATAGGTAAA<br>TAAATATTTTTTTAT	[illegible]	ATTCCTGTTTGATGTAAAAA
[illegible]	6	[illegible]	[illegible]	TTTGTAATTATGCCCTGCATCAAGGTTTGTAGCTACAGTGAAATATTCACGCATCAAACAGTCATTGCTACATCCAAACTGC<br>TCGTACGCAGGAGTTCTCGTGCCATTTGCTATGTCTACAGAGAGGCACAGGTGAAAATCTCGGGACTACCTTCATCTTCAAT<br>GTTCCTTCTCCCTCATCCATATACGTTACTCTGAGTCATTGCAGCAGTGCGCTGTAAAGATTGTTAATCCATGCAATGTAGCT<br>TATTAAGGCTACCAGAACAAACGAGCCCTGTCTTTCTGAGTGCTTGCAATACACGTACTTACCGGGCATAAAGCTTTCCCCA<br>GGTGCCACCATCCCAGAGTACGTTTTATTGAACTGACTTACAGCGTAGTCTGCTCTCTAAGAGAGTGCTGCAACAAGGCAGG<br>GATGAGGAACACGGAGAGCGAGCTCTGTGTGCACAGTGCACGGCCCGCCAGCATTAATGAGGGTTGCGATTGTTCTGGGG<br>GAGAAAGTACGGGATGTTTTCACTGTCCTTATTCATGGGACAGTCGGTGCCTGTGGCACTTTTAGATACGTTTCCCGTTCTG<br>ATAAGCTTGGAGTCTAGTCCTGCCAGGTGACAGCTCAGGCTTTGCTTTAGTGAGTCCACAGCTCTCGTACCTATCATCGCAC<br>CCCGAGGACAGCGGAATGTGGTCCCAGCCTCTGAGGGGTTCAATTCATTGCTGGAAAGCAAGTGATATTTTAGAGGGAGCC<br>CGACTGCAACCACTGCGCTTACATTATGTGGGTAACACTTCTGTCTGGGAAGGGAGAAGATAAAGAAGCCTGAAGTTGGGA<br>AAATAGTGGAAAGCAAGTGAAAGACAATGCAGGAAAGCAACACGGAGAGCTTCCAGGTGCTGGGCTCCGGCCATATGAAAC<br>TGTCCAATCTGGGACTAATCATTTCAGTCATTTATGGGTCGGCGTGCGTCTCACTTCTCATAAGCGGCTCGCCCCGCTGTAA<br>ATGACATTTTGTTTTATGAAAGCATAATGTGAAAATCCACTGATAAGCCAAATAATAAAAACGTTATAGCATGTCAGGGCTTTT<br>TTATTATTATTATTTAACCTCAATCAAGTTATAATACTGAACACATGTATCCTCGGCCTAAATCACCTTGTCAGGTTTAACTGTA<br>TATTCAAGGTCATTTACATGACATATGATCTAACACAGGAGAACAACAATGCTATTGTAGTGTTAATTGAAAACTCCAAACTG<br>GGAGCCAGAGGAAACCACCGGGCTGATTGCTGAGGGTCCCAGGGAAGGGATCTATTCCCACACCTGCAGTCGGTCCATAAC<br>ACGGACATTGTTACACACATCTGGGTCTAATCAGTCCAGGGGCCTTGATACGCTGCTCCCGGGGCACATAAATTCAGCATTA<br>CATTTATTTTAAAAGCTCCCTCTTTCAGTTTTTTAAGGACAAGATAGATTCATGGCTCCACAGAGTCTGACAAAGTCGAAACC<br>ACAGACAAAGTAAGTTACTGCCACAAGCCTCGTAGACTTAACTTACCAGTTCTTTGCCACTTCCATAATGGAATTTTATATCC<br>AGCCTTGGCTAGCAACCGTGCATTTCCACCAACAGTGAAGTTTGACTGAAAAGCTGCATTAAAAATGAA	[illegible]	TTTGTAATTATGCCCTGCAT

Sox6	7	122514384	122515350	TGGCAATGATATAAGTTAGCATATCTTTCTGTAATATATATAAAGAATATAGAGGCCAATGTGCATATATGTGCACATCTGTCTA<br>TATCTCACTCATATCTGTGTATATTTGTTTGGTTACATACATGTACATATATGAAAATATTTGACCAAAAGAGCTAAAACTTATA<br>ACAGCAAATAAGCCCAGATAGCAAGGCAAGACCAAACTACAGAAAGCACTGTGCTACAACCAAGAACAGAAACAATGGCCA<br>TGCTGGTGTGCCCTCCAGGCTCAGCCCATCTCCTCACACTGAGGGGAAAGCCATGGGTTCTGTGGGCAGCAGACCTCACAG<br>TCACACACATACACTGAGCTGTAGGAGCTGAAGCCATCCCTGCCGAGGATTTCCGCCCTTCATCTGGGCCATAATCCACCTC<br>CCAGGCCACACAGTGCCTGGCTTGTGCTTAAACAAGGCACGCACTGTACCAGTGTTCTCACATCACGCTGCGGGAAGGCAA<br>ACAGAGCTGGGGACCTATGCCAAGGTTTTCAAAGGCTGCCCTGAAACGTAAATGTGGCCTCTGTACATTCTTCAGGCAACG<br>CTGGGGATCATCTCTGTGGTACCAGGGCCAGCACAATTACCCTATTACTACCCGATGGCGGCTCCTGATGAATGCATCTCTG<br>TGATGGTTCCTTTTAACCTGACGACAGGAAAAGGAAAATCTATGGAAGGGGGCATGAGGCAGAGTTCCATGAGCTCATGTC<br>TAGTGGGGAAAGCTAAAGGCATGTGAAGGCTGGGGGTGAGTGGATCACATTGCTTCTGGCTTCAGGGGCAATGTTATTGCC<br>CCTTCCTCTAAGAACTTCCATGGGTTAGCCTCAATGTGTGGGGACATCTACCAACTGGAATTCCGTGAAACAGGGGCCACAA<br>ATGTCAGGATATACATTCAGGTTTGTGTACCCTGGGCTGAATAGCTGAAAATCTCTCTCTCTC	967	TGGCAATGATATAAGTTAGC
Sox11	12	28260162	28261218	TTACTTGATTTTTCTATTTATTTGTTTGTTGGTGCATGTGCCACATGTGTGCATCATAGAAGAGTCCAAAAGAAGGGGTGTCA<br>GATTCCCCCTGGAACTGGAGTTACAGGCAGTTGTCAGCCTTCCAACATAGGTCTTACACCTGAACTTGAGTCCTCTGGAAGA<br>GCAGTACTAATTTTAACAGCTCACCCATCATTCTACCTTTCTGTTTACTTAATTTTTCATTTGTGGCTTTTACTTGCTTAGTAGA<br>ATTGAATTTGCTGAGAGCACCAATCAGCAGCTATACTAAGAAGAGACCGTGTCAAGCCATATCCTACTAACCTTGACAGCAG<br>CCATCCAATGTCACCTACATGACCCCCTCCAGCTCTCCAGCGATGTTGCTCCAGCTGGCTGGATAGCCAAGCTGTTCGGCTT<br>CTTGATCCTCTAGGAGGGTGAAGCACAACCATTGTCAAAGCCAGGGAAAGTTTACAAGCTTCCTCAGCCCTGGCAAGTGCA<br>GCTGGGGCAGTTAGAACACAATCCAGCCAATTGTGCTTTCTCCAACAATAACCTTCCTCATGAATAGGGCGGCCATATGGTG<br>CCATTTCCCGGACGCAGTTCTGGTTTATTCCTAGTGACCCTGCGTAACTGCTGAGAGCATTTCTTTTCACTCCCAGACTATGC<br>TGAGCTGGATAATAAACATTACGATCAACCTGCTCATAAACATTATTGTGTGCTGTAATTAATTTATATCAGCATCGTAAGTCA<br>AGATACAAGATAAGTGCCATTTAAGTAACTACCAGTAGGTTCAATGTGAGAGCTATGCTAACTCCATTTATGTTATTAATTATC<br>ACTGCCTAAATTAATTAAATATTCCTTATCTGAGCTACCCAGAAACAGACTTCTGCTTTGCAATAAGCCTTCAAAGCCCTTTCT<br>TCCAAGTCCCTCTAGGGACGCATGGCCTCTGGGTCACACTGGTGCAGGTGATGGCTTCAAGGCGTGTCCCTGAAGCCTTGA<br>GATAGAGGGTGATGTGTTTTCTTGCCCAGCTGCCCTCAGCCATAGCCAGAGAGCCACTCCCCTC	1057	TTACTTGATTTTTCTATTTA
Tgfb2	1	188464587	188465728	CAAGGCTGACATTATTACCTCGGAGGTGGCTTAGCCAGCCTAGTACTTACTACTTCTGCTAAAGCCATGTCCTGTAATTGTCA<br>TGGCTCCTTTGTAATTGTACCTTTCCAAGAAGTCCCAAGTAATTTATGCCTAGCAAGGATAGCTTGCAGAGTCAGCCTGAAAC<br>ACTCGTGAGTGTTTTCCCTTTACAGTATGTAGCTTAATCCTTCATAGACACCTATGTTATGCATAATCATGTGTGCAGCCATGA<br>TCAAGAAGAACACACAGAATCTTTAATGACCTTTATGCTAATGCTACCATATATGTGTTCAGATACACACACACACACACACA<br>CAGATACATAGCACAGACACACACAGACATAGACACAGACATAGACACACAAACACACACACATACACACACACACACACAC<br>ACACAGACATAGACACACAAACACACACACACACACACACACACACACACACCCCACTCCACATCACTCTGTGATGGAAAC<br>ACCTTACAATTCTCTATTGTTCCCCTTTCTTTCTTTCCTTCCTTCTCTACAGATGGGCCTTTGTGCCAACTGTGACTGCATATG<br>GCATAAACAAGTAAATTCACTTCTCCAGGTCTAAGGGATGAAAGAGACCTTTGTCACTTGAAATCTACATGCTGCCACTCAG<br>AGGAACATGGGAGGTCATTGCCGACCTCTCTCACAGCTTGAGAAGGGATTCTACCAGACTGAATCACGTCAGTGCAGGAGA<br>GAGACCATGCAGCATAGCACTCAATGGTTCAAGGACATGGTACCCTTTTGCTGGCTAAACTCCCTCATTCCTTGGTGGCTAT<br>ACCACCCAGGCTAGGACTATGCACTGTGCCAGTGATGCAGTTGTCTCTTTCCACAGATGGGGCTGATTCTGAGATAGGCCCG<br>TGGACTGACTGCCCAGGGCTTTCTTCTGATTTTCTTCTCTGATTTCCTGTTCCAATTCTCTGAGCTGACATGAACACACATGG<br>GCTTGAAGAACACATTTCTAATACCTTCTTTTCTACTTCGCAGTACCTACAATTCACATGAATTATAATTTGTGTATACCTTTAC<br>ATTCAGCTGAGTGTTTTCATTAGTGAGAGGGGCTATACAGTAATAAGCTTAAACAAGTCAATAATAG	1142	CAAGGCTGACATTATTACCT
Tle3	9			CAGCACCTGTAAACCCACCCAGCTACAACTCACTGAGCGAGCGCATTGCTGACCCTTTGAGACCCCCCACCCTCACTACCCT<br>GGCAATAACTGTTCTCCTGAGTCAACAGCTCTCAGGTTCCAGATTACCTCTTGGTAGGGAAGCCACTGAGGGTAAGAGAAG<br>ACCCCTGCCCTGACTCTGAGCCCCTCAGTAACGGGTCCTCCTCAAACCTGAGCTTATCAGCCCATGGCTAGGATCCCGTAAC<br>TTGGGATGACATTCTCAGAGCACTTCCGGAGCGCTCGGCCTCCTTTCTCAACAGGCAGAGGGGACTTCTGGTTTGCTGCTC<br>ATGGGAAAAAACAACAACAACAACAACAAAAAAAAAAAAAACCACAAAAAAACCCCAAGAAATTCCAGTAAGGGATTAATGA<br>CTTAATTATAAATGCAGTTTGGAAATGTAAAGGTTATTAAGGAGGGAGAGAGATGCATGCTAATAAGAGACACGCTATTAACA<br>GCAGAATAAAGATCCATTGTTGGCGCCACCATAAAGTTTTCCAAGTTTGGTAATAAAAACCACCTGGCCTGCACCAGGCTGG<br>CTGCCATGGTGACGCACCGCGCTCTGGATCCGGCTAATGAGGCTTGTCTGCCGGCCTCTGATCATGCTCCCTAGGGGGTTT<br>AGGCTTCCTGGTAAAATTGGCCCACAGGCCTCAAACCACTGCAGAGACAGGCAGGCCCCCAGCTTCTCAGCTACCGCCCAC<br>TGGGCCCTGAGCGCTTTGAGAGTCACCAGAAACAAGCCATTCCTGTTCCCACAGGAAACTAGCAGGCTGTTCTAAGCTGTG<br>TGACCTTGCCCCTGTTTCTCAACCTCTCTGAGCCTGTTTCCTCATCTGCAAAGCGTGGAGATCTTACATCTGGCTCTAGGGA<br>AGAGACACACACATGTAAAGTACCTCACACAATCATGGGACCGTCTTCAGAGGCAAGAGCACCAGAGCATCTCCTTCCAGTA<br>ACCCCACTCTAGCCTACTTGTCTCAGGTTACCTGGCAGCCCCACCCCTAACTGTGTCTGACCTCATTCACCACAGTGTGCCC<br>CG	1063	CAGCACCTGTAAACCCACCC

Table S4 Sox5 (2 pages)

Gene	Microarray		Chip-Seq			Association
	Fold Change Absolute	Regulation	Chip-Seq tags count	Fold enrichment over input	Distance from TSS (bp)	
Ing4	1.6	up	39	6.9	-41	TSS
Ing4	1.8	up	39	6.9	-41	TSS
Magi1	1.9	up	56	8.9	-72	TSS
Mtf2	2.3	down	32	9.0	-138	TSS
Gipc1	1.6	up	30	7.8	-154	TSS
Pin4	3.6	down	44	12.5	-181	TSS
Chchd3	1.6	up	53	18.5	262	TSS
Laptm4a	2.1	up	24	8.4	319	TSS
Sox5	4.6	down	118	35.6	567	TSS
Sox5	6.4	down	118	35.6	567	TSS
Sox5	2.3	down	118	35.6	567	TSS
Sox5	1.9	down	118	35.6	567	TSS
Magi1	1.9	up	28	9.4	-593	TSS
Maf	1.7	up	31	4.7	1180	promoter
Sort1	1.6	up	41	10.9	-1504	promoter
Sort1	1.6	up	41	10.9	-1504	promoter
Ccdc6	3.4	down	26	6.2	-1807	promoter
Gpsm3	1.5	up	179	30.9	-2894	promoter
Sorbs1	3.8	down	27	8.9	7871	proximal
Reep5	1.6	up	29	6.9	9843	proximal
Papss2	1.7	down	22	8.4	6343	intragenic
Papss2	2.0	down	22	8.4	6343	intragenic
Ntng1	2.3	up	24	6.1	-7143	intragenic
Papss2	1.7	down	30	8.0	10832	intragenic
Papss2	2.0	down	30	8.0	10832	intragenic
F2rl1	1.9	up	17	6.1	-12615	intragenic
Ednra	2.4	up	59	12.7	-12940	intragenic
2610035D1	1.6	down	30	6.9	-13207	intragenic
Bach2	4.0	down	29	8.4	13796	intragenic
Col9a1	1.6	down	23	7.0	18627	intragenic
Col9a1	11.8	down	23	7.0	18627	intragenic
Myo10	1.7	up	38	8.4	20188	intragenic
Nfkb1	1.5	up	21	6.6	-26534	intragenic
Col22a1	1.7	up	21	4.7	-29483	intragenic
Nedd4	1.5	up	21	6.5	29752	intragenic
Myo10	1.7	up	41	10.8	31284	intragenic
Igsf3	2.1	down	19	6.4	33860	intragenic
Slc24a3	1.6	up	26	7.7	50443	intragenic
Rapgef4	1.7	up	18	5.6	56846	intragenic
Adamts2	2.2	up	20	4.8	60853	intragenic
Adamts2	2.8	up	20	4.8	60853	intragenic
Tgfb2	4.1	down	27	8.7	-64820	intragenic
Parva	2.1	up	19	5.2	72679	intragenic
Ccdc46	1.5	up	18	6.1	75647	intragenic
Sox5	4.6	down	37	5.2	-152627	intragenic

Sox5	6.4	down	37	5.2	-152627	intragenic
Sox5	2.3	down	37	5.2	-152627	intragenic
Sox5	1.9	down	37	5.2	-152627	intragenic
Dnahc11	1.5	up	44	5.0	-154796	intragenic
Unc5c	1.7	down	23	6.6	155903	intragenic
Sox5	4.6	down	31	8.4	-170168	intragenic
Sox5	6.4	down	31	8.4	-170168	intragenic
Sox5	2.3	down	31	8.4	-170168	intragenic
Sox5	1.9	down	31	8.4	-170168	intragenic
Nfib	2.0	down	28	8.0	-200639	intragenic
Nfib	3.7	down	28	8.0	-200639	intragenic
Nfib	1.5	down	28	8.0	-200639	intragenic
Nfib	3.0	down	28	8.0	-200639	intragenic
Nfib	2.9	down	28	8.0	-200639	intragenic
Lrrc7	1.7	up	20	7.0	-209118	intragenic
Sox6	2.4	down	25	8.0	-379335	intragenic
Sox6	2.4	down	21	8.0	-414475	intragenic
Arhgef16	1.7	up	58	13.7	13560	distal
Frs2	1.6	up	31	9.1	14405	distal
Krt7	1.6	up	25	8.0	-15748	distal
Slmo2	1.7	down	45	11.7	16675	distal
Col25a1	1.5	up	45	8.6	-19767	distal
Col25a1	1.7	up	45	8.6	-19767	distal
Ypel5	1.5	down	34	5.2	30324	distal
Mef2c	1.8	down	30	8.0	-42186	distal
Mef2c	1.6	down	30	8.0	-42186	distal
Krr1	1.6	up	34	7.1	-47067	distal
E130114P1	3.1	down	19	6.4	52207	distal
Sox5	4.6	down	43	14.5	55265	distal
Sox5	6.4	down	43	14.5	55265	distal
Sox5	2.3	down	43	14.5	55265	distal
Sox5	1.9	down	43	14.5	55265	distal
Rasl11b	1.5	up	20	5.6	58943	distal
Rasl11b	2.1	up	20	5.6	58943	distal
Mtx2	1.7	up	23	7.0	62853	distal
Rnpc3	2.0	down	38	9.4	69125	distal
Rasl11b	1.5	up	31	6.6	71025	distal
Rasl11b	2.1	up	31	6.6	71025	distal
Foxo1	2.2	up	36	9.0	-71747	distal
Tmprss2	2.1	up	21	6.1	72330	distal
Hs1bp3	1.6	up	27	8.9	73098	distal

Table S4 Sox6 (2 pages)

Gene	Microarray		Chip-Seq			Association
	Fold Change Absolute	Regulation	Chip-Seq tags count	Fold enrichment over input	Distance from TSS (bp)	
Ing4	1.6	up	39	6.9	-41	TSS
Ing4	1.8	up	39	6.9	-41	TSS
Magi1	1.9	up	56	8.9	-72	TSS
Mtf2	2.3	down	32	9.0	-138	TSS
Gipc1	1.6	up	30	7.8	-154	TSS
Pin4	3.6	down	44	12.5	-181	TSS
Chchd3	1.6	up	53	18.5	262	TSS
Laptm4a	2.1	up	24	8.4	319	TSS
Sox5	4.6	down	118	35.6	567	TSS
Sox5	6.4	down	118	35.6	567	TSS
Sox5	2.3	down	118	35.6	567	TSS
Sox5	1.9	down	118	35.6	567	TSS
Magi1	1.9	up	28	9.4	-593	TSS
Maf	1.7	up	31	4.7	1180	promoter
Sort1	1.6	up	41	10.9	-1504	promoter
Sort1	1.6	up	41	10.9	-1504	promoter
Ccdc6	3.4	down	26	6.2	-1807	promoter
Gpsm3	1.5	up	179	30.9	-2894	promoter
Sorbs1	3.8	down	27	8.9	7871	proximal
Reep5	1.6	up	29	6.9	9843	proximal
Papss2	1.7	down	22	8.4	6343	intragenic
Papss2	2.0	down	22	8.4	6343	intragenic
Ntng1	2.3	up	24	6.1	-7143	intragenic
Papss2	1.7	down	30	8.0	10832	intragenic
Papss2	2.0	down	30	8.0	10832	intragenic
F2rl1	1.9	up	17	6.1	-12615	intragenic
Ednra	2.4	up	59	12.7	-12940	intragenic
2610035D1	1.6	down	30	6.9	-13207	intragenic
Bach2	4.0	down	29	8.4	13796	intragenic
Col9a1	1.6	down	23	7.0	18627	intragenic
Col9a1	11.8	down	23	7.0	18627	intragenic
Myo10	1.7	up	38	8.4	20188	intragenic
Nfkb1	1.5	up	21	6.6	-26534	intragenic
Col22a1	1.7	up	21	4.7	-29483	intragenic
Nedd4	1.5	up	21	6.5	29752	intragenic
Myo10	1.7	up	41	10.8	31284	intragenic
Igsf3	2.1	down	19	6.4	33860	intragenic
Slc24a3	1.6	up	26	7.7	50443	intragenic
Rapgef4	1.7	up	18	5.6	56846	intragenic
Adamts2	2.2	up	20	4.8	60853	intragenic
Adamts2	2.8	up	20	4.8	60853	intragenic
Tgfb2	4.1	down	27	8.7	-64820	intragenic
Parva	2.1	up	19	5.2	72679	intragenic
Ccdc46	1.5	up	18	6.1	75647	intragenic
Sox5	4.6	down	37	5.2	-152627	intragenic

Sox5	6.4	down	37	5.2	-152627	intragenic
Sox5	2.3	down	37	5.2	-152627	intragenic
Sox5	1.9	down	37	5.2	-152627	intragenic
Dnahc11	1.5	up	44	5.0	-154796	intragenic
Unc5c	1.7	down	23	6.6	155903	intragenic
Sox5	4.6	down	31	8.4	-170168	intragenic
Sox5	6.4	down	31	8.4	-170168	intragenic
Sox5	2.3	down	31	8.4	-170168	intragenic
Sox5	1.9	down	31	8.4	-170168	intragenic
Nfib	2.0	down	28	8.0	-200639	intragenic
Nfib	3.7	down	28	8.0	-200639	intragenic
Nfib	1.5	down	28	8.0	-200639	intragenic
Nfib	3.0	down	28	8.0	-200639	intragenic
Nfib	2.9	down	28	8.0	-200639	intragenic
Lrrc7	1.7	up	20	7.0	-209118	intragenic
Sox6	2.4	down	25	8.0	-379335	intragenic
Sox6	2.4	down	21	8.0	-414475	intragenic
Arhgef16	1.7	up	58	13.7	13560	distal
Frs2	1.6	up	31	9.1	14405	distal
Krt7	1.6	up	25	8.0	-15748	distal
Slmo2	1.7	down	45	11.7	16675	distal
Col25a1	1.5	up	45	8.6	-19767	distal
Col25a1	1.7	up	45	8.6	-19767	distal
Ypel5	1.5	down	34	5.2	30324	distal
Mef2c	1.8	down	30	8.0	-42186	distal
Mef2c	1.6	down	30	8.0	-42186	distal
Krr1	1.6	up	34	7.1	-47067	distal
E130114P1	3.1	down	19	6.4	52207	distal
Sox5	4.6	down	43	14.5	55265	distal
Sox5	6.4	down	43	14.5	55265	distal
Sox5	2.3	down	43	14.5	55265	distal
Sox5	1.9	down	43	14.5	55265	distal
Rasl11b	1.5	up	20	5.6	58943	distal
Rasl11b	2.1	up	20	5.6	58943	distal
Mtx2	1.7	up	23	7.0	62853	distal
Rnpc3	2.0	down	38	9.4	69125	distal
Rasl11b	1.5	up	31	6.6	71025	distal
Rasl11b	2.1	up	31	6.6	71025	distal
Foxo1	2.2	up	36	9.0	-71747	distal
Tmprss2	2.1	up	21	6.1	72330	distal
Hs1bp3	1.6	up	27	8.9	73098	distal

Table S4 Sox9 (17 pages)

Gene	Microarray		Chip-Seq	
	Fold Change Absolute	Regulation	Chip-Seq tags count	Fold enrichment over input
Sh3kbp1	2.3	down	18	7.9
Tnrc6b	1.6	up	25	10.9
Ncam1	1.7	up	22	7.3
Zfp113	2.1	up	22	9.1
Calcoco1	2.2	up	36	15.8
Dnm3os	2.1	down	24	7.9
Tssc4	1.6	down	35	7.1
Zfp335	2.1	up	20	7.3
Ppt1	3.0	down	25	7.0
Bdh2	2.0	up	18	8.5
Rtkn	1.9	down	66	17.6
Ylpm1	1.9	up	20	7.3
Tle3	1.9	up	42	20.6
Rbbp9	2.1	down	72	25.3
Mtf2	1.9	down	62	24.9
Prelp	11.8	down	28	10.9
Fgd4	1.7	down	101	50.9
Cltc	3.6	down	27	10.3
Zfp191	2.2	down	40	14.8
Scamp3	1.6	up	26	7.6
Ints10	1.5	up	21	6.7
Rbm5	3.3	up	33	13.6
Prmt5	4.7	down	25	8.3
Angptl1	2.2	down	28	9.1
Klhdc10	2.6	down	19	6.1
Adpgk	1.9	up	23	8.4
Dpagt1	1.5	up	18	6.6
Zfp462	3.3	up	25	9.7
Nosip	1.7	down	25	6.7
Mia1	12.4	down	60	17.8
Rhobtb3	1.5	down	31	10.3
Slc35a3	1.7	down	31	15.2
Slc35a3	1.9	down	31	15.2
Pde6d	2.1	down	21	6.7
Cmtm5	4.4	down	31	9.4
Col5a2	2.1	up	18	5.5
Brd2	1.9	down	50	22.2
Col9a3	1.6	down	33	17.0
Pafah2	1.7	down	24	6.7
Hist1h2be	1.7	down	27	7.1
Ak1	1.5	up	52	21.8
Gnal	2.4	down	19	6.1
Gtf2i	3.1	down	20	6.7
Cobll1	1.7	down	27	13.3

Gabpb1	2.4	down	26	9.7	576	TSS
Sdccag8	2.0	up	20	6.1	-578	TSS
Sox5	3.3	down	51	17.6	591	TSS
Sox5	5.0	down	51	17.6	591	TSS
Sox5	8.5	down	51	17.6	591	TSS
2010106G01Rik	2.4	down	20	7.9	647	TSS
2010106G01Rik	2.0	down	20	7.9	647	TSS
Cyr61	3.3	down	21	6.7	668	TSS
Hoxa9	1.5	down	21	6.7	-753	TSS
Mical1	2.1	up	33	17.0	834	TSS
Mical1	1.6	up	33	17.0	834	TSS
Gfpt2	2.0	down	17	5.5	881	TSS
Gfpt2	1.5	down	17	5.5	881	TSS
Tsc22d1	17.0	down	29	9.4	888	TSS
Tsc22d1	2.1	down	29	9.4	888	TSS
Gtf3c6	1.7	up	24	7.9	889	TSS
Plekhf2	2.2	down	41	19.4	-921	TSS
Sphk1	2.7	down	17	7.3	-932	TSS
4930452G13Rik	1.6	down	27	8.5	946	TSS
Fxyd3	1.7	down	24	9.4	982	TSS
Cyr61	3.3	down	21	7.2	-989	TSS
Mbnl2	1.8	down	77	40.6	-1075	promoter
Ppp2r1a	1.9	down	28	13.9	-1284	promoter
Mapk9	4.3	down	19	6.1	-1521	promoter
Cic	1.9	up	27	9.7	-2080	promoter
Nfatc1	2.4	down	42	18.2	2142	promoter
Nfatc1	2.0	down	42	18.2	2142	promoter
Nfatc1	3.1	down	42	18.2	2142	promoter
Nfatc1	1.6	down	42	18.2	2142	promoter
Tgfbi	2.3	down	21	5.6	-2619	promoter
Slc14a1	2.2	down	35	9.8	2781	promoter
Slc14a1	1.9	down	35	9.8	2781	promoter
Slc14a1	1.7	down	35	9.8	2781	promoter
Foxp2	1.7	down	92	45.4	-3072	promoter
Foxp2	2.9	down	92	45.4	-3072	promoter
Rab8a	1.6	up	24	12.7	-3102	promoter
Rab8a	1.8	up	24	12.7	-3102	promoter
Sphk1	2.7	down	47	14.8	-3131	promoter
Mbnl1	1.6	down	20	12.1	-3236	promoter
Maml2	1.6	down	35	6.2	-3242	promoter
Clasp2	2.5	down	68	38.2	-3302	promoter
Rarg	1.8	down	23	9.1	3308	promoter
Taf13	1.5	up	29	11.1	-3506	promoter
Slc39a3	1.6	up	19	7.3	3563	promoter
Stard3	2.0	up	17	7.9	-4294	promoter
Glis2	1.5	down	19	8.1	-4294	promoter
Glis2	1.8	down	19	8.1	-4294	promoter
Efnb3	1.5	up	28	13.3	4365	promoter
Rgmb	1.6	down	24	9.7	4470	promoter
Wrnip1	4.6	down	37	12.7	-4567	promoter

Prosc	4.1	down	18	8.5	-4670	promoter
Mbp	1.6	down	18	7.8	-4738	promoter
Ech1	1.6	up	24	9.8	-4806	promoter
Olfml3	1.8	up	19	8.1	-3735	proximal
Ttc30b	1.5	down	36	8.0	-4642	proximal
Papss2	3.1	down	59	20.3	-5055	proximal
Papss2	6.5	down	59	20.3	-5055	proximal
Mycbpap	2.1	up	47	18.3	5077	proximal
Tbx15	2.6	down	27	13.3	-5156	proximal
Tspan8	1.7	down	36	12.7	-5314	proximal
Aaas	2.3	down	28	7.9	5390	proximal
Cap1	1.6	up	44	25.5	5870	proximal
Sec14l1	1.9	up	27	4.6	-6327	proximal
Fscn1	2.4	up	23	10.3	-6538	proximal
Tgm2	2.0	down	24	12.1	6691	proximal
Comp	1.5	down	24	8.5	-6705	proximal
9430023L20Rik	1.6	up	22	10.3	-6717	proximal
Hipk2	2.0	up	21	10.3	6719	proximal
1810011O10Rik	1.8	up	21	6.1	6878	proximal
Mest	1.7	down	40	9.1	-7357	proximal
Mest	8.6	down	40	9.1	-7357	proximal
Fbp2	2.3	down	106	49.1	7536	proximal
Slc29a1	2.0	down	26	12.7	-7838	proximal
Lef1	1.7	down	20	7.9	-7994	proximal
Cttnbp2nl	1.5	down	46	21.8	8009	proximal
Sec14l1	1.9	up	40	17.2	-8053	proximal
Evl	1.9	up	15	7.9	-8417	proximal
Kras	1.5	up	64	15.7	8757	proximal
Rasl11b	4.4	down	22	8.5	-8809	proximal
Camk2d	1.7	down	18	8.4	-9216	proximal
Txndc11	1.9	down	322	6.4	9410	proximal
Txndc11	1.8	down	322	6.4	9410	proximal
Nrip1	1.9	up	17	9.7	-1009	intragenic
Lum	3.0	down	20	7.3	1022	intragenic
Bdh1	1.9	down	18	6.6	1128	intragenic
Bdh1	2.2	down	18	6.6	1128	intragenic
Deb1	1.9	down	51	17.0	1248	intragenic
Spnb2	2.3	down	18	7.3	-1364	intragenic
Zfp238	2.9	down	53	16.7	1464	intragenic
Ets2	2.0	down	21	7.9	1578	intragenic
Lect1	1.6	down	18	8.5	-1607	intragenic
Col9a2	3.3	down	52	7.2	1654	intragenic
Cthrc1	7.4	down	19	6.7	1721	intragenic
Rarg	1.8	down	84	40.6	-1786	intragenic
Akap2	5.3	down	17	5.5	1861	intragenic
Slc35e3	2.7	down	39	15.8	-2102	intragenic
Arhgef19	1.6	down	34	15.8	2182	intragenic
Bahcc1	1.5	down	25	9.7	2232	intragenic
Dusp6	1.7	down	19	9.1	2323	intragenic
Arid3b	1.6	up	30	7.3	-2456	intragenic

Col9a3	1.6	down	47	23.0	2631	intragenic
P4ha3	1.8	down	28	7.0	2890	intragenic
P4ha3	2.5	down	28	7.0	2890	intragenic
Plekhb1	1.8	down	30	12.7	-2901	intragenic
Fmnl2	2.7	down	37	17.6	3227	intragenic
Fmnl2	5.0	down	37	17.6	3227	intragenic
Arhgef1	1.6	up	41	18.8	3238	intragenic
Plce1	1.8	down	19	6.0	3461	intragenic
Tmem2	1.7	down	41	20.0	3870	intragenic
Cap1	1.6	up	25	10.3	-4216	intragenic
E330033B04Rik	1.6	up	17	7.9	-4507	intragenic
Fam107a	2.5	down	24	11.5	-4534	intragenic
Slc35e3	2.7	down	38	19.4	-4701	intragenic
Igsf6	1.5	up	19	8.1	-4714	intragenic
Nfe2	1.7	up	96	53.3	-4850	intragenic
Ptpre	1.8	up	32	8.4	5391	intragenic
Papss2	3.1	down	25	11.5	6296	intragenic
Papss2	6.5	down	25	11.5	6296	intragenic
Amotl2	1.5	down	68	28.5	6378	intragenic
Bmp5	2.0	down	75	36.4	6452	intragenic
Col9a3	1.6	down	35	15.8	6696	intragenic
Rgs10	2.5	up	26	9.1	-6905	intragenic
Extl1	3.8	down	65	31.5	-7083	intragenic
Ntng1	1.6	up	25	12.1	-7118	intragenic
Csgalnact1	1.6	up	23	7.9	-7360	intragenic
Dst	1.8	up	18	7.3	7379	intragenic
Lbh	1.9	down	28	5.6	7783	intragenic
Glt25d2	1.9	down	36	13.9	7909	intragenic
Snx9	1.8	down	17	6.1	8305	intragenic
1190002H23Rik	2.6	down	18	9.7	-8348	intragenic
Flnb	2.5	down	40	4.8	8412	intragenic
Mgll	1.5	down	28	10.6	8545	intragenic
Acvr1b	2.2	down	37	9.7	8728	intragenic
Klf15	1.7	down	30	12.1	8737	intragenic
Cntn1	1.7	down	31	12.7	9086	intragenic
Nt5c2	4.8	down	45	19.3	-9232	intragenic
Ptk7	2.0	up	30	11.6	-9792	intragenic
Aspscr1	1.6	down	21	9.1	10156	intragenic
Etv5	1.7	down	47	18.3	-10594	intragenic
Dab2ip	1.8	down	51	20.0	10704	intragenic
Bahcc1	1.5	down	23	8.5	10763	intragenic
Papss2	3.1	down	68	29.7	10766	intragenic
Papss2	6.5	down	68	29.7	10766	intragenic
Glis2	1.5	down	17	9.7	10949	intragenic
Glis2	1.8	down	17	9.7	10949	intragenic
Bcor	1.6	down	26	11.5	-11013	intragenic
Jmy	1.6	down	21	9.1	-11090	intragenic
Bcl2l1	1.6	down	23	7.9	-11153	intragenic
Bcl2l1	1.5	down	23	7.9	-11153	intragenic
Postn	4.6	down	40	15.9	11642	intragenic

Tns3	1.7	down	20	5.5	-11648	intragenic
Eps8	2.7	down	21	9.7	-11686	intragenic
Sox5	3.3	down	19	7.3	-11711	intragenic
Sox5	5.0	down	19	7.3	-11711	intragenic
Sox5	8.5	down	19	7.3	-11711	intragenic
Scrn1	1.5	down	27	13.9	-11834	intragenic
Cdc42ep3	2.5	down	16	7.3	-11904	intragenic
Cdc42ep3	1.7	down	16	7.3	-11904	intragenic
Lrp1	1.8	up	24	9.7	-12051	intragenic
Ppard	1.6	down	54	19.3	12153	intragenic
Adam11	1.9	up	43	12.7	12677	intragenic
Wwtr1	1.5	down	17	6.1	-12689	intragenic
Sulf2	2.4	up	26	8.3	-12780	intragenic
Mccc2	2.5	down	20	6.1	-13598	intragenic
Bach2	1.6	down	54	26.1	13802	intragenic
Slc35f1	2.2	down	25	6.7	13814	intragenic
Col9a3	1.6	down	41	12.1	13938	intragenic
Nsmce2	4.7	down	17	9.1	14308	intragenic
Fgfr1op	3.0	down	17	7.9	14619	intragenic
Nrp2	1.7	down	25	10.3	14830	intragenic
Ctnnd1	1.7	up	20	9.7	-14830	intragenic
Smox	1.7	down	65	33.3	14835	intragenic
Smox	4.0	down	65	33.3	14835	intragenic
Camk2a	1.6	up	27	8.7	15055	intragenic
Eps8	2.7	down	51	20.0	-15136	intragenic
Lrrc8d	1.5	down	40	8.5	15192	intragenic
Srrm1	1.6	up	30	9.0	-15466	intragenic
Mcf2l	2.2	up	22	11.5	15690	intragenic
Tpd52	1.5	up	32	15.8	-15739	intragenic
Ssh1	1.8	up	27	8.8	-15991	intragenic
Chst3	1.7	down	58	19.4	-16258	intragenic
Entpd6	1.8	down	26	8.5	16639	intragenic
Entpd6	1.8	down	26	8.5	16639	intragenic
Cspg4	1.7	down	116	25.9	16653	intragenic
Maea	4.9	down	28	9.1	17595	intragenic
Rgmb	1.6	down	41	13.3	-17714	intragenic
Aff1	2.0	up	29	8.6	17775	intragenic
Cpe	4.4	down	27	8.3	-17868	intragenic
Ppap2b	4.1	down	29	11.1	18124	intragenic
Mbnl2	1.8	down	45	21.9	18465	intragenic
Col9a1	19.6	down	54	18.8	18482	intragenic
Col9a1	4.8	down	54	18.8	18482	intragenic
Prpf40b	1.8	up	20	9.4	18928	intragenic
Sep-09	2.1	up	55	25.5	19229	intragenic
Chst3	1.7	down	29	9.1	-19566	intragenic
Ppcdc	1.6	up	26	11.5	-19685	intragenic
Gja5	1.5	up	79	36.0	20205	intragenic
Slc18a2	1.6	down	41	14.4	20214	intragenic
Pdzd8	2.1	down	27	10.3	-20779	intragenic
Ddah1	2.7	down	47	9.0	20853	intragenic

Scin	11.8	down	39	17.6	-21077	intragenic
Kif21a	2.2	down	18	7.3	-21327	intragenic
Kif21a	2.3	down	18	7.3	-21327	intragenic
Lrp1	1.8	up	29	10.2	-21446	intragenic
Sgms1	1.6	down	29	12.7	-21774	intragenic
Wwp2	1.6	down	40	7.7	21902	intragenic
Wwp2	5.1	down	40	7.7	21902	intragenic
Snx3	1.6	down	29	11.5	22027	intragenic
Robo1	1.6	down	29	10.5	23276	intragenic
Chst3	1.7	down	47	14.2	-23997	intragenic
Lef1	1.7	down	24	12.1	25166	intragenic
Thsd4	2.3	up	58	15.5	-25333	intragenic
Cxxc5	2.2	down	34	12.8	25817	intragenic
Ptn	2.0	down	27	8.5	-26325	intragenic
Mgll	1.5	down	21	6.6	26346	intragenic
Cd9	2.9	down	18	6.7	-26428	intragenic
Lmnb1	6.8	down	21	8.5	26622	intragenic
Lmnb1	3.9	down	21	8.5	26622	intragenic
Mbnl1	1.6	down	41	11.1	26648	intragenic
Acan	3.7	down	22	10.0	26941	intragenic
Acan	6.2	down	22	10.0	26941	intragenic
Cd9	2.9	down	37	14.4	-27241	intragenic
Gli2	1.6	down	26	6.7	-27360	intragenic
Acot7	4.6	down	27	10.3	27433	intragenic
Tex2	1.6	up	15	7.9	-27980	intragenic
Prkar2b	2.3	down	42	19.4	-28129	intragenic
Prkar2b	1.9	down	42	19.4	-28129	intragenic
Lrp6	1.8	down	33	14.5	-28949	intragenic
Csgalnact1	1.6	up	27	12.7	-29133	intragenic
Arhgap28	2.1	down	46	21.1	-29167	intragenic
Wwp2	1.6	down	43	13.3	29170	intragenic
Wwp2	5.1	down	43	13.3	29170	intragenic
Nt5dc3	2.0	down	17	7.9	29175	intragenic
Crim1	2.4	down	25	9.7	29271	intragenic
Sec24d	3.0	down	19	7.3	29368	intragenic
Ptprf	1.9	down	26	6.7	-29427	intragenic
Cdk8	1.9	down	257	9.5	29496	intragenic
Sema3d	2.8	down	16	7.3	29560	intragenic
Zfp521	2.0	down	21	6.7	-29727	intragenic
Clint1	3.6	down	100	49.1	29981	intragenic
Nfix	1.6	down	150	76.1	-30149	intragenic
Pcmtd1	1.8	down	22	9.1	30601	intragenic
Rala	1.7	down	23	9.1	-30988	intragenic
Dab1	1.8	down	66	22.4	32085	intragenic
Tbx5	1.9	up	22	8.5	32424	intragenic
Dlc1	4.2	down	45	14.8	-32702	intragenic
Wwp2	1.6	down	52	12.8	32761	intragenic
Wwp2	5.1	down	52	12.8	32761	intragenic
Tle3	1.9	up	84	30.5	33093	intragenic
Chic2	1.7	down	28	9.1	-33604	intragenic

Chic2	2.1	down	28	9.1	-33604	intragenic
Gulp1	5.8	down	43	13.2	33615	intragenic
Zfc3h1	1.6	down	15	7.3	33686	intragenic
Gas2	2.4	up	40	21.2	35267	intragenic
Gng12	1.9	down	21	7.9	35473	intragenic
Nfix	1.6	down	23	10.9	-35961	intragenic
Ptn	2.0	down	19	8.5	-35984	intragenic
Gramd3	3.3	down	18	9.7	36251	intragenic
Itga9	1.9	up	29	11.1	36360	intragenic
Wwp2	1.6	down	70	25.5	36561	intragenic
Wwp2	5.1	down	70	25.5	36561	intragenic
Lmcd1	2.6	down	29	7.6	37814	intragenic
Cdadc1	1.6	down	36	14.8	-37842	intragenic
Myh9	1.6	up	25	8.9	-38139	intragenic
Actn4	2.6	down	33	14.5	-38228	intragenic
Actn4	7.6	down	33	14.5	-38228	intragenic
Actn4	1.9	down	33	14.5	-38228	intragenic
Vgll4	1.7	down	75	33.2	-38328	intragenic
Sox5	3.3	down	21	7.3	-39689	intragenic
Sox5	5.0	down	21	7.3	-39689	intragenic
Sox5	8.5	down	21	7.3	-39689	intragenic
Ptprm	1.6	down	22	8.8	-39808	intragenic
A430107O13Rik	1.6	down	27	8.2	40661	intragenic
Spsb1	2.5	down	23	7.3	-41019	intragenic
Efnb2	2.0	up	198	104.2	-41035	intragenic
Tasp1	1.8	down	21	8.9	-41037	intragenic
Zfhx3	1.9	up	65	30.9	41132	intragenic
Cdon	1.8	down	47	12.7	41577	intragenic
Ddah1	2.7	down	27	12.7	42144	intragenic
Gmds	1.8	down	18	9.1	-42174	intragenic
Stk39	1.8	down	32	13.3	-42306	intragenic
Wwp2	1.6	down	27	9.7	42636	intragenic
Wwp2	5.1	down	27	9.7	42636	intragenic
Tspan18	2.8	down	61	10.6	-43174	intragenic
Hmcn1	3.7	down	20	9.7	-43258	intragenic
Sox5	3.3	down	26	11.5	-43317	intragenic
Sox5	5.0	down	26	11.5	-43317	intragenic
Sox5	8.5	down	26	11.5	-43317	intragenic
Runx1	2.5	down	48	17.5	-43376	intragenic
Rgs3	1.9	down	37	12.2	43789	intragenic
Syne2	1.6	up	20	9.7	44048	intragenic
Col9a1	19.6	down	32	11.5	44317	intragenic
Col9a1	4.8	down	32	11.5	44317	intragenic
Unc5c	1.5	down	99	44.8	44378	intragenic
Nedd4l	1.5	down	34	8.9	45637	intragenic
Map4k4	1.7	up	25	7.3	47007	intragenic
Cnot2	4.8	down	34	13.3	-47081	intragenic
Lrch1	1.6	down	30	11.7	-48070	intragenic
Ccdc109a	4.1	down	49	21.2	-50376	intragenic
Sox6	3.3	down	30	6.7	-50461	intragenic

Sox6	1.8	down	30	6.7	-50461	intragenic
Zfp827	2.6	up	29	11.3	50988	intragenic
Nedd4l	1.5	down	20	9.7	51011	intragenic
Rbms1	2.0	down	19	8.5	-51879	intragenic
Frmd6	1.7	up	19	7.8	52459	intragenic
Col9a1	19.6	down	28	14.5	53031	intragenic
Col9a1	4.8	down	28	14.5	53031	intragenic
Tssc1	1.8	down	31	16.4	53576	intragenic
Tssc1	2.5	down	31	16.4	53576	intragenic
Tssc1	5.0	down	31	16.4	53576	intragenic
Nfatc1	2.4	down	16	6.0	-53853	intragenic
Nfatc1	2.0	down	16	6.0	-53853	intragenic
Nfatc1	3.1	down	16	6.0	-53853	intragenic
Nfatc1	1.6	down	16	6.0	-53853	intragenic
Bbs9	1.6	down	27	8.5	54130	intragenic
Ift57	1.7	up	52	13.8	54188	intragenic
Fbn1	2.0	up	19	9.1	-54534	intragenic
Rgs3	1.9	down	39	19.7	54619	intragenic
Wwp2	1.6	down	37	12.2	55153	intragenic
Wwp2	5.1	down	37	12.2	55153	intragenic
Glt25d2	1.9	down	28	12.1	55567	intragenic
Cdk6	1.8	down	32	15.2	55999	intragenic
Spsb4	1.8	down	123	55.7	-56039	intragenic
Sorbs2	1.9	down	36	9.2	57026	intragenic
Pitpnc1	2.3	down	91	28.1	-57228	intragenic
Prickle1	3.7	down	26	10.5	-58496	intragenic
Sema3d	2.8	down	23	9.7	59279	intragenic
Camk2d	1.7	down	18	9.1	59473	intragenic
Etv1	4.3	down	29	7.3	60164	intragenic
Etv1	3.9	down	29	7.3	60164	intragenic
Sema6a	1.7	down	17	6.7	-60268	intragenic
Erg	2.0	down	27	7.3	-60517	intragenic
Akap9	1.6	up	22	9.7	61567	intragenic
Mef2c	1.6	down	22	8.5	62045	intragenic
Myo1e	2.6	up	29	14.5	62199	intragenic
Col14a1	2.2	down	21	6.7	62479	intragenic
Tgfb2	2.0	down	38	12.1	-64650	intragenic
Sema3e	1.8	down	16	7.9	65519	intragenic
Pcca	2.2	down	45	20.6	65652	intragenic
Runx1	2.5	down	32	15.2	-66257	intragenic
Nfatc1	2.4	down	72	32.3	-67425	intragenic
Nfatc1	2.0	down	72	32.3	-67425	intragenic
Nfatc1	3.1	down	72	32.3	-67425	intragenic
Nfatc1	1.6	down	72	32.3	-67425	intragenic
Tox	4.6	down	30	13.9	-67933	intragenic
Lgr5	2.3	down	40	20.0	-68757	intragenic
Kirrel3	1.9	up	68	36.4	69351	intragenic
Pdzrn3	1.7	up	30	12.7	-69648	intragenic
Flnb	2.5	down	36	10.9	71572	intragenic
Vcan	5.2	down	28	12.7	-73154	intragenic

Myo1e	2.6	up	16	8.5	74220	intragenic
Zfpm2	1.9	down	33	8.5	75280	intragenic
Nfatc1	2.4	down	42	9.1	-76227	intragenic
Nfatc1	2.0	down	42	9.1	-76227	intragenic
Nfatc1	3.1	down	42	9.1	-76227	intragenic
Nfatc1	1.6	down	42	9.1	-76227	intragenic
Prickle1	3.7	down	33	11.5	-77139	intragenic
Sh3rf1	1.5	up	48	21.2	77693	intragenic
Gmds	1.8	down	17	7.9	-77890	intragenic
Flnb	2.5	down	93	47.3	78968	intragenic
Il1rap	1.7	down	27	9.7	80907	intragenic
Unc5c	1.5	down	22	9.1	81900	intragenic
Fgfr2	4.8	down	100	39.4	-82724	intragenic
Fgfr2	1.8	down	100	39.4	-82724	intragenic
Fgfr2	1.7	down	100	39.4	-82724	intragenic
Slit3	2.0	up	29	12.7	82904	intragenic
2900011O08Rik	2.6	down	33	11.7	84070	intragenic
Fign	2.1	down	24	7.8	-85837	intragenic
Fbn1	2.0	up	26	13.9	-86103	intragenic
Tanc1	1.7	up	40	21.2	86532	intragenic
Pitpnc1	2.3	down	18	9.1	-90267	intragenic
Rere	1.9	up	25	7.3	91065	intragenic
Myo1b	1.9	down	23	9.1	-93907	intragenic
Myo1b	2.1	down	23	9.1	-93907	intragenic
Nedd4l	1.5	down	52	20.1	94600	intragenic
Eps8	2.7	down	15	6.7	-95043	intragenic
Fbn2	1.8	up	35	15.8	-95091	intragenic
Lrch1	1.6	down	36	15.2	-95850	intragenic
Arhgap28	2.1	down	24	8.4	-96249	intragenic
Gli3	3.2	down	25	10.9	96366	intragenic
Camk2d	1.7	down	26	9.7	98645	intragenic
Mef2c	1.6	down	32	10.3	100355	intragenic
Mef2c	1.6	down	18	6.7	101322	intragenic
Ldb2	2.4	down	22	6.7	-101426	intragenic
Eya1	1.9	down	53	23.6	-101810	intragenic
Eya1	3.3	down	53	23.6	-101810	intragenic
Eya1	4.3	down	53	23.6	-101810	intragenic
Trio	1.6	down	15	6.7	-102087	intragenic
Trio	1.6	down	15	6.7	-102087	intragenic
Chst11	1.7	down	25	10.9	103198	intragenic
Thsd4	2.3	up	65	26.0	-103472	intragenic
Fmnl2	2.7	down	25	7.9	106756	intragenic
Fmnl2	5.0	down	25	7.9	106756	intragenic
Mpdz	1.7	down	24	11.5	-109328	intragenic
Mpdz	2.1	down	24	11.5	-109328	intragenic
Epha4	1.7	up	38	9.6	-109909	intragenic
Ldb2	2.4	down	28	10.7	-110572	intragenic
Pitpnc1	2.3	down	27	8.1	-111854	intragenic
Sema6a	1.7	down	40	9.1	-112860	intragenic
Pdzrn3	1.7	up	29	12.1	-114273	intragenic

Col23a1	1.7	up	22	5.6	116729	intragenic
Col23a1	1.5	up	22	5.6	116729	intragenic
Ror1	2.0	down	47	8.6	119057	intragenic
Fmnl2	2.7	down	19	5.8	119242	intragenic
Fmnl2	5.0	down	19	5.8	119242	intragenic
Zfp521	2.0	down	20	8.5	-119486	intragenic
Akap13	1.6	up	19	6.1	119844	intragenic
Sobp	1.8	down	32	6.7	-120499	intragenic
Sorbs2	1.9	down	58	11.0	121177	intragenic
Erg	2.0	down	41	15.2	-121972	intragenic
Gphn	1.9	up	283	8.7	124349	intragenic
Fmnl2	2.7	down	38	16.4	125210	intragenic
Fmnl2	5.0	down	38	16.4	125210	intragenic
Sox5	3.3	down	23	7.3	-127104	intragenic
Sox5	5.0	down	23	7.3	-127104	intragenic
Sox5	8.5	down	23	7.3	-127104	intragenic
Sox6	3.3	down	34	8.5	-129108	intragenic
Sox6	1.8	down	34	8.5	-129108	intragenic
Erg	2.0	down	51	19.4	-131307	intragenic
Lphn3	1.7	down	21	8.5	134615	intragenic
Lphn3	1.8	down	21	8.5	134615	intragenic
Unc5c	1.5	down	35	16.4	135888	intragenic
Hlcs	1.6	up	17	7.9	-139481	intragenic
Pbx3	2.5	down	28	13.3	-142238	intragenic
Chst11	1.7	down	98	42.8	145338	intragenic
Chst11	1.7	down	22	10.3	149578	intragenic
Fat3	2.4	up	21	11.5	-150058	intragenic
Fat3	1.7	up	21	11.5	-150058	intragenic
Tcf12	2.0	down	54	19.3	-151951	intragenic
Sox5	3.3	down	115	29.5	-152701	intragenic
Sox5	5.0	down	115	29.5	-152701	intragenic
Sox5	8.5	down	115	29.5	-152701	intragenic
Edil3	1.7	down	22	10.9	154815	intragenic
Chst11	1.7	down	27	8.3	154951	intragenic
Unc5c	1.5	down	34	9.7	156009	intragenic
Gmds	1.8	down	101	36.6	-158526	intragenic
Chst11	1.7	down	71	28.5	160576	intragenic
Tox	4.6	down	21	7.9	-166430	intragenic
Sox5	3.3	down	77	40.0	-170170	intragenic
Sox5	5.0	down	77	40.0	-170170	intragenic
Sox5	8.5	down	77	40.0	-170170	intragenic
Sox6	3.3	down	23	8.5	-170347	intragenic
Sox6	1.8	down	23	8.5	-170347	intragenic
Col25a1	1.6	down	27	11.7	179816	intragenic
Col25a1	1.9	down	27	11.7	179816	intragenic
Pcca	2.2	down	47	26.7	180695	intragenic
Palld	2.4	up	27	11.5	-193471	intragenic
Grid2	2.6	up	20	7.9	195928	intragenic
Lphn3	1.7	down	29	10.9	197334	intragenic
Lphn3	1.8	down	29	10.9	197334	intragenic

Lphn3	1.7	down	26	12.7	204022	intragenic
Lphn3	1.8	down	26	12.7	204022	intragenic
Kif26b	1.9	down	18	7.9	204050	intragenic
Ptprm	1.6	down	36	4.8	-220540	intragenic
Sox5	3.3	down	77	38.8	-228325	intragenic
Sox5	5.0	down	77	38.8	-228325	intragenic
Sox5	8.5	down	77	38.8	-228325	intragenic
Robo1	1.6	down	27	12.1	234077	intragenic
Odz2	1.6	up	21	10.9	-239964	intragenic
Pcdh7	1.5	up	20	9.1	245039	intragenic
Pcdh7	1.9	up	20	9.1	245039	intragenic
Kif26b	1.9	down	99	34.4	292121	intragenic
Lphn3	1.7	down	45	20.6	302955	intragenic
Lphn3	1.8	down	45	20.6	302955	intragenic
Utrn	3.5	down	30	14.5	-307401	intragenic
Sox6	3.3	down	19	7.3	-325647	intragenic
Sox6	1.8	down	19	7.3	-325647	intragenic
Sox6	3.3	down	104	26.1	-370045	intragenic
Sox6	1.8	down	104	26.1	-370045	intragenic
Sox6	3.3	down	67	15.2	-379120	intragenic
Sox6	1.8	down	67	15.2	-379120	intragenic
Diap2	1.8	up	14	7.3	397199	intragenic
Sox6	3.3	down	19	6.1	-520829	intragenic
Sox6	1.8	down	19	6.1	-520829	intragenic
Lrp1b	1.7	up	22	9.1	-645791	intragenic
Bsdc1	2.2	up	49	19.7	-10008	distal
Rasl11b	4.4	down	35	15.7	10387	distal
Tuba3b	1.9	up	20	8.5	-10444	distal
Phlda1	2.2	down	20	7.3	10888	distal
Fam101a	3.0	down	24	11.1	-10888	distal
Dkk3	1.9	down	22	10.3	11355	distal
Nup214	1.8	up	37	12.2	-11614	distal
Snx7	2.2	down	32	13.9	11654	distal
Snx7	1.7	down	32	13.9	11654	distal
Fbln2	2.3	down	42	11.4	-11670	distal
9230105E05Rik	1.9	up	25	10.3	-12020	distal
Pabpc1	1.7	down	28	10.9	12058	distal
Pabpc1	2.2	down	28	10.9	12058	distal
Pabpc1	3.0	down	28	10.9	12058	distal
Vasn	1.8	down	52	22.4	12062	distal
1110034A24Rik	1.7	down	41	13.3	12222	distal
4930402H24Rik	1.5	up	23	6.7	12223	distal
2410004N09Rik	1.6	up	21	9.7	12228	distal
Zfp687	2.2	up	18	8.5	-12467	distal
Thbs1	5.5	down	20	10.9	-12736	distal
Thbs1	5.3	down	20	10.9	-12736	distal
Tanc1	1.7	up	32	9.9	-13091	distal
Fzd9	1.9	down	84	35.7	13312	distal
Ilf3	1.8	down	22	7.3	-13328	distal
Sh3kbp1	2.3	down	31	17.0	-13415	distal

Gorasp2	3.2	down	18	6.7	-13683	distal
Gorasp2	5.7	down	18	6.7	-13683	distal
Por	1.7	up	25	10.3	-14218	distal
Eapp	2.1	up	65	8.1	14564	distal
Susd5	1.6	down	22	8.5	-14679	distal
Ncf2	7.1	up	38	11.2	-14870	distal
Il17d	1.6	down	38	12.4	-15048	distal
Ppic	2.1	down	20	7.2	15469	distal
1500015O10Rik	1.8	down	20	10.3	-15528	distal
Tpp1	2.0	up	23	8.5	15535	distal
Spg20	1.9	down	22	7.9	-15571	distal
Lect1	1.6	down	48	15.8	16011	distal
Rasl11b	4.4	down	20	4.9	16069	distal
Bbs12	1.7	down	20	6.7	16124	distal
Dab2	1.6	up	28	12.7	-16143	distal
Amotl2	1.5	down	29	13.3	-16618	distal
Plce1	1.8	down	197	100.6	-16793	distal
Rbpjl	3.0	down	71	28.5	-16878	distal
Lrmp	2.6	up	26	7.5	-17370	distal
Fam181b	1.7	down	44	18.8	-17623	distal
Zfp521	2.0	down	26	7.8	18394	distal
Hapln1	6.7	down	27	9.7	-19234	distal
Lrrc8d	1.5	down	40	16.1	-19358	distal
Dusp6	1.7	down	17	7.9	-19445	distal
Dab2	1.6	up	21	9.1	-19473	distal
Rasl11b	4.4	down	39	11.0	-19654	distal
Col25a1	1.6	down	29	10.9	-19790	distal
Col25a1	1.9	down	29	10.9	-19790	distal
Bmpr1a	4.2	down	17	7.6	20254	distal
Tbx4	1.6	up	107	59.4	20426	distal
Kcns1	2.1	down	81	10.7	-20446	distal
Lonp2	1.7	up	26	7.5	-20578	distal
Foxf1a	1.6	down	37	13.9	-20658	distal
Tle3	1.9	up	25	8.5	-21070	distal
Capn6	2.1	down	22	8.5	21090	distal
Gls	3.1	down	23	6.7	21295	distal
Igf2bp3	1.5	up	92	14.6	21558	distal
Col2a1	8.1	down	18	7.3	22031	distal
Col2a1	1.9	down	18	7.3	22031	distal
Col2a1	4.8	down	18	7.3	22031	distal
Fam20b	1.7	down	18	9.5	22289	distal
Prdx6	1.9	up	32	14.5	22460	distal
Bcas2	1.9	up	65	26.7	22574	distal
Rarb	2.4	down	26	7.3	22706	distal
Rarb	2.6	down	26	7.3	22706	distal
Lmnb1	6.8	down	17	6.7	-22886	distal
Lmnb1	3.9	down	17	6.7	-22886	distal
Col2a1	8.1	down	41	14.5	22907	distal
Col2a1	1.9	down	41	14.5	22907	distal
Col2a1	4.8	down	41	14.5	22907	distal

Dact1	2.2	down	16	7.9	22980	distal
Dact1	2.2	down	16	7.9	22980	distal
Hopx	2.0	down	37	17.6	23079	distal
Ptpn23	2.7	up	22	9.7	-23485	distal
Myst4	1.9	up	44	15.2	-23586	distal
Myst4	2.0	up	44	15.2	-23586	distal
Tns4	1.8	up	130	50.7	23995	distal
Synpo	1.9	down	25	9.7	24184	distal
Synpo	1.7	down	25	9.7	24184	distal
Rcl1	3.0	down	45	20.6	-24507	distal
Six2	2.7	down	16	6.6	24893	distal
Gjb6	1.6	down	25	9.4	25047	distal
Laptm4b	2.5	up	37	13.3	-25342	distal
Fzd9	1.9	down	23	6.6	25544	distal
Clic4	3.7	down	28	13.8	25613	distal
Clic4	4.7	down	28	13.8	25613	distal
Tnrc6a	1.6	up	37	8.9	-25653	distal
Slc14a1	2.2	down	36	18.8	-26052	distal
Slc14a1	1.9	down	36	18.8	-26052	distal
Slc14a1	1.7	down	36	18.8	-26052	distal
Col2a1	8.1	down	25	8.3	26359	distal
Col2a1	1.9	down	25	8.3	26359	distal
Col2a1	4.8	down	25	8.3	26359	distal
Olig1	2.6	down	31	12.7	26819	distal
Lyl1	2.0	up	22	8.5	27125	distal
Tgfbi	2.3	down	49	22.1	-27320	distal
Dnajb6	3.0	down	33	11.1	-27368	distal
Tmbim6	1.6	up	34	17.0	27441	distal
4930405A21Rik	1.7	down	28	14.5	-27509	distal
9230105E05Rik	1.9	up	32	9.7	27819	distal
3110079O15Rik	4.8	down	21	8.5	-27822	distal
Cntn1	1.7	down	21	7.9	-28201	distal
Dct	1.6	down	41	20.0	28367	distal
Dct	1.7	down	41	20.0	28367	distal
1110058L19Rik	1.5	down	32	14.5	28546	distal
Zfp825	1.8	down	19	6.1	28806	distal
Kcns1	2.1	down	47	16.4	-28806	distal
Actr10	3.9	down	15	8.5	-29105	distal
Fgfr3	1.8	down	23	8.5	-29252	distal
Fgfr3	5.1	down	23	8.5	-29252	distal
Cdkn2aip	1.7	up	41	9.7	29339	distal
Gfpt2	2.0	down	25	9.1	-29344	distal
Gfpt2	1.5	down	25	9.1	-29344	distal
Bmf	2.1	up	128	53.9	-29412	distal
Dnm3os	2.1	down	28	5.5	-29737	distal
Dact1	2.2	down	18	6.1	-29891	distal
Dact1	2.2	down	18	6.1	-29891	distal
Chst11	1.7	down	63	10.0	-30180	distal
Tmem17	1.6	down	22	7.3	-30490	distal
Angptl1	2.2	down	22	9.4	-30500	distal

Ndufb4	2.3	up	30	10.0	30582	distal
Tmed3	2.5	down	26	10.9	-30705	distal
Itgb1	1.7	down	28	7.9	-30899	distal
Plekha1	2.0	down	37	10.1	-31372	distal
Postn	4.6	down	26	11.5	31575	distal
Sox11	3.1	up	26	7.4	-31706	distal
Sox11	1.6	up	26	7.4	-31706	distal
Acan	3.7	down	34	11.6	-31905	distal
Acan	6.2	down	34	11.6	-31905	distal
Lats2	1.7	down	37	18.8	-32385	distal
Isca2	2.8	up	23	11.5	32474	distal
Dok1	2.9	down	36	9.1	-33186	distal
Dok1	2.8	down	36	9.1	-33186	distal
Lonrf1	2.0	down	36	17.0	33744	distal
Sars	1.6	up	37	13.9	33828	distal
Stmn4	1.6	down	91	38.2	34113	distal
Dab2ip	1.8	down	24	9.7	-34295	distal
Acpl2	1.5	down	37	12.1	34327	distal
Acpl2	2.5	down	37	12.1	34327	distal
Dnajc24	2.6	down	34	10.9	34824	distal
Slc4a3	1.9	up	44	17.0	35828	distal
Mylk	1.9	up	18	7.3	-35871	distal
Rasl11b	4.4	down	34	7.0	-35998	distal
Amotl2	1.5	down	30	10.3	-36074	distal
Erbb2ip	2.3	down	23	9.4	36863	distal
Vcan	5.2	down	56	15.4	36872	distal
Myst4	1.9	up	33	15.2	-37108	distal
Myst4	2.0	up	33	15.2	-37108	distal
Unc13c	1.8	up	36	15.5	37361	distal
Maml2	1.6	down	23	10.3	-38588	distal
Fam101a	3.0	down	121	57.6	-39196	distal
Mtus1	1.7	up	17	6.7	39845	distal
Papss2	3.1	down	33	11.5	-40089	distal
Papss2	6.5	down	33	11.5	-40089	distal
Slc25a4	2.1	up	22	9.1	40226	distal
Asns	3.1	down	35	12.7	40396	distal
Runx1	2.5	down	51	24.1	41890	distal
Vgll4	1.7	down	70	29.1	42065	distal
Cpm	1.5	up	33	4.6	-42508	distal
Sgk1	1.6	down	30	14.5	-42661	distal
Ndst1	1.8	up	40	11.6	-42708	distal
Kcnj2	2.3	down	42	17.6	-43067	distal
Ndst1	1.8	up	32	12.8	-43661	distal
Dab2ip	1.8	down	76	27.4	-44242	distal
Fstl1	30.5	down	27	8.6	-44745	distal
Plce1	1.8	down	46	14.2	-45129	distal
Cdon	1.8	down	41	13.3	-46199	distal
Zmynd11	2.9	down	19	8.5	46827	distal
Zmynd11	1.9	down	19	8.5	46827	distal
Zmynd11	3.2	down	19	8.5	46827	distal

Krr1	3.1	down	204	100.6	-47067	distal
Has2	2.1	down	22	9.7	47673	distal
Tnfrsf21	1.8	down	26	8.5	-47759	distal
C030044B11Rik	1.8	down	44	7.6	48295	distal
Cdc42ep3	2.5	down	26	9.1	-48950	distal
Cdc42ep3	1.7	down	26	9.1	-48950	distal
Tlr13	2.0	up	23	10.3	-49440	distal
Flrt1	1.9	up	31	11.2	49692	distal
Prrc1	1.7	down	58	27.4	50562	distal
Foxp4	1.5	up	47	21.2	51372	distal
Tgif1	1.8	down	67	30.3	-52307	distal
Tgif1	3.4	down	67	30.3	-52307	distal
Cyr61	3.3	down	39	5.6	53748	distal
Scrg1	1.9	down	24	10.3	-54069	distal
Jun	1.8	up	19	8.5	54100	distal
Jdp2	1.6	down	17	8.5	-54199	distal
Fzd5	1.7	down	28	7.9	54531	distal
Sox5	3.3	down	94	28.3	54771	distal
Sox5	5.0	down	94	28.3	54771	distal
Sox5	8.5	down	94	28.3	54771	distal
Dact1	2.2	down	24	7.3	-54999	distal
Dact1	2.2	down	24	7.3	-54999	distal
Susd5	1.6	down	21	6.1	55132	distal
1190002N15Rik	4.5	down	53	25.5	-55254	distal
Nog	4.0	down	41	7.9	56284	distal
Erg	2.0	down	23	9.1	56489	distal
Spred1	2.0	down	26	10.9	-56681	distal
9030625A04Rik	2.3	down	25	7.7	-57053	distal
BC027231	1.6	down	29	9.1	-57670	distal
BC027231	1.6	down	34	7.8	-58660	distal
Rars	1.7	down	27	10.6	58733	distal
Dlc1	4.2	down	29	11.5	-58859	distal
Psat1	2.6	down	31	15.5	-58952	distal
Usp47	1.6	down	21	9.7	-60141	distal
Psat1	2.6	down	23	8.3	-60239	distal
Spsb1	2.5	down	47	13.9	-60925	distal
Glul	2.3	up	23	10.9	62421	distal
Acan	3.7	down	43	11.5	-62978	distal
Acan	6.2	down	43	11.5	-62978	distal
4930545L23Rik	1.6	down	21	8.3	-63572	distal
Ppp2r2a	6.4	down	33	13.9	63710	distal
Itga11	1.5	down	63	19.6	-64624	distal
Tgif1	1.8	down	20	7.9	-65585	distal
Tgif1	3.4	down	20	7.9	-65585	distal
Cep78	2.0	down	17	8.5	65815	distal
Etv5	1.7	down	31	13.3	-66594	distal
Gcnt1	1.9	down	51	26.7	-68102	distal
Gcnt1	2.2	down	51	26.7	-68102	distal
Stk32a	1.6	down	24	6.7	-68580	distal
Prg4	1.6	down	20	7.3	68770	distal

Zmynd11	2.9	down	233	8.4	69026	distal
Zmynd11	1.9	down	233	8.4	69026	distal
Zmynd11	3.2	down	233	8.4	69026	distal
Ppic	2.1	down	71	18.7	-69736	distal
Etv5	1.7	down	27	11.5	69776	distal
Apip	2.1	down	31	12.1	70814	distal
Rasl11b	4.4	down	48	15.8	70945	distal
Cyp26b1	2.6	down	32	15.8	71307	distal
Fndc3b	2.3	down	33	10.9	72010	distal
Smyd2	2.5	down	25	7.8	-72521	distal
Ect2	2.2	down	29	13.3	-72554	distal
Map4k3	1.6	down	37	12.8	72891	distal
Rab11a	3.3	down	63	24.4	-73033	distal
Hs1bp3	1.8	down	47	22.4	73080	distal
Hs1bp3	2.2	down	47	22.4	73080	distal
Frmd6	1.7	up	23	9.9	-73149	distal
Bcor	1.6	down	49	27.9	-73685	distal
Fign	2.1	down	40	18.2	74487	distal
Lbh	1.9	down	19	9.1	75475	distal
Tmem17	1.6	down	20	6.7	75656	distal
Uncx	1.7	up	51	15.8	75869	distal
Fndc3b	2.3	down	27	9.1	76291	distal
Mrpl13	1.6	up	25	10.1	-77520	distal
Kcnk1	1.8	down	70	35.7	78288	distal
Glul	2.3	up	42	17.0	78531	distal
Lsm6	1.5	up	47	21.8	-78549	distal
Snx2	6.8	down	20	7.9	-78797	distal
Snx2	5.2	down	20	7.9	-78797	distal
Elac2	1.9	down	27	8.3	-79005	distal
Col11a1	3.1	down	17	7.9	-79864	distal
Col11a1	2.1	down	17	7.9	-79864	distal
Ptprz1	1.9	down	24	8.5	-80998	distal
Ptprz1	2.0	down	24	8.5	-80998	distal
Micall2	1.7	up	19	7.9	-81594	distal
Postn	4.6	down	52	22.7	82961	distal
Sesn3	2.5	down	121	33.1	-83392	distal
Prrc1	1.7	down	56	29.7	-83518	distal
Fam114a2	1.7	up	31	16.4	-84294	distal
Umps	7.8	down	29	12.7	84423	distal
Foxf2	2.1	up	30	12.1	85166	distal
Trib2	1.6	up	49	22.4	-85308	distal
St3gal1	1.6	down	28	8.5	-86093	distal
Phlda1	2.2	down	37	14.1	86708	distal
Tle3	1.9	up	24	9.4	-87198	distal
Glcci1	2.1	down	22	10.3	88205	distal
Glcci1	1.5	down	22	10.3	88205	distal
Gmds	1.8	down	23	6.7	88420	distal
Snx2	6.8	down	19	7.5	-88618	distal
Snx2	5.2	down	19	7.5	-88618	distal
Dact1	2.2	down	22	5.9	88891	distal

Dact1	2.2	down	22	5.9	88891	distal
Sep-10	1.9	down	18	6.1	-89744	distal
Dnm3os	2.1	down	79	12.0	-90636	distal
Yipf5	1.6	up	27	12.7	-91132	distal
Tgif1	1.8	down	24	7.3	-91154	distal
Tgif1	3.4	down	24	7.3	-91154	distal
Kank1	5.6	down	27	8.5	-91845	distal
Jun	1.8	up	30	8.1	92180	distal
Ndufa10	1.5	up	43	20.0	-94319	distal
Bbox1	1.5	down	17	7.3	-94849	distal
Pde7a	4.1	down	142	70.3	95057	distal
Pde7a	2.6	down	142	70.3	95057	distal
Pdzrn3	1.7	up	24	8.1	95516	distal
Robo2	1.7	up	45	17.7	95606	distal
Robo2	1.5	up	45	17.7	95606	distal
Cyld	2.4	up	22	9.1	95809	distal
Dach1	1.6	down	22	10.3	96131	distal
Pxdn	3.0	down	43	15.9	96797	distal
Psat1	2.6	down	44	9.1	-97184	distal
Tns3	1.7	down	44	15.0	97221	distal
Gdf10	2.2	down	46	13.0	-99068	distal
Mrpl13	1.6	up	48	22.4	-99897	distal
Dleu7	1.8	down	32	7.0	-99973	distal

Table S5 Sox5 (4 pages)

CHIPSeq_MACS_Peak_id	CHIPSeq_MACS_PeakTags	CHIPSeq_MACS_PeakFoldEnrichment	GenesSymbol	AbsoluteDistanceFromPeakToGene	ProbeID	ExpressionFoldChangeAbsolute	ExpressionRegulationDirection
MACS_PEAK_10	38	8.43	Stat4	11411	5560066	2.192642212	up
MACS_PEAK_79	51	19.94	Trim16	672	7550520	1.743749976	up
MACS_PEAK_253	20	6.65	Fam173a	2572	2030181	1.778350115	up
MACS_PEAK_201	18	6.04	Myo10	20199	1470035	1.700320125	up
MACS_PEAK_112	23	9.46	Rhob	113480	1710538	2.576362133	down
MACS_PEAK_98	33	5.15	Etv4	52287	270010	1.609756589	up
MACS_PEAK_374	23	9.06	Map3k7	39636	6860736	1.872915387	down
MACS_PEAK_375	19	5.58	Map3k7	216594	6860736	1.872915387	down
MACS_PEAK_359	22	8.71	Fmo5	3188	5220477	1.681067348	up
MACS_PEAK_223	20	7.25	Txndc11	9554	2030452	1.503304601	up
MACS_PEAK_625	18	6.65	Tgfbr2	209210	4200246	1.528139591	up
MACS_PEAK_626	17	7.86	Tgfbr2	295670	4200246	1.528139591	up
MACS_PEAK_72	25	5.91	Odz2	733896	7050408	2.188945532	down
MACS_PEAK_336	19	6.04	Dok5	104465	5270600	1.511301041	up
MACS_PEAK_336	19	6.04	Dok5	104465	1230474	1.709348679	up
MACS_PEAK_357	17	6.65	Gatad2b	13973	6400086	2.021556139	down
MACS_PEAK_592	19	7.86	Zfhx3	120483	3870068	2.569363832	down
MACS_PEAK_592	19	7.86	Zfhx3	120483	6580376	1.584697604	down
MACS_PEAK_280	16	6.58	Mapre2	189	6860543	1.696675301	down
MACS_PEAK_8	34	14.5	Fhl2	194366	2350019	1.584973693	up
MACS_PEAK_459	22	8.09	Piwil1	62382	6270390	1.564337134	up
MACS_PEAK_523	61	10.11	Pde3a	276607	4070168	1.607645631	up
MACS_PEAK_22	20	6.04	Twist2	182470	6420739	1.793907762	up
MACS_PEAK_404	21	9.06	Ptpru	384315	3170735	2.041645288	up
MACS_PEAK_616	34	6.74	Tle3	246209	2710338	1.613103151	down
MACS_PEAK_56	26	5.81	Fam13c	27369	4540474	1.620222092	up
MACS_PEAK_499	16	6.04	Tax1bp1	26636	840475	1.91344583	down
MACS_PEAK_500	19	7.1	Tax1bp1	81665	840475	1.91344583	down
MACS_PEAK_236	30	12.42	Robo1	570544	2760343	1.873947024	down
MACS_PEAK_506	34	8.78	Dysf	115478	1450068	1.529547811	up
MACS_PEAK_506	34	8.78	Dysf	115478	7610398	1.574743271	up
MACS_PEAK_263	30	6.29	Rftn1	239810	5560349	1.694047093	up
MACS_PEAK_470	18	6.04	Sdk1	505041	380463	2.000249386	up
MACS_PEAK_106	25	6.04	2610035D17Rik	13238	1090044	1.550805688	down
MACS_PEAK_107	18	6.04	2610035D17Rik	19825	1090044	1.550805688	down
MACS_PEAK_465	17	5.44	Cux1	66960	5820465	1.760463476	down
MACS_PEAK_567	18	5.85	Ppapdc1b	30489	770746	1.798686862	up
MACS_PEAK_619	18	9.06	Cgnl1	880	5570736	1.691678286	up
MACS_PEAK_112	23	9.46	Hs1bp3	73072	520711	1.551209569	up
MACS_PEAK_334	26	7.45	Bcas1	119591	6560347	1.657806396	down
MACS_PEAK_490	150	6.87	Smo	13391	2810440	1.552044272	up
MACS_PEAK_401	20	7.78	Gja4	4784	770672	2.194069386	up
MACS_PEAK_528	21	8.46	Sox5	268126	4880553	4.62940979	down
MACS_PEAK_529	21	7.25	Sox5	170042	4880553	4.62940979	down
MACS_PEAK_530	29	12.69	Sox5	166366	4880553	4.62940979	down
MACS_PEAK_531	76	28.21	Sox5	584	4880553	4.62940979	down
MACS_PEAK_532	38	16.5	Sox5	55278	4880553	4.62940979	down
MACS_PEAK_533	23	6.39	Sox5	250705	4880553	4.62940979	down
MACS_PEAK_528	21	8.46	Sox5	268126	5050082	6.362275124	down
MACS_PEAK_529	21	7.25	Sox5	170042	5050082	6.362275124	down
MACS_PEAK_530	29	12.69	Sox5	166366	5050082	6.362275124	down
MACS_PEAK_531	76	28.21	Sox5	584	5050082	6.362275124	down
MACS_PEAK_532	38	16.5	Sox5	55278	5050082	6.362275124	down
MACS_PEAK_533	23	6.39	Sox5	250705	5050082	6.362275124	down
MACS_PEAK_528	21	8.46	Sox5	268126	6020273	2.341292381	down
MACS_PEAK_529	21	7.25	Sox5	170042	6020273	2.341292381	down
MACS_PEAK_530	29	12.69	Sox5	166366	6020273	2.341292381	down
MACS_PEAK_531	76	28.21	Sox5	584	6020273	2.341292381	down
MACS_PEAK_532	38	16.5	Sox5	55278	6020273	2.341292381	down
MACS_PEAK_533	23	6.39	Sox5	250705	6020273	2.341292381	down
MACS_PEAK_189	17	5.44	Itm2b	61269	2970286	1.63623178	up
MACS_PEAK_611	20	7.1	Sorl1	188534	6960577	1.710558295	down
MACS_PEAK_436	17	6.65	Phox2b	19237	6220736	1.636369348	up
MACS_PEAK_34	24	5.32	Dusp10	249166	2000494	1.548292041	up
MACS_PEAK_182	17	7.25	Gjb6	28275	610746	2.173983812	up
MACS_PEAK_440	18	6.65	Lphn3	1732	4260348	1.501995325	up
MACS_PEAK_441	18	6.65	Lphn3	481084	4260348	1.501995325	up
MACS_PEAK_58	17	6.04	Col18a1	2785	3450066	2.501227617	up
MACS_PEAK_58	17	6.04	Col18a1	2785	5690270	2.608283281	up
MACS_PEAK_258	210	5	Gpsm3	2898	3850221	1.547520876	up
MACS_PEAK_152	16	6.04	Neurog1	185	4610541	1.684467912	up
MACS_PEAK_363	15	8.46	Palmd	194182	1440300	2.522102594	up
MACS_PEAK_528	21	8.46	Sox5	268126	6200707	1.902192593	down
MACS_PEAK_529	21	7.25	Sox5	170042	6200707	1.902192593	down
MACS_PEAK_530	29	12.69	Sox5	166366	6200707	1.902192593	down
MACS_PEAK_531	76	28.21	Sox5	584	6200707	1.902192593	down
MACS_PEAK_532	38	16.5	Sox5	55278	6200707	1.902192593	down
MACS_PEAK_533	23	6.39	Sox5	250705	6200707	1.902192593	down
MACS_PEAK_387	18	6.04	4930473A06Rik	244121	7150079	1.749178767	up
MACS_PEAK_164	21	7.26	Cartpt	109674	4050706	1.640872955	up
MACS_PEAK_321	19	9.06	Nat10	54876	2120132	1.53066957	up
MACS_PEAK_327	28	12.42	Slc24a3	50414	4280131	1.602561235	up
MACS_PEAK_62	53	15.71	Chst11	57359	3390678	1.832973957	down
MACS_PEAK_66	17	5.92	Grip1	114771	3460382	1.532074928	down
MACS_PEAK_81	22	6.29	Pmp22	46693	2480129	1.897400498	up
MACS_PEAK_402	18	5.32	Zscan20	143775	3170601	1.565991044	up
MACS_PEAK_403	18	5.44	Matn1	77913	4280487	17.49271202	down
MACS_PEAK_23	15	7.86	Lypd1	73669	6020358	2.41830349	up
MACS_PEAK_55	21	6.65	Ank3	17941	6020176	2.969679832	down
MACS_PEAK_54	17	5.86	Ank3	134939	6020176	2.969679832	down
MACS_PEAK_407	24	7.25	Clic4	50556	3400491	1.885798573	down
MACS_PEAK_394	17	6.65	Ssbp3	45861	1340349	1.716467619	down
MACS_PEAK_492	39	18.73	Chchd3	237	7550743	1.594190598	up
MACS_PEAK_522	26	9.06	Atf7ip	48344	5690451	1.804197907	down
MACS_PEAK_269	17	6.65	Dlgap1	72117	6250102	1.693743587	up
MACS_PEAK_42	19	7.25	Map3k5	33465	5550112	1.780693173	up

MACS_PEAK_88	36	9.06	Tbx2	153812	670367	1.751884937	up
MACS_PEAK_264	16	8.03	Efna5	18358	6840577	1.599610806	up
MACS_PEAK_265	24	7.1	Efna5	286000	6840577	1.599610806	up
MACS_PEAK_612	34	9.46	Mll1	84	2100364	2.11644125	down
MACS_PEAK_333	19	7.86	Atp9a	28071	670414	1.946009755	up
MACS_PEAK_333	19	7.86	Atp9a	28071	1510364	1.845288873	up
MACS_PEAK_212	18	6.51	Mpped1	17542	430296	1.53749311	up
MACS_PEAK_117	39	15.71	Rnf144a	1166967	5690609	1.957864404	down
MACS_PEAK_277	16	7.25	Arhgap12	696	5340156	1.598216772	up
MACS_PEAK_35	33	11.24	Tgfb2	64805	770754	4.134972572	down
MACS_PEAK_64	20	5.32	Frs2	14371	7560195	1.629254341	up
MACS_PEAK_199	15	7.25	Pdzd2	12273	6350241	1.697885036	up
MACS_PEAK_53	19	6.04	Rtkn2	116971	1300438	1.669357896	up
MACS_PEAK_67	22	7.1	Hmga2	979	160270	2.009763241	down
MACS_PEAK_68	27	10.65	Hmga2	76102	160270	2.009763241	down
MACS_PEAK_443	32	5.7	G3bp2	49	4230681	2.240312576	down
MACS_PEAK_28	17	6.65	Rgs16	80463	3890528	1.761289358	up
MACS_PEAK_508	26	8.71	Tia1	377	3060544	1.673606753	up
MACS_PEAK_152	16	6.04	Cxcl14	44573	3520349	1.843580723	up
MACS_PEAK_241	16	6.04	Setd4	24083	3120605	1.749804497	up
MACS_PEAK_288	19	7.25	Tcf4	367903	7550360	1.630245209	down
MACS_PEAK_384	21	9.67	Olfr267	62845	3780519	1.562190294	up
MACS_PEAK_349	20	6.78	Foxo1	71776	6580682	2.203903913	up
MACS_PEAK_518	24	10.27	Ing4	3	6550408	1.596772075	up
MACS_PEAK_548	22	7.76	Sox6	623788	4070386	2.446631193	down
MACS_PEAK_549	33	13.9	Sox6	379306	4070386	2.446631193	down
MACS_PEAK_377	18	6.51	Glipr2	7471	4120687	1.686236024	up
MACS_PEAK_510	26	9.58	Magi1	664	2760328	1.938788414	up
MACS_PEAK_511	19	5.12	Magi1	100151	2760328	1.938788414	up
MACS_PEAK_512	37	10.65	Magi1	90	2760328	1.938788414	up
MACS_PEAK_119	21	7.25	Egln3	26981	2750326	1.56449306	down
MACS_PEAK_41	22	6.87	Cited2	648270	6020379	1.539243817	down
MACS_PEAK_41	22	6.87	Cited2	648270	5860538	1.61139214	down
MACS_PEAK_158	18	7.86	2010111I01Rik	21044	3520092	1.647946477	up
MACS_PEAK_118	17	6.65	Sox11	233059	990681	2.488309383	down
MACS_PEAK_568	21	7.86	Zfp703	202015	380717	1.685684443	up
MACS_PEAK_354	17	6.04	Fstl5	20	2340192	4.335448742	down
MACS_PEAK_214	21	7.1	Phf21b	23918	5900551	1.757664919	down
MACS_PEAK_223	20	7.25	Txndc11	9554	3390343	1.518350482	up
MACS_PEAK_2	17	5.44	B3gat2	40292	4920474	1.510452867	up
MACS_PEAK_273	24	7.02	Ppm1b	9473	7650044	1.824744344	up
MACS_PEAK_87	17	5.44	Rab34	630	1780025	1.829192758	up
MACS_PEAK_363	15	8.46	Palmd	194182	5080279	2.123199224	up
MACS_PEAK_11	19	7.45	Nab1	100	1300709	1.537516475	up
MACS_PEAK_11	19	7.45	Nab1	100	730736	1.925327063	up
MACS_PEAK_175	33	9.45	Zmiz1	81032	5670523	2.562799931	down
MACS_PEAK_176	27	8.46	Zmiz1	1556	5670523	2.562799931	down
MACS_PEAK_174	23	5.44	Zmiz1	572451	5670523	2.562799931	down
MACS_PEAK_389	18	9.06	Cdkn2b	179838	5550240	1.740302086	up
MACS_PEAK_159	46	4.45	Irx2	67017	2970008	1.736776829	up
MACS_PEAK_109	24	6.37	Tmc6	31304	7100300	2.330271721	up
MACS_PEAK_109	24	6.37	Tmc6	31304	5490079	2.286953688	up
MACS_PEAK_109	24	6.37	Tmc6	31304	7570475	2.202062845	up
MACS_PEAK_128	20	6.04	Galc	1428224	1500201	1.791874766	up
MACS_PEAK_376	15	6.04	Cnr1	132	1440647	1.51412046	up
MACS_PEAK_272	19	7.45	Qpct	128797	990687	2.19092536	up
MACS_PEAK_518	24	10.27	Ing4	3	4210168	1.761608839	up
MACS_PEAK_558	29	13.9	Mgmt	551537	3890072	1.876641154	up
MACS_PEAK_94	35	12.42	Rara	21130	2940561	1.639087081	up
MACS_PEAK_134	16	5.44	Kif26a	18048	4070731	1.76064384	up
MACS_PEAK_152	16	6.04	Cxcl14	44573	150746	1.5744313	up
MACS_PEAK_268	17	6.51	Arhgap28	133	6100468	1.562302589	up
MACS_PEAK_43	22	9.06	Hbs1l	37866	50279	1.552370071	up
MACS_PEAK_142	22	5.92	Lyst	164966	4640102	1.550898194	up
MACS_PEAK_226	33	12.09	Leprel1	17926	6330086	1.809220314	up
MACS_PEAK_577	47	12.87	Ednra	12919	4040189	2.376912355	up
MACS_PEAK_453	35	9.06	Ttc28	27266	2370762	3.188261271	down
MACS_PEAK_279	18	7.25	Asxl3	1110	4230167	3.112105608	down
MACS_PEAK_422	33	12.62	Arhgef16	13557	4850719	1.68380928	up
MACS_PEAK_113	16	4.83	Laptm4a	77529	5560411	2.131327391	up
MACS_PEAK_388	27	8.52	Mllt3	112487	1110112	1.572801113	down
MACS_PEAK_522	26	9.06	Atf7ip	48344	4570709	3.180575371	down
MACS_PEAK_471	21	5.13	Foxk1	33563	1510273	1.6241889	up
MACS_PEAK_242	20	6.51	Dscr3	16458	4280768	1.569319129	up
MACS_PEAK_7	27	6.29	2010300C02Rik	32099	3370465	1.583681583	up
MACS_PEAK_90	22	8.46	Rad51c	18675	3360253	1.950005174	down
MACS_PEAK_91	24	5.77	Rad51c	39159	3360253	1.950005174	down
MACS_PEAK_127	17	7.86	1700020O03Rik	126525	5910056	2.101079941	down
MACS_PEAK_497	25	7.86	Nfe2l3	70786	2480204	1.547719479	up
MACS_PEAK_615	21	8.97	Thsd4	70866	630608	2.118721247	down
MACS_PEAK_615	21	8.97	Thsd4	70866	1770086	3.020121813	down
MACS_PEAK_123	56	6.16	Syne2	14944	5090056	3.046834946	down
MACS_PEAK_123	56	6.16	Syne2	14944	5490386	1.561118126	down
MACS_PEAK_141	30	6.09	Actn2	44824	1050678	1.730874062	up
MACS_PEAK_38	21	7	Hhat	76234	6400195	1.552055717	up
MACS_PEAK_462	17	5.92	Auts2	115998	2340097	3.004128218	down
MACS_PEAK_448	18	8.37	Zfp326	49948	6510524	2.000721216	down
MACS_PEAK_449	19	5.49	Zfp326	201	6510524	2.000721216	down
MACS_PEAK_448	18	8.37	Zfp326	49948	4230735	2.138542891	down
MACS_PEAK_449	19	5.49	Zfp326	201	4230735	2.138542891	down
MACS_PEAK_238	57	11.47	D16Ertd472e	95	4780112	1.583073974	down
MACS_PEAK_9	23	7.25	Ercc5	587	4260296	1.576063037	down
MACS_PEAK_205	21	7.25	Oxr1	35	5220685	1.74464941	up
MACS_PEAK_56	26	5.81	Phyhipl	124533	3800035	3.186303616	down
MACS_PEAK_143	23	5.32	Sox4	544575	1850315	1.783293724	down
MACS_PEAK_86	19	5.32	Traf4	1439	1470632	1.509787679	down
MACS_PEAK_105	23	8.46	BC006965	448	780594	1.501077652	up

MACS_PEAK_184	17	6.04	Scara5	37083	160377	2.956916332	up
MACS_PEAK_117	39	15.71	Id2	152197	1030519	4.620919704	down
MACS_PEAK_564	19	8.28	Col4a1	147024	4060102	2.682374954	up
MACS_PEAK_381	21	7.25	Slc44a1	5	7380195	1.802622199	up
MACS_PEAK_381	21	7.25	Slc44a1	5	290360	2.06903863	up
MACS_PEAK_306	19	5.44	Atrnl1	315566	70356	3.97765851	down
MACS_PEAK_323	18	5.92	Tmco5	97281	7330520	1.612348199	up
MACS_PEAK_83	17	6.52	Myh10	53356	7040608	1.665982604	up
MACS_PEAK_109	24	6.37	Tnrc6c	95028	2370484	1.673379421	down
MACS_PEAK_377	18	6.51	Glipr2	7471	3940639	1.803641558	up
MACS_PEAK_75	21	6.58	Ppp2ca	180	4590746	1.536321878	up
MACS_PEAK_136	16	8.46	Dnahc11	154636	4220139	1.538279057	up
MACS_PEAK_573	18	7.32	Gpm6a	160146	7400240	1.517501831	up
MACS_PEAK_150	20	9.06	S1pr3	149321	4920349	1.749848127	up
MACS_PEAK_10	38	8.43	Myo1b	80859	4920215	2.869684696	down
MACS_PEAK_391	18	5.44	Sgip1	32698	3310474	1.610694766	up
MACS_PEAK_164	21	7.26	Mtap1b	274407	5810332	2.097805023	down
MACS_PEAK_101	39	9.23	Helz	373	6550270	2.73856616	down
MACS_PEAK_351	18	7.25	Mbnl1	234918	5090156	1.676026583	down
MACS_PEAK_404	21	9.06	Ptpru	384315	5270161	2.411968231	up
MACS_PEAK_438	22	7.86	Rasl11b	70991	150372	1.523000121	up
MACS_PEAK_438	22	7.86	Rasl11b	70991	290341	2.123251438	up
MACS_PEAK_44	17	6.04	Epb4.1l2	45032	3870452	1.960246444	down
MACS_PEAK_361	21	5.32	Trim45	10480	4640719	1.639558554	up
MACS_PEAK_231	140	4.32	Abi3bp	85059	4490730	2.414456606	up
MACS_PEAK_447	26	7.99	Mapk10	197150	4610411	2.018272877	down
MACS_PEAK_347	31	10.65	Elf2	36691	1780050	1.779895663	down
MACS_PEAK_347	31	10.65	Elf2	36691	130386	1.971977711	down
MACS_PEAK_15	22	5.86	Klf7	893	4850367	2.45822978	down
MACS_PEAK_27	26	8.46	Hmcn1	177150	1450347	2.41880703	up
MACS_PEAK_26	16	7.86	Hmcn1	340383	1450347	2.41880703	up
MACS_PEAK_293	20	7.99	Tshz1	231497	5810184	2.919794083	down
MACS_PEAK_579	30	10.06	Gipc1	152	1090468	1.569516063	up
MACS_PEAK_42	19	7.25	Map3k5	33465	6380068	1.551097274	up
MACS_PEAK_446	18	7.25	Wdfy3	178678	1740220	1.845359206	down
MACS_PEAK_61	19	9.06	Matk	218	1940646	1.540915251	up
MACS_PEAK_556	21	6.65	Zfp668	1063	5860280	1.656523585	down
MACS_PEAK_332	19	7.86	Eya2	2690	2100750	1.58567214	up
MACS_PEAK_316	20	6.16	Gad1	99227	830437	4.026839256	down
MACS_PEAK_624	18	7.25	Osbpl10	140998	1690040	1.558575511	up
MACS_PEAK_605	51	12.1	Fbxl12	475	2690437	1.704402566	up
MACS_PEAK_199	15	7.25	6030458C11Rik	244374	6400136	2.476020098	down
MACS_PEAK_316	20	6.16	Gad1	99227	4920243	3.517460346	down
MACS_PEAK_569	26	9.2	Pdlim3	223673	650452	2.682033539	up
MACS_PEAK_523	61	10.11	Aebp2	348364	610020	1.723865151	up
MACS_PEAK_335	25	9.05	Pfdn4	52417	4590750	4.021124363	down
MACS_PEAK_403	18	5.44	Ptpru	815980	3170735	2.041645288	up
MACS_PEAK_429	18	5.45	Nos3	39066	6290328	1.930089355	up
MACS_PEAK_429	18	5.45	Nos3	39066	7570647	2.211401463	up
MACS_PEAK_563	30	7.74	Tnfsf13b	401151	6840059	1.609321356	up
MACS_PEAK_390	17	6.65	Nfia	331212	130544	1.942586541	up
MACS_PEAK_563	30	7.74	Tnfsf13b	401151	610661	1.764905214	up
MACS_PEAK_619	18	9.06	Tcf12	339337	5080356	4.621706009	down
MACS_PEAK_258	210	5	Notch4	22613	4250059	2.045795679	up
MACS_PEAK_202	18	9.67	Stk3	233926	5270402	2.437416553	up
MACS_PEAK_204	19	7.86	Grhl2	201175	5340731	1.951004982	up
MACS_PEAK_269	17	6.65	Tgif1	212913	7050228	1.705920577	up
MACS_PEAK_269	17	6.65	Tgif1	212913	1090100	2.285291433	up
MACS_PEAK_199	15	7.25	6030458C11Rik	244374	2260148	2.817769766	down
MACS_PEAK_471	21	5.13	Sdk1	1126400	380463	2.000249386	up
MACS_PEAK_316	20	6.16	Gad1	99227	7550053	3.400148869	down
MACS_PEAK_292	21	6.04	Nfatc1	135965	70392	2.159632921	up
MACS_PEAK_292	21	6.04	Nfatc1	135965	7160220	2.385699749	up
MACS_PEAK_290	28	6.21	Acaa2	112135	3310424	2.013747454	up
MACS_PEAK_210	33	5.32	Sox10	28757	4260301	4.689880848	up
MACS_PEAK_602	20	6.51	Birc2	71007	6040647	1.747507811	up
MACS_PEAK_614	18	6.04	Stoml1	5870	5960014	1.722013474	up
MACS_PEAK_358	16	6.78	Prpf3	36043	1440025	1.823522449	up
MACS_PEAK_543	30	10.88	Iqgap1	57613	4830538	2.730445385	down
MACS_PEAK_525	43	18.13	Sox5	709796	4880553	4.62940979	down
MACS_PEAK_526	25	7.25	Sox5	678621	4880553	4.62940979	down
MACS_PEAK_527	23	7.86	Sox5	518337	4880553	4.62940979	down
MACS_PEAK_525	43	18.13	Sox5	709796	5050082	6.362275124	down
MACS_PEAK_526	25	7.25	Sox5	678621	5050082	6.362275124	down
MACS_PEAK_527	23	7.86	Sox5	518337	5050082	6.362275124	down
MACS_PEAK_525	43	18.13	Sox5	709796	6020273	2.341292381	down
MACS_PEAK_526	25	7.25	Sox5	678621	6020273	2.341292381	down
MACS_PEAK_527	23	7.86	Sox5	518337	6020273	2.341292381	down
MACS_PEAK_606	41	6.47	A230050P20Rik	31347	2690091	1.616741896	up
MACS_PEAK_606	41	6.47	A230050P20Rik	31347	6940132	1.528307199	up
MACS_PEAK_589	20	6.65	Cdh5	830811	1230129	2.108512402	up
MACS_PEAK_525	43	18.13	Sox5	709796	6200707	1.902192593	down
MACS_PEAK_526	25	7.25	Sox5	678621	6200707	1.902192593	down
MACS_PEAK_527	23	7.86	Sox5	518337	6200707	1.902192593	down
MACS_PEAK_582	18	5.49	Trmt1	12123	7550240	1.576895714	up
MACS_PEAK_309	23	6.44	Cdc123	277601	7560326	3.020993233	down
MACS_PEAK_404	21	9.06	Matn1	509578	4280487	17.49271202	down
MACS_PEAK_406	29	9.05	Clic4	106100	3400491	1.885798573	down
MACS_PEAK_521	24	9.67	Atf7ip	52786	5690451	1.804197907	down
MACS_PEAK_421	16	7.1	Ajap1	434847	2650731	1.863787413	up
MACS_PEAK_444	18	8.46	Shroom3	81837	2370471	1.598723769	up
MACS_PEAK_523	61	10.11	Aebp2	348364	6350767	1.849905491	up
MACS_PEAK_425	33	7.26	Ski	27051	1940689	1.679907203	down
MACS_PEAK_548	22	7.76	Insc	625532	3520397	6.45871973	up
MACS_PEAK_192	17	7.25	1300010F03Rik	285952	4670397	1.556489348	up
MACS_PEAK_574	22	9.67	Mef2b	19556	6020243	1.606473804	up
MACS_PEAK_625	18	6.65	Rbms3	867910	70497	2.295167208	down

MACS_PEAK_626	17	7.86	Rbms3	781450	70497	2.295167208	down
MACS_PEAK_308	374	2.55	Csf2ra	28660	5550437	1.566088319	up
MACS_PEAK_193	17	7.25	Ndfip2	329701	2940196	3.606239796	down
MACS_PEAK_282	26	7.1	Yipf5	88836	7160402	2.609052896	down
MACS_PEAK_197	16	6.04	Lifr	194784	5360220	1.614066362	up
MACS_PEAK_198	21	9.06	Lifr	152783	5360220	1.614066362	up
MACS_PEAK_197	16	6.04	Lifr	194784	670634	1.600601077	up
MACS_PEAK_198	21	9.06	Lifr	152783	670634	1.600601077	up
MACS_PEAK_283	25	6.3	Ythdc2	731010	7650424	1.631167531	up
MACS_PEAK_312	40	9.25	St6galnac6	14368	6180452	1.534910202	up
MACS_PEAK_473	23	7.73	Fbxl18	77384	7210066	1.584769487	down
MACS_PEAK_474	31	5.02	Fbxl18	74819	7210066	1.584769487	down
MACS_PEAK_475	31	7.78	Fbxl18	71724	7210066	1.584769487	down
MACS_PEAK_274	16	7.25	Srbd1	290838	1500202	2.141215324	down
MACS_PEAK_610	17	6.65	Robo4	17373	2630333	1.980917573	up
MACS_PEAK_571	31	5.8	D030016E14Rik	244986	1030670	1.598960757	up
MACS_PEAK_266	29	8.87	Ptprm	255587	1580484	1.672431231	up
MACS_PEAK_369	17	6.04	Mcoln3	15056	50487	2.695793152	up
MACS_PEAK_226	33	12.09	Cldn1	283981	3450300	2.176027298	up
MACS_PEAK_479	22	8.46	B3galtl	329495	5270315	1.861565471	up
MACS_PEAK_594	32	7.25	Plcg2	162431	1940255	1.661407828	up
MACS_PEAK_600	17	7.99	Pard3	535145	3400343	1.587090254	up
MACS_PEAK_524	19	5.79	St8sia1	89124	2340609	1.859755993	down
MACS_PEAK_251	63	5.34	Mllt4	152524	6940136	3.550232172	down
MACS_PEAK_252	15	7.86	Mllt4	151696	6940136	3.550232172	down
MACS_PEAK_251	63	5.34	Mllt4	152524	1260519	1.815965772	down
MACS_PEAK_252	15	7.86	Mllt4	151696	1260519	1.815965772	down
MACS_PEAK_488	21	7.86	Ptprz1	298115	1690239	1.955455542	down
MACS_PEAK_222	57	5.81	Snn	53570	6110750	1.511974692	up
MACS_PEAK_481	17	7.25	Stard13	204236	4730079	1.853927612	up
MACS_PEAK_429	18	5.45	Nos3	39066	4570066	2.049824476	up
MACS_PEAK_346	23	6.8	Slc7a11	686106	5080369	1.922748923	up
MACS_PEAK_521	24	9.67	Atf7ip	52786	4570709	3.180575371	down
MACS_PEAK_470	18	6.04	Foxk1	654922	1510273	1.6241889	up
MACS_PEAK_500	19	7.1	Jazf1	114226	2070612	1.949540854	up
MACS_PEAK_513	16	6.65	Frmd4b	364260	5050403	2.866351604	down
MACS_PEAK_206	23	7.86	Nsmce2	246811	7400431	1.582140684	down
MACS_PEAK_200	36	8.69	Cdh6	2219237	520544	4.927514076	up
MACS_PEAK_388	27	8.52	BC057079	173710	6840592	1.598837972	up
MACS_PEAK_374	23	9.06	Epha7	3271151	4670148	2.36863327	up
MACS_PEAK_374	23	9.06	Epha7	3271151	7050327	1.909336686	up
MACS_PEAK_446	18	7.25	Arhgap24	232792	6550470	1.760208368	up
MACS_PEAK_383	17	9.06	Svep1	98140	630292	1.949231148	up
MACS_PEAK_280	16	6.58	Zfp397	150529	6560390	1.554591537	up
MACS_PEAK_290	28	6.21	Acaa2	112135	4010398	2.12840867	up
MACS_PEAK_290	28	6.21	Acaa2	112135	5090414	2.446219921	up
MACS_PEAK_460	21	6.1	Auts2	831430	2340097	3.004128218	down
MACS_PEAK_461	21	7.86	Auts2	691283	2340097	3.004128218	down
MACS_PEAK_523	61	10.11	Aebp2	348364	4200086	1.62104094	up
MACS_PEAK_450	24	8.87	Zfp326	384900	6510524	2.000721216	down
MACS_PEAK_450	24	8.87	Zfp326	384900	4230735	2.138542891	down
MACS_PEAK_578	22	8.46	Il15	634971	6940402	1.798205018	up
MACS_PEAK_578	22	8.46	Il15	634971	4480735	1.629272938	up
MACS_PEAK_578	22	8.46	Il15	634971	2690768	1.812394142	up
MACS_PEAK_266	29	8.87	Ptprm	255587	2600367	1.611815929	up
MACS_PEAK_202	18	9.67	Stk3	233926	6330408	2.009322882	up
MACS_PEAK_346	23	6.8	Slc7a11	686106	520470	1.724648714	up
MACS_PEAK_569	26	9.2	Pdlim3	223673	2000482	1.562441349	up
MACS_PEAK_618	18	4.83	Rasl12	7942	1820037	1.698372841	up
MACS_PEAK_589	20	6.65	Cdh5	830811	1030273	1.843039036	up
MACS_PEAK_622	20	7.86	Epm2aip1	39821	1510176	5.14840889	down
MACS_PEAK_303	17	6.65	Sorcs1	1806464	4060273	1.559650183	up
MACS_PEAK_350	27	4.88	Mbnl1	251385	5090156	1.676026583	down
MACS_PEAK_375	19	5.58	Bach2	236734	3460142	3.964662313	down
MACS_PEAK_403	18	5.44	Ptpru	815980	5270161	2.411968231	up
MACS_PEAK_495	17	7.86	1810058I24Rik	31248	1440431	3.318252325	down
MACS_PEAK_341	34	4.88	Dnajc5b	197654	2690301	1.910481453	up
MACS_PEAK_459	22	8.09	Rimbp2	154734	6270019	1.59570837	up
MACS_PEAK_558	29	13.9	Mki67	626695	4490110	1.912924767	down
MACS_PEAK_558	29	13.9	Mki67	626695	5220553	2.990350723	down
MACS_PEAK_514	20	5.32	Shq1	275223	1410014	1.644196033	up
MACS_PEAK_344	26	6.65	Skil	45044	3800274	1.511991978	down
MACS_PEAK_496	16	5.92	Rarres2	17488	6060367	2.030186653	up
MACS_PEAK_561	40	5.32	Tpcn2	77364	2190364	2.939031839	up
MACS_PEAK_381	21	7.25	AI427809	170186	2320564	1.684773922	up
MACS_PEAK_314	36	8.6	Ggta1	52859	4250066	1.651818871	up
MACS_PEAK_619	18	9.06	Tcf12	339337	3830647	1.750166893	down
MACS_PEAK_619	18	9.06	Tcf12	339337	2030274	2.977221966	down
MACS_PEAK_326	19	6.65	Sirpa	65087	3180368	1.617762208	up
MACS_PEAK_443	32	5.7	Uso1	54154	4290746	1.944169879	up

Table S5 Sox6 (8 pages)

CHIPSeq_MACS_Peak_id	CHIPSeq_MACS_PeakTags	CHIPSeq_MACS_PeakFoldEnrichment	GenesSymbol
MACS_PEAK_188	28	7.97	Nfib
MACS_PEAK_146	18	5.62	Rapgef4
MACS_PEAK_109	21	6.09	Ripk4
MACS_PEAK_95	38	8.44	Myo10
MACS_PEAK_96	41	10.78	Myo10
MACS_PEAK_53	18	6.09	Ccdc46
MACS_PEAK_57	27	8.91	Rhob
MACS_PEAK_288	73	17.92	Fbxl12
MACS_PEAK_40	99	27.19	Odz2
MACS_PEAK_120	29	6.94	Reep5
MACS_PEAK_162	46	3.64	Rap2b
MACS_PEAK_252	37	11.72	Dbx1
MACS_PEAK_272	40	12.66	Pdlim3
MACS_PEAK_136	27	8.91	Sorbs1
MACS_PEAK_282	36	13.12	Zfhx3
MACS_PEAK_282	36	13.12	Zfhx3
MACS_PEAK_4	35	11.48	Fhl2
MACS_PEAK_241	159	24.57	Aebp2
MACS_PEAK_241	159	24.57	Pde3a
MACS_PEAK_291	30	5.58	Tle3
MACS_PEAK_192	25	5.62	Nfia
MACS_PEAK_193	19	6.36	Nfia
MACS_PEAK_194	31	7.03	Nfia
MACS_PEAK_36	34	7.1	Krr1
MACS_PEAK_102	20	5.16	Tbx1
MACS_PEAK_114	179	30.85	Notch4
MACS_PEAK_2	40	5.73	Cpa6
MACS_PEAK_188	28	7.97	Nfib
MACS_PEAK_184	19	6.56	Tmem38b
MACS_PEAK_55	30	6.94	2610035D17Rik
MACS_PEAK_130	20	6.09	Trpm3
MACS_PEAK_131	22	5.16	Trpm3
MACS_PEAK_299	44	12.5	Pin4
MACS_PEAK_250	23	6.07	Tshz3
MACS_PEAK_57	27	8.91	Hs1bp3
MACS_PEAK_78	29	6.09	Edil3
MACS_PEAK_294	17	5.16	Fbxo9
MACS_PEAK_41	20	4.77	Adamts2
MACS_PEAK_41	20	4.77	Adamts2
MACS_PEAK_188	28	7.97	Nfib
MACS_PEAK_258	19	5.16	Parva
MACS_PEAK_3	23	7.03	Col9a1
MACS_PEAK_244	31	8.44	Sox5
MACS_PEAK_245	37	5.16	Sox5
MACS_PEAK_246	118	35.62	Sox5
MACS_PEAK_247	43	14.53	Sox5
MACS_PEAK_242	54	14.45	Sox5

MACS_PEAK_243	22	5.77	Sox5
MACS_PEAK_244	31	8.44	Sox5
MACS_PEAK_245	37	5.16	Sox5
MACS_PEAK_246	118	35.62	Sox5
MACS_PEAK_247	43	14.53	Sox5
MACS_PEAK_242	54	14.45	Sox5
MACS_PEAK_243	22	5.77	Sox5
MACS_PEAK_244	31	8.44	Sox5
MACS_PEAK_245	37	5.16	Sox5
MACS_PEAK_246	118	35.62	Sox5
MACS_PEAK_247	43	14.53	Sox5
MACS_PEAK_242	54	14.45	Sox5
MACS_PEAK_243	22	5.77	Sox5
MACS_PEAK_290	32	7.46	Sorl1
MACS_PEAK_299	44	12.5	Rgag4
MACS_PEAK_204	24	8.44	Phox2b
MACS_PEAK_155	44	13.89	Tshz2
MACS_PEAK_114	179	30.85	Gpsm3
MACS_PEAK_191	30	8.91	Cyp2j9
MACS_PEAK_244	31	8.44	Sox5
MACS_PEAK_245	37	5.16	Sox5
MACS_PEAK_246	118	35.62	Sox5
MACS_PEAK_247	43	14.53	Sox5
MACS_PEAK_242	54	14.45	Sox5
MACS_PEAK_243	22	5.77	Sox5
MACS_PEAK_188	28	7.97	Nfib
MACS_PEAK_152	26	7.73	Slc24a3
MACS_PEAK_147	23	7.03	Mtx2
MACS_PEAK_31	26	6.21	Ank3
MACS_PEAK_79	17	6.09	F2rl1
MACS_PEAK_115	24	6.56	Rab31
MACS_PEAK_225	53	18.54	Chchd3
MACS_PEAK_240	34	9.11	Atf7ip
MACS_PEAK_199	26	7.1	Ajap1
MACS_PEAK_193	19	6.36	E130114P18Rik
MACS_PEAK_241	159	24.57	Aebp2
MACS_PEAK_259	34	7.46	Insc
MACS_PEAK_21	27	8.73	Tgfb2
MACS_PEAK_37	31	9.07	Frs2
MACS_PEAK_109	21	6.09	Tmprss2
MACS_PEAK_15	25	8.44	Rgs16
MACS_PEAK_127	27	8.44	Tcf4
MACS_PEAK_97	21	4.69	Col22a1
MACS_PEAK_117	34	5.21	Ypel5
MACS_PEAK_161	36	8.98	Foxo1
MACS_PEAK_237	39	6.91	Ing4
MACS_PEAK_259	34	7.46	Sox6
MACS_PEAK_260	21	7.97	Sox6

MACS_PEAK_261	25	7.97	Sox6
MACS_PEAK_31	26	6.21	Ccdc6
MACS_PEAK_9	20	5.62	Mtap2
MACS_PEAK_231	28	9.37	Magi1
MACS_PEAK_232	56	8.85	Magi1
MACS_PEAK_283	21	6.56	Gm1943
MACS_PEAK_93	44	12.89	Lifr
MACS_PEAK_93	44	12.89	Lifr
MACS_PEAK_123	21	6.74	Csnk1g3
MACS_PEAK_144	53	5.3	St6galnac6
MACS_PEAK_284	31	4.69	Maf
MACS_PEAK_23	38	11.25	Cited2
MACS_PEAK_23	38	11.25	Cited2
MACS_PEAK_59	26	9.37	Sox11
MACS_PEAK_178	20	7.03	Lrrc7
MACS_PEAK_221	47	6.69	Fbxl18
MACS_PEAK_106	52	9.21	Grik1
MACS_PEAK_174	45	8.62	Col25a1
MACS_PEAK_174	45	8.62	Col25a1
MACS_PEAK_116	23	7.03	Ptprm
MACS_PEAK_188	28	7.97	Nfib
MACS_PEAK_3	23	7.03	Col9a1
MACS_PEAK_104	34	8.44	Phldb2
MACS_PEAK_286	23	6.56	Pard3
MACS_PEAK_171	24	6.09	Prmt6
MACS_PEAK_237	39	6.91	Ing4
MACS_PEAK_264	23	7.03	Mgmt
MACS_PEAK_89	28	4.97	Gm1587
MACS_PEAK_223	27	7.03	Stard13
MACS_PEAK_77	30	7.97	Mef2c
MACS_PEAK_160	22	6.09	Slc7a11
MACS_PEAK_9	20	5.62	Mtap2
MACS_PEAK_276	59	12.69	Ednra
MACS_PEAK_200	58	13.71	Arhgef16
MACS_PEAK_58	24	8.44	Laptm4a
MACS_PEAK_240	34	9.11	Atf7ip
MACS_PEAK_9	20	5.62	Mtap2
MACS_PEAK_207	25	5.96	Cnot6l
MACS_PEAK_176	23	6.56	Unc5c
MACS_PEAK_49	29	7.81	Rad51c
MACS_PEAK_77	30	7.97	Mef2c
MACS_PEAK_171	24	6.09	Ntng1
MACS_PEAK_143	22	5.62	Thnsl1
MACS_PEAK_143	22	5.62	Thnsl1
MACS_PEAK_190	25	5.47	BC057079
MACS_PEAK_109	21	6.09	Ripk4
MACS_PEAK_186	20	5.16	Snx30
MACS_PEAK_170	41	10.87	Sort1

MACS_PEAK_106	52	9.21	Bach1
MACS_PEAK_292	21	6.51	Nedd4
MACS_PEAK_172	38	9.37	Col11a1
MACS_PEAK_172	38	9.37	Col11a1
MACS_PEAK_130	20	6.09	Tmem2
MACS_PEAK_220	23	6.56	Auts2
MACS_PEAK_241	159	24.57	Aebp2
MACS_PEAK_116	23	7.03	Ptprm
MACS_PEAK_54	26	7.03	BC006965
MACS_PEAK_160	22	6.09	Slc7a11
MACS_PEAK_272	40	12.66	Pdlim3
MACS_PEAK_289	22	6.56	Ets1
MACS_PEAK_210	32	8.97	Mtf2
MACS_PEAK_163	22	6.56	Mme
MACS_PEAK_168	19	6.39	Igsf3
MACS_PEAK_67	44	5.02	Dnahc11
MACS_PEAK_66	22	6.56	Dnahc11
MACS_PEAK_175	21	6.56	Nfkb1
MACS_PEAK_132	22	8.44	Papss2
MACS_PEAK_133	30	7.97	Papss2
MACS_PEAK_132	22	8.44	Papss2
MACS_PEAK_133	30	7.97	Papss2
MACS_PEAK_224	31	12.19	2610001J05Rik
MACS_PEAK_11	17	7.03	Epha4
MACS_PEAK_106	52	9.21	Grik1
MACS_PEAK_181	29	8.44	Bach2
MACS_PEAK_170	41	10.87	Sort1
MACS_PEAK_205	20	5.62	Rasl11b
MACS_PEAK_206	31	6.64	Rasl11b
MACS_PEAK_205	20	5.62	Rasl11b
MACS_PEAK_206	31	6.64	Rasl11b
MACS_PEAK_188	28	7.97	Zdhhc21
MACS_PEAK_86	20	5.62	Tnfrsf19
MACS_PEAK_264	23	7.03	Mki67
MACS_PEAK_264	23	7.03	Mki67
MACS_PEAK_14	18	6.56	Ube2t
MACS_PEAK_233	19	5.62	Shq1
MACS_PEAK_156	45	11.65	Slmo2
MACS_PEAK_277	30	7.81	Gipc1
MACS_PEAK_184	19	6.56	Zfp462
MACS_PEAK_266	42	7.03	Tpcn2
MACS_PEAK_145	37	6.31	Ggta1
MACS_PEAK_216	29	4.23	Mvk
MACS_PEAK_99	25	7.97	Krt7
MACS_PEAK_172	38	9.37	Rnpc3

AbsoluteDistanceFromPeakToGene	ProbeID	ExpressionFoldChangeAbsolute	ExpressionRegulationDirection
200638	4060075	2.002110958	down
56846	2450601	1.735148907	up
80229	1570598	1.717285275	up
20188	1470035	1.700320125	up
31284	1470035	1.700320125	up
75647	2260301	1.526793838	up
113454	1710538	2.576362133	down
465	2690437	1.704402566	up
734148	7050408	2.188945532	down
9844	730541	1.620755076	up
836767	2710189	1.678804159	up
109201	1710487	1.643950105	down
223656	650452	2.682033539	up
7872	4250373	3.83799386	down
894067	3870068	2.569363832	down
894067	6580376	1.584697604	down
194368	2350019	1.584973693	up
348081	610020	1.723865151	up
276890	4070168	1.607645631	up
246190	2710338	1.613103151	down
115765	130544	1.942586541	up
54856	130544	1.942586541	up
331218	130544	1.942586541	up
47067	730594	1.633091927	up
73346	2900239	1.628616214	up
22617	4250059	2.045795679	up
274009	510601	1.919758916	up
200638	2350162	3.720114231	down
206387	4540626	1.543681145	up
13206	1090044	1.550805688	down
110562	2260600	1.723128915	up
212703	2260600	1.723128915	up
181	5360615	3.598824739	down
682715	380438	1.775392175	up
73098	520711	1.551209569	up
644986	70050	1.579653502	up
31532	2140411	1.588924289	up
60853	7570563	2.20483923	up
60853	6520273	2.847491264	up
200638	540474	1.540476203	down
72679	290037	2.095227003	up
18627	7040373	1.630497336	down
170157	4880553	4.62940979	down
152616	4880553	4.62940979	down
578	4880553	4.62940979	down
55276	4880553	4.62940979	down
709817	4880553	4.62940979	down

678583	4880553	4.62940979	down
170157	5050082	6.362275124	down
152616	5050082	6.362275124	down
578	5050082	6.362275124	down
55276	5050082	6.362275124	down
709817	5050082	6.362275124	down
678583	5050082	6.362275124	down
170157	6020273	2.341292381	down
152616	6020273	2.341292381	down
578	6020273	2.341292381	down
55276	6020273	2.341292381	down
709817	6020273	2.341292381	down
678583	6020273	2.341292381	down
388178	6960577	1.710558295	down
47977	360608	1.550311446	up
130741	6220736	1.636369348	up
296502	6270008	1.758626103	down
2894	3850221	1.547520876	up
38997	4260746	1.525645852	up
170157	6200707	1.902192593	down
152616	6200707	1.902192593	down
578	6200707	1.902192593	down
55276	6200707	1.902192593	down
709817	6200707	1.902192593	down
678583	6200707	1.902192593	down
200638	5290202	2.964277744	down
50443	4280131	1.602561235	up
62853	6400204	1.664726734	up
169782	6020176	2.969679832	down
12614	1410348	1.919010401	up
70221	290315	1.548935175	up
263	7550743	1.594190598	up
52744	5690451	1.804197907	down
214466	2650731	1.863787413	up
52208	4280184	3.115866661	down
348081	6350767	1.849905491	up
625574	3520397	6.45871973	up
64819	770754	4.134972572	down
14406	7560195	1.629254341	up
72331	4070279	2.118969917	up
80426	3890528	1.761289358	up
367856	7550360	1.630245209	down
29482	5090348	1.713129163	up
30324	6760008	1.505259752	down
71747	6580682	2.203903913	up
41	6550408	1.596772075	up
623746	4070386	2.446631193	down
414474	4070386	2.446631193	down

379334	4070386	2.446631193	down
1807	6550022	3.392610073	down
213551	3180593	2.897240162	down
592	2760328	1.938788414	up
71	2760328	1.938788414	up
154914	6760754	1.800988078	down
194812	5360220	1.614066362	up
194812	670634	1.600601077	up
512286	4880600	1.628932357	down
14410	6180452	1.534910202	up
1181	4250450	1.702198863	up
648262	6020379	1.539243817	down
648262	5860538	1.61139214	down
210302	990681	2.488309383	down
209117	7040129	1.692220926	up
77487	7210066	1.584769487	down
453083	1190474	1.662040234	up
19767	6020286	1.512342572	up
19767	4760746	1.686721325	up
694936	1580484	1.672431231	up
200638	4180288	2.907538176	down
18627	3990167	11.82479382	down
92335	4280576	1.582591534	up
201912	3400343	1.587090254	up
114668	2650564	1.552326798	up
41	4210168	1.761608839	up
551466	3890072	1.876641154	up
179396	1050338	1.520992517	up
204269	4730079	1.853927612	up
42186	5820369	1.849823236	down
193593	5080369	1.922748923	up
213551	6100195	2.891719818	down
12939	4040189	2.376912355	up
13561	4850719	1.68380928	up
319	5560411	2.131327391	up
52744	4570709	3.180575371	down
213551	6220632	1.541220665	down
48114	2900725	1.637559295	down
155903	2510291	1.731092215	down
39104	3360253	1.950005174	down
42186	2570427	1.64749217	down
7142	1990519	2.279101133	up
320003	5490575	1.586515665	up
320003	5360242	1.570795536	up
32333	6840592	1.598837972	up
80229	3850086	2.464329481	up
21624	5270671	1.611051083	down
1504	5360706	1.571092129	up

138221	1990487	3.790873051	down
29752	2760386	1.531288147	up
331265	1170192	2.961684942	down
331265	6520184	2.004609585	down
559304	2570142	3.613222599	down
792675	2340097	3.004128218	down
348081	4200086	1.62104094	up
694936	2600367	1.611815929	up
336817	780594	1.501077652	up
193593	520470	1.724648714	up
223656	2000482	1.562441349	up
130243	6020398	3.387423277	up
138	630615	2.279771328	down
237870	7100259	2.044068336	up
33860	2100201	2.070895195	down
154795	4220139	1.538279057	up
308608	4220139	1.538279057	up
26533	4180669	1.526234984	up
6343	2710739	1.707736969	down
10832	2710739	1.707736969	down
6343	4260148	2.034782171	down
10832	4260148	2.034782171	down
760765	3890240	1.686969757	up
590050	770148	1.533346653	up
453083	3890370	1.907122612	up
13796	3460142	3.964662313	down
1504	6370600	1.556075573	up
58943	150372	1.523000121	up
71025	150372	1.523000121	up
58943	290341	2.123251438	up
71025	290341	2.123251438	up
554991	2650064	2.122733355	down
253421	2060546	1.982052088	up
626766	4490110	1.912924767	down
626766	5220553	2.990350723	down
36258	3190403	2.584891558	down
162615	1410014	1.644196033	up
16676	6660497	1.659236312	down
154	1090468	1.569516063	up
915513	1570356	2.887309313	down
77474	2190364	2.939031839	up
52800	4250066	1.651818871	up
106156	1230561	1.720483065	up
15748	7330477	1.59222734	up
69126	2750315	1.966447353	down

Table S5 Sox9 (17 pages)

CHIPSeq_MACS_Peak_id	CHIPSeq_MACS_PeakTags	CHIPSeq_MACS_PeakFoldEnrichment	GenesSymbol	AbsoluteDistanceFromPeakToGene	ProbeID	ExpressionFoldChangeAbsolute	ExpressionRegulationDirection
MACS_PEAK_12	32	14.54	1110058L19Rik	28547	6770020	1.525534511	down
MACS_PEAK_13	54	18.78	Col9a1	18482	7100446	19.59921646	down
MACS_PEAK_14	32	11.51	Col9a1	44317	7100446	19.59921646	down
MACS_PEAK_15	28	14.54	Col9a1	53031	7100446	19.59921646	down
MACS_PEAK_16	18	7.27	Dst	7379	430026	1.81259644	up
MACS_PEAK_26	25	7.27	Map4k4	47007	100610154	1.717309713	up
MACS_PEAK_43	23	6.65	Gls	21296	101580047	3.115065575	down
MACS_PEAK_66	28	7.88	Fzd5	54532	620296	1.685383677	down
MACS_PEAK_103	43	19.99	Ndufa10	94318	105390369	1.53215611	up
MACS_PEAK_135	28	10.91	Prelp	135	5900390	11.84494877	down
MACS_PEAK_150	36	13.85	Glt25d2	7909	540465	1.897881746	down
MACS_PEAK_151	28	12.12	Glt25d2	55567	540465	1.897881746	down
MACS_PEAK_155	23	10.91	Glul	62421	6040253	2.251183271	up
MACS_PEAK_156	42	16.96	Glul	78531	6040253	2.251183271	up
MACS_PEAK_185	20	6.06	Sdccag8	578	1780687	1.983003497	up
MACS_PEAK_206	38	12.12	Tgfb2	64649	4920292	2.04001236	down
MACS_PEAK_213	59	16.72	Sertad4	28819	130593	1.795625091	down
MACS_PEAK_234	30	14.54	Sgk1	42661	1400131	1.599029541	down
MACS_PEAK_236	56	29.69	Moxd1	157576	2450301	3.239943504	down
MACS_PEAK_237	33	13.93	Ctgf	136662	4540577	4.026652813	down
MACS_PEAK_253	33	16.96	Mical1	834	6550048	2.097376823	up
MACS_PEAK_253	33	16.96	Mical1	834	60603	1.592559576	up
MACS_PEAK_258	29	11.51	Snx3	22027	2940484	1.603039861	down
MACS_PEAK_261	32	6.66	Sobp	120498	2320136	1.753223777	down
MACS_PEAK_272	33	16.36	Hace1	314904	4210348	1.551439762	down
MACS_PEAK_273	184	92.09	Hace1	316293	4210348	1.551439762	down
MACS_PEAK_274	34	11.62	Hace1	1524947	4210348	1.551439762	down
MACS_PEAK_276	19	6.66	Asic3	392600	6550132	1.935636044	down
MACS_PEAK_286	47	14.22	Chst3	23996	2370131	1.71955502	down
MACS_PEAK_287	29	9.05	Chst3	19565	2370131	1.71955502	down
MACS_PEAK_288	58	19.39	Chst3	16257	2370131	1.71955502	down
MACS_PEAK_308	19	7.27	Slc39a3	3564	3990397	1.562371969	up
MACS_PEAK_310	25	10.91	Chst11	103198	6760546	1.705717921	down
MACS_PEAK_311	98	42.79	Chst11	145338	6760546	1.705717921	down
MACS_PEAK_312	22	10.3	Chst11	149578	6760546	1.705717921	down
MACS_PEAK_313	27	8.31	Chst11	154951	6760546	1.705717921	down
MACS_PEAK_314	71	28.47	Chst11	160576	6760546	1.705717921	down
MACS_PEAK_321	17	7.88	Nt5dc3	29175	1090195	2.013053179	down
MACS_PEAK_330	28	5.08	Nudt4	165858	6760689	2.079841614	down
MACS_PEAK_332	72	23.63	Btg1	218855	6200133	16.80504227	down
MACS_PEAK_333	18	7.88	Btg1	209436	6200133	16.80504227	down
MACS_PEAK_334	19	6.1	Dcn	165735	5900711	6.7773633	down
MACS_PEAK_334	19	6.1	Dcn	165735	6550092	2.875388622	down
MACS_PEAK_346	204	100.57	Krr1	47067	6130504	3.11702013	down
MACS_PEAK_352	34	13.3	Cnot2	47080	5550113	4.808637142	down
MACS_PEAK_364	19	7.88	Hmga2	109181	5130279	2.021983624	up
MACS_PEAK_378	29	10.16	Lrp1	21445	6270386	1.784999251	up
MACS_PEAK_379	24	9.74	Lrp1	12050	6270386	1.784999251	up
MACS_PEAK_386	21	8.48	Tns3	239877	6380242	1.662731886	down
MACS_PEAK_387	20	5.54	Tns3	11647	6380242	1.662731886	down
MACS_PEAK_388	44	15.01	Tns3	97222	6380242	1.662731886	down
MACS_PEAK_400	22	7.27	Tmem17	30490	5900079	1.602697849	down
MACS_PEAK_401	20	6.66	Tmem17	75056	5900079	1.602697849	down
MACS_PEAK_402	25	9.14	Bcl11a	227426	6860369	1.595377803	up
MACS_PEAK_414	21	10.91	Odz2	239963	430538	1.597476363	up
MACS_PEAK_415	21	8.48	Odz2	854680	430538	1.597476363	up
MACS_PEAK_420	55	18.78	Clint1	595760	7050242	3.635176659	down
MACS_PEAK_421	28	6.97	Clint1	462406	7050242	3.635176659	down
MACS_PEAK_422	46	13.93	Clint1	213612	7050242	3.635176659	down
MACS_PEAK_423	21	6.06	Clint1	181234	7050242	3.635176659	down
MACS_PEAK_424	100	49.07	Clint1	29981	7050242	3.635176659	down
MACS_PEAK_425	25	9.09	Gfpt2	29344	2370129	2.037197351	down
MACS_PEAK_426	17	5.45	Gfpt2	881	2370129	2.037197351	down
MACS_PEAK_427	19	6.1	Mapk9	1521	4070397	4.267343521	down
MACS_PEAK_429	22	5.59	Col23a1	116729	106620088	1.654782653	up
MACS_PEAK_447	40	16.36	Pmp22	332444	6550072	12.38402176	down
MACS_PEAK_447	40	16.36	Pmp22	332444	4010239	2.244052867	down
MACS_PEAK_480	27	10.3	Cltc	141	3130168	3.606269836	down
MACS_PEAK_494	41	7.86	Nog	56285	2320609	4.01401329	down
MACS_PEAK_507	17	7.88	Stard3	4204	5720341	1.97995615	up
MACS_PEAK_509	130	50.65	Tns4	23996	5890253	1.763277054	up
MACS_PEAK_525	42	17.57	Kcnj2	43067	630019	2.289093971	down
MACS_PEAK_526	20	10.91	Kcnj2	257162	630019	2.289093971	down
MACS_PEAK_527	24	12.12	Kcnj2	620315	630019	2.289093971	down
MACS_PEAK_528	28	5.54	Kcnj2	735806	630019	2.289093971	down
MACS_PEAK_529	27	11.64	Kcnj2	740766	630019	2.289093971	down
MACS_PEAK_530	27	7.88	Kcnj2	762401	630019	2.289093971	down
MACS_PEAK_549	40	17.18	Sec14l1	8053	3610537	1.908110738	up
MACS_PEAK_550	27	4.57	Sec14l1	6327	3610537	1.908110738	up
MACS_PEAK_560	25	9.69	Bahcc1	2232	5900435	1.527143121	down
MACS_PEAK_561	23	8.48	Bahcc1	10763	5900435	1.527143121	down
MACS_PEAK_589	26	8.48	Sox11	154973	106400717	3.148519754	up
MACS_PEAK_589	26	8.48	Sox11	154973	104010731	1.644564867	up
MACS_PEAK_590	26	7.4	Sox11	31705	106400717	3.148519754	up
MACS_PEAK_590	26	7.4	Sox11	31705	104010731	1.644564867	up
MACS_PEAK_591	38	16.96	Sox11	233088	106400717	3.148519754	up
MACS_PEAK_591	38	16.96	Sox11	233088	104010731	1.644564867	up
MACS_PEAK_593	31	16.36	Tssc1	53576	6590112	1.825648665	down
MACS_PEAK_593	31	16.36	Tssc1	53576	2940504	2.46271801	down
MACS_PEAK_593	31	16.36	Tssc1	53576	130324	4.981688023	down
MACS_PEAK_611	39	17.57	Scin	21076	5420180	11.84676552	down
MACS_PEAK_633	24	7.27	Dact1	54999	1500050	2.195098639	down
MACS_PEAK_633	24	7.27	Dact1	54999	100630088	2.167698622	down
MACS_PEAK_634	18	6.06	Dact1	29891	1500050	2.195098639	down
MACS_PEAK_634	18	6.06	Dact1	29891	100630088	2.167698622	down
MACS_PEAK_635	16	7.88	Dact1	22980	1500050	2.195098639	down
MACS_PEAK_635	16	7.88	Dact1	22980	100630088	2.167698622	down
MACS_PEAK_636	22	5.94	Dact1	88891	1500050	2.195098639	down
MACS_PEAK_636	22	5.94	Dact1	88891	100630088	2.167698622	down
MACS_PEAK_637	92	27.26	Dact1	260728	1500050	2.195098639	down
MACS_PEAK_637	92	27.26	Dact1	260728	100630088	2.167698622	down
MACS_PEAK_649	25	6.66	Gphn	137049	3840121	1.870468378	up
MACS_PEAK_650	283	8.65	Gphn	124349	3840121	1.870468378	up
MACS_PEAK_653	17	7.27	Zfp36l1	403664	2510138	2.099901676	down
MACS_PEAK_654	30	6.97	Zfp36l1	393379	2510138	2.099901676	down
MACS_PEAK_655	25	10.58	Zfp36l1	380065	2510138	2.099901676	down
MACS_PEAK_656	21	9.94	Zfp36l1	268592	2510138	2.099901676	down
MACS_PEAK_657	26	7.23	Zfp36l1	176208	2510138	2.099901676	down
MACS_PEAK_658	35	15.8	Zfp36l1	147353	2510138	2.099901676	down
MACS_PEAK_659	20	7.27	Zfp36l1	142615	2510138	2.099901676	down
MACS_PEAK_660	49	21.2	Zfp36l1	123307	2510138	2.099901676	down
MACS_PEAK_668	20	7.27	Ylpm1	77	1990148	1.884726048	up
MACS_PEAK_682	104	47.26	Ttc8	121168	2760731	2.469508648	down
MACS_PEAK_682	104	47.26	Ttc8	121168	2370528	2.16131115	down
MACS_PEAK_715	19	8.48	Zmynd11	46828	5360040	2.895047188	down
MACS_PEAK_716	233	8.38	Zmynd11	69027	5360040	2.895047188	down
MACS_PEAK_749	30	12.12	Foxf2	85166	1240091	2.093223333	up
MACS_PEAK_767	37	12.72	Wrnip1	4567	5890725	4.550052166	down
MACS_PEAK_811	49	22.11	Tgfbi	27320	2060446	2.340261459	down
MACS_PEAK_812	21	5.59	Tgfbi	2619	2060446	2.340261459	down
MACS_PEAK_817	106	49.07	Fbp2	7537	1580193	2.343615055	down

MACS_PEAK_828	38	9.65	0610007P08Rik	123110	105720600	1.747234583	down
MACS_PEAK_828	38	9.65	0610007P08Rik	123110	106840121	1.564627409	down
MACS_PEAK_834	19	6.06	Zfp825	28807	100620544	1.752790809	down
MACS_PEAK_836	31	10.3	Rhobtb3	335	4010687	1.504313469	down
MACS_PEAK_850	22	10.91	Edil3	154815	101940605	1.737264156	down
MACS_PEAK_851	23	9.69	Hapln1	204198	580398	6.735909462	down
MACS_PEAK_852	20	7.88	Hapln1	145555	580398	6.735909462	down
MACS_PEAK_853	27	9.69	Hapln1	19234	580398	6.735909462	down
MACS_PEAK_854	28	12.72	Vcan	73153	5910053	5.157835484	down
MACS_PEAK_855	56	15.42	Vcan	36873	5910053	5.157835484	down
MACS_PEAK_867	21	9.09	Jmy	11089	3360242	1.592119217	down
MACS_PEAK_881	20	6.06	Mccc2	13597	6110168	2.450114727	down
MACS_PEAK_883	59	20.03	Slc30a5	155924	2970403	3.024980068	down
MACS_PEAK_883	59	20.03	Slc30a5	155924	2630288	2.769304037	down
MACS_PEAK_884	29	13.93	Slc30a5	330667	2970403	3.024980068	down
MACS_PEAK_884	29	13.93	Slc30a5	330667	2630288	2.769304037	down
MACS_PEAK_885	21	9.69	Slc30a5	382755	2970403	3.024980068	down
MACS_PEAK_885	21	9.69	Slc30a5	382755	2630288	2.769304037	down
MACS_PEAK_886	30	7.88	Pik3r1	264446	4730671	2.600860834	down
MACS_PEAK_887	23	10.3	Pik3r1	197724	4730671	2.600860834	down
MACS_PEAK_888	22	12.72	Pik3r1	364085	4730671	2.600860834	down
MACS_PEAK_913	40	4.79	Flnb	8412	103870397	2.532634735	down
MACS_PEAK_914	36	10.91	Flnb	71572	103870397	2.532634735	down
MACS_PEAK_915	93	47.26	Flnb	78968	103870397	2.532634735	down
MACS_PEAK_922	26	7.27	Rarb	22707	430139	2.411809683	down
MACS_PEAK_922	26	7.27	Rarb	22707	1410138	2.624078751	down
MACS_PEAK_923	28	12.12	Rarb	247517	430139	2.411809683	down
MACS_PEAK_923	28	12.12	Rarb	247517	1410138	2.624078751	down
MACS_PEAK_924	19	9.09	Rarb	306265	430139	2.411809683	down
MACS_PEAK_924	19	9.09	Rarb	306265	1410138	2.624078751	down
MACS_PEAK_925	38	13.33	Ube2e1	176349	1090440	3.91310215	down
MACS_PEAK_928	33	15.15	Myst4	37192	1400563	1.949404359	up
MACS_PEAK_928	33	15.15	Myst4	37192	2570687	1.982156515	up
MACS_PEAK_929	44	15.24	Myst4	23670	1400563	1.949404359	up
MACS_PEAK_929	44	15.24	Myst4	23670	2570687	1.982156515	up
MACS_PEAK_934	46	9.75	Rps24	351827	102690538	1.870081425	down
MACS_PEAK_935	29	8.31	Rps24	410376	102690538	1.870081425	down
MACS_PEAK_936	65	18.72	Rps24	436371	102690538	1.870081425	down
MACS_PEAK_952	17	7.62	Bmpr1a	20255	2190193	4.158643246	down
MACS_PEAK_970	20	7.97	Cmtm5	386	6130390	4.380111694	down
MACS_PEAK_974	20	8.48	Gjb6	25048	5390441	1.630724072	down
MACS_PEAK_981	32	6.97	Dleu7	258087	2320402	1.752730727	down
MACS_PEAK_982	46	19.39	Dleu7	99972	2320402	1.752730727	down
MACS_PEAK_987	33	13.93	Stmn4	34113	2900091	1.638828397	down
MACS_PEAK_988	33	16.36	Ppp2r2a	63753	2900014	6.375761509	down
MACS_PEAK_1002	21	7.27	Tsc22d1	888	6040181	16.97862816	down
MACS_PEAK_1002	21	7.27	Tsc22d1	888	1340739	2.117260933	down
MACS_PEAK_1013	48	15.75	Lect1	1606	2640528	1.574525833	down
MACS_PEAK_1014	27	8.48	Lect1	16012	2640528	1.574525833	down
MACS_PEAK_1037	41	19.99	Dct	557565	1090347	1.577306986	down
MACS_PEAK_1037	41	19.99	Dct	557565	3840494	1.660903931	down
MACS_PEAK_1038	63	30.9	Dct	28368	1090347	1.577306986	down
MACS_PEAK_1038	63	30.9	Dct	28368	3840494	1.660903931	down
MACS_PEAK_1041	77	40.59	Mbnl2	116497	3450707	1.756134391	down
MACS_PEAK_1042	45	21.9	Mbnl2	1075	3450707	1.756134391	down
MACS_PEAK_1043	52	13.33	Mbnl2	18465	3450707	1.756134391	down
MACS_PEAK_1083	28	10.91	Laptm4b	25342	4150398	2.49363327	up
MACS_PEAK_1084	21	9.69	Pabpc1	12059	6020632	1.694299459	down
MACS_PEAK_1084	21	9.69	Pabpc1	12059	2650180	2.18592906	down
MACS_PEAK_1084	21	9.69	Pabpc1	12059	2690253	3.044068575	down
MACS_PEAK_1102	48	22.42	Col14a1	62479	1570398	2.242420435	down
MACS_PEAK_1103	25	10.06	Mrpl13	99896	2850551	1.606062293	up
MACS_PEAK_1104	23	7.76	Mrpl13	77519	2850551	1.606062293	up
MACS_PEAK_1124	30	11.97	St3gal1	86092	5420301	1.560395718	down
MACS_PEAK_1141	26	13.33	Tnrc6b	18	101190041	1.602386117	up
MACS_PEAK_1150	18	8.48	Kif21a	21326	101340647	2.176335573	down
MACS_PEAK_1168	41	14.54	Col2a1	22032	3940500	8.106253624	down
MACS_PEAK_1168	41	14.54	Col2a1	22032	460446	1.865817666	down
MACS_PEAK_1168	41	14.54	Col2a1	22032	104610193	4.782061577	down
MACS_PEAK_1169	25	8.31	Col2a1	22908	3940500	8.106253624	down
MACS_PEAK_1169	25	8.31	Col2a1	22908	460446	1.865817666	down
MACS_PEAK_1169	25	8.31	Col2a1	22908	104610193	4.782061577	down
MACS_PEAK_1170	18	7.27	Col2a1	26360	3940500	8.106253624	down
MACS_PEAK_1170	18	7.27	Col2a1	26360	460446	1.865817666	down
MACS_PEAK_1170	18	7.27	Col2a1	26360	104610193	4.782061577	down
MACS_PEAK_1175	22	10.3	Acvr1b	8728	105670494	2.186805487	down
MACS_PEAK_1179	84	40.59	Rarg	3309	6760136	1.786343336	down
MACS_PEAK_1180	28	7.88	Rarg	1785	6760136	1.786343336	down
MACS_PEAK_1184	31	13.93	Calcoco1	45	2570452	2.164650917	up
MACS_PEAK_1188	47	19.99	Nfe2	4849	3710373	1.718353748	up
MACS_PEAK_1193	22	11.51	Vasn	12062	1770722	1.751005173	down
MACS_PEAK_1200	28	6.65	Txndc11	9411	3610278	1.92543292	down
MACS_PEAK_1200	28	6.65	Txndc11	9411	6100110	1.765415788	down
MACS_PEAK_1202	33	11.51	2900011O08Rik	84070	5050168	2.581374645	down
MACS_PEAK_1225	20	9.69	Bdh1	1128	102470619	1.856939673	down
MACS_PEAK_1239	27	8.64	Ndufb4	30583	5690594	2.297720194	up
MACS_PEAK_1240	14	7.88	Fstl1	44745	100070746	30.46147919	down
MACS_PEAK_1244	29	9.09	BC027231	58658	6840609	1.573701859	down
MACS_PEAK_1245	17	8.48	BC027231	57668	6840609	1.573701859	down
MACS_PEAK_1249	19	11.51	Ift57	54188	1190541	1.671165586	up
MACS_PEAK_1256	25	10.91	Col8a1	192761	106290072	2.177601099	up
MACS_PEAK_1257	29	10.53	Robo1	395086	2900528	1.563209176	down
MACS_PEAK_1258	27	12.12	Robo1	23276	2900528	1.563209176	down
MACS_PEAK_1259	45	17.73	Robo1	234077	2900528	1.563209176	down
MACS_PEAK_1260	16	6.06	Robo2	95607	450136	1.660531163	up
MACS_PEAK_1260	16	6.06	Robo2	95607	100770373	1.523898602	up
MACS_PEAK_1285	26	14.51	Setd4	110075	3610047	1.624795914	up
MACS_PEAK_1292	41	15.15	Erg	131306	1770739	1.973852038	down
MACS_PEAK_1293	27	7.27	Erg	121971	1770739	1.973852038	down
MACS_PEAK_1294	23	9.09	Erg	60516	1770739	1.973852038	down
MACS_PEAK_1295	21	7.88	Erg	56490	1770739	1.973852038	down
MACS_PEAK_1304	17	6.06	Snx9	70743	3290592	1.786275148	down
MACS_PEAK_1305	21	9.69	Snx9	8305	3290592	1.786275148	down
MACS_PEAK_1310	316	164.79	Fgfr1op	14619	3290433	3.044998169	down
MACS_PEAK_1330	32	12.9	Ppp2r1a	1284	2060279	1.922006249	down
MACS_PEAK_1338	24	10.91	Ppard	12153	104280632	1.571586847	down
MACS_PEAK_1343	33	11.51	Brd2	393	104760411	1.940654516	down
MACS_PEAK_1348	18	7.27	Tnfrsf21	47759	6380100	1.828891873	down
MACS_PEAK_1353	29	12.12	Cdc5l	218142	3390025	1.562691808	down
MACS_PEAK_1357	21	7.88	Ptk7	9791	380315	2.03581214	up
MACS_PEAK_1361	26	11.18	Foxp4	51373	6290022	1.508641601	up
MACS_PEAK_1388	20	7.94	Tgif1	91153	2640541	1.82399416	down
MACS_PEAK_1388	20	7.94	Tgif1	91153	2850403	3.366029501	down
MACS_PEAK_1389	67	30.29	Tgif1	65584	2640541	1.82399416	down
MACS_PEAK_1389	67	30.29	Tgif1	65584	2850403	3.366029501	down
MACS_PEAK_1390	85	8.6	Tgif1	52306	2640541	1.82399416	down
MACS_PEAK_1390	85	8.6	Tgif1	52306	2850403	3.366029501	down
MACS_PEAK_1395	19	9.09	Lbh	7783	70093	1.930043578	down
MACS_PEAK_1396	19	7.88	Lbh	75475	70093	1.930043578	down
MACS_PEAK_1401	27	12.12	Crim1	29271	103360519	2.418887138	down
MACS_PEAK_1402	26	9.09	Cdc42ep3	148721	2480138	2.477018833	down
MACS_PEAK_1402	26	9.09	Cdc42ep3	148721	101450088	1.739342928	down

MACS_PEAK_1403	16	7.27	Cdc42ep3	48949	2480138	2.477018833	down
MACS_PEAK_1403	16	7.27	Cdc42ep3	48949	101450088	1.739342928	down
MACS_PEAK_1404	16	7.88	Cdc42ep3	11903	2480138	2.477018833	down
MACS_PEAK_1404	16	7.88	Cdc42ep3	11903	101450088	1.739342928	down
MACS_PEAK_1415	23	6.65	Six2	24894	1450551	2.700210094	down
MACS_PEAK_1416	34	12.12	Six2	181268	1450551	2.700210094	down
MACS_PEAK_1417	55	20.16	Six2	182599	1450551	2.700210094	down
MACS_PEAK_1418	55	29.69	Six2	214535	1450551	2.700210094	down
MACS_PEAK_1419	36	11.25	Six2	226580	1450551	2.700210094	down
MACS_PEAK_1435	39	9.09	Fzd8	599354	1990053	1.892960548	down
MACS_PEAK_1436	28	9.09	Fzd8	568196	1990053	1.892960548	down
MACS_PEAK_1437	34	17.57	Fzd8	472575	1990053	1.892960548	down
MACS_PEAK_1438	41	14.41	Fzd8	344582	1990053	1.892960548	down
MACS_PEAK_1466	17	7.88	Cxxc5	25817	100460601	2.226545811	down
MACS_PEAK_1471	20	6.66	Yipf5	91131	2940008	1.63463676	up
MACS_PEAK_1474	47	21.2	Stk32a	68580	102510731	1.641725421	down
MACS_PEAK_1493	20	7.88	Snx2	88618	3360364	6.765829086	down
MACS_PEAK_1494	71	18.7	Snx2	78797	3360364	6.765829086	down
MACS_PEAK_1504	20	6.04	Gramd3	294752	6650546	3.307068825	down
MACS_PEAK_1505	18	9.69	Gramd3	189393	6650546	3.307068825	down
MACS_PEAK_1506	17	6.66	Gramd3	36251	6650546	3.307068825	down
MACS_PEAK_1510	58	27.36	Prrc1	83518	5870273	1.73284173	down
MACS_PEAK_1511	19	9.09	Prrc1	50562	5870273	1.73284173	down
MACS_PEAK_1515	18	7.27	Fbn2	95090	103830487	1.77337563	up
MACS_PEAK_1518	32	12.75	Synpo	24185	102680377	1.866822839	down
MACS_PEAK_1518	32	12.75	Synpo	24185	104760168	1.684028506	down
MACS_PEAK_1519	40	11.55	Ndst1	43660	102060286	1.803198338	up
MACS_PEAK_1520	53	19.39	Ndst1	42707	102060286	1.803198338	up
MACS_PEAK_1522	27	10.91	Camk2a	15055	1940112	1.617012858	up
MACS_PEAK_1530	20	9.69	Nedd4l	45637	6380368	1.516248822	down
MACS_PEAK_1531	52	20.13	Nedd4l	51011	6380368	1.516248822	down
MACS_PEAK_1532	44	15.75	Nedd4l	94600	6380368	1.516248822	down
MACS_PEAK_1552	35	9.78	Slc14a1	26051	5700154	2.209163189	down
MACS_PEAK_1552	35	9.78	Slc14a1	26051	106420044	1.935239077	down
MACS_PEAK_1552	35	9.78	Slc14a1	26051	1580458	1.719521284	down
MACS_PEAK_1553	46	16.84	Slc14a1	2782	5700154	2.209163189	down
MACS_PEAK_1553	46	16.84	Slc14a1	2782	106420044	1.935239077	down
MACS_PEAK_1553	46	16.84	Slc14a1	2782	1580458	1.719521284	down
MACS_PEAK_1561	72	32.34	Nfatc1	76226	2320348	2.426151514	down
MACS_PEAK_1561	72	32.34	Nfatc1	76226	6180161	1.97121489	down
MACS_PEAK_1561	72	32.34	Nfatc1	76226	510400	3.104269505	down
MACS_PEAK_1561	72	32.34	Nfatc1	76226	6290136	1.625146389	down
MACS_PEAK_1562	16	5.98	Nfatc1	67424	2320348	2.426151514	down
MACS_PEAK_1562	16	5.98	Nfatc1	67424	6180161	1.97121489	down
MACS_PEAK_1562	16	5.98	Nfatc1	67424	510400	3.104269505	down
MACS_PEAK_1562	16	5.98	Nfatc1	67424	6290136	1.625146389	down
MACS_PEAK_1563	42	18.18	Nfatc1	53852	2320348	2.426151514	down
MACS_PEAK_1563	42	18.18	Nfatc1	53852	6180161	1.97121489	down
MACS_PEAK_1563	42	18.18	Nfatc1	53852	510400	3.104269505	down
MACS_PEAK_1563	42	18.18	Nfatc1	53852	6290136	1.625146389	down
MACS_PEAK_1564	29	13.93	Nfatc1	2143	2320348	2.426151514	down
MACS_PEAK_1564	29	13.93	Nfatc1	2143	6180161	1.97121489	down
MACS_PEAK_1564	29	13.93	Nfatc1	2143	510400	3.104269505	down
MACS_PEAK_1564	29	13.93	Nfatc1	2143	6290136	1.625146389	down
MACS_PEAK_1576	18	8.48	Flrt1	49693	4540348	1.882780433	up
MACS_PEAK_1581	23	8.27	Psat1	97586	670446	2.637450457	down
MACS_PEAK_1582	31	15.52	Psat1	60641	670446	2.637450457	down
MACS_PEAK_1583	17	8.48	Psat1	59354	670446	2.637450457	down
MACS_PEAK_1594	63	7.34	Trpm3	132879	6400731	1.53155458	down
MACS_PEAK_1612	197	100.57	Plce1	45129	4780685	1.828465343	down
MACS_PEAK_1613	19	6	Plce1	16793	4780685	1.828465343	down
MACS_PEAK_1614	106	6.83	Plce1	3461	4780685	1.828465343	down
MACS_PEAK_1694	20	7.4	Ak1	441	130605	1.549378395	up
MACS_PEAK_1702	24	7.88	Pbx3	142237	3710577	2.450549364	down
MACS_PEAK_1715	18	5.45	Zeb2	1330791	4150215	2.707919359	up
MACS_PEAK_1731	24	7.76	Rbms1	51878	6400014	1.956834078	down
MACS_PEAK_1732	40	18.18	Fign	85836	7000601	2.133958101	down
MACS_PEAK_1733	27	13.33	Fign	74488	7000601	2.133958101	down
MACS_PEAK_1734	23	10.3	Cobll1	499	6370494	1.6672225	down
MACS_PEAK_1749	73	22.86	Ttc30b	4641	106650142	1.529625416	down
MACS_PEAK_1752	19	8.48	Ube2e3	150822	101980025	2.235800743	up
MACS_PEAK_1756	26	9.69	Ctnnd1	14829	106400139	1.712701678	up
MACS_PEAK_1761	20	9.69	Tspan18	43173	940435	2.772142649	down
MACS_PEAK_1765	19	7.27	Apip	70814	2810471	2.063208818	down
MACS_PEAK_1788	36	11.35	Spred1	56681	6940706	2.010764599	down
MACS_PEAK_1794	44	22.42	Thbs1	330757	430288	5.464723587	down
MACS_PEAK_1794	44	22.42	Thbs1	330757	4560494	5.315876007	down
MACS_PEAK_1795	20	10.91	Thbs1	317041	430288	5.464723587	down
MACS_PEAK_1795	20	10.91	Thbs1	317041	4560494	5.315876007	down
MACS_PEAK_1796	128	53.85	Thbs1	12736	430288	5.464723587	down
MACS_PEAK_1796	128	53.85	Thbs1	12736	4560494	5.315876007	down
MACS_PEAK_1804	56	23.88	2010106G01Rik	648	6040541	2.39048624	down
MACS_PEAK_1804	56	23.88	2010106G01Rik	648	6550369	2.00118947	down
MACS_PEAK_1808	66	17.21	Anapc1	114060	100610463	1.925601721	up
MACS_PEAK_1814	65	33.32	4930402H24Rik	12224	1190164	1.547660351	up
MACS_PEAK_1815	27	7.06	Smox	14835	1940398	1.739189863	down
MACS_PEAK_1815	27	7.06	Smox	14835	3390164	4.023831844	down
MACS_PEAK_1831	19	7.27	Tasp1	41036	520403	1.752894998	down
MACS_PEAK_1832	41	18.78	Flrt3	841962	70685	2.207974911	up
MACS_PEAK_1842	33	10.3	Pax1	261082	3360253	2.354618549	up
MACS_PEAK_1844	30	10.32	Entpd6	16639	6450546	1.803019047	down
MACS_PEAK_1844	30	10.32	Entpd6	16639	104050088	1.828014612	down
MACS_PEAK_1851	27	10.3	Bcl2l1	11152	1580452	1.568951726	down
MACS_PEAK_1851	27	10.3	Bcl2l1	11152	5420484	1.546980739	down
MACS_PEAK_1863	24	12.12	Tgm2	38976	5360452	1.988940001	down
MACS_PEAK_1864	28	11.51	Tgm2	6692	5360452	1.988940001	down
MACS_PEAK_1869	41	8.48	Chd6	149426	102480138	1.612116575	up
MACS_PEAK_1876	20	7.27	Rbpjl	16878	5390019	2.951968431	down
MACS_PEAK_1877	26	8.31	Zfp335	55	3520181	2.123939514	up
MACS_PEAK_1878	17	6.06	Sulf2	12779	106040113	2.373995304	up
MACS_PEAK_1913	23	7.5	Tpd52	15738	4610541	1.524386525	up
MACS_PEAK_1922	44	13.6	Pde7a	95058	2190041	4.084401608	down
MACS_PEAK_1922	44	13.6	Pde7a	95058	1690746	2.622594118	down
MACS_PEAK_1929	31	13.93	Ect2	72553	100630538	2.233487606	down
MACS_PEAK_1934	27	9.14	Fndc3b	72011	1050687	2.314380884	down
MACS_PEAK_1935	25	9.65	Fndc3b	76292	1050687	2.314380884	down
MACS_PEAK_1950	32	12.12	Bbs12	16124	106220114	1.699396372	down
MACS_PEAK_1965	30	9.98	Pcdh10	47754	3190204	1.756918907	up
MACS_PEAK_1965	30	9.98	Pcdh10	47754	940014	1.62713182	up
MACS_PEAK_1974	26	11.51	Postn	11642	450411	4.589205265	down
MACS_PEAK_1975	52	22.66	Postn	31575	450411	4.589205265	down
MACS_PEAK_1976	22	7.88	Postn	82961	450411	4.589205265	down
MACS_PEAK_1985	17	7.27	Pfn2	133591	5700026	2.781931877	down
MACS_PEAK_1989	29	12.72	P2ry1	230684	6040121	2.169948816	down
MACS_PEAK_2001	48	26.05	Shox2	278757	101850632	2.139605284	up
MACS_PEAK_2002	19	7.24	Shox2	183291	101850632	2.139605284	up
MACS_PEAK_2003	21	9.69	B3galnt1	100997	3390746	2.335953236	down
MACS_PEAK_2009	21	8.48	Gria2	160900	5080088	1.619322181	down
MACS_PEAK_2042	19	6.6	Tbx15	5156	4610411	2.563603878	down
MACS_PEAK_2058	29	11.08	Sars	33829	7100278	1.554883003	up
MACS_PEAK_2084	26	8.31	5730508B09Rik	451603	101980537	2.213041544	down
MACS_PEAK_2090	24	12.12	Lef1	7994	7100288	1.737193108	down

MACS_PEAK_2091	18	8.48	Lef1	25166	7100288	1.737193108	down
MACS_PEAK_2092	42	21.81	Bdh2	70	1770397	1.987216711	up
MACS_PEAK_2093	26	10.91	Slc39a8	79658	6860537	1.50484395	down
MACS_PEAK_2116	21	6.66	Cyr61	988	5290026	3.337931395	down
MACS_PEAK_2117	39	5.63	Cyr61	669	5290026	3.337931395	down
MACS_PEAK_2118	47	9.04	Cyr61	53749	5290026	3.337931395	down
MACS_PEAK_2119	27	12.72	Ddah1	20853	6400750	2.672152281	down
MACS_PEAK_2120	18	7.88	Ddah1	42144	6400750	2.672152281	down
MACS_PEAK_2131	30	13.93	Tox	166429	3440053	4.584356308	down
MACS_PEAK_2132	21	7.88	Tox	67932	3440053	4.584356308	down
MACS_PEAK_2133	35	7.62	Tox	567709	3440053	4.584356308	down
MACS_PEAK_2141	84	27.87	Mmp16	316602	106290731	2.130325556	up
MACS_PEAK_2160	19	10.91	Sec61b	265002	840113	1.852417946	down
MACS_PEAK_2166	32	9.28	Smc2	569641	4810133	2.254477501	down
MACS_PEAK_2178	39	19.72	Rgs3	43789	60670	1.874791026	down
MACS_PEAK_2179	33	10.3	Rgs3	54619	60670	1.874791026	down
MACS_PEAK_2188	48	9.02	Ptprd	2062221	3120097	1.835583448	up
MACS_PEAK_2191	24	11.51	Mpdz	219854	4050577	1.670507431	down
MACS_PEAK_2191	24	11.51	Mpdz	219854	1990064	2.104254484	down
MACS_PEAK_2192	25	9.87	Mpdz	109327	4050577	1.670507431	down
MACS_PEAK_2192	25	9.87	Mpdz	109327	1990064	2.104254484	down
MACS_PEAK_2193	37	9.98	Mpdz	185853	4050577	1.670507431	down
MACS_PEAK_2193	37	9.98	Mpdz	185853	1990064	2.104254484	down
MACS_PEAK_2194	22	9.09	Mpdz	484008	4050577	1.670507431	down
MACS_PEAK_2194	22	9.09	Mpdz	484008	1990064	2.104254484	down
MACS_PEAK_2234	48	24.84	Ror1	119057	6220026	1.958731174	down
MACS_PEAK_2274	43	19.39	Bsdc1	10008	4670484	2.169983625	up
MACS_PEAK_2282	65	25.4	Pafah2	422	6510014	1.688534498	down
MACS_PEAK_2286	30	8.96	Clic4	25614	3290368	3.674860239	down
MACS_PEAK_2286	30	8.96	Clic4	25614	6450181	4.74880743	down
MACS_PEAK_2287	32	11.84	Srrm1	15465	6110025	1.615817308	up
MACS_PEAK_2298	28	12.19	Arhgef19	2182	940102	1.560842991	down
MACS_PEAK_2312	41	17.57	Rere	91065	3940279	1.924082041	up
MACS_PEAK_2325	20	6.6	Cdk6	55999	4920253	1.7740798	down
MACS_PEAK_2336	29	11.51	Sema3e	65519	3870707	1.784626603	down
MACS_PEAK_2353	23	10.91	Fgfr3	29252	6020021	1.774785995	down
MACS_PEAK_2353	23	10.91	Fgfr3	29252	5390632	5.09095192	down
MACS_PEAK_2361	21	7.76	Rab28	620331	101990458	2.852120638	down
MACS_PEAK_2361	21	7.76	Rab28	620331	100070053	1.852225065	down
MACS_PEAK_2363	22	6.66	Ldb2	110571	5670441	2.367152691	down
MACS_PEAK_2364	24	11.51	Ldb2	101425	5670441	2.367152691	down
MACS_PEAK_2366	172	65.43	Gpr125	63902	6380025	2.239007235	down
MACS_PEAK_2367	20	7.76	Gpr125	137712	6380025	2.239007235	down
MACS_PEAK_2372	24	9.69	Pcdh7	245039	104780333	1.514340878	up
MACS_PEAK_2399	22	9.98	Chic2	33603	101980039	1.743302584	down
MACS_PEAK_2404	21	8.48	Lphn3	880742	106400315	1.745946884	down
MACS_PEAK_2404	21	8.48	Lphn3	880742	103060309	1.831612229	down
MACS_PEAK_2405	29	10.85	Lphn3	134615	106400315	1.745946884	down
MACS_PEAK_2405	29	10.85	Lphn3	134615	103060309	1.831612229	down
MACS_PEAK_2406	26	12.72	Lphn3	197334	106400315	1.745946884	down
MACS_PEAK_2406	26	12.72	Lphn3	197334	103060309	1.831612229	down
MACS_PEAK_2407	45	20.6	Lphn3	204022	106400315	1.745946884	down
MACS_PEAK_2407	45	20.6	Lphn3	204022	103060309	1.831612229	down
MACS_PEAK_2408	41	17.57	Lphn3	302955	106400315	1.745946884	down
MACS_PEAK_2408	41	17.57	Lphn3	302955	103060309	1.831612229	down
MACS_PEAK_2420	49	21.81	Aff1	17775	100780731	2.008762598	up
MACS_PEAK_2426	40	8.48	Lrrc8d	19358	1780014	1.506517887	down
MACS_PEAK_2427	32	13.93	Lrrc8d	15192	1780014	1.506517887	down
MACS_PEAK_2492	18	6.66	Zfp113	29	1770193	2.064691067	up
MACS_PEAK_2495	19	7.88	Uncx	75869	2060142	1.693639398	up
MACS_PEAK_2510	31	7.62	Hmgb1	47816	102640685	2.085407734	down
MACS_PEAK_2528	27	13.33	Asns	40397	110368	3.113725662	down
MACS_PEAK_2530	31	12.12	Glcci1	88205	3290070	2.098738909	down
MACS_PEAK_2548	24	9.09	Klhdc10	260	3870047	2.631810665	down
MACS_PEAK_2558	49	23.02	Plxna4	148501	102450484	4.854547024	up
MACS_PEAK_2564	27	8.48	Ptn	35983	5910161	1.975698113	down
MACS_PEAK_2565	30	6.1	Ptn	26324	5910161	1.975698113	down
MACS_PEAK_2574	160	3.58	Hipk2	6720	6350647	2.047196627	up
MACS_PEAK_2576	29	14.54	Igf2bp3	21559	2810161	1.510372758	up
MACS_PEAK_2585	77	35.14	Hoxa9	752	4730040	1.513581157	down
MACS_PEAK_2588	119	22.99	Scrn1	11833	6580019	1.527701139	down
MACS_PEAK_2594	70	34.53	Gng12	35473	100060446	1.896968961	down
MACS_PEAK_2598	36	11.51	Tgoln1	69780	4920433	2.579447985	down
MACS_PEAK_2598	36	11.51	Tgoln1	69780	3060270	1.883389592	down
MACS_PEAK_2606	31	12.75	Rtkn	71	6380082	1.949686885	down
MACS_PEAK_2614	21	6.64	Mgll	8545	2030446	1.527316213	down
MACS_PEAK_2615	46	8.94	Mgll	26346	2030446	1.527316213	down
MACS_PEAK_2618	28	8.53	Fbln2	11670	3440215	2.287607193	down
MACS_PEAK_2635	30	12.72	Pdzrn3	114272	2340131	1.666963577	up
MACS_PEAK_2636	24	8.13	Pdzrn3	69647	2340131	1.666963577	up
MACS_PEAK_2637	22	7.84	Pdzrn3	95517	2340131	1.666963577	up
MACS_PEAK_2644	26	11.51	Lmcd1	37814	1740484	2.61991334	down
MACS_PEAK_2663	18	6.66	Cd9	27240	4730041	2.94108367	down
MACS_PEAK_2664	51	9.38	Cd9	26427	4730041	2.94108367	down
MACS_PEAK_2665	42	4.12	Ccnd2	101100	102350114	1.814499736	up
MACS_PEAK_2679	51	19.99	Eps8	95042	7050204	2.723695993	down
MACS_PEAK_2680	21	9.69	Eps8	15135	7050204	2.723695993	down
MACS_PEAK_2681	22	10.45	Eps8	11685	7050204	2.723695993	down
MACS_PEAK_2684	39	18.78	Pde3a	115022	105860180	1.686952591	down
MACS_PEAK_2692	77	39.99	Sox5	228314	3190128	3.311142206	down
MACS_PEAK_2692	77	39.99	Sox5	228314	2370576	4.999372005	down
MACS_PEAK_2692	77	39.99	Sox5	228314	2900167	8.529214859	down
MACS_PEAK_2693	115	29.46	Sox5	170159	3190128	3.311142206	down
MACS_PEAK_2693	115	29.46	Sox5	170159	2370576	4.999372005	down
MACS_PEAK_2693	115	29.46	Sox5	170159	2900167	8.529214859	down
MACS_PEAK_2694	23	7.27	Sox5	152690	3190128	3.311142206	down
MACS_PEAK_2694	23	7.27	Sox5	152690	2370576	4.999372005	down
MACS_PEAK_2694	23	7.27	Sox5	152690	2900167	8.529214859	down
MACS_PEAK_2695	26	11.51	Sox5	127093	3190128	3.311142206	down
MACS_PEAK_2695	26	11.51	Sox5	127093	2370576	4.999372005	down
MACS_PEAK_2695	26	11.51	Sox5	127093	2900167	8.529214859	down
MACS_PEAK_2696	21	7.27	Sox5	43306	3190128	3.311142206	down
MACS_PEAK_2696	21	7.27	Sox5	43306	2370576	4.999372005	down
MACS_PEAK_2696	21	7.27	Sox5	43306	2900167	8.529214859	down
MACS_PEAK_2697	19	7.27	Sox5	39678	3190128	3.311142206	down
MACS_PEAK_2697	19	7.27	Sox5	39678	2370576	4.999372005	down
MACS_PEAK_2697	19	7.27	Sox5	39678	2900167	8.529214859	down
MACS_PEAK_2698	51	17.57	Sox5	11700	3190128	3.311142206	down
MACS_PEAK_2698	51	17.57	Sox5	11700	2370576	4.999372005	down
MACS_PEAK_2698	51	17.57	Sox5	11700	2900167	8.529214859	down
MACS_PEAK_2699	94	28.26	Sox5	602	3190128	3.311142206	down
MACS_PEAK_2699	94	28.26	Sox5	602	2370576	4.999372005	down
MACS_PEAK_2699	94	28.26	Sox5	602	2900167	8.529214859	down
MACS_PEAK_2700	39	12.12	Sox5	54782	3190128	3.311142206	down
MACS_PEAK_2700	39	12.12	Sox5	54782	2370576	4.999372005	down
MACS_PEAK_2700	39	12.12	Sox5	54782	2900167	8.529214859	down
MACS_PEAK_2701	41	12.12	Sox5	103287	3190128	3.311142206	down
MACS_PEAK_2701	41	12.12	Sox5	103287	2370576	4.999372005	down
MACS_PEAK_2701	41	12.12	Sox5	103287	2900167	8.529214859	down
MACS_PEAK_2707	20	8.48	Kras	8758	1240026	1.503148794	up
MACS_PEAK_2708	29	9.85	Tuba3b	10444	5290521	1.886989355	up
MACS_PEAK_2724	31	12.12	Mia1	320	5700273	12.40242386	down

MACS_PEAK_2726	33	14.54	Ech1	4806	4120746	1.582007647	up
MACS_PEAK_2727	17	6.06	Actn4	38227	4590390	2.610746384	down
MACS_PEAK_2727	17	6.06	Actn4	38227	7050132	7.594707966	down
MACS_PEAK_2727	17	6.06	Actn4	38227	3840301	1.867175221	down
MACS_PEAK_2762	15	7.88	St8sia2	104938	2680082	1.841214657	up
MACS_PEAK_2763	18	6.66	St8sia2	160684	2680082	1.841214657	up
MACS_PEAK_2767	19	8.48	Akap13	119844	101450594	1.573993444	up
MACS_PEAK_2795	40	5.23	Wnt11	138437	103840154	1.609444022	up
MACS_PEAK_2795	40	5.23	Wnt11	138437	1230278	1.79390502	up
MACS_PEAK_2807	24	9.69	Tpp1	15536	3990446	1.962062359	up
MACS_PEAK_2813	22	10.3	Usp47	60141	105900047	1.575356126	down
MACS_PEAK_2819	30	8.76	Sox6	623750	6840717	3.285536766	down
MACS_PEAK_2819	30	8.76	Sox6	623750	4070601	1.792667627	down
MACS_PEAK_2820	19	6.06	Sox6	606397	6840717	3.285536766	down
MACS_PEAK_2820	19	6.06	Sox6	606397	4070601	1.792667627	down
MACS_PEAK_2821	67	15.15	Sox6	520828	6840717	3.285536766	down
MACS_PEAK_2821	67	15.15	Sox6	520828	4070601	1.792667627	down
MACS_PEAK_2822	104	26.05	Sox6	379119	6840717	3.285536766	down
MACS_PEAK_2822	104	26.05	Sox6	379119	4070601	1.792667627	down
MACS_PEAK_2823	19	7.27	Sox6	370044	6840717	3.285536766	down
MACS_PEAK_2823	19	7.27	Sox6	370044	4070601	1.792667627	down
MACS_PEAK_2824	23	8.48	Sox6	325646	6840717	3.285536766	down
MACS_PEAK_2824	23	8.48	Sox6	325646	4070601	1.792667627	down
MACS_PEAK_2825	34	8.48	Sox6	170346	6840717	3.285536766	down
MACS_PEAK_2825	34	8.48	Sox6	170346	4070601	1.792667627	down
MACS_PEAK_2826	30	6.65	Sox6	129107	6840717	3.285536766	down
MACS_PEAK_2826	30	6.65	Sox6	129107	4070601	1.792667627	down
MACS_PEAK_2827	21	10.3	Sox6	50460	6840717	3.285536766	down
MACS_PEAK_2827	21	10.3	Sox6	50460	4070601	1.792667627	down
MACS_PEAK_2841	63	30.29	Rgs10	6904	2340292	2.4998281	up
MACS_PEAK_2848	17	5.45	Plekha1	31372	1780059	1.997643352	down
MACS_PEAK_2867	21	9.09	Tssc4	47	3140731	1.620042682	down
MACS_PEAK_2878	27	13.33	Efnb2	800417	5340136	2.006764174	up
MACS_PEAK_2879	198	104.21	Efnb2	350258	5340136	2.006764174	up
MACS_PEAK_2880	52	23.02	Efnb2	41034	5340136	2.006764174	up
MACS_PEAK_2889	48	6.81	Xkr5	515	2230441	1.539257407	up
MACS_PEAK_2892	23	10.3	1810011O10Rik	6879	104670091	1.849944472	up
MACS_PEAK_2896	37	18.78	Prosc	4670	380484	4.061861992	down
MACS_PEAK_2899	29	11.51	Lonrf1	33745	100670136	2.00988245	down
MACS_PEAK_2905	17	6.06	Mtus1	39846	780348	1.660579801	up
MACS_PEAK_2916	22	7.88	Slc25a4	40227	2360519	2.12780714	up
MACS_PEAK_2926	19	6.66	Scrg1	54069	2060541	1.8617239	down
MACS_PEAK_2928	43	11.79	Sh3rf1	77693	1190463	1.549773216	up
MACS_PEAK_2939	56	32.11	Ints10	178	2120609	1.54038012	up
MACS_PEAK_2944	72	30.9	Rab8a	3102	6020736	1.560746551	up
MACS_PEAK_2944	72	30.9	Rab8a	3102	3610594	1.825872421	up
MACS_PEAK_2948	70	36.35	Zfp827	50988	5670021	2.582318783	up
MACS_PEAK_2959	23	10.91	Lyl1	27125	2900242	2.016277552	up
MACS_PEAK_2960	150	76.05	Nfix	35960	2450152	1.575346112	down
MACS_PEAK_2961	26	7.5	Nfix	30148	2450152	1.575346112	down
MACS_PEAK_2962	264	147.83	Lonp2	20578	5290687	1.659163117	up
MACS_PEAK_2968	22	12.72	Cyld	95809	101660066	2.369335175	up
MACS_PEAK_3000	22	10.91	Zfhx3	41132	7040465	1.900578856	up
MACS_PEAK_3003	30	10.91	Mon1b	245818	102480270	1.941350937	up
MACS_PEAK_3036	35	6.21	Maml2	38588	102100093	1.647469759	down
MACS_PEAK_3037	28	11.68	Maml2	3242	102100093	1.647469759	down
MACS_PEAK_3041	35	16.96	Fat3	150057	105670446	2.421111107	up
MACS_PEAK_3047	37	15.1	Bbs9	54130	4540368	1.631353974	down
MACS_PEAK_3061	39	15.94	Kirrel3	69351	2630195	1.939929843	up
MACS_PEAK_3064	47	12.72	Cdon	46199	104810102	1.829356194	down
MACS_PEAK_3065	21	9.69	Cdon	41577	104810102	1.829356194	down
MACS_PEAK_3077	29	13.33	Dpagt1	289	1850324	1.541120768	up
MACS_PEAK_3084	24	9.69	Ncam1	19	105690114	1.675677061	up
MACS_PEAK_3090	64	8.94	Ppcdc	19684	1410619	1.588259459	up
MACS_PEAK_3097	21	7.88	Adpgk	285	4200164	1.868386626	up
MACS_PEAK_3101	25	8.48	Tle3	87198	105270110	1.855359912	up
MACS_PEAK_3102	42	20.6	Tle3	21070	105270110	1.855359912	up
MACS_PEAK_3103	84	30.48	Tle3	93	105270110	1.855359912	up
MACS_PEAK_3104	19	9.69	Tle3	33093	105270110	1.855359912	up
MACS_PEAK_3105	30	10.3	Tle3	167042	105270110	1.855359912	up
MACS_PEAK_3106	22	10.91	Tle3	177533	105270110	1.855359912	up
MACS_PEAK_3107	36	8.61	Tle3	207365	105270110	1.855359912	up
MACS_PEAK_3108	27	7.32	Tle3	246197	105270110	1.855359912	up
MACS_PEAK_3127	16	8.48	Myo1e	62199	105890411	2.609994411	up
MACS_PEAK_3128	18	8.48	Myo1e	74220	105890411	2.609994411	up
MACS_PEAK_3134	68	28.96	Unc13c	37362	106350110	1.754839301	up
MACS_PEAK_3143	18	6.66	Cd109	264886	130014	1.82360971	down
MACS_PEAK_3143	18	6.66	Cd109	264886	540152	1.708335042	down
MACS_PEAK_3144	24	12.12	Cd109	310704	130014	1.82360971	down
MACS_PEAK_3144	24	12.12	Cd109	310704	540152	1.708335042	down
MACS_PEAK_3152	15	8.48	Tmed3	30704	6940605	2.500232935	down
MACS_PEAK_3159	123	55.74	Acpl2	34328	1690736	1.52359724	down
MACS_PEAK_3159	123	55.74	Acpl2	34328	6200364	2.482869387	down
MACS_PEAK_3160	23	10.3	Spsb4	56038	4070673	1.836065888	down
MACS_PEAK_3181	24	6.58	Ptpn23	23484	130110	2.744419813	up
MACS_PEAK_3185	21	6.06	Susd5	14679	103830014	1.583449841	down
MACS_PEAK_3186	25	5.63	Susd5	55132	103830014	1.583449841	down
MACS_PEAK_3187	18	7.88	Tgfbr2	253205	6550398	2.211287975	down
MACS_PEAK_3188	31	11.68	Tgfbr2	469272	6550398	2.211287975	down
MACS_PEAK_3208	26	8.87	Deb1	1248	5720082	1.929145098	down
MACS_PEAK_3246	47	21.81	Diap2	397199	100840398	1.756209016	up
MACS_PEAK_3253	18	7.88	Sh3kbp1	13415	2850458	2.331632614	down
MACS_PEAK_3254	21	12.72	Sh3kbp1	16	2850458	2.331632614	down
MACS_PEAK_2	34	13.33	Rrs1	19345	4560551	1.514943242	up
MACS_PEAK_7	23	9.47	Stau2	269866	2810411	1.965145826	down
MACS_PEAK_7	23	9.47	Stau2	269866	100110041	3.7445755	down
MACS_PEAK_48	27	11.51	9130024F11Rik	124768	101980091	1.864141822	down
MACS_PEAK_107	22	10.3	St8sia4	633953	3060215	2.122776031	up
MACS_PEAK_113	21	6.1	Vps4b	36738	3440332	2.351050854	up
MACS_PEAK_122	43	14.07	Marco	147525	870056	2.083778143	up
MACS_PEAK_130	37	16.36	Thsd7b	623626	2570750	1.682179809	up
MACS_PEAK_150	36	13.85	Tsen15	21094	430270	2.167198658	down
MACS_PEAK_151	28	12.12	Tsen15	68752	430270	2.167198658	down
MACS_PEAK_157	29	9.09	Glul	290270	6040253	2.251183271	up
MACS_PEAK_190	18	7.88	Efcab2	327294	3190358	1.514025807	down
MACS_PEAK_191	99	34.4	Efcab2	415365	3190358	1.514025807	down
MACS_PEAK_195	14	7.88	Itpkb	63135	102100373	1.975757837	up
MACS_PEAK_196	17	8.48	Itpkb	73660	102100373	1.975757837	up
MACS_PEAK_212	34	13.33	Sertad4	115060	130593	1.795625091	down
MACS_PEAK_220	26	12.12	Ust	431639	104060253	1.561433434	up
MACS_PEAK_236	56	29.69	Ctgf	214349	4540577	4.026652813	down
MACS_PEAK_237	33	13.93	Moxd1	235263	2450301	3.239943504	down
MACS_PEAK_238	76	35.74	Ctgf	69465	4540577	4.026652813	down
MACS_PEAK_275	14	8.48	Ascc3	1138937	6550132	1.935636044	down
MACS_PEAK_285	21	7.27	Chst3	67957	2370131	1.71955502	down
MACS_PEAK_305	39	15.75	Bsg	8139	2900193	2.248334885	down
MACS_PEAK_309	63	10.03	Chst11	30180	6760546	1.705717921	down
MACS_PEAK_321	17	7.88	BC030307	95415	105050575	1.571127295	up
MACS_PEAK_323	26	7.27	Tmpo	687637	102060411	4.569718361	down
MACS_PEAK_323	26	7.27	Tmpo	687637	105670161	1.981946707	down
MACS_PEAK_323	26	7.27	Tmpo	687637	102760300	5.473426819	down
MACS_PEAK_324	24	8.53	Tmpo	864763	102060411	4.569718361	down

MACS_PEAK_324	24	8.53	Tmpo	864763	105670161	1.981946707	down
MACS_PEAK_324	24	8.53	Tmpo	864763	102760300	5.473426819	down
MACS_PEAK_331	30	7.34	Btg1	491001	6200133	16.80504227	down
MACS_PEAK_333	18	7.88	Dcn	653063	5900711	6.7773633	down
MACS_PEAK_333	18	7.88	Dcn	653063	6550092	2.875388622	down
MACS_PEAK_334	19	6.1	Btg1	696764	6200133	16.80504227	down
MACS_PEAK_340	21	9.69	Slc6a15	612724	6040017	3.621177435	down
MACS_PEAK_344	20	7.27	Krr1	455521	6130504	3.11702013	down
MACS_PEAK_345	37	14.07	Krr1	379701	6130504	3.11702013	down
MACS_PEAK_351	19	8.31	Ptprb	69936	5220053	2.290420294	up
MACS_PEAK_355	33	4.62	Cpsf6	210019	100780500	1.602885008	up
MACS_PEAK_362	25	10.3	Hmga2	99432	5130279	2.021983624	up
MACS_PEAK_363	32	9.65	Hmga2	59593	5130279	2.021983624	up
MACS_PEAK_365	28	7.72	Hmga2	275019	5130279	2.021983624	up
MACS_PEAK_367	20	6.28	Wif1	172278	7100184	2.992610455	down
MACS_PEAK_368	55	16.96	Wif1	217675	7100184	2.992610455	down
MACS_PEAK_385	17	8.48	Zmiz2	10570	5700382	1.840798736	up
MACS_PEAK_389	22	9.09	Tns3	285633	6380242	1.662731886	down
MACS_PEAK_390	44	18.18	Tns3	294179	6380242	1.662731886	down
MACS_PEAK_411	29	12.72	Ccdc99	370719	4210091	1.664839625	down
MACS_PEAK_426	17	5.45	Mapk9	51715	4070397	4.267343521	down
MACS_PEAK_427	19	6.1	Gfpt2	51075	2370129	2.037197351	down
MACS_PEAK_428	19	6.66	Mapk9	31010	4070397	4.267343521	down
MACS_PEAK_435	48	26.05	Gm2a	27079	430095	1.636600494	up
MACS_PEAK_436	31	16.36	Gria1	422660	100580563	1.693127275	up
MACS_PEAK_446	46	12.12	Pmp22	47213	6550072	12.38402176	down
MACS_PEAK_446	46	12.12	Pmp22	47213	4010239	2.244052887	down
MACS_PEAK_447	40	16.36	Hs3st3b1	458330	105550114	1.865917325	down
MACS_PEAK_447	40	16.36	Hs3st3b1	458330	101340047	2.216298819	down
MACS_PEAK_470	189	96.98	Evi2a	53854	6840110	2.567021608	up
MACS_PEAK_480	27	10.3	Dhx40	50027	360672	1.63516438	up
MACS_PEAK_502	68	26.05	Col1a1	19068	730020	1.851671576	up
MACS_PEAK_512	22	8.48	Vat1	11272	1050040	2.656697989	up
MACS_PEAK_531	43	21.2	Kcnj2	978397	630019	2.289093971	down
MACS_PEAK_546	31	9.09	Wbp2	17615	3170128	1.767673254	down
MACS_PEAK_549	40	17.18	2810008D09Rik	30336	106220711	1.630763769	up
MACS_PEAK_550	27	4.57	2810008D09Rik	32062	106220711	1.630763769	up
MACS_PEAK_551	55	25.45	Sec14l1	170303	3610537	1.908110738	up
MACS_PEAK_567	47	22.42	Rhob	113472	1500309	1.795479774	up
MACS_PEAK_593	31	16.36	Ttc15	54951	106770341	1.531815052	up
MACS_PEAK_603	20	8.48	Snx13	142816	102340142	1.833219647	down
MACS_PEAK_638	21	7.88	Dact1	319209	1500050	2.195098639	down
MACS_PEAK_638	21	7.88	Dact1	319209	100630088	2.167698622	down
MACS_PEAK_649	25	6.66	Fut8	850585	2340056	2.135364532	down
MACS_PEAK_649	25	6.66	Fut8	850585	1340068	1.797354579	down
MACS_PEAK_750	26	11.51	Foxf2	94237	1240091	2.093223333	up
MACS_PEAK_751	96	48.19	Foxf2	139740	1240091	2.093223333	up
MACS_PEAK_752	48	19.95	Foxf2	150135	1240091	2.093223333	up
MACS_PEAK_766	75	37.56	Wrnip1	96580	5890725	4.550052166	down
MACS_PEAK_768	22	5.98	Tubb2b	18536	1230500	9.238461494	down
MACS_PEAK_783	17	6.66	Rnf182	124078	3140537	1.947802186	down
MACS_PEAK_797	32	12.12	Barx1	56811	7050075	5.78196907	down
MACS_PEAK_813	17	7.76	Tgfbi	90954	2060446	2.340261459	down
MACS_PEAK_814	24	9.6	Zcchc6	276849	102450717	2.124472618	up
MACS_PEAK_815	27	7.84	Dapk1	264368	101940079	1.667805314	up
MACS_PEAK_816	21	10.3	Dapk1	200652	101940079	1.667805314	up
MACS_PEAK_825	19	8.24	Fancc	107316	2060022	1.544645429	up
MACS_PEAK_826	20	7.27	Fancc	125315	2060022	1.544645429	up
MACS_PEAK_827	22	4.69	0610007P08Rik	172070	105720600	1.747234583	down
MACS_PEAK_827	22	4.69	0610007P08Rik	172070	106840121	1.564627409	down
MACS_PEAK_847	24	9.69	Tmem161b	480312	6590673	1.719977498	up
MACS_PEAK_848	20	9.69	Tmem161b	349255	6590673	1.719977498	up
MACS_PEAK_849	22	9.69	Rasa1	615817	104590722	1.986502409	down
MACS_PEAK_850	22	10.91	Hapln1	564349	580398	6.735909462	down
MACS_PEAK_851	23	9.69	Edil3	514966	101940605	1.737264156	down
MACS_PEAK_852	20	7.88	Edil3	573609	101940605	1.737264156	down
MACS_PEAK_853	27	9.69	Edil3	699930	101940605	1.737264156	down
MACS_PEAK_854	28	12.72	Hapln1	128723	580398	6.735909462	down
MACS_PEAK_863	21	7.24	Atg10	146993	2470066	2.110445023	down
MACS_PEAK_864	21	7.27	Atg10	207709	2470066	2.110445023	down
MACS_PEAK_883	59	20.03	Pik3r1	703296	4730671	2.600860834	down
MACS_PEAK_884	29	13.93	Pik3r1	528553	4730671	2.600860834	down
MACS_PEAK_885	21	9.69	Pik3r1	476465	4730671	2.600860834	down
MACS_PEAK_886	30	7.88	Slc30a5	594774	2970403	3.024980068	down
MACS_PEAK_886	30	7.88	Slc30a5	594774	2630288	2.769304037	down
MACS_PEAK_887	23	10.3	Slc30a5	661496	2970403	3.024980068	down
MACS_PEAK_887	23	10.3	Slc30a5	661496	2630288	2.769304037	down
MACS_PEAK_928	33	15.15	Adk	410029	380338	3.20015502	down
MACS_PEAK_929	44	15.24	Adk	423551	380338	3.20015502	down
MACS_PEAK_933	18	7.27	Kcnma1	625375	100110338	2.169963598	up
MACS_PEAK_937	31	9.91	Rps24	508743	102690538	1.870081425	down
MACS_PEAK_938	26	9.69	Rps24	679395	102690538	1.870081425	down
MACS_PEAK_939	18	9.09	Rps24	816602	102690538	1.870081425	down
MACS_PEAK_940	19	7.2	Rps24	827770	102690538	1.870081425	down
MACS_PEAK_941	32	15.75	Rps24	881371	102690538	1.870081425	down
MACS_PEAK_949	20	9.09	Ccdc66	301597	5220270	1.524603486	down
MACS_PEAK_960	28	7.88	Bmp4	333370	100520100	1.95533061	down
MACS_PEAK_961	36	17.57	Gch1	137123	670364	1.952455997	up
MACS_PEAK_972	17	6.66	Nfatc4	28318	2470735	1.957414746	up
MACS_PEAK_977	36	14.8	Xpo4	37078	3840551	2.114598036	down
MACS_PEAK_980	35	15.38	Dleu7	418280	2320402	1.752730727	down
MACS_PEAK_993	17	7.88	Bmp1	48910	380594	1.86990571	up
MACS_PEAK_993	17	7.88	Bmp1	48910	2940576	1.60881567	up
MACS_PEAK_1010	45	18.29	1300010F03Rik	257933	4570253	1.801385999	up
MACS_PEAK_1011	18	9.69	1300010F03Rik	286103	4570253	1.801385999	up
MACS_PEAK_1013	48	15.75	Sugt1	72873	1690670	2.026542902	up
MACS_PEAK_1015	24	9.14	Lect1	41696	2640528	1.574525833	down
MACS_PEAK_1028	42	21.2	Commd6	146195	105390717	1.578427196	down
MACS_PEAK_1034	48	26.05	Dct	1110518	1090347	1.577306986	down
MACS_PEAK_1034	48	26.05	Dct	1110518	3840494	1.660903931	down
MACS_PEAK_1035	17	7.88	Dct	1087133	1090347	1.577306986	down
MACS_PEAK_1035	17	7.88	Dct	1087133	3840494	1.660903931	down
MACS_PEAK_1036	31	15.75	Dct	1015957	1090347	1.577306986	down
MACS_PEAK_1036	31	15.75	Dct	1015957	3840494	1.660903931	down
MACS_PEAK_1039	25	7.88	Mbnl2	128403	3450707	1.756134391	down
MACS_PEAK_1040	25	11.51	Mbnl2	126588	3450707	1.756134391	down
MACS_PEAK_1044	128	67.25	Mbnl2	107761	3450707	1.756134391	down
MACS_PEAK_1045	19	8.48	Mbnl2	112849	3450707	1.756134391	down
MACS_PEAK_1083	28	10.91	Mtdh	129965	2510541	1.778023362	down
MACS_PEAK_1084	21	9.69	Ywhaz	173506	1230717	2.80567503	up
MACS_PEAK_1085	44	21.81	Ywhaz	126997	1230717	2.80567503	up
MACS_PEAK_1095	19	6.66	Trps1	224075	870100	2.253350019	down
MACS_PEAK_1096	35	9.98	Trps1	193354	870100	2.253350019	down
MACS_PEAK_1097	64	29.69	Trps1	612591	870100	2.253350019	down
MACS_PEAK_1098	15	6.66	Trps1	614923	870100	2.253350019	down
MACS_PEAK_1099	38	10.63	Trps1	778427	870100	2.253350019	down
MACS_PEAK_1100	27	11.51	Utp23	78619	60711	1.532169223	down
MACS_PEAK_1102	48	22.42	Mrpl13	187083	2850551	1.606062293	up
MACS_PEAK_1103	25	10.06	Col14a1	149666	1570398	2.242420435	down
MACS_PEAK_1104	23	7.76	Col14a1	172043	1570398	2.242420435	down
MACS_PEAK_1114	35	14.54	E430025E21Rik	14354	3060017	1.801285028	up
MACS_PEAK_1118	23	7.76	Myc	800879	380541	1.514363527	down

MACS_PEAK_1140	25	10.91	Tnrc6b	31600	101190041	1.602386117	up
MACS_PEAK_1168	41	14.54	Pfkm	65833	5720168	4.260520458	down
MACS_PEAK_1169	25	8.31	Pfkm	64957	5720168	4.260520458	down
MACS_PEAK_1170	18	7.27	Pfkm	61505	5720168	4.260520458	down
MACS_PEAK_1171	20	9.42	Lmbr1l	10658	2190403	1.618604183	up
MACS_PEAK_1175	22	10.3	Grasp	41354	3940450	1.931490898	down
MACS_PEAK_1208	227	7.15	Tbx1	73353	6590121	2.809188366	up
MACS_PEAK_1209	44	13.93	Tbx1	53697	6590121	2.809188366	up
MACS_PEAK_1222	26	9.09	Opa1	314602	3520390	1.51439774	down
MACS_PEAK_1222	26	9.09	Opa1	314602	4730537	1.844746113	down
MACS_PEAK_1222	26	9.09	Opa1	314602	2030433	1.719260216	down
MACS_PEAK_1226	26	12.72	Lrrc33	34011	2510184	3.905851841	up
MACS_PEAK_1238	30	9.98	Dtx3l	42696	1050168	1.838681459	up
MACS_PEAK_1239	27	8.64	Fstl1	92104	100070746	30.46147919	down
MACS_PEAK_1240	14	7.88	Ndufb4	77942	5690594	2.297720194	up
MACS_PEAK_1243	34	7.84	BC027231	108522	6840609	1.573701859	down
MACS_PEAK_1261	35	14.54	Robo2	601834	450136	1.660531163	up
MACS_PEAK_1261	35	14.54	Robo2	601834	100770373	1.523898602	up
MACS_PEAK_1269	31	14.54	Atp5j	206032	6900288	1.566173673	up
MACS_PEAK_1270	41	14.54	Atp5j	210919	6900288	1.566173673	up
MACS_PEAK_1271	28	13.93	Atp5j	292849	6900288	1.566173673	up
MACS_PEAK_1276	18	7.27	Ifnar2	76195	7100484	1.985054493	down
MACS_PEAK_1288	25	9.09	Pigp	101700	5890440	1.88066709	down
MACS_PEAK_1296	175	60.34	Erg	173620	1770739	1.973852038	down
MACS_PEAK_1306	34	12.75	Snx9	123961	3290592	1.786275148	down
MACS_PEAK_1322	30	9.85	Thbs2	273668	2850136	1.530437708	down
MACS_PEAK_1332	28	11.84	Sox8	6810	580026	1.502229095	down
MACS_PEAK_1333	21	10.91	Sox8	38330	580026	1.502229095	down
MACS_PEAK_1334	47	18.29	Sox8	64859	580026	1.502229095	down
MACS_PEAK_1344	26	13.93	Notch4	22585	6370707	1.774282813	up
MACS_PEAK_1354	26	12.72	Aars2	29720	102450609	1.886784554	up
MACS_PEAK_1355	31	7.03	Hsp90ab1	11295	5390102	3.361030817	down
MACS_PEAK_1355	31	7.03	Hsp90ab1	11295	6040093	5.004231453	down
MACS_PEAK_1360	47	21.2	Foxp4	60972	6290022	1.508641601	up
MACS_PEAK_1362	92	38.1	Foxp4	125431	6290022	1.508641601	up
MACS_PEAK_1368	22	9.69	Kat2b	23051	6550451	1.971818686	down
MACS_PEAK_1368	22	9.69	Kat2b	23051	2570369	1.707809448	down
MACS_PEAK_1377	24	12.72	Ppp4r1	39514	5670088	2.129499674	down
MACS_PEAK_1387	24	7.27	Tgif1	212864	2640541	1.82399416	down
MACS_PEAK_1387	24	7.27	Tgif1	212864	2850403	3.366029501	down
MACS_PEAK_1391	49	18.18	Tgif1	110178	2640541	1.82399416	down
MACS_PEAK_1391	49	18.18	Tgif1	110178	2850403	3.366029501	down
MACS_PEAK_1397	34	10.91	Galnt14	101163	3990278	1.679883838	up
MACS_PEAK_1401	27	12.12	Fez2	188584	5910181	1.603789568	down
MACS_PEAK_1432	26	9.09	Fzd8	1222458	1990053	1.892960548	down
MACS_PEAK_1433	25	8.48	Fzd8	885858	1990053	1.892960548	down
MACS_PEAK_1434	33	17.57	Fzd8	884747	1990053	1.892960548	down
MACS_PEAK_1440	53	8.91	Rock1	147212	130044	3.036412716	down
MACS_PEAK_1446	40	15.47	Riok3	102506	7040026	2.018571138	down
MACS_PEAK_1496	22	7.88	Prdm6	31069	101230070	2.116293669	down
MACS_PEAK_1501	25	7.11	Gramd3	1285174	6650546	3.307068825	down
MACS_PEAK_1502	48	6.66	Gramd3	936850	6650546	3.307068825	down
MACS_PEAK_1503	56	24.74	Gramd3	808360	6650546	3.307068825	down
MACS_PEAK_1513	18	9.69	Fbn2	234552	103830487	1.77337563	up
MACS_PEAK_1514	35	15.75	Fbn2	224237	103830487	1.77337563	up
MACS_PEAK_1516	76	39.99	Fbn2	177156	103830487	1.77337563	up
MACS_PEAK_1518	32	12.75	Ndst1	64899	102060286	1.803198338	up
MACS_PEAK_1519	40	11.55	Synpo	45424	102680377	1.866822839	down
MACS_PEAK_1519	40	11.55	Synpo	45424	104760168	1.684028506	down
MACS_PEAK_1520	53	19.39	Synpo	46377	102680377	1.866822839	down
MACS_PEAK_1520	53	19.39	Synpo	46377	104760168	1.684028506	down
MACS_PEAK_1521	27	8.73	Tcof1	56779	101340575	1.891676545	up
MACS_PEAK_1526	103	43.46	Txnl1	198627	101400593	1.59683907	up
MACS_PEAK_1527	19	6.06	Txnl1	178685	101400593	1.59683907	up
MACS_PEAK_1528	24	10.3	Nedd4l	202619	6380368	1.516248822	down
MACS_PEAK_1529	34	8.87	Nedd4l	117533	6380368	1.516248822	down
MACS_PEAK_1533	39	20.6	Malt1	89686	4670292	1.50060451	up
MACS_PEAK_1534	29	9.69	Malt1	92651	4670292	1.50060451	up
MACS_PEAK_1535	23	9.09	Malt1	99581	4670292	1.50060451	up
MACS_PEAK_1559	35	8.24	Nfatc1	159221	2320348	2.426151514	down
MACS_PEAK_1559	35	8.24	Nfatc1	159221	6180161	1.97121489	down
MACS_PEAK_1559	35	8.24	Nfatc1	159221	510400	3.104269505	down
MACS_PEAK_1559	35	8.24	Nfatc1	159221	6290136	1.625146389	down
MACS_PEAK_1560	42	9.14	Nfatc1	119935	2320348	2.426151514	down
MACS_PEAK_1560	42	9.14	Nfatc1	119935	6180161	1.97121489	down
MACS_PEAK_1560	42	9.14	Nfatc1	119935	510400	3.104269505	down
MACS_PEAK_1560	42	9.14	Nfatc1	119935	6290136	1.625146389	down
MACS_PEAK_1564	29	13.93	Atp9b	223742	3710494	2.144056559	up
MACS_PEAK_1580	44	9.09	Psat1	776138	670446	2.637450457	down
MACS_PEAK_1595	47	21.81	Trpm3	159452	6400731	1.53155458	down
MACS_PEAK_1607	25	11.51	Minpp1	69857	5860736	2.846674681	down
MACS_PEAK_1607	25	11.51	Minpp1	69857	870301	7.255403996	down
MACS_PEAK_1607	25	11.51	Minpp1	69857	6110112	1.528426886	down
MACS_PEAK_1608	68	29.69	Minpp1	116242	5860736	2.846674681	down
MACS_PEAK_1608	68	29.69	Minpp1	116242	870301	7.255403996	down
MACS_PEAK_1608	68	29.69	Minpp1	116242	6110112	1.528426886	down
MACS_PEAK_1609	59	20.32	Minpp1	120712	5860736	2.846674681	down
MACS_PEAK_1609	59	20.32	Minpp1	120712	870301	7.255403996	down
MACS_PEAK_1609	59	20.32	Minpp1	120712	6110112	1.528426886	down
MACS_PEAK_1610	35	11.51	Minpp1	129181	5860736	2.846674681	down
MACS_PEAK_1610	35	11.51	Minpp1	129181	870301	7.255403996	down
MACS_PEAK_1610	35	11.51	Minpp1	129181	6110112	1.528426886	down
MACS_PEAK_1655	65	26.05	Camk1d	335342	101230692	1.549100399	up
MACS_PEAK_1659	31	11.29	Gata3	2763121	6130068	1.555933714	down
MACS_PEAK_1679	48	12.19	Egfl7	47691	670170	1.795167565	up
MACS_PEAK_1680	29	11.25	Egfl7	45257	670170	1.795167565	up
MACS_PEAK_1681	28	10.91	Egfl7	40051	670170	1.795167565	up
MACS_PEAK_1694	20	7.4	St6galnac6	14356	1230315	1.544875264	down
MACS_PEAK_1700	25	12.12	Pbx3	455307	3710577	2.450549364	down
MACS_PEAK_1701	28	13.33	Pbx3	429747	3710577	2.450549364	down
MACS_PEAK_1704	24	9.69	Ggta1	52791	1570292	1.871118188	up
MACS_PEAK_1705	51	19.95	Ggta1	196274	1570292	1.871118188	up
MACS_PEAK_1706	27	9.34	Ggta1	241273	1570292	1.871118188	up
MACS_PEAK_1717	25	7.88	Stam2	118946	2120377	1.636899948	down
MACS_PEAK_1718	19	5.78	Stam2	222475	2120377	1.636899948	down
MACS_PEAK_1730	19	8.48	Rbms1	178150	6400014	1.956834078	down
MACS_PEAK_1742	20	7.88	Sp3	455854	104210341	2.323744059	up
MACS_PEAK_1753	30	13.93	Ube2e3	337903	101980025	2.235800743	up
MACS_PEAK_1760	61	10.57	Tspan18	117664	940435	2.772142649	down
MACS_PEAK_1762	44	19.95	Tspan18	99243	940435	2.772142649	down
MACS_PEAK_1767	42	21.2	Cat	80037	4280152	1.666490912	up
MACS_PEAK_1768	24	11.08	Cat	101456	4280152	1.666490912	up
MACS_PEAK_1787	26	10.91	Spred1	145639	6940706	2.010764599	down
MACS_PEAK_1789	22	10.91	Thbs1	555435	430288	5.464723587	down
MACS_PEAK_1789	22	10.91	Thbs1	555435	4560494	5.315876007	down
MACS_PEAK_1790	20	8.48	Thbs1	553488	430288	5.464723587	down
MACS_PEAK_1790	20	8.48	Thbs1	553488	4560494	5.315876007	down
MACS_PEAK_1791	30	13.93	Thbs1	491918	430288	5.464723587	down
MACS_PEAK_1791	30	13.93	Thbs1	491918	4560494	5.315876007	down
MACS_PEAK_1792	60	28.47	Thbs1	432442	430288	5.464723587	down
MACS_PEAK_1792	60	28.47	Thbs1	432442	4560494	5.315876007	down
MACS_PEAK_1793	24	8.7	Thbs1	387701	430288	5.464723587	down

MACS_PEAK_1793	24	8.7	Thbs1	387701	4560494	5.315876007	down
MACS_PEAK_1809	26	5.46	Anapc1	21991	100610463	1.925601721	up
MACS_PEAK_1810	39	12.19	Anapc1	26452	100610463	1.925601721	up
MACS_PEAK_1813	23	6.66	4930402H24Rik	20826	1190164	1.547660351	up
MACS_PEAK_1829	36	15.75	Tasp1	291472	520403	1.752894998	down
MACS_PEAK_1830	21	8.87	Tasp1	262601	520403	1.752894998	down
MACS_PEAK_1850	23	7.88	Bcl2l1	46037	1580452	1.568951726	down
MACS_PEAK_1850	23	7.88	Bcl2l1	46037	5420484	1.546980739	down
MACS_PEAK_1859	47	19.99	2900097C17Rik	14202	101580368	2.592229843	down
MACS_PEAK_1860	28	14.54	2900097C17Rik	47323	101580368	2.592229843	down
MACS_PEAK_1873	47	16.41	Tomm34	36945	5690102	1.965200901	down
MACS_PEAK_1896	22	9.09	Npepl1	90223	103870315	1.711185455	up
MACS_PEAK_1923	53	23.83	Pde7a	114807	2190041	4.084401608	down
MACS_PEAK_1923	53	23.83	Pde7a	114807	1690746	2.622594118	down
MACS_PEAK_1931	87	27.26	Fndc3b	116411	1050687	2.314380884	down
MACS_PEAK_1932	31	13.93	Fndc3b	102788	1050687	2.314380884	down
MACS_PEAK_1933	33	10.91	Fndc3b	96976	1050687	2.314380884	down
MACS_PEAK_1963	27	11.51	Pcdh10	3371421	3190204	1.756918907	up
MACS_PEAK_1963	27	11.51	Pcdh10	3371421	940014	1.62713182	up
MACS_PEAK_1964	22	8.48	Pcdh10	3312848	3190204	1.756918907	up
MACS_PEAK_1964	22	8.48	Pcdh10	3312848	940014	1.62713182	up
MACS_PEAK_1973	40	15.94	Cog6	496720	3370601	1.77967155	down
MACS_PEAK_1982	23	9.09	Nbea	771894	106620717	1.603463292	down
MACS_PEAK_1990	17	9.69	P2ry1	341034	6040121	2.169948816	down
MACS_PEAK_1993	53	22.42	Dhx36	112530	2470465	1.817358971	down
MACS_PEAK_1994	18	6.06	Dhx36	109212	2470465	1.817358971	down
MACS_PEAK_2003	21	9.69	Ppm1l	180946	102570082	1.582718134	up
MACS_PEAK_2020	26	7.62	Arhgef2	25731	3360577	1.610099435	down
MACS_PEAK_2021	33	15.75	Clk2	12503	510079	2.464841843	down
MACS_PEAK_2027	27	10.79	Anp32e	43881	6510706	2.025931835	down
MACS_PEAK_2035	23	7.88	Sec22b	33079	3190278	1.934768319	down
MACS_PEAK_2036	47	4.88	Sec22b	40722	3190278	1.934768319	down
MACS_PEAK_2039	52	7.21	Hmgcs2	36872	770725	1.805868745	down
MACS_PEAK_2043	23	8.48	Tbx15	412700	4610411	2.563603878	down
MACS_PEAK_2048	17	8.48	Trim33	85008	2230280	3.103994131	down
MACS_PEAK_2048	17	8.48	Trim33	85008	580619	3.768191814	down
MACS_PEAK_2055	71	34.53	Csf1	112949	3120400	1.510946751	up
MACS_PEAK_2089	20	7.88	Lef1	243364	7100288	1.737193108	down
MACS_PEAK_2091	18	8.48	Hadh	136400	3830465	5.792607307	down
MACS_PEAK_2116	21	6.66	Znhit6	72789	6510458	1.624250531	down
MACS_PEAK_2117	39	5.63	Ddah1	108038	6400750	2.672152281	down
MACS_PEAK_2118	47	9.04	Ddah1	54958	6400750	2.672152281	down
MACS_PEAK_2119	27	12.72	Cyr61	129560	5290026	3.337931395	down
MACS_PEAK_2123	28	10.91	Fubp1	17869	5390373	2.243709564	down
MACS_PEAK_2134	43	18.78	Tox	987670	3440053	4.584356308	down
MACS_PEAK_2135	24	7.88	Tox	1105272	3440053	4.584356308	down
MACS_PEAK_2137	23	10.81	Chd7	84998	3870372	1.861780882	down
MACS_PEAK_2178	39	19.72	Zfp618	302113	3290369	1.567986131	up
MACS_PEAK_2179	33	10.3	Zfp618	291283	3290369	1.567986131	up
MACS_PEAK_2180	29	9.14	Col27a1	28892	100130541	2.459280014	down
MACS_PEAK_2180	29	9.14	Col27a1	28892	1740390	4.319157124	down
MACS_PEAK_2189	18	8.37	Ptprd	2179714	3120097	1.835583448	up
MACS_PEAK_2190	23	7.88	Ptprd	3924561	3120097	1.835583448	up
MACS_PEAK_2195	50	11.18	Mpdz	840795	4050577	1.670507431	down
MACS_PEAK_2195	50	11.18	Mpdz	840795	1990064	2.104254484	down
MACS_PEAK_2200	48	21.2	Psip1	345310	5050594	5.23514986	down
MACS_PEAK_2215	26	11.08	Dmrta1	218065	3190333	2.103105068	down
MACS_PEAK_2222	97	31.04	Hook1	219600	2480500	2.538444281	down
MACS_PEAK_2235	18	6.66	Ror1	259929	6220026	1.958731174	down
MACS_PEAK_2273	49	19.72	Trim62	43768	4760451	2.022188902	up
MACS_PEAK_2284	20	6.66	Clic4	125866	3290368	3.674860239	down
MACS_PEAK_2284	20	6.66	Clic4	125866	6450181	4.74880743	down
MACS_PEAK_2285	28	13.75	Clic4	106057	3290368	3.674860239	down
MACS_PEAK_2285	28	13.75	Clic4	106057	6450181	4.74880743	down
MACS_PEAK_2286	30	8.96	Srrm1	54840	6110025	1.615817308	up
MACS_PEAK_2287	32	11.84	A330049M08Rik	47923	430112	2.046452045	down
MACS_PEAK_2305	50	22.72	Ube4b	18519	3610154	1.58241725	up
MACS_PEAK_2313	18	7.11	Errfi1	35068	3450273	1.543256164	down
MACS_PEAK_2317	29	9.65	Gpr153	47085	4120332	1.725471854	up
MACS_PEAK_2347	15	7.27	Srpk2	55834	6380341	2.011079073	down
MACS_PEAK_2361	21	7.76	Hs3st1	1443193	1850193	1.553391099	down
MACS_PEAK_2362	28	10.65	Ldb2	450535	5670441	2.367152691	down
MACS_PEAK_2368	21	9.09	Gpr125	981447	6380025	2.239007235	down
MACS_PEAK_2369	25	10.3	Gpr125	1353556	6380025	2.239007235	down
MACS_PEAK_2370	34	13.85	Tbc1d19	226075	4050170	1.680420637	up
MACS_PEAK_2388	34	7.03	Sgcb	29088	6370711	1.52116406	down
MACS_PEAK_2396	40	17.57	Fip1l1	62236	6510333	1.568021417	down
MACS_PEAK_2397	32	15.15	Fip1l1	23468	6510333	1.568021417	down
MACS_PEAK_2398	28	9.09	Chic2	202833	101980039	1.743302584	down
MACS_PEAK_2412	15	8.48	Art3	39733	2760348	1.571328759	down
MACS_PEAK_2418	29	12.12	Nkx6-1	428063	6040731	2.532574654	up
MACS_PEAK_2421	27	12.12	Nudt9	25400	2480070	1.657863975	down
MACS_PEAK_2425	40	16.07	Lrrc8d	113825	1780014	1.506517887	down
MACS_PEAK_2443	29	8.27	Ung	41975	6100364	2.298276901	down
MACS_PEAK_2447	33	11.38	Hspb8	148252	540563	1.629815936	up
MACS_PEAK_2468	27	9.09	Gbas	37063	6760278	1.902266145	down
MACS_PEAK_2469	18	6.66	Tyw1	366444	106650348	2.425904274	up
MACS_PEAK_2496	17	8.48	Uncx	110842	2060142	1.693639398	up
MACS_PEAK_2509	19	10.91	Hmgb1	95754	102640685	2.085407734	down
MACS_PEAK_2511	18	7.88	Alox5ap	79352	3170440	2.468556643	up
MACS_PEAK_2520	18	6.66	Peg10	69939	104210161	1.547018647	down
MACS_PEAK_2521	49	17.18	Peg10	212107	104210161	1.547018647	down
MACS_PEAK_2527	35	12.72	Tac1	470491	7000195	1.699343801	down
MACS_PEAK_2531	34	19.99	Glcci1	185717	3290070	2.098738909	down
MACS_PEAK_2557	33	11.64	Plxna4	326715	102450484	4.854547024	up
MACS_PEAK_2561	31	8.94	Cald1	165741	104730707	1.581065774	down
MACS_PEAK_2561	31	8.94	Cald1	165741	1770129	1.575563431	down
MACS_PEAK_2566	33	10.79	Ptn	253256	5910161	1.975698113	down
MACS_PEAK_2567	35	16.96	Ptn	286563	5910161	1.975698113	down
MACS_PEAK_2574	160	3.58	Tbxas1	36076	5050053	6.862919331	up
MACS_PEAK_2574	160	3.58	Tbxas1	36076	6900286	1.623475432	up
MACS_PEAK_2575	92	14.63	Ezh2	54628	6130605	3.118177891	down
MACS_PEAK_2588	119	22.99	Fkbp14	38579	6510601	2.260330915	down
MACS_PEAK_2599	74	12.07	Tgoln1	105132	4920433	2.579447985	down
MACS_PEAK_2599	74	12.07	Tgoln1	105132	3060270	1.883389592	down
MACS_PEAK_2610	22	9.95	Npm3-ps1	200049	3610300	2.108114243	down
MACS_PEAK_2613	28	10.6	Nfu1	31269	5700047	1.652709723	up
MACS_PEAK_2633	46	11.81	Pdzrn3	401962	2340131	1.666963577	up
MACS_PEAK_2634	29	12.12	Pdzrn3	400305	2340131	1.666963577	up
MACS_PEAK_2641	72	29.97	Sumf1	78510	730050	1.816872358	up
MACS_PEAK_2657	20	7.88	Wnk1	34654	3170603	1.862841845	down
MACS_PEAK_2662	37	14.41	Cd9	61445	4730041	2.94108367	down
MACS_PEAK_2663	18	6.66	Vwf	85448	4670519	3.012349367	up
MACS_PEAK_2664	51	9.38	Vwf	84635	4670519	3.012349367	up
MACS_PEAK_2677	21	7.27	Eps8	215135	7050204	2.723695993	down
MACS_PEAK_2678	15	6.66	Eps8	177724	7050204	2.723695993	down
MACS_PEAK_2682	35	14.54	Mgst1	36155	6020605	2.017282248	down
MACS_PEAK_2684	39	18.78	Aebp2	509949	106550154	1.500701666	down
MACS_PEAK_2688	67	15.15	Sox5	830709	3190128	3.311142206	down
MACS_PEAK_2688	67	15.15	Sox5	830709	2370576	4.999372005	down
MACS_PEAK_2688	67	15.15	Sox5	830709	2900167	8.529214859	down
MACS_PEAK_2689	65	26.05	Sox5	709819	3190128	3.311142206	down

MACS_PEAK_2689	65	26.05	Sox5	709819	2370576	4.999372005	down
MACS_PEAK_2689	65	26.05	Sox5	709819	2900167	8.529214859	down
MACS_PEAK_2690	45	10.3	Sox5	678769	3190128	3.311142206	down
MACS_PEAK_2690	45	10.3	Sox5	678769	2370576	4.999372005	down
MACS_PEAK_2690	45	10.3	Sox5	678769	2900167	8.529214859	down
MACS_PEAK_2691	77	38.77	Sox5	650875	3190128	3.311142206	down
MACS_PEAK_2691	77	38.77	Sox5	650875	2370576	4.999372005	down
MACS_PEAK_2691	77	38.77	Sox5	650875	2900167	8.529214859	down
MACS_PEAK_2702	24	9.69	Sox5	710245	3190128	3.311142206	down
MACS_PEAK_2702	24	9.69	Sox5	710245	2370576	4.999372005	down
MACS_PEAK_2702	24	9.69	Sox5	710245	2900167	8.529214859	down
MACS_PEAK_2703	148	59.09	Sox5	729447	3190128	3.311142206	down
MACS_PEAK_2703	148	59.09	Sox5	729447	2370576	4.999372005	down
MACS_PEAK_2703	148	59.09	Sox5	729447	2900167	8.529214859	down
MACS_PEAK_2704	32	14.54	Sox5	735585	3190128	3.311142206	down
MACS_PEAK_2704	32	14.54	Sox5	735585	2370576	4.999372005	down
MACS_PEAK_2704	32	14.54	Sox5	735585	2900167	8.529214859	down
MACS_PEAK_2705	26	7.5	Sox5	752240	3190128	3.311142206	down
MACS_PEAK_2705	26	7.5	Sox5	752240	2370576	4.999372005	down
MACS_PEAK_2705	26	7.5	Sox5	752240	2900167	8.529214859	down
MACS_PEAK_2709	25	9.09	4933424B01Rik	177710	4920239	4.310250759	down
MACS_PEAK_2712	21	6.66	Pthlh	163096	5290739	1.747664928	down
MACS_PEAK_2724	31	12.12	Snrpa	14291	2570100	8.081143379	down
MACS_PEAK_2724	31	12.12	Snrpa	14291	4150121	8.390077591	down
MACS_PEAK_2734	66	36.35	C80913	329164	2360458	1.696633697	down
MACS_PEAK_2761	29	7.84	Chd2	149558	102120066	1.902526021	down
MACS_PEAK_2764	36	13.73	St8sia2	299911	2680082	1.841214657	up
MACS_PEAK_2765	32	9.19	St8sia2	332916	2680082	1.841214657	up
MACS_PEAK_2766	19	6.06	Akap13	158613	101450594	1.573993444	up
MACS_PEAK_2787	22	9.69	Picalm	70061	1940280	1.597013712	up
MACS_PEAK_2788	39	18.78	Dlg2	1460362	107050452	1.835449576	up
MACS_PEAK_2798	36	9.35	Spcs2	35455	360301	1.929940462	up
MACS_PEAK_2803	22	8.48	P2ry6	40582	5290400	6.581582546	up
MACS_PEAK_2804	16	7.62	Stard10	28737	4570170	1.654127836	down
MACS_PEAK_2812	21	9.69	Usp47	288128	105900047	1.575356126	down
MACS_PEAK_2818	108	46.04	Sox6	1003626	6840717	3.285536766	down
MACS_PEAK_2818	108	46.04	Sox6	1003626	4070601	1.792667627	down
MACS_PEAK_2839	39	4.74	Lat	7885	3170025	1.69903791	up
MACS_PEAK_2893	58	15.75	Plekha2	92937	2850091	2.480962276	up
MACS_PEAK_2899	29	11.51	6430573F11Rik	205930	7000347	1.823604465	down
MACS_PEAK_2900	45	14.81	6430573F11Rik	65894	7000347	1.823604465	down
MACS_PEAK_2901	35	16.96	6430573F11Rik	92051	7000347	1.823604465	down
MACS_PEAK_2902	25	13.33	A730069N07Rik	34933	100360010	2.787984133	up
MACS_PEAK_2905	17	6.06	Fgl1	41584	2350358	1.56188643	up
MACS_PEAK_2937	23	7.88	Ints10	87944	2120609	1.54038012	up
MACS_PEAK_2938	21	6.65	Ints10	66171	2120609	1.54038012	up
MACS_PEAK_2946	47	21.81	Ednra	277426	106550204	2.295722961	up
MACS_PEAK_2947	29	11.25	Slc10a7	233269	7320438	1.822438002	down
MACS_PEAK_2959	23	10.91	Nfix	45787	2450152	1.575346112	down
MACS_PEAK_2960	150	76.05	Lyl1	36952	2900242	2.016277552	up
MACS_PEAK_2961	26	7.5	Lyl1	42764	2900242	2.016277552	up
MACS_PEAK_2989	43	13.33	Nob1	33262	5700184	1.675647736	down
MACS_PEAK_2990	52	12.75	Nob1	40530	5700184	1.675647736	down
MACS_PEAK_2991	70	25.45	Nob1	44121	5700184	1.675647736	down
MACS_PEAK_2992	27	9.69	Nob1	47921	5700184	1.675647736	down
MACS_PEAK_2993	37	12.19	Nob1	53996	5700184	1.675647736	down
MACS_PEAK_2999	65	30.9	Zfhx3	686846	7040465	1.900578856	up
MACS_PEAK_3004	27	12.72	Adamts18	150236	104070017	1.659858108	down
MACS_PEAK_3010	22	6.6	Irf8	327440	610161	3.621077776	up
MACS_PEAK_3042	19	8.48	Fat3	1494738	105670446	2.421111107	up
MACS_PEAK_3048	33	12.19	Bbs9	586852	4540368	1.631353974	down
MACS_PEAK_3049	23	6.66	Bbs9	758713	4540368	1.631353974	down
MACS_PEAK_3060	68	36.35	Kirrel3	1627971	2630195	1.939929843	up
MACS_PEAK_3063	41	13.33	Cdon	120742	104810102	1.829356194	down
MACS_PEAK_3066	19	6.66	Cdon	56293	104810102	1.829356194	down
MACS_PEAK_3085	26	7.76	Ncam1	351304	105690114	1.675677061	up
MACS_PEAK_3090	64	8.94	Scamp5	47594	6290021	1.893077731	up
MACS_PEAK_3096	23	8.42	Adpgk	133183	4200164	1.868386626	up
MACS_PEAK_3109	20	10.3	Tle3	517662	105270110	1.855359912	up
MACS_PEAK_3110	32	12.72	Tle3	531334	105270110	1.855359912	up
MACS_PEAK_3121	23	7.88	Snx1	46185	3190670	1.925142765	up
MACS_PEAK_3135	25	11.51	Gnb5	67394	6550722	2.050322056	down
MACS_PEAK_3145	31	13.33	Cd109	843966	130014	1.82360971	down
MACS_PEAK_3145	31	13.33	Cd109	843966	540152	1.708335042	down
MACS_PEAK_3151	26	10.91	Tmed3	84178	6940605	2.500232935	down
MACS_PEAK_3153	37	16.36	Tbc1d2b	760316	107100039	1.949914932	up
MACS_PEAK_3159	123	55.74	Spsb4	94605	4070673	1.836065888	down
MACS_PEAK_3160	23	10.3	Acpl2	72895	1690736	1.52359724	down
MACS_PEAK_3160	23	10.3	Acpl2	72895	6200364	2.482869387	down
MACS_PEAK_3173	88	44.83	Rpl29	44979	1240075	2.101823807	down
MACS_PEAK_3175	33	13.6	Gnai2	22568	102470047	1.68414557	up
MACS_PEAK_3176	46	11.08	Rbm6	101634	6020446	2.289750576	down
MACS_PEAK_3176	46	11.08	Rbm6	101634	3840563	2.153254986	down
MACS_PEAK_3176	46	11.08	Rbm6	101634	4780129	3.783868313	down
MACS_PEAK_3181	24	6.58	Scap	51433	3800706	1.572818518	up
MACS_PEAK_3189	38	21.81	Tgfbr2	632533	6550398	2.211287975	down
MACS_PEAK_3190	22	8.48	Tgfbr2	889025	6550398	2.211287975	down
MACS_PEAK_3223	53	12.19	Usp9x	171677	3120338	1.864694238	down
MACS_PEAK_3235	41	16.36	Fam122b	103058	770050	1.527989864	down
MACS_PEAK_3241	29	11.51	Yipf6	82610	1850338	1.743624806	down
MACS_PEAK_3243	23	10.3	Cox7b	60310	2340504	1.761149645	up
MACS_PEAK_3253	18	7.88	A830080D01Rik	294385	101780402	1.836452127	down
MACS_PEAK_3254	21	12.72	A830080D01Rik	307784	101780402	1.836452127	down
MACS_PEAK_1	22	9.09	Pcmtd1	30601	1450603	1.791262388	down
MACS_PEAK_5	53	23.63	Eya1	101809	101580487	1.914695263	down
MACS_PEAK_5	53	23.63	Eya1	101809	1450278	3.289761305	down
MACS_PEAK_5	53	23.63	Eya1	101809	5220390	4.272898674	down
MACS_PEAK_13	54	18.78	Col9a1	18482	105860463	4.804460526	down
MACS_PEAK_14	32	11.51	Col9a1	44317	105860463	4.804460526	down
MACS_PEAK_15	28	14.54	Col9a1	53031	105860463	4.804460526	down
MACS_PEAK_33	24	12.12	1500015O10Rik	114444	3130368	1.779098392	down
MACS_PEAK_34	20	10.3	1500015O10Rik	15528	3130368	1.779098392	down
MACS_PEAK_35	43	13.21	Gulp1	33615	5570446	5.826993942	down
MACS_PEAK_36	90	48.47	Gulp1	169599	5570446	5.826993942	down
MACS_PEAK_38	18	5.45	Col5a2	389	104670129	2.087709427	up
MACS_PEAK_41	23	9.09	Myo1b	93906	101410168	1.891579628	down
MACS_PEAK_41	23	9.09	Myo1b	93906	105910021	2.122879982	down
MACS_PEAK_62	19	9.05	Nrp2	390588	6650446	1.683444738	down
MACS_PEAK_63	25	10.3	Nrp2	14830	6650446	1.683444738	down
MACS_PEAK_82	44	16.96	Slc4a3	35828	360603	1.932770133	up
MACS_PEAK_86	38	9.58	Epha4	109908	460750	1.665304303	up
MACS_PEAK_94	21	6.66	Pde6d	360	3390014	2.121124744	down
MACS_PEAK_96	21	8.48	3110079O15Rik	27822	7100292	4.763119698	down
MACS_PEAK_118	26	6.65	Gli2	27359	3060632	1.640829682	down
MACS_PEAK_144	20	7.27	Prg4	68771	5860156	1.597024322	down
MACS_PEAK_145	20	9.69	Hmcn1	43257	104570670	3.694500208	down
MACS_PEAK_152	38	11.18	Ncf2	14870	2650133	7.074065685	up
MACS_PEAK_158	18	9.51	Fam20b	22290	2030215	1.650509834	down
MACS_PEAK_159	22	9.42	Angptl1	30500	104920372	2.244338512	down
MACS_PEAK_160	28	9.14	Angptl1	252	104920372	2.244338512	down
MACS_PEAK_163	32	14.54	Prdx6	22461	6380601	1.883167624	up
MACS_PEAK_170	79	12.03	Dnm3os	90636	102940671	2.063773394	down

MACS_PEAK_171	28	5.45	Dnm3os	29737	102940671	2.063773394	down
MACS_PEAK_172	24	7.88	Dnm3os	46	102940671	2.063773394	down
MACS_PEAK_188	53	16.71	Zfp238	1464	5050537	2.892208099	down
MACS_PEAK_190	18	7.88	Kif26b	204050	1940288	1.922086239	down
MACS_PEAK_191	99	34.4	Kif26b	292121	1940288	1.922086239	down
MACS_PEAK_192	20	7.88	Kif26b	490685	1940288	1.922086239	down
MACS_PEAK_205	18	7.88	Lyplal1	266598	5290717	2.084866047	down
MACS_PEAK_211	25	7.82	Smyd2	72520	5050026	2.524350405	down
MACS_PEAK_222	30	14.54	Utrn	307400	3390048	3.522297144	down
MACS_PEAK_247	45	10.89	Rev3l	59317	1090717	1.678638935	down
MACS_PEAK_248	24	7.88	Gtf3c6	890	360546	1.66050005	up
MACS_PEAK_278	25	6.66	Slc35f1	13814	3850164	2.190327406	down
MACS_PEAK_282	49	21.2	Ccdc109a	50375	106520647	4.056745529	down
MACS_PEAK_334	19	6.1	Dcn	165735	510332	3.202011585	down
MACS_PEAK_335	20	7.27	Lum	1022	5420079	2.972158909	down
MACS_PEAK_337	17	5.45	Atp2b1	210481	104150181	1.961512804	down
MACS_PEAK_338	17	7.88	Dusp6	19445	7100070	1.744878888	down
MACS_PEAK_339	19	9.09	Dusp6	2323	7100070	1.744878888	down
MACS_PEAK_344	20	7.27	Phlda1	10888	2450020	2.164865017	down
MACS_PEAK_345	37	14.07	Phlda1	86708	2450020	2.164865017	down
MACS_PEAK_348	15	7.27	Zfc3h1	33686	4070706	1.60190773	down
MACS_PEAK_349	40	19.99	Lgr5	68756	6020400	2.284122944	down
MACS_PEAK_350	36	12.72	Tspan8	5314	1740538	1.740787864	down
MACS_PEAK_355	33	4.62	Cpm	42508	103440025	1.511164546	up
MACS_PEAK_357	38	19.39	Slc35e3	4700	6760707	2.735560656	down
MACS_PEAK_358	39	15.75	Slc35e3	2101	6760707	2.735560656	down
MACS_PEAK_402	25	9.14	Bcl11a	227426	106380398	1.969925761	up
MACS_PEAK_406	18	7.27	Spnb2	1363	380091	2.274885654	down
MACS_PEAK_411	29	12.72	Slit3	82904	106650441	2.003383636	up
MACS_PEAK_413	27	10.61	Rars	58734	380390	1.694197536	down
MACS_PEAK_425	25	9.09	Gfpt2	29344	102810242	1.50763905	down
MACS_PEAK_426	17	5.45	Gfpt2	881	102810242	1.50763905	down
MACS_PEAK_429	22	5.59	Col23a1	116729	2030358	1.54298389	up
MACS_PEAK_432	34	13.31	Hspa4	194743	1050170	1.625148892	up
MACS_PEAK_436	31	16.36	Fam114a2	84293	3610685	1.651664257	up
MACS_PEAK_449	63	29.08	2810001G20Rik	131680	102320292	1.537947655	down
MACS_PEAK_450	33	12.12	Hs3st3a1	113112	2230497	1.850928664	down
MACS_PEAK_452	22	7.27	Elac2	145069	3440167	1.903346419	down
MACS_PEAK_453	27	8.31	Elac2	79005	3440167	1.903346419	down
MACS_PEAK_459	28	13.33	Efnb3	4366	5570594	1.549831986	up
MACS_PEAK_479	107	59.37	Tbx4	20426	106370097	1.557803273	up
MACS_PEAK_499	49	26.05	Tom1l1	294546	3190070	1.684084654	down
MACS_PEAK_501	47	18.29	Mycbpap	5078	4010022	2.147407293	up
MACS_PEAK_514	43	12.72	Adam11	12677	104560019	1.94789505	up
MACS_PEAK_517	15	7.88	Tex2	27979	3060494	1.597836733	up
MACS_PEAK_519	27	8.13	Pitpnc1	111853	3990017	2.315482378	down
MACS_PEAK_520	18	9.09	Pitpnc1	90266	3990017	2.315482378	down
MACS_PEAK_521	91	28.14	Pitpnc1	57227	3990017	2.315482378	down
MACS_PEAK_547	47	14.81	Sphk1	3131	2470113	2.671342373	down
MACS_PEAK_548	17	7.27	Sphk1	932	2470113	2.671342373	down
MACS_PEAK_563	21	9.09	Aspscr1	10156	3990739	1.595929027	down
MACS_PEAK_567	47	22.42	Hs1bp3	73080	103940735	1.757426143	down
MACS_PEAK_567	47	22.42	Hs1bp3	73080	380524	2.154220581	down
MACS_PEAK_571	26	6.06	Osr1	235195	1500025	1.59414053	up
MACS_PEAK_571	26	6.06	Osr1	235195	5270706	2.1889112	up
MACS_PEAK_578	23	9.09	Trib2	177440	4120605	1.596457601	up
MACS_PEAK_579	49	22.42	Trib2	85307	4120605	1.596457601	up
MACS_PEAK_580	27	11.51	Trib2	121219	4120605	1.596457601	up
MACS_PEAK_581	21	9.09	1700030C10Rik	618556	103610020	2.162940025	up
MACS_PEAK_594	43	15.94	Pxdn	96797	106760300	3.044472218	down
MACS_PEAK_596	42	19.39	Prkar2b	28128	3130593	2.270573378	down
MACS_PEAK_596	42	19.39	Prkar2b	28128	5220577	1.900593281	down
MACS_PEAK_607	29	7.27	Etv1	60164	70735	4.297760963	down
MACS_PEAK_607	29	7.27	Etv1	60164	5080463	3.938217402	down
MACS_PEAK_608	18	7.27	Etv1	145389	70735	4.297760963	down
MACS_PEAK_608	18	7.27	Etv1	145389	5080463	3.938217402	down
MACS_PEAK_618	65	8.07	Eapp	14565	4070575	2.126230717	up
MACS_PEAK_626	41	13.33	1110034A24Rik	12223	6940280	1.730407715	down
MACS_PEAK_630	23	9.89	Frmd6	73149	4670019	1.692186475	up
MACS_PEAK_632	15	8.45	Actr10	29105	5860458	3.908699751	down
MACS_PEAK_646	20	9.69	Syne2	44048	101980154	1.566098332	up
MACS_PEAK_667	23	11.53	Isca2	32474	3990450	2.766831875	up
MACS_PEAK_669	17	8.48	Jdp2	54199	2360500	1.564003229	down
MACS_PEAK_696	15	7.88	Evl	8417	1740113	1.865595222	up
MACS_PEAK_715	19	8.48	Zmynd11	46828	630181	1.85174334	down
MACS_PEAK_715	19	8.48	Zmynd11	46828	2570019	3.239306688	down
MACS_PEAK_716	233	8.38	Zmynd11	69027	630181	1.85174334	down
MACS_PEAK_716	233	8.38	Zmynd11	69027	2570019	3.239306688	down
MACS_PEAK_717	22	10.3	Gli3	351493	5690148	3.173316717	down
MACS_PEAK_718	22	8.87	Gli3	165009	5690148	3.173316717	down
MACS_PEAK_719	25	10.91	Gli3	96366	5690148	3.173316717	down
MACS_PEAK_721	23	9.09	Rala	30987	2680471	1.733900785	down
MACS_PEAK_732	27	7.11	Hist1h2be	427	3170168	1.672124267	down
MACS_PEAK_741	45	18.29	Sox4	321263	2260091	2.331992149	up
MACS_PEAK_742	65	26.66	Sox4	262922	2260091	2.331992149	up
MACS_PEAK_743	21	7.76	Sox4	114675	2260091	2.331992149	up
MACS_PEAK_744	33	10.89	Sox4	108906	2260091	2.331992149	up
MACS_PEAK_760	101	36.58	Gmds	158525	1050519	1.810869336	down
MACS_PEAK_761	17	7.88	Gmds	77889	1050519	1.810869336	down
MACS_PEAK_762	18	9.09	Gmds	42173	1050519	1.810869336	down
MACS_PEAK_763	23	6.66	Gmds	88421	1050519	1.810869336	down
MACS_PEAK_764	56	29.08	Gmds	154327	1050519	1.810869336	down
MACS_PEAK_772	18	6.66	Cdyl	158169	4730397	2.133082867	down
MACS_PEAK_792	27	11.68	Mylip	150484	50717	1.537615657	up
MACS_PEAK_798	44	7.58	C030044B11Rik	48295	105890162	1.757001281	down
MACS_PEAK_842	18	6.66	Mef2c	360537	780338	1.61142087	down
MACS_PEAK_843	33	15.75	Mef2c	291254	780338	1.61142087	down
MACS_PEAK_844	22	8.48	Mef2c	62045	780338	1.61142087	down
MACS_PEAK_845	32	10.3	Mef2c	100355	780338	1.61142087	down
MACS_PEAK_846	18	6.66	Mef2c	101322	780338	1.61142087	down
MACS_PEAK_892	23	9.42	Erbb2ip	36864	580253	2.336574078	down
MACS_PEAK_917	24	11.51	Fam107a	4533	6180088	2.543306351	down
MACS_PEAK_951	46	12.95	Gdf10	99068	4850082	2.198053122	down
MACS_PEAK_967	28	7.27	Prmt5	235	4590072	4.670490742	down
MACS_PEAK_976	37	18.78	Il17d	15048	5900377	1.621251225	down
MACS_PEAK_977	36	14.8	Lats2	32384	6020494	1.704494596	down
MACS_PEAK_978	28	9.98	Cdadc1	37841	4120064	1.581121564	down
MACS_PEAK_994	68	32.27	Fndc3a	679522	2690097	3.297939777	down
MACS_PEAK_996	30	11.72	Lrch1	95849	103120008	1.604456425	down
MACS_PEAK_997	60	28.26	Lrch1	48069	103120008	1.604456425	down
MACS_PEAK_1008	25	7.67	9030625A04Rik	160934	2350441	2.277412415	down
MACS_PEAK_1009	20	7.82	9030625A04Rik	57052	2350441	2.277412415	down
MACS_PEAK_1012	18	8.48	1190002H23Rik	8347	4480128	2.61289382	down
MACS_PEAK_1023	18	8.48	Dach1	96132	2450593	1.550462127	down
MACS_PEAK_1026	16	8.48	Klf12	211742	1660095	1.634319186	up
MACS_PEAK_1057	47	26.66	Pcca	65652	3390400	2.243657351	down
MACS_PEAK_1058	45	20.6	Pcca	180695	3390400	2.243657351	down
MACS_PEAK_1061	28	12.72	Dab2	19473	103130670	1.610951066	up
MACS_PEAK_1062	21	9.09	Dab2	16143	103130670	1.610951066	up
MACS_PEAK_1078	22	7.88	Trio	102086	102320605	1.6330235	down
MACS_PEAK_1078	22	7.88	Trio	102086	103360497	1.626712561	down
MACS_PEAK_1091	26	7.2	Cthrc1	1721	103850632	7.363954544	down

MACS_PEAK_1092	33	8.53	Lrp12	171230	2370112	2.219601631	down
MACS_PEAK_1093	22	8.48	Zfpm2	75280	460072	1.900486469	down
MACS_PEAK_1109	33	13.93	Has2	47674	5360181	2.12053895	down
MACS_PEAK_1114	35	14.54	Nsmce2	14308	1770168	4.732778072	down
MACS_PEAK_1136	26	6.65	Myh9	38138	4850292	1.610285163	up
MACS_PEAK_1150	18	8.48	Kif21a	21326	2970338	2.268258572	down
MACS_PEAK_1152	31	12.72	Cntn1	28201	3800204	1.655365348	down
MACS_PEAK_1153	28	12.72	Cntn1	9086	3800204	1.655365348	down
MACS_PEAK_1156	26	10.53	Prickle1	77138	103190427	3.681575537	down
MACS_PEAK_1157	21	7.27	Prickle1	58495	103190427	3.681575537	down
MACS_PEAK_1158	26	11.51	Prickle1	178568	103190427	3.681575537	down
MACS_PEAK_1159	28	13.93	Prickle1	197204	103190427	3.681575537	down
MACS_PEAK_1165	40	15.52	E330033B04Rik	4506	101780592	1.597389221	up
MACS_PEAK_1172	34	16.96	Prpf40b	18928	5390706	1.827224493	up
MACS_PEAK_1173	48	21.2	Tmbim6	27441	1770538	1.648805022	up
MACS_PEAK_1176	90	36.35	9430023L20Rik	6717	5670092	1.620031238	up
MACS_PEAK_1181	20	7.27	Aaas	5391	6400528	2.296341658	down
MACS_PEAK_1191	17	9.69	Glis2	4294	4810170	1.506240129	down
MACS_PEAK_1191	17	9.69	Glis2	4294	4150471	1.845925927	down
MACS_PEAK_1192	52	22.42	Glis2	10949	4810170	1.506240129	down
MACS_PEAK_1192	52	22.42	Glis2	10949	4150471	1.845925927	down
MACS_PEAK_1206	28	11.51	Fgd4	138	6550338	1.711675644	down
MACS_PEAK_1214	47	18.29	Etv5	66593	110017	1.707441449	down
MACS_PEAK_1215	27	11.51	Etv5	10593	110017	1.707441449	down
MACS_PEAK_1216	17	6.66	Etv5	69777	110017	1.707441449	down
MACS_PEAK_1220	32	15.75	Il1rap	80907	3940301	1.749590874	down
MACS_PEAK_1225	20	9.69	Bdh1	1128	2850390	2.179454565	down
MACS_PEAK_1230	52	22.72	Umps	84424	4730438	7.797474861	down
MACS_PEAK_1231	21	7.88	Umps	158868	4730438	7.797474861	down
MACS_PEAK_1235	25	11.51	Mylk	35871	7000364	1.881766915	up
MACS_PEAK_1241	23	12.3	Igsf11	142191	60471	1.629356027	down
MACS_PEAK_1263	108	50.29	Nrip1	1008	3190039	1.901708245	up
MACS_PEAK_1265	31	12.12	Cxadr	247955	106550400	2.025967121	down
MACS_PEAK_1266	24	11.51	Cxadr	243997	106550400	2.025967121	down
MACS_PEAK_1276	18	7.27	Olig1	26819	1660594	2.554029942	down
MACS_PEAK_1281	48	17.5	Runx1	66256	3840711	2.467631817	down
MACS_PEAK_1282	51	24.14	Runx1	43375	3840711	2.467631817	down
MACS_PEAK_1283	37	13.93	Runx1	41891	3840711	2.467631817	down
MACS_PEAK_1284	48	14.54	Runx1	217109	3840711	2.467631817	down
MACS_PEAK_1287	36	13.3	Hlcs	139480	2060537	1.634361148	up
MACS_PEAK_1289	24	7.62	Dyrk1a	134971	102940021	1.69041121	up
MACS_PEAK_1296	175	60.34	Ets2	1578	360451	1.974570274	down
MACS_PEAK_1325	24	9.69	Rgmb	17713	4570450	1.638943791	down
MACS_PEAK_1326	24	10.3	Rgmb	4471	4570450	1.638943791	down
MACS_PEAK_1327	22	7.11	Rgmb	167458	4570450	1.638943791	down
MACS_PEAK_1355	31	7.03	Slc29a1	7837	106860154	1.97650373	down
MACS_PEAK_1381	22	8.78	Ptprm	220539	6370136	1.623142838	down
MACS_PEAK_1382	24	8.44	Ptprm	39807	6370136	1.623142838	down
MACS_PEAK_1383	46	21.08	Arhgap28	96248	5890064	2.059422255	down
MACS_PEAK_1384	19	9.69	Arhgap28	29166	5890064	2.059422255	down
MACS_PEAK_1385	23	10.79	Arhgap28	107973	5890064	2.059422255	down
MACS_PEAK_1406	34	7.28	Map4k3	72892	5390551	1.625558496	down
MACS_PEAK_1449	21	6.66	Zfp521	119485	540541	2.007312298	down
MACS_PEAK_1450	26	7.84	Zfp521	29726	540541	2.007312298	down
MACS_PEAK_1451	52	24.23	Zfp521	18395	540541	2.007312298	down
MACS_PEAK_1456	25	9.66	Zfp191	157	3830750	2.150001049	down
MACS_PEAK_1477	29	8.71	Sema6a	199719	3060021	1.69858098	down
MACS_PEAK_1478	40	9.05	Sema6a	191881	3060021	1.69858098	down
MACS_PEAK_1479	17	6.66	Sema6a	112859	3060021	1.69858098	down
MACS_PEAK_1480	19	7.27	Sema6a	60267	3060021	1.69858098	down
MACS_PEAK_1493	20	7.88	Snx2	88618	2470068	5.240269661	down
MACS_PEAK_1494	71	18.7	Snx2	78797	2470068	5.240269661	down
MACS_PEAK_1495	20	7.2	Ppic	69735	4570110	2.057356834	down
MACS_PEAK_1496	22	7.88	Ppic	15470	4570110	2.057356834	down
MACS_PEAK_1507	21	8.48	Lmnb1	22886	6020008	6.803228855	down
MACS_PEAK_1507	21	8.48	Lmnb1	22886	5890292	3.909193039	down
MACS_PEAK_1508	21	6.66	Lmnb1	26622	6020008	6.803228855	down
MACS_PEAK_1508	21	6.66	Lmnb1	26622	5890292	3.909193039	down
MACS_PEAK_1509	56	29.69	Lmnb1	106432	6020008	6.803228855	down
MACS_PEAK_1509	56	29.69	Lmnb1	106432	5890292	3.909193039	down
MACS_PEAK_1538	19	6.17	Gnal	446	5080364	2.386161327	down
MACS_PEAK_1567	18	6.66	Mbp	4738	5220369	1.633033872	down
MACS_PEAK_1584	29	12.72	Cep78	65816	103830086	1.997898817	down
MACS_PEAK_1585	51	26.66	Gcnt1	113609	940129	1.885344744	down
MACS_PEAK_1585	51	26.66	Gcnt1	113609	1780215	2.185174465	down
MACS_PEAK_1586	39	17.78	Gcnt1	68101	940129	1.885344744	down
MACS_PEAK_1586	39	17.78	Gcnt1	68101	1780215	2.185174465	down
MACS_PEAK_1593	35	11.08	Tmem2	3870	3290020	1.734024167	down
MACS_PEAK_1598	60	18.18	Kank1	91845	3450546	5.633127213	down
MACS_PEAK_1603	28	11.18	Rcl1	24507	2680008	3.034357309	down
MACS_PEAK_1606	33	11.47	Sgms1	21773	4780195	1.556291699	down
MACS_PEAK_1607	25	11.51	Papss2	40089	107000132	3.08749795	down
MACS_PEAK_1607	25	11.51	Papss2	40089	870113	6.537036419	down
MACS_PEAK_1608	68	29.69	Papss2	6296	107000132	3.08749795	down
MACS_PEAK_1608	68	29.69	Papss2	6296	870113	6.537036419	down
MACS_PEAK_1609	59	20.32	Papss2	10766	107000132	3.08749795	down
MACS_PEAK_1609	59	20.32	Papss2	10766	870113	6.537036419	down
MACS_PEAK_1610	35	11.51	Papss2	5055	107000132	3.08749795	down
MACS_PEAK_1610	35	11.51	Papss2	5055	870113	6.537036419	down
MACS_PEAK_1622	21	6.66	Nt5c2	9231	4280735	4.751771927	down
MACS_PEAK_1633	27	12.75	Adra2a	266164	104780167	2.713639736	up
MACS_PEAK_1646	27	10.3	Slc18a2	20214	6420541	1.563637495	down
MACS_PEAK_1647	21	6.06	Pdzd8	20778	104060162	2.089199781	down
MACS_PEAK_1690	17	6.66	Nup214	11614	105900524	1.786797881	up
MACS_PEAK_1704	24	9.69	Dab2ip	44242	6200441	1.777291298	down
MACS_PEAK_1705	51	19.95	Dab2ip	34295	6200441	1.777291298	down
MACS_PEAK_1706	27	9.34	Dab2ip	10704	6200441	1.777291298	down
MACS_PEAK_1714	58	27.26	Lrp1b	645790	102470176	1.663841486	up
MACS_PEAK_1717	25	7.88	Fmnl2	3227	4060438	2.74194026	down
MACS_PEAK_1717	25	7.88	Fmnl2	3227	104050215	5.00082922	down
MACS_PEAK_1718	19	5.78	Fmnl2	106756	4060438	2.74194026	down
MACS_PEAK_1718	19	5.78	Fmnl2	106756	104050215	5.00082922	down
MACS_PEAK_1719	38	16.36	Fmnl2	119242	4060438	2.74194026	down
MACS_PEAK_1719	38	16.36	Fmnl2	119242	104050215	5.00082922	down
MACS_PEAK_1720	40	15.52	Fmnl2	125210	4060438	2.74194026	down
MACS_PEAK_1720	40	15.52	Fmnl2	125210	104050215	5.00082922	down
MACS_PEAK_1728	40	21.2	Tanc1	13091	3450180	1.740174651	up
MACS_PEAK_1729	20	7.27	Tanc1	86532	3450180	1.740174651	up
MACS_PEAK_1738	18	6.66	Stk39	42305	50692	1.816866875	down
MACS_PEAK_1739	14	7.27	Gorasp2	13683	7040706	3.213990211	down
MACS_PEAK_1739	14	7.27	Gorasp2	13683	4060309	5.674937725	down
MACS_PEAK_1774	45	21.81	Dnajc24	34825	670095	2.561006069	down
MACS_PEAK_1779	17	7.27	Bbox1	128789	2030538	1.536293983	down
MACS_PEAK_1780	31	6.6	Bbox1	94848	2030538	1.536293983	down
MACS_PEAK_1797	26	13.93	Bmf	29411	102370026	2.116255999	up
MACS_PEAK_1798	19	9.09	Fbn1	86102	3170181	1.988004923	up
MACS_PEAK_1799	51	16.76	Fbn1	54533	3170181	1.988004923	up
MACS_PEAK_1803	20	7.88	Gabpb1	577	730021	2.331307411	down
MACS_PEAK_1803	20	7.88	Gabpb1	577	6900037	2.433291197	down
MACS_PEAK_1835	23	8.48	Rbbp9	99	2480139	2.113550901	down
MACS_PEAK_1861	112	56.95	Dlgap4	27584	5690161	1.559213519	up
MACS_PEAK_1861	112	56.95	Dlgap4	27584	105700670	1.550408244	up

MACS_PEAK_1874	81	10.67	Kcns1	28805	2570195	2.092708826	down
MACS_PEAK_1875	71	28.45	Kcns1	20445	2570195	2.092708826	down
MACS_PEAK_1903	47	23.02	Col9a3	404	4050541	1.575743914	down
MACS_PEAK_1904	35	15.75	Col9a3	2631	4050541	1.575743914	down
MACS_PEAK_1905	41	12.12	Col9a3	6696	4050541	1.575743914	down
MACS_PEAK_1906	41	17.57	Col9a3	13938	4050541	1.575743914	down
MACS_PEAK_1977	21	8.96	Spg20	15571	3360202	1.907341599	down
MACS_PEAK_1984	15	7.27	Wwtr1	12688	2570341	1.507518649	down
MACS_PEAK_1987	41	11.12	Mbnl1	3236	7100048	1.567408085	down
MACS_PEAK_1988	40	18.18	Mbnl1	26648	7100048	1.567408085	down
MACS_PEAK_1995	89	46.65	Mme	237623	6040673	1.818969607	down
MACS_PEAK_2001	48	26.05	Shox2	278757	6450059	2.458697319	up
MACS_PEAK_2002	19	7.24	Shox2	183291	6450059	2.458697319	up
MACS_PEAK_2006	23	12.12	Rapgef2	1359623	105080278	1.643891573	down
MACS_PEAK_2007	39	16.63	Rapgef2	252694	105080278	1.643891573	down
MACS_PEAK_2021	33	15.75	Scamp3	177	2360707	1.615253448	up
MACS_PEAK_2024	25	8.87	Zfp687	12466	2360672	2.247606039	up
MACS_PEAK_2029	17	8.48	Gja5	20205	1740026	1.517576218	up
MACS_PEAK_2048	17	8.48	Bcas2	22574	2340494	1.929402709	up
MACS_PEAK_2050	22	7.27	Olfml3	3734	7100463	1.811375737	up
MACS_PEAK_2053	24	9.98	Cttnbp2nl	8010	510451	1.536500096	down
MACS_PEAK_2054	17	8.48	Cttnbp2nl	156219	510451	1.536500096	down
MACS_PEAK_2059	67	19.05	Taf13	3506	100360687	1.507123232	up
MACS_PEAK_2065	48	16.96	Ntng1	7117	1410427	1.566989303	up
MACS_PEAK_2067	31	15.15	Col11a1	79864	2100300	3.070882559	down
MACS_PEAK_2067	31	15.15	Col11a1	79864	4810524	2.09778595	down
MACS_PEAK_2068	97	38.45	Slc35a3	344	4760066	1.659265637	down
MACS_PEAK_2068	97	38.45	Slc35a3	344	100060278	1.9222188	down
MACS_PEAK_2070	26	9.38	Snx7	11655	1090446	2.196594715	down
MACS_PEAK_2070	26	9.38	Snx7	11655	6980600	1.729214668	down
MACS_PEAK_2075	42	19.99	Sec24d	29368	3060279	3.02913022	down
MACS_PEAK_2077	18	9.09	Camk2d	9216	2370685	1.692703247	down
MACS_PEAK_2078	26	9.69	Camk2d	59473	2370685	1.692703247	down
MACS_PEAK_2079	23	9.69	Camk2d	98645	2370685	1.692703247	down
MACS_PEAK_2085	29	10.91	Elovl6	107970	103120253	1.853826523	up
MACS_PEAK_2086	27	11.68	Col25a1	19790	4480168	1.570342064	down
MACS_PEAK_2086	27	11.68	Col25a1	19790	1580072	1.931287408	down
MACS_PEAK_2087	36	11.38	Col25a1	179816	4480168	1.570342064	down
MACS_PEAK_2087	36	11.38	Col25a1	179816	1580072	1.931287408	down
MACS_PEAK_2098	22	9.09	Unc5c	44378	101090577	1.519138575	down
MACS_PEAK_2099	35	16.39	Unc5c	81900	101090577	1.519138575	down
MACS_PEAK_2100	34	9.69	Unc5c	135888	101090577	1.519138575	down
MACS_PEAK_2101	38	12.72	Unc5c	156009	101090577	1.519138575	down
MACS_PEAK_2139	14	8.48	Plekhf2	920	1850075	2.222766399	down
MACS_PEAK_2148	24	7.11	Bach2	119969	6760131	1.610206008	down
MACS_PEAK_2149	54	26.05	Bach2	112963	6760131	1.610206008	down
MACS_PEAK_2150	17	8.48	Bach2	13802	6760131	1.610206008	down
MACS_PEAK_2160	19	10.91	Sec61b	265002	4540687	1.893554211	down
MACS_PEAK_2169	52	24.05	Zfp462	308	6900100	3.295689821	up
MACS_PEAK_2174	22	7.27	Akap2	1861	102470053	5.325752258	down
MACS_PEAK_2183	16	9.69	Tle1	711731	103440167	1.573440909	down
MACS_PEAK_2184	94	8.92	Tle1	399031	103440167	1.573440909	down
MACS_PEAK_2185	29	9.09	Tle1	172811	103440167	1.573440909	down
MACS_PEAK_2218	30	8.13	Jun	54101	840170	1.758324981	up
MACS_PEAK_2219	39	10.83	Jun	92181	840170	1.758324981	up
MACS_PEAK_2220	16	7.27	Jun	133359	840170	1.758324981	up
MACS_PEAK_2242	29	11.08	Dab1	32085	7000605	1.782429457	down
MACS_PEAK_2243	30	9.35	Ppap2b	18124	4730280	4.089593887	down
MACS_PEAK_2244	22	7.11	Ppap2b	270580	4730280	4.089593887	down
MACS_PEAK_2245	21	7.88	Ppap2b	491797	4730280	4.089593887	down
MACS_PEAK_2260	50	13.85	Ptprf	29426	1770528	1.939987421	down
MACS_PEAK_2262	52	7.24	Col9a2	522	940670	3.261986256	down
MACS_PEAK_2263	25	7.03	Col9a2	1654	940670	3.261986256	down
MACS_PEAK_2264	25	10.3	Ppt1	68	6450440	2.951411963	down
MACS_PEAK_2265	44	25.45	Cap1	4215	2650278	1.58344388	up
MACS_PEAK_2266	20	7.88	Cap1	5871	2650278	1.58344388	up
MACS_PEAK_2268	23	11.51	Pou3f1	256568	3710022	2.063205719	up
MACS_PEAK_2269	24	7.08	Pou3f1	206286	3710022	2.063205719	up
MACS_PEAK_2281	24	6.66	Extl1	7082	2690136	3.756691933	down
MACS_PEAK_2309	23	7.27	Spsb1	60924	2850093	2.480366468	down
MACS_PEAK_2310	21	8.48	Spsb1	41018	2850093	2.480366468	down
MACS_PEAK_2314	30	8.13	Vamp3	297560	7100050	2.437590837	down
MACS_PEAK_2317	29	9.65	Acot7	27433	7050053	4.576246262	down
MACS_PEAK_2327	19	6.66	Akap9	61567	2060195	1.569421291	up
MACS_PEAK_2333	23	9.69	Sema3d	29560	103060037	2.825622559	down
MACS_PEAK_2334	21	7.88	Sema3d	59279	103060037	2.825622559	down
MACS_PEAK_2349	20	5.59	Dnajb6	27368	2810309	2.975131273	down
MACS_PEAK_2351	24	8.13	Maea	17595	1170392	4.912212849	down
MACS_PEAK_2365	19	6.66	Lcorl	875111	730195	1.54573679	up
MACS_PEAK_2372	24	9.69	Pcdh7	245039	103830706	1.887733936	up
MACS_PEAK_2389	39	10.95	Rasl11b	35998	102680427	4.432396889	down
MACS_PEAK_2390	22	8.48	Rasl11b	19654	102680427	4.432396889	down
MACS_PEAK_2391	35	15.66	Rasl11b	8809	102680427	4.432396889	down
MACS_PEAK_2392	20	4.88	Rasl11b	10387	102680427	4.432396889	down
MACS_PEAK_2393	48	15.75	Rasl11b	16069	102680427	4.432396889	down
MACS_PEAK_2394	18	7.27	Rasl11b	70945	102680427	4.432396889	down
MACS_PEAK_2395	59	21.84	Rasl11b	148124	102680427	4.432396889	down
MACS_PEAK_2399	22	9.98	Chic2	33603	4540722	2.126761436	down
MACS_PEAK_2403	25	11.51	Hopx	23080	103850021	2.034017324	down
MACS_PEAK_2430	38	14.07	Mtf2	122	50333	1.923413634	down
MACS_PEAK_2442	20	10.91	Ssh1	15990	106650592	1.837801218	up
MACS_PEAK_2455	17	6.66	Tbx5	32424	106840364	1.863815188	up
MACS_PEAK_2460	24	11.14	Fam101a	39196	5570040	2.988392353	down
MACS_PEAK_2461	81	38.03	Fam101a	10888	5570040	2.988392353	down
MACS_PEAK_2470	35	13.3	Wbscr17	219390	2630095	2.079356194	up
MACS_PEAK_2471	58	11.64	Wbscr17	389446	2630095	2.079356194	up
MACS_PEAK_2472	31	6.66	Wbscr17	403390	2630095	2.079356194	up
MACS_PEAK_2473	60	22.42	Wbscr17	468352	2630095	2.079356194	up
MACS_PEAK_2479	23	10.91	Gtf2i	461	106290711	3.137578249	down
MACS_PEAK_2483	23	6.57	Fzd9	13313	5360136	1.93507576	down
MACS_PEAK_2484	25	10.3	Fzd9	25545	5360136	1.93507576	down
MACS_PEAK_2485	15	8.48	Por	14218	6100093	1.717088819	up
MACS_PEAK_2496	17	8.48	Micall2	81593	1050292	1.728844047	up
MACS_PEAK_2502	29	12.19	Fscn1	6538	3450463	2.383372784	up
MACS_PEAK_2505	47	13.3	Cdk8	29496	1050113	1.871616602	down
MACS_PEAK_2530	31	12.12	Glcci1	88205	2640082	1.538626552	down
MACS_PEAK_2536	22	7.45	Foxp2	3072	4760524	1.72091639	down
MACS_PEAK_2536	22	7.45	Foxp2	3072	4150372	2.867068768	down
MACS_PEAK_2538	23	9.09	Tcfec	345929	1170722	1.795204163	up
MACS_PEAK_2542	25	12.12	A430107O13Rik	40661	3290075	1.644015312	down
MACS_PEAK_2544	24	8.48	Ptprz1	132862	100780411	1.908211827	down
MACS_PEAK_2544	24	8.48	Ptprz1	132862	105900121	2.008227348	down
MACS_PEAK_2545	26	10.3	Ptprz1	80998	100780411	1.908211827	down
MACS_PEAK_2545	26	10.3	Ptprz1	80998	105900121	2.008227348	down
MACS_PEAK_2555	44	15.15	Mest	7357	6620292	1.687818408	down
MACS_PEAK_2555	44	15.15	Mest	7357	4780440	8.560221672	down
MACS_PEAK_2593	21	7.88	Grid2	195928	103190133	2.567212582	up
MACS_PEAK_2605	66	17.57	Dok1	33185	2680112	2.928308487	down
MACS_PEAK_2605	66	17.57	Dok1	33185	5220273	2.811432123	down
MACS_PEAK_2609	45	19.95	Cyp26b1	71308	2630142	2.649281502	down
MACS_PEAK_2617	42	11.43	Klf15	8737	4230164	1.65427351	down
MACS_PEAK_2650	54	18.72	Vgll4	120974	6860463	1.723965287	down

MACS_PEAK_2651	75	33.2	Vgll4	117320	6860463	1.723965287	down
MACS_PEAK_2652	70	29.13	Vgll4	38327	6860463	1.723965287	down
MACS_PEAK_2653	29	13.93	Vgll4	42066	6860463	1.723965287	down
MACS_PEAK_2670	23	7.97	Lrp6	28948	5130064	1.753937602	down
MACS_PEAK_2706	64	15.72	Lrmp	17370	6290193	2.638993025	up
MACS_PEAK_2721	21	8.48	Arhgef1	3238	100110544	1.593886375	up
MACS_PEAK_2723	60	17.78	Cic	2080	6370161	1.930728555	up
MACS_PEAK_2730	36	6.1	Fxyd3	983	2320450	1.668602943	down
MACS_PEAK_2732	30	12.72	Zfp507	287468	103840497	1.507071495	up
MACS_PEAK_2738	18	7.27	Nosip	312	6380064	1.728900671	down
MACS_PEAK_2744	28	12.72	Gas2	35267	1340082	2.430493116	up
MACS_PEAK_2757	20	7.27	Arrdc4	307872	100870044	2.774963856	up
MACS_PEAK_2758	30	7.97	Arrdc4	339174	100870044	2.774963856	up
MACS_PEAK_2769	34	11.64	Acan	62978	430647	3.726875544	down
MACS_PEAK_2769	34	11.64	Acan	62978	4760092	6.213802338	down
MACS_PEAK_2770	22	9.98	Acan	31905	430647	3.726875544	down
MACS_PEAK_2770	22	9.98	Acan	31905	4760092	6.213802338	down
MACS_PEAK_2771	57	30.29	Acan	26941	430647	3.726875544	down
MACS_PEAK_2771	57	30.29	Acan	26941	4760092	6.213802338	down
MACS_PEAK_2790	33	8.71	Fam181b	17623	3120072	1.706959963	down
MACS_PEAK_2800	22	9.69	P4ha3	2890	100630484	1.80704093	down
MACS_PEAK_2800	22	9.69	P4ha3	2890	6520037	2.543177605	down
MACS_PEAK_2802	40	14.54	Plekhb1	2900	870463	1.838961244	down
MACS_PEAK_2814	39	14.96	Dkk3	11356	7050128	1.85215342	down
MACS_PEAK_2835	37	8.91	Igsf6	4713	5340348	1.535048485	up
MACS_PEAK_2836	20	6.66	Tnrc6a	25653	1240537	1.597840071	up
MACS_PEAK_2846	35	11.51	Fgfr2	82723	5570402	4.836047649	down
MACS_PEAK_2846	35	11.51	Fgfr2	82723	100780364	1.819450498	down
MACS_PEAK_2846	35	11.51	Fgfr2	82723	4670601	1.686376214	down
MACS_PEAK_2854	40	21.2	Ptpre	5391	1660286	1.759332657	up
MACS_PEAK_2881	30	8.61	Arglu1	513967	6510398	1.500149727	up
MACS_PEAK_2883	26	9.98	Col4a1	146999	1740575	3.014300823	up
MACS_PEAK_2887	24	9.69	Mcf2l	15690	1570056	2.18674159	up
MACS_PEAK_2898	36	16.96	Ppp1r3b	163560	6130253	2.058467388	down
MACS_PEAK_2900	45	14.81	Dlc1	58858	1090632	4.186814785	down
MACS_PEAK_2901	35	16.96	Dlc1	32701	1090632	4.186814785	down
MACS_PEAK_2912	58	10.97	Sorbs2	57019	6350112	1.909857392	down
MACS_PEAK_2913	20	6.45	Sorbs2	121170	6350112	1.909857392	down
MACS_PEAK_2920	22	8.48	Cdkn2aip	29340	3060670	1.746379733	up
MACS_PEAK_2930	22	10.3	Palld	193470	102060332	2.444493532	up
MACS_PEAK_2932	24	6.45	Cpe	17867	2940441	4.363157749	down
MACS_PEAK_2937	23	7.88	Csgalnact1	29132	5390746	1.569012284	up
MACS_PEAK_2938	21	6.65	Csgalnact1	7359	5390746	1.569012284	up
MACS_PEAK_2941	39	19.99	Comp	6705	3800273	1.549821734	down
MACS_PEAK_2947	29	11.25	Lsm6	78548	104050600	1.530593514	up
MACS_PEAK_2963	34	8.92	Cbln1	122663	5670358	2.384750605	up
MACS_PEAK_2969	74	22.42	Sall1	266519	5420020	1.546113729	down
MACS_PEAK_2970	33	10.91	Sall1	588255	5420020	1.546113729	down
MACS_PEAK_2977	58	21.2	Cdh8	1455340	2100348	1.914093852	up
MACS_PEAK_2986	18	6.66	Cdh5	314638	5340487	2.503920078	up
MACS_PEAK_2989	43	13.33	Wwp2	21902	6550079	1.589534521	down
MACS_PEAK_2989	43	13.33	Wwp2	21902	1090102	5.120452404	down
MACS_PEAK_2990	52	12.75	Wwp2	29170	6550079	1.589534521	down
MACS_PEAK_2990	52	12.75	Wwp2	29170	1090102	5.120452404	down
MACS_PEAK_2991	70	25.45	Wwp2	32761	6550079	1.589534521	down
MACS_PEAK_2991	70	25.45	Wwp2	32761	1090102	5.120452404	down
MACS_PEAK_2992	27	9.69	Wwp2	36561	6550079	1.589534521	down
MACS_PEAK_2992	27	9.69	Wwp2	36561	1090102	5.120452404	down
MACS_PEAK_2993	37	12.19	Wwp2	42636	6550079	1.589534521	down
MACS_PEAK_2993	37	12.19	Wwp2	42636	1090102	5.120452404	down
MACS_PEAK_2994	36	10.91	Wwp2	55153	6550079	1.589534521	down
MACS_PEAK_2994	36	10.91	Wwp2	55153	1090102	5.120452404	down
MACS_PEAK_3010	22	6.6	Foxf1a	20658	6180017	1.569296002	down
MACS_PEAK_3025	34	12.72	Kcnk1	78288	1050068	1.755455256	down
MACS_PEAK_3031	2999	2.14	Itgb1	30899	5080156	1.658383369	down
MACS_PEAK_3039	29	13.93	Sesn3	83392	110687	2.475210667	down
MACS_PEAK_3041	35	16.96	Fat3	150057	4670324	1.660501838	up
MACS_PEAK_3045	21	8.48	Ilf3	13328	6520110	1.76270628	down
MACS_PEAK_3054	43	14.54	Barx2	189958	4480273	1.63368845	down
MACS_PEAK_3054	43	14.54	Barx2	189958	105360403	2.952434301	down
MACS_PEAK_3086	20	10.3	AI593442	114517	6960605	2.21384573	up
MACS_PEAK_3089	26	11.51	Cspg4	16653	2970270	1.713827133	down
MACS_PEAK_3093	42	15.54	Arid3b	2455	6650008	1.576095343	up
MACS_PEAK_3096	23	8.42	Neo1	121948	100730537	1.652311444	down
MACS_PEAK_3099	65	26.04	Thsd4	25332	6040037	2.296028614	up
MACS_PEAK_3100	24	9.42	Thsd4	103471	6040037	2.296028614	up
MACS_PEAK_3114	22	8.48	Itga11	64624	1740112	1.52748549	down
MACS_PEAK_3119	26	6.57	Rab11a	73032	5360079	3.261560678	down
MACS_PEAK_3131	30	6.06	Tcf12	151950	3610324	1.968023896	down
MACS_PEAK_3137	17	6.66	Bmp5	6452	4670048	1.953491926	down
MACS_PEAK_3145	31	13.33	Col12a1	259166	100110731	1.992657542	down
MACS_PEAK_3157	25	12.72	1190002N15Rik	55253	4390040	4.486179829	down
MACS_PEAK_3165	29	13.33	Amotl2	36074	4730082	1.523490906	down
MACS_PEAK_3166	68	28.47	Amotl2	16618	4730082	1.523490906	down
MACS_PEAK_3167	29	11.08	Amotl2	6378	4730082	1.523490906	down
MACS_PEAK_3176	46	11.08	Rbm5	183	3520402	3.3000741	up
MACS_PEAK_3184	22	8.48	Clasp2	3302	104070121	2.483433247	down
MACS_PEAK_3192	22	6.97	Itga9	36360	670180	1.867954493	up
MACS_PEAK_3220	26	11.51	Bcor	73684	3940053	1.556888342	down
MACS_PEAK_3221	36	16.96	Bcor	11012	3940053	1.556888342	down
MACS_PEAK_3222	27	10.3	Atp6ap2	177637	4010059	6.724124908	down
MACS_PEAK_3222	27	10.3	Atp6ap2	177637	7100347	6.21543026	down
MACS_PEAK_3244	33	6.66	Tlr13	49440	4810546	1.988589168	up
MACS_PEAK_3249	28	13.93	Capn6	21091	1740168	2.12635541	down
MACS_PEAK_31	22	8.87	1500015O10Rik	181726	3130368	1.779098392	down
MACS_PEAK_32	19	6.06	1500015O10Rik	146834	3130368	1.779098392	down
MACS_PEAK_37	22	9.69	Col5a2	108798	104670129	2.087709427	up
MACS_PEAK_42	40	7.88	Myo1b	81459	101410168	1.891579628	down
MACS_PEAK_42	40	7.88	Myo1b	81459	105910021	2.122879982	down
MACS_PEAK_43	23	6.65	Nab1	245920	6660056	2.304262161	down
MACS_PEAK_44	20	6.9	Pms1	94944	4540164	1.542927265	up
MACS_PEAK_60	25	12.12	Nrp2	628174	6650446	1.683444738	down
MACS_PEAK_61	30	11.08	Nrp2	547985	6650446	1.683444738	down
MACS_PEAK_71	22	6.66	Atic	173058	6380463	3.777183056	down
MACS_PEAK_71	22	6.66	Atic	173058	4010593	1.716966033	down
MACS_PEAK_72	28	9.85	Fn1	232898	6220288	3.997449636	down
MACS_PEAK_72	28	9.85	Fn1	232898	2970647	4.103619576	down
MACS_PEAK_72	28	9.85	Fn1	232898	1170601	1.853502989	down
MACS_PEAK_83	28	13.93	Slc4a3	479097	360603	1.932770133	up
MACS_PEAK_84	23	9.09	Slc4a3	544307	360603	1.932770133	up
MACS_PEAK_85	18	8.31	Slc4a3	565059	360603	1.932770133	up
MACS_PEAK_87	23	10.91	Epha4	501660	460750	1.665304303	up
MACS_PEAK_103	43	19.99	Hdac4	231047	460079	1.829596639	up
MACS_PEAK_108	30	12.72	Gin1	153499	104760605	1.599875689	down
MACS_PEAK_116	51	16.26	Gli2	335421	3060632	1.640829682	down
MACS_PEAK_117	39	14.96	Gli2	253774	3060632	1.640829682	down
MACS_PEAK_119	16	6.66	Ralb	67079	6130397	1.575715542	up
MACS_PEAK_134	33	16.96	Tmcc2	28759	6900762	1.511455655	up
MACS_PEAK_144	20	7.27	Hmcn1	458499	104570670	3.694500208	down
MACS_PEAK_158	18	9.51	Angptl1	97823	104920372	2.244338512	down
MACS_PEAK_159	22	9.42	Fam20b	89613	2030215	1.650509834	down
MACS_PEAK_164	82	13	Dnm3os	217321	102940671	2.063773394	down

MACS_PEAK_165	51	9.09	Dnm3os	202855	102940671	2.063773394	down
MACS_PEAK_166	66	22.42	Dnm3os	200520	102940671	2.063773394	down
MACS_PEAK_167	17	8.48	Dnm3os	197067	102940671	2.063773394	down
MACS_PEAK_168	172	67.85	Dnm3os	180919	102940671	2.063773394	down
MACS_PEAK_169	15	8.48	Dnm3os	158916	102940671	2.063773394	down
MACS_PEAK_183	36	13.72	Atp1a2	10018	110278	1.724053264	down
MACS_PEAK_184	20	7.27	Slamf9	69024	3710300	1.585953236	up
MACS_PEAK_185	20	6.06	Cep170	7110	4850575	2.833632231	down
MACS_PEAK_186	22	12.12	Zfp238	243515	5050537	2.892208099	down
MACS_PEAK_187	15	7.27	Zfp238	105552	5050537	2.892208099	down
MACS_PEAK_200	66	18.29	Lbr	307059	107040471	3.998609781	down
MACS_PEAK_221	18	8.14	Utrn	1369568	3390048	3.522297144	down
MACS_PEAK_225	24	9.69	Pex3	161566	2760100	2.427631855	down
MACS_PEAK_230	23	9.42	Pex7	28911	6660270	1.907020807	down
MACS_PEAK_231	25	9.65	Ahi1	61357	460520	1.553654432	up
MACS_PEAK_232	23	10.3	Ahi1	72399	460520	1.553654432	up
MACS_PEAK_240	20	9.09	Tpd52l1	146343	5570575	2.081928015	down
MACS_PEAK_240	20	9.09	Tpd52l1	146343	4590711	2.67099905	down
MACS_PEAK_265	21	8.48	Cd24a	23266	1780091	1.825586438	up
MACS_PEAK_266	31	9.69	Cd24a	42573	1780091	1.825586438	up
MACS_PEAK_279	60	21.34	Hsf2	617053	1400465	1.642094493	up
MACS_PEAK_281	87	26.26	Ccdc109a	154120	106520647	4.056745529	down
MACS_PEAK_301	49	19.99	Mif	21401	105270133	1.607923269	down
MACS_PEAK_302	28	13.33	Pcnt	79479	4760053	1.886447549	up
MACS_PEAK_303	38	14.96	Pcbp3	231631	940332	1.630687714	up
MACS_PEAK_323	26	7.27	Tmpo	687637	4050494	4.254616261	down
MACS_PEAK_324	24	8.53	Tmpo	864763	4050494	4.254616261	down
MACS_PEAK_333	18	7.88	Dcn	653063	510332	3.202011585	down
MACS_PEAK_336	16	7.27	Atp2b1	622796	104150181	1.961512804	down
MACS_PEAK_343	31	9.65	Zdhhc17	79215	104050288	2.077921391	down
MACS_PEAK_346	204	100.57	Phlda1	419342	2450020	2.164865017	down
MACS_PEAK_349	40	19.99	Zfc3h1	134065	4070706	1.60190773	down
MACS_PEAK_350	36	12.72	Lgr5	224190	6020400	2.284122944	down
MACS_PEAK_354	21	7.88	Yeats4	61668	3130242	3.189333439	down
MACS_PEAK_356	73	35.14	Cpm	43602	103440025	1.511164546	up
MACS_PEAK_366	21	7.88	Msrb3	26677	103140053	2.706700563	down
MACS_PEAK_366	21	7.88	Msrb3	26677	510014	1.54449749	down
MACS_PEAK_386	21	8.48	Igfbp3	1210735	2370500	5.452507973	down
MACS_PEAK_393	21	8.48	Grb10	397336	106110546	2.013310194	up
MACS_PEAK_407	22	6.1	Spnb2	147853	380091	2.274885654	down
MACS_PEAK_412	26	10.91	Slit3	391394	106650441	2.003383636	up
MACS_PEAK_427	19	6.1	Gfpt2	51075	102810242	1.50763905	down
MACS_PEAK_429	22	5.59	Clk4	143478	3610156	11.9104557	down
MACS_PEAK_431	15	7.88	Hspa4	381116	1050170	1.625148892	up
MACS_PEAK_448	28	12.12	2810001G20Rik	55190	102320292	1.537947655	down
MACS_PEAK_449	63	29.08	Hs3st3a1	224168	2230497	1.850928664	down
MACS_PEAK_450	33	12.12	2810001G20Rik	242736	102320292	1.537947655	down
MACS_PEAK_451	29	9.69	Elac2	429511	3440167	1.903346419	down
MACS_PEAK_455	81	29.08	Myh10	53448	2640673	1.793132901	down
MACS_PEAK_483	41	13.3	Rad51c	18716	3780592	2.507941961	down
MACS_PEAK_484	25	6.66	Rad51c	22434	3780592	2.507941961	down
MACS_PEAK_485	89	7.86	Rad51c	38837	3780592	2.507941961	down
MACS_PEAK_486	16	5.45	Rad51c	44338	3780592	2.507941961	down
MACS_PEAK_487	17	5.45	Rad51c	58031	3780592	2.507941961	down
MACS_PEAK_491	26	8.44	Msi2	409121	103140390	2.370386839	down
MACS_PEAK_492	46	19.99	Akap1	86193	2260019	2.008526325	down
MACS_PEAK_492	46	19.99	Akap1	86193	110148	2.098371983	down
MACS_PEAK_493	33	10.3	Akap1	80948	2260019	2.008526325	down
MACS_PEAK_493	33	10.3	Akap1	80948	110148	2.098371983	down
MACS_PEAK_515	25	10.35	Myl4	87658	6860288	2.878082514	up
MACS_PEAK_516	22	8.21	Tlk2	104341	1170161	2.544542074	up
MACS_PEAK_518	42	13.93	Pitpnc1	246585	3990017	2.315482378	down
MACS_PEAK_570	18	7.03	Osr1	407844	1500025	1.59414053	up
MACS_PEAK_570	18	7.03	Osr1	407844	5270706	2.1889112	up
MACS_PEAK_582	18	4.7	2410018L13Rik	1565775	103130193	2.196941376	down
MACS_PEAK_583	23	9.69	2410018L13Rik	904410	103130193	2.196941376	down
MACS_PEAK_595	29	16.36	Cbll1	44694	102030487	2.064683437	up
MACS_PEAK_609	19	8.31	Etv1	1159437	70735	4.297760963	down
MACS_PEAK_609	19	8.31	Etv1	1159437	5080463	3.938217402	down
MACS_PEAK_610	25	9.79	Etv1	1233296	70735	4.297760963	down
MACS_PEAK_610	25	9.79	Etv1	1233296	5080463	3.938217402	down
MACS_PEAK_623	27	9.09	Sec23a	581978	5860035	11.70175171	down
MACS_PEAK_624	29	12.72	Sec23a	460966	5860035	11.70175171	down
MACS_PEAK_629	25	9.09	Frmd6	292452	4670019	1.692186475	up
MACS_PEAK_631	19	7.76	Actr10	59884	5860458	3.908699751	down
MACS_PEAK_640	15	7.88	Mnat1	100349	2350364	1.589990497	down
MACS_PEAK_641	14	8.48	Mnat1	131615	2350364	1.589990497	down
MACS_PEAK_670	23	6.65	0610007P14Rik	65930	6450273	8.547762871	down
MACS_PEAK_692	34	8.64	Papola	287146	4200286	2.137450933	down
MACS_PEAK_693	47	19.39	Bcl11b	1229588	106860278	1.845429659	down
MACS_PEAK_694	28	11.14	Bcl11b	1217871	106860278	1.845429659	down
MACS_PEAK_695	102	49.07	Bcl11b	1206516	106860278	1.845429659	down
MACS_PEAK_710	31	11.18	Itgb8	162951	105050025	1.710262179	up
MACS_PEAK_714	44	14.74	Klf6	1089990	110541	1.667094707	up
MACS_PEAK_727	17	9.09	Zfp184	46906	101770338	1.631226897	up
MACS_PEAK_728	19	6.66	Zfp184	30011	101770338	1.631226897	up
MACS_PEAK_740	32	8.64	Sox4	535687	2260091	2.331992149	up
MACS_PEAK_745	26	8.48	Sox4	463494	2260091	2.331992149	up
MACS_PEAK_753	27	15.15	Gmds	374534	1050519	1.810869336	down
MACS_PEAK_754	56	15.68	Gmds	367587	1050519	1.810869336	down
MACS_PEAK_755	28	4.85	Gmds	345783	1050519	1.810869336	down
MACS_PEAK_756	19	7.88	Gmds	329377	1050519	1.810869336	down
MACS_PEAK_757	139	70.88	Gmds	301428	1050519	1.810869336	down
MACS_PEAK_758	18	7.88	Gmds	298219	1050519	1.810869336	down
MACS_PEAK_759	120	21.57	Gmds	289901	1050519	1.810869336	down
MACS_PEAK_765	26	12.12	Gmds	228114	1050519	1.810869336	down
MACS_PEAK_769	18	6.06	1300014I06Rik	264417	3780673	2.328416109	down
MACS_PEAK_770	20	8.48	1300014I06Rik	173370	3780673	2.328416109	down
MACS_PEAK_777	15	7.88	Tmem14c	33378	670647	2.00219965	down
MACS_PEAK_801	71	29.07	Gadd45g	85237	2510142	1.811520219	down
MACS_PEAK_842	18	6.66	Cetn3	1360205	3170402	1.552112699	down
MACS_PEAK_843	33	15.75	Cetn3	1429488	3170402	1.552112699	down
MACS_PEAK_868	23	11.51	Zbed3	50413	3290040	1.952820301	up
MACS_PEAK_869	31	9.09	Zbed3	7553	3290040	1.952820301	up
MACS_PEAK_879	23	10.91	Mtap1b	68490	6040435	1.969672322	down
MACS_PEAK_879	23	10.91	Mtap1b	68490	2230332	4.394807816	down
MACS_PEAK_880	30	9.09	Mtap1b	299352	6040435	1.969672322	down
MACS_PEAK_880	30	9.09	Mtap1b	299352	2230332	4.394807816	down
MACS_PEAK_882	24	12.72	Cdk7	38207	102230128	1.786380172	up
MACS_PEAK_900	20	8.48	Gpbp1	222517	2060162	1.781497478	down
MACS_PEAK_901	33	11.76	Gpbp1	197895	2060162	1.781497478	down
MACS_PEAK_903	49	15.78	Esm1	208498	1410594	1.808077216	up
MACS_PEAK_904	27	8.71	Esm1	377861	1410594	1.808077216	up
MACS_PEAK_912	23	6.06	Emb	247121	610270	1.961876035	down
MACS_PEAK_927	78	27.94	2700060E02Rik	96667	4150091	4.437078953	down
MACS_PEAK_928	33	15.15	Adk	410029	520180	6.292619705	down
MACS_PEAK_929	44	15.24	Adk	423551	520180	6.292619705	down
MACS_PEAK_971	39	16.93	Dhrs4	199071	630670	2.238277435	down
MACS_PEAK_998	23	9.42	Slc25a30	18902	6840707	2.348610163	down
MACS_PEAK_1006	86	25.45	9030625A04Rik	348436	2350441	2.277412415	down
MACS_PEAK_1007	48	9.69	9030625A04Rik	237443	2350441	2.277412415	down
MACS_PEAK_1016	19	8.48	Pcdh17	4175124	103840008	1.968291402	up

MACS_PEAK_1017	21	9.69	Pcdh17	4155712	103840008	1.968291402	up
MACS_PEAK_1024	21	7.88	Dach1	476018	2450593	1.550462127	down
MACS_PEAK_1025	82	44.83	Dach1	820064	2450593	1.550462127	down
MACS_PEAK_1027	21	6.65	Klf12	917167	1660095	1.634319186	up
MACS_PEAK_1028	42	21.2	Commd6	146195	1660474	1.511464834	down
MACS_PEAK_1059	23	9.09	Pcca	341964	3390400	2.243657351	down
MACS_PEAK_1075	22	6.66	Trio	245832	102320605	1.6330235	down
MACS_PEAK_1075	22	6.66	Trio	245832	103360497	1.626712561	down
MACS_PEAK_1076	31	11.51	Trio	237467	102320605	1.6330235	down
MACS_PEAK_1076	31	11.51	Trio	237467	103360497	1.626712561	down
MACS_PEAK_1077	15	6.66	Trio	227925	102320605	1.6330235	down
MACS_PEAK_1077	15	6.66	Trio	227925	103360497	1.626712561	down
MACS_PEAK_1090	19	6.66	Cthrc1	58521	103850632	7.363954544	down
MACS_PEAK_1092	33	8.53	Zfpm2	540055	460072	1.900486469	down
MACS_PEAK_1094	25	10.3	Zfpm2	724924	460072	1.900486469	down
MACS_PEAK_1105	29	15.75	Mtbp	243202	50193	3.607527256	down
MACS_PEAK_1106	20	8.48	Mtbp	283089	50193	3.607527256	down
MACS_PEAK_1110	37	5.23	Has2	467970	5360181	2.12053895	down
MACS_PEAK_1115	44	20.6	Nsmce2	174822	1770168	4.732778072	down
MACS_PEAK_1116	47	18.78	Nsmce2	260869	1770168	4.732778072	down
MACS_PEAK_1142	32	15.75	Mkl1	71920	101170039	3.165697575	up
MACS_PEAK_1143	21	7.88	Rangap1	32548	2320593	3.907580376	down
MACS_PEAK_1151	21	7.88	Lrrk2	162297	4670707	1.88201201	up
MACS_PEAK_1154	28	12.72	Pdzrn4	414213	103120746	1.600646615	down
MACS_PEAK_1160	17	7.76	Prickle1	599160	103190427	3.681575537	down
MACS_PEAK_1207	24	7.88	Comt	32833	360687	1.879665375	down
MACS_PEAK_1212	17	6.06	Etv5	172890	110017	1.707441449	down
MACS_PEAK_1213	31	13.33	Etv5	98088	110017	1.707441449	down
MACS_PEAK_1227	16	7.88	Pcyt1a	41212	1450619	1.650879741	up
MACS_PEAK_1229	29	12.72	Snx4	74792	106520008	1.918561816	up
MACS_PEAK_1237	181	8.81	Pdia5	94730	5550040	2.353853941	down
MACS_PEAK_1241	23	12.3	Lsamp	2488806	2810056	1.558461189	down
MACS_PEAK_1247	21	10.3	Phldb2	92384	6590685	2.147843361	up
MACS_PEAK_1250	18	7.88	Cblb	1184868	106520066	2.736266136	up
MACS_PEAK_1251	39	14.54	Cblb	913638	106520066	2.736266136	up
MACS_PEAK_1264	68	34.91	Cxadr	387157	106550400	2.025967121	down
MACS_PEAK_1290	40	21.2	Dyrk1a	256293	102940021	1.69041121	up
MACS_PEAK_1295	21	7.88	Ets2	115552	360451	1.974570274	down
MACS_PEAK_1297	50	16.96	Ets2	146293	360451	1.974570274	down
MACS_PEAK_1298	41	13.5	Ets2	220301	360451	1.974570274	down
MACS_PEAK_1299	22	8.31	Ets2	222681	360451	1.974570274	down
MACS_PEAK_1312	19	7.27	T	286933	6370164	1.527101755	down
MACS_PEAK_1325	24	9.69	Chd1	103906	4590048	2.344444752	down
MACS_PEAK_1345	99	5.76	H2-T23	11684	102340121	1.792632103	up
MACS_PEAK_1345	99	5.76	H2-T23	11684	2850609	1.710852146	up
MACS_PEAK_1380	36	4.81	Ptprm	695005	6370136	1.623142838	down
MACS_PEAK_1407	33	14.54	Slc8a1	1410190	3440717	1.785566211	up
MACS_PEAK_1407	33	14.54	Slc8a1	1410190	106290288	1.83786571	up
MACS_PEAK_1408	28	8.48	Slc8a1	1418498	3440717	1.785566211	up
MACS_PEAK_1408	28	8.48	Slc8a1	1418498	106290288	1.83786571	up
MACS_PEAK_1415	23	6.65	Srbd1	432027	6980750	1.95819664	up
MACS_PEAK_1416	34	12.12	Srbd1	275653	6980750	1.95819664	up
MACS_PEAK_1417	55	20.16	Srbd1	274322	6980750	1.95819664	up
MACS_PEAK_1418	55	29.69	Srbd1	242386	6980750	1.95819664	up
MACS_PEAK_1419	36	11.25	Srbd1	230341	6980750	1.95819664	up
MACS_PEAK_1427	21	7.88	Cul2	126215	102190519	1.710577726	down
MACS_PEAK_1447	23	9.09	Zfp521	891151	540541	2.007312298	down
MACS_PEAK_1448	20	8.48	Zfp521	512522	540541	2.007312298	down
MACS_PEAK_1449	21	6.66	Ss18	829666	6900129	1.854062438	down
MACS_PEAK_1450	26	7.84	Ss18	739907	6900129	1.854062438	down
MACS_PEAK_1451	52	24.23	Ss18	691786	6900129	1.854062438	down
MACS_PEAK_1452	42	23.02	Ss18	22536	6900129	1.854062438	down
MACS_PEAK_1456	25	9.66	Ino80c	100891	2510632	1.872328758	down
MACS_PEAK_1489	23	9.42	Dmxl1	60281	101500465	2.709820271	down
MACS_PEAK_1495	20	7.2	Snx24	102610	5720398	1.611333132	down
MACS_PEAK_1549	24	7.42	Dcc	935633	101230170	1.609910727	up
MACS_PEAK_1570	20	7.88	1700034H14Rik	746729	6100348	2.242739677	down
MACS_PEAK_1570	20	7.88	1700034H14Rik	746729	840021	5.542374134	down
MACS_PEAK_1579	18	9.09	Mpeg1	27580	103780671	12.81386948	up
MACS_PEAK_1584	29	12.72	Gnaq	82026	430670	1.843773246	down
MACS_PEAK_1587	50	15.75	Gcnt1	13749	940129	1.885344744	down
MACS_PEAK_1587	50	15.75	Gcnt1	13749	1780215	2.185174465	down
MACS_PEAK_1593	35	11.08	Fam108b	128903	6650487	2.886203051	down
MACS_PEAK_1594	63	7.34	Tmem2	536987	3290020	1.734024167	down
MACS_PEAK_1599	84	28.3	Kank1	158288	3450546	5.633127213	down
MACS_PEAK_1600	47	18.78	Kank1	164584	3450546	5.633127213	down
MACS_PEAK_1604	28	9.09	Ranbp6	121079	360164	2.431586504	down
MACS_PEAK_1632	49	12.91	Smc3	92211	870546	2.958533287	down
MACS_PEAK_1633	27	12.75	Gpam	788101	1740180	1.720186472	down
MACS_PEAK_1647	21	6.06	Slc18a2	64124	6420541	1.563637495	down
MACS_PEAK_1661	24	8.13	Atp5c1	90305	102630438	1.586648583	down
MACS_PEAK_1666	21	6.66	Arl5b	1115709	3840059	1.855834484	up
MACS_PEAK_1677	33	10.02	Sec16a	42148	5690280	1.556519032	up
MACS_PEAK_1678	25	8.78	Sec16a	56595	5690280	1.556519032	up
MACS_PEAK_1688	14	7.27	Crat	26178	540020	1.672328115	up
MACS_PEAK_1710	20	9.14	Psmb7	128234	5890647	3.241960049	down
MACS_PEAK_1711	83	42.41	Psmb7	103450	5890647	3.241960049	down
MACS_PEAK_1716	37	17.57	Nmi	64809	520270	2.010234594	up
MACS_PEAK_1721	16	7.27	Fmnl2	195942	4060438	2.74194026	down
MACS_PEAK_1721	16	7.27	Fmnl2	195942	104050215	5.00082922	down
MACS_PEAK_1723	76	34.53	Cytip	104466	1780408	1.794008255	up
MACS_PEAK_1754	25	13.33	Pde1a	109544	1050458	1.505442142	up
MACS_PEAK_1775	16	8.48	Dnajc24	412181	670095	2.561006069	down
MACS_PEAK_1776	36	14.54	Dnajc24	421568	670095	2.561006069	down
MACS_PEAK_1781	47	15.75	Bbox1	47773	2030538	1.536293983	down
MACS_PEAK_1784	27	13.93	Aqr	67659	106760358	1.522784352	up
MACS_PEAK_1803	20	7.88	Usp8	31299	2640347	3.155760288	down
MACS_PEAK_1825	24	9.98	Mkks	16214	2760722	2.561979294	down
MACS_PEAK_1826	58	28.47	Jag1	1161889	3440390	2.487917185	up
MACS_PEAK_1844	30	10.32	Pygb	21076	2510731	1.859453082	up
MACS_PEAK_1881	54	23.63	Ube2v1	88415	2470093	3.053926945	down
MACS_PEAK_1882	53	16.66	Ptpn1	204998	104070408	1.964437604	up
MACS_PEAK_1895	36	9.87	Pmepa1	143687	105910082	1.68865335	up
MACS_PEAK_1914	18	9.69	Zfp704	141210	4120452	1.911498547	up
MACS_PEAK_1915	19	5.3	Zfp704	185160	4120452	1.911498547	up
MACS_PEAK_1919	32	15.75	Zfand1	30443	5080215	1.753351569	up
MACS_PEAK_1920	60	19.39	Zfand1	36170	5080215	1.753351569	up
MACS_PEAK_1986	20	12.12	Mbnl1	360935	7100048	1.567408085	down
MACS_PEAK_1989	29	12.72	Mbnl1	270859	7100048	1.567408085	down
MACS_PEAK_2000	18	7.88	Ptx3	72728	3520102	2.430686712	down
MACS_PEAK_2000	18	7.88	Ptx3	72728	870309	2.150045156	down
MACS_PEAK_2008	28	12.72	Rapgef2	127676	105080278	1.643891573	down
MACS_PEAK_2014	53	29.08	Sfrp2	156778	4850097	2.048348904	down
MACS_PEAK_2015	26	9.09	Sfrp2	125690	4850097	2.048348904	down
MACS_PEAK_2018	21	8.48	Fcrls	112687	60204	17.65731239	up
MACS_PEAK_2019	21	7.42	Crabp2	11960	6940725	1.603922963	up
MACS_PEAK_2047	65	26.66	Tspan2	25678	3940161	2.072137594	down
MACS_PEAK_2049	19	8.05	Olfml3	87355	7100463	1.811375737	up
MACS_PEAK_2052	46	21.81	Cttnbp2nl	58965	510451	1.536500096	down
MACS_PEAK_2057	37	13.93	Psma5	49426	5390537	2.644881964	up
MACS_PEAK_2066	17	7.88	Col11a1	331330	2100300	3.070882559	down
MACS_PEAK_2066	17	7.88	Col11a1	331330	4810524	2.09778595	down

MACS_PEAK_2071	39	15.64	Alg14	98578	5390204	1.762085676	down
MACS_PEAK_2084	26	8.31	Pitx2	851992	870537	2.135507107	down
MACS_PEAK_2088	48	16.36	Col25a1	270105	4480168	1.570342064	down
MACS_PEAK_2088	48	16.36	Col25a1	270105	1580072	1.931287408	down
MACS_PEAK_2094	25	9.65	Ppp3ca	597210	4760332	1.671500087	down
MACS_PEAK_2095	56	19.7	Ppp3ca	619092	4760332	1.671500087	down
MACS_PEAK_2138	41	19.39	Asph	210125	2810064	2.211745739	down
MACS_PEAK_2138	41	19.39	Asph	210125	4280133	3.164973259	down
MACS_PEAK_2138	41	19.39	Asph	210125	3140040	1.784584761	down
MACS_PEAK_2138	41	19.39	Asph	210125	6370671	2.434479952	down
MACS_PEAK_2138	41	19.39	Asph	210125	6940338	1.963395834	down
MACS_PEAK_2138	41	19.39	Asph	210125	2100066	1.670410752	down
MACS_PEAK_2138	41	19.39	Asph	210125	2970008	2.169843674	down
MACS_PEAK_2138	41	19.39	Asph	210125	360722	1.685213923	down
MACS_PEAK_2138	41	19.39	Asph	210125	3800750	1.967881799	down
MACS_PEAK_2138	41	19.39	Asph	210125	4210014	4.216923714	down
MACS_PEAK_2138	41	19.39	Asph	210125	2340121	2.216856718	down
MACS_PEAK_2138	41	19.39	Asph	210125	2650113	1.98672688	down
MACS_PEAK_2147	44	21.81	Bach2	333981	6760131	1.610206008	down
MACS_PEAK_2156	40	9.75	Melk	76573	130022	3.728048325	down
MACS_PEAK_2167	18	6.65	Zfp462	915547	6900100	3.295689821	up
MACS_PEAK_2168	25	9.69	Zfp462	829296	6900100	3.295689821	up
MACS_PEAK_2221	39	10.63	Jun	431608	840170	1.758324981	up
MACS_PEAK_2242	29	11.08	C8b	366661	6770687	3.325871229	down
MACS_PEAK_2246	18	7.88	Ppap2b	883189	4730280	4.089593887	down
MACS_PEAK_2247	25	12.12	Ppap2b	904542	4730280	4.089593887	down
MACS_PEAK_2248	55	21.2	Ppap2b	1075270	4730280	4.089593887	down
MACS_PEAK_2250	25	8.29	Yipf1	27047	6900300	1.664879918	up
MACS_PEAK_2265	44	25.45	Ppt1	45439	6450440	2.951411963	down
MACS_PEAK_2271	51	19.59	Trappc3	16279	6020609	5.257313251	down
MACS_PEAK_2271	51	19.59	Trappc3	16279	2030176	2.373871088	down
MACS_PEAK_2271	51	19.59	Trappc3	16279	6590373	3.298223019	down
MACS_PEAK_2282	65	25.4	Extl1	23391	2690136	3.756691933	down
MACS_PEAK_2289	20	7.27	Ephb2	157267	2100541	1.536585331	down
MACS_PEAK_2308	47	13.93	Spsb1	111063	2850093	2.480366468	down
MACS_PEAK_2314	30	8.13	Camta1	506255	2630458	2.402442694	down
MACS_PEAK_2326	22	9.69	Akap9	34121	2060195	1.569421291	up
MACS_PEAK_2337	39	10.67	Hgf	781543	102030184	2.11736989	up
MACS_PEAK_2342	30	13.33	Ptpn12	120046	6290609	2.747475624	down
MACS_PEAK_2342	30	13.33	Ptpn12	120046	6020725	3.96179533	down
MACS_PEAK_2342	30	13.33	Ptpn12	120046	6130746	5.469593525	down
MACS_PEAK_2352	23	8.48	Maea	43107	1170392	4.912212849	down
MACS_PEAK_2365	19	6.66	Slit2	1250504	106400110	2.072291613	up
MACS_PEAK_2365	19	6.66	Slit2	1250504	101190064	2.298761606	up
MACS_PEAK_2398	28	9.09	Chic2	202833	4540722	2.126761436	down
MACS_PEAK_2401	29	14.54	Exoc1	83783	102810438	2.457518816	down
MACS_PEAK_2409	30	13.33	Dck	279153	6900411	2.230581284	down
MACS_PEAK_2413	31	10.3	Nup54	39858	4060278	2.283360004	down
MACS_PEAK_2413	31	10.3	Nup54	39858	2360156	2.326942205	down
MACS_PEAK_2414	22	7.27	Nup54	58350	4060278	2.283360004	down
MACS_PEAK_2414	22	7.27	Nup54	58350	2360156	2.326942205	down
MACS_PEAK_2417	37	13.33	Rasgef1b	299831	5670524	1.734761953	down
MACS_PEAK_2427	32	13.93	Zfp326	160566	4010524	2.246153831	up
MACS_PEAK_2428	27	12.72	Zfp326	329301	4010524	2.246153831	up
MACS_PEAK_2431	37	12.12	Idua	8757	2690131	1.696261525	down
MACS_PEAK_2446	26	8.16	Pxn	17243	102370632	1.854892135	up
MACS_PEAK_2462	20	8.63	Fam101a	131525	5570040	2.988392353	down
MACS_PEAK_2463	27	9.69	Fam101a	148629	5570040	2.988392353	down
MACS_PEAK_2465	72	29.93	5930412G12Rik	435713	106100670	1.563243985	up
MACS_PEAK_2474	37	11.18	Wbscr17	685840	2630095	2.079356194	up
MACS_PEAK_2475	31	8.48	Wbscr17	878805	2630095	2.079356194	up
MACS_PEAK_2476	23	9.69	Wbscr17	922192	2630095	2.079356194	up
MACS_PEAK_2480	21	7.76	Gtf2i	80180	106290711	3.137578249	down
MACS_PEAK_2485	15	8.48	Rhbdd2	42342	870497	1.896586418	up
MACS_PEAK_2490	19	8.48	Rabl5	46848	770368	1.91449523	down
MACS_PEAK_2495	19	7.88	Micall2	116566	1050292	1.728844047	up
MACS_PEAK_2500	21	8.48	Fbxl18	81307	106100253	1.78158164	up
MACS_PEAK_2501	23	10.3	Fbxl18	77454	106100253	1.78158164	up
MACS_PEAK_2502	29	12.19	Actb	47093	5050047	3.328948975	up
MACS_PEAK_2502	29	12.19	Actb	47093	2030204	2.797592163	up
MACS_PEAK_2502	29	12.19	Actb	47093	2060215	6.623762131	up
MACS_PEAK_2506	21	9.09	Cdk8	83823	1050113	1.871616602	down
MACS_PEAK_2531	34	19.99	Glcci1	185717	2640082	1.538626552	down
MACS_PEAK_2537	35	16.96	Tcfec	811186	1170722	1.795204163	up
MACS_PEAK_2540	23	9.09	Ing3	145711	2060390	4.327756405	down
MACS_PEAK_2541	27	8.2	Ing3	125946	2060390	4.327756405	down
MACS_PEAK_2542	25	12.12	Ing3	76956	2060390	4.327756405	down
MACS_PEAK_2543	20	9.09	Ptprz1	298049	100780411	1.908211827	down
MACS_PEAK_2543	20	9.09	Ptprz1	298049	105900121	2.008227348	down
MACS_PEAK_2548	24	9.09	Zc3hc1	10639	5720040	4.774459839	down
MACS_PEAK_2548	24	9.09	Zc3hc1	10639	3520541	1.506136537	down
MACS_PEAK_2554	40	9.09	Mest	43495	6620292	1.687818408	down
MACS_PEAK_2554	40	9.09	Mest	43495	4780440	8.560221672	down
MACS_PEAK_2584	21	6.66	Hoxa2	19368	2120121	1.682744145	down
MACS_PEAK_2594	70	34.53	Gadd45a	105043	2900717	1.737415195	down
MACS_PEAK_2595	20	9.09	Gadd45a	133041	2900717	1.737415195	down
MACS_PEAK_2618	28	8.53	Hdac11	44279	2510242	1.870505929	up
MACS_PEAK_2621	63	26.89	Adamts9	160778	101580446	1.56271708	up
MACS_PEAK_2622	30	13.93	Adamts9	165405	101580446	1.56271708	up
MACS_PEAK_2629	15	7.27	Frmd4b	329006	6770736	1.907487988	up
MACS_PEAK_2630	22	6.66	Frmd4b	266119	6770736	1.907487988	up
MACS_PEAK_2631	111	45.72	Frmd4b	333071	6770736	1.907487988	up
MACS_PEAK_2658	33	13.93	Ccdc77	131299	780092	1.5072577	up
MACS_PEAK_2669	33	14.54	Lrp6	126589	5130064	1.753937602	down
MACS_PEAK_2687	45	14.22	Etnk1	94220	100510161	3.267770767	down
MACS_PEAK_2687	45	14.22	Etnk1	94220	106420402	1.760130048	down
MACS_PEAK_2687	45	14.22	Etnk1	94220	103990484	1.924120307	down
MACS_PEAK_2731	24	7.62	Chst8	117433	1940050	1.671821713	up
MACS_PEAK_2749	25	11.51	Tjp1	228888	6350184	1.732068419	up
MACS_PEAK_2750	48	23.63	Pcsk6	168560	520097	2.70586133	down
MACS_PEAK_2751	22	6.36	H47	56749	4070181	1.856332779	down
MACS_PEAK_2801	30	12.72	Mrpl48	27357	6100044	2.831741333	down
MACS_PEAK_2801	30	12.72	Mrpl48	27357	2260093	1.950306773	down
MACS_PEAK_2802	40	14.54	Mrpl48	46069	6100044	2.831741333	down
MACS_PEAK_2802	40	14.54	Mrpl48	46069	2260093	1.950306773	down
MACS_PEAK_2806	23	8.48	Stim1	166889	104810400	1.953275561	up
MACS_PEAK_2807	24	9.69	Dchs1	19807	105890152	1.511523962	up
MACS_PEAK_2807	24	9.69	Dchs1	19807	106510204	3.529207468	up
MACS_PEAK_2843	29	10.91	Fgfr2	522411	5570402	4.836047649	down
MACS_PEAK_2843	29	10.91	Fgfr2	522411	100780364	1.819450498	down
MACS_PEAK_2843	29	10.91	Fgfr2	522411	4670601	1.686376214	down
MACS_PEAK_2844	55	23.63	Fgfr2	465147	5570402	4.836047649	down
MACS_PEAK_2844	55	23.63	Fgfr2	465147	100780364	1.819450498	down
MACS_PEAK_2844	55	23.63	Fgfr2	465147	4670601	1.686376214	down
MACS_PEAK_2845	100	39.38	Fgfr2	456515	5570402	4.836047649	down
MACS_PEAK_2845	100	39.38	Fgfr2	456515	100780364	1.819450498	down
MACS_PEAK_2845	100	39.38	Fgfr2	456515	4670601	1.686376214	down
MACS_PEAK_2855	31	13.33	Ptpre	49173	1660286	1.759332657	up
MACS_PEAK_2860	45	14.22	Ebf3	228163	4200129	2.323424101	down
MACS_PEAK_2861	38	11.18	Glrx3	33267	70563	1.531902909	up
MACS_PEAK_2873	24	10.98	Ccnd1	162421	100380692	1.749664187	down
MACS_PEAK_2874	84	24.38	Ccnd1	254683	100380692	1.749664187	down

MACS_PEAK_2875	54	7.47	Tpcn2	77672	1690603	1.936539292		up
MACS_PEAK_2880	52	23.02	Arglu1	70798	6510398	1.500149727		up
MACS_PEAK_2882	88	30.01	Col4a1	213559	1740575	3.014300823		up
MACS_PEAK_2896	37	18.78	Erlin2	14026	5340603	2.231492758		down
MACS_PEAK_2897	32	12.12	Nrg1	786059	103060161	1.833201647		up
MACS_PEAK_2914	30	7.88	Sorbs2	336865	6350112	1.909857392		down
MACS_PEAK_2915	22	9.09	Sorbs2	362267	6350112	1.909857392		down
MACS_PEAK_2917	36	15.75	Irf2	224012	5390044	1.701186299		up
MACS_PEAK_2918	25	12.12	Irf2	257453	5390044	1.701186299		up
MACS_PEAK_2921	33	12.59	Cdkn2aip	74090	3060670	1.746379733		up
MACS_PEAK_2922	17	9.09	Cdkn2aip	97416	3060670	1.746379733		up
MACS_PEAK_2925	24	10.3	Gpm6a	2101572	1660044	2.084709644		down
MACS_PEAK_2929	27	11.51	Palld	232156	102060332	2.444493532		up
MACS_PEAK_2939	56	32.11	Csgalnact1	58634	5390746	1.569012284		up
MACS_PEAK_2941	39	19.99	Upf1	13570	1190750	1.780835986		up
MACS_PEAK_2941	39	19.99	Upf1	13570	430164	2.72619462		up
MACS_PEAK_2944	72	30.9	Tpm4	22806	3850072	5.901847839		down
MACS_PEAK_2944	72	30.9	Tpm4	22806	4920162	11.63345242		down
MACS_PEAK_2946	47	21.81	Ednra	277426	6900133	1.612930536		up
MACS_PEAK_2952	26	13.33	Smarca5	130599	6620050	3.157713652		up
MACS_PEAK_2955	23	8.44	Il15	631723	4560332	2.160155296		up
MACS_PEAK_2956	16	9.09	Zfp330	181502	1990504	1.561439037		down
MACS_PEAK_2968	22	12.72	Sall1	251309	5420020	1.546113729		down
MACS_PEAK_2973	26	12.72	Cdh8	2924032	2100348	1.914093852		up
MACS_PEAK_2974	22	10.3	Cdh8	2917124	2100348	1.914093852		up
MACS_PEAK_2975	24	10.3	Cdh8	2548126	2100348	1.914093852		up
MACS_PEAK_2976	20	7.88	Cdh8	2386197	2100348	1.914093852		up
MACS_PEAK_2978	43	20.6	Cdh8	3137195	2100348	1.914093852		up
MACS_PEAK_2979	17	7.27	Cdh5	1296556	5340487	2.503920078		up
MACS_PEAK_2980	28	7.62	Cdh5	1248705	5340487	2.503920078		up
MACS_PEAK_2981	19	7.27	Cdh5	1117617	5340487	2.503920078		up
MACS_PEAK_2982	24	11.51	Cdh5	1022979	5340487	2.503920078		up
MACS_PEAK_2983	49	14.1	Cdh5	876964	5340487	2.503920078		up
MACS_PEAK_2984	44	13.01	Cdh5	830726	5340487	2.503920078		up
MACS_PEAK_2985	57	27.87	Cdh5	786795	5340487	2.503920078		up
MACS_PEAK_2988	40	7.66	Slc12a4	14856	130731	1.859230638		up
MACS_PEAK_2995	19	10.3	Wwp2	59248	6550079	1.589534521		down
MACS_PEAK_2995	19	10.3	Wwp2	59248	1090102	5.120452404		down
MACS_PEAK_2996	39	10.16	Wwp2	83603	6550079	1.589534521		down
MACS_PEAK_2996	39	10.16	Wwp2	83603	1090102	5.120452404		down
MACS_PEAK_2997	77	25.49	Wwp2	86312	6550079	1.589534521		down
MACS_PEAK_2997	77	25.49	Wwp2	86312	1090102	5.120452404		down
MACS_PEAK_2998	36	13.93	Wwp2	108451	6550079	1.589534521		down
MACS_PEAK_2998	36	13.93	Wwp2	108451	1090102	5.120452404		down
MACS_PEAK_3007	21	6.66	Plcg2	162287	5720008	2.247225523		up
MACS_PEAK_3009	37	13.93	Gse1	165596	510273	1.679089785		up
MACS_PEAK_3021	60	29.69	Rhou	99421	2470706	2.100575924		up
MACS_PEAK_3038	121	33.05	Sesn3	386437	110687	2.475210667		down
MACS_PEAK_3042	19	8.48	Fat3	1494738	4670324	1.660501838		up
MACS_PEAK_3045	21	8.48	Slc44a2	16847	4670020	2.969364405		down
MACS_PEAK_3045	21	8.48	Slc44a2	16847	6590347	4.12567997		down
MACS_PEAK_3046	27	8.48	Anln	22231	1780113	1.848033309		down
MACS_PEAK_3088	116	25.88	Etfa	105354	50347	2.289352417		down
MACS_PEAK_3097	21	7.88	Neo1	254846	100730537	1.652311444		down
MACS_PEAK_3122	22	9.09	Tln2	293960	100730390	1.781723261		up
MACS_PEAK_3123	17	7.88	Tln2	151955	100730390	1.781723261		up
MACS_PEAK_3142	34	11.51	Lrrc1	101001	6180746	1.585395694		down
MACS_PEAK_3143	18	6.66	Col12a1	838246	100110731	1.992657542		down
MACS_PEAK_3144	24	12.12	Col12a1	792428	100110731	1.992657542		down
MACS_PEAK_3146	29	12.12	Lca5	148784	106130102	1.678013563		down
MACS_PEAK_3147	20	7.88	Ttk	202759	105890725	1.543180704		down
MACS_PEAK_3150	23	10.3	Syncrip	40132	3140113	1.773158789		down
MACS_PEAK_3150	23	10.3	Syncrip	40132	1690195	1.894646764		down
MACS_PEAK_3156	53	25.45	1190002N15Rik	1661132	4390040	4.486179829		down
MACS_PEAK_3163	30	7.27	Cep70	73928	1780546	1.532244563		down
MACS_PEAK_3179	50	8.49	Smarcc1	74177	7100047	2.587359428		down
MACS_PEAK_3179	50	8.49	Smarcc1	74177	5080019	1.68176949		down
MACS_PEAK_3191	29	11.08	Azi2	913267	2680463	1.792396307		down
MACS_PEAK_3193	59	19.39	Itga9	119407	670180	1.867954493		up
MACS_PEAK_3197	23	9.69	Vill	20213	1980504	2.05437851		down
MACS_PEAK_3198	21	9.69	Vill	10572	1980504	2.05437851		down
MACS_PEAK_3221	36	16.96	Atp6ap2	518218	4010059	6.724124908		down
MACS_PEAK_3221	36	16.96	Atp6ap2	518218	7100347	6.21543026		down
MACS_PEAK_3222	27	10.3	Bcor	249767	3940053	1.556888342		down
MACS_PEAK_3230	25	12.12	6720401G13Rik	164663	106220215	1.954547167		up
MACS_PEAK_3248	22	8.48	Pak3	113685	4210136	1.949670911		down

Supplementary Table 6. Number (No.) of zebrafish injected with the morpholinos and their respective phenotype.

	No. Injected	No. Alive at 24 hpf	Mild morphant	Severe morphant	Normal	Morpholino Sequence
Scrambled	173	155	12	0	143	5'CCTCTTACCTCAGTTA CAATTTATA– 3'
Tgfb2	188	140	82	45	13	5'GGAGGCTCAAGACGT ACAAGTTCAT3'
Fbxl18	210	187	100	68	19	5'GTTTCGATTTGATTAT TCATGGCGC3'
Tle3(Gro2)	188	154	77	48	29	5'CGGCCCTGTGGATACA TCTTGAAAT3'

Note: A p53 morpholino- 5'GACCTCCTCTCCACTAAACTACGAT3' was co-injected with all the morpholinos to reduce the off target effects.
